# Exploring Molecular Mechanisms of *Aloe barbadmsis* Miller on Diphenoxylate-Induced Constipation in Mice

**DOI:** 10.1155/2022/6225758

**Published:** 2022-05-06

**Authors:** Ruying Tang, Jianjun Zhang, Haipeng Nan, Ruilin Lv, Xiuhong Chen, Yao Liu, Xiangshan Wang, Linyuan Wang

**Affiliations:** ^1^School of Chinese Materia Medica, Beijing University of Chinese Medicine, Beijing, China; ^2^School of Traditional Chinese Medicine, Beijing University of Chinese Medicine, Beijing, China

## Abstract

*Aloe barbadensis* Miller (Aloe) known as a common succulent perennial herb had been traditionally used in constipation for more than 1,000 years. Aloe contained anthraquinones and other active compounds which had laxative effect and could modulate constipation. However, the therapeutic effects and mechanisms of aloe in constipation were still unclear. To explore the therapeutic effects and mechanisms of aloe in treating constipation, we employed network pharmacology, molecular docking, and mice experiments in this study. Our network pharmacology indicated that beta-carotene, sitosterol, campest-5-en-3beta-ol, CLR, arachidonic acid, aloe-emodin, quercetin, and barbaloin were the main active ingredients of aloe in treating constipation. Besides, the MAPK signaling pathway was the principal pathway utilized by aloe in treating constipation. Molecular docking results revealed that beta-carotene and sitosterol were acting as interference factors in attenuating inflammation by binding to an accessory protein of ERK, JNK, AKT, and NF-*κ*B p65. Otherwise, in vivo experiments, we used diphenoxylate-induced constipation mice model to explore the therapeutic effects and mechanisms of aloe. Results showed that aloe modulated the constipation mice by reducing the discharge time of first melena, improving the fecal conditions, increasing the gastric intestinal charcoal transit ratio, and improving the intestinal secretion in small intestine. Besides, aloe played an important regulation in promoting intestinal motility sufficiency and the levels of neurotransmitters balance with 5-HT, SP, and VIP on constipation mice. Moreover, aloe significantly inhibited the mRNA and proteins expressions of ERK, JNK, AKT and NF-*κ*B p65 in colon. Our study proved that aloe could reverse diphenoxylate-induced changes relating to the intestinal motility, intestinal moisture, and inhibition of the MAPK (ERK, JNK)/AKT/NF-*κ*B p65 inflammatory pathway. Our study provided experimental evidences of the laxative effect of aloe, which was beneficial to the further research and development of aloe.

## 1. Introduction

Constipation was an intestinal disease performing with clinical functional disorder. The course of constipation was long, and the condition was easy to repeat [1, 2]. Recently, with the improvement of the pace of life and the changes of lifestyle, such as sitting for a long time, staying up late, pressure, and many other factors had led to the emergence of constipation. In affected individuals, physical health, mental health, and social functioning were impacted [3]. Constipation had became one of the vexing problems for modern people. At present, using laxatives of chemical drugs to promote gastrointestinal movement was conventional treatment of constipation in clinic [4]. However, long-term use of these medicines to moisten the intestines and defecate was usually accompanied by clinical adverse reactions such as leading to drug dependence, damaging to stomach, and other symptoms [5]. Constipation had a long course and was prone to repeated attacks. Without drugs, it was easy to aggravate the course of constipation, but long-term use of stimulating laxatives was easy to induce other gastrointestinal diseases. Therefore, it was very important to find a drug that can improve the symptoms of constipation while at the same time had little adverse reactions.

In most cases, constipation was generally not considered to be a disease, it could be relieved by regulating diet, correcting some bad habits and taking alternative therapeutics including probiotics, herbal medicines, and so on [6]. A person with constipation was often treated with laxatives, drugs to promote gastrointestinal motility, adjustment of gastrointestinal flora, surgical treatment, etc. Although the treatment worked quickly, it was easy to cause drug-dependence, and some drugs will also cause other digestive system diseases [7]. Traditional Chinese Medicine (TCM) could work against constipation by internal and external application of Chinese medicine, acupuncture, cupping, massage, and other methods. Nowadays, based on the advantages of safety, low toxicity, and fewer side effects, some herbal medicines had unique superiority in treating constipation [8]. For example, *Aloe barbadensis Miller*, (Aloe) known as a common Chinese herb medicine, had good prospects in treating constipation [9]. In China, gel and whole leaf dry powder of aloe were considered as edible new resource food since the year of 2009 by Ministry of Health. With the biological activity and material basis of aloe constantly revealed, it had been widely used in the fields of health food, beauty, and medicine. External use of aloe could promote wound healing, moisturize the skin, performing the function of sterilization and anti-inflammatory wound healing. Oral medicine of aloe could enhance body immunity, anti-inflammatory and detoxification, increase appetite, invigorate blood circulation, and remove blood stasis to improve cardiovascular diseases [10]. Importantly, according to the previous studies reported, aloe was useful in relaxing the bowels, for its leaves and clear gel contain aloin and aloe emodin, which could promote the secretion of large intestine fluid, increase the activity of lipase, restore the dysfunctional autonomic nerve function of large intestine [11]. Although whole leaf dry powder of aloe was already proved to be laxative in daily life of people, molecular mechanisms of its laxative effect were still less well elucidated.

**Figure 1:**
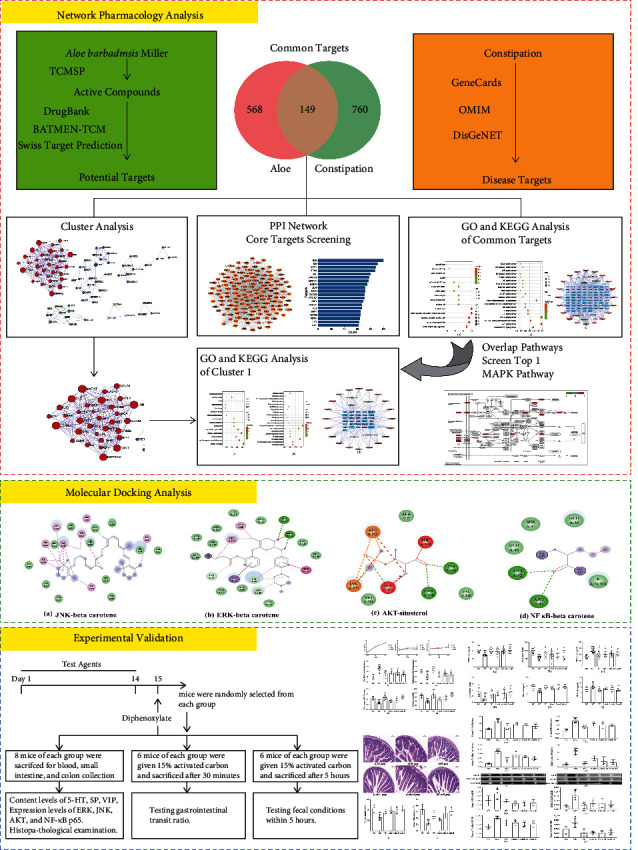
Analysis steps of *A. barbadmsis* Miller against constipation.

TCM had a significant curative effect, but it was difficult to reveal its molecular mechanisms for its diverse components and the complex interaction with human body. Nowadays, a network of “drug-component-target” constructed by network pharmacology was used commonly, which explained the pharmacological action of drugs and the pathogenesis of diseases [12]. Observing that, network pharmacology was a research method in line with the multicomponent and multitarget effects of TCM, it emphasized the multichannel regulation of signal pathways [13]. Besides, molecular docking was usually used in verifying the reliability of pharmacological predictions in networks. Nowadays, the application of network pharmacology, molecular docking, and experiment verification was a common mode for revealing the pharmacodynamic mechanisms of TCM [14]. Therefore, we explored the molecular mechanisms of aloe in constipation mice induced by diphenoxylate via network pharmacology, molecular docking, and experimental validation. In brief, we obtained the active components of aloe, then predicted the targets and pathways of aloe against constipation, and verified the active components and core targets of aloe through molecular docking, explored and verified the molecular mechanisms of aloe in constipation mice finally. Analysis steps were shown in Figure 1.

## 2. Materials and Methods

### 2.1. Network Pharmacology

#### 2.1.1. Acquisition of Ingredients Information in *A. barbadmsis* Miller

Active ingredients information of aloe was collected from TCMSP database and PubChem [15, 16]. Based on the active ingredients information obtained above, ingredients' targets were screened out from databases of DrugBank [17], PubChem [16], BATMEN-TCM [18], and Swiss Target Prediction [19]. After that the BATMEN-TCM score ≥20 was set, and the FDR under Benjamini–Hochberg procedure was used to set *P* ≤ 0.05, whereas in the Swiss Target prediction, selecting “*Homo sapiens*”, the probability ≥0.6 was set to screen out the targets [20]. Finally, the gene symbols of the active ingredients' targets were screened out from UniProt [21].

#### 2.1.2. Identification of Constipation Targets and the Acquisition of Common Targets

Constipation-associated target genes were collected from databases including GeneCards [22], OMIM [23], and DisGeNET [24], which were searched using the keyword “constipation.” All of these targets and the corresponding gene symbols were also screened out from UniProt. By comparing the targets of aloe and constipation, we acquired the common targets using Venny [25] for the next analysis.

#### 2.1.3. PPI Network Analysis

According to the previous study, we analyzed potential common targets using String by setting organism as “*Homo sapiens*” with a confidence score ≥0.4 [20]. Besides, using Cytoscape, PPI network, and active components-common targets-signal pathways networks were constructed and visualized. Then, top 20 hub genes were selected by the method of maximum neighborhood component (MNC) [20]. Finally, we used Metascape [26] to analyze clusters of common targets.

#### 2.1.4. GO and KEGG Analysis and Molecular Docking

DAVID online database was used to analyze with the adjusted *P* ≤ 0.05 [27]. Key signaling pathways were screened out by overlapping the KEGG enrichment pathways of cluster 1 targets and the KEGG enrichment pathways of common targets. Then, the structures of ingredients were obtained from the TCMSP database, and the crystal structures of the top 1 pathway relative proteins were obtained from RCSB PDB database. Finally, docking between ingredients and target proteins were performed with the help of software AutoDock 4.2.6 and the Discovery Studio [20].

### 2.2. Experimental Validation

#### 2.2.1. Animals

A total of 120 Kunming male mice 6-weeks old and weighing 18–22 g from SPF (Beijing) Biotechnology Co. Ltd., SCXK, Beijing, 2016-0002) were used for the experiment. Mice were maintained at controlled temperature of 23°C ± 2°C and humidity 60% ± 5%. Approval was obtained from the Ethics Committee in Beijing University of Chinese Medicine, BUCM-4-2019071502-3082.

#### 2.2.2. Drugs and Reagents

Whole leaf drying powder of aloe was purchased from Yunnan Wanlu Biological Co., Ltd (Lot number: 20180502, Yunnan, China). Quantitative analysis of aloe and its reference compounds information was shown in Table 1. Aloe was prepared as the final concentration of 15 mg/mL. Diphenoxylate (0.025 mg atropine and 2.5 mg diphenoxylate in one tablet) was prepared as the final concentration of 0.25 mg/mL. Phenolphthalein (0.5 mg phenolphthalein in one tablet, Lot number: 190706, Shanxi Hengruida Pharmaceutical Co., Ltd., Shanxi, China) was prepared as the final concentration of 1.5 mg/mL. These different solutions were stored at 4°C and heated to room temperature before use. The 15% activated carbon solution was prepared as previous research [28].

#### 2.2.3. Experimental Design

After 7 days acclimatization, mice were divided into six groups randomly as 20 mice per group. Constipation was induced in the mice by diphenoxylate as in previous research [29, 30]. The intragastric dose of mice was 0.2 mL/10 g and adjusted according to body weight (BW). The normal control group (NC) and the model control group (MC) were given distilled water; the positive control group (PC) was given phenolphthalein 30 mg/kg BW; Aloe low-dose group (Aloe-L) was given Aloe 75 mg/kg BW; Aloe middle-dose group (Aloe-M) was given Aloe 150 mg/kg BW; Aloe high-dose group (Aloe-H) was given Aloe 300 mg/kg BW.

Each group was administered in the abovementioned manner once daily for 14 consecutive days. On 15th day, diphenoxylate 5 mg/kg BW was used to induce constipation except in the NC group [29, 30]. Thirty minutes later, eight mice of each group were utilized for blood, small intestine tissue, and colon tissue collection. While the remaining 12 mice were given 15% activated carbon (0.5 mL/mouse) by gavage. Among them, six mice were sacrificed after thirty minutes later for testing gastrointestinal transit ratio, while the remaining six mice were used for testing fecal conditions within 5 hours.

#### 2.2.4. Body Weight Changes, Food Intake, Water Intake, and Fecal Conditions within 5 Hours of Mice

On day 1, day 7, and day 14, body weight, food, and water intake were recorded, respectively. Besides, the time of the first appearance of melena, fecal weight and fecal granule number within 5 hours were tested as in the previous researches [29]. Finally, feces within 5 hours were collected and weighed as “A,” then weighed again after drying for 3 hours, and recorded as “B”, and fecal moisture content percentage (%) = (A − B)/A × 100% [28].

#### 2.2.5. Propulsion of Activated Carbon in the Intestines

The remaining six mice of each group in “2.2.4.” were sacrificed 30 minutes later after giving 15% activated carbon. Then the entire small intestine was removed and pulled into a straight line to measure the length as “a,” while the stroke of activated carbon was measured as “b,” gastrointestinal transit ratio (%) = b/a × 100% [28].

#### 2.2.6. Histopathological Examination

The fixed colon tissue was immersed in 4% paraformaldehyde and embedded in paraffin wax for H&E. With the help of imaging system and image acquisition software, all images were photographed. Morphological structure was observed through a normal optical microscope (NIKON ECLIPSE CI, Japan), and then the villus length and muscle thickness were measured.

#### 2.2.7. Contents of Neurotransmitters in Serum and Colon

The blood samples of mice were collected by extracting eyeballs and centrifuged to get serum. After being sacrificed, colon tissue of mice was excised and immersed in −80°C liquid nitrogen for further analysis. Levels of 5-hydroxytryptamine (5-HT), substance P (SP), and vasoactive intestinal peptide (VIP) in serum and colon were tested (ELISA kits, Beijing Best way Biotechnology Co., Ltd., Beijing, China).

#### 2.2.8. Reverse Transcription Polymerase Chain Reaction

Fresh mice colon tissue weighing 100 mg was homogenized and the total RNA was extracted, and then the first strand of cDNA was synthesized. All the targeted parameters are shown in Table 2 [31–33]. Amplification of cDNA was performed by fluorescence quantitative PCR instrument (ABI, Stepone plus). GAPDH is an internal reference gene. The 2^−ΔΔCt^ method was used to assess the mRNA expressions including ERK, JNK, AKT, and NF-*κ*B p65.

#### 2.2.9. Western Blot Analysis

Total proteins of the colon were measured by a BCA kit (Thermo, MA, USA). Then 40 *μ*g of protein samples were mixed with equal volume of loading buffer which was separated and transferred onto PVDF membranes. Next, 5% defatted milk powder was used to blocked the membranes for 1 h and then separately incubated with anti-ERK, anti-JNK, anti-AKT, and anti-NF-*κ*B p65, respectively, at 4°C for a night. Following this, membranes were washed and incubated with secondary antibody. Finally, target bands were exposed and quantified [34].

#### 2.2.10. Statistical Analysis

Data were expressed as Means ± SD. Using SPSS 22.0 with ANOVA, or a nonparametric test for data processing was based on the normality test. The LSD method was adopted for comparisons between groups. The statistically significant difference for *P* value was *<*0.05.

## 3. Results

### 3.1. Aloe's Potential Target Prediction, Constipation Genes, and Common Targets PPI Analysis

**Figure 2:**
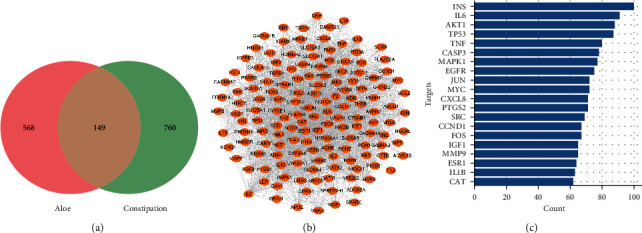
(a) Venn diagram of the overlap of aloe and constipation. (b) Common targets PPI network. (c) The top 20 hub genes of common targets.

In Table 3, eight active chemical ingredients of Aloe were obtained (OB ≥ 30% and DL ≥ 0.18) from TCMSP. Barbaloin, OB ＜ 30%, as in the previous study was verified for its effects on constipation, so that we got beta-carotene, sitosterol, campest-5-en-3beta-ol, CLR, arachidonic acid, aloe-emodin, quercetin, and barbaloin as the active ingredients of Aloe in treating constipation (ADME parameters for these compounds were screened after looking up the ingredients from TCMSP and this is shown in Table S1). Besides, as results shown in the Figure 2(a), Venn diagram shows 149 common targets results for the intersection of 717 aloe targets and 909 constipation targets (Tables S2, S3, and S4). In Figures 2(b)–2(c), top 20 core targets in the PPI network were also derived, among which the top five targets included INS, IL6, AKT1, TP53, and TNF.

### 3.2. Modules Enrichment Analysis and Core Target Screening

**Figure 3:**
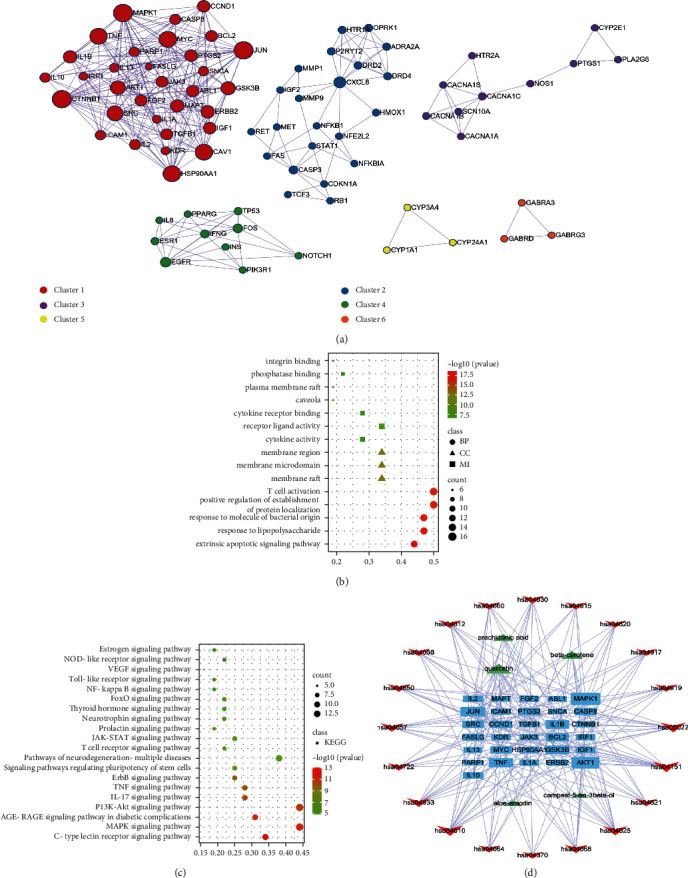
PPI network module clusters analysis and its cluster 1 analysis. (a) Common target PPI network module clusters. (b) Top 5 GO terms of PPI network module cluster 1 targets of aloe against constipation. (c) Top 20 KEGG signaling pathway enrichment of PPI network module cluster 1 targets of aloe against constipation. (d) Network of “key ingredients-protein targets-signaling pathways” of PPI network module cluster 1 targets about aloe against constipation.

Modules were considered to be biologically significant sets, especially the results of cluster 1 contributing to important biological significance. As shown in Figure 3(a), with the help of Metascape, we get six clusters after entering 149 common targets. Next, we obtained targets of cluster 1 for analysis. The results obtained were as 2183 GO terms, including 2091 BPs, 34 CCs, and 58 MFs, while KEGG analysis resulted in 150 pathways (Table S5). As shown in Figure 3(b), BPs mainly involved were extrinsically apoptotic, and responded to lipopolysaccharide and T cell activation, while the CCs mainly involved membrane raft, microdomain, and region. Moreover, the MFs mainly involved cytokine receptor binding, phosphatase binding, and integrin binding. For signaling pathways analysis suggested that, aloe-treated constipation concentrated on MAPK, AGE-RAGE, PI3K-Akt, and IL-17 signaling pathways as shown in Figure 3(c). Finally, the network of “key ingredients-protein targets-signaling pathways” of PPI network module cluster 1 targets was constructed as shown in Figure 3(d).

### 3.3. Common Targets GO, KEGG, and Pathway Network Analysis

**Figure 4:**
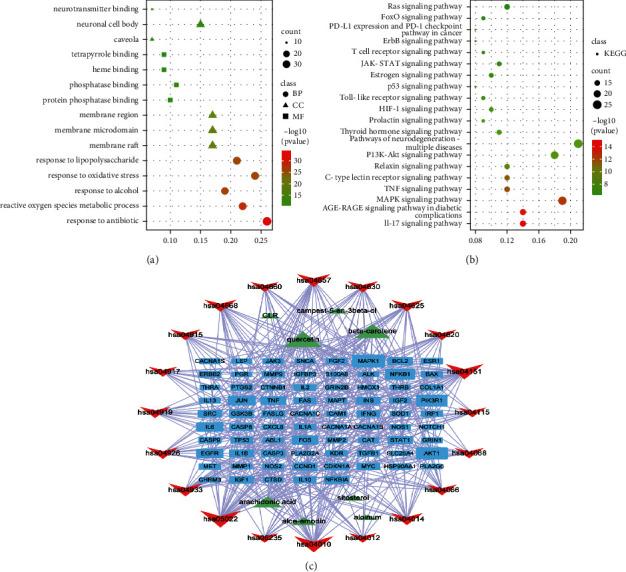
Common targets of GO, KEGG, and pathway network analysis. (a) Top five GO terms of common targets of aloe against constipation. (b) Top 20 KEGG signaling pathway enrichment of common targets of aloe against constipation. (c) Network of “key ingredients-protein targets-signaling pathways” of common targets about aloe against constipation.

As outcomes, 2804 GO terms were gained, including 207 BPs, 106 CCs, and 207 MFs, while KEGG pathway enrichment analysis screened out 171 signaling pathways (Table S6). As shown in Figure 4(a), BPs mainly involved response to antibiotic, alcohol, oxidative stress, and lipopolysaccharide, while the CCs mainly involved microdomain, region, caveola, and neuronal cell body. Moreover, the MFs mainly involved protein phosphatase binding, phosphatase binding, heme binding, tetrapyrrole binding, and neurotransmitter binding. In Figure 4(b), signaling pathways analysis revealed that the use of aloe against constipation was concentrated on IL-17, AGE-RAGE, MAPK, and TNF signaling pathways. Network of “key ingredients-protein targets-signaling pathways” of common targets was constructed as shown in Figure 4(c). After overlapping signaling pathways of Cluster 1 KEGG Enrichment pathways and Common Targets KEGG Enrichment pathways, we obtained important pathways including MAPK, AGE-RAGE, IL-17 signaling pathways, etc., in Table 4. Especially, MAPK pathway may be important for aloe in treating constipation according to the top one results of overlap signaling pathways.

### 3.4. Molecular Docking Analysis

**Figure 5:**
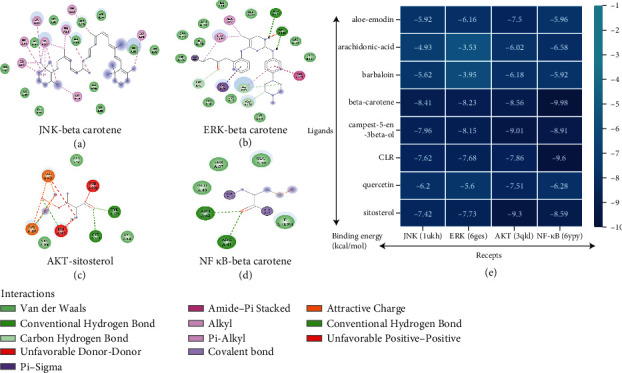
The minimum binding energy (kcal/mol) between active ingredients and proteins, the most stable of the binding mode which is shown in (a) JNK-beta carotene, (b) ERK-beta carotene, (c) AKT-sitosterol, (d) NF *κ*B- beta carotene. (e) Screening docking results between ligands (active compounds: aloe-emodin, arachidonic acid, barbaloin, beta-carotene, campest-5-en-3beta-ol, CLR, quercetin, sitosterol), and receptors (JNK, with PDB ID: 1ukh; ERK, with PDB ID: 6ges; AKT, with PDB ID: 3qkl; NF-*κ*B, with PDB ID: 6ypy).

From the above analysis it was concluded that MAPK signaling pathway may be an important mechanism of aloe in treating constipation. Therefore, we verified the binding mode of the aloe ingredients and the target proteins in MAPK signaling pathway as was illustrated in Figure 5(e). Figure 5(a)–5(d) shows that beta-carotene and sitosterol bound well to the ERK, JNK, AKT, and NF-*κ*B with the lowest value of the binding energy. Moreover, their binding patterns mainly involved hydrogen bonding. Results revealed that, among the eight active ingredients of aloe, beta-carotene and sitosterol might be the core active compounds and interference factors in attenuating inflammation by binding to an accessory protein of ERK, JNK, AKT, and NF-*κ*B.

### 3.5. Changes on Body Weight, Food Intake, Water Intake, First Black Excretion Time, Fecal Conditions within 5 Hours, and Fecal Moisture Content

**Figure 6:**
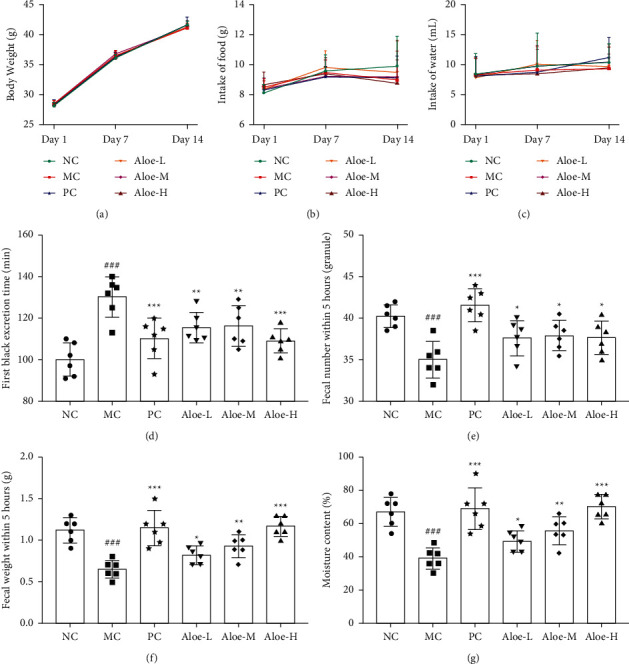
General status of each group: (a) Body weight, (b) Food intake, (c) Water intake, (d) First black excretion time, (e) Fecal number within 5 hours, (f) Fecal weight within 5 hours, and (g) Fecal moisture content in constipated mice induced by diphenoxylate. ^###^*P* < 0.001 versus NC group; ^*∗∗∗*^*P* < 0.001, ^*∗∗*^*P* < 0.01, ^*∗*^*P* < 0.05 versus MC group; *n* = 6.

Figures 6(a)–6(c)show that body weight, food, and water intake of mice on day 1, day 7, and day 14 had no significant changes in each group (*P* > 0.05). This meant that there existed a balance across the groups. So we randomly assigned mice to six groups to test the catharsis effect of aloe on constipation mice. In Figures 6(d)–6(g), the first black excretion time was significantly prolonged, while the fecal number, the fecal weight, and the moisture content of feces were decreased in the MC group (*P* < 0.001). Inversely, the above fecal condition was improved after orally treating with aloe (*P* < 0.001, or *P* < 0.01, or *P* < 0.05) (Figure 6).

### 3.6. Changes on Gastrointestinal Intestinal Transit Ratio

In MC group, gastric intestinal transit ratio decreased (*P* < 0.01), while this ratio increased (*P* < 0.05, or *P* < 0.01) in every test agent group as shown in Table 5.

### 3.7. Changes on Histological Alterations of Colon

**Figure 7:**
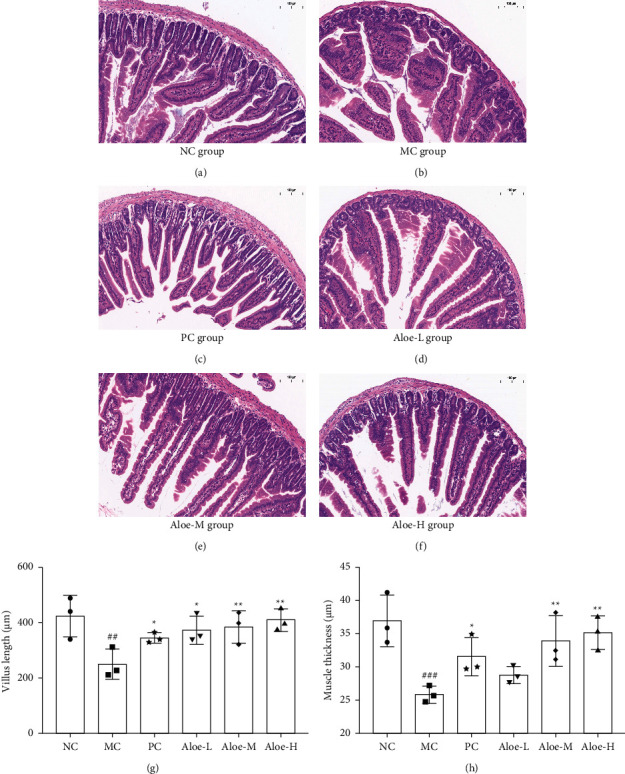
(a) to (f) Histological findings in the small intestine with H&E staining, magnification 10x, scale bar 100 *μ*M. (g) Villus length. (h) Muscle thickness. ^###^*P* < 0.001, ^##^*P* < 0.01 versus NC group; ^*∗∗*^*P* < 0.01, ^*∗*^*P* < 0.05 versus MC group; *n* = 3.

In Figures 7(a)–7(f), the small intestinal mucosa in NC group mice was smooth, and there were normal arterioles and venules in the submucosa and a lot of goblet cells existed. Besides, the muscular layer was composed of smooth muscle cells and the subserosa was intact, whereas, the small intestinal structure of the MC group was destroyed, the mucosa was damaged, and inflammatory cells infiltrated to the mucosa. After treating with the test agents, the epithelial cells, goblet cells, mucus secretion, and musculoskeletal thickness were increased, and the submucosal tissues of the small intestine tended to be intact (Table S7). As the results show in Figures 7(g)–7(h), the average length of the villus length and muscle thickness was dramatically shorter in MC group (*P* < 0.01, or *P* < 0.001). However, they were increased after treating with phenolphthalein, or aloe. Overall, these findings indicated that, aloe can improve the pathological state of small intestine in constipated mice.

### 3.8. Changes on Parameters of Serum and Colon

**Figure 8:**
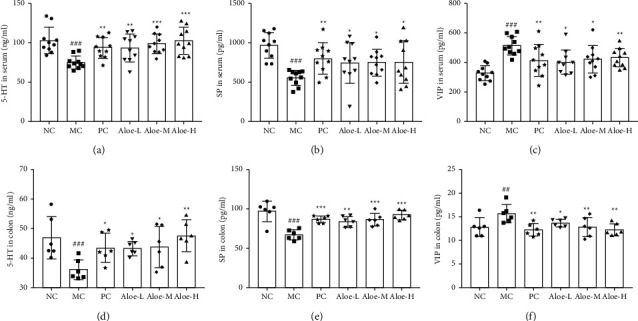
ELISA kits detection of serum and colon tissues. Contents of (a) 5-HT, (b) SP, (c) VIP in serum, *n* = 10. Contents of (d) 5-HT, (e) SP, (f) VIP in colon, *n* = 6. ^###^*P* < 0.001, ^##^*P* < 0.01 versus NC group; ^*∗∗∗*^*P* < 0.001, ^*∗∗*^*P* < 0.01, ^*∗*^*P* < 0.05 versus MC group.

In MC group, levels of 5-HT and SP were decreased (*P* < 0.01, or *P* < 0.001) in Figures 8(a), 8(b), 8(d), 8(e), while VIP was increased (*P* < 0.01, or *P* < 0.001) in (Figures 8(c), 8(f)). After treating with phenolphthalein and aloe, these disorders were improved (*P* < 0.001, or *P* < 0.01, or *P* < 0.05) (Table S8).

### 3.9. Changes on the mRNA Expression and Protein Expression of ERK, JNK, AKT, and NF-κB p65

**Figure 9:**
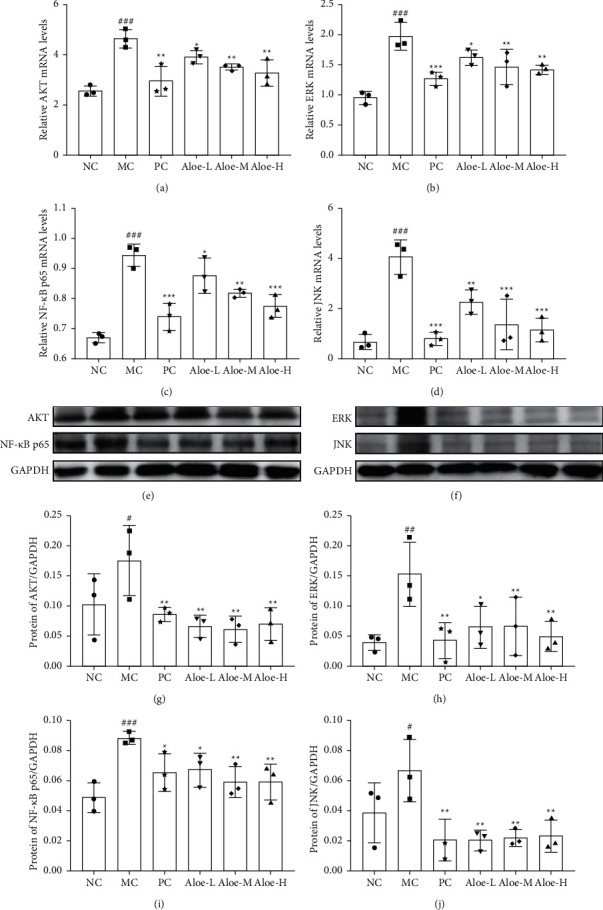
Relative mRNA expression of (a) AKT, (b) ERK, (c) NF-*κ*B p65, and (d) JNK in colon. Protein expression of (e)–(g) AKT, (e)–(i) NF-*κ*B p65, (f)–(h) ERK, and (f)–(j) JNK in colon. ^###^*P* < 0.001, ^##^*P* < 0.01, ^#^*P* < 0.05 versus NC group; ^*∗∗∗*^*P* < 0.001, ^*∗∗*^*P* < 0.01, ^*∗*^*P* < 0.05 versus MC group; *n* = 3.

**Figure 10:**
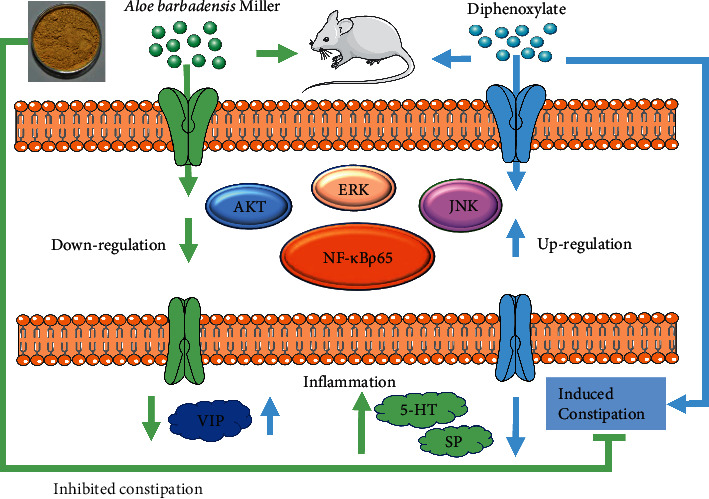
*A. barbadmsis* Miller against constipation by inhibiting the MAPK inflammatory pathway.

According to the predicted results, MAPK inflammatory pathway put outstanding in Aloe against constipation. Thus, we designed to examine this pathway in the colon tissue of constipation mice induced by diphenoxylate. As shown in Figures 9(a)–9(d), mRNA expressions of ERK, JNK, AKT, and NF-*κ*B p65 in colons of the MC group were significantly increased (*P* <0.001), while phenolphthalein and aloe could reverse the abnormal increasing expressions (P <0.001, P <0.01, P <0.05) (Table S9). Besides, shown in Figures 9(e)–9(j), protein quantitative value of western blot analysis revealed that phenolphthalein and Aloe could decrease the protein expression levels of ERK, JNK, AKT, and NF-*κ*B p65 (P <0.001, P <0.01, P <0.05) (Table S10). Briefly, Aloe treated constipation mice by inhibiting the MAPK inflammatory pathway, and the mechanism diagram of our study is shown in Figure 10.

## 4. Discussion

Due to insufficient intestinal power and the imbalance of intestinal mucus and water secretion, constipation appeared among people and clinically manifested as abdominal pain, abdominal distention, reduced frequency of defecation, dry stool, and difficult emptying [35, 36]. As was known that diphenoxylate often used as a modeling agent in constipation mice or rat model for its side effects that it could lead to constipation by inhibiting peristalsis, delaying the passage of intestinal contents, and increasing the absorption of water in the intestine [37]. In our experiment, constipation mice induced by diphenoxylate was used according to previous studies [38, 39]. Aloe was usually taken orally to resolve heat and diminish inflammation to treat constipation [40]. Our study demonstrated that aloe could significantly reverse these gastrointestinal changes to treat constipation. Besides, aloe could alleviate constipation symptoms by upregulating the contents of 5-HT and SP, while downregulating the level of VIP in the serum and the colon. What's more, according to the network pharmacology and molecular docking screening results, aloe significantly inhibited proteins expression and the mRNA levels of ERK, JNK, AKT, and NF-*κ*B p65 in our experiment validation, which in turn inhibited inflammatory signal transduction. Our results suggested that mechanisms of aloe against constipation may work by inhibiting the MAPK inflammatory pathway in constipation mice induced by diphenoxylate (Figure 10).

Anthraquinones such as aloe-emodin and barbaloin were the main components of laxation of aloe, with which we could treat constipation through effectively strengthening the intestinal peristalsis and reducing the reabsorption of water of intestinal wall [41]. Besides, the present study suggested that active compound of aloe quercetin also exerted a protective effect against constipation [42, 43]. Different from other laxatives, aloe had a mild purgative effect, and it cold improve the body's immunity and supplement a variety of nutrients [44]. However, the existing researches of aloe mainly focused on anti-inflammatory [45], anti-colorectal cancer [46], and wound healing [47], while its mechanisms of laxative action were rarely discussed. From the aspect of laxative effect of aloe, our study explained its laxative action and mechanisms. Importantly, excitatory neurotransmitters 5-HT and SP, as well as inhibitory neurotransmitters VIP were closely correlated to the intestinal motility and intestinal moisture in constipation which were the important indexes for clinical evaluation of constipation [48]. 5-HT and SP could promote gastrointestinal peristalsis and increase the frequency of smooth muscle contraction [49, 50]. While VIP was an important peptide active substance, which was widely distributed in gastrointestinal tract, liver, bile, pancreas, etc., and was very sensitive in the body relating to many diseases of digestive tract [45, 51]. Additionally, MAPK(ERK, JNK) and AKT acted as upstream kinases of NF-*κ*B p65 by downregulating NF-*κ*B p65 and could reduce intestinal inflammation and regulate the metabolism of intestinal water in constipation mice [46, 50]. Consistent with the results of previous studies of constipation, our studies demonstrated that aloe could be used against constipation by balancing levels of 5-HT, SP, and VIP, and inhibiting the MAPK(ERK, JNK)/AKT/NF-*κ*B p65 inflammatory signaling pathway. Summing up we could conclude that active compounds such as aloe-emodin, barbaloin, and quercetin, etc., of aloe could treat constipation by regulating the intestinal motility, intestinal moisture, and diminishing inflammation.

In our studies, through the theoretical predictions combining with the experimental verification of mice in vivo, the laxative effect and mechanism of aloe against constipation were demonstrated, which provided evidence for guiding the clinical application of aloe. Favorably, our study had explained the TCM efficacy and active ingredients of aloe in clearing heat and eliminating inflammation through modern mechanism. The potential components of aloe and the pharmacodynamic mechanisms of laxative effects of it were revealed, which provided references for the rational application of aloe against constipation and experimental evidences for the in-depth studies of aloe in treating constipation. Briefly, through network pharmacology, molecular docking prediction, and experiment verification, our study comprehensively revealed the defecation effect and mechanism of aloe which act as the characters of multicomponents and multitargets. Especially, the laxation mechanisms of aloe were screened out by network pharmacology through the intersection of pathway enrichment results of cluster 1 targets and common targets. The pathways screening results of aloe were more relevant and reliable in treating constipation. Finally, in the mice experimental verification, intestinal neurotransmitters 5-HT, SP, and VIP indicators were tested which were also commonly tested in clinical laxative projects. These provided relevant experimental data reference for aloe in clinical treatment of constipation. However, our study only selected the top one prediction pathway of the network pharmacology results for verification, other pathways need to be further explored. In addition, the intestinal flora is increasingly mature in the application of TCM in treating gastrointestinal diseases, so we may be able to conduct in-depth research on laxative effects of aloe from the perspective of intestinal flora in the next.

## 5. Conclusion

In conclusion, aloe had laxative eﬀects. First, network pharmacology and molecular docking revealed that AMPK signaling pathway was the principal pathway utilized by aloe against constipation, and beta-carotene and sitosterol might act as interference factors in attenuating inflammation by binding to an accessory protein of ERK, JNK, AKT, and NF-*κ*B p65. Second, in vivo experiments showed that aloe could increase intestinal motility and fecal water content to promote defecation and improve fecal condition in constipated mice. Besides, aloe also repaired the damaged intestinal structure of constipation mice and promoted the normal secretion of intestinal mucus as well. In addition, aloe played an important regulation in promoting intestinal motility sufficiency in the levels of neurotransmitters balance of 5-HT, SP, and VIP in serum and colon. Moreover, aloe significantly inhibited the mRNA and protein expressions of the ERK, JNK, AKT, and NF-*κ*B p65 in colon. Results proved that aloe could reverse diphenoxylate-induced changes relating to the intestinal motility, intestinal moisture, and inhibiting the MAPK(ERK, JNK)/AKT/NF-*κ*B p65 inflammatory pathway. Our study provided experimental evidences for the mechanisms of Aloe intreating constipation. It is high time to explore the therapeutic mechanisms of aloe in treating constipation from other aspects in the future, which can guide the clinical application among people.

## Figures and Tables

**Table 1:** Quantitative analysis of aloe and its reference compounds.

Compound	Content
Barbaloin	2.68 (g/100 g)
Total anthraquinone (quantitated by 1, 8-dihydroxyanthraquinone)	368 (mg/100 g)
Moisture content	4.18 (%)
Ash content	6.78 (%)

**Table 2:** Information of primers.

Primer name	Sequence (5′ to 3′)
ERK	Forward: CCGCTTGCCTCATTAAAGCC
	Reverse: TTAGACGTGGCAGCTTGGTT
JNK	Forward: CATGGCAGAAATGGTCCTCCA
	Reverse: TCTGCAGATGGTGTTCCTAGC
AKT	Forward: CCAAGCACCGTGTGACCATGAA
	Reverse: TGGCGACGATGACCTCCTTCTT
NF-*κ*B p65	Forward: ACCTGTTCCAAAGAGCACCCA
	Reverse: GGTCTGTGAACACTCCTGGGTC
GAPDH	Forward: CTGGAGAAACCTGCCAAGTATG
	Reverse: GGTGGAAGAATGGGAGTTGCT

**Table 3:** Information of ingredients in *A. barbadmsis* Miller.

Drug source	Molecule ID	Molecule name	OB (%)	DL
Aloe	MOL002773	Beta-carotene	37.18	0.58
	MOL000359	Sitosterol	36.91	0.75
	MOL005043	Campest-5-en-3beta-ol	37.58	0.71
	MOL000953	CLR	37.87	0.68
	MOL001439	Arachidonic acid	45.57	0.20
	MOL000471	Aloe-emodin	83.38	0.24
	MOL000098	Quercetin	46.43	0.28
	MOL005051	Barbaloin	22.18	0.71

**Table 4:** Overlap signaling pathways of Cluster 1 KEGG Enrichment pathways and Common Targets KEGG Enrichment pathways.

Pathway ID	Description	*P* value	Relevant gene
hsa04010	MAPK signaling pathway	1.36E-12	AKT1/FASLG/ERBB2/FGF2/IGF1/IL1A/IL1B/JUN/KDR/MAPT/MYC/MAPK1/TGFB1/TNF
hsa04933	AGE-RAGE signaling pathway in diabetic complications	2.73E-12	AKT1/CCND1/BCL2/ICAM1/IL1A/IL1B/JUN/MAPK1/TGFB1/TNF
hsa04657	IL-17 signaling pathway	5.92E-11	CASP8/GSK3B/HSP90AA1/IL1B/IL13/JUN/MAPK1/PTGS2/TNF
hsa04668	TNF signaling pathway	2.92E-10	AKT1/CASP8/ICAM1/IL1B/IRF1/JUN/MAPK1/PTGS2/TNF
hsa05022	Pathways of neurodegeneration-multiple diseases	1.16E-07	FASLG/BCL2/CASP8/CTNNB1/GSK3B/IL1A/IL1B/MAPT/MAPK1/PTGS2/SNCA/TNF
hsa04917	Prolactin signaling pathway	2.57E-07	AKT1/CCND1/GSK3B/IRF1/MAPK1/SRC
hsa04919	Thyroid hormone signaling pathway	3.49E-07	AKT1/CCND1/CTNNB1/GSK3B/MYC/MAPK1/SRC
hsa04620	Toll-like receptor signaling pathway	2.71E-06	AKT1/CASP8/IL1B/JUN/MAPK1/TNF
hsa04915	Estrogen signaling pathway	1.39E-05	AKT1/BCL2/HSP90AA1/JUN/MAPK1/SRC

**Table 5:** Gastrointestinal intestinal charcoal transit ratio in diphenoxylate constipated mice.

Groups	Small intestine length (cm)	Charcoal meal transferred length (cm)	Gastric charcoal transit ratio (%)
NC group	45.43 ± 2.10	56.65 ± 1.62	80.21 ± 3.24
MC group	41.60 ± 2.16	56.18 ± 2.71	74.06 ± 2.24^##^
PC group	44.63 ± 2.91	56.28 ± 1.89	79.37 ± 5.81^*∗*^
Aloe-L group	44.50 ± 3.18	55.92 ± 2.59	79.56 ± 3.69^*∗*^
Aloe-M group	45.28 ± 1.46	56.38 ± 2.29	80.35 ± 1.88^*∗∗*^
Aloe-H group	45.03 ± 1.31	55.92 ± 2.17	80.59 ± 2.51^*∗∗*^

^##^ *P* < 0.01 versus NC group; ^*∗∗*^*P* < 0.01, ^*∗*^*P* < 0.05 versus MC group; *n* = 6.

## Data Availability

The data used to support the findings of this study are included in the article and the supplementary information files, or available from the authors upon request.

## Supplementary Materials

Supplementary Materials
*Table S1*. Active ingredients of Aloe. *Table S2*. Potential targets related to active ingredients. *Table S3*. potential targets related to constipation. *Table S4*. Common targets related to active ingredients. *Table S5*. *Table S5-1*. Detailed information of BP enrichment of PPI network cluster 1 targets; *Table S5-2*. Detailed information of CC enrichment of PPI network cluster 1 targets; *Table S5-3*. Detailed information of MF enrichment of PPI network cluster 1 targets; *Table S5-4*. Detailed information of KEGG pathways enrichment of PPI network cluster 1 targets. *Table S6. Table S6-1*. Detailed information of BP enrichment of common targets; *Table S6-2*. Detailed information of CC enrichment of common targets; *Table S6-3*. Detailed information of MF enrichment of common targets; *Table S6-4*. Detailed information of KEGG pathways enrichment of common targets. *Table S7*. Original images of H&E staining in colon of three repeats in each group. *Table S8*. Raw data of 5-HT, SP, and VIP in serum and colon determined by ELISA kits. *Table S9*. Raw data of NF-κB p65, AKT, ERK, and JNK in colon determined by RT-PCR method. *Table S10*. Original images of ERK, JNK, AKT, and NF-κB p65 in colon of Western Blot, and its raw data quantification.
