# Supplementary material for: Exploring Molecular Mechanisms of Aloe barbadmsis Miller on Diphenoxylate-Induced Constipation in Mice
Source: Evid Based Complement Alternat Med. 2022 May 6;2022:6225758. doi: 10.1155/2022/6225758 (PMC9106447; doi:10.1155/2022/6225758)

Raw data and Original images of western blot

Data of NF-κB p65/GAPDH ratio:

groups	IntDen data of NF-κB p65-1	IntDen data of GAPDH-1	NF-κB p65-1/GAPDH-1 ratio	IntDen data of NF-κB p65-2	IntDen data of GAPDH-2	NF-κB p65-2/GAPDH-2 ratio	IntDen data of NF-κB p65-3	IntDen data of GAPDH-3	NF-κB p65-3/GAPDH-3 ratio
NC group	0.0550	1.1630	0.0473	0.0900	2.2480	0.0400	0.0460	0.7740	0.0594
MC group	0.1320	1.4170	0.0932	0.2020	2.3180	0.0871	0.0920	1.0840	0.0849
PC group	0.1070	1.6830	0.0636	0.1260	2.3360	0.0539	0.1080	1.3720	0.0787
Aloe-L group	0.1300	1.8570	0.0700	0.1340	2.1750	0.0616	0.1000	1.4980	0.0668
Aloe-M group	0.0940	1.9140	0.0491	0.1380	2.1470	0.0643	0.0650	1.4390	0.0452
Aloe-H group	0.0980	1.6060	0.0610	0.1540	2.3780	0.0648	0.0500	1.5100	0.0331
Aloe-L group	0.0580	1.4980	0.0387	0.1620	2.0050	0.0808	0.1400	1.6520	0.0847
Aloe-M group	0.1090	1.7970	0.0607	0.1730	2.2380	0.0773	0.0950	1.6480	0.0576
Aloe-H group	0.0610	2.0400	0.0299	0.1710	2.4090	0.0710	0.1520	1.6010	0.0949

Data of NF-κB p65/GAPDH ratio:

group	number	NF-κB p65/GAPDH ratio
NC group	1	0.0473
NC group	2	0.0400
NC group	3	0.0594
MC group	1	0.0932
MC group	2	0.0871
MC group	3	0.0849
PC group	1	0.0636
PC group	2	0.0539
PC group	3	0.0787
Aloe-L group	1	0.0544
Aloe-L group	2	0.0712
Aloe-L group	3	0.0758
Aloe-M group	1	0.0549
Aloe-M group	2	0.0708
Aloe-M group	3	0.0514
Aloe-H group	1	0.0455
Aloe-H group	2	0.0679
Aloe-H group	3	0.0640

Data of AKT/GAPDH ratio:

groups	IntDen data of AKT-1	IntDen data of GAPDH-1	AKT-1/GAPDH-1 ratio	IntDen data of AKT-2	IntDen data of GAPDH-2	AKT-2/GAPDH-2 ratio	IntDen data of AKT-3	IntDen data of GAPDH-3	AKT-3/GAPDH-3 ratio
NC group	0.1380	1.1630	0.1187	0.1020	2.2480	0.0454	0.1110	0.7740	0.1434
MC group	0.2670	1.4170	0.1884	0.2590	2.3180	0.1117	0.2440	1.0840	0.2251
PC group	0.1490	1.6830	0.0885	0.1720	2.3360	0.0736	0.1310	1.3720	0.0955
Aloe-L group	0.0990	1.8570	0.0533	0.0990	2.1750	0.0455	0.1280	1.4980	0.0854
Aloe-M group	0.1000	1.9140	0.0522	0.0960	2.1470	0.0447	0.1450	1.4390	0.1008
Aloe-H group	0.1400	1.6060	0.0872	0.0610	2.3780	0.0257	0.1450	1.5100	0.0960
Aloe-L group	0.1550	1.4980	0.1035	0.0880	2.0050	0.0439	0.1050	1.6520	0.0636
Aloe-M group	0.1520	1.7970	0.0846	0.0630	2.2380	0.0282	0.0910	1.6480	0.0552
Aloe-H group	0.2110	2.0400	0.1034	0.1390	2.4090	0.0577	0.0800	1.6010	0.0500

Data of AKT/GAPDH ratio:

group	number	AKT/GAPDH ratio
NC group	1	0.1187
NC group	2	0.0454
NC group	3	0.1434
MC group	1	0.1884
MC group	2	0.1117
MC group	3	0.2251
PC group	1	0.0885
PC group	2	0.0736
PC group	3	0.0955
Aloe-L group	1	0.0784
Aloe-L group	2	0.0447
Aloe-L group	3	0.0745
Aloe-M group	1	0.0684
Aloe-M group	2	0.0364
Aloe-M group	3	0.0780
Aloe-H group	1	0.0953
Aloe-H group	2	0.0417
Aloe-H group	3	0.0730

Data of ERK/GAPDH ratio:

groups	IntDen data of ERK-1	IntDen data of GAPDH-1	ERK-1/GAPDH-1 ratio	IntDen data of ERK-2	IntDen data of GAPDH-2	ERK-2/GAPDH-2 ratio	IntDen data of ERK-3	IntDen data of GAPDH-3	ERK-3/GAPDH-3 ratio
NC group	0.0550	1.1630	0.0473	0.1000	2.2480	0.0445	0.0180	0.7740	0.0233
MC group	0.3020	1.4170	0.2131	0.2573	2.3180	0.1110	0.1450	1.0840	0.1338
PC group	0.1050	1.6830	0.0624	0.1340	2.3360	0.0574	0.0100	1.3720	0.0073
Aloe-L group	0.1170	1.8570	0.0630	0.1080	2.1750	0.0497	0.0750	1.4980	0.0501
Aloe-M group	0.1780	1.9140	0.0930	0.1150	2.1470	0.0536	0.0040	1.4390	0.0028
Aloe-H group	0.1710	1.6060	0.1065	0.0410	2.3780	0.0172	0.0410	1.5100	0.0272
Aloe-L group	0.2130	1.4980	0.1422	0.1200	2.0050	0.0599	0.0320	1.6520	0.0194
Aloe-M group	0.2420	1.7970	0.1347	0.1790	2.2380	0.0800	0.0490	1.6480	0.0297
Aloe-H group	0.1010	2.0400	0.0495	0.1490	2.4090	0.0619	0.0530	1.6010	0.0331

Data of ERK/GAPDH ratio:

group	number	ERK/GAPDH ratio
NC group	1	0.0473
NC group	2	0.0445
NC group	3	0.0233
MC group	1	0.2131
MC group	2	0.1110
MC group	3	0.1338
PC group	1	0.0624
PC group	2	0.0574
PC group	3	0.0073
Aloe-L group	1	0.1026
Aloe-L group	2	0.0548
Aloe-L group	3	0.0347
Aloe-M group	1	0.1138
Aloe-M group	2	0.0668
Aloe-M group	3	0.0163
Aloe-H group	1	0.0780
Aloe-H group	2	0.0395
Aloe-H group	3	0.0301

Data of JNK/GAPDH ratio:

groups	IntDen data of JNK-1	IntDen data of GAPDH-1	JNK-1/GAPDH-1 ratio	IntDen data of JNK-2	IntDen data of GAPDH-2	JNK-2/GAPDH-2 ratio	IntDen data of JNK-3	IntDen data of GAPDH-3	JNK-3/GAPDH-3 ratio
NC group	0.0180	1.1630	0.0155	0.1090	2.2480	0.0485	0.0400	0.7740	0.0517
MC group	0.0680	1.4170	0.0480	0.2060	2.3180	0.0889	0.0680	1.0840	0.0627
PC group	0.0130	1.6830	0.0077	0.0820	2.3360	0.0351	0.0250	1.3720	0.0182
Aloe-L group	0.0210	1.8570	0.0113	0.0480	2.1750	0.0221	0.0330	1.4980	0.0220
Aloe-M group	0.0290	1.9140	0.0152	0.0400	2.1470	0.0186	0.0210	1.4390	0.0146
Aloe-H group	0.0500	1.6060	0.0311	0.0590	2.3780	0.0248	0.0300	1.5100	0.0199
Aloe-L group	0.0580	1.4980	0.0387	0.0490	2.0050	0.0244	0.0050	1.6520	0.0030
Aloe-M group	0.0740	1.7970	0.0412	0.0470	2.2380	0.0210	0.0350	1.6480	0.0212
Aloe-H group	0.0810	2.0400	0.0397	0.0280	2.4090	0.0116	0.0190	1.6010	0.0119

Data of JNK/GAPDH ratio:

group	number	JNK/GAPDH ratio
NC group	1	0.0155
NC group	2	0.0485
NC group	3	0.0517
MC group	1	0.0480
MC group	2	0.0889
MC group	3	0.0627
PC group	1	0.0077
PC group	2	0.0351
PC group	3	0.0182
Aloe-L group	1	0.0250
Aloe-L group	2	0.0233
Aloe-L group	3	0.0125
Aloe-M group	1	0.0282
Aloe-M group	2	0.0198
Aloe-M group	3	0.0179
Aloe-H group	1	0.0354
Aloe-H group	2	0.0182
Aloe-H group	3	0.0159

Annotation:
The original images of western blot. The strips from left to right are as followings:
the normal control group (NC),
the model control group (MC),
the positive control group (PC),
Aloe low dose group (Aloe-L) repeat 1,
Aloe middle dose group (Aloe-M) repeat 1,
Aloe hight dose group (Aloe-H) repeat 1,
Aloe low dose group (Aloe-L) repeat 2,
Aloe middle dose group (Aloe-M) repeat 2,
Aloe hight dose group (Aloe-H) repeat 2.

NF-κB p65- repeat 1

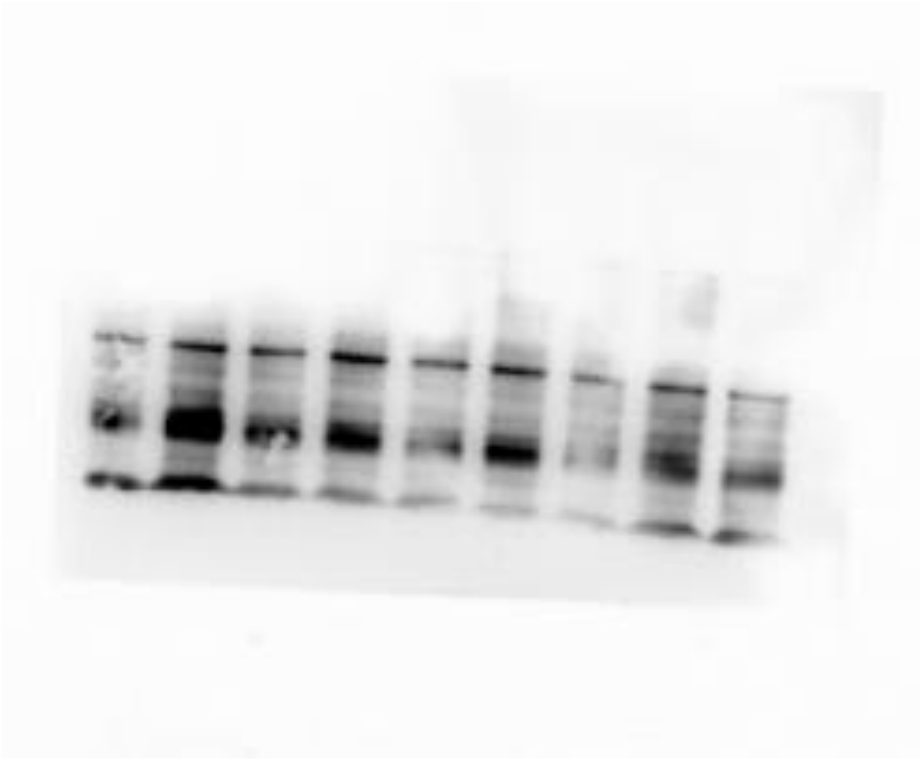

NF-κB p65- repeat 2

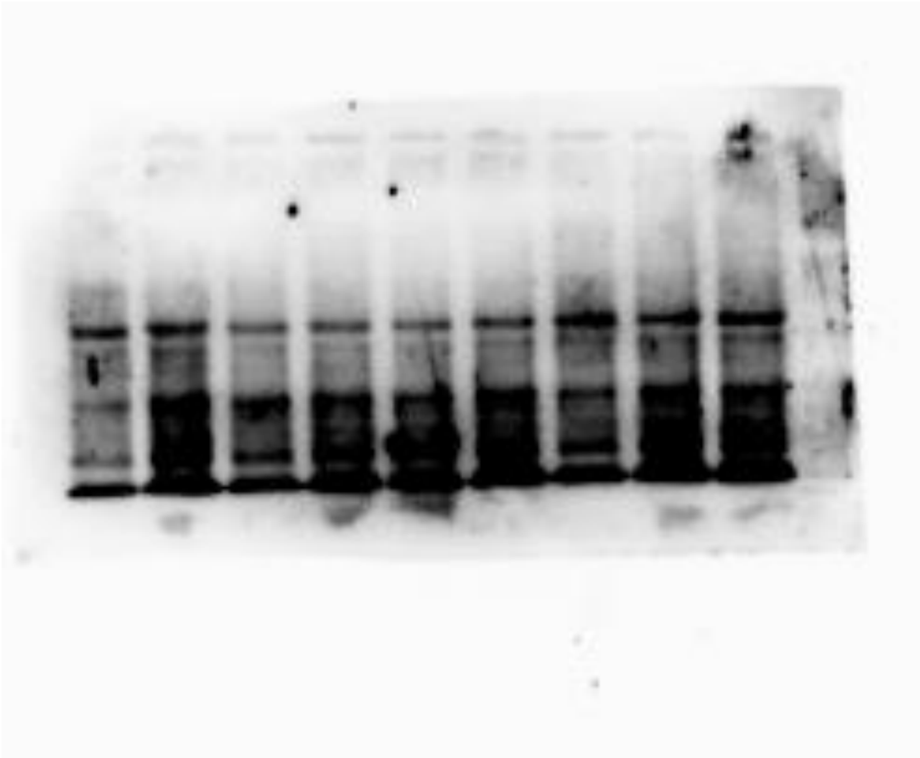

NF-κB p65- repeat 3

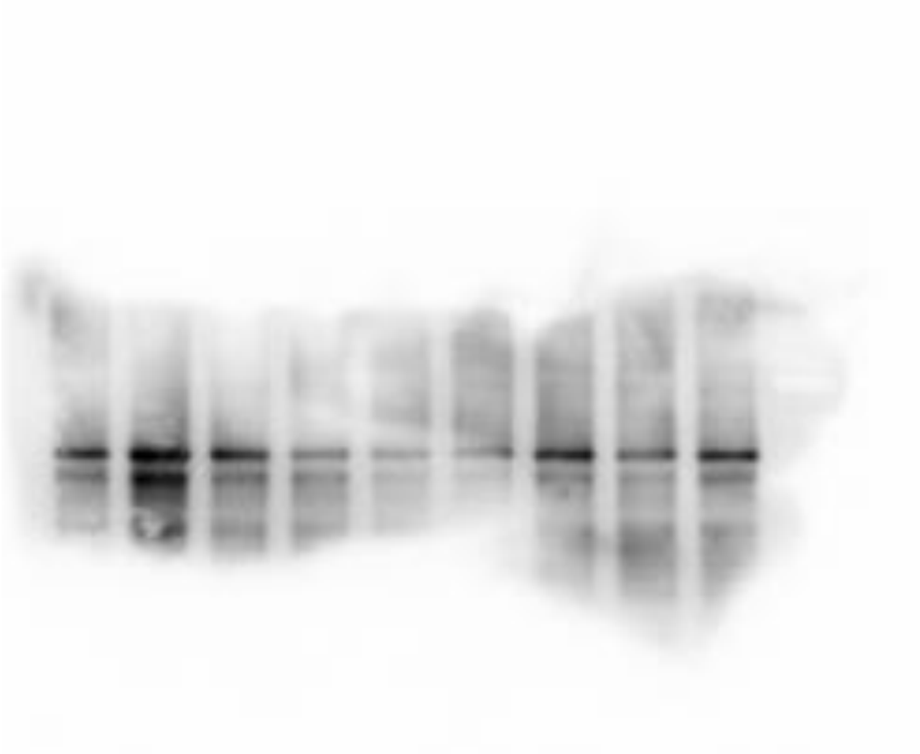

AKT-repeat 1

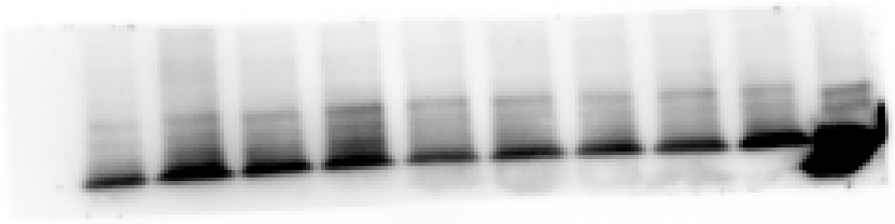

AKT- repeat 2

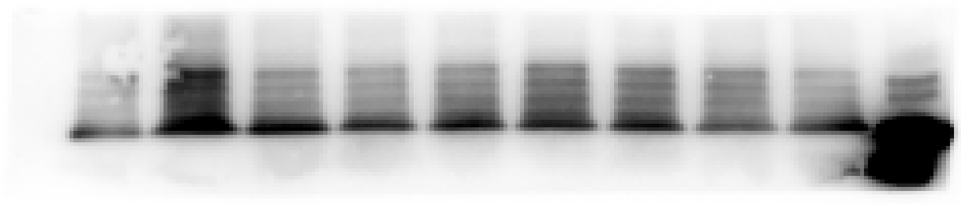

AKT- repeat 3

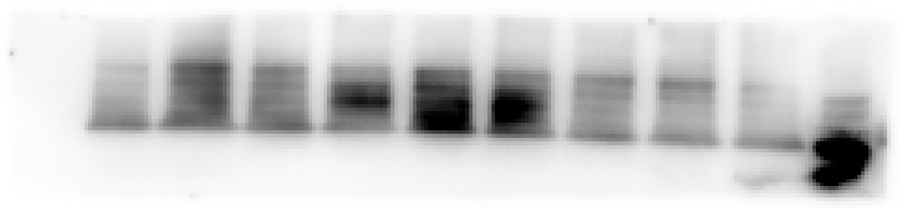

ERK- repeat 1

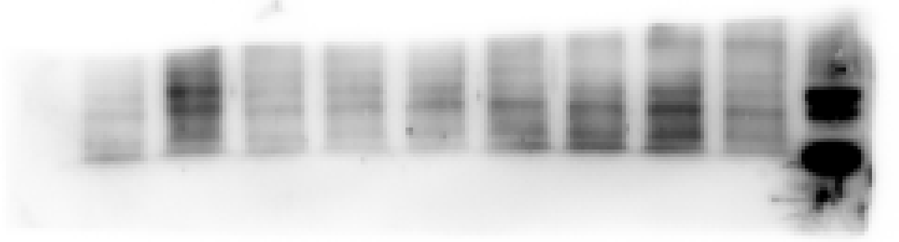

ERK- repeat 2

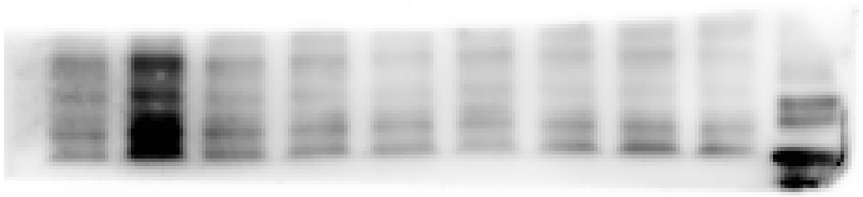

ERK- repeat 3

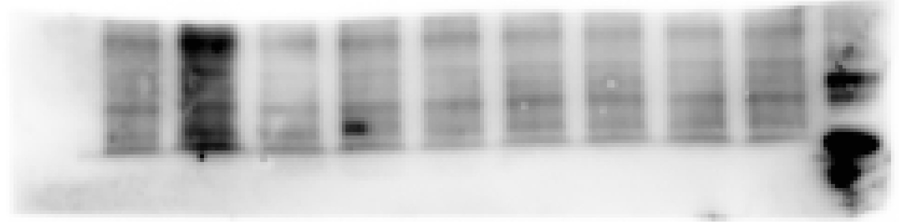

JNK- repeat 1

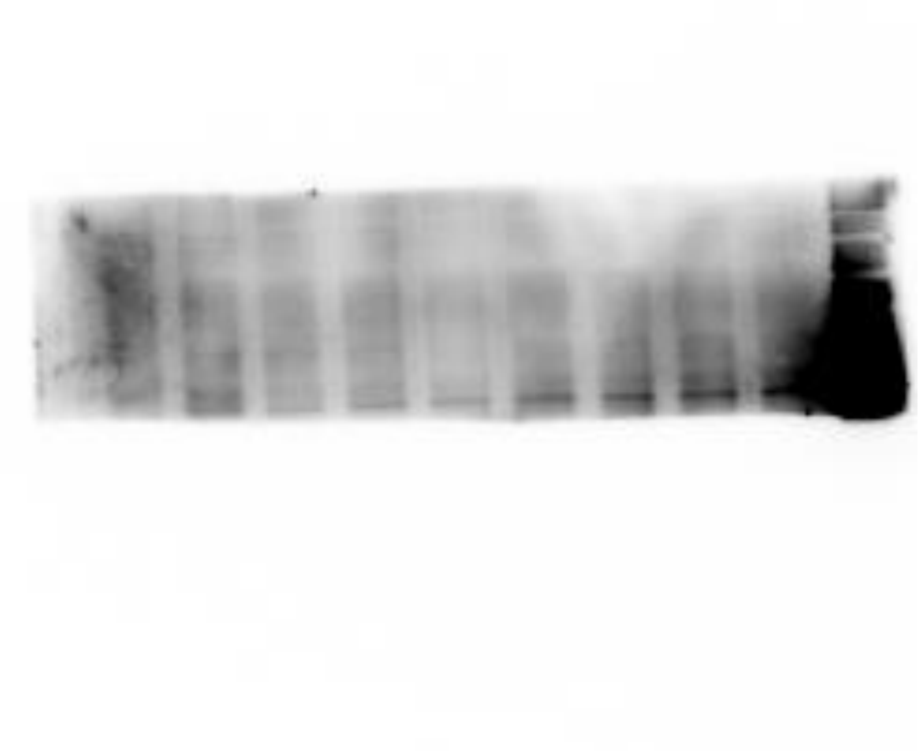

JNK- repeat 2

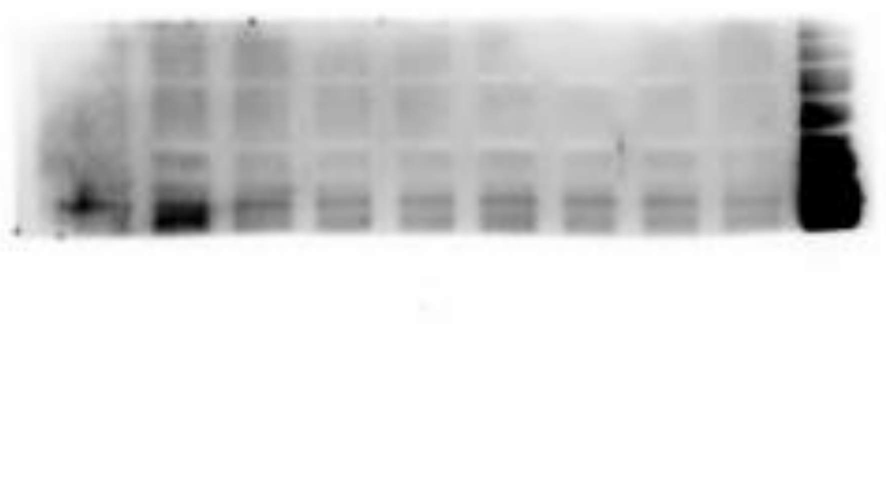

JNK- repeat 3

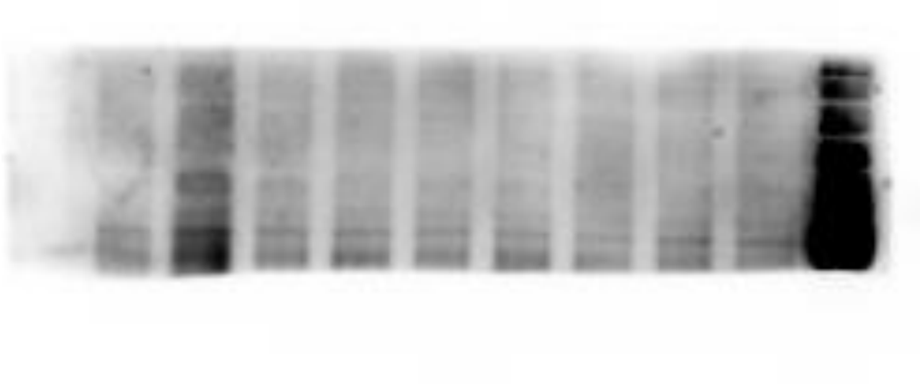

GAPDH- repeat 1

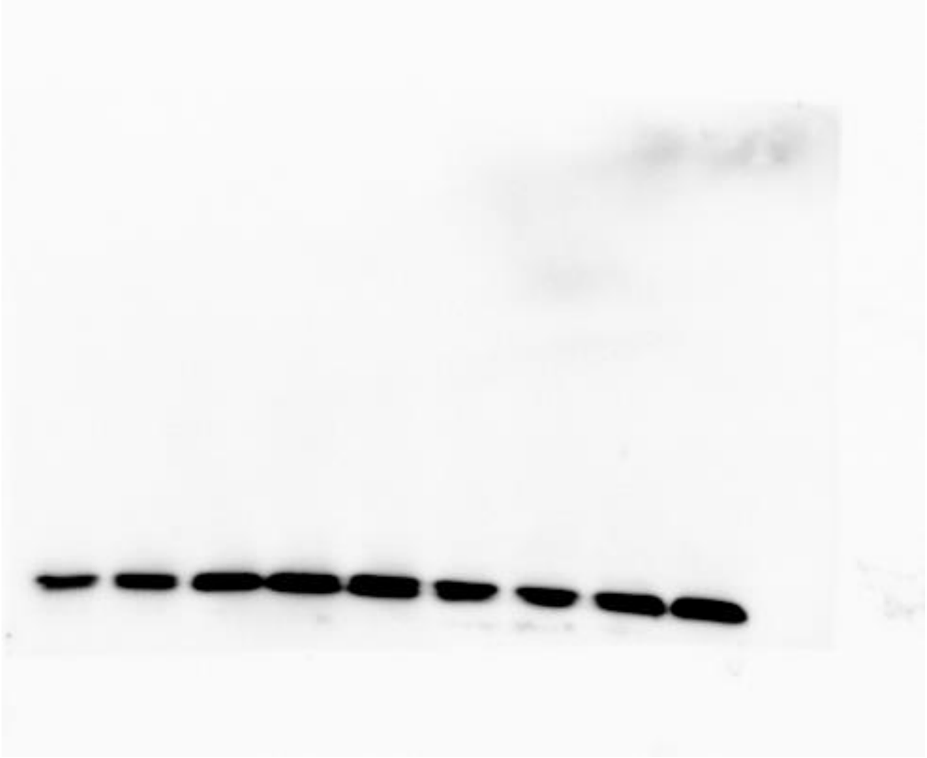

GAPDH- repeat 2

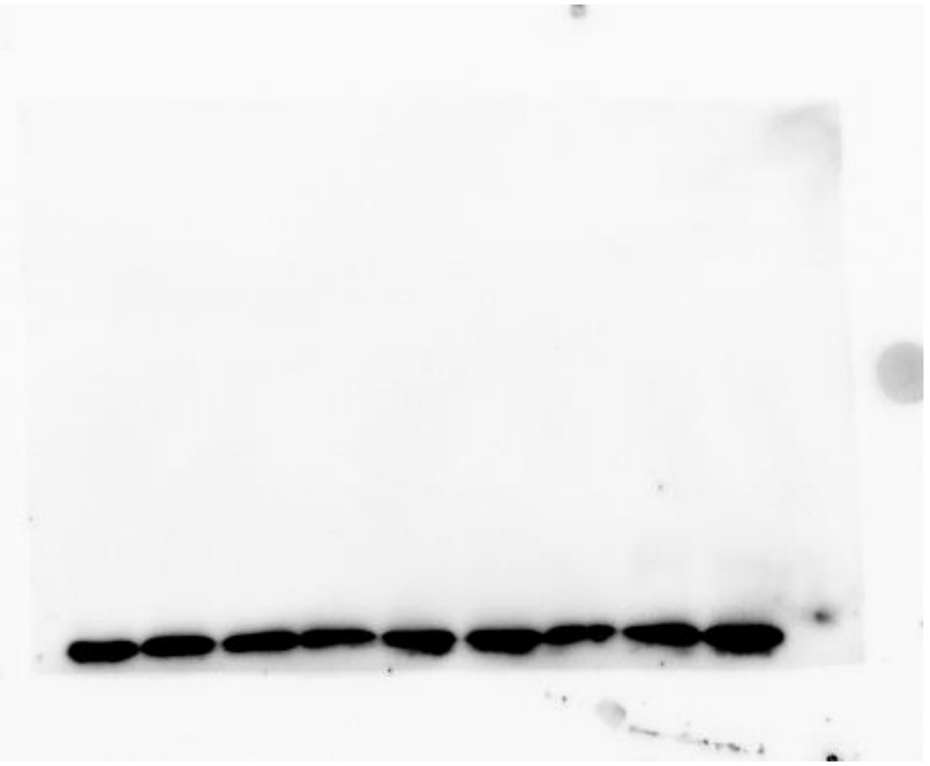

GAPDH- repeat 3

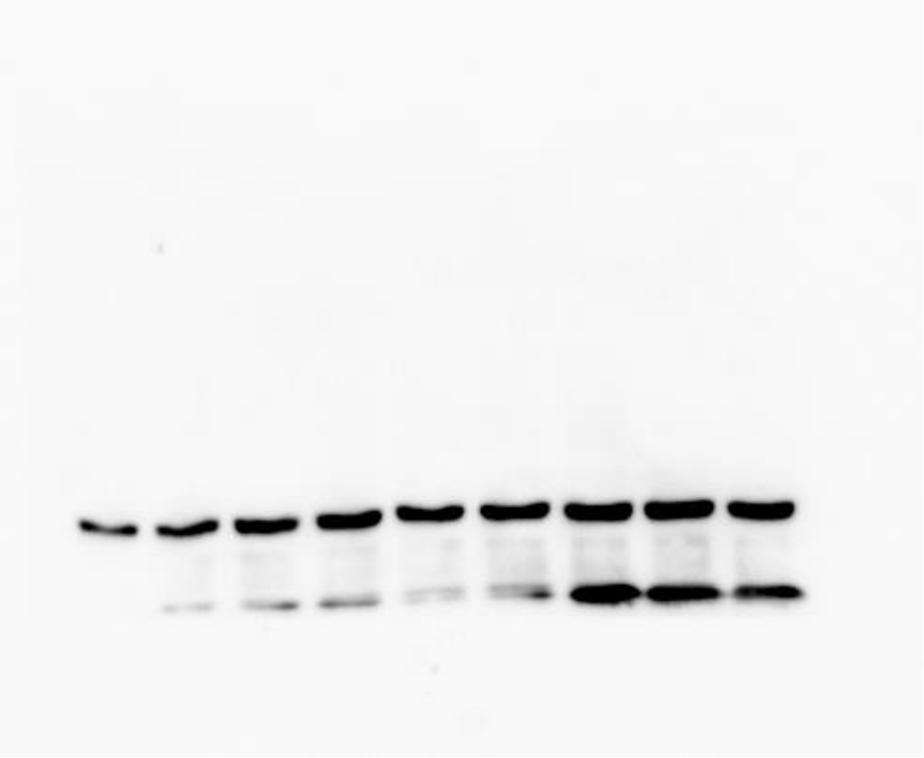

Supplement: Supplementary Materials — Table S1. Active ingredients of Aloe. Table S2. Potential targets related to active ingredients. Table S3. potential targets related to constipation. Table S4. Common targets related to active ingredients. Table S5. Table S5-1. Detailed information of BP enrichment of PPI network cluster 1 targets; Table S5-2. Detailed information of CC enrichment of PPI network cluster 1 targets; Table S5-3. Detailed information of MF enrichment of PPI network cluster 1 targets; Table S5-4. Detailed information of KEGG pathways enrichment of PPI network cluster 1 targets. Table S6. Table S6-1. Detailed information of BP enrichment of common targets; Table S6-2. Detailed information of CC enrichment of common targets; Table S6-3. Detailed information of MF enrichment of common targets; Table S6-4. Detailed information of KEGG pathways enrichment of common targets. Table S7. Original images of H&E staining in colon of three repeats in each group. Table S8. Raw data of 5-HT, SP, and VIP in serum and colon determined by ELISA kits. Table S9. Raw data of NF-κB p65, AKT, ERK, and JNK in colon determined by RT-PCR method. Table S10. Original images of ERK, JNK, AKT, and NF-κB p65 in colon of Western Blot, and its raw data quantification. [file 6225758.f1.zip › suppl table 1-10/Table S10 (1) (1).pdf]
