# Supplementary material for: Exploring Molecular Mechanisms of Aloe barbadmsis Miller on Diphenoxylate-Induced Constipation in Mice
Source: Evid Based Complement Alternat Med. 2022 May 6;2022:6225758. doi: 10.1155/2022/6225758 (PMC9106447; doi:10.1155/2022/6225758)
Supplement: Supplementary Materials — Table S1. Active ingredients of Aloe. Table S2. Potential targets related to active ingredients. Table S3. potential targets related to constipation. Table S4. Common targets related to active ingredients. Table S5. Table S5-1. Detailed information of BP enrichment of PPI network cluster 1 targets; Table S5-2. Detailed information of CC enrichment of PPI network cluster 1 targets; Table S5-3. Detailed information of MF enrichment of PPI network cluster 1 targets; Table S5-4. Detailed information of KEGG pathways enrichment of PPI network cluster 1 targets. Table S6. Table S6-1. Detailed information of BP enrichment of common targets; Table S6-2. Detailed information of CC enrichment of common targets; Table S6-3. Detailed information of MF enrichment of common targets; Table S6-4. Detailed information of KEGG pathways enrichment of common targets. Table S7. Original images of H&E staining in colon of three repeats in each group. Table S8. Raw data of 5-HT, SP, and VIP in serum and colon determined by ELISA kits. Table S9. Raw data of NF-κB p65, AKT, ERK, and JNK in colon determined by RT-PCR method. Table S10. Original images of ERK, JNK, AKT, and NF-κB p65 in colon of Western Blot, and its raw data quantification. [file 6225758.f1.zip › suppl table 1-10/Table S2 (1) (1).pdf]

Supplementory Table S2: potential targets related to active ingredients

Gene symbol	Uniprot ID	Description
ABCA1	O00767	Phospholipid-transporting ATPase ABCA1
ABCB1	P24310	ATP-dependent translocase ABCB1
ABCB11	A1L3X0	Bile salt export pump
ABCC1	P33527	Multidrug resistance-associated protein 1
ABCC2	P78411	Canalicular multispecific organic anion transporter 1
ABCC4	O15439	Multidrug resistance-associated protein 4
ABCC8	P08684	ATP-binding cassette sub-family C member 8
ABCC9	P11511	ATP-binding cassette sub-family C member 9
ABCF1	Q63ZY3	ATP-binding cassette sub-family F member 1
ABCG1	Q9NSA2	ATP-binding cassette sub-family G member 1
ABHD5	Q86SK9	1-acylglycerol-3-phosphate O-acyltransferase ABHD5
ABL1	Q9BTZ2	Tyrosine-protein kinase ABL1
ABL2	Q9BTZ2	Tyrosine-protein kinase ABL2
ACACA	Q13085	Acetyl-CoA carboxylase 1
ACHE	P48023	Acetylcholinesterase
ACP3	P15309	Prostatic acid phosphatase
ACSL1	Q9BPW9	Long-chain-fatty-acid--CoA ligase 1
ACSL3	Q8TC12	Long-chain-fatty-acid--CoA ligase 3
ACSL4	P15559	Long-chain-fatty-acid--CoA ligase 4
ACSS1	Q6VVX0	Acetyl-coenzyme A synthetase 2-like, mitochondrial
ACSS2	P05108	Acetyl-coenzyme A synthetase, cytoplasmic
ACVR1	O75911	Activin receptor type-1
ACVR1B	P08684	Activin receptor type-1B
ACVRL1	Q6VVX0	Serine/threonine-protein kinase receptor R3
ACY1	P00325	Aminoacylase-1
ACY3	Q08289	N-acyl-aromatic-L-amino acid amidohydrolase
ADCY1	O75911	Adenylate cyclase type 1
ADH1A	P08263	Alcohol dehydrogenase 1A
ADH1B	O75715	All-trans-retinol dehydrogenase [NAD
ADH1C	Q7RTV2	Alcohol dehydrogenase 1C
ADH4	P07327	All-trans-retinol dehydrogenase [NAD
ADH7	O60488	All-trans-retinol dehydrogenase [NAD
ADIPOQ	P10276	Adiponectin
ADORA1	P41145	Adenosine receptor A1
ADORA2A	Q92615	Adenosine receptor A2a
ADORA2B	O95237	Adenosine receptor A2b
ADORA3	P01584	Adenosine receptor A3
ADRA1A	Q96RI1	Alpha-1A adrenergic receptor
ADRA1B	P10153	Alpha-1B adrenergic receptor
ADRA1D	P08235	Alpha-1D adrenergic receptor
ADRA2A	P08195	Alpha-2A adrenergic receptor

ADRA2B	P10827	Alpha-2B adrenergic receptor
ADRA2C	P07204	Alpha-2C adrenergic receptor
ADRB1	Q6NUM9	Beta-1 adrenergic receptor
ADRB2	Q9NYR8	Beta-2 adrenergic receptor
ADRB3	Q15349	Beta-3 adrenergic receptor
AFG3L2	Q9GZR5	AFG3-like protein 2
AGTR2	Q15784	Type-2 angiotensin II receptor
AHRR	A9YTQ3	Aryl hydrocarbon receptor
AHSA1	O95433	Activator of 90 kDa heat shock protein ATPase homolog 1
AKR1C1	P09455	Aldo-keto reductase family 1 member C1
AKR1C2	P46531	Aldo-keto reductase family 1 member C2
AKR1C3	Q9ULI3	Aldo-keto reductase family 1 member C3
AKR1C4	Q6NVY1	Aldo-keto reductase family 1 member C4
AKR1D1	Q6NUM9	Aldo-keto reductase family 1 member D1
AKT1	P05181	RAC-alpha serine/threonine-protein kinase
ALDH1A1	Q96AD5	Retinal dehydrogenase 1
ALDH1A2	Q07869	Retinal dehydrogenase 2
ALDH1A3	P23415	Aldehyde dehydrogenase family 1 member A3
ALDH1B1	Q13698	Aldehyde dehydrogenase X, mitochondrial
ALDH2	P45844	Aldehyde dehydrogenase, mitochondrial
ALDH3A1	P28222	Aldehyde dehydrogenase, dimeric NADP-preferring
ALDH3B1	Q04828	Aldehyde dehydrogenase family 3 member B1
ALDH3B2	Q04828	Aldehyde dehydrogenase family 3 member B2
ALDH8A1	P10916	2-aminomuconic semialdehyde dehydrogenase
ALK	P78352	ALK tyrosine kinase receptor
ALOX12B	O75342	Arachidonate 12-lipoxygenase
ALOX15	P10276	Polyunsaturated fatty acid lipoxygenase ALOX15
ALOX15B	P22460	Polyunsaturated fatty acid lipoxygenase ALOX15B
ALOX5	P45984	Polyunsaturated fatty acid 5-lipoxygenase
ALOX5AP	P35408	Arachidonate 5-lipoxygenase-activating protein
AMHR2	P10606	Anti-Muellerian hormone type-2 receptor
ANAPC2	O00712	Anaphase-promoting complex subunit 2
ANK3	P05937	Ankyrin-3
ANPEP	P02774	Aminopeptidase N
ANXA1	P54619	Annexin A1
ANXA13	Q99735	Annexin A13
APAF1	Q07973	Apoptotic protease-activating factor 1
APOA2	Q8NBN7	Apolipoprotein A-II
APOE	Q9NR19	Apolipoprotein E
AQP8	O60488	Aquaporin-8
AR	P43116	Androgen receptor
ARAF	P08684	Serine/threonine-protein kinase A-Raf
ARX	P18825	Homeobox protein ARX
ASNS	Q6VVX0	Asparagine synthetase [glutamine-hydrolyzing]

ASPA	Q9NUB1	Aspartoacylase
ASS1	P04798	Argininosuccinate synthase
AVP	P16389	Vasopressin-neurophysin 2-copeptin
AVPR1A	P19793	Vasopressin V1a receptor
AVPR2	P30518	Vasopressin V2 receptor
AXL	P30530	Tyrosine-protein kinase receptor UFO
B4GALT1	P23771	Beta-1,4-galactosyltransferase 1
BACE1	P56817	Beta-secretase 1
BAX	Q8TDU6	Apoptosis regulator BAX
BCHE	P19087	Cholinesterase
BCL2	P29590	Apoptosis regulator Bcl-2
BCL2L1	Q07817	Bcl-2-like protein 1
BDKRB2	P22001	B2 bradykinin receptor
BGLAP	P56705	Osteocalcin
BIRC5	O15392	Baculoviral IAP repeat-containing protein 5
C1R	P00736	Complement C1r subcomponent
CACNA1A	P19793	Voltage-dependent P/Q-type calcium channel subunit alpha-1A
CACNA1B	P33121	Voltage-dependent N-type calcium channel subunit alpha-1B
CACNA1C	O96014	Voltage-dependent L-type calcium channel subunit alpha-1C
CACNA1D	O95863	Voltage-dependent L-type calcium channel subunit alpha-1D
CACNA1F	O94788	Voltage-dependent L-type calcium channel subunit alpha-1F
CACNA1G	P18089	Voltage-dependent T-type calcium channel subunit alpha-1G
CACNA1I	Q9P0X4	Voltage-dependent T-type calcium channel subunit alpha-1I
CACNA1S	Q6J4K2	Voltage-dependent L-type calcium channel subunit alpha-1S
CACNB1	P11473	Voltage-dependent L-type calcium channel subunit beta-1
CACNB2	P30837	Voltage-dependent L-type calcium channel subunit beta-2
CACNB3	Q13698	Voltage-dependent L-type calcium channel subunit beta-3
CACNB4	Q96QS3	Voltage-dependent L-type calcium channel subunit beta-4
CACNG1	B1AH88	Voltage-dependent calcium channel gamma-1 subunit
CACNG2	P11473	Voltage-dependent calcium channel gamma-2 subunit
CALB1	Q14353	Calbindin
CALY	P11473	Neuron-specific vesicular protein calcyon
CAPN3	Q63ZY3	Calpain-3
CASP3	P42574	Caspase-3
CASP7	P55210	Caspase-7
CASP8	Q14790	Caspase-8
CASP9	P55211	Caspase-9
CAT	Q00975	Catalase
CAV1	Q03135	Caveolin-1
CCM2L	P13631	Cerebral cavernous malformations 2 protein-like
CCNB1	P14635	G2/mitotic-specific cyclin-B1
CCND1	P24385	G1/S-specific cyclin-D1
CD40LG	P29965	CD40 ligand
CDC20	P19838	Cell division cycle protein 20 homolog

CDK1	P06493	Cyclin-dependent kinase 1
CDK15	P14854	Cyclin-dependent kinase 15
CDKN1A	P38936	Cyclin-dependent kinase inhibitor 1
CES1	P18283	Liver carboxylesterase 1
CETP	P11597	Cholesteryl ester transfer protein
CFTR	P11021	Cystic fibrosis transmembrane conductance regulator
CHEK2	O96017	Serine/threonine-protein kinase Chk2
CHRFAM7A	Q14542	CHRNA7-FAM7A fusion protein
CHRM1	Q9UDX4	Muscarinic acetylcholine receptor M1
CHRM2	Q9UDX3	Muscarinic acetylcholine receptor M2
CHRM3	Q9UDX4	Muscarinic acetylcholine receptor M3
CHRM4	Q9UDX3	Muscarinic acetylcholine receptor M4
CHRNA10	P19634	Neuronal acetylcholine receptor subunit alpha-10
CHRNA2	Q05940	Neuronal acetylcholine receptor subunit alpha-2
CHRNA3	Q02447	Neuronal acetylcholine receptor subunit alpha-3
CHRNA4	P32418	Neuronal acetylcholine receptor subunit alpha-4
CHRNA5	O43623	Neuronal acetylcholine receptor subunit alpha-5
CHRNA6	O95863	Neuronal acetylcholine receptor subunit alpha-6
CHRNA7	Q9UHW9	Neuronal acetylcholine receptor subunit alpha-7
CHRNA9	O15090	Neuronal acetylcholine receptor subunit alpha-9
CHRNB2	P54219	Neuronal acetylcholine receptor subunit beta-2
CHRNB3	Q9UPU3	Neuronal acetylcholine receptor subunit beta-3
CHRNB4	Q96NH3	Neuronal acetylcholine receptor subunit beta-4
CHUK	O15111	Inhibitor of nuclear factor kappa-B kinase subunit alpha
CKB	P08908	Creatine kinase B-type
CKM	P51843	Creatine kinase M-type
CKMT1A	P20393	Creatine kinase U-type, mitochondrial
CKMT2	P51787	Creatine kinase S-type, mitochondrial
CLDN4	O14493	Claudin-4
CNST	Q9Y5Y4	Consortin
CNTNAP4	P48448	Contactin-associated protein-like 4
COL1A1	P17658	Collagen alpha-1
COL27A1	Q9HBH5	Collagen alpha-1
COX4I1	O15217	Cytochrome c oxidase subunit 4 isoform 1, mitochondrial
COX5A	Q16772	Cytochrome c oxidase subunit 5A, mitochondrial
COX5B	P09210	Cytochrome c oxidase subunit 5B, mitochondrial
COX6A2	Q99684	Cytochrome c oxidase subunit 6A2, mitochondrial
COX6B1	P05413	Cytochrome c oxidase subunit 6B1
COX6C	P36969	Cytochrome c oxidase subunit 6C
COX7A1	Q99684	Cytochrome c oxidase subunit 7A1, mitochondrial
COX7B	P38435	Cytochrome c oxidase subunit 7B, mitochondrial
COX7B2	P30711	Cytochrome c oxidase subunit 7B2, mitochondrial
COX7C	P08709	Cytochrome c oxidase subunit 7C, mitochondrial
COX8A	Q9Y2Q3	Cytochrome c oxidase subunit 8A, mitochondrial

CPLX2	P25963	Complexin-2
CRLF1	P37023	Cytokine receptor-like factor 1
CRP	P02741	C-reactive protein
CTNNB1	P35222	Catenin beta-1
CTSD	P07339	Cathepsin D
CXCL10	P02778	C-X-C motif chemokine 10
CXCL11	O14625	C-X-C motif chemokine 11
CXCL2	P19875	C-X-C motif chemokine 2
CXCL8	P10145	Interleukin-8
CYGB	P05091	Cytoglobin
CYP11A1	Q04760	Cholesterol side-chain cleavage enzyme, mitochondrial
CYP17A1	P62508	Steroid 17-alpha-hydroxylase/17,20 lyase
CYP19A1	O00591	Aromatase
CYP1A1	P10826	Cytochrome P450 1A1
CYP1B1	Q16678	Cytochrome P450 1B1
CYP24A1	P09038	1,25-dihydroxyvitamin D
CYP27A1	Q8TCU5	Sterol 26-hydroxylase, mitochondrial
CYP27B1	P19440	25-hydroxyvitamin D-1 alpha hydroxylase, mitochondrial
CYP2E1	P04040	Cytochrome P450 2E1
CYP2R1	P08631	Vitamin D 25-hydroxylase
CYP3A4	Q9GZV9	Cytochrome P450 3A4
CYP4F2	P23759	Cytochrome P450 4F2
DAB2IP	Q07812	Disabled homolog 2-interacting protein
DAPK1	P53355	Death-associated protein kinase 1
DCAF5	Q96JK2	DDB1- and CUL4-associated factor 5
DGKA	P21941	Diacylglycerol kinase alpha
DGKI	Q02641	Diacylglycerol kinase iota
DHRS3	P21266	Short-chain dehydrogenase/reductase 3
DHRS4	O14764	Dehydrogenase/reductase SDR family member 4
DHRS9	Q14524	Dehydrogenase/reductase SDR family member 9
DIO1	P49895	Type I iodothyronine deiodinase
DLG4	P31749	Disks large homolog 4
DNAJA3	P06401	DnaJ homolog subfamily A member 3, mitochondrial
DPP4	P27487	Dipeptidyl peptidase 4
DRD1	P35610	D
DRD2	P04278	D
DRD3	Q13621	D
DRD4	P31645	D
DRD5	P31645	D
DUOX2	Q9NRD8	Dual oxidase 2
E2F1	Q01094	Transcription factor E2F1
E2F2	Q14209	Transcription factor E2F2
EDN1	P43353	Endothelin-1
EDNRA	P04035	Endothelin-1 receptor

EGFR	P00533	Epidermal growth factor receptor
EIF2AK1	P30301	Eukaryotic translation initiation factor 2-alpha kinase 1
EIF6	P56537	Eukaryotic translation initiation factor 6
ELK1	P19419	ETS domain-containing protein Elk-1
ELOVL1	P04070	Elongation of very long chain fatty acids protein 1
ELOVL3	P00439	Elongation of very long chain fatty acids protein 3
ELOVL4	O14939	Elongation of very long chain fatty acids protein 4
ELOVL6	Q16322	Elongation of very long chain fatty acids protein 6
ELOVL7	P19838	Elongation of very long chain fatty acids protein 7
ERBB2	P04626	Receptor tyrosine-protein kinase erbB-2
ERBB3	P21860	Receptor tyrosine-protein kinase erbB-3
ESD	Q9UK32	S-formylglutathione hydrolase
ESR1	P07203	Estrogen receptor
ESR2	Q99684	Estrogen receptor beta
ESRRG	P17252	Estrogen-related receptor gamma
F10	Q9BQA1	Coagulation factor X
F12	Q9NS86	Coagulation factor XII
F2	P15923	Prothrombin
F3	P13726	Tissue factor
F7	Q9BQB6	Coagulation factor VII
F8	P00451	Coagulation factor VII
F9	P07202	Coagulation factor IX
FABP2	Q8TC12	Fatty acid-binding protein, intestinal
FABP3	P04054	Fatty acid-binding protein, heart
FABP6	P48169	Gastrotropin
FADS1	P34931	Acyl-CoA
FADS2	P10745	Acyl-CoA 6-desaturase
FAS	Q16671	Tumor necrosis factor receptor superfamily member 6
FASLG	P05771	Tumor necrosis factor ligand superfamily member 6
FASN	P49327	Fatty acid synthase
FBP1	P08243	Fructose-1,6-bisphosphatase 1
FECH	P59796	Ferrochelatase, mitochondrial
FFAR1	Q13393	Free fatty acid receptor 1
FGF10	P48443	Fibroblast growth factor 10
FGF2	O76054	Fibroblast growth factor 2
FGF23	Q8TDU6	Fibroblast growth factor 23
FGF4	Q16647	Fibroblast growth factor 4
FMR1	Q5U5Q3	Synaptic functional regulator FMR1
FNDC5	P19793	Fibronectin type III domain-containing protein 5
FOS	P01100	Proto-oncogene c-Fos
FOXA1	Q02962	Hepatocyte nuclear factor 3-alpha
G6PD	P11413	Glucose-6-phosphate 1-dehydrogenase
GABRA1	Q13277	Gamma-aminobutyric acid receptor subunit alpha-1
GABRA2	P48029	Gamma-aminobutyric acid receptor subunit alpha-2

GABRA3	P04637	Gamma-aminobutyric acid receptor subunit alpha-3
GABRA4	O15164	Gamma-aminobutyric acid receptor subunit alpha-4
GABRA5	Q16623	Gamma-aminobutyric acid receptor subunit alpha-5
GABRA6	Q99808	Gamma-aminobutyric acid receptor subunit alpha-6
GABRB1	P01375	Gamma-aminobutyric acid receptor subunit beta-1
GABRB2	O15164	Gamma-aminobutyric acid receptor subunit beta-2
GABRB3	O94768	Gamma-aminobutyric acid receptor subunit beta-3
GABRD	Q9UP95	Gamma-aminobutyric acid receptor subunit delta
GABRE	O60248	Gamma-aminobutyric acid receptor subunit epsilon
GABRG1	P48436	Gamma-aminobutyric acid receptor subunit gamma-1
GABRG3	Q07912	Gamma-aminobutyric acid receptor subunit gamma-3
GABRP	Q6ZSM3	Gamma-aminobutyric acid receptor subunit pi
GABRQ	O15164	Gamma-aminobutyric acid receptor subunit theta
GAMT	P51843	Guanidinoacetate N-methyltransferase
GATA3	P29377	Trans-acting T-cell-specific transcription factor GATA-3
GATM	P28221	Glycine amidinotransferase, mitochondrial
GC	Q8TED1	Vitamin D-binding protein
GDF5	P28702	Growth/differentiation factor 5
GFI1	O15399	Zinc finger protein Gfi-1
GGCX	Q8N0U8	Vitamin K-dependent gamma-carboxylase
GGT1	O75469	Glutathione hydrolase 1 proenzyme
GJA1	P17302	Gap junction alpha-1 protein
GJA5	P52895	Gap junction alpha-5 protein
GJD4	Q00653	Gap junction delta-4 protein
GLB1	P16278	Beta-galactosidase
GLO1	Q8WXA8	Lactoylglutathione lyase
GLRA1	P11168	Glycine receptor subunit alpha-1
GLRA2	Q9UPR5	Glycine receptor subunit alpha-2
GLRX	P58400	Glutaredoxin-1
GLRX2	Q9H1Y3	Glutaredoxin-2, mitochondrial
GNAT2	P49585	Guanine nucleotide-binding protein G
GPBAR1	Q9UN88	G-protein coupled bile acid receptor 1
GPR35	Q9HC97	G-protein coupled receptor 35
GPRC5A	Q9UEF7	Retinoic acid-induced protein 3
GPX1	P01584	Glutathione peroxidase 1
GPX2	Q9BZF1	Glutathione peroxidase 2
GPX3	Q70Z44	Glutathione peroxidase 3
GPX4	Q9NS86	Phospholipid hydroperoxide glutathione peroxidase
GPX5	O95819	Epididymal secretory glutathione peroxidase
GPX6	Q14623	Glutathione peroxidase 6
GPX7	P62714	Glutathione peroxidase 7
GPX8	Q16873	Probable glutathione peroxidase 8
GRIA1	P11473	Glutamate receptor 1
GRIA2	P08047	Glutamate receptor 2

GRIA3	O43623	Glutamate receptor 3
GRIA4	P55011	Glutamate receptor 4
GRIK2	P17516	Glutamate receptor ionotropic, kainate 2
GRIN1	P27216	Glutamate receptor ionotropic, NMDA 1
GRIN2A	P30411	Glutamate receptor ionotropic, NMDA 2A
GRIN2B	P42330	Glutamate receptor ionotropic, NMDA 2B
GRIN2C	P40394	Glutamate receptor ionotropic, NMDA 2C
GRIN2D	Q92887	Glutamate receptor ionotropic, NMDA 2D
GRIN3A	P56705	Glutamate receptor ionotropic, NMDA 3A
GRIN3B	P42330	Glutamate receptor ionotropic, NMDA 3B
GSK3B	P49841	Glycogen synthase kinase-3 beta
GSR	Q92781	Glutathione reductase, mitochondrial
GSS	P04150	Glutathione synthetase
GSTA1	Q9UGI9	Glutathione S-transferase A1
GSTA2	A5X5Y0	Glutathione S-transferase A2
GSTA3	P32245	Glutathione S-transferase A3
GSTA4	P05019	Glutathione S-transferase A4
GSTA5	P67775	Glutathione S-transferase A5
GSTK1	Q9Y478	Glutathione S-transferase kappa 1
GSTM1	P54646	Glutathione S-transferase Mu 1
GSTM2	P37231	Glutathione S-transferase Mu 2
GSTM3	Q9Y478	Glutathione S-transferase Mu 3
GSTM4	P12271	Glutathione S-transferase Mu 4
GSTM5	P07949	Glutathione S-transferase Mu 5
GSTO1	P28482	Glutathione S-transferase omega-1
GSTO2	P04150	Glutathione S-transferase omega-2
GSTP1	O43741	Glutathione S-transferase P
GSTT1	P49902	Glutathione S-transferase theta-1
GSTZ1	Q8NF37	Maleylacetoacetate isomerase
HAGH	Q15648	Hydroxyacylglutathione hydrolase, mitochondrial
HAS2	Q92819	Hyaluronan synthase 2
HCK	P15923	Tyrosine-protein kinase HCK
HDAC1	P43026	Histone deacetylase 1
HDAC2	P02774	Histone deacetylase 2
HEG1	P48443	Protein HEG homolog 1
HIBCH	P15923	3-hydroxyisobutyryl-CoA hydrolase, mitochondrial
HIF1A	Q16665	Hypoxia-inducible factor 1-alpha
HK2	P52789	Hexokinase-2
HMGCR	Q8NAU1	3-hydroxy-3-methylglutaryl-coenzyme A reductase
HMOX1	P09601	Heme oxygenase 1
HOPX	Q15648	Homeodomain-only protein
HPGDS	Q9UHM6	Hematopoietic prostaglandin D synthase
HSF1	Q00613	Heat shock factor protein 1
HSP90AA1	P07900	Heat shock protein HSP 90-alpha

HSPA1A	Q86UL8	Heat shock 70 kDa protein 1A
HSPA1B	O95264	Heat shock 70 kDa protein 1B
HSPA1L	Q9HAZ2	Heat shock 70 kDa protein 1-like
HSPA2	Q9NYR8	Heat shock-related 70 kDa protein 2
HSPA5	Q9BPY8	Endoplasmic reticulum chaperone BiP
HSPA6	P04150	Heat shock 70 kDa protein 6
HSPA8	P78411	Heat shock cognate 71 kDa protein
HSPB1	P04792	Heat shock protein beta-1
HTR1A	Q13573	5-hydroxytryptamine receptor 1A
HTR1B	Q13573	5-hydroxytryptamine receptor 1B
HTR1D	P19320	5-hydroxytryptamine receptor 1D
HTR2A	Q6NUS8	5-hydroxytryptamine receptor 2A
HTR2B	Q9BYB0	5-hydroxytryptamine receptor 2B
HTR2C	P15923	5-hydroxytryptamine receptor 2C
HTR3A	O43623	5-hydroxytryptamine receptor 3A
HTR3B	P12235	5-hydroxytryptamine receptor 3B
HTR3C	O75312	5-hydroxytryptamine receptor 3C
HTR3D	P09486	5-hydroxytryptamine receptor 3D
HTR3E	P56705	5-hydroxytryptamine receptor 3E
ICAM1	P05362	Intercellular adhesion molecule 1
IFNG	P01579	Interferon gamma
IGF1	P05771	Insulin-like growth factor I
IGF2	P01344	Insulin-like growth factor II
IGFBP3	P17936	Insulin-like growth factor-binding protein 3
IHH	P19793	Indian hedgehog protein
IKBKB	P01160	Inhibitor of nuclear factor kappa-B kinase subunit beta
IL10	P22301	Interleukin-10
IL13	Q9HBH5	Interleukin-13
IL1A	P01583	Interleukin-1 alpha
IL1B	Q9UEF7	Interleukin-1 beta
IL2	P60568	Interleukin-2
IL6	P05231	Interleukin-6
INS	P22459	Insulin [Cleaved into: Insulin B chain; Insulin A chain]
INSR	P06213	Insulin receptor
IRF1	P10914	Interferon regulatory factor 1
IRX5	O60391	Iroquois-class homeodomain protein IRX-5
ITGAL	P55317	Integrin alpha-L
ITGAV	Q13564	Integrin alpha-V
ITGB3	P10620	Integrin beta-3
IYD	P10275	Iodotyrosine deiodinase 1
JAK3	P06401	Tyrosine-protein kinase JAK3
JUN	P05412	Transcription factor AP-1
JUNB	P29377	Transcription factor jun-B
KANK2	Q13547	KN motif and ankyrin repeat domain-containing protein 2

KCNA1	P30542	Potassium voltage-gated channel subfamily A member 1
KCNA10	O14727	Potassium voltage-gated channel subfamily A member 10
KCNA2	P51857	Potassium voltage-gated channel subfamily A member 2
KCNA3	Q92887	Potassium voltage-gated channel subfamily A member 3
KCNA4	P0DMS8	Potassium voltage-gated channel subfamily A member 4
KCNA5	P29377	Potassium voltage-gated channel subfamily A member 5
KCNA6	P10275	Potassium voltage-gated channel subfamily A member 6
KCNA7	P00352	Potassium voltage-gated channel subfamily A member 7
KCNB1	O60706	Potassium voltage-gated channel subfamily B member 1
KCNB2	P29275	Potassium voltage-gated channel subfamily B member 2
KCNC1	Q13936	Potassium voltage-gated channel subfamily C member 1
KCNC2	P02649	Potassium voltage-gated channel subfamily C member 2
KCNC3	Q09428	Potassium voltage-gated channel subfamily C member 3
KCND1	O00555	Potassium voltage-gated channel subfamily D member 1
KCND2	Q13936	Potassium voltage-gated channel subfamily D member 2
KCND3	Q01668	Potassium voltage-gated channel subfamily D member 3
KCNE5	Q96HD9	Potassium voltage-gated channel subfamily E regulatory beta subunit 5
KCNH2	O95573	Potassium voltage-gated channel subfamily H member 2
KCNIP2	P01185	Kv channel-interacting protein 2
KCNJ11	P08913	ATP-sensitive inward rectifier potassium channel 11
KCNJ3	O60248	G protein-activated inward rectifier potassium channel 1
KCNJ5	O15164	G protein-activated inward rectifier potassium channel 4
KCNJ6	P57103	G protein-activated inward rectifier potassium channel 2
KCNJ9	Q8NER1	G protein-activated inward rectifier potassium channel 3
KCNK10	P57789	Potassium channel subfamily K member 10
KCNK2	O95069	Potassium channel subfamily K member 2
KCNK4	P00352	Potassium channel subfamily K member 4
KCNQ1	Q01668	Potassium voltage-gated channel subfamily KQT member 1
KDR	P35968	Vascular endothelial growth factor receptor 2
KL	Q8TCU5	Klotho
LANCL2	Q16775	LanC-like protein 2
LARP4B	P30559	La-related protein 4B
LEP	Q03154	Leptin
LPCAT1	Q03181	Lysophosphatidylcholine acyltransferase 1
LRAT	O15520	Lecithin retinol acyltransferase
LRRC4B	Q02641	Leucine-rich repeat-containing protein 4B
LTC4S	Q9Y478	Leukotriene C4 synthase
MAGI2	P54284	Membrane-associated guanylate kinase, WW and PDZ domain-containing protein 2
MAOB	P27338	Amine oxidase [flavin-containing] B
MAP4K4	P52333	Mitogen-activated protein kinase kinase kinase kinase 4
MAPK1	P28335	Mitogen-activated protein kinase 1
MAPK12	P53778	Mitogen-activated protein kinase 1
MAPK9	P19793	Mitogen-activated protein kinase 9

MAPT	P10636	Microtubule-associated protein tau
MATN1	P43115	Cartilage matrix protein
MC4R	Q04771	Melanocortin receptor 4
MECOM	P27986	Histone-lysine N-methyltransferase MECOM
MED1	P05106	Mediator of RNA polymerase II transcription subunit 1
MET	P08581	Hepatocyte growth factor receptor
METRNL	Q96IZ0	Meteorin-like protein
MEX3C	O75845	RNA-binding E3 ubiquitin-protein ligase MEX3C
MGAM	O43451	Maltase-glucoamylase, intestinal
MGST1	O14920	Microsomal glutathione S-transferase 1
MGST2	P41159	Microsomal glutathione S-transferase 2
MGST3	P41595	Microsomal glutathione S-transferase 3
MIP	P15291	Lens fiber major intrinsic protein
MMP1	P03956	Interstitial collagenase
MMP2	P08253	72 kDa type IV collagenase
MMP3	P08254	Stromelysin-1
MMP9	P14780	Matrix metalloproteinase-9
MPO	P05164	Myeloperoxidase
MYC	P01106	Myc proto-oncogene protein
MYCN	P04198	Myc proto-oncogene protein
MYL2	Q92569	Myosin regulatory light chain 2, ventricular/cardiac muscle isoform
MYLK	Q15746	Myosin light chain kinase, smooth muscle
MYOD1	Q92736	Myoblast determination protein 1
NAE1	P09669	NEDD8-activating enzyme E1 regulatory subunit
NAMPT	P01178	Nicotinamide phosphoribosyltransferase
NAPRT	Q14721	Nicotinate phosphoribosyltransferase
NCF1	P14598	Neutrophil cytosol factor 1
NCOA2	Q15596	Nuclear receptor coactivator 2
NDRG2	Q15648	Protein NDRG2
NEK2	P51955	Serine/threonine-protein kinase NEK2
NEK6	Q9HC98	Serine/threonine-protein kinase NEK6
NEUROD2	P06401	Neurogenic differentiation factor 2
NFE2L2	Q16236	Nuclear factor erythroid 2-related factor 2
NFIB	P18825	Nuclear factor 1 B-type
NFKB1	P49788	Nuclear factor NF-kappa-B p105 subunit
NFKB2	P49788	Nuclear factor NF-kappa-B p100 subunit
NFKBIA	Q9UJ90	NF-kappa-B inhibitor alpha
NKX2-1	P09917	Homeobox protein Nkx-2.1
NKX3-1	Q99801	Homeobox protein Nkx-3.1
NLGN1	Q9Y4W6	Neuroligin-1
NODAL	P48547	Nodal homolog
NOS1	Q96PR1	Nitric oxide synthase, brain
NOS2	P62714	Nitric oxide synthase, inducible
NOS3	P29474	Nitric oxide synthase, endothelial

NOTCH1	Q96RP8	Neurogenic locus notch homolog protein 1
NOX4	Q9NPH5	NADPH oxidase 4
NPC1L1	P00748	NPC1-like intracellular cholesterol transporter 1
NPEPPS	P55786	Puromycin-sensitive aminopeptidase
NPPA	P09917	Natriuretic peptides A
NPPC	Q9Y5B6	C-type natriuretic peptide [Cleaved into: CNP-22; CNP-29; CNP-53]
NQO1	Q7Z2W7	NAD
NQO2	Q9H2X9	Ribosyldihydronicotinamide dehydrogenase [quinone]
NR0B1	P17066	Nuclear receptor subfamily 0 group B member 1
NR1D1	P19838	Nuclear receptor subfamily 1 group D member 1
NR1H2	Q9UN36	Oxysterols receptor LXR-beta
NR1H3	Q641Q3	Oxysterols receptor LXR-alpha
NR1H4	P51161	Bile acid receptor
NR1I2	Q13131	Nuclear receptor subfamily 1 group I member 2
NR1I3	Q14994	Nuclear receptor subfamily 1 group I member 3
NR3C1	P05019	Glucocorticoid receptor
NR3C2	P22352	Mineralocorticoid receptor
NRXN1	P08588	Neurexin-1-beta
NRXN2	Q07812	Neurexin-2
NRXN3	P45381	Neurexin-3-beta
NT5C2	Q9BW60	Cytosolic purine 5'-nucleotidase
NUAK1	O60285	NUAK family SNF1-like kinase 1
NUFIP2	P07225	Nuclear fragile X mental retardation-interacting protein 2
OAZ2	O95190	Ornithine decarboxylase
OPN3	P20701	Opsin-3
OPN4	P19793	Melanopsin
OPRK1	P00742	Kappa-type opioid receptor
OSBPL8	Q9H244	Oxysterol-binding protein-related protein 8
OXER1	P23759	Oxoeicosanoid receptor 1
OXTR	P05937	Oxytocin receptor
P2RY12	P47712	P2Y purinoceptor 12
PAH	P12271	Phenylalanine-4-hydroxylase
PARP1	P09874	Poly [ADP-ribose] polymerase 1
PAWR	P11309	PRKC apoptosis WT1 regulator protein
PAX2	Q6XQN6	Paired box protein Pax-2
PAX7	P48736	Paired box protein Pax-7
PAXBP1	P17706	PAX3- and PAX7-binding protein 1
PCNA	P12004	Proliferating cell nuclear antigen
PCOLCE	Q15113	Procollagen C-endopeptidase enhancer 1
PCYT1A	P18505	Choline-phosphate cytidylyltransferase A
PCYT1B	O95864	Choline-phosphate cytidylyltransferase B
PDPK1	Q9NS86	3-phosphoinositide-dependent protein kinase 1
PECAM1	P16284	Platelet endothelial cell adhesion molecule
PGR	P00390	Progesterone receptor

PHOSPHO1	P25445	Phosphoethanolamine/phosphocholine phosphatase
PIK3CG	O95881	Phosphatidylinositol 4,5-bisphosphate 3-kinase catalytic subunit gamma isoform
PIK3R1	P04150	Phosphatidylinositol 3-kinase regulatory subunit alpha
PIK3R2	Q15418	Phosphatidylinositol 3-kinase regulatory subunit beta
PIK3R3	O95237	Phosphatidylinositol 3-kinase regulatory subunit gamma
PIM1	Q2M3R5	Serine/threonine-protein kinase pim-1
PKIA	P61925	cAMP-dependent protein kinase inhibitor alpha
PKN1	Q16512	Protein kinase N1
PLA2G1B	P12104	Phospholipase A2
PLA2G2A	P46098	Phospholipase A2, membrane associated
PLA2G4A	Q9NT99	Cytosolic phospholipase A2
PLA2G6	P51812	85/88 kDa calcium-independent phospholipase A2
PLA2R1	Q6PHW0	Secretory phospholipase A2 receptor
PLAT	P54652	Tissue-type plasminogen activator
PLAU	P00749	Urokinase-type plasminogen activator
PLCB1	Q9Y4G8	1-phosphatidylinositol 4,5-bisphosphate phosphodiesterase beta-1
PLCL1	Q15648	Inactive phospholipase C-like protein 1
PLD1	P42261	Phospholipase D1
PLD2	Q9NS18	Phospholipase D2
PLK1	P53350	Serine/threonine-protein kinase PLK1
PML	Q9GZV9	Protein PML
PNPLA2	P28702	Patatin-like phospholipase domain-containing protein 2
PON1	P27169	Serum paraoxonase/arylesterase 1
PPARA	Q03112	Peroxisome proliferator-activated receptor alpha
PPARD	P48544	Peroxisome proliferator-activated receptor delta
PPARG	P23582	Peroxisome proliferator-activated receptor gamma
PPARGC1B	Q09470	Peroxisome proliferator-activated receptor gamma coactivator 1-beta
PPP2CA	P67775	Serine/threonine-protein phosphatase 2A catalytic subunit alpha isoform
PPP2CB	Q7Z417	Serine/threonine-protein phosphatase 2A catalytic subunit beta isoform
PRDM16	Q96S42	Histone-lysine N-methyltransferase PRDM16
PRKAA1	Q9NS61	5'-AMP-activated protein kinase catalytic subunit alpha-1
PRKAA2	P00750	5'-AMP-activated protein kinase catalytic subunit alpha-2
PRKAB1	P35228	5'-AMP-activated protein kinase subunit beta-1
PRKAB2	Q15111	5'-AMP-activated protein kinase subunit beta-2
PRKACA	P17612	cAMP-dependent protein kinase catalytic subunit alpha
PRKAG1	O60760	5'-AMP-activated protein kinase subunit gamma-1
PRKAG2	O60733	5'-AMP-activated protein kinase subunit gamma-2
PRKAG3	P0DMV8	5'-AMP-activated protein kinase subunit gamma-3
PRKCA	Q13131	Protein kinase C alpha type
PRKCB	P01308	Protein kinase C beta type
PRKCD	Q05655	Protein kinase C delta type
PRKCE	Q02156	Protein kinase C epsilon type
PROC	O95863	Vitamin K-dependent protein C
PROS1	P35610	Vitamin K-dependent protein S

PROZ	P10828	Vitamin K-dependent protein Z
PRSS1	P07477	Trypsin-1
PSMD3	O43242	26S proteasome non-ATPase regulatory subunit 3
PTGDR2	Q03181	Prostaglandin D2 receptor 2
PTGER1	Q12809	Prostaglandin E2 receptor EP1 subtype
PTGER2	Q9UHC9	Prostaglandin E2 receptor EP2 subtype
PTGER3	P09455	Prostaglandin E2 receptor EP3 subtype
PTGER4	Q9UHC9	Prostaglandin E2 receptor EP4 subtype
PTGES	O14684	Prostaglandin E synthase
PTGES2	Q9H7Z7	Prostaglandin E synthase 2
PTGIR	P13631	Prostacyclin receptor
PTGIS	Q9NZV8	Prostacyclin synthase
PTGR2	Q9P2S2	Prostaglandin reductase 2
PTGS1	Q9NQ66	Prostaglandin G/H synthase 1
PTGS2	Q14654	Prostaglandin G/H synthase 2
PTK2	Q05397	Focal adhesion kinase 1
PTPN2	P43490	Tyrosine-protein phosphatase non-receptor type 2
RAB3A	O00305	Ras-related protein Rab-3A
RAF1	P04049	RAF proto-oncogene serine/threonine-protein kinase
RAPGEF2	O15296	Rap guanine nucleotide exchange factor 2
RARA	Q8NBN7	Retinoic acid receptor alpha
RARB	P29590	Retinoic acid receptor beta
RARG	Q9NYG8	Retinoic acid receptor gamma
RARRES1	Q96NR8	Retinoic acid receptor responder protein 1
RASA1	P20936	Ras GTPase-activating protein 1
RASSF1	Q9NS23	Ras association domain-containing protein 1
RB1	P06400	Retinoblastoma-associated protein
RBP1	Q13002	Retinol-binding protein 1
RBP3	O60427	Retinol-binding protein 3
RDH11	Q16445	Retinol dehydrogenase 11
RDH12	P42263	Retinol dehydrogenase 12
RDH13	P42262	Retinol dehydrogenase 13
RDH14	Q05586	Retinol dehydrogenase 14
RDH5	P47870	Retinol dehydrogenase 5
RDH8	Q99928	Retinol dehydrogenase 8
RELA	Q04206	Transcription factor p65
RET	P35354	Proto-oncogene tyrosine-protein kinase receptor Ret
RETSAT	Q96KN9	All-trans-retinol 13,14-reductase
RGCC	P17252	Regulator of cell cycle RGCC
RLBP1	Q8N1C3	Retinaldehyde-binding protein 1
RNASE1	O94778	Ribonuclease pancreatic
RNASE2	P20292	Non-secretory ribonuclease
RNASE8	Q9UM73	Ribonuclease 8
RPL7A	P22891	60S ribosomal protein L7a

RPS6KA1	P29475	Ribosomal protein S6 kinase alpha-1
RPS6KA2	Q9UK17	Ribosomal protein S6 kinase alpha-2
RPS6KA3	P10745	Ribosomal protein S6 kinase alpha-3
RPS6KA6	P48051	Ribosomal protein S6 kinase alpha-6
RUNX1T1	Q06455	Protein CBFA2T1
RUNX2	Q13950	Runt-related transcription factor 2
RXRA	P55055	Retinoic acid receptor RXR-alpha
RXRB	Q92806	Retinoic acid receptor RXR-beta
RXRG	Q96NR8	Retinoic acid receptor RXR-gamma
RYR2	P11684	Ryanodine receptor 2
RYR3	P05937	Ryanodine receptor 3
S100A8	Q9Y5K3	Protein S100-A8
S100A9	Q14003	Protein S100-A9
S100G	Q8NFJ5	Protein S100-G
SC5D	Q9UEF7	Lathosterol oxidase
SCD	P20336	Stearoyl-CoA desaturase
SCD5	P06702	Stearoyl-CoA desaturase 5
SCGB1A1	P37288	Uteroglobin
SCN10A	Q06432	Sodium channel protein type 10 subunit alpha
SCN5A	P22303	Sodium channel protein type 5 subunit alpha
SEC14L2	Q8TDE3	SEC14-like protein 2
SEC14L3	Q96RI1	SEC14-like protein 3
SEC14L4	Q8TDS5	SEC14-like protein 4
SELE	P16581	E-selectin
SELP	P16109	P-selectin
SERPINB7	P28223	Serpin B7
SERPINE1	P05121	Plasminogen activator inhibitor 1
SERPINH1	P15172	Serpin H1
SHANK3	P00966	SH3 and multiple ankyrin repeat domains protein 3
SHBG	Q9Y5B6	Sex hormone-binding globulin
SLC12A1	P62424	Solute carrier family 12 member 1
SLC12A2	Q92781	Solute carrier family 12 member 2
SLC12A4	Q9UGJ0	Solute carrier family 12 member 4
SLC12A5	P37231	Solute carrier family 12 member 5
SLC12A6	Q9HDB5	Solute carrier family 12 member 6
SLC12A7	O75469	Solute carrier family 12 member 7
SLC16A12	Q96RI1	Monocarboxylate transporter 12
SLC17A7	Q9H2A2	Vesicular glutamate transporter 1
SLC18A1	Q9H4X1	Chromaffin granule amine transporter
SLC18A2	P07998	Synaptic vesicular amine transporter
SLC25A4	Q96EY1	ADP/ATP translocase 1
SLC29A1	Q9Y666	Equilibrative nucleoside transporter 1
SLC29A2	O75762	Equilibrative nucleoside transporter 2
SLC2A2	P34096	Solute carrier family 2, facilitated glucose transporter member 2

SLC2A4	P14672	Solute carrier family 2, facilitated glucose transporter member 4
SLC35G1	Q9Y5Y9	Solute carrier family 35 member G1
SLC3A2	Q8TCT1	4F2 cell-surface antigen heavy chain
SLC6A2	P23975	Sodium-dependent noradrenaline transporter
SLC6A4	P43119	Sodium-dependent serotonin transporter
SLC6A8	P11142	Sodium- and chloride-dependent creatine transporter 1
SLC8A1	P0DMV9	Sodium/calcium exchanger 1
SLC8A2	Q63ZY3	Sodium/calcium exchanger 2
SLC8A3	P34995	Sodium/calcium exchanger 3
SLC8B1	P06756	Mitochondrial sodium/calcium exchanger protein
SLC9A1	Q07869	Sodium/hydrogen exchanger 1
SNAI1	Q8TF08	Zinc finger protein SNAI1
SNAI2	Q8TDU6	Zinc finger protein SNAI2
SNCA	O00459	Alpha-synuclein
SNTG2	P16050	Gamma-2-syntrophin
SNW1	P09488	SNW domain-containing protein 1
SOAT1	Q06787	Sterol O-acyltransferase 1
SOD1	P00441	Superoxide dismutase [Cu-Zn]
SOX15	O14880	Protein SOX-15
SOX9	P10826	Transcription factor SOX-9
SP1	O15530	Transcription factor Sp1
SP3	P05109	Transcription factor Sp3
SPARC	P36896	SPARC
SPP1	P10451	Osteopontin
SRC	P12931	Tyrosine-protein kinase SRC
STAT1	P42224	Signal transducer and activator of transcription 1-alpha/beta
STK17B	P11168	Serine/threonine-protein kinase 17B
STX1A	P15291	Syntaxin-1A
STX3	P17275	Syntaxin-3
SYK	P43405	Tyrosine-protein kinase SYK
TBC1D32	P23219	Protein broad-minded
TCF3	Q15413	Transcription factor E2-alpha
TGFB1	P01137	Transforming growth factor beta-1
THBD	P16083	Thrombomodulin
THRA	P28472	Thyroid hormone receptor alpha
THRB	P78417	Thyroid hormone receptor beta
TNF	Q13133	Tumor necrosis factor
TNFRSF1B	P20333	Tumor necrosis factor receptor superfamily member 1B
TNK2	P05093	Activated CDC42 kinase 1
TOP1	P11387	DNA topoisomerase 1
TOP2A	P11388	DNA topoisomerase 2-alpha
TP53	Q13018	Cellular tumor antigen p53
TPO	P02652	Thyroid peroxidase
TRIM24	Q8NFJ5	Transcription intermediary factor 1-alpha

TRPA1	O76054	Transient receptor potential cation channel subfamily A member 1
TRPM8	P43699	Transient receptor potential cation channel subfamily M member 8
TRPV1	P48549	Transient receptor potential cation channel subfamily V member 1
TSPO	Q92953	Putative peripheral benzodiazepine receptor-related protein
TXNDC12	P78411	Thioredoxin domain-containing protein 12
UCP2	P55851	Mitochondrial uncoupling protein 2
UGT3A1	P50454	UDP-glucuronosyltransferase 3A1
VCAM1	P11473	Vascular cell adhesion protein 1
VDR	Q8N2Q7	Vitamin D3 receptor
VKORC1	Q9P2U7	Vitamin K epoxide reductase complex subunit 1
VKORC1L1	P37840	Vitamin K epoxide reductase complex subunit 1-like protein 1
WDR77	P19838	Methylosome protein 50
WNT11	P06401	Protein Wnt-11
WNT4	Q9GZV9	Protein Wnt-4
XDH	P47989	Xanthine dehydrogenase
ZNF536	P22460	Zinc finger protein 536
ZPR1	Q08828	Zinc finger protein ZPR1
