# Supplementary material for: Exploring Molecular Mechanisms of Aloe barbadmsis Miller on Diphenoxylate-Induced Constipation in Mice
Source: Evid Based Complement Alternat Med. 2022 May 6;2022:6225758. doi: 10.1155/2022/6225758 (PMC9106447; doi:10.1155/2022/6225758)
Supplement: Supplementary Materials — Table S1. Active ingredients of Aloe. Table S2. Potential targets related to active ingredients. Table S3. potential targets related to constipation. Table S4. Common targets related to active ingredients. Table S5. Table S5-1. Detailed information of BP enrichment of PPI network cluster 1 targets; Table S5-2. Detailed information of CC enrichment of PPI network cluster 1 targets; Table S5-3. Detailed information of MF enrichment of PPI network cluster 1 targets; Table S5-4. Detailed information of KEGG pathways enrichment of PPI network cluster 1 targets. Table S6. Table S6-1. Detailed information of BP enrichment of common targets; Table S6-2. Detailed information of CC enrichment of common targets; Table S6-3. Detailed information of MF enrichment of common targets; Table S6-4. Detailed information of KEGG pathways enrichment of common targets. Table S7. Original images of H&E staining in colon of three repeats in each group. Table S8. Raw data of 5-HT, SP, and VIP in serum and colon determined by ELISA kits. Table S9. Raw data of NF-κB p65, AKT, ERK, and JNK in colon determined by RT-PCR method. Table S10. Original images of ERK, JNK, AKT, and NF-κB p65 in colon of Western Blot, and its raw data quantification. [file 6225758.f1.zip › suppl table 1-10/Table S3 (1) (1).pdf]

Supplementory Table S3: potential targets related to constipation

Gene symbol	Uniprot ID	Description
ABCB1	P08183	ATP Binding Cassette Subfamily B Member 1
ABCC9	O60706	ATP Binding Cassette Subfamily C Member 9
ABCD1	P33897	ATP Binding Cassette Subfamily D Member 1
ABCG2	Q9UNQ0	ATP Binding Cassette Subfamily G Member 2 (Junior Blood Group)
ABL1	P00519	ABL proto-oncogene 1, non-receptor tyrosine kinase
ACE	P12821	Angiotensin I Converting Enzyme
ACHE	P22303	Acetylcholinesterase (Cartwright Blood Group)
ACTB	P60709	Actin Beta
ACTC1	P68032	Actin Alpha Cardiac Muscle 1
ACTG2	P63267	actin gamma 2, smooth muscle
ACVR1B	P36896	Activin A Receptor Type 1B
ACVR2A	P27037	Activin A Receptor Type 2A
ADAR	P55265	Adenosine Deaminase RNA Specific
ADAT3	Q96EY9	Adenosine Deaminase TRNA Specific 3
ADCYAP1	P18509	Adenylate Cyclase Activating Polypeptide 1
ADH1C	P00326	alcohol dehydrogenase 1C (class I), gamma polypeptide
ADNP	Q9H2P0	Activity Dependent Neuroprotector Homeobox
ADRA2A	P08913	Adrenoceptor Alpha 2A
AFF4	Q9UHB7	AF4/FMR2 Family Member 4
AFP	P02771	Alpha Fetoprotein
AIRE	O43918	Autoimmune Regulator
AKR1B1	P15121	Aldo-Keto Reductase Family 1 Member B
AKT1	P31749	AKT Serine/Threonine Kinase 1
ALAD	P13716	aminolevulinate dehydratase
ALAS1	P13196	5'-Aminolevulinate Synthase 1
ALAS2	P22557	5'-Aminolevulinate Synthase 2
ALB	P02768	Albumin
ALDOB	P05062	aldolase, fructose-bisphosphate B
ALK	Q9UM73	ALK Receptor Tyrosine Kinase
ALPL	P05186	alkaline phosphatase, biomineralization associated
ALPP	P05187	Alkaline Phosphatase, Placental
AMER1	Q5JTC6	APC Membrane Recruitment Protein 1
ANO1	Q5XXA6	Anoctamin 1
ANO5	Q75V66	Anoctamin 5
AP2S1	P53680	Adaptor Related Protein Complex 2 Subunit Sigma 1
AP4E1	Q9UPM8	Adaptor Related Protein Complex 4 Subunit Epsilon 1
APBB1	O00213	Amyloid Beta Precursor Protein Binding Family B Member 1
APC	P25054	APC regulator of WNT signaling pathway
APC2	O95996	APC Regulator Of WNT Signaling Pathway 2
APOB	P04114	Apolipoprotein B
APOE	P02649	Apolipoprotein E
APP	P05067	Amyloid Beta Precursor Protein

AQP2	P41181	aquaporin 2
AQP3	Q92482	Aquaporin 3 (Gill Blood Group)
AQP4	P55087	Aquaporin 4
AQP7	O14520	Aquaporin 7
AQP9	O43315	Aquaporin 9
ARID1A	O14497	AT-Rich Interaction Domain 1A
ARID1B	Q8NFD5	AT-Rich Interaction Domain 1B
ARID2	Q68CP9	AT-rich interaction domain 2
ARNT2	Q9HBZ2	aryl hydrocarbon receptor nuclear translocator 2
ARRB2	P32121	Arrestin Beta 2
ARSJ	Q5FYB0	Arylsulfatase Family Member J
ARTN	Q5T4W7	Artemin
ARVCF	O00192	ARVCF delta catenin family member
ASCL1	P50553	achaete-scute family bHLH transcription factor 1
ATM	Q13315	ATM Serine/Threonine Kinase
ATN1	P54259	atrophin 1
ATP12A	P54707	ATPase H+/K+ Transporting Non-Gastric Alpha2 Subunit
ATP4A	P20648	ATPase H+/K+ Transporting Subunit Alpha
ATP7B	P35670	ATPase Copper Transporting Beta
ATRX	P46100	ATRX chromatin remodeler
ATXN2	Q99700	ataxin 2
ATXN8OS	P0DMR3	ATXN8 opposite strand lncRNA
AURKA	O14965	Aurora Kinase A
AVP	P01185	Arginine Vasopressin
AVPR2	P30518	arginine vasopressin receptor 2
AXIN1	O15169	Axin 1
AXIN2	Q9Y2T1	Axin 2
B2M	P61769	Beta-2-Microglobulin
BAX	Q07812	BCL2 Associated X, Apoptosis Regulator
BAZ1B	Q9UIG0	bromodomain adjacent to zinc finger domain 1B
BCHE	P06276	Butyrylcholinesterase
BCL10	O95999	BCL10 immune signaling adaptor
BCL2	P10415	BCL2 Apoptosis Regulator
BDNF	P23560	Brain Derived Neurotrophic Factor
BIRC3	Q13489	baculoviral IAP repeat containing 3
BLM	P54132	BLM RecQ Like Helicase
BLVRB	P30043	Biliverdin Reductase B
BMP1	P13497	Bone Morphogenetic Protein 1
BMP2	P12643	Bone Morphogenetic Protein 2
BMP6	P22004	Bone Morphogenetic Protein 6
BMPR1A	P36894	bone morphogenetic protein receptor type 1A
BPTF	Q12830	bromodomain PHD finger transcription factor
BRAF	P15056	B-Raf proto-oncogene, serine/threonine kinase
BRCA1	P38398	BRCA1 DNA repair associated

BRCA2	P51587	BRCA2 DNA Repair Associated
BRIP1	Q9BX63	BRCA1 Interacting Helicase 1
BRWD3	Q6RI45	Bromodomain And WD Repeat Domain Containing 3
BSND	Q8WZ55	Barttin CLCNK Type Accessory Subunit Beta
BUB1	O43683	BUB1 Mitotic Checkpoint Serine/Threonine Kinase
BUB1B	O60566	BUB1 Mitotic Checkpoint Serine/Threonine Kinase B
CACNA1A	O00555	calcium voltage-gated channel subunit alpha1 A
CACNA1B	Q00975	Calcium Voltage-Gated Channel Subunit Alpha1 B
CACNA1C	Q13936	calcium voltage-gated channel subunit alpha1 C
CACNA1S	Q13698	calcium voltage-gated channel subunit alpha1 S
CALB2	P22676	Calbindin 2
CALCA	P01258	Calcitonin Related Polypeptide Alpha
CALR	P27797	Calreticulin
CAMK2B	Q13554	calcium/calmodulin dependent protein kinase II beta
CAMTA1	Q9Y6Y1	calmodulin binding transcription activator 1
CARD14	Q9BXL6	Caspase Recruitment Domain Family Member 14
CASK	O14936	Calcium/Calmodulin Dependent Serine Protein Kinase
CASP10	Q92851	Caspase 10
CASP3	P42574	Caspase 3
CASP8	Q14790	Caspase 8
CASP9	P55211	Caspase 9
CASR	P41180	calcium sensing receptor
CAT	P04040	Catalase
CAV1	Q03135	Caveolin 1
CAVIN1	Q6NZI2	caveolae associated protein 1
CBR1	P16152	Carbonyl Reductase 1
CCDC107	Q8WV48	Coiled-Coil Domain Containing 107
CCK	P06307	Cholecystokinin
CCKBR	P32239	Cholecystokinin B Receptor
CCN2	P29279	Cellular Communication Network Factor 2
CCNA2	P20248	Cyclin A2
CCND1	P24385	Cyclin D1
CCR6	P51684	C-C Motif Chemokine Receptor 6
CCR9	P51686	C-C Motif Chemokine Receptor 9
CD274	Q9NZQ7	CD274 Molecule
CD34	P28906	CD34 Molecule
CD4	P01730	CD4 Molecule
CD40	P25942	CD40 Molecule
CD44	P16070	CD44 Molecule (Indian Blood Group)
CD8A	P01732	CD8a Molecule
CD96	P40200	CD96 molecule
CDC42	P60953	Cell Division Cycle 42
CDC73	Q6P1J9	cell division cycle 73
CDH1	P12830	Cadherin 1

CDK13	Q14004	cyclin dependent kinase 13
CDKL5	O76039	cyclin dependent kinase like 5
CDKN1A	P38936	Cyclin Dependent Kinase Inhibitor 1A
CDKN1B	P46527	Cyclin Dependent Kinase Inhibitor 1B
CDKN1C	P49918	Cyclin Dependent Kinase Inhibitor 1C
CDKN2A	Q8N726	Cyclin Dependent Kinase Inhibitor 2A
CDKN2B	P42772	Cyclin Dependent Kinase Inhibitor 2B
CDKN2C	P42773	Cyclin Dependent Kinase Inhibitor 2C
CDKN3	Q16667	Cyclin Dependent Kinase Inhibitor 3
CDON	Q4KMG0	Cell Adhesion Associated, Oncogene Regulated
CDX2	Q99626	Caudal Type Homeobox 2
CEACAM5	P06731	CEA Cell Adhesion Molecule 5
CEP128	Q6ZU80	Centrosomal Protein 128
CFTR	P13569	CF Transmembrane Conductance Regulator
CHAT	P28329	Choline O-Acetyltransferase
CHCHD2	Q9Y6H1	coiled-coil-helix-coiled-coil-helix domain containing 2
CHD7	Q9P2D1	Chromodomain Helicase DNA Binding Protein 7
CHD8	Q9HCK8	chromodomain helicase DNA binding protein 8
CHGA	P10645	Chromogranin A
CHRM3	P20309	cholinergic receptor muscarinic 3
CHST14	Q8NCH0	carbohydrate sulfotransferase 14
CISD2	Q8N5K1	CDGSH iron sulfur domain 2
CLCN2	P51788	Chloride Voltage-Gated Channel 2
CLCNKA	P51800	Chloride Voltage-Gated Channel Ka
CLCNKB	P51801	chloride voltage-gated channel Kb
CLEC7A	Q9BXN2	C-Type Lectin Domain Containing 7A
CLIP2	Q9UDT6	CAP-Gly domain containing linker protein 2
CLMP	Q9H6B4	CXADR Like Membrane Protein
CNNM2	Q9H8M5	Cyclin And CBS Domain Divalent Metal Cation Transport Mediator 2
CNP	P09543	2',3'-Cyclic Nucleotide 3' Phosphodiesterase
CNR1	P21554	Cannabinoid Receptor 1
CNTNAP2	Q9UHC6	Contactin Associated Protein 2
COL17A1	Q9UMD9	collagen type XVII alpha 1 chain
COL1A1	P02452	Collagen Type I Alpha 1 Chain
COL1A2	P08123	Collagen Type I Alpha 2 Chain
COL4A5	P29400	Collagen Type IV Alpha 5 Chain
COL4A6	Q14031	Collagen Type IV Alpha 6 Chain
COL5A1	P20908	Collagen Type V Alpha 1 Chain
COL5A2	P05997	Collagen Type V Alpha 2 Chain
COL6A1	P12109	collagen type VI alpha 1 chain
COL7A1	Q02388	collagen type VII alpha 1 chain
COMT	P21964	catechol-O-methyltransferase
COQ2	Q96H96	coenzyme Q2, polyprenyltransferase
COX1	P00395	cytochrome c oxidase subunit I

COX2	P00403	cytochrome c oxidase subunit II
COX3	P00414	cytochrome c oxidase III
CP	P00450	Ceruloplasmin
CPOX	P36551	coproporphyrinogen oxidase
CPT2	P23786	Carnitine Palmitoyltransferase 2
CREB3L1	Q96BA8	CAMP Responsive Element Binding Protein 3 Like 1
CREBBP	Q92793	CREB binding protein
CRH	P06850	Corticotropin Releasing Hormone
CRNN	Q9UBG3	Cornulin
CRP	P02741	C-Reactive Protein
CSF2	P04141	Colony Stimulating Factor 2
CSF3	P09919	Colony Stimulating Factor 3
CSNK2A1	P68400	casein kinase 2 alpha 1
CTLA4	P16410	Cytotoxic T-Lymphocyte Associated Protein 4
CTNNA1	P35221	Catenin Alpha 1
CTNNB1	P35222	Catenin Beta 1
CTNND2	Q9UQB3	Catenin Delta 2
CTNS	O60931	cystinosin, lysosomal cystine transporter
CTSD	P07339	Cathepsin D
CXCL8	P10145	C-X-C Motif Chemokine Ligand 8
CYCS	P99999	Cytochrome C, Somatic
CYP1A1	P04798	Cytochrome P450 Family 1 Subfamily A Member 1
CYP1A2	P05177	Cytochrome P450 Family 1 Subfamily A Member 2
CYP24A1	Q07973	Cytochrome P450 Family 24 Subfamily A Member 1
CYP2C19	P33261	Cytochrome P450 Family 2 Subfamily C Member 19
CYP2D6	P10635	Cytochrome P450 Family 2 Subfamily D Member 6
CYP2E1	P05181	Cytochrome P450 Family 2 Subfamily E Member 1
CYP3A4	P08684	Cytochrome P450 Family 3 Subfamily A Member 4
CYP7A1	P22680	Cytochrome P450 Family 7 Subfamily A Member 1
DACT1	Q9NYF0	dishevelled binding antagonist of beta catenin 1
DBH	P09172	Dopamine Beta-Hydroxylase
DCC	P43146	DCC Netrin 1 Receptor
DDC	P20711	dopa decarboxylase
DDHD2	O94830	DDHD Domain Containing 2
DDOST	P39656	dolichyl-diphosphooligosaccharide--protein glycosyltransferase non-[illegible] subunit
DDX3X	O00571	DEAD-box helicase 3 X-linked
DEAF1	O75398	DEAF1 transcription factor
DES	P17661	desmin
DHCR7	Q9UBM7	7-dehydrocholesterol reductase
DHPS	P49366	deoxyhypusine synthase
DISP1	Q96F81	Dispatched RND Transporter Family Member 1
DLC1	Q96QB1	DLC1 Rho GTPase Activating Protein
DLEC1	Q9Y238	DLEC1 Cilia And Flagella Associated Protein

DLK1	P80370	Delta Like Non-Canonical Notch Ligand 1
DLL1	O00548	Delta Like Canonical Notch Ligand 1
DMD	P11532	Dystrophin
DMPK	Q09013	DM1 Protein Kinase
DNAJC13	O75165	DnaJ Heat Shock Protein Family (Hsp40) Member C13
DNAJC6	O75061	DnaJ heat shock protein family (Hsp40) member C6
DNMT1	P26358	DNA Methyltransferase 1
DPF2	Q92785	double PHD fingers 2
DPP4	P27487	Dipeptidyl Peptidase 4
DPYD	Q12882	Dihydropyrimidine Dehydrogenase
DRD2	P14416	Dopamine Receptor D2
DRD4	P21917	Dopamine Receptor D4
DSE	Q9UL01	Dermatan Sulfate Epimerase
DUOX2	Q9NRD8	dual oxidase 2
DUOXA2	Q1HG44	dual oxidase maturation factor 2
DYNC1H1	Q14204	dynein cytoplasmic 1 heavy chain 1
DYRK1A	Q13627	Dual Specificity Tyrosine Phosphorylation Regulated Kinase 1A
EBF3	Q9H4W6	EBF transcription factor 3
ECE1	P42892	endothelin converting enzyme 1
EDAR	Q9UNE0	Ectodysplasin A Receptor
EDN3	P14138	endothelin 3
EDNRB	P24530	endothelin receptor type B
EFEMP1	Q12805	EGF Containing Fibulin Extracellular Matrix Protein 1
EFL1	Q7Z2Z2	elongation factor like GTPase 1
EGF	P01133	Epidermal Growth Factor
EGFR	P00533	Epidermal Growth Factor Receptor
EHHADH	Q08426	Enoyl-CoA Hydratase And 3-Hydroxyacyl CoA Dehydrogenase
EHMT1	Q9H9B1	Euchromatic Histone Lysine Methyltransferase 1
EIF4G1	Q04637	Eukaryotic Translation Initiation Factor 4 Gamma 1
ELAVL3	Q14576	ELAV Like RNA Binding Protein 3
ELN	P15502	elastin
ELP1	O95163	elongator complex protein 1
EME2	A4GXA9	Essential Meiotic Structure-Specific Endonuclease Subunit 2
ENO2	P09104	Enolase 2
EP300	Q09472	E1A binding protein p300
EPC2	Q52LR7	Enhancer Of Polycomb Homolog 2
EPCAM	P16422	epithelial cell adhesion molecule
EPO	P01588	Erythropoietin
ERBB2	P04626	Erb-B2 Receptor Tyrosine Kinase 2
ERCC6	P0DP91	ERCC Excision Repair 6, Chromatin Remodeling Factor
ESR1	P03372	Estrogen Receptor 1
ETS1	P14921	ETS Proto-Oncogene 1, Transcription Factor
EWSR1	Q01844	EWS RNA Binding Protein 1
EXT2	Q93063	exostosin glycosyltransferase 2

F12	P00748	Coagulation Factor XII
F2	P00734	Coagulation Factor II, Thrombin
FAAH	O00519	Fatty Acid Amide Hydrolase
FAN1	Q9Y2M0	FANCD2 and FANCI associated nuclease 1
FARSB	Q9NSD9	Phenylalanyl-TRNA Synthetase Subunit Beta
FAS	P25445	Fas Cell Surface Death Receptor
FASLG	P48023	Fas Ligand
FBN1	P35555	Fibrillin 1
FBXW7	Q969H0	F-Box And WD Repeat Domain Containing 7
FDFT1	P37268	Farnesyl-Diphosphate Farnesyltransferase 1
FECH	P22830	Ferrochelatase
FFAR2	O15552	Free Fatty Acid Receptor 2
FGF12	P61328	Fibroblast Growth Factor 12
FGF13	Q92913	Fibroblast Growth Factor 13
FGF19	O95750	Fibroblast Growth Factor 19
FGF2	P09038	Fibroblast Growth Factor 2
FGF8	P55075	Fibroblast Growth Factor 8
FGFR1	P11362	fibroblast growth factor receptor 1
FGFR2	P21802	Fibroblast Growth Factor Receptor 2
FGFR3	P22607	Fibroblast Growth Factor Receptor 3
FGFR4	P22455	Fibroblast Growth Factor Receptor 4
FHIT	P49789	Fragile Histidine Triad Diadenosine Triphosphatase
FKBP10	Q96AY3	FKBP Prolyl Isomerase 10
FKBP14	Q9NWM8	FKBP Prolyl Isomerase 14
FLCN	Q8NFG4	Folliculin
FLI1	Q01543	Fli-1 proto-oncogene, ETS transcription factor
FLII	Q13045	FLII actin remodeling protein
FLNA	P21333	filamin A
FMR1	Q06787	FMRP Translational Regulator 1
FOS	P01100	Fos Proto-Oncogene, AP-1 Transcription Factor Subunit
FOXA2	Q9Y261	Forkhead Box A2
FOXE1	O00358	forkhead box E1
FOXG1	P55316	forkhead box G1
FOXH1	O75593	Forkhead Box H1
FOXP1	Q9H334	forkhead box P1
FOXP3	Q9BZS1	Forkhead Box P3
FREM2	Q5SZK8	FRAS1 Related Extracellular Matrix 2
FTL	P02792	ferritin light chain
FUZ	Q9BT04	Fuzzy Planar Cell Polarity Protein
FZD3	Q9NPG1	Frizzled Class Receptor 3
FZD6	O60353	Frizzled Class Receptor 6
GABBR2	O75899	Gamma-Aminobutyric Acid Type B Receptor Subunit 2
GABRA3	P34903	gamma-aminobutyric acid type A receptor subunit alpha3
GABRD	O14764	gamma-aminobutyric acid type A receptor subunit delta

GABRG3	Q99928	Gamma-Aminobutyric Acid Type A Receptor Subunit Gamma3
GAD1	Q99259	Glutamate Decarboxylase 1
GAL	P22466	Galanin And GMAP Prepropeptide
GALNT2	Q10471	Polypeptide N-Acetylgalactosaminyltransferase 2
GAPDH	P04406	Glyceraldehyde-3-Phosphate Dehydrogenase
GAS1	P54826	Growth Arrest Specific 1
GAST	P01350	Gastrin
GATAD2B	Q8WXI9	GATA Zinc Finger Domain Containing 2B
GATM	P50440	Glycine Amidinotransferase
GBA	P04062	glucosylceramidase beta
GCG	P01275	glucagon
GCH1	P30793	GTP Cyclohydrolase 1
GDF6	Q6KF10	Growth Differentiation Factor 6
GDNF	P39905	glial cell derived neurotrophic factor
GFAP	P14136	Glial Fibrillary Acidic Protein
GFRA1	P56159	GDNF Family Receptor Alpha 1
GGT1	P19440	Gamma-Glutamyltransferase 1
GHRL	Q9UBU3	Ghrelin And Obestatin Prepropeptide
GIGYF2	Q6Y7W6	GRB10 Interacting GYF Protein 2
GJC2	Q5T442	gap junction protein gamma 2
GLI2	P10070	GLI family zinc finger 2
GLI3	P10071	GLI Family Zinc Finger 3
GLUD2	P49448	glutamate dehydrogenase 2
GMPPA	Q96IJ6	GDP-Mannose Pyrophosphorylase A
GNAS	P63092	GNAS Complex Locus
GNB3	P16520	G Protein Subunit Beta 3
GNPTAB	Q3T906	N-Acetylglucosamine-1-Phosphate Transferase Subunits Alpha And Beta
GNRH1	P01148	Gonadotropin Releasing Hormone 1
GNS	P15586	Glucosamine (N-Acetyl)-6-Sulfatase
GP1BB	P13224	glycoprotein Ib platelet subunit beta
GP6	Q9HCN6	Glycoprotein VI Platelet
GPBAR1	Q8TDU6	G protein-coupled bile acid receptor 1
GPR19	Q15760	G Protein-Coupled Receptor 19
GPT	P24298	Glutamic--Pyruvic Transaminase
GREM1	O60565	Gremlin 1, DAN Family BMP Antagonist
GRIN1	Q05586	glutamate ionotropic receptor NMDA type subunit 1
GRIN2B	Q13224	glutamate ionotropic receptor NMDA type subunit 2B
GRP	P07492	Gastrin Releasing Peptide
GSK3B	P49841	Glycogen Synthase Kinase 3 Beta
GTDC1	Q4AE62	Glycosyltransferase Like Domain Containing 1
GTF2I	P78347	general transcription factor IIi
GTF2IRD1	Q9UHL9	GTF2I repeat domain containing 1
GUCA2A	Q02747	Guanylate Cyclase Activator 2A
GUCY2C	P25092	Guanylate Cyclase 2C

GUSB	P08236	Glucuronidase Beta
H2AC18	Q6FI13	H2A Clustered Histone 18
H3-2	Q5TEC6	H3.2 Histone (Putative)
H3-3A	P84243	H3.3 histone A
HAMP	P81172	Hepcidin Antimicrobial Peptide
HAND2	P61296	Heart And Neural Crest Derivatives Expressed 2
HCRT	O43612	Hypocretin Neuropeptide Precursor
HESX1	Q9UBX0	HESX homeobox 1
HFE	Q30201	homeostatic iron regulator
HGSNAT	Q68CP4	Heparan-Alpha-Glucosaminide N-Acetyltransferase
HIRA	P54198	histone cell cycle regulator
HIVEP2	P31629	HIVEP zinc finger 2
HJV	Q6ZVN8	Hemojuvelin BMP Co-Receptor
HLA-A	P04439	Major Histocompatibility Complex, Class I, A
HLA-DQA1	P01909	Major Histocompatibility Complex, Class II, DQ Alpha 1
HLA-DQB1	P01920	Major Histocompatibility Complex, Class II, DQ Beta 1
HLA-DRB1	P01911	Major Histocompatibility Complex, Class II, DR Beta 1
HMBS	P08397	hydroxymethylbilane synthase
HMGA2	P52926	High Mobility Group AT-Hook 2
HMGCR	P04035	3-Hydroxy-3-Methylglutaryl-CoA Reductase
HMOX1	P09601	Heme Oxygenase 1
HNRNPH2	P55795	heterogeneous nuclear ribonucleoprotein H2
HNRNPK	P61978	heterogeneous nuclear ribonucleoprotein K
HOXA1	P49639	Homeobox A1
HOXB5	P09067	Homeobox B5
HP	P00738	Haptoglobin
HPSE	Q9Y251	Heparanase
HPSE2	Q8WWQ2	heparanase 2 (inactive)
HRAS	P01112	HRas Proto-Oncogene, GTPase
HSP90AA1	P07900	Heat Shock Protein 90 Alpha Family Class A Member 1
HSPA4	P34932	Heat Shock Protein Family A (Hsp70) Member 4
HTR1A	P08908	5-Hydroxytryptamine Receptor 1A
HTR2A	P28223	5-Hydroxytryptamine Receptor 2A
HTR3A	P46098	5-Hydroxytryptamine Receptor 3A
HTR4	Q13639	5-Hydroxytryptamine Receptor 4
HTR7	P34969	5-Hydroxytryptamine Receptor 7
HTRA2	O43464	HtrA serine peptidase 2
HTT	P42858	Huntingtin
HUWE1	Q7Z6Z7	HECT, UBA And WWE Domain Containing E3 Ubiquitin Protein Ligase
ICAM1	P05362	Intercellular Adhesion Molecule 1
ICOSLG	O75144	Inducible T Cell Costimulator Ligand
IFNA2	P01563	Interferon Alpha 2
IFNG	P01579	Interferon Gamma
IFNGR1	P15260	Interferon Gamma Receptor 1

IGF1	P05019	Insulin Like Growth Factor 1
IGF2	P01344	Insulin Like Growth Factor 2
IGF2R	P11717	Insulin Like Growth Factor 2 Receptor
IGFBP3	P17936	Insulin Like Growth Factor Binding Protein 3
IGHE	P01854	Immunoglobulin Heavy Constant Epsilon
IGHMBP2	P38935	immunoglobulin mu DNA binding protein 2
IL10	P22301	Interleukin 10
IL13	P35225	Interleukin 13
IL17A	Q16552	Interleukin 17A
IL17F	Q96PD4	Interleukin 17F
IL17RA	Q96F46	Interleukin 17 Receptor A
IL17RC	Q8NAC3	Interleukin 17 Receptor C
IL18	Q14116	Interleukin 18
IL1A	P01583	Interleukin 1 Alpha
IL1B	P01584	Interleukin 1 Beta
IL2	P60568	Interleukin 2
IL2RA	P01589	Interleukin 2 Receptor Subunit Alpha
IL4	P05112	Interleukin 4
IL5	P05113	Interleukin 5
IL6	P05231	Interleukin 6
INS	P01308	Insulin
INS-IGF2	F8WCM5	INS-IGF2 Readthrough
IQSEC2	Q5JU85	IQ motif and Sec7 domain ArfGEF 2
IRAK1BP1	Q5VVH5	interleukin 1 receptor associated kinase 1 binding protein 1
IRF1	P10914	Interferon Regulatory Factor 1
IRF2BPL	Q9H1B7	interferon regulatory factor 2 binding protein like
IRF5	Q13568	Interferon Regulatory Factor 5
ITGA2	P17301	Integrin Subunit Alpha 2
IYD	Q6PHW0	iodotyrosine deiodinase
JAK3	P52333	Janus Kinase 3
JMJD1C	Q15652	jumonji domain containing 1C
JUN	P05412	Jun Proto-Oncogene, AP-1 Transcription Factor Subunit
KANSL1	Q7Z3B3	KAT8 regulatory NSL complex subunit 1
KCNAB2	Q13303	potassium voltage-gated channel subfamily A regulatory beta subuni
KCNH1	O95259	Potassium Voltage-Gated Channel Subfamily H Member 1
KCNJ1	P48048	potassium inwardly rectifying channel subfamily J member 1
KCNJ16	Q9NPI9	Potassium Inwardly Rectifying Channel Subfamily J Member 16
KCNJ18	B7U540	potassium inwardly rectifying channel subfamily J member 18
KCNQ1	P51787	Potassium Voltage-Gated Channel Subfamily Q Member 1
KDM1A	O60341	lysine demethylase 1A
KDM4C	Q9H3R0	Lysine Demethylase 4C
KDR	P35968	Kinase Insert Domain Receptor
KIF26A	Q9ULI4	Kinesin Family Member 26A
KIF5C	O60282	Kinesin Family Member 5C

KIFBP	Q96EK5	Kinesin Family Binding Protein
KIT	P10721	KIT proto-oncogene, receptor tyrosine kinase
KLB	Q86Z14	Klotho Beta
KLF4	O43474	Kruppel Like Factor 4
KLF6	Q99612	Kruppel Like Factor 6
KLK3	P07288	Kallikrein Related Peptidase 3
KMT2A	Q03164	Lysine Methyltransferase 2A
KMT2C	Q8NEZ4	Lysine Methyltransferase 2C
KMT2E	Q8IZD2	lysine methyltransferase 2E
KRAS	P01116	KRAS proto-oncogene, GTPase
KRT14	P02533	keratin 14
KRT18	P05783	Keratin 18
KRT19	P08727	Keratin 19
KRT20	P35900	Keratin 20
KRT5	P13647	keratin 5
KRT7	P08729	Keratin 7
KRT8	P05787	Keratin 8
L1CAM	P32004	L1 Cell Adhesion Molecule
LAMA3	Q16787	Laminin Subunit Alpha 3
LAMB3	Q13751	Laminin Subunit Beta 3
LAMC2	Q13753	Laminin Subunit Gamma 2
LCT	P09848	Lactase
LEP	P41159	Leptin
LHX3	Q9UBR4	LIM homeobox 3
LHX4	Q969G2	LIM homeobox 4
LIMK1	P53667	LIM domain kinase 1
LMNB1	P20700	lamin B1
LMOD1	P29536	Leiomodin 1
LMX1B	O60663	LIM Homeobox Transcription Factor 1 Beta
LRIG2	O94898	leucine rich repeats and immunoglobulin like domains 2
LRP5	O75197	LDL Receptor Related Protein 5
LRRK2	Q5S007	leucine rich repeat kinase 2
LSM1	O15116	LSM1 Homolog, MRNA Degradation Associated
LYZ	P61626	Lysozyme
LZTS1	Q9Y250	Leucine Zipper Tumor Suppressor 1
MADD	Q8WXG6	MAP Kinase Activating Death Domain
MAGED2	Q9UNF1	MAGE Family Member D2
MAGEL2	Q9UJ55	MAGE family member L2
MALT1	Q9UDY8	MALT1 paracaspase
MAOB	P27338	Monoamine Oxidase B
MAP2K1	Q02750	mitogen-activated protein kinase kinase 1
MAP2K2	P36507	Mitogen-Activated Protein Kinase Kinase 2
MAPK1	P28482	Mitogen-Activated Protein Kinase 1
MAPK14	Q16539	Mitogen-Activated Protein Kinase 14

MAPK3	P27361	Mitogen-Activated Protein Kinase 3
MAPK8	P45983	Mitogen-Activated Protein Kinase 8
MAPT	P10636	microtubule associated protein tau
MBD5	Q9P267	methyl-CpG binding domain protein 5
MC2R	Q01718	melanocortin 2 receptor
MCC	P23508	MCC Regulator Of WNT Signaling Pathway
MDH2	P40926	malate dehydrogenase 2
MDM2	Q00987	MDM2 Proto-Oncogene
MECP2	P51608	methyl-CpG binding protein 2
MED12	Q93074	mediator complex subunit 12
MED12L	Q86YW9	Mediator Complex Subunit 12L
MED13	Q9UHV7	mediator complex subunit 13
MED25	Q71SY5	Mediator Complex Subunit 25
MEF2C	Q06413	Myocyte Enhancer Factor 2C
MEFV	O15553	MEFV innate immuity regulator, pyrin
MEN1	O00255	Menin 1
MET	P08581	MET Proto-Oncogene, Receptor Tyrosine Kinase
MGMT	P16455	O-6-Methylguanine-DNA Methyltransferase
MILR1	Q7Z6M3	Mast Cell Immunoglobulin Like Receptor 1
MIPEP	Q99797	mitochondrial intermediate peptidase
MITF	O75030	melanocyte inducing transcription factor
MLH1	P40692	mutL homolog 1
MLH3	Q9UHC1	mutL homolog 3
MLN	P12872	Motilin
MLNR	O43193	Motilin Receptor
MLXIPL	Q9NP71	MLX interacting protein like
MLYCD	O95822	malonyl-CoA decarboxylase
MME	P08473	Membrane Metalloendopeptidase
MMP1	P03956	matrix metallopeptidase 1
MMP2	P08253	Matrix Metallopeptidase 2
MMP7	P09237	Matrix Metallopeptidase 7
MMP9	P14780	Matrix Metallopeptidase 9
MNX1	P50219	Motor Neuron And Pancreas Homeobox 1
MORC2	Q9Y6X9	MORC Family CW-Type Zinc Finger 2
MPO	P05164	Myeloperoxidase
MRAP	Q8TCY5	melanocortin 2 receptor accessory protein
MRPS34	P82930	mitochondrial ribosomal protein S34
MSH2	P43246	mutS homolog 2
MSH6	P52701	mutS homolog 6
MSL3	Q8N5Y2	MSL Complex Subunit 3
MT-ATP6	P00846	Mitochondrially Encoded ATP Synthase Membrane Subunit 6
MT-CO1	P00395	Mitochondrially Encoded Cytochrome C Oxidase I
MT-CO2	P00403	Mitochondrially Encoded Cytochrome C Oxidase II
MT-CO3	P00414	Mitochondrially Encoded Cytochrome C Oxidase III

MT-CYB	P00156	Mitochondrially Encoded Cytochrome B
MTHFR	P42898	Methylenetetrahydrofolate Reductase
MT-ND1	P03886	Mitochondrially Encoded NADH:Ubiquinone Oxidoreductase Core Subun
MT-ND4	P03905	Mitochondrially Encoded NADH:Ubiquinone Oxidoreductase Core Subun
MT-ND4L	P03901	Mitochondrially Encoded NADH:Ubiquinone Oxidoreductase Core Subun
MT-ND5	P03915	Mitochondrially Encoded NADH:Ubiquinone Oxidoreductase Core Subun
MT-ND6	P03923	Mitochondrially Encoded NADH:Ubiquinone Oxidoreductase Core Subun
MTOR	P42345	mechanistic target of rapamycin kinase
MTUS1	Q9ULD2	Microtubule Associated Scaffold Protein 1
MUC1	P15941	Mucin 1, Cell Surface Associated
MUC16	Q8WXI7	Mucin 16, Cell Surface Associated
MUTYH	Q9UIF7	MutY DNA Glycosylase
MYC	P01106	MYC Proto-Oncogene, BHLH Transcription Factor
MYH11	P35749	Myosin Heavy Chain 11
MYLK	Q15746	Myosin Light Chain Kinase
MYMK	A6NI61	Myomaker, Myoblast Fusion Factor
MYO1B	O43795	Myosin IB
MYO1H	Q8N1T3	Myosin IH
MYOCD	Q8IZQ8	Myocardin
NAB2	Q15742	NGFI-A binding protein 2
NAGA	P17050	Alpha-N-Acetylgalactosaminidase
NAGLU	P54802	N-Acetyl-Alpha-Glucosaminidase
NALCN	Q8IZF0	sodium leak channel, non-selective
NCAM1	P13591	Neural Cell Adhesion Molecule 1
ND1	P03886	NADH dehydrogenase, subunit 1 (complex I)
ND4	P03905	NADH dehydrogenase, subunit 4 (complex I)
ND5	P03915	NADH dehydrogenase, subunit 5 (complex I)
ND6	P03923	NADH dehydrogenase, subunit 6 (complex I)
NDUFAF6	Q330K2	NADH:Ubiquinone Oxidoreductase Complex Assembly Factor 6
NEB	P20929	Nebulin
NF1	P21359	Neurofibromin 1
NFE2L2	Q16236	Nuclear Factor, Erythroid 2 Like 2
NFIX	Q14938	Nuclear Factor I X
NFKB1	P19838	Nuclear Factor Kappa B Subunit 1
NFKBIA	P25963	NFKB Inhibitor Alpha
NGF	P01138	Nerve Growth Factor
NGLY1	Q96IV0	N-Glycanase 1
NKX2-1	P43699	NK2 homeobox 1
NKX2-5	P52952	NK2 homeobox 5
NLRP3	Q96P20	NLR Family Pyrin Domain Containing 3
NNT	Q13423	nicotinamide nucleotide transhydrogenase
NOD2	Q9HC29	Nucleotide Binding Oligomerization Domain Containing 2
NODAL	Q96S42	Nodal Growth Differentiation Factor
NOS1	P29475	Nitric Oxide Synthase 1

NOS2	P35228	Nitric Oxide Synthase 2
NOTCH1	P46531	Notch Receptor 1
NOTCH2NLC	P0DPK4	Notch 2 N-Terminal Like C
NPC1	O15118	NPC Intracellular Cholesterol Transporter 1
NPR3	P17342	Natriuretic Peptide Receptor 3
NPY	P01303	Neuropeptide Y
NQO1	P15559	NAD(P)H Quinone Dehydrogenase 1
NR4A2	P43354	nuclear receptor subfamily 4 group A member 2
NRAS	P01111	NRAS Proto-Oncogene, GTPase
NRTN	Q99748	neurturin
NRXN1	P58400;Q9ULB1	neurexin 1
NSD1	Q96L73	Nuclear Receptor Binding SET Domain Protein 1
NTF3	P20783	Neurotrophin 3
NTN1	O95631	Netrin 1
NTNG1	Q9Y2I2	Netrin G1
NTNG2	Q96CW9	Netrin G2
NTRK1	P04629	Neurotrophic Receptor Tyrosine Kinase 1
NTRK2	Q16620	Neurotrophic Receptor Tyrosine Kinase 2
NTRK3	Q16288	Neurotrophic Receptor Tyrosine Kinase 3
NTS	P30990	Neurotensin
NXPH1	P58417	Neurexophilin 1
OCA2	Q04671	OCA2 Melanosomal Transmembrane Protein
OCLN	Q16625	Occludin
OCRL	Q01968	OCRL inositol polyphosphate-5-phosphatase
ODC1	P11926	Ornithine Decarboxylase 1
OPRK1	P41145	Opioid Receptor Kappa 1
OPRM1	P35372	opioid receptor mu 1
ORC4	O43929	Origin Recognition Complex Subunit 4
OTUD6B	Q8N6M0	OTU Deubiquitinase 6B
OTX2	P32243	orthodenticle homeobox 2
P2RY12	Q9H244	Purinergic Receptor P2Y12
P3H1	Q32P28	Prolyl 3-Hydroxylase 1
P4HTM	Q9NXG6	prolyl 4-hydroxylase, transmembrane
PACRG	Q96M98	Parkin Coregulated
PACS1	Q6VY07	phosphofurin acidic cluster sorting protein 1
PAEP	P09466	Progestagen Associated Endometrial Protein
PAGR1	Q9BTK6	PAXIP1 Associated Glutamate Rich Protein 1
PAH	P00439	Phenylalanine Hydroxylase
PALB2	Q86YC2	Partner And Localizer Of BRCA2
PALLD	Q8WX93	Palladin, Cytoskeletal Associated Protein
PARK7	Q99497	Parkinsonism associated deglycase
PARP1	P09874	Poly(ADP-Ribose) Polymerase 1
PAX3	P23760	Paired Box 3
PAX8	Q06710	paired box 8

PCCA	P05165	propionyl-CoA carboxylase subunit alpha
PCCB	P05166	propionyl-CoA carboxylase subunit beta
PCGF2	P35227	polycomb group ring finger 2
PDGFRA	P16234	platelet derived growth factor receptor alpha
PDGFRL	Q15198	Platelet Derived Growth Factor Receptor Like
PDX1	P52945	Pancreatic And Duodenal Homeobox 1
PDYN	P01213	Prodynorphin
PEX16	Q9Y5Y5	peroxisomal biogenesis factor 16
PGR	P06401	Progesterone Receptor
PHIP	Q8WWQ0	pleckstrin homology domain interacting protein
PHOX2A	O14813	Paired Like Homeobox 2A
PHOX2B	Q99453	paired like homeobox 2B
PIGG	Q5H8A4	Phosphatidylinositol Glycan Anchor Biosynthesis Class G
PIGO	Q8TEQ8	Phosphatidylinositol Glycan Anchor Biosynthesis Class O
PIGS	Q96S52	Phosphatidylinositol Glycan Anchor Biosynthesis Class S
PIGV	Q9NUD9	Phosphatidylinositol Glycan Anchor Biosynthesis Class V
PIK3CA	P42336	phosphatidylinositol-4,5-bisphosphate 3-kinase catalytic subunit alp
PIK3R1	P27986	Phosphoinositide-3-Kinase Regulatory Subunit 1
PINK1	Q9BXM7	PTEN induced kinase 1
PKHD1	P08F94	PKHD1 Ciliary IPT Domain Containing Fibrocystin/Polyductin
PLA2G2A	P14555	Phospholipase A2 Group IIA
PLA2G6	O60733	Phospholipase A2 Group VI
PLAG1	Q6DJT9	PLAG1 Zinc Finger
PLAU	P00749	Plasminogen Activator, Urokinase
PLEC	Q15149	Plectin
PLOD1	Q02809	Procollagen-Lysine,2-Oxoglutarate 5-Dioxygenase 1
PMPCA	Q10713	Peptidase, Mitochondrial Processing Subunit Alpha
PMS1	P54277	PMS1 homolog 1, mismatch repair system component
PMS2	P54278	PMS1 homolog 2, mismatch repair system component
PODXL	O00592	podocalyxin like
POGZ	Q7Z3K3	pogo transposable element derived with ZNF domain
POLD1	P28340	DNA Polymerase Delta 1, Catalytic Subunit
POLE	Q07864	DNA Polymerase Epsilon, Catalytic Subunit
POLG	P54098	DNA polymerase gamma, catalytic subunit
POLG2	Q9UHN1	DNA polymerase gamma 2, accessory subunit
POLR2F	P61218	RNA Polymerase II, I And III Subunit F
POMC	P01189	proopiomelanocortin
PON1	P27169	paraoxonase 1
POU1F1	P28069	POU class 1 homeobox 1
PPARG	P37231	Peroxisome Proliferator Activated Receptor Gamma
PPIG	Q13427	Peptidylprolyl Isomerase G
PPM1D	O15297	protein phosphatase, Mg2+/Mn2+ dependent 1D
PPOX	P50336	protoporphyrinogen oxidase
PPP3CA	Q08209	protein phosphatase 3 catalytic subunit alpha

PRDM16	Q9HAZ2	PR/SET domain 16
PRKCQ	Q04759	Protein Kinase C Theta
PRKN	O60260	parkin RBR E3 ubiquitin protein ligase
PRL	P01236	Prolactin
PRNP	P04156	Prion Protein
PRODH	O43272	Proline Dehydrogenase 1
PROKR2	Q8NFJ6	prokineticin receptor 2
PROP1	O75360	PROP paired-like homeobox 1
PSAP	P07602	Prosaposin
PSMB4	P28070	Proteasome 20S Subunit Beta 4
PSMB8	P28062	Proteasome 20S Subunit Beta 8
PSPN	O60542	Persephin
PTCH1	Q13635	Patched 1
PTEN	P60484	Phosphatase And Tensin Homolog
PTGS1	P23219	Prostaglandin-Endoperoxide Synthase 1
PTGS2	P35354	Prostaglandin-Endoperoxide Synthase 2
PTH	P01270	Parathyroid Hormone
PTPN12	Q05209	Protein Tyrosine Phosphatase Non-Receptor Type 12
PTPN22	Q9Y2R2	Protein Tyrosine Phosphatase Non-Receptor Type 22
PTPN3	P26045	Protein Tyrosine Phosphatase Non-Receptor Type 3
PTPRJ	Q12913	Protein Tyrosine Phosphatase Receptor Type J
PURA	Q00577	Purine Rich Element Binding Protein A
PYY	P10082	Peptide YY
RAD21	O60216	RAD21 Cohesin Complex Component
RAD51	Q06609	RAD51 recombinase
RAD54B	Q9Y620	RAD54 Homolog B
RAI1	Q7Z5J4	retinoic acid induced 1
RB1	P06400	RB Transcriptional Corepressor 1
RBBP8	Q99708	RB Binding Protein 8, Endonuclease
RBFOX1	Q9NWB1	RNA Binding Fox-1 Homolog 1
REN	P00797	Renin
RERE	Q9P2R6	arginine-glutamic acid dipeptide repeats
RET	P07949	ret proto-oncogene
RFC2	P35250	replication factor C subunit 2
RHOBTB2	Q9BYZ6	Rho related BTB domain containing 2
RIF1	Q5UIP0	Replication Timing Regulatory Factor 1
RNF6	Q9Y252	Ring Finger Protein 6
RORA	P35398	RAR Related Orphan Receptor A
RPS20	P60866	ribosomal protein S20
RREB1	Q92766	ras responsive element binding protein 1
RRM2B	Q7LG56	ribonucleotide reductase regulatory TP53 inducible subunit M2B
RTL1	A6NKG5	Retrotransposon Gag Like 1
S100A8	P05109	S100 Calcium Binding Protein A8
S100B	P04271	S100 Calcium Binding Protein B

SAG	P10523	S-Antigen Visual Arrestin
SALL1	Q9NSC2	spalt like transcription factor 1
SAR1B	Q9Y6B6	Secretion Associated Ras Related GTPase 1B
SATB1	Q01826	SATB Homeobox 1
SCAMP4	Q969E2	Secretory Carrier Membrane Protein 4
SCN10A	Q9Y5Y9	sodium voltage-gated channel alpha subunit 10
SCN11A	Q9UI33	sodium voltage-gated channel alpha subunit 11
SCN4A	P35499	Sodium Voltage-Gated Channel Alpha Subunit 4
SCN5A	Q14524	Sodium Voltage-Gated Channel Alpha Subunit 5
SCN9A	Q15858	sodium voltage-gated channel alpha subunit 9
SCNN1A	P37088	sodium channel epithelial 1 subunit alpha
SCNN1B	P51168	sodium channel epithelial 1 subunit beta
SCNN1G	P51170	sodium channel epithelial 1 subunit gamma
SCO2	O43819	Synthesis Of Cytochrome C Oxidase 2
SCT	P09683	Secretin
SDHA	P31040	succinate dehydrogenase complex flavoprotein subunit A
SDHB	P21912	succinate dehydrogenase complex iron sulfur subunit B
SDHC	Q99643	succinate dehydrogenase complex subunit C
SEC24C	P53992	SEC24 homolog C, COPII coat complex component
SEMA3C	Q99985	semaphorin 3C
SEMA3D	O95025	semaphorin 3D
SEMA4A	Q9H3S1	semaphorin 4A
SEMA5A	Q13591	Semaphorin 5A
SERPINA1	P01009	Serpin Family A Member 1
SERPINA3	P01011	Serpin Family A Member 3
SERPINA7	P05543	Serpin Family A Member 7
SERPINF1	P36955	Serpin Family F Member 1
SERPINH1	P50454	Serpin Family H Member 1
SETD1A	O15047	SET Domain Containing 1A, Histone Lysine Methyltransferase
SETD2	Q9BYW2	SET Domain Containing 2, Histone Lysine Methyltransferase
SETD5	Q9C0A6	SET Domain Containing 5
SGO1	Q5FBB7	Shugoshin 1
SGSH	P51688	N-Sulfoglucosamine Sulfohydrolase
SH2B1	Q9NRF2	SH2B Adaptor Protein 1
SHANK3	Q9BYB0	SH3 And Multiple Ankyrin Repeat Domains 3
SHH	Q15465	Sonic Hedgehog Signaling Molecule
SIK3	Q9Y2K2	SIK family kinase 3
SIL1	Q9H173	SIL1 Nucleotide Exchange Factor
SIX3	O95343	SIX homeobox 3
SKI	P12755	SKI proto-oncogene
SLA	Q13239	Src Like Adaptor
SLC12A1	Q13621	solute carrier family 12 member 1
SLC12A2	P55011	Solute Carrier Family 12 Member 2
SLC12A3	P55017	solute carrier family 12 member 3

SLC25A4	P12235	solute carrier family 25 member 4
SLC26A11	Q86WA9	Solute Carrier Family 26 Member 11
SLC26A3	P40879	Solute Carrier Family 26 Member 3
SLC26A4	O43511	solute carrier family 26 member 4
SLC34A1	Q06495	Solute Carrier Family 34 Member 1
SLC35A2	P78381	solute carrier family 35 member A2
SLC40A1	Q9NP59	Solute Carrier Family 40 Member 1
SLC41A1	Q8IVJ1	Solute Carrier Family 41 Member 1
SLC5A5	Q92911	solute carrier family 5 member 5
SLC6A2	P23975	Solute Carrier Family 6 Member 2
SLC6A3	Q01959	solute carrier family 6 member 3
SLC6A4	P31645	Solute Carrier Family 6 Member 4
SLC6A8	P48029	solute carrier family 6 member 8
SLC9A6	Q92581	Solute Carrier Family 9 Member A6
SLC9A7	Q96T83	Solute Carrier Family 9 Member A7
SLC9A9	Q8IVB4	Solute Carrier Family 9 Member A9
SLC9B1	Q4ZJI4	Solute Carrier Family 9 Member B1
SMAD4	Q13485	SMAD Family Member 4
SMC1A	Q14683	Structural Maintenance Of Chromosomes 1A
SMC3	Q9UQE7	Structural Maintenance Of Chromosomes 3
SMN1	Q16637	Survival Of Motor Neuron 1, Telomeric
SMO	Q99835	Smoothened, Frizzled Class Receptor
SMPD1	P17405	sphingomyelin phosphodiesterase 1
SMTN	P53814	Smoothelin
SNAI2	O43623	Snail Family Transcriptional Repressor 2
SNCA	P37840	synuclein alpha
SNCAIP	Q9Y6H5	synuclein alpha interacting protein
SNCB	Q16143	Synuclein Beta
SNRPN	P63162	Small Nuclear Ribonucleoprotein Polypeptide N
SOD1	P00441	Superoxide Dismutase 1
SOD2	P04179	Superoxide Dismutase 2
SOST	Q9BQB4	Sclerostin
SOX10	P56693	SRY-box transcription factor 10
SOX2	P48431	SRY-box transcription factor 2
SOX3	P41225	SRY-box transcription factor 3
SOX4	Q06945	SRY-box transcription factor 4
SOX9	P48436	SRY-Box Transcription Factor 9
SP7	Q8TDD2	Sp7 Transcription Factor
SPARC	P09486	Secreted Protein Acidic And Cysteine Rich
SPART	Q8N0X7	spartin
SPATA5	Q8NB90	Spermatogenesis Associated 5
SPINK1	P00995	Serine Peptidase Inhibitor Kazal Type 1
SPOP	O43791	Speckle Type BTB/POZ Protein
SRC	P12931	SRC Proto-Oncogene, Non-Receptor Tyrosine Kinase

SRCAP	Q6ZRS2	Snf2 related CREBBP activator protein
SST	P61278	Somatostatin
STAG2	Q8N3U4	Stromal Antigen 2
STAR	P49675	steroidogenic acute regulatory protein
STAT1	P42224	Signal Transducer And Activator Of Transcription 1
STAT3	P40763	Signal Transducer And Activator Of Transcription 3
STAT6	P42226	signal transducer and activator of transcription 6
STK11	Q15831	Serine/Threonine Kinase 11
STK39	Q9UEW8	Serine/Threonine Kinase 39
STXBP1	P61764	Syntaxin Binding Protein 1
SYNJ1	O43426	Synaptojanin 1
SYP	P08247	Synaptophysin
TAC1	P20366	Tachykinin Precursor 1
TAC3	Q9UHF0	Tachykinin Precursor 3
TACR1	P25103	Tachykinin Receptor 1
TACR2	P21452	Tachykinin Receptor 2
TACR3	P29371	Tachykinin Receptor 3
TAF1	P21675	TATA-box binding protein associated factor 1
TANC2	Q9HCD6	Tetratricopeptide Repeat, Ankyrin Repeat And Coiled-Coil Containing 2
TBCD	Q9BTW9	tubulin folding cofactor D
TBL1X	O60907	Transducin Beta Like 1 X-Linked
TBL2	Q9Y4P3	transducin beta like 2
TBP	P20226	TATA-box binding protein
TBX1	O43435	T-box transcription factor 1
TCERG1	O14776	Transcription Elongation Regulator 1
TCF20	Q9UGU0	transcription factor 20
TCF3	P15923	Transcription Factor 3
TCF4	P15884	transcription factor 4
TCOF1	Q13428	Treacle Ribosome Biogenesis Factor 1
TDGF1	P13385	Teratocarcinoma-Derived Growth Factor 1
TDO2	P48775	Tryptophan 2,3-Dioxygenase
TERT	O14746	Telomerase Reverse Transcriptase
TFR2	Q9UP52	Transferrin Receptor 2
TFRC	P02786	Transferrin Receptor
TG	P01266	thyroglobulin
TGFB1	P01137	Transforming Growth Factor Beta 1
TGFB2	P61812	Transforming Growth Factor Beta 2
TGFBR2	P37173	transforming growth factor beta receptor 2
TGIF1	Q15583	TGFB Induced Factor Homeobox 1
TGM2	P21980	Transglutaminase 2
TH	P07101	tyrosine hydroxylase
THRA	P10827	thyroid hormone receptor alpha
THRB	P10828	thyroid hormone receptor beta
TIMP1	P01033	TIMP Metallopeptidase Inhibitor 1

TJP1	Q07157	Tight Junction Protein 1
TLK2	Q86UE8	tousled like kinase 2
TLR2	O60603	Toll Like Receptor 2
TLR4	O00206	Toll Like Receptor 4
TLR9	Q9NR96	Toll Like Receptor 9
TLX2	O43763	T Cell Leukemia Homeobox 2
TMEM38B	Q9NVV0	Transmembrane Protein 38B
TMEM71	Q6P5X7	Transmembrane Protein 71
TMEM94	Q12767	transmembrane protein 94
TNF	P01375	Tumor Necrosis Factor
TNFRSF10A	O00220	TNF Receptor Superfamily Member 10a
TNFRSF1A	P19438	TNF receptor superfamily member 1A
TNFSF15	O95150	TNF Superfamily Member 15
TONSL	Q96HA7	Tonsoku Like, DNA Repair Protein
TP53	P04637	Tumor Protein P53
TPH1	P17752	Tryptophan Hydroxylase 1
TPO	P07202	thyroid peroxidase
TRAF3IP2	O43734	TRAF3 Interacting Protein 2
TRH	P20396	thyrotropin releasing hormone
TRHR	P34981	thyrotropin releasing hormone receptor
TRIO	O75962	Trio Rho Guanine Nucleotide Exchange Factor
TRIP12	Q14669	thyroid hormone receptor interactor 12
TRPA1	O75762	Transient Receptor Potential Cation Channel Subfamily A Member 1
TRPV1	Q8NER1	Transient Receptor Potential Cation Channel Subfamily V Member 1
TRPV6	Q9H1D0	Transient Receptor Potential Cation Channel Subfamily V Member 6
TSC1	Q92574	TSC Complex Subunit 1
TSC2	P49815	TSC Complex Subunit 2
TSHB	P01222	thyroid stimulating hormone subunit beta
TSHR	P16473	thyroid stimulating hormone receptor
TTR	P02766	transthyretin
TUBB	P07437	Tubulin Beta Class I
TUBB1	Q9H4B7	Tubulin Beta 1 Class VI
TWNK	Q96RR1	twinkle mtDNA helicase
TXNRD2	Q9NNW7	thioredoxin reductase 2
TYMP	P19971	thymidine phosphorylase
UBE2A	P49459	Ubiquitin Conjugating Enzyme E2 A
UBE3A	Q05086	ubiquitin protein ligase E3A
UBE3B	Q7Z3V4	ubiquitin protein ligase E3B
UCHL1	P09936	ubiquitin C-terminal hydrolase L1
UFC1	Q9Y3C8	ubiquitin-fold modifier conjugating enzyme 1
UFD1	Q92890	ubiquitin recognition factor in ER associated degradation 1
UGP2	Q16851	UDP-Glucose Pyrophosphorylase 2
UNC80	Q8N2C7	unc-80 homolog, NALCN channel complex subunit
UPF3B	Q9BZI7	UPF3B Regulator Of Nonsense Mediated MRNA Decay

UQCRFS1	P47985	Ubiquinol-Cytochrome C Reductase, Rieske Iron-Sulfur Polypeptide 1
UROD	P06132	Uroporphyrinogen Decarboxylase
UROS	P10746	Uroporphyrinogen III Synthase
USH2A	O75445	Usherin
USP7	Q93009	ubiquitin specific peptidase 7
VANGL1	Q8TAA9	VANGL planar cell polarity protein 1
VEGFA	P15692	Vascular Endothelial Growth Factor A
VIM	P08670	Vimentin
VIP	P01282	Vasoactive Intestinal Peptide
VPS11	Q9H270	VPS11 core subunit of CORVET and HOPS complexes
VPS13B	Q7Z7G8	vacuolar protein sorting 13 homolog B
VPS13C	Q709C8	vacuolar protein sorting 13 homolog C
VPS35	Q96QK1	VPS35 Retromer Complex Component
VPS51	Q9UID3	VPS51 Subunit Of GARP Complex
WAC	Q9BTA9	WW domain containing adaptor with coiled-coil
WASF1	Q92558	WASP Family Member 1
WDFY1	Q8IWB7	WD Repeat And FYVE Domain Containing 1
WDR1	O75083	WD Repeat Domain 1
WDR26	Q9H7D7	WD repeat domain 26
WFS1	O76024	wolframin ER transmembrane glycoprotein
WNT1	P04628	Wnt Family Member 1
WT1	P19544	WT1 Transcription Factor
WWOX	Q9NZC7	WW Domain Containing Oxidoreductase
ZBED2	Q9BTP6	Zinc Finger BED-Type Containing 2
ZEB1	P37275	Zinc Finger E-Box Binding Homeobox 1
ZEB2	O60315	Zinc Finger E-Box Binding Homeobox 2
ZIC2	O95409	Zic Family Member 2
ZMIZ1	Q9ULJ6	Zinc Finger MIZ-Type Containing 1
ZMYND11	Q15326	Zinc Finger MYND-Type Containing 11
ZNF292	O60281	Zinc Finger Protein 292
ZNF41	P51814	Zinc Finger Protein 41
ZSWIM6	Q9HCJ5	zinc finger SWIM-type containing 6
