# Supplementary material for: Exploring Molecular Mechanisms of Aloe barbadmsis Miller on Diphenoxylate-Induced Constipation in Mice
Source: Evid Based Complement Alternat Med. 2022 May 6;2022:6225758. doi: 10.1155/2022/6225758 (PMC9106447; doi:10.1155/2022/6225758)
Supplement: Supplementary Materials — Table S1. Active ingredients of Aloe. Table S2. Potential targets related to active ingredients. Table S3. potential targets related to constipation. Table S4. Common targets related to active ingredients. Table S5. Table S5-1. Detailed information of BP enrichment of PPI network cluster 1 targets; Table S5-2. Detailed information of CC enrichment of PPI network cluster 1 targets; Table S5-3. Detailed information of MF enrichment of PPI network cluster 1 targets; Table S5-4. Detailed information of KEGG pathways enrichment of PPI network cluster 1 targets. Table S6. Table S6-1. Detailed information of BP enrichment of common targets; Table S6-2. Detailed information of CC enrichment of common targets; Table S6-3. Detailed information of MF enrichment of common targets; Table S6-4. Detailed information of KEGG pathways enrichment of common targets. Table S7. Original images of H&E staining in colon of three repeats in each group. Table S8. Raw data of 5-HT, SP, and VIP in serum and colon determined by ELISA kits. Table S9. Raw data of NF-κB p65, AKT, ERK, and JNK in colon determined by RT-PCR method. Table S10. Original images of ERK, JNK, AKT, and NF-κB p65 in colon of Western Blot, and its raw data quantification. [file 6225758.f1.zip › suppl table 1-10/Table S4 (1) (1).pdf]

Supplementory Table S4: Common targets related to active ingredients

Gene symbol	Uniprot ID	Description	Active ingredients Mol ID	Active ingredients Name
MMP2	P08253	72 kDa type IV collagenase	MOL002773	beta-carotene
SLC6A2	P23975	Sodium-dependent noradrenaline transporter	MOL001439	arachidonic acid
IGF1	P30838	Insulin-like growth factor I	MOL002773	beta-carotene
PLAU	P00749	Urokinase-type plasminogen activator	MOL000098	quercetin
NOS2	P62714	Nitric oxide synthase, inducible	MOL001439	arachidonic acid
SLC6A4	P43119	Sodium-dependent serotonin transporter	MOL001439	arachidonic acid
MET	P08581	Hepatocyte growth factor receptor	MOL000098	quercetin
IRF1	P10914	Interferon regulatory factor 1	MOL000098	quercetin
LEP	Q03154	Leptin	MOL002773	beta-carotene
NOS1	Q96PR1	Nitric oxide synthase, brain	MOL001439	arachidonic acid
COL1A1	P17658	Collagen alpha-1	MOL001439	arachidonic acid
DPP4	P27487	Dipeptidyl peptidase 4	MOL000098	quercetin
PLA2G2A	P46098	Phospholipase A2, membrane associated	MOL001439	arachidonic acid
ALK	Q9UM73	ALK tyrosine kinase receptor	MOL000098	quercetin
APOE	Q9NR19	Apolipoprotein E	MOL002773	beta-carotene
FAS	Q16671	Tumor necrosis factor receptor superfamily member 6	MOL002773	beta-carotene
PON1	P27169	Serum paraoxonase/arylesterase 1	MOL000098	quercetin
PRDM16	Q96S42	Histone-lysine N-methyltransferase PRDM16	MOL001439	arachidonic acid
BCHE	P19087	Cholinesterase	MOL000953	CLR
BCL2	P10415	Apoptosis regulator Bcl-2	MOL002773	beta-carotene
THRB	P78417	Thyroid hormone receptor beta	MOL000953	CLR
ADRA2A	Q86YN6	Alpha-2A adrenergic receptor	MOL001439	arachidonic acid
CYP1A1	P04798	Cytochrome P450 1A1	MOL000098	quercetin

DUOX2	Q9NRD8	Dual oxidase 2	MOL000098	quercetin
HTR3A	O43623	Glutamate receptor 3	MOL000098	quercetin
CASP8	Q14790	Caspase-8	MOL002773	beta-carotene
OPRK1	P00742	Coagulation factor X	MOL000953	CLR
PPARG	P37231	Peroxisome proliferator-activated receptor gamma	MOL001439	arachidonic acid
CACNA1B	P33121	Voltage-dependent N-type calcium channel subunit alpha-1B	MOL002773	beta-carotene
CRP	P02741	C-reactive protein	MOL000098	quercetin
RET	P35354	Prostaglandin G/H synthase 2	MOL001439	arachidonic acid
MAPT	P10636	Microtubule-associated protein tau	MOL000098	quercetin
CXCL8	P10145	Interleukin-8	MOL000098	quercetin
SLC12A2	Q92781	Glutathione reductase, mitochondrial	MOL001439	arachidonic acid
ABCB1	P24310	ATP-dependent translocase ABCB1	MOL005043	campest-5-en-3beta-ol
NFE2L2	Q16236	Nuclear factor erythroid 2-related factor 2	MOL000098	quercetin
PLA2G6	P51812	85/88 kDa calcium-independent phospholipase A2	MOL001439	arachidonic acid
SOX9	P10826	Transcription factor SOX-9	MOL001439	arachidonic acid
TNF	P01375	Tumor necrosis factor	MOL000471	aloe-emodin
IL1A	P01583	Interleukin-1 alpha	MOL000098	quercetin
DRD2	P04278	D	MOL000098	quercetin
MPO	P05164	Myeloperoxidase	MOL000098	quercetin
GRIN2B	P42330	Retinol dehydrogenase 12	MOL002773	beta-carotene
F2	P00734	Prothrombin	MOL000098	quercetin
TRPA1	O76054	Fibroblast growth factor 2	MOL001439	arachidonic acid
PTGS2	P35354	Prostaglandin G/H synthase 2	MOL002773	beta-carotene
CTNNB1	P35222	Catenin beta-1	MOL002773	beta-carotene
ADH1C	Q8NE71	Alcohol dehydrogenase 1C	MOL002773	beta-carotene
P2RY12	P47712	Cytosolic phospholipase A2	MOL001439	arachidonic acid

CCND1	P24385	G1/S-specific cyclin-D1	MOL001439	arachidonic acid
CYP2E1	P04040	Cytochrome P450 2E1	MOL002773	beta-carotene
ESR1	P03372	Estrogen receptor	MOL005060	aloinum
GABRG3	Q07912	Gamma-aminobutyric acid receptor subunit gamma-3	MOL000098	quercetin
SNCA	O00459	Alpha-synuclein	MOL001439	arachidonic acid
CTSD	P07339	Cathepsin D	MOL000098	quercetin
TGFB1	P01137	Transforming growth factor beta-1	MOL000098	quercetin
SHANK3	P00966	SH3 and multiple ankyrin repeat domains protein 3	MOL002773	beta-carotene
MYC	P01106	Myc proto-oncogene protein	MOL002773	beta-carotene
NRXN1	P08588	Neurexin-1-beta	MOL002773	beta-carotene
AVPR2	P30518	Vasopressin V2 receptor	MOL000098	quercetin
S100A8	Q9Y5K3	Protein S100-A8	MOL001439	arachidonic acid
NKX2-1	P09917	Homeobox protein Nkx-2.1	MOL002773	beta-carotene
IL13	Q9HBH5	Interleukin-13	MOL001439	arachidonic acid
ACHE	P22303	Acetylcholinesterase	MOL000098	quercetin
GSK3B	P49841	Glycogen synthase kinase-3 beta	MOL000098	quercetin
MMP1	P03956	Interstitial collagenase	MOL002773	beta-carotene
STAT1	P42224	Signal transducer and activator of transcription 1-alpha/beta	MOL000098	quercetin
IL6	P05231	Interleukin-6	MOL000098	quercetin
KCNQ1	Q01668	Potassium voltage-gated channel subfamily D member 3	MOL002773	beta-carotene
TRPV1	Q8NER1	Transient receptor potential cation channel subfamily V member 1	MOL001439	arachidonic acid
HSP90AA1	P07900	Heat shock protein HSP 90-alpha	MOL000471	aloe-emodin
CASP3	P42574	Caspase-3	MOL002773	beta-carotene
PARP1	P09874	Poly [ADP-ribose] polymerase 1	MOL000098	quercetin
ABCC9	P11511	ATP-binding cassette sub-family C member 9	MOL005043	campest-5-en-3beta-ol
HMGCR	Q8NAU1	3-hydroxy-3-methylglutaryl-coenzyme A reductase	MOL000953	CLR

CACNA1C	P18089	Voltage-dependent L-type calcium channel subunit alpha-1C	MOL002773	beta-carotene
FASLG	P05771	Protein kinase C beta type	MOL001439	arachidonic acid
GGT1	O75469	Nuclear receptor subfamily 1 group I member 2	MOL001439	arachidonic acid
KDR	P35968	Vascular endothelial growth factor receptor 2	MOL000098	quercetin
HMOX1	P09601	Heme oxygenase 1	MOL002773	beta-carotene
INS	P22459	Insulin [Cleaved into: Insulin B chain; Insulin A chain]	MOL001439	arachidonic acid
HTR1A	Q13573	5-hydroxytryptamine receptor 1B	MOL000098	quercetin
SERPINH1	P15172	Serpin H1	MOL001439	arachidonic acid
FMR1	Q5U5Q3	Synaptic functional regulator FMR1	MOL001439	arachidonic acid
MAOB	P27338	Amine oxidase [flavin-containing] B	MOL000098	quercetin
IL10	P22301	Interleukin-10	MOL000098	quercetin
MAPK1	P28482	Mitogen-activated protein kinase 1	MOL000098	quercetin
IGF2	P01344	Insulin-like growth factor II	MOL000098	quercetin
EGFR	P00533	Epidermal growth factor receptor	MOL000098	quercetin
SLC25A4	Q96EY1	ADP/ATP translocase 1	MOL005043	campest-5-en-3beta-ol
NQO1	Q7Z2W7	NAD	MOL000098	quercetin
NODAL	P48547	Nodal homolog	MOL001439	arachidonic acid
MYLK	Q15746	Myosin light chain kinase, smooth muscle	MOL000098	quercetin
SOD1	P00441	Superoxide dismutase [Cu-Zn]	MOL000098	quercetin
GPBAR1	P20309	G-protein coupled bile acid receptor 1	MOL000359	sitosterol
IL2	P60568	Interleukin-2	MOL000098	quercetin
ERBB2	P04626	Receptor tyrosine-protein kinase erbB-2	MOL000098	quercetin
CACNA1A	P10398	Voltage-dependent P/Q-type calcium channel subunit alpha-1A	MOL002773	beta-carotene
GRIN1	P27216	Glutamate receptor ionotropic, NMDA 1	MOL002773	beta-carotene
IFNG	P01579	Interferon gamma	MOL000098	quercetin
FOS	P01100	Proto-oncogene c-Fos	MOL000098	quercetin

SLC12A1	P62424	Solute carrier family 12 member 1	MOL001439	arachidonic acid
FGF2	O76054	Fibroblast growth factor 2	MOL001439	arachidonic acid
GABRD	Q9UP95	Gamma-aminobutyric acid receptor subunit delta	MOL000098	quercetin
ICAM1	P05362	Intercellular adhesion molecule 1	MOL000098	quercetin
CAV1	Q03135	Caveolin-1	MOL002773	beta-carotene
ABL1	Q9BTZ2	Tyrosine-protein kinase ABL2	MOL005043	campest-5-en-3beta-ol
SLC6A8	P11142	Sodium- and chloride-dependent creatine transporter 1	MOL001439	arachidonic acid
CACNA1S	P47895	Voltage-dependent L-type calcium channel subunit alpha-1S	MOL002773	beta-carotene
TPO	P02652	Thyroid peroxidase	MOL002773	beta-carotene
DRD4	P31645	D	MOL000098	quercetin
RB1	P06400	Retinoblastoma-associated protein	MOL000098	quercetin
CFTR	P11021	Cystic fibrosis transmembrane conductance regulator	MOL001439	arachidonic acid
SRC	P12931	Tyrosine-protein kinase SRC	MOL000098	quercetin
PGR	P06401	Progesterone receptor	MOL000359	sitosterol
THRA	P28472	Thyroid hormone receptor alpha	MOL000953	CLR
FECH	P59796	Ferrochelatase, mitochondrial	MOL000953	CLR
TCF3	Q9GZZ6	Transcription factor E2-alpha	MOL000359	sitosterol
IYD	P10275	Androgen receptor	MOL002773	beta-carotene
ACVR1B	P08684	Cytochrome P450 3A4	MOL005043	campest-5-en-3beta-ol
TP53	P04637	Cellular tumor antigen p53	MOL000471	aloe-emodin
CASP9	P55211	Caspase-9	MOL002773	beta-carotene
CDKN1A	P38936	Cyclin-dependent kinase inhibitor 1	MOL000471	aloe-emodin
CAT	Q00975	Catalase	MOL002773	beta-carotene
JAK3	P06401	Progesterone receptor	MOL001439	arachidonic acid
AKT1	P31749	RAC-alpha serine/threonine-protein kinase	MOL002773	beta-carotene
PIK3R1	P04150	Glutathione synthetase	MOL001439	arachidonic acid

NFKB1	Q05901	Nuclear factor NF-kappa-B p105 subunit	MOL000359	sitosterol
IL1B	P01584	Interleukin-1 beta	MOL000471	aloe-emodin
CHRM3	Q9UDX4	Muscarinic acetylcholine receptor M3	MOL000471	aloe-emodin
F12	Q9NS86	Coagulation factor XII	MOL001439	arachidonic acid
NFKBIA	P25963	NF-kappa-B inhibitor alpha	MOL000098	quercetin
SPARC	P36896	SPARC	MOL002773	beta-carotene
IGFBP3	P17936	Insulin-like growth factor-binding protein 3	MOL000098	quercetin
PTGS1	P23219	Prostaglandin G/H synthase 1	MOL001439	arachidonic acid
HTR2A	Q6NUS8	5-hydroxytryptamine receptor 2A	MOL000098	quercetin
SCN10A	Q06432	Sodium channel protein type 10 subunit alpha	MOL002773	beta-carotene
JUN	P05412	Transcription factor AP-1	MOL002773	beta-carotene
SNAI2	P30532	Zinc finger protein SNAI2	MOL000359	sitosterol
GATM	P35348	Glycine amidinotransferase, mitochondrial	MOL002773	beta-carotene
GABRA3	P04637	Cellular tumor antigen p53	MOL000098	quercetin
NOTCH1	Q96RP8	Neurogenic locus notch homolog protein 1	MOL001439	arachidonic acid
BAX	Q07812	Apoptosis regulator BAX	MOL000471	aloe-emodin
CYP24A1	Q96Q40	1,25-dihydroxyvitamin D	MOL000359	sitosterol
CYP3A4	P08684	Cytochrome P450 3A4	MOL002773	beta-carotene
SCN5A	Q14524	Sodium channel protein type 5 subunit alpha	MOL000098	quercetin
PAH	P12271	Phenylalanine-4-hydroxylase	MOL001439	arachidonic acid
AVP	P16389	Vasopressin-neurophysin 2-copeptin	MOL001439	arachidonic acid
MMP9	P14780	Matrix metalloproteinase-9	MOL000098	quercetin
