# Supplementary material for: Exploring Molecular Mechanisms of Aloe barbadmsis Miller on Diphenoxylate-Induced Constipation in Mice
Source: Evid Based Complement Alternat Med. 2022 May 6;2022:6225758. doi: 10.1155/2022/6225758 (PMC9106447; doi:10.1155/2022/6225758)
Supplement: Supplementary Materials — Table S1. Active ingredients of Aloe. Table S2. Potential targets related to active ingredients. Table S3. potential targets related to constipation. Table S4. Common targets related to active ingredients. Table S5. Table S5-1. Detailed information of BP enrichment of PPI network cluster 1 targets; Table S5-2. Detailed information of CC enrichment of PPI network cluster 1 targets; Table S5-3. Detailed information of MF enrichment of PPI network cluster 1 targets; Table S5-4. Detailed information of KEGG pathways enrichment of PPI network cluster 1 targets. Table S6. Table S6-1. Detailed information of BP enrichment of common targets; Table S6-2. Detailed information of CC enrichment of common targets; Table S6-3. Detailed information of MF enrichment of common targets; Table S6-4. Detailed information of KEGG pathways enrichment of common targets. Table S7. Original images of H&E staining in colon of three repeats in each group. Table S8. Raw data of 5-HT, SP, and VIP in serum and colon determined by ELISA kits. Table S9. Raw data of NF-κB p65, AKT, ERK, and JNK in colon determined by RT-PCR method. Table S10. Original images of ERK, JNK, AKT, and NF-κB p65 in colon of Western Blot, and its raw data quantification. [file 6225758.f1.zip › suppl table 1-10/Table S5 (1) (1).pdf]

Supplementary Table S5-1: Datailed information of biological process(BP)enrichment of PPI network cluster 1 targets

ID	Description	GeneRatio	BgRatio	pvalue	p.adjust	qvalue	geneID
GO:0097191	extrinsic apoptotic signaling pathway	14/32	224/18670	3.31E-19	1.06E-15	2.90E-16	AKT1/FASLG/BCL2/CASP8/CAV1/GSK3B/ICAM1/IGF1/IL1A/IL1B/IL2/SRC/TGFB1/TNF
GO:0032496	response to lipopolysaccharide	15/32	330/18670	1.61E-18	2.59E-15	7.04E-16	ABL1/AKT1/FASLG/CASP8/ICAM1/IL1B/IL10/IL13/JUN/MAPK1/PTGS2/SNCA/SRC/TGFB1/TNF
GO:0002237	response to molecule of bacterial origin	15/32	343/18670	2.88E-18	3.08E-15	8.39E-16	ABL1/AKT1/FASLG/CASP8/ICAM1/IL1B/IL10/IL13/JUN/MAPK1/PTGS2/SNCA/SRC/TGFB1/TNF
GO:1904951	positive regulation of establishment of protein localization	16/32	456/18670	5.19E-18	4.16E-15	1.13E-15	ABL1/BCL2/CASP8/ERBB2/GSK3B/IGF1/IL1A/IL1B/IL2/IL10/IL13/MAPK1/PTGS2/SRC/TGFB1/TNF
GO:0042110	T cell activation	16/32	464/18670	6.84E-18	4.39E-15	1.20E-15	ABL1/AKT1/BCL2/CASP8/CAV1/CTNNB1/ERBB2/ICAM1/IGF1/IL1B/IL2/IL10/IRF1/JAK3/SRC/TGFB1
GO:0045785	positive regulation of cell adhesion	15/32	403/18670	3.22E-17	1.49E-14	4.07E-15	ABL1/AKT1/CAV1/ERBB2/GSK3B/ICAM1/IGF1/IL1B/IL2/IL10/JAK3/KDR/SRC/TGFB1/TNF
GO:2001233	regulation of apoptotic signaling pathway	15/32	406/18670	3.59E-17	1.49E-14	4.07E-15	PARP1/AKT1/FASLG/BCL2/CASP8/CAV1/CTNNB1/GSK3B/ICAM1/IGF1/IL1A/IL1B/PTGS2/SRC/TNF
GO:1901214	regulation of neuron death	14/32	313/18670	3.73E-17	1.49E-14	4.07E-15	ABL1/PARP1/AKT1/FASLG/BCL2/CASP8/CTNNB1/GSK3B/IL10/IL13/JUN/MAPT/SNCA/TNF
GO:0070997	neuron death	14/32	348/18670	1.64E-16	5.84E-14	1.59E-14	ABL1/PARP1/AKT1/FASLG/BCL2/CASP8/CTNNB1/GSK3B/IL10/IL13/JUN/MAPT/SNCA/TNF
GO:2000377	regulation of reactive oxygen species metabolic process	12/32	195/18670	2.25E-16	7.23E-14	1.97E-14	AKT1/BCL2/CAV1/HSP90AA1/ICAM1/IL1B/IL10/MAPT/PTGS2/SNCA/TGFB1/TNF
GO:1901216	positive regulation of neuron death	10/32	94/18670	3.77E-16	1.10E-13	2.99E-14	ABL1/PARP1/FASLG/CASP8/CTNNB1/GSK3B/JUN/MAPT/SNCA/TNF
GO:0051249	regulation of lymphocyte activation	15/32	485/18670	5.04E-16	1.35E-13	3.67E-14	ABL1/AKT1/BCL2/CAV1/CTNNB1/ERBB2/IGF1/IL1B/IL2/IL10/IL13/IRF1/JAK3/SRC/TGFB1

GO:2001236	regulation of extrinsic apoptotic signaling pathway	11/32	155/18670	1.00E-15	2.46E-13	6.71E-14	AKT1/FASLG/BCL2/CASP8/CAV1/ICAM1/IGF1/IL1A/IL1B/SRC/TNF
GO:2001237	negative regulation of extrinsic apoptotic signaling pathway	10/32	104/18670	1.08E-15	2.46E-13	6.71E-14	AKT1/FASLG/BCL2/CASP8/ICAM1/IGF1/IL1A/IL1B/SRC/TNF
GO:1903037	regulation of leukocyte cell-cell adhesion	13/32	304/18670	1.15E-15	2.46E-13	6.71E-14	AKT1/CAV1/ERBB2/ICAM1/IGF1/IL1B/IL2/IL10/IRF1/JAK3/SRC/TGFB1/TNF
GO:0022407	regulation of cell-cell adhesion	14/32	403/18670	1.26E-15	2.53E-13	6.89E-14	ABL1/AKT1/CAV1/ERBB2/ICAM1/IGF1/IL1B/IL2/IL10/IRF1/JAK3/SRC/TGFB1/TNF
GO:2001234	negative regulation of apoptotic signaling pathway	12/32	230/18670	1.66E-15	3.12E-13	8.51E-14	AKT1/FASLG/BCL2/CASP8/CTNNB1/ICAM1/IGF1/IL1A/IL1B/PTGS2/SRC/TNF
GO:0050863	regulation of T cell activation	13/32	314/18670	1.75E-15	3.12E-13	8.51E-14	ABL1/AKT1/CAV1/CTNNB1/ERBB2/IGF1/IL1B/IL2/IL10/IRF1/JAK3/SRC/TGFB1
GO:0070371	ERK1 and ERK2 cascade	13/32	317/18670	1.98E-15	3.35E-13	9.12E-14	ABL1/ERBB2/FGF2/ICAM1/IGF1/IL1B/JUN/KDR/MYC/MAPK1/SRC/TGFB1/TNF
GO:0051222	positive regulation of protein transport	14/32	418/18670	2.09E-15	3.36E-13	9.14E-14	ABL1/ERBB2/GSK3B/IGF1/IL1A/IL1B/IL2/IL10/IL13/MAPK1/PTGS2/SRC/TGFB1/TNF
GO:1903829	positive regulation of cellular protein localization	13/32	324/18670	2.63E-15	4.02E-13	1.09E-13	PARP1/AKT1/BCL2/CASP8/ERBB2/GSK3B/IL1B/MAPT/MAPK1/PTGS2/SRC/TGFB1/TNF
GO:0050673	epithelial cell proliferation	14/32	434/18670	3.52E-15	5.13E-13	1.40E-13	AKT1/CCND1/CAV1/CTNNB1/ERBB2/FGF2/IGF1/IL10/JUN/KDR/MYC/MAPK1/TGFB1/TNF
GO:0007159	leukocyte cell-cell adhesion	13/32	337/18670	4.38E-15	6.10E-13	1.66E-13	AKT1/CAV1/ERBB2/ICAM1/IGF1/IL1B/IL2/IL10/IRF1/JAK3/SRC/TGFB1/TNF
GO:0010038	response to metal ion	13/32	364/18670	1.18E-14	1.58E-12	4.30E-13	PARP1/AKT1/CCND1/BCL2/CASP8/CAV1/ICAM1/IL1A/JUN/MAPT/MAPK1/PTGS2/SNCA
GO:0046651	lymphocyte proliferation	12/32	272/18670	1.25E-14	1.60E-12	4.37E-13	ABL1/BCL2/CTNNB1/ERBB2/IGF1/IL1B/IL2/IL10/IL13/IRF1/JAK3/TGFB1

GO:0032943	mononuclear cell proliferation	12/32	274/18670	1.36E-14	1.68E-12	4.59E-13	ABL1/BCL2/CTNNB1/ERBB2/IGF1/IL1B/IL2/IL10/IL13/IRF1/JAK3/TGFB1
GO:0050678	regulation of epithelial cell proliferation	13/32	378/18670	1.92E-14	2.28E-12	6.21E-13	AKT1/CCND1/CAV1/CTNNB1/ERBB2/FGF2/IGF1/IL10/JUN/KDR/MYC/TGFB1/TNF
GO:0072593	reactive oxygen species metabolic process	12/32	284/18670	2.10E-14	2.40E-12	6.54E-13	AKT1/BCL2/CAV1/HSP90AA1/ICAM1/IL1B/IL10/MAPT/PTGS2/SNCA/TGFB1/TNF
GO:0050679	positive regulation of epithelial cell proliferation	11/32	206/18670	2.38E-14	2.63E-12	7.16E-13	AKT1/CCND1/CTNNB1/ERBB2/FGF2/IGF1/IL10/JUN/KDR/MYC/TGFB1
GO:0050670	regulation of lymphocyte proliferation	11/32	208/18670	2.65E-14	2.83E-12	7.71E-13	BCL2/CTNNB1/ERBB2/IGF1/IL1B/IL2/IL10/IL13/IRF1/JAK3/TGFB1
GO:0032944	regulation of mononuclear cell proliferation	11/32	209/18670	2.79E-14	2.89E-12	7.86E-13	BCL2/CTNNB1/ERBB2/IGF1/IL1B/IL2/IL10/IL13/IRF1/JAK3/TGFB1
GO:0070661	leukocyte proliferation	12/32	298/18670	3.72E-14	3.73E-12	1.02E-12	ABL1/BCL2/CTNNB1/ERBB2/IGF1/IL1B/IL2/IL10/IL13/IRF1/JAK3/TGFB1
GO:0034599	cellular response to oxidative stress	12/32	302/18670	4.36E-14	4.24E-12	1.16E-12	ABL1/PARP1/AKT1/BCL2/CTNNB1/IL10/JUN/MAPT/MAPK1/SNCA/SRC/TNF
GO:0070663	regulation of leukocyte proliferation	11/32	222/18670	5.43E-14	5.12E-12	1.40E-12	BCL2/CTNNB1/ERBB2/IGF1/IL1B/IL2/IL10/IL13/IRF1/JAK3/TGFB1
GO:1903426	regulation of reactive oxygen species biosynthetic process	9/32	101/18670	6.98E-14	6.39E-12	1.74E-12	AKT1/CAV1/HSP90AA1/ICAM1/IL1B/IL10/PTGS2/SNCA/TNF
GO:0051054	positive regulation of DNA metabolic process	11/32	228/18670	7.29E-14	6.49E-12	1.77E-12	PARP1/AKT1/CTNNB1/FGF2/HSP90AA1/IL2/JUN/MYC/MAPK1/SRC/TGFB1
GO:2000379	positive regulation of reactive oxygen species metabolic process	9/32	102/18670	7.64E-14	6.62E-12	1.80E-12	AKT1/HSP90AA1/ICAM1/IL1B/MAPT/PTGS2/SNCA/TGFB1/TNF
GO:0018209	peptidyl-serine modification	12/32	322/18670	9.36E-14	7.90E-12	2.15E-12	PARP1/AKT1/BCL2/CAV1/GSK3B/HSP90AA1/MAPK1/PTGS2/SNCA/SRC/TGFB1/TNF

GO:0045428	regulation of nitric oxide biosynthetic process	8/32	66/18670	1.55E-13	1.27E-11	3.46E-12	AKT1/CAV1/HSP90AA1/ICAM1/IL1B/IL10/PTGS2/TNF
GO:0006979	response to oxidative stress	13/32	451/18670	1.84E-13	1.47E-11	4.02E-12	ABL1/PARP1/AKT1/BCL2/CTNNB1/IL10/JUN/MAPT/MAPK1/PTGS2/SNCA/SRC/TNF
GO:0022409	positive regulation of cell-cell adhesion	11/32	255/18670	2.49E-13	1.95E-11	5.31E-12	AKT1/CAV1/ICAM1/IGF1/IL1B/IL2/IL10/JAK3/SRC/TGFB1/TNF
GO:0001819	positive regulation of cytokine production	13/32	464/18670	2.64E-13	2.02E-11	5.49E-12	ABL1/CASP8/CTNNB1/IL1A/IL1B/IL2/IL10/IL13/IRF1/PTGS2/SRC/TGFB1/TNF
GO:0042098	T cell proliferation	10/32	184/18670	3.61E-13	2.69E-11	7.34E-12	ABL1/CTNNB1/ERBB2/IGF1/IL1B/IL2/IL10/IRF1/JAK3/TGFB1
GO:0018108	peptidyl-tyrosine phosphorylation	12/32	363/18670	3.88E-13	2.83E-11	7.70E-12	ABL1/CAV1/ERBB2/ICAM1/IGF1/IL2/IL13/JAK3/KDR/SRC/TGFB1/TNF
GO:1903409	reactive oxygen species biosynthetic process	9/32	122/18670	3.98E-13	2.84E-11	7.73E-12	AKT1/CAV1/HSP90AA1/ICAM1/IL1B/IL10/PTGS2/SNCA/TNF
GO:0018212	peptidyl-tyrosine modification	12/32	366/18670	4.27E-13	2.98E-11	8.12E-12	ABL1/CAV1/ERBB2/ICAM1/IGF1/IL2/IL13/JAK3/KDR/SRC/TGFB1/TNF
GO:0150076	neuroinflammatory response	8/32	75/18670	4.50E-13	3.07E-11	8.36E-12	IGF1/IL1B/IL13/JUN/MAPT/PTGS2/SNCA/TNF
GO:0035690	cellular response to drug	12/32	369/18670	4.71E-13	3.14E-11	8.57E-12	ABL1/CTNNB1/ICAM1/IL1B/IL10/KDR/MYC/MAPK1/PTGS2/SRC/TGFB1/TNF
GO:1902105	regulation of leukocyte differentiation	11/32	272/18670	5.05E-13	3.30E-11	9.00E-12	ABL1/CASP8/CTNNB1/ERBB2/IL2/IRF1/JAK3/JUN/MYC/TGFB1/TNF
GO:0051098	regulation of binding	12/32	373/18670	5.34E-13	3.43E-11	9.34E-12	ABL1/PARP1/AKT1/BCL2/CAV1/CTNNB1/GSK3B/IGF1/IL10/JUN/SRC/TGFB1
GO:0006809	nitric oxide biosynthetic process	8/32	77/18670	5.59E-13	3.52E-11	9.58E-12	AKT1/CAV1/HSP90AA1/ICAM1/IL1B/IL10/PTGS2/TNF

GO:0046209	nitric oxide metabolic process	8/32	82/18670	9.42E-13	5.81E-11	1.58E-11	AKT1/CAV1/HSP90AA1/ICAM1/IL1B/IL10/PTGS2/TNF
GO:2001057	reactive nitrogen species metabolic process	8/32	85/18670	1.27E-12	7.67E-11	2.09E-11	AKT1/CAV1/HSP90AA1/ICAM1/IL1B/IL10/PTGS2/TNF
GO:0018105	peptidyl-serine phosphorylation	11/32	299/18670	1.42E-12	8.41E-11	2.29E-11	AKT1/BCL2/CAV1/GSK3B/HSP90AA1/MAPK1/PTGS2/SNCA/SRC/TGFB1/TNF
GO:1903039	positive regulation of leukocyte cell-cell adhesion	10/32	218/18670	1.97E-12	1.15E-10	3.14E-11	AKT1/CAV1/ICAM1/IGF1/IL1B/IL2/JAK3/SRC/TGFB1/TNF
GO:0051052	regulation of DNA metabolic process	12/32	429/18670	2.77E-12	1.59E-10	4.32E-11	PARP1/AKT1/CTNNB1/FGF2/HSP90AA1/IL2/IL10/JUN/MYC/MAPK1/SRC/TGFB1
GO:0048732	gland development	12/32	434/18670	3.17E-12	1.79E-10	4.87E-11	ABL1/AKT1/CCND1/BCL2/CAV1/CTNNB1/IL10/JUN/MAPK1/SRC/TGFB1/TNF
GO:0046677	response to antibiotic	11/32	327/18670	3.74E-12	2.03E-10	5.55E-11	ABL1/CCND1/BCL2/CASP8/HSP90AA1/ICAM1/IL2/IL10/IL13/JUN/SRC
GO:0042129	regulation of T cell proliferation	9/32	156/18670	3.74E-12	2.03E-10	5.55E-11	CTNNB1/ERBB2/IGF1/IL1B/IL2/IL10/IRF1/JAK3/TGFB1
GO:0051235	maintenance of location	11/32	330/18670	4.13E-12	2.21E-10	6.01E-11	ABL1/AKT1/FASLG/CAV1/FGF2/IL1B/IL10/IL13/SNCA/TGFB1/TNF
GO:0071216	cellular response to biotic stimulus	10/32	236/18670	4.35E-12	2.29E-10	6.23E-11	ABL1/AKT1/GSK3B/ICAM1/IL1B/IL10/MAPK1/SRC/TGFB1/TNF
GO:0071902	positive regulation of protein serine/threonine kinase activity	11/32	334/18670	4.70E-12	2.43E-10	6.63E-11	AKT1/CCND1/ERBB2/FGF2/IGF1/IL1B/MAPK1/SNCA/SRC/TGFB1/TNF
GO:0033002	muscle cell proliferation	10/32	239/18670	4.93E-12	2.51E-10	6.84E-11	AKT1/CTNNB1/FGF2/IGF1/IL10/IL13/JUN/MAPK1/PTGS2/TNF
GO:0048661	positive regulation of smooth muscle cell proliferation	8/32	101/18670	5.23E-12	2.60E-10	7.08E-11	AKT1/FGF2/IGF1/IL10/IL13/JUN/PTGS2/TNF

GO:0031960	response to corticosteroid	9/32	162/18670	5.27E-12	2.60E-10	7.08E-11	PARP1/CCND1/BCL2/ICAM1/IL10/PTGS2/SRC/TGFB1/TNF
GO:0071496	cellular response to external stimulus	11/32	339/18670	5.52E-12	2.68E-10	7.31E-11	AKT1/BCL2/CASP8/ICAM1/IL1B/IL13/IRF1/JUN/MAPK1/PTGS2/TGFB1
GO:0033138	positive regulation of peptidyl-serine phosphorylation	8/32	105/18670	7.18E-12	3.43E-10	9.36E-11	AKT1/BCL2/CAV1/HSP90AA1/PTGS2/SNCA/TGFB1/TNF
GO:0043254	regulation of protein complex assembly	12/32	467/18670	7.48E-12	3.53E-10	9.61E-11	ABL1/PARP1/CTNNB1/GSK3B/HSP90AA1/ICAM1/JUN/MAPT/SNCA/SRC/TGFB1/TNF
GO:0048660	regulation of smooth muscle cell proliferation	9/32	169/18670	7.73E-12	3.55E-10	9.68E-11	AKT1/CTNNB1/FGF2/IGF1/IL10/IL13/JUN/PTGS2/TNF
GO:0042136	neurotransmitter biosynthetic process	8/32	106/18670	7.76E-12	3.55E-10	9.68E-11	AKT1/CAV1/HSP90AA1/ICAM1/IL1B/IL10/PTGS2/TNF
GO:0051341	regulation of oxidoreductase activity	8/32	107/18670	8.37E-12	3.78E-10	1.03E-10	ABL1/AKT1/CAV1/HSP90AA1/IL1B/IL13/SNCA/TNF
GO:0048659	smooth muscle cell proliferation	9/32	171/18670	8.59E-12	3.83E-10	1.04E-10	AKT1/CTNNB1/FGF2/IGF1/IL10/IL13/JUN/PTGS2/TNF
GO:0045862	positive regulation of proteolysis	11/32	363/18670	1.16E-11	5.08E-10	1.38E-10	AKT1/FASLG/CASP8/CAV1/GSK3B/IL1B/MAPT/MYC/SNCA/SRC/TNF
GO:0072577	endothelial cell apoptotic process	7/32	65/18670	1.40E-11	6.05E-10	1.65E-10	ABL1/FASLG/ICAM1/IL10/IL13/KDR/TNF
GO:0061138	morphogenesis of a branching epithelium	9/32	182/18670	1.51E-11	6.39E-10	1.74E-10	ABL1/BCL2/CTNNB1/FGF2/IL10/MYC/SRC/TGFB1/TNF
GO:0031334	positive regulation of protein complex assembly	10/32	268/18670	1.53E-11	6.39E-10	1.74E-10	PARP1/CTNNB1/GSK3B/HSP90AA1/ICAM1/JUN/MAPT/SRC/TGFB1/TNF
GO:0050714	positive regulation of protein secretion	10/32	268/18670	1.53E-11	6.39E-10	1.74E-10	ABL1/IGF1/IL1A/IL1B/IL2/IL10/IL13/SRC/TGFB1/TNF

GO:0043491	protein kinase B signaling	10/32	269/18670	1.59E-11	6.54E-10	1.78E-10	AKT1/ERBB2/FGF2/HSP90AA1/IGF1/IL1B/KDR/SRC/TGFB1/TNF
GO:0002696	positive regulation of leukocyte activation	11/32	380/18670	1.89E-11	7.68E-10	2.09E-10	AKT1/BCL2/CAV1/IGF1/IL1B/IL2/IL10/IL13/JAK3/SRC/TGFB1
GO:0048545	response to steroid hormone	11/32	385/18670	2.18E-11	8.72E-10	2.38E-10	PARP1/CCND1/BCL2/CAV1/CTNNB1/ICAM1/IL10/PTGS2/SRC/TGFB1/TNF
GO:0050867	positive regulation of cell activation	11/32	394/18670	2.79E-11	1.10E-09	3.01E-10	AKT1/BCL2/CAV1/IGF1/IL1B/IL2/IL10/IL13/JAK3/SRC/TGFB1
GO:0038034	signal transduction in absence of ligand	7/32	72/18670	2.93E-11	1.11E-09	3.02E-10	AKT1/BCL2/GSK3B/IL1A/IL1B/IL2/TNF
GO:0051881	regulation of mitochondrial membrane potential	7/32	72/18670	2.93E-11	1.11E-09	3.02E-10	ABL1/PARP1/AKT1/BCL2/KDR/MAPT/SRC
GO:0097192	extrinsic apoptotic signaling pathway in absence of ligand	7/32	72/18670	2.93E-11	1.11E-09	3.02E-10	AKT1/BCL2/GSK3B/IL1A/IL1B/IL2/TNF
GO:0001763	morphogenesis of a branching structure	9/32	196/18670	2.93E-11	1.11E-09	3.02E-10	ABL1/BCL2/CTNNB1/FGF2/IL10/MYC/SRC/TGFB1/TNF
GO:0002793	positive regulation of peptide secretion	10/32	288/18670	3.12E-11	1.16E-09	3.17E-10	ABL1/IGF1/IL1A/IL1B/IL2/IL10/IL13/SRC/TGFB1/TNF
GO:1903532	positive regulation of secretion by cell	11/32	399/18670	3.19E-11	1.18E-09	3.21E-10	ABL1/IGF1/IL1A/IL1B/IL2/IL10/IL13/SNCA/SRC/TGFB1/TNF
GO:0071222	cellular response to lipopolysaccharide	9/32	205/18670	4.39E-11	1.60E-09	4.35E-10	ABL1/AKT1/ICAM1/IL1B/IL10/MAPK1/SRC/TGFB1/TNF
GO:0070372	regulation of ERK1 and ERK2 cascade	10/32	300/18670	4.66E-11	1.68E-09	4.58E-10	ABL1/ERBB2/FGF2/ICAM1/IL1B/JUN/KDR/SRC/TGFB1/TNF
GO:0009612	response to mechanical stimulus	9/32	210/18670	5.44E-11	1.94E-09	5.28E-10	AKT1/CASP8/IL1B/IL13/IRF1/JUN/PTGS2/SRC/TGFB1

GO:0071260	cellular response to mechanical stimulus	7/32	79/18670	5.72E-11	2.02E-09	5.49E-10	AKT1/CASP8/IL1B/IL13/IRF1/PTGS2/TGFB1
GO:0071219	cellular response to molecule of bacterial origin	9/32	212/18670	5.92E-11	2.06E-09	5.62E-10	ABL1/AKT1/ICAM1/IL1B/IL10/MAPK1/SRC/TGFB1/TNF
GO:0043281	regulation of cysteine-type endopeptidase activity involved in apoptotic process	9/32	215/18670	6.71E-11	2.31E-09	6.29E-10	AKT1/FASLG/CASP8/MAPT/MYC/PTGS2/SNCA/SRC/TNF
GO:0051047	positive regulation of secretion	11/32	428/18670	6.77E-11	2.31E-09	6.29E-10	ABL1/IGF1/IL1A/IL1B/IL2/IL10/IL13/SNCA/SRC/TGFB1/TNF
GO:0033135	regulation of peptidyl-serine phosphorylation	8/32	139/18670	6.97E-11	2.35E-09	6.41E-10	AKT1/BCL2/CAV1/HSP90AA1/PTGS2/SNCA/TGFB1/TNF
GO:0010001	glial cell differentiation	9/32	218/18670	7.60E-11	2.54E-09	6.92E-10	ABL1/AKT1/CTNNB1/ERBB2/IL1B/MAPT/MAPK1/TGFB1/TNF
GO:0006816	calcium ion transport	11/32	434/18670	7.85E-11	2.57E-09	7.00E-10	ABL1/FASLG/BCL2/CAV1/CTNNB1/FGF2/ICAM1/IL13/PTGS2/SNCA/TGFB1
GO:0042391	regulation of membrane potential	11/32	434/18670	7.85E-11	2.57E-09	7.00E-10	ABL1/PARP1/AKT1/BCL2/CAV1/GSK3B/JUN/KDR/MAPT/SNCA/SRC
GO:0043434	response to peptide hormone	11/32	436/18670	8.25E-11	2.65E-09	7.23E-10	PARP1/AKT1/CAV1/GSK3B/ICAM1/IL1B/IL10/JAK3/PTGS2/SRC/TGFB1
GO:0038127	ERBB signaling pathway	8/32	142/18670	8.28E-11	2.65E-09	7.23E-10	ABL1/AKT1/FASLG/ERBB2/HSP90AA1/MAPK1/SRC/TGFB1
GO:0045429	positive regulation of nitric oxide biosynthetic process	6/32	43/18670	8.99E-11	2.85E-09	7.76E-10	AKT1/HSP90AA1/ICAM1/IL1B/PTGS2/TNF
GO:0007568	aging	10/32	321/18670	9.06E-11	2.85E-09	7.76E-10	ABL1/AKT1/BCL2/ICAM1/IL10/JUN/MAPK1/PTGS2/SNCA/TGFB1
GO:1904407	positive regulation of nitric oxide metabolic process	6/32	44/18670	1.04E-10	3.24E-09	8.82E-10	AKT1/HSP90AA1/ICAM1/IL1B/PTGS2/TNF

GO:0048754	branching morphogenesis of an epithelial tube	8/32	150/18670	1.29E-10	3.96E-09	1.08E-09	ABL1/BCL2/CTNNB1/FGF2/MYC/SRC/TGFB1/TNF
GO:0000302	response to reactive oxygen species	9/32	232/18670	1.32E-10	4.04E-09	1.10E-09	ABL1/AKT1/BCL2/IL10/JUN/MAPT/MAPK1/SRC/TNF
GO:0051251	positive regulation of lymphocyte activation	10/32	334/18670	1.34E-10	4.04E-09	1.10E-09	AKT1/BCL2/CAV1/IGF1/IL1B/IL2/IL13/JAK3/SRC/TGFB1
GO:0097305	response to alcohol	9/32	233/18670	1.37E-10	4.12E-09	1.12E-09	PARP1/AKT1/CCND1/CASP8/CTNNB1/ICAM1/IL2/IL13/TGFB1
GO:0062012	regulation of small molecule metabolic process	11/32	459/18670	1.43E-10	4.23E-09	1.15E-09	PARP1/AKT1/CAV1/GSK3B/IGF1/IL1B/PTGS2/SNCA/SRC/TGFB1/TNF
GO:0042133	neurotransmitter metabolic process	8/32	153/18670	1.51E-10	4.43E-09	1.21E-09	AKT1/CAV1/HSP90AA1/ICAM1/IL1B/IL10/PTGS2/TNF
GO:0051899	membrane depolarization	7/32	91/18670	1.58E-10	4.59E-09	1.25E-09	ABL1/PARP1/BCL2/CAV1/JUN/KDR/SRC
GO:2000116	regulation of cysteine-type endopeptidase activity	9/32	239/18670	1.72E-10	4.98E-09	1.36E-09	AKT1/FASLG/CASP8/MAPT/MYC/PTGS2/SNCA/SRC/TNF
GO:0060402	calcium ion transport into cytosol	8/32	158/18670	1.95E-10	5.58E-09	1.52E-09	ABL1/FASLG/BCL2/CAV1/FGF2/IL13/SNCA/TGFB1
GO:0009266	response to temperature stimulus	9/32	243/18670	2.00E-10	5.67E-09	1.54E-09	AKT1/CASP8/GSK3B/HSP90AA1/IGF1/IL1A/MAPT/MAPK1/PTGS2
GO:1903706	regulation of hemopoiesis	11/32	475/18670	2.05E-10	5.77E-09	1.57E-09	ABL1/CASP8/CTNNB1/ERBB2/IL2/IRF1/JAK3/JUN/MYC/TGFB1/TNF
GO:0001505	regulation of neurotransmitter levels	10/32	354/18670	2.36E-10	6.57E-09	1.79E-09	AKT1/CAV1/GSK3B/HSP90AA1/ICAM1/IL1B/IL10/PTGS2/SNCA/TNF
GO:0070838	divalent metal ion transport	11/32	483/18670	2.45E-10	6.77E-09	1.85E-09	ABL1/FASLG/BCL2/CAV1/CTNNB1/FGF2/ICAM1/IL13/PTGS2/SNCA/TGFB1

GO:0016049	cell growth	11/32	484/18670	2.50E-10	6.86E-09	1.87E-09	ABL1/AKT1/BCL2/CTNNB1/ERBB2/GSK3B/HSP90AA1/IGF1/IL2/MAPT/TGFB1	
GO:0072511	divalent inorganic cation transport	11/32	489/18670	2.79E-10	7.59E-09	2.07E-09	ABL1/FASLG/BCL2/CAV1/CTNNB1/FGF2/ICAM1/IL13/PTGS2/SNCA/TGFB1	
GO:0051924	regulation of calcium ion transport	9/32	254/18670	2.96E-10	7.97E-09	2.17E-09	ABL1/BCL2/CAV1/CTNNB1/ICAM1/IL13/PTGS2/SNCA/TGFB1	
GO:0050730	regulation of peptidyl-tyrosine phosphorylation	9/32	256/18670	3.17E-10	8.43E-09	2.30E-09	ABL1/CAV1/ICAM1/IGF1/IL2/IL13/SRC/TGFB1/TNF	
GO:0034614	cellular response to reactive oxygen species	8/32	168/18670	3.18E-10	8.43E-09	2.30E-09	ABL1/AKT1/IL10/JUN/MAPT/MAPK1/SRC/TNF	
GO:0051882	mitochondrial depolarization	5/32	23/18670	3.51E-10	9.22E-09	2.51E-09	ABL1/PARP1/BCL2/KDR/SRC	
GO:0010634	positive regulation of epithelial cell migration	8/32	171/18670	3.66E-10	9.48E-09	2.58E-09	ABL1/AKT1/FGF2/JUN/KDR/PTGS2/SRC/TGFB1	
GO:0060401	cytosolic calcium ion transport	8/32	171/18670	3.66E-10	9.48E-09	2.58E-09	ABL1/FASLG/BCL2/CAV1/FGF2/IL13/SNCA/TGFB1	
GO:0009408	response to heat	8/32	176/18670	4.61E-10	1.18E-08	3.22E-09	AKT1/GSK3B/HSP90AA1/IGF1/IL1A/MAPT/MAPK1/PTGS2	
GO:0045765	regulation of angiogenesis	10/32	383/18670	5.07E-10	1.29E-08	3.51E-09	ABL1/FASLG/CTNNB1/ERBB2/FGF2/IL1A/IL1B/IL10/KDR/PTGS2	
GO:1903428	positive regulation of reactive oxygen species biosynthetic process	6/32	57/18670	5.26E-10	1.33E-08	3.62E-09	AKT1/HSP90AA1/ICAM1/IL1B/PTGS2/TNF	
GO:1901653	cellular response to peptide	10/32	385/18670	5.33E-10	1.33E-08	3.64E-09	PARP1/AKT1/CAV1/GSK3B/ICAM1/IGF1/IL1B/JAK3/SRC/TGFB1	
GO:0022408	negative regulation of cell-cell adhesion	8/32	181/18670	5.76E-10	1.43E-08	3.90E-09	ABL1/AKT1/ERBB2/IL2/IL10/IRF1/JAK3/TGFB1	

GO:0061900	glial cell activation	6/32	58/18670	5.86E-10	1.45E-08	3.94E-09	IL1B/IL13/JUN/MAPT/SNCA/TNF
GO:1904019	epithelial cell apoptotic process	7/32	111/18670	6.46E-10	1.58E-08	4.31E-09	ABL1/FASLG/ICAM1/IL10/IL13/KDR/TNF
GO:2000351	regulation of endothelial cell apoptotic process	6/32	59/18670	6.52E-10	1.58E-08	4.32E-09	ABL1/FASLG/ICAM1/IL13/KDR/TNF
GO:0010959	regulation of metal ion transport	10/32	394/18670	6.67E-10	1.61E-08	4.38E-09	ABL1/AKT1/BCL2/CAV1/CTNNB1/ICAM1/IL13/PTGS2/SNCA/TGFB1
GO:1900180	regulation of protein localization to nucleus	7/32	116/18670	8.81E-10	2.11E-08	5.74E-09	PARP1/AKT1/GSK3B/MAPK1/PTGS2/SRC/TGFB1
GO:0002260	lymphocyte homeostasis	6/32	62/18670	8.86E-10	2.11E-08	5.74E-09	ABL1/AKT1/BCL2/IL2/JAK3/TGFB1
GO:0050731	positive regulation of peptidyl-tyrosine phosphorylation	8/32	192/18670	9.20E-10	2.17E-08	5.90E-09	ABL1/ICAM1/IGF1/IL2/IL13/SRC/TGFB1/TNF
GO:0007162	negative regulation of cell adhesion	9/32	289/18670	9.25E-10	2.17E-08	5.90E-09	ABL1/AKT1/ERBB2/IL2/IL10/IRF1/JAK3/SRC/TGFB1
GO:0042063	gliogenesis	9/32	290/18670	9.54E-10	2.22E-08	6.04E-09	ABL1/AKT1/CTNNB1/ERBB2/IL1B/MAPT/MAPK1/TGFB1/TNF
GO:0032768	regulation of monooxygenase activity	6/32	64/18670	1.08E-09	2.49E-08	6.78E-09	AKT1/CAV1/HSP90AA1/IL1B/SNCA/TNF
GO:1901342	regulation of vasculature development	10/32	422/18670	1.29E-09	2.97E-08	8.08E-09	ABL1/FASLG/CTNNB1/ERBB2/FGF2/IL1A/IL1B/IL10/KDR/PTGS2
GO:0050870	positive regulation of T cell activation	8/32	202/18670	1.38E-09	3.13E-08	8.53E-09	AKT1/CAV1/IGF1/IL1B/IL2/JAK3/SRC/TGFB1
GO:2000573	positive regulation of DNA biosynthetic process	6/32	67/18670	1.43E-09	3.23E-08	8.80E-09	CTNNB1/FGF2/HSP90AA1/MYC/MAPK1/SRC

GO:0001660	fever generation	4/32	10/18670	1.48E-09	3.31E-08	9.02E-09	IL1A/IL1B/PTGS2/TNF
GO:0002573	myeloid leukocyte differentiation	8/32	204/18670	1.49E-09	3.31E-08	9.02E-09	PARP1/CASP8/CTNNB1/JUN/MYC/SRC/TGFB1/TNF
GO:0048608	reproductive structure development	10/32	431/18670	1.59E-09	3.51E-08	9.56E-09	AKT1/CCND1/BCL2/CASP8/CTNNB1/ICAM1/IL10/MAPK1/PTGS2/SRC
GO:0051090	regulation of DNA-binding transcription factor activity	10/32	432/18670	1.62E-09	3.56E-08	9.71E-09	AKT1/CAV1/CTNNB1/ICAM1/IL1B/IL10/JUN/MAPK1/TGFB1/TNF
GO:0061458	reproductive system development	10/32	434/18670	1.70E-09	3.70E-08	1.01E-08	AKT1/CCND1/BCL2/CASP8/CTNNB1/ICAM1/IL10/MAPK1/PTGS2/SRC
GO:1901215	negative regulation of neuron death	8/32	208/18670	1.73E-09	3.76E-08	1.02E-08	AKT1/BCL2/CTNNB1/GSK3B/IL10/IL13/JUN/SNCA
GO:1903038	negative regulation of leukocyte cell-cell adhesion	7/32	129/18670	1.86E-09	4.00E-08	1.09E-08	AKT1/ERBB2/IL2/IL10/IRF1/JAK3/TGFB1
GO:0007204	positive regulation of cytosolic calcium ion concentration	9/32	319/18670	2.20E-09	4.71E-08	1.28E-08	ABL1/FASLG/BCL2/CAV1/FGF2/IL2/IL13/SNCA/TGFB1
GO:0070374	positive regulation of ERK1 and ERK2 cascade	8/32	215/18670	2.25E-09	4.78E-08	1.30E-08	ABL1/FGF2/ICAM1/JUN/KDR/SRC/TGFB1/TNF
GO:0052547	regulation of peptidase activity	10/32	452/18670	2.51E-09	5.29E-08	1.44E-08	AKT1/FASLG/CASP8/CAV1/MAPT/MYC/PTGS2/SNCA/SRC/TNF
GO:1900182	positive regulation of protein localization to nucleus	6/32	74/18670	2.63E-09	5.52E-08	1.50E-08	PARP1/AKT1/MAPK1/PTGS2/SRC/TGFB1
GO:0046425	regulation of JAK-STAT cascade	7/32	137/18670	2.84E-09	5.90E-08	1.61E-08	CAV1/IGF1/IL2/IL10/IL13/JAK3/TNF
GO:0071214	cellular response to abiotic stimulus	9/32	331/18670	3.04E-09	6.25E-08	1.70E-08	PARP1/AKT1/CASP8/IL1B/IL13/IRF1/MYC/PTGS2/TGFB1

GO:0104004	cellular response to environmental stimulus	9/32	331/18670	3.04E-09	6.25E-08	1.70E-08	PARP1/AKT1/CASP8/IL1B/IL13/IRF1/MYC/PTGS2/TGFB1
GO:0002700	regulation of production of molecular mediator of immune response	7/32	139/18670	3.14E-09	6.41E-08	1.75E-08	IL1B/IL2/IL10/IL13/JAK3/TGFB1/TNF
GO:0002683	negative regulation of immune system process	10/32	463/18670	3.16E-09	6.41E-08	1.75E-08	AKT1/CTNNB1/ERBB2/IL2/IL10/IRF1/JAK3/MYC/TGFB1/TNF
GO:0010623	programmed cell death involved in cell development	4/32	12/18670	3.48E-09	7.02E-08	1.91E-08	FASLG/BCL2/IL1A/IL1B
GO:0042326	negative regulation of phosphorylation	10/32	468/18670	3.50E-09	7.02E-08	1.91E-08	ABL1/AKT1/CAV1/IL1B/IL2/JUN/MAPT/MYC/SNCA/TGFB1
GO:0043405	regulation of MAP kinase activity	9/32	337/18670	3.56E-09	7.09E-08	1.93E-08	CAV1/ERBB2/FGF2/IGF1/IL1B/MAPK1/SRC/TGFB1/TNF
GO:0050708	regulation of protein secretion	10/32	472/18670	3.80E-09	7.52E-08	2.05E-08	ABL1/IGF1/IL1A/IL1B/IL2/IL10/IL13/SRC/TGFB1/TNF
GO:1901099	negative regulation of signal transduction in absence of ligand	5/32	36/18670	3.87E-09	7.57E-08	2.06E-08	AKT1/BCL2/IL1A/IL1B/TNF
GO:2001240	negative regulation of extrinsic apoptotic signaling pathway in absence of ligand	5/32	36/18670	3.87E-09	7.57E-08	2.06E-08	AKT1/BCL2/IL1A/IL1B/TNF
GO:0043536	positive regulation of blood vessel endothelial cell migration	6/32	79/18670	3.93E-09	7.63E-08	2.08E-08	ABL1/AKT1/FGF2/KDR/PTGS2/TGFB1
GO:0033044	regulation of chromosome organization	9/32	342/18670	4.05E-09	7.82E-08	2.13E-08	PARP1/CTNNB1/IL1B/MAPT/MYC/MAPK1/SNCA/SRC/TGFB1
GO:0045834	positive regulation of lipid metabolic process	7/32	146/18670	4.42E-09	8.39E-08	2.29E-08	AKT1/FGF2/IL1B/PTGS2/SRC/TGFB1/TNF
GO:0051384	response to glucocorticoid	7/32	146/18670	4.42E-09	8.39E-08	2.29E-08	CCND1/BCL2/ICAM1/IL10/PTGS2/TGFB1/TNF

GO:1904892	regulation of STAT cascade	7/32	146/18670	4.42E-09	8.39E-08	2.29E-08	CAV1/IGF1/IL2/IL10/IL13/JAK3/TNF
GO:0043029	T cell homeostasis	5/32	37/18670	4.47E-09	8.43E-08	2.30E-08	AKT1/BCL2/IL2/JAK3/TGFB1
GO:0046777	protein autophosphorylation	8/32	235/18670	4.53E-09	8.50E-08	2.32E-08	ABL1/AKT1/CAV1/ERBB2/GSK3B/JUN/KDR/SRC
GO:0014065	phosphatidylinositol 3-kinase signaling	7/32	148/18670	4.86E-09	9.06E-08	2.47E-08	AKT1/ERBB2/IGF1/KDR/MAPK1/SRC/TNF
GO:0048145	regulation of fibroblast proliferation	6/32	83/18670	5.31E-09	9.80E-08	2.67E-08	CTNNB1/IGF1/IL13/JUN/MYC/TGFB1
GO:0030098	lymphocyte differentiation	9/32	353/18670	5.33E-09	9.80E-08	2.67E-08	ABL1/BCL2/CTNNB1/ERBB2/IL2/IL10/IRF1/JAK3/TGFB1
GO:0030217	T cell differentiation	8/32	240/18670	5.35E-09	9.80E-08	2.67E-08	ABL1/BCL2/CTNNB1/ERBB2/IL2/IRF1/JAK3/TGFB1
GO:0048144	fibroblast proliferation	6/32	84/18670	5.71E-09	1.04E-07	2.83E-08	CTNNB1/IGF1/IL13/JUN/MYC/TGFB1
GO:0051480	regulation of cytosolic calcium ion concentration	9/32	357/18670	5.88E-09	1.07E-07	2.90E-08	ABL1/FASLG/BCL2/CAV1/FGF2/IL2/IL13/SNCA/TGFB1
GO:0006914	autophagy	10/32	496/18670	6.11E-09	1.09E-07	2.98E-08	ABL1/AKT1/BCL2/GSK3B/HSP90AA1/IL10/KDR/MAPT/SNCA/SRC
GO:0061919	process utilizing autophagic mechanism	10/32	496/18670	6.11E-09	1.09E-07	2.98E-08	ABL1/AKT1/BCL2/GSK3B/HSP90AA1/IL10/KDR/MAPT/SNCA/SRC
GO:0062013	positive regulation of small molecule metabolic process	7/32	153/18670	6.13E-09	1.09E-07	2.98E-08	AKT1/IGF1/IL1B/PTGS2/SNCA/SRC/TNF
GO:0031667	response to nutrient levels	10/32	499/18670	6.47E-09	1.14E-07	3.11E-08	AKT1/CCND1/BCL2/ICAM1/IL1B/JUN/MAPK1/PTGS2/SRC/TGFB1

GO:0031331	positive regulation of cellular catabolic process	9/32	361/18670	6.48E-09	1.14E-07	3.11E-08	AKT1/CAV1/GSK3B/HSP90AA1/IGF1/IL1B/KDR/SNCA/TNF
GO:0001776	leukocyte homeostasis	6/32	86/18670	6.59E-09	1.14E-07	3.11E-08	ABL1/AKT1/BCL2/IL2/JAK3/TGFB1
GO:0007260	tyrosine phosphorylation of STAT protein	6/32	86/18670	6.59E-09	1.14E-07	3.11E-08	CAV1/IGF1/IL2/IL13/JAK3/TNF
GO:0002791	regulation of peptide secretion	10/32	500/18670	6.59E-09	1.14E-07	3.11E-08	ABL1/IGF1/IL1A/IL1B/IL2/IL10/IL13/SRC/TGFB1/TNF
GO:0007259	JAK-STAT cascade	7/32	156/18670	7.02E-09	1.20E-07	3.27E-08	CAV1/IGF1/IL2/IL10/IL13/JAK3/TNF
GO:0030856	regulation of epithelial cell differentiation	7/32	156/18670	7.02E-09	1.20E-07	3.27E-08	CCND1/CAV1/CTNNB1/GSK3B/IL1B/IL13/TNF
GO:0045844	positive regulation of striated muscle tissue development	6/32	87/18670	7.07E-09	1.20E-07	3.27E-08	BCL2/CTNNB1/FGF2/IGF1/MAPK1/TGFB1
GO:0048636	positive regulation of muscle organ development	6/32	87/18670	7.07E-09	1.20E-07	3.27E-08	BCL2/CTNNB1/FGF2/IGF1/MAPK1/TGFB1
GO:0043271	negative regulation of ion transport	7/32	157/18670	7.33E-09	1.24E-07	3.37E-08	AKT1/BCL2/CAV1/ICAM1/PTGS2/SNCA/TGFB1
GO:1901863	positive regulation of muscle tissue development	6/32	88/18670	7.58E-09	1.26E-07	3.45E-08	BCL2/CTNNB1/FGF2/IGF1/MAPK1/TGFB1
GO:1904035	regulation of epithelial cell apoptotic process	6/32	88/18670	7.58E-09	1.26E-07	3.45E-08	ABL1/FASLG/ICAM1/IL13/KDR/TNF
GO:0002819	regulation of adaptive immune response	7/32	160/18670	8.37E-09	1.39E-07	3.79E-08	IL1B/IL2/IL10/IRF1/JAK3/TGFB1/TNF
GO:0043406	positive regulation of MAP kinase activity	8/32	258/18670	9.42E-09	1.56E-07	4.24E-08	ERBB2/FGF2/IGF1/IL1B/MAPK1/SRC/TGFB1/TNF

GO:0045931	positive regulation of mitotic cell cycle	7/32	163/18670	9.52E-09	1.56E-07	4.26E-08	ABL1/AKT1/CCND1/IGF1/IL1A/IL1B/TGFB1
GO:0051091	positive regulation of DNA-binding transcription factor activity	8/32	261/18670	1.03E-08	1.69E-07	4.60E-08	AKT1/CAV1/CTNNB1/ICAM1/IL1B/IL10/TGFB1/TNF
GO:0097696	STAT cascade	7/32	166/18670	1.08E-08	1.76E-07	4.79E-08	CAV1/IGF1/IL2/IL10/IL13/JAK3/TNF
GO:0042116	macrophage activation	6/32	95/18670	1.20E-08	1.95E-07	5.32E-08	IL10/IL13/JUN/MAPT/SNCA/TNF
GO:0045787	positive regulation of cell cycle	9/32	389/18670	1.24E-08	2.00E-07	5.44E-08	ABL1/AKT1/CCND1/IGF1/IL1A/IL1B/IL10/SRC/TGFB1
GO:0045927	positive regulation of growth	8/32	270/18670	1.34E-08	2.15E-07	5.87E-08	AKT1/BCL2/ERBB2/FGF2/IGF1/IL2/MAPT/MAPK1
GO:2001239	regulation of extrinsic apoptotic signaling pathway in absence of ligand	5/32	47/18670	1.55E-08	2.48E-07	6.76E-08	AKT1/BCL2/IL1A/IL1B/TNF
GO:0001936	regulation of endothelial cell proliferation	7/32	176/18670	1.62E-08	2.57E-07	7.01E-08	AKT1/CAV1/FGF2/IL10/JUN/KDR/TNF
GO:0031649	heat generation	4/32	17/18670	1.66E-08	2.63E-07	7.17E-08	IL1A/IL1B/PTGS2/TNF
GO:0001774	microglial cell activation	5/32	48/18670	1.73E-08	2.70E-07	7.35E-08	IL13/JUN/MAPT/SNCA/TNF
GO:0002269	leukocyte activation involved in inflammatory response	5/32	48/18670	1.73E-08	2.70E-07	7.35E-08	IL13/JUN/MAPT/SNCA/TNF
GO:0051972	regulation of telomerase activity	5/32	48/18670	1.73E-08	2.70E-07	7.35E-08	CTNNB1/HSP90AA1/MYC/MAPK1/SRC
GO:0051099	positive regulation of binding	7/32	179/18670	1.82E-08	2.82E-07	7.68E-08	ABL1/PARP1/CAV1/CTNNB1/GSK3B/IGF1/TGFB1

GO:0010522	regulation of calcium ion transport into cytosol	6/32	102/18670	1.85E-08	2.85E-07	7.77E-08	ABL1/BCL2/CAV1/IL13/SNCA/TGFB1
GO:0030857	negative regulation of epithelial cell differentiation	5/32	49/18670	1.93E-08	2.94E-07	8.02E-08	CCND1/CAV1/CTNNB1/GSK3B/IL13
GO:1904707	positive regulation of vascular smooth muscle cell proliferation	5/32	49/18670	1.93E-08	2.94E-07	8.02E-08	FGF2/IGF1/IL10/JUN/TNF
GO:0019216	regulation of lipid metabolic process	9/32	410/18670	1.95E-08	2.96E-07	8.06E-08	AKT1/CAV1/FGF2/IL1B/PTGS2/SNCA/SRC/TGFB1/TNF
GO:1902106	negative regulation of leukocyte differentiation	6/32	103/18670	1.96E-08	2.96E-07	8.06E-08	CTNNB1/ERBB2/IL2/IRF1/JAK3/MYC
GO:0048015	phosphatidylinositol-mediated signaling	7/32	181/18670	1.97E-08	2.96E-07	8.06E-08	AKT1/ERBB2/IGF1/KDR/MAPK1/SRC/TNF
GO:0010821	regulation of mitochondrion organization	7/32	182/18670	2.04E-08	3.06E-07	8.34E-08	AKT1/BCL2/CASP8/GSK3B/IGF1/KDR/MAPT
GO:0043123	positive regulation of I-kappaB kinase/NF-kappaB signaling	7/32	183/18670	2.12E-08	3.16E-07	8.62E-08	ABL1/AKT1/FASLG/CASP8/CTNNB1/IL1B/TNF
GO:0048017	inositol lipid-mediated signaling	7/32	184/18670	2.20E-08	3.25E-07	8.86E-08	AKT1/ERBB2/IGF1/KDR/MAPK1/SRC/TNF
GO:0001558	regulation of cell growth	9/32	416/18670	2.21E-08	3.25E-07	8.86E-08	ABL1/AKT1/BCL2/ERBB2/GSK3B/IGF1/IL2/MAPT/TGFB1
GO:0030099	myeloid cell differentiation	9/32	416/18670	2.21E-08	3.25E-07	8.86E-08	PARP1/CASP8/CTNNB1/JAK3/JUN/MYC/SRC/TGFB1/TNF
GO:0097193	intrinsic apoptotic signaling pathway	8/32	289/18670	2.28E-08	3.34E-07	9.10E-08	ABL1/PARP1/AKT1/BCL2/CAV1/PTGS2/SRC/TNF
GO:0034330	cell junction organization	8/32	290/18670	2.34E-08	3.41E-07	9.30E-08	ABL1/BCL2/CAV1/CTNNB1/KDR/SRC/TGFB1/TNF

GO:0048146	positive regulation of fibroblast proliferation	5/32	51/18670	2.37E-08	3.42E-07	9.32E-08	IGF1/IL13/JUN/MYC/TGFB1
GO:0050999	regulation of nitric-oxide synthase activity	5/32	51/18670	2.37E-08	3.42E-07	9.32E-08	AKT1/CAV1/HSP90AA1/IL1B/TNF
GO:0010632	regulation of epithelial cell migration	8/32	291/18670	2.41E-08	3.46E-07	9.42E-08	ABL1/AKT1/FGF2/JUN/KDR/PTGS2/SRC/TGFB1
GO:0009896	positive regulation of catabolic process	9/32	423/18670	2.55E-08	3.66E-07	9.96E-08	AKT1/CAV1/GSK3B/HSP90AA1/IGF1/IL1B/KDR/SNCA/TNF
GO:2000278	regulation of DNA biosynthetic process	6/32	108/18670	2.61E-08	3.72E-07	1.01E-07	CTNNB1/FGF2/HSP90AA1/MYC/MAPK1/SRC
GO:0052548	regulation of endopeptidase activity	9/32	425/18670	2.66E-08	3.77E-07	1.03E-07	AKT1/FASLG/CASP8/MAPT/MYC/PTGS2/SNCA/SRC/TNF
GO:0001935	endothelial cell proliferation	7/32	191/18670	2.85E-08	4.02E-07	1.10E-07	AKT1/CAV1/FGF2/IL10/JUN/KDR/TNF
GO:0001933	negative regulation of protein phosphorylation	9/32	429/18670	2.88E-08	4.05E-07	1.10E-07	ABL1/AKT1/CAV1/IL1B/IL2/JUN/MYC/SNCA/TGFB1
GO:1901654	response to ketone	7/32	193/18670	3.06E-08	4.28E-07	1.17E-07	PARP1/AKT1/CCND1/CAV1/ICAM1/SRC/TGFB1
GO:0051353	positive regulation of oxidoreductase activity	5/32	54/18670	3.18E-08	4.43E-07	1.21E-07	ABL1/AKT1/IL1B/SNCA/TNF
GO:0050868	negative regulation of T cell activation	6/32	112/18670	3.25E-08	4.51E-07	1.23E-07	ERBB2/IL2/IL10/IRF1/JAK3/TGFB1
GO:0050804	modulation of chemical synaptic transmission	9/32	436/18670	3.31E-08	4.58E-07	1.25E-07	ABL1/GSK3B/IL1B/MAPT/MAPK1/PTGS2/SNCA/SRC/TNF
GO:0099177	regulation of trans-synaptic signaling	9/32	437/18670	3.38E-08	4.65E-07	1.27E-07	ABL1/GSK3B/IL1B/MAPT/MAPK1/PTGS2/SNCA/SRC/TNF

GO:0010952	positive regulation of peptidase activity	7/32	197/18670	3.52E-08	4.83E-07	1.32E-07	FASLG/CASP8/CAV1/MAPT/MYC/SNCA/TNF
GO:0042113	B cell activation	8/32	310/18670	3.93E-08	5.36E-07	1.46E-07	ABL1/BCL2/CASP8/IL2/IL10/IL13/JAK3/TGFB1
GO:0002703	regulation of leukocyte mediated immunity	7/32	201/18670	4.05E-08	5.50E-07	1.50E-07	IL1B/IL2/IL10/IL13/JAK3/TGFB1/TNF
GO:0051900	regulation of mitochondrial depolarization	4/32	21/18670	4.17E-08	5.63E-07	1.54E-07	PARP1/BCL2/KDR/SRC
GO:0009314	response to radiation	9/32	448/18670	4.18E-08	5.63E-07	1.54E-07	PARP1/AKT1/CCND1/BCL2/ICAM1/JUN/MYC/PTGS2/TGFB1
GO:0002761	regulation of myeloid leukocyte differentiation	6/32	117/18670	4.22E-08	5.66E-07	1.54E-07	CASP8/CTNNB1/JUN/MYC/TGFB1/TNF
GO:0045766	positive regulation of angiogenesis	7/32	204/18670	4.48E-08	5.98E-07	1.63E-07	ABL1/FGF2/IL1A/IL1B/IL10/KDR/PTGS2
GO:0031663	lipopolysaccharide-mediated signaling pathway	5/32	58/18670	4.58E-08	6.09E-07	1.66E-07	AKT1/IL1B/MAPK1/TGFB1/TNF
GO:0001658	branching involved in ureteric bud morphogenesis	5/32	59/18670	5.00E-08	6.62E-07	1.80E-07	BCL2/CTNNB1/FGF2/MYC/TGFB1
GO:0006874	cellular calcium ion homeostasis	9/32	458/18670	5.05E-08	6.67E-07	1.82E-07	ABL1/FASLG/BCL2/CAV1/FGF2/IL2/IL13/SNCA/TGFB1
GO:0071375	cellular response to peptide hormone stimulus	8/32	321/18670	5.14E-08	6.75E-07	1.84E-07	PARP1/AKT1/CAV1/GSK3B/IL1B/JAK3/SRC/TGFB1
GO:0060562	epithelial tube morphogenesis	8/32	322/18670	5.27E-08	6.89E-07	1.88E-07	ABL1/BCL2/CTNNB1/FGF2/MYC/SRC/TGFB1/TNF
GO:0032103	positive regulation of response to external stimulus	8/32	323/18670	5.39E-08	7.03E-07	1.92E-07	FGF2/IL1B/IL2/KDR/PTGS2/SNCA/TGFB1/TNF

GO:1905477	positive regulation of protein localization to membrane	6/32	122/18670	5.42E-08	7.04E-07	1.92E-07	AKT1/BCL2/CASP8/ERBB2/TGFB1/TNF
GO:0043523	regulation of neuron apoptotic process	7/32	210/18670	5.46E-08	7.06E-07	1.93E-07	PARP1/FASLG/BCL2/CTNNB1/JUN/SNCA/TNF
GO:0010506	regulation of autophagy	8/32	327/18670	5.93E-08	7.63E-07	2.08E-07	ABL1/AKT1/BCL2/GSK3B/IL10/KDR/MAPT/SNCA
GO:0008637	apoptotic mitochondrial changes	6/32	124/18670	5.98E-08	7.64E-07	2.08E-07	AKT1/BCL2/CASP8/GSK3B/IGF1/JUN
GO:0051209	release of sequestered calcium ion into cytosol	6/32	124/18670	5.98E-08	7.64E-07	2.08E-07	ABL1/FASLG/FGF2/IL13/SNCA/TGFB1
GO:0032388	positive regulation of intracellular transport	7/32	215/18670	6.42E-08	8.13E-07	2.21E-07	ERBB2/GSK3B/IL1B/IL13/MAPK1/PTGS2/TGFB1
GO:0055074	calcium ion homeostasis	9/32	471/18670	6.42E-08	8.13E-07	2.21E-07	ABL1/FASLG/BCL2/CAV1/FGF2/IL2/IL13/SNCA/TGFB1
GO:0046824	positive regulation of nucleocytoplasmic transport	5/32	62/18670	6.44E-08	8.13E-07	2.21E-07	GSK3B/IL1B/MAPK1/PTGS2/TGFB1
GO:0051283	negative regulation of sequestering of calcium ion	6/32	126/18670	6.58E-08	8.27E-07	2.25E-07	ABL1/FASLG/FGF2/IL13/SNCA/TGFB1
GO:0032147	activation of protein kinase activity	8/32	333/18670	6.82E-08	8.53E-07	2.33E-07	ABL1/AKT1/FGF2/IGF1/IL1B/MAPK1/SRC/TNF
GO:0043393	regulation of protein binding	7/32	217/18670	6.84E-08	8.53E-07	2.33E-07	ABL1/AKT1/BCL2/CAV1/GSK3B/IL10/SRC
GO:0002637	regulation of immunoglobulin production	5/32	63/18670	6.99E-08	8.68E-07	2.37E-07	IL2/IL10/IL13/TGFB1/TNF
GO:0010595	positive regulation of endothelial cell migration	6/32	128/18670	7.23E-08	8.91E-07	2.43E-07	ABL1/AKT1/FGF2/KDR/PTGS2/TGFB1

GO:0051282	regulation of sequestering of calcium ion	6/32	128/18670	7.23E-08	8.91E-07	2.43E-07	ABL1/FASLG/FGF2/IL13/SNCA/TGFB1
GO:1903672	positive regulation of sprouting angiogenesis	5/32	64/18670	7.57E-08	9.30E-07	2.53E-07	ABL1/FGF2/IL10/KDR/PTGS2
GO:0050671	positive regulation of lymphocyte proliferation	6/32	130/18670	7.93E-08	9.70E-07	2.64E-07	BCL2/IGF1/IL1B/IL2/IL13/JAK3
GO:0060675	ureteric bud morphogenesis	5/32	65/18670	8.19E-08	9.98E-07	2.72E-07	BCL2/CTNNB1/FGF2/MYC/TGFB1
GO:0048871	multicellular organismal homeostasis	9/32	485/18670	8.25E-08	1.00E-06	2.73E-07	BCL2/CAV1/CTNNB1/IL1A/IL1B/IL13/PTGS2/SRC/TNF
GO:0032946	positive regulation of mononuclear cell proliferation	6/32	131/18670	8.30E-08	1.00E-06	2.73E-07	BCL2/IGF1/IL1B/IL2/IL13/JAK3
GO:0051208	sequestering of calcium ion	6/32	131/18670	8.30E-08	1.00E-06	2.73E-07	ABL1/FASLG/FGF2/IL13/SNCA/TGFB1
GO:0043280	positive regulation of cysteine-type endopeptidase activity involved in apoptotic process	6/32	132/18670	8.68E-08	1.04E-06	2.84E-07	FASLG/CASP8/MAPT/MYC/SNCA/TNF
GO:0002719	negative regulation of cytokine production involved in immune response	4/32	25/18670	8.76E-08	1.04E-06	2.85E-07	IL10/JAK3/TGFB1/TNF
GO:2000679	positive regulation of transcription regulatory region DNA binding	4/32	25/18670	8.76E-08	1.04E-06	2.85E-07	PARP1/CTNNB1/IGF1/TGFB1
GO:0072171	mesonephric tubule morphogenesis	5/32	66/18670	8.85E-08	1.05E-06	2.86E-07	BCL2/CTNNB1/FGF2/MYC/TGFB1
GO:0048638	regulation of developmental growth	8/32	347/18670	9.36E-08	1.11E-06	3.02E-07	ABL1/AKT1/BCL2/FGF2/GSK3B/IGF1/MAPT/MAPK1
GO:0072503	cellular divalent inorganic cation homeostasis	9/32	493/18670	9.48E-08	1.12E-06	3.04E-07	ABL1/FASLG/BCL2/CAV1/FGF2/IL2/IL13/SNCA/TGFB1

GO:0032355	response to estradiol	6/32	134/18670	9.50E-08	1.12E-06	3.04E-07	CCND1/CASP8/CTNNB1/IL10/PTGS2/TGFB1
GO:1904018	positive regulation of vasculature development	7/32	230/18670	1.02E-07	1.19E-06	3.25E-07	ABL1/FGF2/IL1A/IL1B/IL10/KDR/PTGS2
GO:0010631	epithelial cell migration	8/32	351/18670	1.02E-07	1.19E-06	3.25E-07	ABL1/AKT1/FGF2/JUN/KDR/PTGS2/SRC/TGFB1
GO:0034605	cellular response to heat	6/32	137/18670	1.08E-07	1.26E-06	3.43E-07	GSK3B/HSP90AA1/IL1A/MAPT/MAPK1/PTGS2
GO:0090132	epithelium migration	8/32	354/18670	1.09E-07	1.26E-06	3.44E-07	ABL1/AKT1/FGF2/JUN/KDR/PTGS2/SRC/TGFB1
GO:0070227	lymphocyte apoptotic process	5/32	69/18670	1.11E-07	1.28E-06	3.48E-07	AKT1/FASLG/IL2/IL10/JAK3
GO:0071453	cellular response to oxygen levels	7/32	234/18670	1.14E-07	1.32E-06	3.59E-07	AKT1/BCL2/CAV1/ICAM1/MYC/PTGS2/SRC
GO:0045580	regulation of T cell differentiation	6/32	139/18670	1.18E-07	1.34E-06	3.66E-07	ABL1/ERBB2/IL2/IRF1/JAK3/TGFB1
GO:0050715	positive regulation of cytokine secretion	6/32	139/18670	1.18E-07	1.34E-06	3.66E-07	ABL1/IL1A/IL1B/IL10/SRC/TNF
GO:0070665	positive regulation of leukocyte proliferation	6/32	139/18670	1.18E-07	1.34E-06	3.66E-07	BCL2/IGF1/IL1B/IL2/IL13/JAK3
GO:0001666	response to hypoxia	8/32	359/18670	1.22E-07	1.38E-06	3.75E-07	AKT1/BCL2/CAV1/ICAM1/MYC/PTGS2/SRC/TGFB1
GO:0097553	calcium ion transmembrane import into cytosol	6/32	140/18670	1.23E-07	1.39E-06	3.79E-07	ABL1/FASLG/FGF2/IL13/SNCA/TGFB1
GO:0090130	tissue migration	8/32	360/18670	1.24E-07	1.40E-06	3.81E-07	ABL1/AKT1/FGF2/JUN/KDR/PTGS2/SRC/TGFB1

GO:0043122	regulation of I-kappaB kinase/NF-kappaB signaling	7/32	237/18670	1.25E-07	1.40E-06	3.81E-07	ABL1/AKT1/FASLG/CASP8/CTNNB1/IL1B/TNF
GO:0009411	response to UV	6/32	141/18670	1.29E-07	1.44E-06	3.91E-07	PARP1/AKT1/CCND1/BCL2/MYC/PTGS2
GO:0051402	neuron apoptotic process	7/32	239/18670	1.32E-07	1.47E-06	4.01E-07	PARP1/FASLG/BCL2/CTNNB1/JUN/SNCA/TNF
GO:0061180	mammary gland epithelium development	5/32	72/18670	1.38E-07	1.53E-06	4.16E-07	AKT1/CCND1/MAPK1/SRC/TGFB1
GO:0030879	mammary gland development	6/32	143/18670	1.40E-07	1.54E-06	4.20E-07	AKT1/CCND1/CAV1/MAPK1/SRC/TGFB1
GO:0034329	cell junction assembly	7/32	241/18670	1.40E-07	1.54E-06	4.20E-07	ABL1/BCL2/CAV1/CTNNB1/KDR/SRC/TNF
GO:0002822	regulation of adaptive immune response based on somatic recombination of immune receptors built from immunoglobulin superfamily domains	6/32	145/18670	1.52E-07	1.67E-06	4.54E-07	IL1B/IL2/IL10/JAK3/TGFB1/TNF
GO:0051896	regulation of protein kinase B signaling	7/32	244/18670	1.52E-07	1.67E-06	4.54E-07	AKT1/ERBB2/FGF2/HSP90AA1/SRC/TGFB1/TNF
GO:0036293	response to decreased oxygen levels	8/32	370/18670	1.53E-07	1.67E-06	4.55E-07	AKT1/BCL2/CAV1/ICAM1/MYC/PTGS2/SRC/TGFB1
GO:0072078	nephron tubule morphogenesis	5/32	74/18670	1.58E-07	1.71E-06	4.67E-07	BCL2/CTNNB1/FGF2/MYC/TGFB1
GO:0051250	negative regulation of lymphocyte activation	6/32	146/18670	1.58E-07	1.71E-06	4.67E-07	ERBB2/IL2/IL10/IRF1/JAK3/TGFB1
GO:0010575	positive regulation of vascular endothelial growth factor production	4/32	29/18670	1.64E-07	1.77E-06	4.82E-07	IL1A/IL1B/PTGS2/TGFB1

GO:0010212	response to ionizing radiation	6/32	147/18670	1.65E-07	1.77E-06	4.83E-07	PARP1/CCND1/BCL2/ICAM1/MYC/TGFB1
GO:2001056	positive regulation of cysteine-type endopeptidase activity	6/32	149/18670	1.78E-07	1.91E-06	5.21E-07	FASLG/CASP8/MAPT/MYC/SNCA/TNF
GO:0072088	nephron epithelium morphogenesis	5/32	76/18670	1.81E-07	1.93E-06	5.26E-07	BCL2/CTNNB1/FGF2/MYC/TGFB1
GO:0042176	regulation of protein catabolic process	8/32	381/18670	1.92E-07	2.04E-06	5.56E-07	AKT1/CAV1/GSK3B/HSP90AA1/IL1B/IL10/SNCA/TNF
GO:0016202	regulation of striated muscle tissue development	6/32	152/18670	2.01E-07	2.13E-06	5.81E-07	BCL2/CTNNB1/FGF2/IGF1/MAPK1/TGFB1
GO:0061333	renal tubule morphogenesis	5/32	78/18670	2.06E-07	2.17E-06	5.92E-07	BCL2/CTNNB1/FGF2/MYC/TGFB1
GO:0072028	nephron morphogenesis	5/32	78/18670	2.06E-07	2.17E-06	5.92E-07	BCL2/CTNNB1/FGF2/MYC/TGFB1
GO:0090316	positive regulation of intracellular protein transport	6/32	153/18670	2.09E-07	2.19E-06	5.98E-07	ERBB2/GSK3B/IL1B/MAPK1/PTGS2/TGFB1
GO:1901861	regulation of muscle tissue development	6/32	155/18670	2.25E-07	2.35E-06	6.41E-07	BCL2/CTNNB1/FGF2/IGF1/MAPK1/TGFB1
GO:1903707	negative regulation of hemopoiesis	6/32	155/18670	2.25E-07	2.35E-06	6.41E-07	CTNNB1/ERBB2/IL2/IRF1/JAK3/MYC
GO:0043535	regulation of blood vessel endothelial cell migration	6/32	156/18670	2.34E-07	2.43E-06	6.62E-07	ABL1/AKT1/FGF2/KDR/PTGS2/TGFB1
GO:0048634	regulation of muscle organ development	6/32	156/18670	2.34E-07	2.43E-06	6.62E-07	BCL2/CTNNB1/FGF2/IGF1/MAPK1/TGFB1
GO:0034504	protein localization to nucleus	7/32	262/18670	2.47E-07	2.54E-06	6.93E-07	PARP1/AKT1/GSK3B/MAPK1/PTGS2/SRC/TGFB1

GO:0045907	positive regulation of vasoconstriction	4/32	32/18670	2.47E-07	2.54E-06	6.93E-07	AKT1/CAV1/ICAM1/PTGS2
GO:0070482	response to oxygen levels	8/32	394/18670	2.47E-07	2.54E-06	6.93E-07	AKT1/BCL2/CAV1/ICAM1/MYC/PTGS2/SRC/TGFB1
GO:0032204	regulation of telomere maintenance	5/32	81/18670	2.49E-07	2.54E-06	6.93E-07	PARP1/CTNNB1/MYC/MAPK1/SRC
GO:0048708	astrocyte differentiation	5/32	81/18670	2.49E-07	2.54E-06	6.93E-07	ABL1/IL1B/MAPT/MAPK1/TNF
GO:0010833	telomere maintenance via telomere lengthening	5/32	82/18670	2.65E-07	2.70E-06	7.35E-07	PARP1/CTNNB1/HSP90AA1/MAPK1/SRC
GO:0001503	ossification	8/32	398/18670	2.67E-07	2.71E-06	7.39E-07	AKT1/BCL2/CTNNB1/IGF1/MAPK1/PTGS2/TGFB1/TNF
GO:2000352	negative regulation of endothelial cell apoptotic process	4/32	33/18670	2.81E-07	2.84E-06	7.74E-07	ABL1/ICAM1/IL13/KDR
GO:0042509	regulation of tyrosine phosphorylation of STAT protein	5/32	83/18670	2.82E-07	2.84E-06	7.74E-07	CAV1/IGF1/IL2/IL13/TNF
GO:0000723	telomere maintenance	6/32	162/18670	2.92E-07	2.94E-06	8.01E-07	PARP1/CTNNB1/HSP90AA1/MYC/MAPK1/SRC
GO:0007249	I-kappaB kinase/NF-kappaB signaling	7/32	269/18670	2.95E-07	2.95E-06	8.05E-07	ABL1/AKT1/FASLG/CASP8/CTNNB1/IL1B/TNF
GO:0002718	regulation of cytokine production involved in immune response	5/32	84/18670	2.99E-07	2.99E-06	8.14E-07	IL1B/IL10/JAK3/TGFB1/TNF
GO:1904705	regulation of vascular smooth muscle cell proliferation	5/32	85/18670	3.17E-07	3.13E-06	8.52E-07	FGF2/IGF1/IL10/JUN/TNF
GO:1990874	vascular smooth muscle cell proliferation	5/32	85/18670	3.17E-07	3.13E-06	8.52E-07	FGF2/IGF1/IL10/JUN/TNF

GO:0010574	regulation of vascular endothelial growth factor production	4/32	34/18670	3.18E-07	3.13E-06	8.52E-07	IL1A/IL1B/PTGS2/TGFB1
GO:0043276	anoikis	4/32	34/18670	3.18E-07	3.13E-06	8.52E-07	AKT1/BCL2/CAV1/SRC
GO:0032868	response to insulin	7/32	272/18670	3.18E-07	3.13E-06	8.52E-07	PARP1/AKT1/GSK3B/ICAM1/IL1B/IL10/SRC
GO:2001242	regulation of intrinsic apoptotic signaling pathway	6/32	165/18670	3.26E-07	3.19E-06	8.71E-07	PARP1/AKT1/BCL2/CAV1/PTGS2/SRC
GO:0006919	activation of cysteine-type endopeptidase activity involved in apoptotic process	5/32	86/18670	3.37E-07	3.28E-06	8.94E-07	FASLG/CASP8/MAPT/SNCA/TNF
GO:0008625	extrinsic apoptotic signaling pathway via death domain receptors	5/32	86/18670	3.37E-07	3.28E-06	8.94E-07	FASLG/BCL2/CASP8/ICAM1/TNF
GO:0002701	negative regulation of production of molecular mediator of immune response	4/32	35/18670	3.58E-07	3.48E-06	9.49E-07	IL10/JAK3/TGFB1/TNF
GO:0050806	positive regulation of synaptic transmission	6/32	168/18670	3.62E-07	3.51E-06	9.56E-07	ABL1/GSK3B/MAPK1/PTGS2/SNCA/TNF
GO:0060485	mesenchyme development	7/32	278/18670	3.68E-07	3.56E-06	9.69E-07	BCL2/CTNNB1/GSK3B/IL1B/MYC/MAPK1/TGFB1
GO:0030307	positive regulation of cell growth	6/32	169/18670	3.75E-07	3.60E-06	9.82E-07	AKT1/BCL2/ERBB2/IGF1/IL2/MAPT
GO:0045619	regulation of lymphocyte differentiation	6/32	169/18670	3.75E-07	3.60E-06	9.82E-07	ABL1/ERBB2/IL2/IRF1/JAK3/TGFB1
GO:1900407	regulation of cellular response to oxidative stress	5/32	88/18670	3.78E-07	3.60E-06	9.82E-07	PARP1/AKT1/CTNNB1/IL10/TNF
GO:1901888	regulation of cell junction assembly	5/32	88/18670	3.78E-07	3.60E-06	9.82E-07	ABL1/CAV1/KDR/SRC/TNF

GO:0030100	regulation of endocytosis	7/32	281/18670	3.96E-07	3.77E-06	1.03E-06	ABL1/CAV1/IL1B/SNCA/SRC/TGFB1/TNF
GO:0046427	positive regulation of JAK-STAT cascade	5/32	89/18670	4.00E-07	3.79E-06	1.03E-06	IGF1/IL2/IL10/IL13/TNF
GO:0010573	vascular endothelial growth factor production	4/32	36/18670	4.03E-07	3.80E-06	1.03E-06	IL1A/IL1B/PTGS2/TGFB1
GO:0051973	positive regulation of telomerase activity	4/32	36/18670	4.03E-07	3.80E-06	1.03E-06	CTNNB1/HSP90AA1/MYC/MAPK1
GO:0034333	adherens junction assembly	5/32	90/18670	4.23E-07	3.97E-06	1.08E-06	ABL1/BCL2/CTNNB1/KDR/SRC
GO:0032386	regulation of intracellular transport	8/32	423/18670	4.24E-07	3.98E-06	1.08E-06	ERBB2/GSK3B/IL1B/IL13/MAPK1/PTGS2/SRC/TGFB1
GO:0001659	temperature homeostasis	6/32	173/18670	4.31E-07	4.01E-06	1.09E-06	CAV1/IL1A/IL1B/IL13/PTGS2/TNF
GO:0003018	vascular process in circulatory system	6/32	173/18670	4.31E-07	4.01E-06	1.09E-06	AKT1/CAV1/ICAM1/PTGS2/SRC/TGFB1
GO:0051348	negative regulation of transferase activity	7/32	285/18670	4.36E-07	4.05E-06	1.10E-06	ABL1/AKT1/CAV1/GSK3B/IL1B/MAPT/SRC
GO:0002440	production of molecular mediator of immune response	7/32	286/18670	4.46E-07	4.12E-06	1.12E-06	IL1B/IL2/IL10/IL13/JAK3/TGFB1/TNF
GO:0030212	hyaluronan metabolic process	4/32	37/18670	4.51E-07	4.12E-06	1.12E-06	AKT1/FGF2/IL1B/TGFB1
GO:0032885	regulation of polysaccharide biosynthetic process	4/32	37/18670	4.51E-07	4.12E-06	1.12E-06	AKT1/GSK3B/IGF1/TGFB1
GO:0034405	response to fluid shear stress	4/32	37/18670	4.51E-07	4.12E-06	1.12E-06	AKT1/PTGS2/SRC/TGFB1

GO:0038083	peptidyl-tyrosine autophosphorylation	4/32	37/18670	4.51E-07	4.12E-06	1.12E-06	ABL1/CAV1/KDR/SRC
GO:0090050	positive regulation of cell migration involved in sprouting angiogenesis	4/32	37/18670	4.51E-07	4.12E-06	1.12E-06	ABL1/FGF2/KDR/PTGS2
GO:0002695	negative regulation of leukocyte activation	6/32	175/18670	4.61E-07	4.18E-06	1.14E-06	ERBB2/IL2/IL10/IRF1/JAK3/TGFB1
GO:0032200	telomere organization	6/32	175/18670	4.61E-07	4.18E-06	1.14E-06	PARP1/CTNNB1/HSP90AA1/MYC/MAPK1/SRC
GO:0048568	embryonic organ development	8/32	428/18670	4.64E-07	4.20E-06	1.14E-06	AKT1/CASP8/CTNNB1/IL10/KDR/MAPK1/TGFB1/TNF
GO:1904894	positive regulation of STAT cascade	5/32	92/18670	4.72E-07	4.26E-06	1.16E-06	IGF1/IL2/IL10/IL13/TNF
GO:0051897	positive regulation of protein kinase B signaling	6/32	176/18670	4.76E-07	4.29E-06	1.17E-06	ERBB2/FGF2/HSP90AA1/SRC/TGFB1/TNF
GO:0036473	cell death in response to oxidative stress	5/32	93/18670	4.98E-07	4.46E-06	1.22E-06	PARP1/AKT1/BCL2/CTNNB1/IL10
GO:0072080	nephron tubule development	5/32	93/18670	4.98E-07	4.46E-06	1.22E-06	BCL2/CTNNB1/FGF2/MYC/TGFB1
GO:0010950	positive regulation of endopeptidase activity	6/32	178/18670	5.09E-07	4.55E-06	1.24E-06	FASLG/CASP8/MAPT/MYC/SNCA/TNF
GO:0060993	kidney morphogenesis	5/32	94/18670	5.25E-07	4.67E-06	1.27E-06	BCL2/CTNNB1/FGF2/MYC/TGFB1
GO:0048771	tissue remodeling	6/32	179/18670	5.26E-07	4.67E-06	1.27E-06	CAV1/CTNNB1/IL1A/IL2/SRC/TGFB1
GO:0060249	anatomical structure homeostasis	8/32	437/18670	5.43E-07	4.80E-06	1.31E-06	PARP1/BCL2/CTNNB1/HSP90AA1/MYC/MAPK1/SRC/TGFB1

GO:0043534	blood vessel endothelial cell migration	6/32	180/18670	5.44E-07	4.80E-06	1.31E-06	ABL1/AKT1/FGF2/KDR/PTGS2/TGFB1
GO:0042100	B cell proliferation	5/32	95/18670	5.54E-07	4.87E-06	1.33E-06	ABL1/BCL2/IL2/IL10/IL13
GO:0061326	renal tubule development	5/32	95/18670	5.54E-07	4.87E-06	1.33E-06	BCL2/CTNNB1/FGF2/MYC/TGFB1
GO:0002285	lymphocyte activation involved in immune response	6/32	181/18670	5.62E-07	4.92E-06	1.34E-06	ABL1/ICAM1/IL2/IL10/JAK3/TGFB1
GO:0019217	regulation of fatty acid metabolic process	5/32	96/18670	5.84E-07	5.10E-06	1.39E-06	AKT1/CAV1/IL1B/PTGS2/SNCA
GO:0002040	sprouting angiogenesis	6/32	183/18670	5.99E-07	5.22E-06	1.42E-06	ABL1/AKT1/FGF2/IL10/KDR/PTGS2
GO:0001657	ureteric bud development	5/32	97/18670	6.15E-07	5.31E-06	1.45E-06	BCL2/CTNNB1/FGF2/MYC/TGFB1
GO:0043255	regulation of carbohydrate biosynthetic process	5/32	97/18670	6.15E-07	5.31E-06	1.45E-06	AKT1/GSK3B/IGF1/SNCA/TGFB1
GO:1902882	regulation of response to oxidative stress	5/32	97/18670	6.15E-07	5.31E-06	1.45E-06	PARP1/AKT1/CTNNB1/IL10/TNF
GO:0048639	positive regulation of developmental growth	6/32	184/18670	6.18E-07	5.33E-06	1.45E-06	AKT1/BCL2/FGF2/IGF1/MAPT/MAPK1
GO:0002042	cell migration involved in sprouting angiogenesis	5/32	98/18670	6.47E-07	5.53E-06	1.51E-06	ABL1/AKT1/FGF2/KDR/PTGS2
GO:0072163	mesonephric epithelium development	5/32	98/18670	6.47E-07	5.53E-06	1.51E-06	BCL2/CTNNB1/FGF2/MYC/TGFB1
GO:0072164	mesonephric tubule development	5/32	98/18670	6.47E-07	5.53E-06	1.51E-06	BCL2/CTNNB1/FGF2/MYC/TGFB1

GO:0048167	regulation of synaptic plasticity	6/32	187/18670	6.80E-07	5.78E-06	1.58E-06	ABL1/GSK3B/MAPT/MAPK1/PTGS2/SNCA
GO:1905475	regulation of protein localization to membrane	6/32	187/18670	6.80E-07	5.78E-06	1.58E-06	AKT1/BCL2/CASP8/ERBB2/TGFB1/TNF
GO:0061028	establishment of endothelial barrier	4/32	41/18670	6.88E-07	5.82E-06	1.59E-06	CTNNB1/ICAM1/IL1B/TNF
GO:0150077	regulation of neuroinflammatory response	4/32	41/18670	6.88E-07	5.82E-06	1.59E-06	IGF1/IL1B/PTGS2/TNF
GO:0071248	cellular response to metal ion	6/32	190/18670	7.46E-07	6.27E-06	1.71E-06	PARP1/AKT1/JUN/MAPK1/PTGS2/SNCA
GO:0031652	positive regulation of heat generation	3/32	11/18670	7.48E-07	6.27E-06	1.71E-06	IL1B/PTGS2/TNF
GO:0072584	caveolin-mediated endocytosis	3/32	11/18670	7.48E-07	6.27E-06	1.71E-06	CAV1/MAPK1/SRC
GO:0032649	regulation of interferon-gamma production	5/32	101/18670	7.52E-07	6.28E-06	1.71E-06	ABL1/IL1B/IL2/IL10/TNF
GO:0046632	alpha-beta T cell differentiation	5/32	101/18670	7.52E-07	6.28E-06	1.71E-06	ABL1/BCL2/IL2/IRF1/JAK3
GO:0010559	regulation of glycoprotein biosynthetic process	4/32	42/18670	7.60E-07	6.33E-06	1.72E-06	BCL2/CTNNB1/IGF1/JAK3
GO:0001823	mesonephros development	5/32	102/18670	7.90E-07	6.54E-06	1.78E-06	BCL2/CTNNB1/FGF2/MYC/TGFB1
GO:0002367	cytokine production involved in immune response	5/32	102/18670	7.90E-07	6.54E-06	1.78E-06	IL1B/IL10/JAK3/TGFB1/TNF
GO:0007565	female pregnancy	6/32	192/18670	7.94E-07	6.56E-06	1.79E-06	AKT1/BCL2/IL1B/MAPK1/PTGS2/TGFB1

GO:0001667	ameboidal-type cell migration	8/32	461/18670	8.12E-07	6.70E-06	1.82E-06	ABL1/AKT1/FGF2/JUN/KDR/PTGS2/SRC/TGFB1
GO:0032881	regulation of polysaccharide metabolic process	4/32	43/18670	8.37E-07	6.86E-06	1.87E-06	AKT1/GSK3B/IGF1/TGFB1
GO:1904646	cellular response to amyloid-beta	4/32	43/18670	8.37E-07	6.86E-06	1.87E-06	PARP1/GSK3B/ICAM1/IGF1
GO:0046822	regulation of nucleocytoplasmic transport	5/32	104/18670	8.70E-07	7.10E-06	1.93E-06	GSK3B/IL1B/MAPK1/PTGS2/TGFB1
GO:0071887	leukocyte apoptotic process	5/32	104/18670	8.70E-07	7.10E-06	1.93E-06	AKT1/FASLG/IL2/IL10/JAK3
GO:0071897	DNA biosynthetic process	6/32	196/18670	8.95E-07	7.28E-06	1.98E-06	CTNNB1/FGF2/HSP90AA1/MYC/MAPK1/SRC
GO:0007409	axonogenesis	8/32	468/18670	9.10E-07	7.39E-06	2.01E-06	ABL1/BCL2/ERBB2/GSK3B/HSP90AA1/MAPT/MAPK1/SRC
GO:0070266	necroptotic process	4/32	44/18670	9.19E-07	7.44E-06	2.03E-06	FASLG/CASP8/CAV1/TNF
GO:0031099	regeneration	6/32	198/18670	9.50E-07	7.67E-06	2.09E-06	CCND1/BCL2/IGF1/IL10/JUN/TGFB1
GO:0050866	negative regulation of cell activation	6/32	199/18670	9.78E-07	7.88E-06	2.15E-06	ERBB2/IL2/IL10/IRF1/JAK3/TGFB1
GO:0045581	negative regulation of T cell differentiation	4/32	45/18670	1.01E-06	8.08E-06	2.20E-06	ERBB2/IL2/IRF1/JAK3
GO:0048538	thymus development	4/32	45/18670	1.01E-06	8.08E-06	2.20E-06	ABL1/BCL2/CTNNB1/MAPK1
GO:0051149	positive regulation of muscle cell differentiation	5/32	109/18670	1.10E-06	8.73E-06	2.38E-06	ABL1/BCL2/CTNNB1/IGF1/TGFB1

GO:0072009	nephron epithelium development	5/32	109/18670	1.10E-06	8.73E-06	2.38E-06	BCL2/CTNNB1/FGF2/MYC/TGFB1
GO:0030278	regulation of ossification	6/32	203/18670	1.10E-06	8.73E-06	2.38E-06	BCL2/CTNNB1/IGF1/MAPK1/TGFB1/TNF
GO:0060711	labyrinthine layer development	4/32	46/18670	1.10E-06	8.73E-06	2.38E-06	AKT1/CASP8/IL10/MAPK1
GO:1904036	negative regulation of epithelial cell apoptotic process	4/32	46/18670	1.10E-06	8.73E-06	2.38E-06	ABL1/ICAM1/IL13/KDR
GO:0006109	regulation of carbohydrate metabolic process	6/32	206/18670	1.20E-06	9.45E-06	2.58E-06	AKT1/GSK3B/IGF1/SNCA/SRC/TGFB1
GO:0006953	acute-phase response	4/32	47/18670	1.20E-06	9.48E-06	2.58E-06	IL1A/IL1B/PTGS2/TNF
GO:0071456	cellular response to hypoxia	6/32	207/18670	1.23E-06	9.67E-06	2.64E-06	AKT1/BCL2/ICAM1/MYC/PTGS2/SRC
GO:0001938	positive regulation of endothelial cell proliferation	5/32	112/18670	1.26E-06	9.85E-06	2.68E-06	AKT1/FGF2/IL10/JUN/KDR
GO:0031650	regulation of heat generation	3/32	13/18670	1.29E-06	1.01E-05	2.76E-06	IL1B/PTGS2/TNF
GO:0051701	interaction with host	6/32	209/18670	1.30E-06	1.02E-05	2.77E-06	CASP8/CAV1/CTNNB1/ICAM1/SRC/TGFB1
GO:0032609	interferon-gamma production	5/32	113/18670	1.31E-06	1.02E-05	2.78E-06	ABL1/IL1B/IL2/IL10/TNF
GO:0050707	regulation of cytokine secretion	6/32	210/18670	1.34E-06	1.04E-05	2.83E-06	ABL1/IL1A/IL1B/IL10/SRC/TNF
GO:0045930	negative regulation of mitotic cell cycle	7/32	338/18670	1.37E-06	1.06E-05	2.88E-06	ABL1/CCND1/BCL2/CTNNB1/IL10/TGFB1/TNF

GO:0042035	regulation of cytokine biosynthetic process	5/32	114/18670	1.37E-06	1.06E-05	2.88E-06	IL1A/IL1B/IL10/IRF1/TNF
GO:0045446	endothelial cell differentiation	5/32	114/18670	1.37E-06	1.06E-05	2.88E-06	CTNNB1/ICAM1/IL1B/KDR/TNF
GO:0003254	regulation of membrane depolarization	4/32	49/18670	1.43E-06	1.09E-05	2.97E-06	PARP1/BCL2/KDR/SRC
GO:0035094	response to nicotine	4/32	49/18670	1.43E-06	1.09E-05	2.97E-06	BCL2/IL13/MAPK1/TNF
GO:0097300	programmed necrotic cell death	4/32	49/18670	1.43E-06	1.09E-05	2.97E-06	FASLG/CASP8/CAV1/TNF
GO:1903018	regulation of glycoprotein metabolic process	4/32	49/18670	1.43E-06	1.09E-05	2.97E-06	BCL2/CTNNB1/IGF1/JAK3
GO:0050900	leukocyte migration	8/32	499/18670	1.47E-06	1.12E-05	3.05E-06	AKT1/CAV1/ICAM1/IL1B/IL10/SRC/TGFB1/TNF
GO:0045732	positive regulation of protein catabolic process	6/32	214/18670	1.49E-06	1.13E-05	3.09E-06	AKT1/CAV1/GSK3B/HSP90AA1/IL1B/TNF
GO:0021782	glial cell development	5/32	116/18670	1.50E-06	1.13E-05	3.09E-06	AKT1/IL1B/MAPT/TGFB1/TNF
GO:0010721	negative regulation of cell development	7/32	344/18670	1.54E-06	1.16E-05	3.16E-06	BCL2/CTNNB1/GSK3B/IGF1/IL1B/TGFB1/TNF
GO:0036294	cellular response to decreased oxygen levels	6/32	217/18670	1.62E-06	1.22E-05	3.32E-06	AKT1/BCL2/ICAM1/MYC/PTGS2/SRC
GO:0071241	cellular response to inorganic substance	6/32	217/18670	1.62E-06	1.22E-05	3.32E-06	PARP1/AKT1/JUN/MAPK1/PTGS2/SNCA
GO:0032886	regulation of microtubule-based process	6/32	218/18670	1.66E-06	1.25E-05	3.40E-06	ABL1/CTNNB1/ERBB2/GSK3B/MAPT/SNCA

GO:0002712	regulation of B cell mediated immunity	4/32	51/18670	1.68E-06	1.25E-05	3.42E-06	IL2/IL10/TGFB1/TNF
GO:0002889	regulation of immunoglobulin mediated immune response	4/32	51/18670	1.68E-06	1.25E-05	3.42E-06	IL2/IL10/TGFB1/TNF
GO:0001952	regulation of cell-matrix adhesion	5/32	119/18670	1.70E-06	1.26E-05	3.44E-06	ABL1/BCL2/GSK3B/KDR/SRC
GO:0007173	epidermal growth factor receptor signaling pathway	5/32	119/18670	1.70E-06	1.26E-05	3.44E-06	ABL1/AKT1/FASLG/SRC/TGFB1
GO:0048762	mesenchymal cell differentiation	6/32	219/18670	1.71E-06	1.27E-05	3.46E-06	BCL2/CTNNB1/GSK3B/IL1B/MAPK1/TGFB1
GO:0002698	negative regulation of immune effector process	5/32	120/18670	1.77E-06	1.31E-05	3.56E-06	IL2/IL10/JAK3/TGFB1/TNF
GO:0022612	gland morphogenesis	5/32	120/18670	1.77E-06	1.31E-05	3.56E-06	BCL2/CAV1/SRC/TGFB1/TNF
GO:2000677	regulation of transcription regulatory region DNA binding	4/32	52/18670	1.82E-06	1.34E-05	3.65E-06	PARP1/CTNNB1/IGF1/TGFB1
GO:0044706	multi-multicellular organism process	6/32	222/18670	1.85E-06	1.36E-05	3.70E-06	AKT1/BCL2/IL1B/MAPK1/PTGS2/TGFB1
GO:0045840	positive regulation of mitotic nuclear division	4/32	53/18670	1.96E-06	1.44E-05	3.92E-06	IGF1/IL1A/IL1B/TGFB1
GO:0042089	cytokine biosynthetic process	5/32	123/18670	2.00E-06	1.46E-05	3.97E-06	IL1A/IL1B/IL10/IRF1/TNF
GO:0007160	cell-matrix adhesion	6/32	225/18670	2.00E-06	1.46E-05	3.97E-06	ABL1/BCL2/CTNNB1/GSK3B/KDR/SRC
GO:0033157	regulation of intracellular protein transport	6/32	225/18670	2.00E-06	1.46E-05	3.97E-06	ERBB2/GSK3B/IL1B/MAPK1/PTGS2/TGFB1

GO:0035635	entry of bacterium into host cell	3/32	15/18670	2.05E-06	1.49E-05	4.07E-06	CAV1/CTNNB1/SRC
GO:0014066	regulation of phosphatidylinositol 3-kinase signaling	5/32	124/18670	2.08E-06	1.50E-05	4.09E-06	IGF1/KDR/MAPK1/SRC/TNF
GO:0042107	cytokine metabolic process	5/32	124/18670	2.08E-06	1.50E-05	4.09E-06	IL1A/IL1B/IL10/IRF1/TNF
GO:0051101	regulation of DNA binding	5/32	124/18670	2.08E-06	1.50E-05	4.09E-06	PARP1/CTNNB1/IGF1/JUN/TGFB1
GO:0002460	adaptive immune response based on somatic recombination of immune receptors built from immunoglobulin superfamily domains	7/32	361/18670	2.12E-06	1.51E-05	4.13E-06	ICAM1/IL1B/IL2/IL10/JAK3/TGFB1/TNF
GO:0002763	positive regulation of myeloid leukocyte differentiation	4/32	54/18670	2.12E-06	1.51E-05	4.13E-06	CASP8/JUN/TGFB1/TNF
GO:0010524	positive regulation of calcium ion transport into cytosol	4/32	54/18670	2.12E-06	1.51E-05	4.13E-06	ABL1/CAV1/IL13/SNCA
GO:1904645	response to amyloid-beta	4/32	54/18670	2.12E-06	1.51E-05	4.13E-06	PARP1/GSK3B/ICAM1/IGF1
GO:0045471	response to ethanol	5/32	125/18670	2.16E-06	1.54E-05	4.21E-06	CCND1/CASP8/ICAM1/IL2/IL13
GO:0010594	regulation of endothelial cell migration	6/32	229/18670	2.21E-06	1.58E-05	4.30E-06	ABL1/AKT1/FGF2/KDR/PTGS2/TGFB1
GO:0018107	peptidyl-threonine phosphorylation	5/32	126/18670	2.25E-06	1.60E-05	4.36E-06	AKT1/BCL2/GSK3B/MAPK1/TGFB1
GO:0045620	negative regulation of lymphocyte differentiation	4/32	55/18670	2.28E-06	1.62E-05	4.41E-06	ERBB2/IL2/IRF1/JAK3

GO:0034763	negative regulation of transmembrane transport	5/32	128/18670	2.43E-06	1.72E-05	4.69E-06	AKT1/CAV1/IL1B/TGFB1/TNF
GO:0006909	phagocytosis	7/32	369/18670	2.45E-06	1.73E-05	4.71E-06	ABL1/HSP90AA1/IL1B/MAPK1/SRC/TGFB1/TNF
GO:0060688	regulation of morphogenesis of a branching structure	4/32	56/18670	2.45E-06	1.73E-05	4.71E-06	ABL1/CTNNB1/TGFB1/TNF
GO:0048588	developmental cell growth	6/32	234/18670	2.51E-06	1.76E-05	4.81E-06	ABL1/CTNNB1/GSK3B/HSP90AA1/IGF1/MAPT
GO:0034116	positive regulation of heterotypic cell-cell adhesion	3/32	16/18670	2.52E-06	1.77E-05	4.82E-06	IL1B/IL10/TNF
GO:0060560	developmental growth involved in morphogenesis	6/32	235/18670	2.57E-06	1.80E-05	4.90E-06	ABL1/CTNNB1/GSK3B/HSP90AA1/MAPT/TGFB1
GO:0043525	positive regulation of neuron apoptotic process	4/32	57/18670	2.63E-06	1.84E-05	5.01E-06	FASLG/CTNNB1/JUN/TNF
GO:0019229	regulation of vasoconstriction	4/32	58/18670	2.83E-06	1.97E-05	5.36E-06	AKT1/CAV1/ICAM1/PTGS2
GO:0003158	endothelium development	5/32	132/18670	2.83E-06	1.97E-05	5.36E-06	CTNNB1/ICAM1/IL1B/KDR/TNF
GO:0050663	cytokine secretion	6/32	240/18670	2.90E-06	2.01E-05	5.49E-06	ABL1/IL1A/IL1B/IL10/SRC/TNF
GO:0002705	positive regulation of leukocyte mediated immunity	5/32	133/18670	2.94E-06	2.03E-05	5.54E-06	IL1B/IL2/IL13/TGFB1/TNF
GO:0001836	release of cytochrome c from mitochondria	4/32	59/18670	3.03E-06	2.08E-05	5.66E-06	AKT1/BCL2/IGF1/JUN
GO:0001885	endothelial cell development	4/32	59/18670	3.03E-06	2.08E-05	5.66E-06	CTNNB1/ICAM1/IL1B/TNF

GO:0033619	membrane protein proteolysis	4/32	59/18670	3.03E-06	2.08E-05	5.66E-06	IL1B/IL10/TGFB1/TNF
GO:0043388	positive regulation of DNA binding	4/32	59/18670	3.03E-06	2.08E-05	5.66E-06	PARP1/CTNNB1/IGF1/TGFB1
GO:0007006	mitochondrial membrane organization	5/32	134/18670	3.05E-06	2.08E-05	5.67E-06	BCL2/CASP8/GSK3B/HSP90AA1/SNCA
GO:0018210	peptidyl-threonine modification	5/32	134/18670	3.05E-06	2.08E-05	5.67E-06	AKT1/BCL2/GSK3B/MAPK1/TGFB1
GO:2000811	negative regulation of anoikis	3/32	17/18670	3.06E-06	2.09E-05	5.69E-06	BCL2/CAV1/SRC
GO:0007292	female gamete generation	5/32	136/18670	3.28E-06	2.23E-05	6.08E-06	BCL2/CTNNB1/IGF1/PTGS2/SRC
GO:0048872	homeostasis of number of cells	6/32	246/18670	3.35E-06	2.27E-05	6.20E-06	ABL1/AKT1/BCL2/IL2/JAK3/TGFB1
GO:0072655	establishment of protein localization to mitochondrion	5/32	137/18670	3.40E-06	2.30E-05	6.26E-06	AKT1/BCL2/CASP8/HSP90AA1/MAPT
GO:1903670	regulation of sprouting angiogenesis	5/32	137/18670	3.40E-06	2.30E-05	6.26E-06	ABL1/FGF2/IL10/KDR/PTGS2
GO:0097237	cellular response to toxic substance	6/32	247/18670	3.43E-06	2.31E-05	6.30E-06	ABL1/IL10/KDR/PTGS2/SRC/TNF
GO:0030888	regulation of B cell proliferation	4/32	61/18670	3.46E-06	2.33E-05	6.36E-06	BCL2/IL2/IL10/IL13
GO:0046631	alpha-beta T cell activation	5/32	138/18670	3.52E-06	2.36E-05	6.44E-06	ABL1/BCL2/IL2/IRF1/JAK3
GO:0014706	striated muscle tissue development	7/32	390/18670	3.53E-06	2.37E-05	6.45E-06	BCL2/CAV1/CTNNB1/FGF2/IGF1/MAPK1/TGFB1

GO:0010676	positive regulation of cellular carbohydrate metabolic process	4/32	62/18670	3.70E-06	2.46E-05	6.70E-06	AKT1/IGF1/SNCA/SRC
GO:0055025	positive regulation of cardiac muscle tissue development	4/32	62/18670	3.70E-06	2.46E-05	6.70E-06	FGF2/IGF1/MAPK1/TGFB1
GO:0070265	necrotic cell death	4/32	62/18670	3.70E-06	2.46E-05	6.70E-06	FASLG/CASP8/CAV1/TNF
GO:1904356	regulation of telomere maintenance via telomere lengthening	4/32	62/18670	3.70E-06	2.46E-05	6.70E-06	PARP1/CTNNB1/MAPK1/SRC
GO:0045637	regulation of myeloid cell differentiation	6/32	251/18670	3.76E-06	2.50E-05	6.80E-06	CASP8/CTNNB1/JUN/MYC/TGFB1/TNF
GO:0072073	kidney epithelium development	5/32	140/18670	3.78E-06	2.50E-05	6.82E-06	BCL2/CTNNB1/FGF2/MYC/TGFB1
GO:0070585	protein localization to mitochondrion	5/32	141/18670	3.91E-06	2.58E-05	7.04E-06	AKT1/BCL2/CASP8/HSP90AA1/MAPT
GO:2000027	regulation of animal organ morphogenesis	6/32	253/18670	3.94E-06	2.60E-05	7.07E-06	ABL1/BCL2/CTNNB1/MYC/TGFB1/TNF
GO:0046622	positive regulation of organ growth	4/32	63/18670	3.94E-06	2.60E-05	7.07E-06	AKT1/FGF2/IGF1/MAPK1
GO:0010675	regulation of cellular carbohydrate metabolic process	5/32	142/18670	4.05E-06	2.65E-05	7.22E-06	AKT1/GSK3B/IGF1/SNCA/SRC
GO:0034332	adherens junction organization	5/32	142/18670	4.05E-06	2.65E-05	7.22E-06	ABL1/BCL2/CTNNB1/KDR/SRC
GO:0072006	nephron development	5/32	142/18670	4.05E-06	2.65E-05	7.22E-06	BCL2/CTNNB1/FGF2/MYC/TGFB1
GO:1902107	positive regulation of leukocyte differentiation	5/32	144/18670	4.33E-06	2.83E-05	7.71E-06	CASP8/IL2/JUN/TGFB1/TNF

GO:0032729	positive regulation of interferon-gamma production	4/32	65/18670	4.47E-06	2.91E-05	7.94E-06	ABL1/IL1B/IL2/TNF
GO:0042542	response to hydrogen peroxide	5/32	146/18670	4.64E-06	3.02E-05	8.22E-06	ABL1/BCL2/IL10/JUN/SRC
GO:0050808	synapse organization	7/32	408/18670	4.75E-06	3.07E-05	8.36E-06	ABL1/CTNNB1/ERBB2/IL10/MAPT/SNCA/TNF
GO:0060537	muscle tissue development	7/32	408/18670	4.75E-06	3.07E-05	8.36E-06	BCL2/CAV1/CTNNB1/FGF2/IGF1/MAPK1/TGFB1
GO:0051785	positive regulation of nuclear division	4/32	66/18670	4.75E-06	3.07E-05	8.36E-06	IGF1/IL1A/IL1B/TGFB1
GO:0051926	negative regulation of calcium ion transport	4/32	66/18670	4.75E-06	3.07E-05	8.36E-06	BCL2/ICAM1/PTGS2/TGFB1
GO:0007517	muscle organ development	7/32	410/18670	4.90E-06	3.15E-05	8.60E-06	BCL2/CAV1/CTNNB1/FGF2/IGF1/MAPK1/TGFB1
GO:0042108	positive regulation of cytokine biosynthetic process	4/32	67/18670	5.05E-06	3.24E-05	8.84E-06	IL1A/IL1B/IRF1/TNF
GO:0002706	regulation of lymphocyte mediated immunity	5/32	149/18670	5.12E-06	3.28E-05	8.93E-06	IL1B/IL2/IL10/TGFB1/TNF
GO:0051092	positive regulation of NF-kappaB transcription factor activity	5/32	149/18670	5.12E-06	3.28E-05	8.93E-06	CAV1/ICAM1/IL1B/TGFB1/TNF
GO:0050777	negative regulation of immune response	5/32	150/18670	5.29E-06	3.38E-05	9.21E-06	IL2/IL10/JAK3/TGFB1/TNF
GO:0000187	activation of MAPK activity	5/32	152/18670	5.65E-06	3.59E-05	9.79E-06	FGF2/IGF1/IL1B/MAPK1/TNF
GO:0001890	placenta development	5/32	152/18670	5.65E-06	3.59E-05	9.79E-06	AKT1/CASP8/IL10/MAPK1/PTGS2

GO:0050729	positive regulation of inflammatory response	5/32	153/18670	5.83E-06	3.70E-05	1.01E-05	IL1B/IL2/PTGS2/SNCA/TNF
GO:1904886	beta-catenin destruction complex disassembly	3/32	21/18670	5.96E-06	3.77E-05	1.03E-05	CAV1/CTNNB1/GSK3B
GO:0007004	telomere maintenance via telomerase	4/32	70/18670	6.02E-06	3.81E-05	1.04E-05	CTNNB1/HSP90AA1/MAPK1/SRC
GO:0043542	endothelial cell migration	6/32	273/18670	6.09E-06	3.84E-05	1.05E-05	ABL1/AKT1/FGF2/KDR/PTGS2/TGFB1
GO:0043270	positive regulation of ion transport	6/32	275/18670	6.35E-06	4.00E-05	1.09E-05	ABL1/AKT1/CAV1/IL1B/IL13/SNCA
GO:0042531	positive regulation of tyrosine phosphorylation of STAT protein	4/32	71/18670	6.37E-06	4.00E-05	1.09E-05	IGF1/IL2/IL13/TNF
GO:0050805	negative regulation of synaptic transmission	4/32	72/18670	6.74E-06	4.23E-05	1.15E-05	IL1B/MAPT/PTGS2/SNCA
GO:0002312	B cell activation involved in immune response	4/32	73/18670	7.12E-06	4.42E-05	1.21E-05	ABL1/IL2/IL10/TGFB1
GO:0032945	negative regulation of mononuclear cell proliferation	4/32	73/18670	7.12E-06	4.42E-05	1.21E-05	ERBB2/IL2/IL10/TGFB1
GO:0050672	negative regulation of lymphocyte proliferation	4/32	73/18670	7.12E-06	4.42E-05	1.21E-05	ERBB2/IL2/IL10/TGFB1
GO:1901983	regulation of protein acetylation	4/32	73/18670	7.12E-06	4.42E-05	1.21E-05	GSK3B/IL1B/SNCA/TGFB1
GO:1903524	positive regulation of blood circulation	4/32	73/18670	7.12E-06	4.42E-05	1.21E-05	AKT1/CAV1/ICAM1/PTGS2
GO:0031647	regulation of protein stability	6/32	284/18670	7.64E-06	4.74E-05	1.29E-05	BCL2/HSP90AA1/IGF1/MAPK1/SNCA/SRC

GO:0050995	negative regulation of lipid catabolic process	3/32	23/18670	7.91E-06	4.89E-05	1.33E-05	AKT1/IL1B/TNF
GO:0051043	regulation of membrane protein ectodomain proteolysis	3/32	23/18670	7.91E-06	4.89E-05	1.33E-05	IL1B/IL10/TNF
GO:1903201	regulation of oxidative stress-induced cell death	4/32	75/18670	7.93E-06	4.89E-05	1.33E-05	PARP1/AKT1/CTNNB1/IL10
GO:0042310	vasoconstriction	4/32	76/18670	8.36E-06	5.15E-05	1.40E-05	AKT1/CAV1/ICAM1/PTGS2
GO:0006278	RNA-dependent DNA biosynthetic process	4/32	77/18670	8.81E-06	5.41E-05	1.47E-05	CTNNB1/HSP90AA1/MAPK1/SRC
GO:0090287	regulation of cellular response to growth factor stimulus	6/32	292/18670	8.95E-06	5.48E-05	1.49E-05	ABL1/CAV1/CTNNB1/FGF2/IL1B/TGFB1
GO:2000209	regulation of anoikis	3/32	24/18670	9.03E-06	5.53E-05	1.51E-05	BCL2/CAV1/SRC
GO:0051302	regulation of cell division	5/32	168/18670	9.19E-06	5.60E-05	1.53E-05	FGF2/IL1A/IL1B/MYC/TGFB1
GO:1990138	neuron projection extension	5/32	168/18670	9.19E-06	5.60E-05	1.53E-05	ABL1/CTNNB1/GSK3B/HSP90AA1/MAPT
GO:0000271	polysaccharide biosynthetic process	4/32	78/18670	9.27E-06	5.62E-05	1.53E-05	AKT1/GSK3B/IGF1/TGFB1
GO:0010827	regulation of glucose transmembrane transport	4/32	78/18670	9.27E-06	5.62E-05	1.53E-05	AKT1/IGF1/IL1B/TNF
GO:0070664	negative regulation of leukocyte proliferation	4/32	78/18670	9.27E-06	5.62E-05	1.53E-05	ERBB2/IL2/IL10/TGFB1
GO:0051100	negative regulation of binding	5/32	169/18670	9.46E-06	5.72E-05	1.56E-05	AKT1/CAV1/GSK3B/IL10/JUN

GO:0001818	negative regulation of cytokine production	6/32	296/18670	9.67E-06	5.84E-05	1.59E-05	IGF1/IL10/IL13/JAK3/TGFB1/TNF
GO:0001570	vasculogenesis	4/32	79/18670	9.76E-06	5.87E-05	1.60E-05	CAV1/CTNNB1/KDR/TGFB1
GO:1900034	regulation of cellular response to heat	4/32	79/18670	9.76E-06	5.87E-05	1.60E-05	GSK3B/HSP90AA1/MAPT/MAPK1
GO:0090068	positive regulation of cell cycle process	6/32	298/18670	1.00E-05	6.03E-05	1.64E-05	AKT1/CCND1/IGF1/IL1A/IL1B/TGFB1
GO:0002697	regulation of immune effector process	7/32	458/18670	1.01E-05	6.05E-05	1.65E-05	IL1B/IL2/IL10/IL13/JAK3/TGFB1/TNF
GO:0007045	cell-substrate adherens junction assembly	4/32	81/18670	1.08E-05	6.43E-05	1.75E-05	ABL1/BCL2/KDR/SRC
GO:0048041	focal adhesion assembly	4/32	81/18670	1.08E-05	6.43E-05	1.75E-05	ABL1/BCL2/KDR/SRC
GO:0051346	negative regulation of hydrolase activity	7/32	466/18670	1.13E-05	6.72E-05	1.83E-05	ABL1/AKT1/GSK3B/PTGS2/SNCA/SRC/TNF
GO:0051279	regulation of release of sequestered calcium ion into cytosol	4/32	82/18670	1.13E-05	6.72E-05	1.83E-05	ABL1/IL13/SNCA/TGFB1
GO:0090049	regulation of cell migration involved in sprouting angiogenesis	4/32	82/18670	1.13E-05	6.72E-05	1.83E-05	ABL1/FGF2/KDR/PTGS2
GO:0034114	regulation of heterotypic cell-cell adhesion	3/32	26/18670	1.16E-05	6.85E-05	1.87E-05	IL1B/IL10/TNF
GO:1903203	regulation of oxidative stress-induced neuron death	3/32	26/18670	1.16E-05	6.85E-05	1.87E-05	PARP1/CTNNB1/IL10
GO:0045913	positive regulation of carbohydrate metabolic process	4/32	83/18670	1.19E-05	7.01E-05	1.91E-05	AKT1/IGF1/SNCA/SRC

GO:0009895	negative regulation of catabolic process	6/32	308/18670	1.21E-05	7.14E-05	1.95E-05	AKT1/BCL2/IL1B/IL10/SNCA/TNF
GO:0010507	negative regulation of autophagy	4/32	84/18670	1.25E-05	7.29E-05	1.99E-05	AKT1/BCL2/IL10/SNCA
GO:0043154	negative regulation of cysteine-type endopeptidase activity involved in apoptotic process	4/32	84/18670	1.25E-05	7.29E-05	1.99E-05	AKT1/PTGS2/SNCA/SRC
GO:0046889	positive regulation of lipid biosynthetic process	4/32	84/18670	1.25E-05	7.29E-05	1.99E-05	AKT1/IL1B/PTGS2/TNF
GO:0097756	negative regulation of blood vessel diameter	4/32	84/18670	1.25E-05	7.29E-05	1.99E-05	AKT1/CAV1/ICAM1/PTGS2
GO:0050769	positive regulation of neurogenesis	7/32	474/18670	1.26E-05	7.37E-05	2.01E-05	BCL2/CTNNB1/IL1B/IL2/MAPT/TGFB1/TNF
GO:0036475	neuron death in response to oxidative stress	3/32	27/18670	1.30E-05	7.57E-05	2.06E-05	PARP1/CTNNB1/IL10
GO:0048143	astrocyte activation	3/32	27/18670	1.30E-05	7.57E-05	2.06E-05	IL1B/MAPT/TNF
GO:0034612	response to tumor necrosis factor	6/32	312/18670	1.30E-05	7.57E-05	2.06E-05	AKT1/CASP8/ICAM1/MAPK1/PTGS2/TNF
GO:0010565	regulation of cellular ketone metabolic process	5/32	181/18670	1.32E-05	7.64E-05	2.08E-05	AKT1/CAV1/IL1B/PTGS2/SNCA
GO:0051147	regulation of muscle cell differentiation	5/32	181/18670	1.32E-05	7.64E-05	2.08E-05	ABL1/BCL2/CTNNB1/IGF1/TGFB1
GO:0009416	response to light stimulus	6/32	314/18670	1.35E-05	7.81E-05	2.13E-05	PARP1/AKT1/CCND1/BCL2/MYC/PTGS2
GO:0060291	long-term synaptic potentiation	4/32	86/18670	1.37E-05	7.88E-05	2.15E-05	ABL1/GSK3B/MAPK1/SNCA

GO:0070588	calcium ion transmembrane transport	6/32	315/18670	1.38E-05	7.93E-05	2.16E-05	ABL1/FASLG/FGF2/IL13/SNCA/TGFB1
GO:0043409	negative regulation of MAPK cascade	5/32	184/18670	1.43E-05	8.14E-05	2.22E-05	ABL1/AKT1/CAV1/IL1B/MYC
GO:0050864	regulation of B cell activation	5/32	184/18670	1.43E-05	8.14E-05	2.22E-05	BCL2/IL2/IL10/IL13/TGFB1
GO:0001892	embryonic placenta development	4/32	87/18670	1.43E-05	8.14E-05	2.22E-05	AKT1/CASP8/IL10/MAPK1
GO:0006112	energy reserve metabolic process	4/32	87/18670	1.43E-05	8.14E-05	2.22E-05	AKT1/GSK3B/IGF1/MYC
GO:0014068	positive regulation of phosphatidylinositol 3-kinase signaling	4/32	87/18670	1.43E-05	8.14E-05	2.22E-05	IGF1/KDR/SRC/TNF
GO:0048477	oogenesis	4/32	87/18670	1.43E-05	8.14E-05	2.22E-05	BCL2/CTNNB1/IGF1/SRC
GO:0051781	positive regulation of cell division	4/32	87/18670	1.43E-05	8.14E-05	2.22E-05	FGF2/IL1A/IL1B/TGFB1
GO:0032770	positive regulation of monooxygenase activity	3/32	28/18670	1.46E-05	8.23E-05	2.24E-05	AKT1/IL1B/TNF
GO:1902932	positive regulation of alcohol biosynthetic process	3/32	28/18670	1.46E-05	8.23E-05	2.24E-05	IL1B/SNCA/TNF
GO:1990776	response to angiotensin	3/32	28/18670	1.46E-05	8.23E-05	2.24E-05	CAV1/PTGS2/SRC
GO:0050727	regulation of inflammatory response	7/32	485/18670	1.46E-05	8.26E-05	2.25E-05	IGF1/IL1B/IL2/IL10/PTGS2/SNCA/TNF
GO:1903708	positive regulation of hemopoiesis	5/32	185/18670	1.47E-05	8.26E-05	2.25E-05	CASP8/IL2/JUN/TGFB1/TNF

GO:1903321	negative regulation of protein modification by small protein conjugation or removal	4/32	88/18670	1.50E-05	8.43E-05	2.30E-05	ABL1/AKT1/CAV1/CTNNB1
GO:0070507	regulation of microtubule cytoskeleton organization	5/32	186/18670	1.51E-05	8.45E-05	2.30E-05	ABL1/CTNNB1/GSK3B/MAPT/SNCA
GO:0005979	regulation of glycogen biosynthetic process	3/32	29/18670	1.62E-05	9.07E-05	2.47E-05	AKT1/GSK3B/IGF1
GO:0010962	regulation of glucan biosynthetic process	3/32	29/18670	1.62E-05	9.07E-05	2.47E-05	AKT1/GSK3B/IGF1
GO:0045639	positive regulation of myeloid cell differentiation	4/32	91/18670	1.71E-05	9.56E-05	2.60E-05	CASP8/JUN/TGFB1/TNF
GO:0043112	receptor metabolic process	5/32	192/18670	1.75E-05	9.78E-05	2.67E-05	CAV1/IL10/SNCA/TGFB1/TNF
GO:2000117	negative regulation of cysteine-type endopeptidase activity	4/32	92/18670	1.79E-05	9.94E-05	2.71E-05	AKT1/PTGS2/SNCA/SRC
GO:0002377	immunoglobulin production	5/32	193/18670	1.80E-05	9.96E-05	2.72E-05	IL2/IL10/IL13/TGFB1/TNF
GO:0032743	positive regulation of interleukin-2 production	3/32	30/18670	1.80E-05	9.96E-05	2.72E-05	ABL1/IL1A/IL1B
GO:0097421	liver regeneration	3/32	30/18670	1.80E-05	9.96E-05	2.72E-05	CCND1/IL10/TGFB1
GO:1901655	cellular response to ketone	4/32	93/18670	1.86E-05	0.000103	2.81E-05	AKT1/ICAM1/SRC/TGFB1
GO:0002675	positive regulation of acute inflammatory response	3/32	31/18670	1.99E-05	0.000109	2.98E-05	IL1B/PTGS2/TNF
GO:0010762	regulation of fibroblast migration	3/32	31/18670	1.99E-05	0.000109	2.98E-05	AKT1/FGF2/TGFB1

GO:0045737	positive regulation of cyclin-dependent protein serine/threonine kinase activity	3/32	31/18670	1.99E-05	0.000109	2.98E-05	AKT1/CCND1/SRC
GO:1902895	positive regulation of pri-miRNA transcription by RNA polymerase II	3/32	31/18670	1.99E-05	0.000109	2.98E-05	IL10/JUN/TGFB1
GO:0002702	positive regulation of production of molecular mediator of immune response	4/32	95/18670	2.03E-05	0.000111	3.02E-05	IL1B/IL2/IL13/TGFB1
GO:0042102	positive regulation of T cell proliferation	4/32	95/18670	2.03E-05	0.000111	3.02E-05	IGF1/IL1B/IL2/JAK3
GO:0007179	transforming growth factor beta receptor signaling pathway	5/32	199/18670	2.08E-05	0.000114	3.10E-05	PARP1/CAV1/JUN/SRC/TGFB1
GO:0017038	protein import	5/32	199/18670	2.08E-05	0.000114	3.10E-05	AKT1/HSP90AA1/MAPK1/PTGS2/TGFB1
GO:0048010	vascular endothelial growth factor receptor signaling pathway	4/32	96/18670	2.11E-05	0.000115	3.14E-05	HSP90AA1/IL1B/KDR/SRC
GO:0038128	ERBB2 signaling pathway	3/32	32/18670	2.19E-05	0.000119	3.23E-05	ERBB2/HSP90AA1/SRC
GO:0043552	positive regulation of phosphatidylinositol 3-kinase activity	3/32	32/18670	2.19E-05	0.000119	3.23E-05	FGF2/SRC/TGFB1
GO:0045589	regulation of regulatory T cell differentiation	3/32	32/18670	2.19E-05	0.000119	3.23E-05	IL2/IRF1/TGFB1
GO:0051385	response to mineralocorticoid	3/32	32/18670	2.19E-05	0.000119	3.23E-05	PARP1/CCND1/SRC
GO:0007044	cell-substrate junction assembly	4/32	97/18670	2.20E-05	0.000119	3.23E-05	ABL1/BCL2/KDR/SRC
GO:0030316	osteoclast differentiation	4/32	97/18670	2.20E-05	0.000119	3.23E-05	CTNNB1/SRC/TGFB1/TNF

GO:0001101	response to acid chemical	6/32	343/18670	2.23E-05	0.00012	3.26E-05	AKT1/ICAM1/KDR/PTGS2/SRC/TNF
GO:0006913	nucleocytoplasmic transport	6/32	343/18670	2.23E-05	0.00012	3.26E-05	AKT1/GSK3B/IL1B/MAPK1/PTGS2/TGFB1
GO:0050848	regulation of calcium-mediated signaling	4/32	98/18670	2.29E-05	0.000123	3.34E-05	GSK3B/IGF1/MAPT/TNF
GO:2001243	negative regulation of intrinsic apoptotic signaling pathway	4/32	98/18670	2.29E-05	0.000123	3.34E-05	AKT1/BCL2/PTGS2/SRC
GO:0051169	nuclear transport	6/32	346/18670	2.34E-05	0.000125	3.40E-05	AKT1/GSK3B/IL1B/MAPK1/PTGS2/TGFB1
GO:0006473	protein acetylation	5/32	204/18670	2.35E-05	0.000125	3.40E-05	GSK3B/IL1B/MAPT/SNCA/TGFB1
GO:0034764	positive regulation of transmembrane transport	5/32	204/18670	2.35E-05	0.000125	3.40E-05	ABL1/AKT1/IGF1/IL13/SNCA
GO:0035265	organ growth	5/32	204/18670	2.35E-05	0.000125	3.40E-05	AKT1/BCL2/FGF2/IGF1/MAPK1
GO:0010039	response to iron ion	3/32	33/18670	2.41E-05	0.000127	3.47E-05	CCND1/BCL2/SNCA
GO:0045191	regulation of isotype switching	3/32	33/18670	2.41E-05	0.000127	3.47E-05	IL2/IL10/TGFB1
GO:1901890	positive regulation of cell junction assembly	3/32	33/18670	2.41E-05	0.000127	3.47E-05	ABL1/CAV1/KDR
GO:0007178	transmembrane receptor protein serine/threonine kinase signaling pathway	6/32	349/18670	2.46E-05	0.00013	3.54E-05	ABL1/PARP1/CAV1/JUN/SRC/TGFB1

GO:0002824	positive regulation of adaptive immune response based on somatic recombination of immune receptors built from immunoglobulin superfamily domains	4/32	100/18670	2.48E-05	0.000131	3.56E-05	IL1B/IL2/TGFB1/TNF
GO:0055024	regulation of cardiac muscle tissue development	4/32	100/18670	2.48E-05	0.000131	3.56E-05	FGF2/IGF1/MAPK1/TGFB1
GO:0002064	epithelial cell development	5/32	207/18670	2.52E-05	0.000132	3.60E-05	CTNNB1/GSK3B/ICAM1/IL1B/TNF
GO:0070555	response to interleukin-1	5/32	207/18670	2.52E-05	0.000132	3.60E-05	ICAM1/IL1A/IL1B/SNCA/SRC
GO:0002449	lymphocyte mediated immunity	6/32	352/18670	2.58E-05	0.000135	3.68E-05	ICAM1/IL1B/IL2/IL10/TGFB1/TNF
GO:0000018	regulation of DNA recombination	4/32	101/18670	2.58E-05	0.000135	3.68E-05	PARP1/IL2/IL10/TGFB1
GO:0006006	glucose metabolic process	5/32	209/18670	2.64E-05	0.000137	3.72E-05	AKT1/GSK3B/IGF1/SRC/TNF
GO:0032148	activation of protein kinase B activity	3/32	34/18670	2.64E-05	0.000137	3.72E-05	AKT1/IGF1/SRC
GO:0045066	regulatory T cell differentiation	3/32	34/18670	2.64E-05	0.000137	3.72E-05	IL2/IRF1/TGFB1
GO:0070884	regulation of calcineurin-NFAT signaling cascade	3/32	34/18670	2.64E-05	0.000137	3.72E-05	GSK3B/IGF1/TNF
GO:0071868	cellular response to monoamine stimulus	3/32	34/18670	2.64E-05	0.000137	3.72E-05	ABL1/MAPK1/SNCA
GO:0071870	cellular response to catecholamine stimulus	3/32	34/18670	2.64E-05	0.000137	3.72E-05	ABL1/MAPK1/SNCA

GO:0106056	regulation of calcineurin-mediated signaling	3/32	34/18670	2.64E-05	0.000137	3.72E-05	GSK3B/IGF1/TNF
GO:0031589	cell-substrate adhesion	6/32	354/18670	2.66E-05	0.000137	3.74E-05	ABL1/BCL2/CTNNB1/GSK3B/KDR/SRC
GO:0032091	negative regulation of protein binding	4/32	103/18670	2.79E-05	0.000144	3.91E-05	AKT1/CAV1/GSK3B/IL10
GO:0022412	cellular process involved in reproduction in multicellular organism	6/32	357/18670	2.79E-05	0.000144	3.91E-05	AKT1/BCL2/CTNNB1/IGF1/SRC/TGFB1
GO:0001893	maternal placenta development	3/32	35/18670	2.88E-05	0.000147	4.00E-05	AKT1/MAPK1/PTGS2
GO:0051354	negative regulation of oxidoreductase activity	3/32	35/18670	2.88E-05	0.000147	4.00E-05	CAV1/IL13/SNCA
GO:0070873	regulation of glycogen metabolic process	3/32	35/18670	2.88E-05	0.000147	4.00E-05	AKT1/GSK3B/IGF1
GO:0110111	negative regulation of animal organ morphogenesis	3/32	35/18670	2.88E-05	0.000147	4.00E-05	BCL2/CTNNB1/TNF
GO:1901030	positive regulation of mitochondrial outer membrane permeabilization involved in apoptotic signaling pathway	3/32	35/18670	2.88E-05	0.000147	4.00E-05	BCL2/CASP8/GSK3B
GO:1904031	positive regulation of cyclin-dependent protein kinase activity	3/32	35/18670	2.88E-05	0.000147	4.00E-05	AKT1/CCND1/SRC
GO:1905332	positive regulation of morphogenesis of an epithelium	3/32	35/18670	2.88E-05	0.000147	4.00E-05	ABL1/CTNNB1/TGFB1
GO:0016051	carbohydrate biosynthetic process	5/32	214/18670	2.95E-05	0.00015	4.08E-05	AKT1/GSK3B/IGF1/SNCA/TGFB1
GO:2001020	regulation of response to DNA damage stimulus	5/32	214/18670	2.95E-05	0.00015	4.08E-05	ABL1/PARP1/BCL2/MAPT/MYC

GO:0002708	positive regulation of lymphocyte mediated immunity	4/32	105/18670	3.01E-05	0.000152	4.15E-05	IL1B/IL2/TGFB1/TNF
GO:0002821	positive regulation of adaptive immune response	4/32	105/18670	3.01E-05	0.000152	4.15E-05	IL1B/IL2/TGFB1/TNF
GO:0010810	regulation of cell-substrate adhesion	5/32	215/18670	3.02E-05	0.000153	4.16E-05	ABL1/BCL2/GSK3B/KDR/SRC
GO:0002699	positive regulation of immune effector process	5/32	216/18670	3.09E-05	0.000155	4.24E-05	IL1B/IL2/IL13/TGFB1/TNF
GO:0032869	cellular response to insulin stimulus	5/32	216/18670	3.09E-05	0.000155	4.24E-05	PARP1/AKT1/GSK3B/IL1B/SRC
GO:0090218	positive regulation of lipid kinase activity	3/32	36/18670	3.14E-05	0.000158	4.30E-05	FGF2/SRC/TGFB1
GO:0090322	regulation of superoxide metabolic process	3/32	36/18670	3.14E-05	0.000158	4.30E-05	MAPT/TGFB1/TNF
GO:0019722	calcium-mediated signaling	5/32	218/18670	3.23E-05	0.000161	4.40E-05	GSK3B/IGF1/KDR/MAPT/TNF
GO:0032271	regulation of protein polymerization	5/32	218/18670	3.23E-05	0.000161	4.40E-05	ABL1/HSP90AA1/ICAM1/MAPT/SNCA
GO:0062014	negative regulation of small molecule metabolic process	4/32	107/18670	3.24E-05	0.000162	4.41E-05	PARP1/AKT1/SNCA/TGFB1
GO:0030198	extracellular matrix organization	6/32	368/18670	3.31E-05	0.000165	4.49E-05	ABL1/FGF2/ICAM1/KDR/TGFB1/TNF
GO:1904659	glucose transmembrane transport	4/32	108/18670	3.36E-05	0.000167	4.56E-05	AKT1/IGF1/IL1B/TNF
GO:0002526	acute inflammatory response	5/32	220/18670	3.37E-05	0.000168	4.57E-05	ICAM1/IL1A/IL1B/PTGS2/TNF

GO:0002714	positive regulation of B cell mediated immunity	3/32	37/18670	3.42E-05	0.000169	4.60E-05	IL2/TGFB1/TNF
GO:0002891	positive regulation of immunoglobulin mediated immune response	3/32	37/18670	3.42E-05	0.000169	4.60E-05	IL2/TGFB1/TNF
GO:0042307	positive regulation of protein import into nucleus	3/32	37/18670	3.42E-05	0.000169	4.60E-05	MAPK1/PTGS2/TGFB1
GO:0071276	cellular response to cadmium ion	3/32	37/18670	3.42E-05	0.000169	4.60E-05	AKT1/JUN/MAPK1
GO:0001701	in utero embryonic development	6/32	373/18670	3.57E-05	0.000176	4.79E-05	AKT1/CASP8/CTNNB1/IGF1/IL10/MAPK1
GO:0045444	fat cell differentiation	5/32	223/18670	3.60E-05	0.000177	4.83E-05	AKT1/CCND1/PTGS2/TGFB1/TNF
GO:0071867	response to monoamine	3/32	38/18670	3.70E-05	0.000182	4.96E-05	ABL1/MAPK1/SNCA
GO:0071869	response to catecholamine	3/32	38/18670	3.70E-05	0.000182	4.96E-05	ABL1/MAPK1/SNCA
GO:0008645	hexose transmembrane transport	4/32	112/18670	3.88E-05	0.000189	5.16E-05	AKT1/IGF1/IL1B/TNF
GO:0042303	molting cycle	4/32	112/18670	3.88E-05	0.000189	5.16E-05	BCL2/CTNNB1/PTGS2/TNF
GO:0042633	hair cycle	4/32	112/18670	3.88E-05	0.000189	5.16E-05	BCL2/CTNNB1/PTGS2/TNF
GO:1903510	mucopolysaccharide metabolic process	4/32	112/18670	3.88E-05	0.000189	5.16E-05	AKT1/FGF2/IL1B/TGFB1
GO:0010761	fibroblast migration	3/32	39/18670	4.01E-05	0.000194	5.30E-05	AKT1/FGF2/TGFB1

GO:0045911	positive regulation of DNA recombination	3/32	39/18670	4.01E-05	0.000194	5.30E-05	PARP1/IL2/TGFB1
GO:1902042	negative regulation of extrinsic apoptotic signaling pathway via death domain receptors	3/32	39/18670	4.01E-05	0.000194	5.30E-05	FASLG/CASP8/ICAM1
GO:1904591	positive regulation of protein import	3/32	39/18670	4.01E-05	0.000194	5.30E-05	MAPK1/PTGS2/TGFB1
GO:0046620	regulation of organ growth	4/32	113/18670	4.01E-05	0.000194	5.30E-05	AKT1/FGF2/IGF1/MAPK1
GO:0005976	polysaccharide metabolic process	4/32	114/18670	4.15E-05	0.000201	5.47E-05	AKT1/GSK3B/IGF1/TGFB1
GO:0015749	monosaccharide transmembrane transport	4/32	114/18670	4.15E-05	0.000201	5.47E-05	AKT1/IGF1/IL1B/TNF
GO:0042692	muscle cell differentiation	6/32	385/18670	4.26E-05	0.000205	5.58E-05	ABL1/AKT1/BCL2/CTNNB1/IGF1/TGFB1
GO:1903320	regulation of protein modification by small protein conjugation or removal	5/32	231/18670	4.26E-05	0.000205	5.58E-05	ABL1/AKT1/CAV1/CTNNB1/HSP90AA1
GO:0032612	interleukin-1 production	4/32	115/18670	4.30E-05	0.000207	5.63E-05	CASP8/IGF1/IL1B/IL10
GO:0009165	nucleotide biosynthetic process	6/32	386/18670	4.32E-05	0.000207	5.64E-05	PARP1/IGF1/MAPK1/PTGS2/SNCA/TGFB1
GO:0051281	positive regulation of release of sequestered calcium ion into cytosol	3/32	40/18670	4.33E-05	0.000207	5.64E-05	ABL1/IL13/SNCA
GO:2000008	regulation of protein localization to cell surface	3/32	40/18670	4.33E-05	0.000207	5.64E-05	AKT1/CTNNB1/TNF
GO:0034219	carbohydrate transmembrane transport	4/32	116/18670	4.45E-05	0.000213	5.79E-05	AKT1/IGF1/IL1B/TNF

GO:1901293	nucleoside phosphate biosynthetic process	6/32	390/18670	4.57E-05	0.000218	5.95E-05	PARP1/IGF1/MAPK1/PTGS2/SNCA/TGFB1
GO:0010822	positive regulation of mitochondrion organization	4/32	117/18670	4.60E-05	0.000219	5.96E-05	BCL2/CASP8/GSK3B/KDR
GO:0010906	regulation of glucose metabolic process	4/32	117/18670	4.60E-05	0.000219	5.96E-05	AKT1/GSK3B/IGF1/SRC
GO:0030890	positive regulation of B cell proliferation	3/32	41/18670	4.66E-05	0.000221	6.02E-05	BCL2/IL2/IL13
GO:0050798	activated T cell proliferation	3/32	41/18670	4.66E-05	0.000221	6.02E-05	ABL1/IGF1/IL2
GO:1902893	regulation of pri-miRNA transcription by RNA polymerase II	3/32	41/18670	4.66E-05	0.000221	6.02E-05	IL10/JUN/TGFB1
GO:0007050	cell cycle arrest	5/32	237/18670	4.81E-05	0.000227	6.18E-05	ABL1/CCND1/IRF1/MYC/TGFB1
GO:0031669	cellular response to nutrient levels	5/32	237/18670	4.81E-05	0.000227	6.18E-05	BCL2/ICAM1/JUN/MAPK1/PTGS2
GO:0032872	regulation of stress-activated MAPK cascade	5/32	237/18670	4.81E-05	0.000227	6.18E-05	AKT1/IL1B/MYC/MAPK1/TNF
GO:0010639	negative regulation of organelle organization	6/32	395/18670	4.91E-05	0.000231	6.30E-05	PARP1/AKT1/IGF1/MAPT/SNCA/SRC
GO:0070302	regulation of stress-activated protein kinase signaling cascade	5/32	239/18670	5.01E-05	0.000235	6.40E-05	AKT1/IL1B/MYC/MAPK1/TNF
GO:0002639	positive regulation of immunoglobulin production	3/32	42/18670	5.02E-05	0.000235	6.40E-05	IL2/IL13/TGFB1
GO:0006509	membrane protein ectodomain proteolysis	3/32	42/18670	5.02E-05	0.000235	6.40E-05	IL1B/IL10/TNF

GO:0010907	positive regulation of glucose metabolic process	3/32	42/18670	5.02E-05	0.000235	6.40E-05	AKT1/IGF1/SRC
GO:0038093	Fc receptor signaling pathway	5/32	241/18670	5.21E-05	0.000243	6.63E-05	ABL1/HSP90AA1/JUN/MAPK1/SRC
GO:0048675	axon extension	4/32	121/18670	5.25E-05	0.000245	6.67E-05	ABL1/GSK3B/HSP90AA1/MAPT
GO:0008631	intrinsic apoptotic signaling pathway in response to oxidative stress	3/32	43/18670	5.39E-05	0.000251	6.83E-05	PARP1/AKT1/BCL2
GO:0033173	calcineurin-NFAT signaling cascade	3/32	43/18670	5.39E-05	0.000251	6.83E-05	GSK3B/IGF1/TNF
GO:0051928	positive regulation of calcium ion transport	4/32	123/18670	5.59E-05	0.00026	7.08E-05	ABL1/CAV1/IL13/SNCA
GO:0043900	regulation of multi-organism process	6/32	405/18670	5.64E-05	0.000262	7.13E-05	BCL2/CAV1/IGF1/IL1B/JUN/TNF
GO:0014013	regulation of gliogenesis	4/32	124/18670	5.77E-05	0.000266	7.24E-05	CTNNB1/IL1B/TGFB1/TNF
GO:0014002	astrocyte development	3/32	44/18670	5.77E-05	0.000266	7.24E-05	IL1B/MAPT/TNF
GO:0030225	macrophage differentiation	3/32	44/18670	5.77E-05	0.000266	7.24E-05	PARP1/CASP8/TGFB1
GO:0045601	regulation of endothelial cell differentiation	3/32	44/18670	5.77E-05	0.000266	7.24E-05	CTNNB1/IL1B/TNF
GO:0046688	response to copper ion	3/32	44/18670	5.77E-05	0.000266	7.24E-05	ICAM1/IL1A/SNCA
GO:1901028	regulation of mitochondrial outer membrane permeabilization involved in apoptotic signaling pathway	3/32	44/18670	5.77E-05	0.000266	7.24E-05	BCL2/CASP8/GSK3B

GO:0042180	cellular ketone metabolic process	5/32	248/18670	5.97E-05	0.000274	7.46E-05	AKT1/CAV1/IL1B/PTGS2/SNCA
GO:0043543	protein acylation	5/32	248/18670	5.97E-05	0.000274	7.46E-05	GSK3B/IL1B/MAPT/SNCA/TGFB1
GO:0019318	hexose metabolic process	5/32	249/18670	6.08E-05	0.000278	7.58E-05	AKT1/GSK3B/IGF1/SRC/TNF
GO:0071560	cellular response to transforming growth factor beta stimulus	5/32	249/18670	6.08E-05	0.000278	7.58E-05	PARP1/CAV1/JUN/SRC/TGFB1
GO:0032570	response to progesterone	3/32	45/18670	6.18E-05	0.000282	7.69E-05	CAV1/SRC/TGFB1
GO:0071383	cellular response to steroid hormone stimulus	5/32	250/18670	6.20E-05	0.000283	7.70E-05	PARP1/CTNNB1/ICAM1/SRC/TGFB1
GO:0002437	inflammatory response to antigenic stimulus	3/32	46/18670	6.60E-05	0.000299	8.14E-05	ICAM1/IL10/TNF
GO:0005978	glycogen biosynthetic process	3/32	46/18670	6.60E-05	0.000299	8.14E-05	AKT1/GSK3B/IGF1
GO:0009250	glucan biosynthetic process	3/32	46/18670	6.60E-05	0.000299	8.14E-05	AKT1/GSK3B/IGF1
GO:0048599	oocyte development	3/32	46/18670	6.60E-05	0.000299	8.14E-05	BCL2/CTNNB1/IGF1
GO:0060443	mammary gland morphogenesis	3/32	46/18670	6.60E-05	0.000299	8.14E-05	CAV1/SRC/TGFB1
GO:0097720	calcineurin-mediated signaling	3/32	46/18670	6.60E-05	0.000299	8.14E-05	GSK3B/IGF1/TNF
GO:0071559	response to transforming growth factor beta	5/32	255/18670	6.81E-05	0.000307	8.38E-05	PARP1/CAV1/JUN/SRC/TGFB1

GO:0002204	somatic recombination of immunoglobulin genes involved in immune response	3/32	47/18670	7.05E-05	0.000315	8.57E-05	IL2/IL10/TGFB1
GO:0002208	somatic diversification of immunoglobulins involved in immune response	3/32	47/18670	7.05E-05	0.000315	8.57E-05	IL2/IL10/TGFB1
GO:0014911	positive regulation of smooth muscle cell migration	3/32	47/18670	7.05E-05	0.000315	8.57E-05	BCL2/IGF1/SRC
GO:0031952	regulation of protein autophosphorylation	3/32	47/18670	7.05E-05	0.000315	8.57E-05	CAV1/JUN/SRC
GO:0045190	isotype switching	3/32	47/18670	7.05E-05	0.000315	8.57E-05	IL2/IL10/TGFB1
GO:0061614	pri-miRNA transcription by RNA polymerase II	3/32	47/18670	7.05E-05	0.000315	8.57E-05	IL10/JUN/TGFB1
GO:0070231	T cell apoptotic process	3/32	47/18670	7.05E-05	0.000315	8.57E-05	AKT1/FASLG/JAK3
GO:0070849	response to epidermal growth factor	3/32	47/18670	7.05E-05	0.000315	8.57E-05	AKT1/ERBB2/MAPK1
GO:0033673	negative regulation of kinase activity	5/32	257/18670	7.06E-05	0.000315	8.58E-05	ABL1/AKT1/CAV1/IL1B/MAPT
GO:0043062	extracellular structure organization	6/32	422/18670	7.08E-05	0.000315	8.60E-05	ABL1/FGF2/ICAM1/KDR/TGFB1/TNF
GO:0030183	B cell differentiation	4/32	131/18670	7.15E-05	0.000318	8.66E-05	ABL1/BCL2/IL10/JAK3
GO:0042552	myelination	4/32	132/18670	7.36E-05	0.000326	8.90E-05	AKT1/CTNNB1/ERBB2/TGFB1
GO:0045598	regulation of fat cell differentiation	4/32	132/18670	7.36E-05	0.000326	8.90E-05	AKT1/PTGS2/TGFB1/TNF

GO:0055023	positive regulation of cardiac muscle tissue growth	3/32	48/18670	7.51E-05	0.000332	9.05E-05	FGF2/IGF1/MAPK1
GO:1903727	positive regulation of phospholipid metabolic process	3/32	48/18670	7.51E-05	0.000332	9.05E-05	FGF2/SRC/TGFB1
GO:0007281	germ cell development	5/32	262/18670	7.74E-05	0.000341	9.30E-05	AKT1/BCL2/CTNNB1/IGF1/SRC
GO:0007272	ensheathment of neurons	4/32	134/18670	7.81E-05	0.000341	9.30E-05	AKT1/CTNNB1/ERBB2/TGFB1
GO:0008366	axon ensheathment	4/32	134/18670	7.81E-05	0.000341	9.30E-05	AKT1/CTNNB1/ERBB2/TGFB1
GO:0030260	entry into host cell	4/32	134/18670	7.81E-05	0.000341	9.30E-05	CAV1/CTNNB1/ICAM1/SRC
GO:0044409	entry into host	4/32	134/18670	7.81E-05	0.000341	9.30E-05	CAV1/CTNNB1/ICAM1/SRC
GO:0048565	digestive tract development	4/32	134/18670	7.81E-05	0.000341	9.30E-05	BCL2/CTNNB1/TGFB1/TNF
GO:0051806	entry into cell of other organism involved in symbiotic interaction	4/32	134/18670	7.81E-05	0.000341	9.30E-05	CAV1/CTNNB1/ICAM1/SRC
GO:0051828	entry into other organism involved in symbiotic interaction	4/32	134/18670	7.81E-05	0.000341	9.30E-05	CAV1/CTNNB1/ICAM1/SRC
GO:0001889	liver development	4/32	135/18670	8.03E-05	0.000351	9.56E-05	CCND1/IL10/JUN/TGFB1
GO:0010718	positive regulation of epithelial to mesenchymal transition	3/32	50/18670	8.49E-05	0.000369	0.000101	CTNNB1/IL1B/TGFB1
GO:0031113	regulation of microtubule polymerization	3/32	50/18670	8.49E-05	0.000369	0.000101	ABL1/MAPT/SNCA

GO:0060425	lung morphogenesis	3/32	50/18670	8.49E-05	0.000369	0.000101	CTNNB1/MAPK1/TNF
GO:0031668	cellular response to extracellular stimulus	5/32	268/18670	8.61E-05	0.000374	0.000102	BCL2/ICAM1/JUN/MAPK1/PTGS2
GO:0061008	hepaticobiliary system development	4/32	138/18670	8.75E-05	0.00038	0.000103	CCND1/IL10/JUN/TGFB1
GO:0007548	sex differentiation	5/32	270/18670	8.92E-05	0.000386	0.000105	CCND1/BCL2/CTNNB1/ICAM1/SRC
GO:0002433	immune response-regulating cell surface receptor signaling pathway involved in phagocytosis	4/32	139/18670	9.00E-05	0.000388	0.000106	ABL1/HSP90AA1/MAPK1/SRC
GO:0038096	Fc-gamma receptor signaling pathway involved in phagocytosis	4/32	139/18670	9.00E-05	0.000388	0.000106	ABL1/HSP90AA1/MAPK1/SRC
GO:0001954	positive regulation of cell-matrix adhesion	3/32	51/18670	9.01E-05	0.000388	0.000106	ABL1/GSK3B/KDR
GO:0009994	oocyte differentiation	3/32	51/18670	9.01E-05	0.000388	0.000106	BCL2/CTNNB1/IGF1
GO:0048641	regulation of skeletal muscle tissue development	3/32	51/18670	9.01E-05	0.000388	0.000106	BCL2/CTNNB1/TGFB1
GO:0001837	epithelial to mesenchymal transition	4/32	140/18670	9.25E-05	0.000397	0.000108	CTNNB1/GSK3B/IL1B/TGFB1
GO:1903364	positive regulation of cellular protein catabolic process	4/32	140/18670	9.25E-05	0.000397	0.000108	AKT1/CAV1/GSK3B/HSP90AA1
GO:0008286	insulin receptor signaling pathway	4/32	141/18670	9.51E-05	0.000408	0.000111	AKT1/GSK3B/IL1B/SRC
GO:0016447	somatic recombination of immunoglobulin gene segments	3/32	52/18670	9.55E-05	0.000408	0.000111	IL2/IL10/TGFB1

GO:0060421	positive regulation of heart growth	3/32	52/18670	9.55E-05	0.000408	0.000111	FGF2/IGF1/MAPK1
GO:0038094	Fc-gamma receptor signaling pathway	4/32	142/18670	9.77E-05	0.000417	0.000114	ABL1/HSP90AA1/MAPK1/SRC
GO:0050871	positive regulation of B cell activation	4/32	142/18670	9.77E-05	0.000417	0.000114	BCL2/IL2/IL13/TGFB1
GO:0048285	organelle fission	6/32	449/18670	9.97E-05	0.000424	0.000116	IGF1/IL1A/IL1B/KDR/MAPT/TGFB1
GO:0006606	protein import into nucleus	4/32	143/18670	0.0001	0.000424	0.000116	AKT1/MAPK1/PTGS2/TGFB1
GO:0031056	regulation of histone modification	4/32	143/18670	0.0001	0.000424	0.000116	CTNNB1/IL1B/SNCA/TGFB1
GO:0035296	regulation of tube diameter	4/32	143/18670	0.0001	0.000424	0.000116	AKT1/CAV1/ICAM1/PTGS2
GO:0044344	cellular response to fibroblast growth factor stimulus	4/32	143/18670	0.0001	0.000424	0.000116	CTNNB1/FGF2/MAPK1/SNCA
GO:0050880	regulation of blood vessel size	4/32	143/18670	0.0001	0.000424	0.000116	AKT1/CAV1/ICAM1/PTGS2
GO:0097746	regulation of blood vessel diameter	4/32	143/18670	0.0001	0.000424	0.000116	AKT1/CAV1/ICAM1/PTGS2
GO:0035065	regulation of histone acetylation	3/32	53/18670	0.000101	0.000426	0.000116	IL1B/SNCA/TGFB1
GO:0070228	regulation of lymphocyte apoptotic process	3/32	53/18670	0.000101	0.000426	0.000116	IL2/IL10/JAK3
GO:0001822	kidney development	5/32	278/18670	0.000102	0.00043	0.000117	BCL2/CTNNB1/FGF2/MYC/TGFB1

GO:0035150	regulation of tube size	4/32	144/18670	0.000103	0.000434	0.000118	AKT1/CAV1/ICAM1/PTGS2
GO:0002431	Fc receptor mediated stimulatory signaling pathway	4/32	145/18670	0.000106	0.000444	0.000121	ABL1/HSP90AA1/MAPK1/SRC
GO:0071901	negative regulation of protein serine/threonine kinase activity	4/32	145/18670	0.000106	0.000444	0.000121	ABL1/AKT1/CAV1/IL1B
GO:0032210	regulation of telomere maintenance via telomerase	3/32	54/18670	0.000107	0.000445	0.000121	CTNNB1/MAPK1/SRC
GO:0032655	regulation of interleukin-12 production	3/32	54/18670	0.000107	0.000445	0.000121	IL10/IRF1/JAK3
GO:0032663	regulation of interleukin-2 production	3/32	54/18670	0.000107	0.000445	0.000121	ABL1/IL1A/IL1B
GO:0050994	regulation of lipid catabolic process	3/32	54/18670	0.000107	0.000445	0.000121	AKT1/IL1B/TNF
GO:0097345	mitochondrial outer membrane permeabilization	3/32	54/18670	0.000107	0.000445	0.000121	BCL2/CASP8/GSK3B
GO:0055123	digestive system development	4/32	146/18670	0.000109	0.000453	0.000123	BCL2/CTNNB1/TGFB1/TNF
GO:0051258	protein polymerization	5/32	283/18670	0.000111	0.000462	0.000126	ABL1/HSP90AA1/ICAM1/MAPT/SNCA
GO:0001541	ovarian follicle development	3/32	55/18670	0.000113	0.000465	0.000127	BCL2/ICAM1/SRC
GO:0010823	negative regulation of mitochondrion organization	3/32	55/18670	0.000113	0.000465	0.000127	AKT1/IGF1/MAPT
GO:0042306	regulation of protein import into nucleus	3/32	55/18670	0.000113	0.000465	0.000127	MAPK1/PTGS2/TGFB1

GO:0043030	regulation of macrophage activation	3/32	55/18670	0.000113	0.000465	0.000127	IL10/IL13/SNCA
GO:0043551	regulation of phosphatidylinositol 3-kinase activity	3/32	55/18670	0.000113	0.000465	0.000127	FGF2/SRC/TGFB1
GO:0048016	inositol phosphate-mediated signaling	3/32	55/18670	0.000113	0.000465	0.000127	GSK3B/IGF1/TNF
GO:0008643	carbohydrate transport	4/32	148/18670	0.000115	0.000472	0.000129	AKT1/IGF1/IL1B/TNF
GO:0015980	energy derivation by oxidation of organic compounds	5/32	285/18670	0.000115	0.000472	0.000129	AKT1/GSK3B/IGF1/MYC/SNCA
GO:0034976	response to endoplasmic reticulum stress	5/32	285/18670	0.000115	0.000472	0.000129	CCND1/BCL2/CAV1/GSK3B/JUN
GO:0044262	cellular carbohydrate metabolic process	5/32	286/18670	0.000117	0.000478	0.00013	AKT1/GSK3B/IGF1/SNCA/SRC
GO:0051403	stress-activated MAPK cascade	5/32	286/18670	0.000117	0.000478	0.00013	AKT1/IL1B/MYC/MAPK1/TNF
GO:0002381	immunoglobulin production involved in immunoglobulin mediated immune response	3/32	56/18670	0.000119	0.000484	0.000132	IL2/IL10/TGFB1
GO:0010332	response to gamma radiation	3/32	56/18670	0.000119	0.000484	0.000132	PARP1/BCL2/MYC
GO:0031295	T cell costimulation	3/32	56/18670	0.000119	0.000484	0.000132	AKT1/CAV1/SRC
GO:0032615	interleukin-12 production	3/32	56/18670	0.000119	0.000484	0.000132	IL10/IRF1/JAK3
GO:0090183	regulation of kidney development	3/32	56/18670	0.000119	0.000484	0.000132	CTNNB1/MYC/TGFB1

GO:1903202	negative regulation of oxidative stress-induced cell death	3/32	56/18670	0.000119	0.000484	0.000132	AKT1/CTNNB1/IL10
GO:0071774	response to fibroblast growth factor	4/32	150/18670	0.000121	0.00049	0.000134	CTNNB1/FGF2/MAPK1/SNCA
GO:1903169	regulation of calcium ion transmembrane transport	4/32	151/18670	0.000124	0.000502	0.000137	ABL1/IL13/SNCA/TGFB1
GO:0031294	lymphocyte costimulation	3/32	57/18670	0.000126	0.000507	0.000138	AKT1/CAV1/SRC
GO:0048008	platelet-derived growth factor receptor signaling pathway	3/32	57/18670	0.000126	0.000507	0.000138	ABL1/SNCA/SRC
GO:1900408	negative regulation of cellular response to oxidative stress	3/32	57/18670	0.000126	0.000507	0.000138	AKT1/CTNNB1/IL10
GO:0071356	cellular response to tumor necrosis factor	5/32	291/18670	0.000127	0.000511	0.000139	AKT1/CASP8/ICAM1/MAPK1/TNF
GO:0051901	positive regulation of mitochondrial depolarization	2/32	10/18670	0.000127	0.000511	0.000139	PARP1/KDR
GO:0005996	monosaccharide metabolic process	5/32	292/18670	0.000129	0.000518	0.000141	AKT1/GSK3B/IGF1/SRC/TNF
GO:0072001	renal system development	5/32	293/18670	0.000131	0.000526	0.000143	BCL2/CTNNB1/FGF2/MYC/TGFB1
GO:1902041	regulation of extrinsic apoptotic signaling pathway via death domain receptors	3/32	58/18670	0.000132	0.00053	0.000145	FASLG/CASP8/ICAM1
GO:1904589	regulation of protein import	3/32	58/18670	0.000132	0.00053	0.000145	MAPK1/PTGS2/TGFB1
GO:0050768	negative regulation of neurogenesis	5/32	295/18670	0.000135	0.000541	0.000147	CTNNB1/GSK3B/IL1B/TGFB1/TNF

GO:0050890	cognition	5/32	296/18670	0.000137	0.000549	0.00015	JUN/MAPT/MAPK1/PTGS2/TNF
GO:0032890	regulation of organic acid transport	3/32	59/18670	0.000139	0.000554	0.000151	AKT1/IL1B/SNCA
GO:0042130	negative regulation of T cell proliferation	3/32	59/18670	0.000139	0.000554	0.000151	ERBB2/IL10/TGFB1
GO:1902883	negative regulation of response to oxidative stress	3/32	59/18670	0.000139	0.000554	0.000151	AKT1/CTNNB1/IL10
GO:2000756	regulation of peptidyl-lysine acetylation	3/32	59/18670	0.000139	0.000554	0.000151	IL1B/SNCA/TGFB1
GO:1903522	regulation of blood circulation	5/32	297/18670	0.000139	0.000554	0.000151	AKT1/CAV1/ICAM1/IL2/PTGS2
GO:0046324	regulation of glucose import	3/32	60/18670	0.000146	0.000579	0.000158	AKT1/IGF1/TNF
GO:0051893	regulation of focal adhesion assembly	3/32	60/18670	0.000146	0.000579	0.000158	ABL1/KDR/SRC
GO:0090109	regulation of cell-substrate junction assembly	3/32	60/18670	0.000146	0.000579	0.000158	ABL1/KDR/SRC
GO:1902110	positive regulation of mitochondrial membrane permeability involved in apoptotic process	3/32	60/18670	0.000146	0.000579	0.000158	BCL2/CASP8/GSK3B
GO:0043467	regulation of generation of precursor metabolites and energy	4/32	158/18670	0.000148	0.000583	0.000159	AKT1/GSK3B/IGF1/SNCA
GO:0034248	regulation of cellular amide metabolic process	6/32	483/18670	0.000149	0.000585	0.00016	AKT1/ERBB2/IGF1/MAPK1/SNCA/TNF
GO:0034765	regulation of ion transmembrane transport	6/32	483/18670	0.000149	0.000585	0.00016	ABL1/AKT1/CAV1/IL13/SNCA/TGFB1

GO:0022604	regulation of cell morphogenesis	6/32	484/18670	0.00015	0.000591	0.000161	ABL1/GSK3B/ICAM1/KDR/MAPT/SRC
GO:0034113	heterotypic cell-cell adhesion	3/32	61/18670	0.000154	0.000604	0.000165	IL1B/IL10/TNF
GO:0007519	skeletal muscle tissue development	4/32	160/18670	0.000155	0.000605	0.000165	BCL2/CAV1/CTNNB1/TGFB1
GO:0030203	glycosaminoglycan metabolic process	4/32	160/18670	0.000155	0.000605	0.000165	AKT1/FGF2/IL1B/TGFB1
GO:0046661	male sex differentiation	4/32	160/18670	0.000155	0.000605	0.000165	CCND1/BCL2/CTNNB1/ICAM1
GO:0046886	positive regulation of hormone biosynthetic process	2/32	11/18670	0.000155	0.000605	0.000165	IL1B/TNF
GO:0060439	trachea morphogenesis	2/32	11/18670	0.000155	0.000605	0.000165	CTNNB1/MAPK1
GO:0071803	positive regulation of podosome assembly	2/32	11/18670	0.000155	0.000605	0.000165	SRC/TNF
GO:0002562	somatic diversification of immune receptors via germline recombination within a single locus	3/32	62/18670	0.000161	0.000627	0.000171	IL2/IL10/TGFB1
GO:0016444	somatic cell DNA recombination	3/32	62/18670	0.000161	0.000627	0.000171	IL2/IL10/TGFB1
GO:0032623	interleukin-2 production	3/32	62/18670	0.000161	0.000627	0.000171	ABL1/IL1A/IL1B
GO:1902686	mitochondrial outer membrane permeabilization involved in programmed cell death	3/32	62/18670	0.000161	0.000627	0.000171	BCL2/CASP8/GSK3B
GO:0051170	import into nucleus	4/32	163/18670	0.000166	0.000645	0.000176	AKT1/MAPK1/PTGS2/TGFB1

GO:0034394	protein localization to cell surface	3/32	63/18670	0.000169	0.000655	0.000178	AKT1/CTNNB1/TNF
GO:0046686	response to cadmium ion	3/32	63/18670	0.000169	0.000655	0.000178	AKT1/JUN/MAPK1
GO:0007088	regulation of mitotic nuclear division	4/32	164/18670	0.00017	0.000658	0.000179	IGF1/IL1A/IL1B/TGFB1
GO:0016445	somatic diversification of immunoglobulins	3/32	64/18670	0.000177	0.000681	0.000186	IL2/IL10/TGFB1
GO:0035794	positive regulation of mitochondrial membrane permeability	3/32	64/18670	0.000177	0.000681	0.000186	BCL2/CASP8/GSK3B
GO:0043550	regulation of lipid kinase activity	3/32	64/18670	0.000177	0.000681	0.000186	FGF2/SRC/TGFB1
GO:0060135	maternal process involved in female pregnancy	3/32	64/18670	0.000177	0.000681	0.000186	AKT1/MAPK1/PTGS2
GO:2000378	negative regulation of reactive oxygen species metabolic process	3/32	64/18670	0.000177	0.000681	0.000186	BCL2/CAV1/IL10
GO:0006475	internal protein amino acid acetylation	4/32	166/18670	0.000178	0.000684	0.000187	IL1B/MAPT/SNCA/TGFB1
GO:0031098	stress-activated protein kinase signaling cascade	5/32	315/18670	0.000183	0.000702	0.000191	AKT1/IL1B/MYC/MAPK1/TNF
GO:0051961	negative regulation of nervous system development	5/32	315/18670	0.000183	0.000702	0.000191	CTNNB1/GSK3B/IL1B/TGFB1/TNF
GO:0031392	regulation of prostaglandin biosynthetic process	2/32	12/18670	0.000186	0.000706	0.000192	IL1B/PTGS2
GO:0038110	interleukin-2-mediated signaling pathway	2/32	12/18670	0.000186	0.000706	0.000192	IL2/JAK3

GO:0042368	vitamin D biosynthetic process	2/32	12/18670	0.000186	0.000706	0.000192	IL1B/TNF
GO:0045080	positive regulation of chemokine biosynthetic process	2/32	12/18670	0.000186	0.000706	0.000192	IL1B/TNF
GO:0060391	positive regulation of SMAD protein signal transduction	2/32	12/18670	0.000186	0.000706	0.000192	PARP1/TGFB1
GO:1904181	positive regulation of membrane depolarization	2/32	12/18670	0.000186	0.000706	0.000192	PARP1/KDR
GO:0060538	skeletal muscle organ development	4/32	169/18670	0.000191	0.000725	0.000198	BCL2/CAV1/CTNNB1/TGFB1
GO:1902108	regulation of mitochondrial membrane permeability involved in apoptotic process	3/32	66/18670	0.000194	0.000736	0.000201	BCL2/CASP8/GSK3B
GO:1905710	positive regulation of membrane permeability	3/32	66/18670	0.000194	0.000736	0.000201	BCL2/CASP8/GSK3B
GO:0006022	aminoglycan metabolic process	4/32	170/18670	0.000196	0.000739	0.000201	AKT1/FGF2/IL1B/TGFB1
GO:1903391	regulation of adherens junction organization	3/32	67/18670	0.000203	0.000768	0.000209	ABL1/KDR/SRC
GO:0030324	lung development	4/32	172/18670	0.000204	0.000771	0.00021	CTNNB1/IL13/MAPK1/TNF
GO:0019915	lipid storage	3/32	68/18670	0.000213	0.0008	0.000218	CAV1/IL1B/TNF
GO:0046323	glucose import	3/32	68/18670	0.000213	0.0008	0.000218	AKT1/IGF1/TNF
GO:2001252	positive regulation of chromosome organization	4/32	174/18670	0.000214	0.000803	0.000219	CTNNB1/IL1B/MAPK1/TGFB1

GO:0009615	response to virus	5/32	326/18670	0.000215	0.000808	0.00022	BCL2/IL1B/IRF1/SRC/TNF
GO:0046165	alcohol biosynthetic process	4/32	175/18670	0.000218	0.000814	0.000222	FGF2/IL1B/SNCA/TNF
GO:0007183	SMAD protein complex assembly	2/32	13/18670	0.000219	0.000814	0.000222	PARP1/TGFB1
GO:0010763	positive regulation of fibroblast migration	2/32	13/18670	0.000219	0.000814	0.000222	AKT1/TGFB1
GO:0030656	regulation of vitamin metabolic process	2/32	13/18670	0.000219	0.000814	0.000222	IL1B/TNF
GO:0031953	negative regulation of protein autophosphorylation	2/32	13/18670	0.000219	0.000814	0.000222	CAV1/JUN
GO:0045086	positive regulation of interleukin-2 biosynthetic process	2/32	13/18670	0.000219	0.000814	0.000222	IL1A/IL1B
GO:0071352	cellular response to interleukin-2	2/32	13/18670	0.000219	0.000814	0.000222	IL2/JAK3
GO:0090594	inflammatory response to wounding	2/32	13/18670	0.000219	0.000814	0.000222	IL1A/TGFB1
GO:1901550	regulation of endothelial cell development	2/32	13/18670	0.000219	0.000814	0.000222	IL1B/TNF
GO:1903140	regulation of establishment of endothelial barrier	2/32	13/18670	0.000219	0.000814	0.000222	IL1B/TNF
GO:0030323	respiratory tube development	4/32	176/18670	0.000223	0.000828	0.000226	CTNNB1/IL13/MAPK1/TNF
GO:0032984	protein-containing complex disassembly	5/32	329/18670	0.000225	0.000831	0.000227	CAV1/CTNNB1/GSK3B/MYC/TNF

GO:0001655	urogenital system development	5/32	330/18670	0.000228	0.000842	0.000229	BCL2/CTNNB1/FGF2/MYC/TGFB1
GO:0010517	regulation of phospholipase activity	3/32	70/18670	0.000232	0.000853	0.000233	ABL1/FGF2/SNCA
GO:0033077	T cell differentiation in thymus	3/32	70/18670	0.000232	0.000853	0.000233	BCL2/CTNNB1/ERBB2
GO:0060395	SMAD protein signal transduction	3/32	70/18670	0.000232	0.000853	0.000233	PARP1/JUN/TGFB1
GO:0008361	regulation of cell size	4/32	179/18670	0.000238	0.000876	0.000239	ABL1/AKT1/GSK3B/MAPT
GO:2001235	positive regulation of apoptotic signaling pathway	4/32	179/18670	0.000238	0.000876	0.000239	BCL2/CASP8/CAV1/GSK3B
GO:0033692	cellular polysaccharide biosynthetic process	3/32	71/18670	0.000242	0.000886	0.000241	AKT1/GSK3B/IGF1
GO:1904427	positive regulation of calcium ion transmembrane transport	3/32	71/18670	0.000242	0.000886	0.000241	ABL1/IL13/SNCA
GO:1905330	regulation of morphogenesis of an epithelium	4/32	180/18670	0.000243	0.000892	0.000243	ABL1/CTNNB1/TGFB1/TNF
GO:0051155	positive regulation of striated muscle cell differentiation	3/32	72/18670	0.000252	0.00092	0.000251	BCL2/IGF1/TGFB1
GO:1903747	regulation of establishment of protein localization to mitochondrion	3/32	72/18670	0.000252	0.00092	0.000251	BCL2/CASP8/MAPT
GO:0030213	hyaluronan biosynthetic process	2/32	14/18670	0.000256	0.000925	0.000252	IL1B/TGFB1
GO:0035791	platelet-derived growth factor receptor-beta signaling pathway	2/32	14/18670	0.000256	0.000925	0.000252	ABL1/SRC

GO:0038166	angiotensin-activated signaling pathway	2/32	14/18670	0.000256	0.000925	0.000252	CAV1/SRC
GO:0043374	CD8-positive, alpha-beta T cell differentiation	2/32	14/18670	0.000256	0.000925	0.000252	BCL2/IRF1
GO:0045591	positive regulation of regulatory T cell differentiation	2/32	14/18670	0.000256	0.000925	0.000252	IL2/TGFB1
GO:0045651	positive regulation of macrophage differentiation	2/32	14/18670	0.000256	0.000925	0.000252	CASP8/TGFB1
GO:0070669	response to interleukin-2	2/32	14/18670	0.000256	0.000925	0.000252	IL2/JAK3
GO:1903351	cellular response to dopamine	2/32	14/18670	0.000256	0.000925	0.000252	ABL1/MAPK1
GO:2001279	regulation of unsaturated fatty acid biosynthetic process	2/32	14/18670	0.000256	0.000925	0.000252	IL1B/PTGS2
GO:0006801	superoxide metabolic process	3/32	73/18670	0.000262	0.000946	0.000258	MAPT/TGFB1/TNF
GO:0031100	animal organ regeneration	3/32	73/18670	0.000262	0.000946	0.000258	CCND1/IL10/TGFB1
GO:0043627	response to estrogen	3/32	73/18670	0.000262	0.000946	0.000258	CCND1/CAV1/MAPK1
GO:1904062	regulation of cation transmembrane transport	5/32	342/18670	0.000269	0.000967	0.000264	ABL1/CAV1/IL13/SNCA/TGFB1
GO:1902275	regulation of chromatin organization	4/32	185/18670	0.00027	0.000972	0.000265	CTNNB1/IL1B/SNCA/TGFB1
GO:0005977	glycogen metabolic process	3/32	74/18670	0.000273	0.000979	0.000267	AKT1/GSK3B/IGF1

GO:0014015	positive regulation of gliogenesis	3/32	74/18670	0.000273	0.000979	0.000267	IL1B/TGFB1/TNF
GO:0032677	regulation of interleukin-8 production	3/32	74/18670	0.000273	0.000979	0.000267	IL1B/IL10/TNF
GO:0002200	somatic diversification of immune receptors	3/32	75/18670	0.000284	0.001015	0.000277	IL2/IL10/TGFB1
GO:0006073	cellular glucan metabolic process	3/32	75/18670	0.000284	0.001015	0.000277	AKT1/GSK3B/IGF1
GO:0044042	glucan metabolic process	3/32	75/18670	0.000284	0.001015	0.000277	AKT1/GSK3B/IGF1
GO:0051783	regulation of nuclear division	4/32	188/18670	0.000287	0.001025	0.000279	IGF1/IL1A/IL1B/TGFB1
GO:0030730	sequestering of triglyceride	2/32	15/18670	0.000295	0.001042	0.000284	IL1B/TNF
GO:0032352	positive regulation of hormone metabolic process	2/32	15/18670	0.000295	0.001042	0.000284	IL1B/TNF
GO:0042362	fat-soluble vitamin biosynthetic process	2/32	15/18670	0.000295	0.001042	0.000284	IL1B/TNF
GO:0045073	regulation of chemokine biosynthetic process	2/32	15/18670	0.000295	0.001042	0.000284	IL1B/TNF
GO:0045410	positive regulation of interleukin-6 biosynthetic process	2/32	15/18670	0.000295	0.001042	0.000284	IL1B/TNF
GO:0051044	positive regulation of membrane protein ectodomain proteolysis	2/32	15/18670	0.000295	0.001042	0.000284	IL1B/TNF
GO:0071801	regulation of podosome assembly	2/32	15/18670	0.000295	0.001042	0.000284	SRC/TNF

GO:1903350	response to dopamine	2/32	15/18670	0.000295	0.001042	0.000284	ABL1/MAPK1
GO:2001028	positive regulation of endothelial cell chemotaxis	2/32	15/18670	0.000295	0.001042	0.000284	FGF2/KDR
GO:0046785	microtubule polymerization	3/32	76/18670	0.000295	0.001042	0.000284	ABL1/MAPT/SNCA
GO:0046902	regulation of mitochondrial membrane permeability	3/32	76/18670	0.000295	0.001042	0.000284	BCL2/CASP8/GSK3B
GO:0071478	cellular response to radiation	4/32	191/18670	0.000305	0.001075	0.000293	PARP1/MYC/PTGS2/TGFB1
GO:0031397	negative regulation of protein ubiquitination	3/32	77/18670	0.000307	0.00108	0.000294	ABL1/AKT1/CAV1
GO:0031016	pancreas development	3/32	78/18670	0.000319	0.00112	0.000305	AKT1/CTNNB1/GSK3B
GO:0055021	regulation of cardiac muscle tissue growth	3/32	78/18670	0.000319	0.00112	0.000305	FGF2/IGF1/MAPK1
GO:0031110	regulation of microtubule polymerization or depolymerization	3/32	79/18670	0.000331	0.001161	0.000316	ABL1/MAPT/SNCA
GO:0002739	regulation of cytokine secretion involved in immune response	2/32	16/18670	0.000336	0.001165	0.000317	IL10/TNF
GO:0030214	hyaluronan catabolic process	2/32	16/18670	0.000336	0.001165	0.000317	FGF2/TGFB1
GO:0032225	regulation of synaptic transmission, dopaminergic	2/32	16/18670	0.000336	0.001165	0.000317	PTGS2/SNCA
GO:0032695	negative regulation of interleukin-12 production	2/32	16/18670	0.000336	0.001165	0.000317	IL10/JAK3

GO:0042033	chemokine biosynthetic process	2/32	16/18670	0.000336	0.001165	0.000317	IL1B/TNF
GO:0045623	negative regulation of T-helper cell differentiation	2/32	16/18670	0.000336	0.001165	0.000317	IL2/JAK3
GO:0050755	chemokine metabolic process	2/32	16/18670	0.000336	0.001165	0.000317	IL1B/TNF
GO:0061684	chaperone-mediated autophagy	2/32	16/18670	0.000336	0.001165	0.000317	HSP90AA1/SNCA
GO:0070886	positive regulation of calcineurin-NFAT signaling cascade	2/32	16/18670	0.000336	0.001165	0.000317	IGF1/TNF
GO:0086103	G protein-coupled receptor signaling pathway involved in heart process	2/32	16/18670	0.000336	0.001165	0.000317	CAV1/SRC
GO:0106058	positive regulation of calcineurin-mediated signaling	2/32	16/18670	0.000336	0.001165	0.000317	IGF1/TNF
GO:0002685	regulation of leukocyte migration	4/32	196/18670	0.000336	0.001165	0.000317	AKT1/ICAM1/TGFB1/TNF
GO:1904950	negative regulation of establishment of protein localization	4/32	197/18670	0.000343	0.001186	0.000323	IL1B/IL10/MAPT/TNF
GO:0001654	eye development	5/32	362/18670	0.000349	0.001205	0.000328	FASLG/BCL2/CTNNB1/JUN/TGFB1
GO:0046890	regulation of lipid biosynthetic process	4/32	198/18670	0.00035	0.001206	0.000329	AKT1/IL1B/PTGS2/TNF
GO:0060541	respiratory system development	4/32	198/18670	0.00035	0.001206	0.000329	CTNNB1/IL13/MAPK1/TNF
GO:0030111	regulation of Wnt signaling pathway	5/32	363/18670	0.000353	0.001215	0.000331	ABL1/CAV1/CTNNB1/GSK3B/SRC

GO:0045861	negative regulation of proteolysis	5/32	363/18670	0.000353	0.001215	0.000331	AKT1/IL10/PTGS2/SNCA/SRC
GO:0032436	positive regulation of proteasomal ubiquitin-dependent protein catabolic process	3/32	81/18670	0.000356	0.001222	0.000333	AKT1/CAV1/GSK3B
GO:0110110	positive regulation of animal organ morphogenesis	3/32	81/18670	0.000356	0.001222	0.000333	CTNNB1/MYC/TGFB1
GO:1902930	regulation of alcohol biosynthetic process	3/32	81/18670	0.000356	0.001222	0.000333	IL1B/SNCA/TNF
GO:0006066	alcohol metabolic process	5/32	364/18670	0.000358	0.001226	0.000334	FGF2/IGF1/IL1B/SNCA/TNF
GO:0150063	visual system development	5/32	366/18670	0.000367	0.001255	0.000342	FASLG/BCL2/CTNNB1/JUN/TGFB1
GO:0030279	negative regulation of ossification	3/32	82/18670	0.000369	0.001259	0.000343	BCL2/TGFB1/TNF
GO:0032637	interleukin-8 production	3/32	82/18670	0.000369	0.001259	0.000343	IL1B/IL10/TNF
GO:0032642	regulation of chemokine production	3/32	82/18670	0.000369	0.001259	0.000343	IL1B/IL10/TNF
GO:0034644	cellular response to UV	3/32	82/18670	0.000369	0.001259	0.000343	PARP1/MYC/PTGS2
GO:0032740	positive regulation of interleukin-17 production	2/32	17/18670	0.000381	0.001291	0.000352	IL2/TGFB1
GO:0045725	positive regulation of glycogen biosynthetic process	2/32	17/18670	0.000381	0.001291	0.000352	AKT1/IGF1
GO:1904996	positive regulation of leukocyte adhesion to vascular endothelial cell	2/32	17/18670	0.000381	0.001291	0.000352	ICAM1/TNF

GO:1905331	negative regulation of morphogenesis of an epithelium	2/32	17/18670	0.000381	0.001291	0.000352	CTNNB1/TNF
GO:2000641	regulation of early endosome to late endosome transport	2/32	17/18670	0.000381	0.001291	0.000352	MAPK1/SRC
GO:2000106	regulation of leukocyte apoptotic process	3/32	83/18670	0.000383	0.001296	0.000353	IL2/IL10/JAK3
GO:0032535	regulation of cellular component size	5/32	370/18670	0.000386	0.001304	0.000355	ABL1/AKT1/GSK3B/ICAM1/MAPT
GO:0048880	sensory system development	5/32	371/18670	0.00039	0.001319	0.000359	FASLG/BCL2/CTNNB1/JUN/TGFB1
GO:0031396	regulation of protein ubiquitination	4/32	204/18670	0.000392	0.001322	0.00036	ABL1/AKT1/CAV1/HSP90AA1
GO:0014910	regulation of smooth muscle cell migration	3/32	84/18670	0.000397	0.001334	0.000364	BCL2/IGF1/SRC
GO:0045445	myoblast differentiation	3/32	84/18670	0.000397	0.001334	0.000364	IGF1/TGFB1/TNF
GO:0060420	regulation of heart growth	3/32	84/18670	0.000397	0.001334	0.000364	FGF2/IGF1/MAPK1
GO:0001942	hair follicle development	3/32	86/18670	0.000425	0.001424	0.000388	BCL2/CTNNB1/TNF
GO:0034637	cellular carbohydrate biosynthetic process	3/32	86/18670	0.000425	0.001424	0.000388	AKT1/GSK3B/IGF1
GO:0070542	response to fatty acid	3/32	86/18670	0.000425	0.001424	0.000388	AKT1/PTGS2/SRC
GO:0090559	regulation of membrane permeability	3/32	86/18670	0.000425	0.001424	0.000388	BCL2/CASP8/GSK3B

GO:0002902	regulation of B cell apoptotic process	2/32	18/18670	0.000428	0.001428	0.000389	IL2/IL10
GO:0051023	regulation of immunoglobulin secretion	2/32	18/18670	0.000428	0.001428	0.000389	IL2/TNF
GO:0070875	positive regulation of glycogen metabolic process	2/32	18/18670	0.000428	0.001428	0.000389	AKT1/IGF1
GO:0150078	positive regulation of neuroinflammatory response	2/32	18/18670	0.000428	0.001428	0.000389	IL1B/TNF
GO:0035303	regulation of dephosphorylation	4/32	209/18670	0.000429	0.001429	0.000389	GSK3B/SRC/TGFB1/TNF
GO:0071229	cellular response to acid chemical	4/32	209/18670	0.000429	0.001429	0.000389	AKT1/KDR/SRC/TNF
GO:1903531	negative regulation of secretion by cell	4/32	211/18670	0.000445	0.001479	0.000403	IL1B/IL10/SNCA/TNF
GO:0006631	fatty acid metabolic process	5/32	383/18670	0.000452	0.0015	0.000409	AKT1/CAV1/IL1B/PTGS2/SNCA
GO:0022404	molting cycle process	3/32	88/18670	0.000455	0.001503	0.000409	BCL2/CTNNB1/TNF
GO:0022405	hair cycle process	3/32	88/18670	0.000455	0.001503	0.000409	BCL2/CTNNB1/TNF
GO:0034103	regulation of tissue remodeling	3/32	88/18670	0.000455	0.001503	0.000409	IL2/SRC/TGFB1
GO:0098773	skin epidermis development	3/32	88/18670	0.000455	0.001503	0.000409	BCL2/CTNNB1/TNF
GO:1903725	regulation of phospholipid metabolic process	3/32	88/18670	0.000455	0.001503	0.000409	FGF2/SRC/TGFB1

GO:0032602	chemokine production	3/32	89/18670	0.00047	0.001552	0.000423	IL1B/IL10/TNF
GO:0002544	chronic inflammatory response	2/32	19/18670	0.000478	0.001557	0.000424	IL10/TNF
GO:0002922	positive regulation of humoral immune response	2/32	19/18670	0.000478	0.001557	0.000424	IL1B/TNF
GO:0010523	negative regulation of calcium ion transport into cytosol	2/32	19/18670	0.000478	0.001557	0.000424	BCL2/TGFB1
GO:0032026	response to magnesium ion	2/32	19/18670	0.000478	0.001557	0.000424	CCND1/SNCA
GO:0032930	positive regulation of superoxide anion generation	2/32	19/18670	0.000478	0.001557	0.000424	MAPT/TGFB1
GO:0034138	toll-like receptor 3 signaling pathway	2/32	19/18670	0.000478	0.001557	0.000424	CASP8/CAV1
GO:0045076	regulation of interleukin-2 biosynthetic process	2/32	19/18670	0.000478	0.001557	0.000424	IL1A/IL1B
GO:0060252	positive regulation of glial cell proliferation	2/32	19/18670	0.000478	0.001557	0.000424	IL1B/TNF
GO:0060438	trachea development	2/32	19/18670	0.000478	0.001557	0.000424	CTNNB1/MAPK1
GO:0060716	labyrinthine layer blood vessel development	2/32	19/18670	0.000478	0.001557	0.000424	AKT1/MAPK1
GO:0071800	podosome assembly	2/32	19/18670	0.000478	0.001557	0.000424	SRC/TNF
GO:0090201	negative regulation of release of cytochrome c from mitochondria	2/32	19/18670	0.000478	0.001557	0.000424	AKT1/IGF1

GO:2000010	positive regulation of protein localization to cell surface	2/32	19/18670	0.000478	0.001557	0.000424	AKT1/TNF
GO:0001656	metanephros development	3/32	90/18670	0.000486	0.001572	0.000429	BCL2/CTNNB1/MYC
GO:0010717	regulation of epithelial to mesenchymal transition	3/32	90/18670	0.000486	0.001572	0.000429	CTNNB1/IL1B/TGFB1
GO:0030641	regulation of cellular pH	3/32	90/18670	0.000486	0.001572	0.000429	FASLG/BCL2/MAPK1
GO:0031058	positive regulation of histone modification	3/32	90/18670	0.000486	0.001572	0.000429	CTNNB1/IL1B/TGFB1
GO:0045778	positive regulation of ossification	3/32	90/18670	0.000486	0.001572	0.000429	CTNNB1/IGF1/TGFB1
GO:0046849	bone remodeling	3/32	90/18670	0.000486	0.001572	0.000429	CTNNB1/SRC/TGFB1
GO:0008406	gonad development	4/32	217/18670	0.000494	0.0016	0.000436	CCND1/BCL2/ICAM1/SRC
GO:0014909	smooth muscle cell migration	3/32	91/18670	0.000502	0.001619	0.000441	BCL2/IGF1/SRC
GO:1901992	positive regulation of mitotic cell cycle phase transition	3/32	91/18670	0.000502	0.001619	0.000441	AKT1/CCND1/TGFB1
GO:0016064	immunoglobulin mediated immune response	4/32	218/18670	0.000503	0.001621	0.000442	IL2/IL10/TGFB1/TNF
GO:0050807	regulation of synapse organization	4/32	218/18670	0.000503	0.001621	0.000442	ABL1/IL10/SNCA/TNF
GO:0007584	response to nutrient	4/32	219/18670	0.000512	0.001648	0.000449	CCND1/IL1B/PTGS2/TGFB1

GO:0045833	negative regulation of lipid metabolic process	3/32	92/18670	0.000518	0.001665	0.000454	AKT1/IL1B/TNF
GO:0019724	B cell mediated immunity	4/32	221/18670	0.00053	0.001687	0.00046	IL2/IL10/TGFB1/TNF
GO:0002374	cytokine secretion involved in immune response	2/32	20/18670	0.00053	0.001687	0.00046	IL10/TNF
GO:0010560	positive regulation of glycoprotein biosynthetic process	2/32	20/18670	0.00053	0.001687	0.00046	CTNNB1/IGF1
GO:0032769	negative regulation of monooxygenase activity	2/32	20/18670	0.00053	0.001687	0.00046	CAV1/SNCA
GO:0043371	negative regulation of CD4-positive, alpha-beta T cell differentiation	2/32	20/18670	0.00053	0.001687	0.00046	IL2/JAK3
GO:0045019	negative regulation of nitric oxide biosynthetic process	2/32	20/18670	0.00053	0.001687	0.00046	CAV1/IL10
GO:0045655	regulation of monocyte differentiation	2/32	20/18670	0.00053	0.001687	0.00046	JUN/MYC
GO:0051797	regulation of hair follicle development	2/32	20/18670	0.00053	0.001687	0.00046	CTNNB1/TNF
GO:0097709	connective tissue replacement	2/32	20/18670	0.00053	0.001687	0.00046	IL1A/TGFB1
GO:1903204	negative regulation of oxidative stress-induced neuron death	2/32	20/18670	0.00053	0.001687	0.00046	CTNNB1/IL10
GO:1904406	negative regulation of nitric oxide metabolic process	2/32	20/18670	0.00053	0.001687	0.00046	CAV1/IL10
GO:0032092	positive regulation of protein binding	3/32	93/18670	0.000534	0.001693	0.000461	ABL1/CAV1/GSK3B

GO:0033273	response to vitamin	3/32	93/18670	0.000534	0.001693	0.000461	CCND1/PTGS2/TGFB1
GO:0046634	regulation of alpha-beta T cell activation	3/32	93/18670	0.000534	0.001693	0.000461	IL2/IRF1/JAK3
GO:1901184	regulation of ERBB signaling pathway	3/32	93/18670	0.000534	0.001693	0.000461	AKT1/FASLG/ERBB2
GO:0043903	regulation of symbiosis, encompassing mutualism through parasitism	4/32	222/18670	0.000539	0.001705	0.000465	BCL2/CAV1/JUN/TNF
GO:0045137	development of primary sexual characteristics	4/32	223/18670	0.000548	0.001732	0.000472	CCND1/BCL2/ICAM1/SRC
GO:0001649	osteoblast differentiation	4/32	225/18670	0.000567	0.00179	0.000488	AKT1/CTNNB1/IGF1/TNF
GO:0044070	regulation of anion transport	3/32	95/18670	0.000569	0.001791	0.000488	AKT1/IL1B/SNCA
GO:1903076	regulation of protein localization to plasma membrane	3/32	95/18670	0.000569	0.001791	0.000488	AKT1/TGFB1/TNF
GO:2000060	positive regulation of ubiquitin-dependent protein catabolic process	3/32	95/18670	0.000569	0.001791	0.000488	AKT1/CAV1/GSK3B
GO:0010829	negative regulation of glucose transmembrane transport	2/32	21/18670	0.000586	0.001833	0.000499	IL1B/TNF
GO:0010893	positive regulation of steroid biosynthetic process	2/32	21/18670	0.000586	0.001833	0.000499	IL1B/TNF
GO:0046827	positive regulation of protein export from nucleus	2/32	21/18670	0.000586	0.001833	0.000499	GSK3B/IL1B
GO:0071498	cellular response to fluid shear stress	2/32	21/18670	0.000586	0.001833	0.000499	PTGS2/SRC

GO:0050803	regulation of synapse structure or activity	4/32	227/18670	0.000586	0.001833	0.000499	ABL1/IL10/SNCA/TNF
GO:0008585	female gonad development	3/32	96/18670	0.000586	0.001833	0.000499	BCL2/ICAM1/SRC
GO:0030516	regulation of axon extension	3/32	96/18670	0.000586	0.001833	0.000499	ABL1/GSK3B/MAPT
GO:0097327	response to antineoplastic agent	3/32	96/18670	0.000586	0.001833	0.000499	CTNNB1/ICAM1/TGFB1
GO:0048709	oligodendrocyte differentiation	3/32	97/18670	0.000604	0.001887	0.000514	CTNNB1/ERBB2/TGFB1
GO:0009743	response to carbohydrate	4/32	230/18670	0.000615	0.001919	0.000523	ICAM1/IL1B/PTGS2/TGFB1
GO:0006885	regulation of pH	3/32	98/18670	0.000623	0.001937	0.000528	FASLG/BCL2/MAPK1
GO:0050764	regulation of phagocytosis	3/32	98/18670	0.000623	0.001937	0.000528	IL1B/TGFB1/TNF
GO:0060191	regulation of lipase activity	3/32	98/18670	0.000623	0.001937	0.000528	ABL1/FGF2/SNCA
GO:0070301	cellular response to hydrogen peroxide	3/32	99/18670	0.000642	0.001982	0.00054	ABL1/IL10/SRC
GO:0032928	regulation of superoxide anion generation	2/32	22/18670	0.000644	0.001982	0.00054	MAPT/TGFB1
GO:0042094	interleukin-2 biosynthetic process	2/32	22/18670	0.000644	0.001982	0.00054	IL1A/IL1B
GO:0042359	vitamin D metabolic process	2/32	22/18670	0.000644	0.001982	0.00054	IL1B/TNF

GO:0045649	regulation of macrophage differentiation	2/32	22/18670	0.000644	0.001982	0.00054	CASP8/TGFB1
GO:0045723	positive regulation of fatty acid biosynthetic process	2/32	22/18670	0.000644	0.001982	0.00054	IL1B/PTGS2
GO:0048305	immunoglobulin secretion	2/32	22/18670	0.000644	0.001982	0.00054	IL2/TNF
GO:0051000	positive regulation of nitric-oxide synthase activity	2/32	22/18670	0.000644	0.001982	0.00054	AKT1/TNF
GO:0060390	regulation of SMAD protein signal transduction	2/32	22/18670	0.000644	0.001982	0.00054	PARP1/TGFB1
GO:1901984	negative regulation of protein acetylation	2/32	22/18670	0.000644	0.001982	0.00054	GSK3B/SNCA
GO:0048738	cardiac muscle tissue development	4/32	233/18670	0.000646	0.001988	0.000542	FGF2/IGF1/MAPK1/TGFB1
GO:0060079	excitatory postsynaptic potential	3/32	100/18670	0.000661	0.002031	0.000553	AKT1/GSK3B/SNCA
GO:0006469	negative regulation of protein kinase activity	4/32	235/18670	0.000667	0.002048	0.000558	ABL1/AKT1/CAV1/IL1B
GO:0032611	interleukin-1 beta production	3/32	101/18670	0.00068	0.002084	0.000568	CASP8/IGF1/IL1B
GO:0046545	development of primary female sexual characteristics	3/32	101/18670	0.00068	0.002084	0.000568	BCL2/ICAM1/SRC
GO:0051048	negative regulation of secretion	4/32	238/18670	0.000699	0.002141	0.000583	IL1B/IL10/SNCA/TNF
GO:0000079	regulation of cyclin-dependent protein serine/threonine kinase activity	3/32	102/18670	0.0007	0.002141	0.000583	AKT1/CCND1/SRC

GO:0036037	CD8-positive, alpha-beta T cell activation	2/32	23/18670	0.000704	0.002144	0.000584	BCL2/IRF1
GO:0060479	lung cell differentiation	2/32	23/18670	0.000704	0.002144	0.000584	CTNNB1/IL13
GO:0072215	regulation of metanephros development	2/32	23/18670	0.000704	0.002144	0.000584	CTNNB1/MYC
GO:0090140	regulation of mitochondrial fission	2/32	23/18670	0.000704	0.002144	0.000584	KDR/MAPT
GO:1901889	negative regulation of cell junction assembly	2/32	23/18670	0.000704	0.002144	0.000584	SRC/TNF
GO:0032652	regulation of interleukin-1 production	3/32	103/18670	0.00072	0.002184	0.000595	CASP8/IGF1/IL10
GO:0034766	negative regulation of ion transmembrane transport	3/32	103/18670	0.00072	0.002184	0.000595	AKT1/CAV1/TGFB1
GO:0044264	cellular polysaccharide metabolic process	3/32	103/18670	0.00072	0.002184	0.000595	AKT1/GSK3B/IGF1
GO:1905269	positive regulation of chromatin organization	3/32	103/18670	0.00072	0.002184	0.000595	CTNNB1/IL1B/TGFB1
GO:0090092	regulation of transmembrane receptor protein serine/threonine kinase signaling pathway	4/32	241/18670	0.000733	0.002221	0.000605	ABL1/PARP1/CAV1/TGFB1
GO:0008630	intrinsic apoptotic signaling pathway in response to DNA damage	3/32	104/18670	0.000741	0.002236	0.000609	ABL1/BCL2/TNF
GO:0014812	muscle cell migration	3/32	104/18670	0.000741	0.002236	0.000609	BCL2/IGF1/SRC
GO:0019233	sensory perception of pain	3/32	104/18670	0.000741	0.002236	0.000609	IL10/MAPK1/PTGS2

GO:1901800	positive regulation of proteasomal protein catabolic process	3/32	104/18670	0.000741	0.002236	0.000609	AKT1/CAV1/GSK3B
GO:0045185	maintenance of protein location	3/32	105/18670	0.000761	0.002286	0.000623	AKT1/CAV1/IL10
GO:0055017	cardiac muscle tissue growth	3/32	105/18670	0.000761	0.002286	0.000623	FGF2/IGF1/MAPK1
GO:0001783	B cell apoptotic process	2/32	24/18670	0.000767	0.002286	0.000623	IL2/IL10
GO:0002861	regulation of inflammatory response to antigenic stimulus	2/32	24/18670	0.000767	0.002286	0.000623	IL10/TNF
GO:0009110	vitamin biosynthetic process	2/32	24/18670	0.000767	0.002286	0.000623	IL1B/TNF
GO:0010288	response to lead ion	2/32	24/18670	0.000767	0.002286	0.000623	MAPT/PTGS2
GO:0010640	regulation of platelet-derived growth factor receptor signaling pathway	2/32	24/18670	0.000767	0.002286	0.000623	SNCA/SRC
GO:0042104	positive regulation of activated T cell proliferation	2/32	24/18670	0.000767	0.002286	0.000623	IGF1/IL2
GO:0046639	negative regulation of alpha-beta T cell differentiation	2/32	24/18670	0.000767	0.002286	0.000623	IL2/JAK3
GO:0046697	decidualization	2/32	24/18670	0.000767	0.002286	0.000623	MAPK1/PTGS2
GO:0071677	positive regulation of mononuclear cell migration	2/32	24/18670	0.000767	0.002286	0.000623	TGFB1/TNF
GO:1903020	positive regulation of glycoprotein metabolic process	2/32	24/18670	0.000767	0.002286	0.000623	CTNNB1/IGF1

GO:1903649	regulation of cytoplasmic transport	2/32	24/18670	0.000767	0.002286	0.000623	MAPK1/SRC
GO:2001026	regulation of endothelial cell chemotaxis	2/32	24/18670	0.000767	0.002286	0.000623	FGF2/KDR
GO:0002286	T cell activation involved in immune response	3/32	106/18670	0.000783	0.002326	0.000634	ICAM1/IL2/JAK3
GO:1901989	positive regulation of cell cycle phase transition	3/32	106/18670	0.000783	0.002326	0.000634	AKT1/CCND1/TGFB1
GO:1904029	regulation of cyclin-dependent protein kinase activity	3/32	106/18670	0.000783	0.002326	0.000634	AKT1/CCND1/SRC
GO:1903362	regulation of cellular protein catabolic process	4/32	247/18670	0.000803	0.002385	0.00065	AKT1/CAV1/GSK3B/HSP90AA1
GO:0030004	cellular monovalent inorganic cation homeostasis	3/32	108/18670	0.000826	0.002449	0.000667	FASLG/BCL2/MAPK1
GO:0099565	chemical synaptic transmission, postsynaptic	3/32	108/18670	0.000826	0.002449	0.000667	AKT1/GSK3B/SNCA
GO:0002053	positive regulation of mesenchymal cell proliferation	2/32	25/18670	0.000833	0.002452	0.000668	CTNNB1/MYC
GO:0031069	hair follicle morphogenesis	2/32	25/18670	0.000833	0.002452	0.000668	BCL2/CTNNB1
GO:0045662	negative regulation of myoblast differentiation	2/32	25/18670	0.000833	0.002452	0.000668	TGFB1/TNF
GO:0045830	positive regulation of isotype switching	2/32	25/18670	0.000833	0.002452	0.000668	IL2/TGFB1
GO:0051894	positive regulation of focal adhesion assembly	2/32	25/18670	0.000833	0.002452	0.000668	ABL1/KDR

GO:0060444	branching involved in mammary gland duct morphogenesis	2/32	25/18670	0.000833	0.002452	0.000668	SRC/TGFB1
GO:1904385	cellular response to angiotensin	2/32	25/18670	0.000833	0.002452	0.000668	CAV1/SRC
GO:0019932	second-messenger-mediated signaling	5/32	439/18670	0.000838	0.002464	0.000671	GSK3B/IGF1/KDR/MAPT/TNF
GO:0010951	negative regulation of endopeptidase activity	4/32	250/18670	0.00084	0.00247	0.000673	AKT1/PTGS2/SNCA/SRC
GO:0061387	regulation of extent of cell growth	3/32	110/18670	0.000872	0.002559	0.000697	ABL1/GSK3B/MAPT
GO:1901990	regulation of mitotic cell cycle phase transition	5/32	444/18670	0.000881	0.002585	0.000705	AKT1/CCND1/BCL2/HSP90AA1/TGFB1
GO:0032800	receptor biosynthetic process	2/32	26/18670	0.000902	0.00263	0.000717	IL10/TNF
GO:0060544	regulation of necroptotic process	2/32	26/18670	0.000902	0.00263	0.000717	CASP8/CAV1
GO:0060706	cell differentiation involved in embryonic placenta development	2/32	26/18670	0.000902	0.00263	0.000717	AKT1/CASP8
GO:1900739	regulation of protein insertion into mitochondrial membrane involved in apoptotic signaling pathway	2/32	26/18670	0.000902	0.00263	0.000717	BCL2/CASP8
GO:1900740	positive regulation of protein insertion into mitochondrial membrane involved in apoptotic signaling pathway	2/32	26/18670	0.000902	0.00263	0.000717	BCL2/CASP8
GO:1903579	negative regulation of ATP metabolic process	2/32	26/18670	0.000902	0.00263	0.000717	PARP1/SNCA
GO:0006839	mitochondrial transport	4/32	256/18670	0.000918	0.002659	0.000725	BCL2/CASP8/GSK3B/HSP90AA1

GO:0007611	learning or memory	4/32	256/18670	0.000918	0.002659	0.000725	JUN/MAPT/MAPK1/PTGS2
GO:0090596	sensory organ morphogenesis	4/32	256/18670	0.000918	0.002659	0.000725	FASLG/BCL2/CTNNB1/MAPK1
GO:0002065	columnar/cuboidal epithelial cell differentiation	3/32	112/18670	0.000918	0.002659	0.000725	ABL1/GSK3B/IL13
GO:0031109	microtubule polymerization or depolymerization	3/32	112/18670	0.000918	0.002659	0.000725	ABL1/MAPT/SNCA
GO:0051817	modification of morphology or physiology of other organism involved in symbiotic interaction	3/32	112/18670	0.000918	0.002659	0.000725	CASP8/JUN/TGFB1
GO:0060419	heart growth	3/32	112/18670	0.000918	0.002659	0.000725	FGF2/IGF1/MAPK1
GO:1903828	negative regulation of cellular protein localization	3/32	112/18670	0.000918	0.002659	0.000725	GSK3B/MAPT/TGFB1
GO:0090257	regulation of muscle system process	4/32	259/18670	0.000959	0.002774	0.000756	PARP1/CAV1/IGF1/PTGS2
GO:0008543	fibroblast growth factor receptor signaling pathway	3/32	114/18670	0.000967	0.002789	0.00076	CTNNB1/FGF2/MAPK1
GO:0030282	bone mineralization	3/32	114/18670	0.000967	0.002789	0.00076	IGF1/PTGS2/TGFB1
GO:0002825	regulation of T-helper 1 type immune response	2/32	27/18670	0.000973	0.002789	0.00076	IL1B/JAK3
GO:0043032	positive regulation of macrophage activation	2/32	27/18670	0.000973	0.002789	0.00076	IL10/IL13
GO:0046885	regulation of hormone biosynthetic process	2/32	27/18670	0.000973	0.002789	0.00076	IL1B/TNF

GO:0048643	positive regulation of skeletal muscle tissue development	2/32	27/18670	0.000973	0.002789	0.00076	BCL2/CTNNB1
GO:1902175	regulation of oxidative stress-induced intrinsic apoptotic signaling pathway	2/32	27/18670	0.000973	0.002789	0.00076	PARP1/AKT1
GO:1904357	negative regulation of telomere maintenance via telomere lengthening	2/32	27/18670	0.000973	0.002789	0.00076	PARP1/SRC
GO:2000144	positive regulation of DNA-templated transcription, initiation	2/32	27/18670	0.000973	0.002789	0.00076	CTNNB1/JUN
GO:2000191	regulation of fatty acid transport	2/32	27/18670	0.000973	0.002789	0.00076	AKT1/IL1B
GO:0046660	female sex differentiation	3/32	115/18670	0.000991	0.002838	0.000773	BCL2/ICAM1/SRC
GO:1904375	regulation of protein localization to cell periphery	3/32	115/18670	0.000991	0.002838	0.000773	AKT1/TGFB1/TNF
GO:0010466	negative regulation of peptidase activity	4/32	262/18670	0.001	0.002861	0.00078	AKT1/PTGS2/SNCA/SRC
GO:0007569	cell aging	3/32	116/18670	0.001016	0.002904	0.000791	ABL1/BCL2/ICAM1
GO:0031330	negative regulation of cellular catabolic process	4/32	264/18670	0.001029	0.002935	0.0008	AKT1/BCL2/IL10/SNCA
GO:0140014	mitotic nuclear division	4/32	264/18670	0.001029	0.002935	0.0008	IGF1/IL1A/IL1B/TGFB1
GO:0051153	regulation of striated muscle cell differentiation	3/32	117/18670	0.001042	0.002953	0.000805	BCL2/IGF1/TGFB1
GO:1900371	regulation of purine nucleotide biosynthetic process	3/32	117/18670	0.001042	0.002953	0.000805	PARP1/IGF1/SNCA

GO:0001516	prostaglandin biosynthetic process	2/32	28/18670	0.001046	0.002953	0.000805	IL1B/PTGS2
GO:0001773	myeloid dendritic cell activation	2/32	28/18670	0.001046	0.002953	0.000805	IL10/TGFB1
GO:0033598	mammary gland epithelial cell proliferation	2/32	28/18670	0.001046	0.002953	0.000805	CCND1/MAPK1
GO:0033688	regulation of osteoblast proliferation	2/32	28/18670	0.001046	0.002953	0.000805	ABL1/BCL2
GO:0042634	regulation of hair cycle	2/32	28/18670	0.001046	0.002953	0.000805	CTNNB1/TNF
GO:0045408	regulation of interleukin-6 biosynthetic process	2/32	28/18670	0.001046	0.002953	0.000805	IL1B/TNF
GO:0046457	prostanoid biosynthetic process	2/32	28/18670	0.001046	0.002953	0.000805	IL1B/PTGS2
GO:1900543	negative regulation of purine nucleotide metabolic process	2/32	28/18670	0.001046	0.002953	0.000805	PARP1/SNCA
GO:1902003	regulation of amyloid-beta formation	2/32	28/18670	0.001046	0.002953	0.000805	IGF1/TNF
GO:1904994	regulation of leukocyte adhesion to vascular endothelial cell	2/32	28/18670	0.001046	0.002953	0.000805	ICAM1/TNF
GO:0030808	regulation of nucleotide biosynthetic process	3/32	118/18670	0.001068	0.003011	0.000821	PARP1/IGF1/SNCA
GO:0003012	muscle system process	5/32	465/18670	0.001084	0.003053	0.000832	PARP1/CAV1/IGF1/IL1B/PTGS2
GO:0072089	stem cell proliferation	3/32	120/18670	0.001121	0.003127	0.000852	CTNNB1/FGF2/TGFB1

GO:1903052	positive regulation of proteolysis involved in cellular protein catabolic process	3/32	120/18670	0.001121	0.003127	0.000852	AKT1/CAV1/GSK3B
GO:0010800	positive regulation of peptidyl-threonine phosphorylation	2/32	29/18670	0.001123	0.003127	0.000852	MAPK1/TGFB1
GO:0010954	positive regulation of protein processing	2/32	29/18670	0.001123	0.003127	0.000852	IL1B/SRC
GO:0042226	interleukin-6 biosynthetic process	2/32	29/18670	0.001123	0.003127	0.000852	IL1B/TNF
GO:0044030	regulation of DNA methylation	2/32	29/18670	0.001123	0.003127	0.000852	PARP1/MYC
GO:0045980	negative regulation of nucleotide metabolic process	2/32	29/18670	0.001123	0.003127	0.000852	PARP1/SNCA
GO:0048730	epidermis morphogenesis	2/32	29/18670	0.001123	0.003127	0.000852	BCL2/CTNNB1
GO:0060441	epithelial tube branching involved in lung morphogenesis	2/32	29/18670	0.001123	0.003127	0.000852	CTNNB1/TNF
GO:0070229	negative regulation of lymphocyte apoptotic process	2/32	29/18670	0.001123	0.003127	0.000852	IL2/JAK3
GO:1900027	regulation of ruffle assembly	2/32	29/18670	0.001123	0.003127	0.000852	CAV1/ICAM1
GO:2000515	negative regulation of CD4-positive, alpha-beta T cell activation	2/32	29/18670	0.001123	0.003127	0.000852	IL2/JAK3
GO:2000727	positive regulation of cardiac muscle cell differentiation	2/32	29/18670	0.001123	0.003127	0.000852	IGF1/TGFB1
GO:1901617	organic hydroxy compound biosynthetic process	4/32	271/18670	0.001134	0.003155	0.00086	FGF2/IL1B/SNCA/TNF

GO:0010811	positive regulation of cell-substrate adhesion	3/32	121/18670	0.001148	0.003193	0.00087	ABL1/GSK3B/KDR
GO:0002429	immune response-activating cell surface receptor signaling pathway	5/32	473/18670	0.001169	0.003248	0.000885	ABL1/BCL2/HSP90AA1/MAPK1/SRC
GO:0032434	regulation of proteasomal ubiquitin-dependent protein catabolic process	3/32	122/18670	0.001176	0.003258	0.000888	AKT1/CAV1/GSK3B
GO:0043500	muscle adaptation	3/32	122/18670	0.001176	0.003258	0.000888	PARP1/IGF1/IL1B
GO:1903578	regulation of ATP metabolic process	3/32	122/18670	0.001176	0.003258	0.000888	PARP1/IGF1/SNCA
GO:0001782	B cell homeostasis	2/32	30/18670	0.001201	0.003312	0.000903	ABL1/BCL2
GO:0001844	protein insertion into mitochondrial membrane involved in apoptotic signaling pathway	2/32	30/18670	0.001201	0.003312	0.000903	BCL2/CASP8
GO:0035066	positive regulation of histone acetylation	2/32	30/18670	0.001201	0.003312	0.000903	IL1B/TGFB1
GO:0045940	positive regulation of steroid metabolic process	2/32	30/18670	0.001201	0.003312	0.000903	IL1B/TNF
GO:0071549	cellular response to dexamethasone stimulus	2/32	30/18670	0.001201	0.003312	0.000903	ICAM1/TGFB1
GO:1904837	beta-catenin-TCF complex assembly	2/32	30/18670	0.001201	0.003312	0.000903	CTNNB1/MYC
GO:0016311	dephosphorylation	5/32	478/18670	0.001225	0.003375	0.00092	BCL2/GSK3B/SRC/TGFB1/TNF
GO:0001838	embryonic epithelial tube formation	3/32	124/18670	0.001232	0.003391	0.000924	ABL1/CTNNB1/TGFB1

GO:1901987	regulation of cell cycle phase transition	5/32	480/18670	0.001248	0.003432	0.000935	AKT1/CCND1/BCL2/HSP90AA1/TGFB1
GO:0000082	G1/S transition of mitotic cell cycle	4/32	279/18670	0.001262	0.003468	0.000945	AKT1/CCND1/BCL2/MYC
GO:0009152	purine ribonucleotide biosynthetic process	4/32	280/18670	0.001279	0.003498	0.000953	PARP1/IGF1/SNCA/TGFB1
GO:0030522	intracellular receptor signaling pathway	4/32	280/18670	0.001279	0.003498	0.000953	PARP1/CASP8/CTNNB1/SRC
GO:0001963	synaptic transmission, dopaminergic	2/32	31/18670	0.001283	0.003498	0.000953	PTGS2/SNCA
GO:0033198	response to ATP	2/32	31/18670	0.001283	0.003498	0.000953	IL1B/PTGS2
GO:0033687	osteoblast proliferation	2/32	31/18670	0.001283	0.003498	0.000953	ABL1/BCL2
GO:0035767	endothelial cell chemotaxis	2/32	31/18670	0.001283	0.003498	0.000953	FGF2/KDR
GO:0045742	positive regulation of epidermal growth factor receptor signaling pathway	2/32	31/18670	0.001283	0.003498	0.000953	AKT1/FASLG
GO:1903319	positive regulation of protein maturation	2/32	31/18670	0.001283	0.003498	0.000953	IL1B/SRC
GO:1903393	positive regulation of adherens junction organization	2/32	31/18670	0.001283	0.003498	0.000953	ABL1/KDR
GO:0032479	regulation of type I interferon production	3/32	126/18670	0.00129	0.003509	0.000956	CTNNB1/IL10/IRF1
GO:0045667	regulation of osteoblast differentiation	3/32	126/18670	0.00129	0.003509	0.000956	CTNNB1/IGF1/TNF

GO:0090101	negative regulation of transmembrane receptor protein serine/threonine kinase signaling pathway	3/32	126/18670	0.00129	0.003509	0.000956	ABL1/CAV1/TGFB1
GO:0035270	endocrine system development	3/32	127/18670	0.00132	0.003583	0.000976	AKT1/GSK3B/MAPK1
GO:0045727	positive regulation of translation	3/32	127/18670	0.00132	0.003583	0.000976	ERBB2/MAPK1/TNF
GO:0002687	positive regulation of leukocyte migration	3/32	128/18670	0.00135	0.003659	0.000997	ICAM1/TGFB1/TNF
GO:0032606	type I interferon production	3/32	128/18670	0.00135	0.003659	0.000997	CTNNB1/IL10/IRF1
GO:0001569	branching involved in blood vessel morphogenesis	2/32	32/18670	0.001367	0.003692	0.001006	ABL1/CTNNB1
GO:0060674	placenta blood vessel development	2/32	32/18670	0.001367	0.003692	0.001006	AKT1/MAPK1
GO:0061311	cell surface receptor signaling pathway involved in heart development	2/32	32/18670	0.001367	0.003692	0.001006	CTNNB1/TGFB1
GO:2000778	positive regulation of interleukin-6 secretion	2/32	32/18670	0.001367	0.003692	0.001006	IL1B/TNF
GO:0050853	B cell receptor signaling pathway	3/32	129/18670	0.001381	0.003723	0.001014	ABL1/BCL2/MAPK1
GO:0071482	cellular response to light stimulus	3/32	129/18670	0.001381	0.003723	0.001014	PARP1/MYC/PTGS2
GO:0060828	regulation of canonical Wnt signaling pathway	4/32	286/18670	0.001383	0.003726	0.001015	CAV1/CTNNB1/GSK3B/SRC
GO:0010464	regulation of mesenchymal cell proliferation	2/32	33/18670	0.001454	0.003887	0.001059	CTNNB1/MYC

GO:0048536	spleen development	2/32	33/18670	0.001454	0.003887	0.001059	ABL1/BCL2
GO:0051194	positive regulation of cofactor metabolic process	2/32	33/18670	0.001454	0.003887	0.001059	IGF1/SNCA
GO:0051204	protein insertion into mitochondrial membrane	2/32	33/18670	0.001454	0.003887	0.001059	BCL2/CASP8
GO:0060603	mammary gland duct morphogenesis	2/32	33/18670	0.001454	0.003887	0.001059	SRC/TGFB1
GO:1901186	positive regulation of ERBB signaling pathway	2/32	33/18670	0.001454	0.003887	0.001059	AKT1/FASLG
GO:1902692	regulation of neuroblast proliferation	2/32	33/18670	0.001454	0.003887	0.001059	CTNNB1/TGFB1
GO:2000758	positive regulation of peptidyl-lysine acetylation	2/32	33/18670	0.001454	0.003887	0.001059	IL1B/TGFB1
GO:2001024	negative regulation of response to drug	2/32	33/18670	0.001454	0.003887	0.001059	IL10/SNCA
GO:0030518	intracellular steroid hormone receptor signaling pathway	3/32	132/18670	0.001475	0.00393	0.001071	PARP1/CTNNB1/SRC
GO:0042476	odontogenesis	3/32	132/18670	0.001475	0.00393	0.001071	CTNNB1/SRC/TGFB1
GO:0045995	regulation of embryonic development	3/32	132/18670	0.001475	0.00393	0.001071	CTNNB1/IGF1/IL10
GO:0072175	epithelial tube formation	3/32	132/18670	0.001475	0.00393	0.001071	ABL1/CTNNB1/TGFB1
GO:0010975	regulation of neuron projection development	5/32	499/18670	0.001482	0.003947	0.001076	ABL1/AKT1/GSK3B/IL2/MAPT

GO:0006310	DNA recombination	4/32	292/18670	0.001493	0.003971	0.001082	PARP1/IL2/IL10/TGFB1
GO:0009260	ribonucleotide biosynthetic process	4/32	293/18670	0.001511	0.004015	0.001094	PARP1/IGF1/SNCA/TGFB1
GO:0051146	striated muscle cell differentiation	4/32	293/18670	0.001511	0.004015	0.001094	AKT1/BCL2/IGF1/TGFB1
GO:0032273	positive regulation of protein polymerization	3/32	134/18670	0.00154	0.004078	0.001111	HSP90AA1/ICAM1/MAPT
GO:0046683	response to organophosphorus	3/32	134/18670	0.00154	0.004078	0.001111	IL1B/JUN/PTGS2
GO:0007435	salivary gland morphogenesis	2/32	34/18670	0.001543	0.004078	0.001111	TGFB1/TNF
GO:0032212	positive regulation of telomere maintenance via telomerase	2/32	34/18670	0.001543	0.004078	0.001111	CTNNB1/MAPK1
GO:0032660	regulation of interleukin-17 production	2/32	34/18670	0.001543	0.004078	0.001111	IL2/TGFB1
GO:0034205	amyloid-beta formation	2/32	34/18670	0.001543	0.004078	0.001111	IGF1/TNF
GO:0050921	positive regulation of chemotaxis	3/32	135/18670	0.001573	0.004151	0.001131	FGF2/KDR/TGFB1
GO:0060359	response to ammonium ion	3/32	135/18670	0.001573	0.004151	0.001131	ABL1/MAPK1/SNCA
GO:0002758	innate immune response-activating signal transduction	4/32	298/18670	0.001608	0.004236	0.001154	CASP8/CAV1/IRF1/SRC
GO:0044843	cell cycle G1/S phase transition	4/32	298/18670	0.001608	0.004236	0.001154	AKT1/CCND1/BCL2/MYC

GO:0000266	mitochondrial fission	2/32	35/18670	0.001635	0.004289	0.001169	KDR/MAPT
GO:0014904	myotube cell development	2/32	35/18670	0.001635	0.004289	0.001169	BCL2/IGF1
GO:0045622	regulation of T-helper cell differentiation	2/32	35/18670	0.001635	0.004289	0.001169	IL2/JAK3
GO:0060251	regulation of glial cell proliferation	2/32	35/18670	0.001635	0.004289	0.001169	IL1B/TNF
GO:1902991	regulation of amyloid precursor protein catabolic process	2/32	35/18670	0.001635	0.004289	0.001169	IGF1/TNF
GO:0006164	purine nucleotide biosynthetic process	4/32	300/18670	0.001648	0.004317	0.001176	PARP1/IGF1/SNCA/TGFB1
GO:0046390	ribose phosphate biosynthetic process	4/32	300/18670	0.001648	0.004317	0.001176	PARP1/IGF1/SNCA/TGFB1
GO:0008584	male gonad development	3/32	138/18670	0.001675	0.004384	0.001195	CCND1/BCL2/ICAM1
GO:0031333	negative regulation of protein complex assembly	3/32	139/18670	0.00171	0.004465	0.001217	GSK3B/SNCA/SRC
GO:0035304	regulation of protein dephosphorylation	3/32	139/18670	0.00171	0.004465	0.001217	GSK3B/TGFB1/TNF
GO:0046546	development of primary male sexual characteristics	3/32	139/18670	0.00171	0.004465	0.001217	CCND1/BCL2/ICAM1
GO:0030224	monocyte differentiation	2/32	36/18670	0.001729	0.004494	0.001225	JUN/MYC
GO:0032205	negative regulation of telomere maintenance	2/32	36/18670	0.001729	0.004494	0.001225	PARP1/SRC

GO:0042554	superoxide anion generation	2/32	36/18670	0.001729	0.004494	0.001225	MAPT/TGFB1
GO:0071634	regulation of transforming growth factor beta production	2/32	36/18670	0.001729	0.004494	0.001225	IL13/PTGS2
GO:1903131	mononuclear cell differentiation	2/32	36/18670	0.001729	0.004494	0.001225	JUN/MYC
GO:0060326	cell chemotaxis	4/32	304/18670	0.00173	0.004494	0.001225	FGF2/IL1B/IL10/KDR
GO:0060078	regulation of postsynaptic membrane potential	3/32	140/18670	0.001746	0.004532	0.001235	AKT1/GSK3B/SNCA
GO:0046034	ATP metabolic process	4/32	305/18670	0.001751	0.004541	0.001238	PARP1/IGF1/SNCA/TGFB1
GO:0050709	negative regulation of protein secretion	3/32	141/18670	0.001781	0.004617	0.001258	IL1B/IL10/TNF
GO:1900542	regulation of purine nucleotide metabolic process	3/32	142/18670	0.001818	0.00469	0.001278	PARP1/IGF1/SNCA
GO:0007431	salivary gland development	2/32	37/18670	0.001826	0.00469	0.001278	TGFB1/TNF
GO:0033280	response to vitamin D	2/32	37/18670	0.001826	0.00469	0.001278	PTGS2/TGFB1
GO:0043114	regulation of vascular permeability	2/32	37/18670	0.001826	0.00469	0.001278	SRC/TGFB1
GO:0045923	positive regulation of fatty acid metabolic process	2/32	37/18670	0.001826	0.00469	0.001278	IL1B/PTGS2
GO:0046006	regulation of activated T cell proliferation	2/32	37/18670	0.001826	0.00469	0.001278	IGF1/IL2

GO:0071542	dopaminergic neuron differentiation	2/32	37/18670	0.001826	0.00469	0.001278	CTNNB1/GSK3B
GO:1903427	negative regulation of reactive oxygen species biosynthetic process	2/32	37/18670	0.001826	0.00469	0.001278	CAV1/IL10
GO:1904358	positive regulation of telomere maintenance via telomere lengthening	2/32	37/18670	0.001826	0.00469	0.001278	CTNNB1/MAPK1
GO:2000142	regulation of DNA-templated transcription, initiation	2/32	37/18670	0.001826	0.00469	0.001278	CTNNB1/JUN
GO:2000249	regulation of actin cytoskeleton reorganization	2/32	37/18670	0.001826	0.00469	0.001278	ABL1/TGFB1
GO:1990778	protein localization to cell periphery	4/32	311/18670	0.00188	0.004825	0.001315	AKT1/CAV1/TGFB1/TNF
GO:1904064	positive regulation of cation transmembrane transport	3/32	144/18670	0.001892	0.004852	0.001322	ABL1/IL13/SNCA
GO:0072522	purine-containing compound biosynthetic process	4/32	313/18670	0.001924	0.004891	0.001333	PARP1/IGF1/SNCA/TGFB1
GO:0010939	regulation of necrotic cell death	2/32	38/18670	0.001925	0.004891	0.001333	CASP8/CAV1
GO:0032350	regulation of hormone metabolic process	2/32	38/18670	0.001925	0.004891	0.001333	IL1B/TNF
GO:0032620	interleukin-17 production	2/32	38/18670	0.001925	0.004891	0.001333	IL2/TGFB1
GO:0045022	early endosome to late endosome transport	2/32	38/18670	0.001925	0.004891	0.001333	MAPK1/SRC
GO:0046326	positive regulation of glucose import	2/32	38/18670	0.001925	0.004891	0.001333	AKT1/IGF1

GO:0046636	negative regulation of alpha-beta T cell activation	2/32	38/18670	0.001925	0.004891	0.001333	IL2/JAK3
GO:0046825	regulation of protein export from nucleus	2/32	38/18670	0.001925	0.004891	0.001333	GSK3B/IL1B
GO:0048009	insulin-like growth factor receptor signaling pathway	2/32	38/18670	0.001925	0.004891	0.001333	AKT1/IGF1
GO:0060045	positive regulation of cardiac muscle cell proliferation	2/32	38/18670	0.001925	0.004891	0.001333	FGF2/MAPK1
GO:0060416	response to growth hormone	2/32	38/18670	0.001925	0.004891	0.001333	AKT1/JAK3
GO:0071604	transforming growth factor beta production	2/32	38/18670	0.001925	0.004891	0.001333	IL13/PTGS2
GO:0002224	toll-like receptor signaling pathway	3/32	146/18670	0.001968	0.004983	0.001358	CASP8/CAV1/IRF1
GO:0006140	regulation of nucleotide metabolic process	3/32	146/18670	0.001968	0.004983	0.001358	PARP1/IGF1/SNCA
GO:2000241	regulation of reproductive process	3/32	146/18670	0.001968	0.004983	0.001358	CTNNB1/IGF1/SRC
GO:2001251	negative regulation of chromosome organization	3/32	146/18670	0.001968	0.004983	0.001358	PARP1/SNCA/SRC
GO:0043524	negative regulation of neuron apoptotic process	3/32	147/18670	0.002006	0.005073	0.001383	BCL2/JUN/SNCA
GO:0071236	cellular response to antibiotic	3/32	147/18670	0.002006	0.005073	0.001383	ABL1/IL10/SRC
GO:0019048	modulation by virus of host morphology or physiology	2/32	39/18670	0.002027	0.005113	0.001393	CASP8/TGFB1

GO:0032692	negative regulation of interleukin-1 production	2/32	39/18670	0.002027	0.005113	0.001393	IGF1/IL10
GO:0033146	regulation of intracellular estrogen receptor signaling pathway	2/32	39/18670	0.002027	0.005113	0.001393	PARP1/SRC
GO:0002792	negative regulation of peptide secretion	3/32	148/18670	0.002046	0.005148	0.001403	IL1B/IL10/TNF
GO:0035148	tube formation	3/32	148/18670	0.002046	0.005148	0.001403	ABL1/CTNNB1/TGFB1
GO:0051592	response to calcium ion	3/32	148/18670	0.002046	0.005148	0.001403	CCND1/CAV1/JUN
GO:0002218	activation of innate immune response	4/32	319/18670	0.002062	0.005185	0.001413	CASP8/CAV1/IRF1/SRC
GO:0009267	cellular response to starvation	3/32	149/18670	0.002085	0.005227	0.001424	BCL2/JUN/MAPK1
GO:0014074	response to purine-containing compound	3/32	149/18670	0.002085	0.005227	0.001424	IL1B/JUN/PTGS2
GO:0048592	eye morphogenesis	3/32	149/18670	0.002085	0.005227	0.001424	FASLG/BCL2/CTNNB1
GO:2000058	regulation of ubiquitin-dependent protein catabolic process	3/32	149/18670	0.002085	0.005227	0.001424	AKT1/CAV1/GSK3B
GO:0006470	protein dephosphorylation	4/32	321/18670	0.002109	0.005283	0.00144	BCL2/GSK3B/TGFB1/TNF
GO:0016331	morphogenesis of embryonic epithelium	3/32	150/18670	0.002125	0.005319	0.00145	ABL1/CTNNB1/TGFB1

GO:0002823	negative regulation of adaptive immune response based on somatic recombination of immune receptors built from immunoglobulin superfamily domains	2/32	40/18670	0.002131	0.005322	0.00145	IL2/JAK3
GO:0071548	response to dexamethasone	2/32	40/18670	0.002131	0.005322	0.00145	ICAM1/TGFB1
GO:0090184	positive regulation of kidney development	2/32	40/18670	0.002131	0.005322	0.00145	MYC/TGFB1
GO:0016050	vesicle organization	4/32	325/18670	0.002206	0.005489	0.001496	FASLG/BCL2/CAV1/SNCA
GO:0030902	hindbrain development	3/32	152/18670	0.002207	0.005489	0.001496	ABL1/BCL2/CTNNB1
GO:0032675	regulation of interleukin-6 production	3/32	152/18670	0.002207	0.005489	0.001496	IL1B/IL10/TNF
GO:0048489	synaptic vesicle transport	3/32	152/18670	0.002207	0.005489	0.001496	CTNNB1/GSK3B/SNCA
GO:0097480	establishment of synaptic vesicle localization	3/32	152/18670	0.002207	0.005489	0.001496	CTNNB1/GSK3B/SNCA
GO:0040019	positive regulation of embryonic development	2/32	41/18670	0.002238	0.005546	0.001511	CTNNB1/IGF1
GO:0097178	ruffle assembly	2/32	41/18670	0.002238	0.005546	0.001511	CAV1/ICAM1
GO:0098927	vesicle-mediated transport between endosomal compartments	2/32	41/18670	0.002238	0.005546	0.001511	MAPK1/SRC
GO:1903053	regulation of extracellular matrix organization	2/32	41/18670	0.002238	0.005546	0.001511	ABL1/TGFB1

GO:1905209	positive regulation of cardiocyte differentiation	2/32	41/18670	0.002238	0.005546	0.001511	IGF1/TGFB1
GO:0034250	positive regulation of cellular amide metabolic process	3/32	153/18670	0.002248	0.005562	0.001516	ERBB2/MAPK1/TNF
GO:0045807	positive regulation of endocytosis	3/32	153/18670	0.002248	0.005562	0.001516	IL1B/SNCA/TNF
GO:0016485	protein processing	4/32	328/18670	0.002281	0.005634	0.001535	PARP1/CASP8/IL1B/SRC
GO:0019058	viral life cycle	4/32	328/18670	0.002281	0.005634	0.001535	BCL2/CAV1/ICAM1/TNF
GO:0055067	monovalent inorganic cation homeostasis	3/32	154/18670	0.002291	0.005653	0.001541	FASLG/BCL2/MAPK1
GO:0010613	positive regulation of cardiac muscle hypertrophy	2/32	42/18670	0.002348	0.005777	0.001574	PARP1/IGF1
GO:0031641	regulation of myelination	2/32	42/18670	0.002348	0.005777	0.001574	AKT1/CTNNB1
GO:0032958	inositol phosphate biosynthetic process	2/32	42/18670	0.002348	0.005777	0.001574	FGF2/SNCA
GO:1901031	regulation of response to reactive oxygen species	2/32	42/18670	0.002348	0.005777	0.001574	IL10/TNF
GO:0016573	histone acetylation	3/32	156/18670	0.002376	0.005833	0.001589	IL1B/SNCA/TGFB1
GO:0034767	positive regulation of ion transmembrane transport	3/32	156/18670	0.002376	0.005833	0.001589	ABL1/IL13/SNCA
GO:0045216	cell-cell junction organization	3/32	156/18670	0.002376	0.005833	0.001589	CAV1/TGFB1/TNF

GO:0090150	establishment of protein localization to membrane	4/32	332/18670	0.002384	0.005847	0.001593	BCL2/CASP8/ERBB2/HSP90AA1
GO:1905952	regulation of lipid localization	3/32	157/18670	0.002419	0.00593	0.001616	AKT1/IL1B/TNF
GO:0030336	negative regulation of cell migration	4/32	334/18670	0.002436	0.005966	0.001626	AKT1/BCL2/FGF2/TGFB1
GO:0006692	prostanoid metabolic process	2/32	43/18670	0.00246	0.005985	0.001631	IL1B/PTGS2
GO:0006693	prostaglandin metabolic process	2/32	43/18670	0.00246	0.005985	0.001631	IL1B/PTGS2
GO:0006775	fat-soluble vitamin metabolic process	2/32	43/18670	0.00246	0.005985	0.001631	IL1B/TNF
GO:0014742	positive regulation of muscle hypertrophy	2/32	43/18670	0.00246	0.005985	0.001631	PARP1/IGF1
GO:0042088	T-helper 1 type immune response	2/32	43/18670	0.00246	0.005985	0.001631	IL1B/JAK3
GO:0050850	positive regulation of calcium-mediated signaling	2/32	43/18670	0.00246	0.005985	0.001631	IGF1/TNF
GO:0071364	cellular response to epidermal growth factor stimulus	2/32	43/18670	0.00246	0.005985	0.001631	AKT1/ERBB2
GO:1901985	positive regulation of protein acetylation	2/32	43/18670	0.00246	0.005985	0.001631	IL1B/TGFB1
GO:0009205	purine ribonucleoside triphosphate metabolic process	4/32	335/18670	0.002462	0.005985	0.001631	PARP1/IGF1/SNCA/TGFB1
GO:0060070	canonical Wnt signaling pathway	4/32	335/18670	0.002462	0.005985	0.001631	CAV1/CTNNB1/GSK3B/SRC

GO:0007596	blood coagulation	4/32	336/18670	0.002489	0.006046	0.001648	CAV1/IRF1/MAPK1/SRC
GO:0002673	regulation of acute inflammatory response	3/32	159/18670	0.002508	0.006082	0.001657	IL1B/PTGS2/TNF
GO:0098727	maintenance of cell number	3/32	159/18670	0.002508	0.006082	0.001657	CTNNB1/FGF2/IGF1
GO:0006754	ATP biosynthetic process	3/32	160/18670	0.002553	0.006186	0.001686	PARP1/IGF1/TGFB1
GO:0010463	mesenchymal cell proliferation	2/32	44/18670	0.002574	0.006214	0.001693	CTNNB1/MYC
GO:0010828	positive regulation of glucose transmembrane transport	2/32	44/18670	0.002574	0.006214	0.001693	AKT1/IGF1
GO:0042987	amyloid precursor protein catabolic process	2/32	44/18670	0.002574	0.006214	0.001693	IGF1/TNF
GO:0061756	leukocyte adhesion to vascular endothelial cell	2/32	44/18670	0.002574	0.006214	0.001693	ICAM1/TNF
GO:0090311	regulation of protein deacetylation	2/32	44/18670	0.002574	0.006214	0.001693	MAPT/TGFB1
GO:0009167	purine ribonucleoside monophosphate metabolic process	4/32	340/18670	0.002598	0.006259	0.001706	PARP1/IGF1/SNCA/TGFB1
GO:0018393	internal peptidyl-lysine acetylation	3/32	161/18670	0.002598	0.006259	0.001706	IL1B/SNCA/TGFB1
GO:0032635	interleukin-6 production	3/32	161/18670	0.002598	0.006259	0.001706	IL1B/IL10/TNF
GO:0007599	hemostasis	4/32	341/18670	0.002626	0.00631	0.00172	CAV1/IRF1/MAPK1/SRC

GO:0009126	purine nucleoside monophosphate metabolic process	4/32	341/18670	0.002626	0.00631	0.00172	PARP1/IGF1/SNCA/TGFB1
GO:0009199	ribonucleoside triphosphate metabolic process	4/32	341/18670	0.002626	0.00631	0.00172	PARP1/IGF1/SNCA/TGFB1
GO:0009144	purine nucleoside triphosphate metabolic process	4/32	342/18670	0.002653	0.006367	0.001735	PARP1/IGF1/SNCA/TGFB1
GO:0050817	coagulation	4/32	342/18670	0.002653	0.006367	0.001735	CAV1/IRF1/MAPK1/SRC
GO:0031214	biomineral tissue development	3/32	163/18670	0.002691	0.006429	0.001752	IGF1/PTGS2/TGFB1
GO:0097479	synaptic vesicle localization	3/32	163/18670	0.002691	0.006429	0.001752	CTNNB1/GSK3B/SNCA
GO:0002820	negative regulation of adaptive immune response	2/32	45/18670	0.002691	0.006429	0.001752	IL2/JAK3
GO:0031018	endocrine pancreas development	2/32	45/18670	0.002691	0.006429	0.001752	AKT1/GSK3B
GO:1900271	regulation of long-term synaptic potentiation	2/32	45/18670	0.002691	0.006429	0.001752	ABL1/GSK3B
GO:1900274	regulation of phospholipase C activity	2/32	45/18670	0.002691	0.006429	0.001752	ABL1/FGF2
GO:0035821	modification of morphology or physiology of other organism	3/32	164/18670	0.002738	0.006535	0.001781	CASP8/JUN/TGFB1
GO:0050680	negative regulation of epithelial cell proliferation	3/32	165/18670	0.002785	0.006639	0.001809	CAV1/TGFB1/TNF
GO:0071695	anatomical structure maturation	3/32	165/18670	0.002785	0.006639	0.001809	BCL2/CTNNB1/IGF1

GO:0002067	glandular epithelial cell differentiation	2/32	46/18670	0.00281	0.006674	0.001819	GSK3B/IL13
GO:0031648	protein destabilization	2/32	46/18670	0.00281	0.006674	0.001819	SNCA/SRC
GO:0043330	response to exogenous dsRNA	2/32	46/18670	0.00281	0.006674	0.001819	CAV1/MAPK1
GO:0044003	modification by symbiont of host morphology or physiology	2/32	46/18670	0.00281	0.006674	0.001819	CASP8/TGFB1
GO:0071675	regulation of mononuclear cell migration	2/32	46/18670	0.00281	0.006674	0.001819	TGFB1/TNF
GO:0009101	glycoprotein biosynthetic process	4/32	348/18670	0.002825	0.006704	0.001827	BCL2/CTNNB1/IGF1/JAK3
GO:0002244	hematopoietic progenitor cell differentiation	3/32	166/18670	0.002833	0.006713	0.001829	ABL1/BCL2/TGFB1
GO:0090288	negative regulation of cellular response to growth factor stimulus	3/32	166/18670	0.002833	0.006713	0.001829	ABL1/CAV1/TGFB1
GO:2000146	negative regulation of cell motility	4/32	349/18670	0.002854	0.006759	0.001842	AKT1/BCL2/FGF2/TGFB1
GO:0035051	cardiocyte differentiation	3/32	167/18670	0.002882	0.006818	0.001858	IGF1/MAPK1/TGFB1
GO:0010799	regulation of peptidyl-threonine phosphorylation	2/32	47/18670	0.002932	0.006917	0.001885	MAPK1/TGFB1
GO:0043370	regulation of CD4-positive, alpha-beta T cell differentiation	2/32	47/18670	0.002932	0.006917	0.001885	IL2/JAK3
GO:0050435	amyloid-beta metabolic process	2/32	47/18670	0.002932	0.006917	0.001885	IGF1/TNF

GO:2000107	negative regulation of leukocyte apoptotic process	2/32	47/18670	0.002932	0.006917	0.001885	IL2/JAK3
GO:1902903	regulation of supramolecular fiber organization	4/32	352/18670	0.002944	0.006939	0.001891	ABL1/ICAM1/MAPT/SNCA
GO:0018394	peptidyl-lysine acetylation	3/32	169/18670	0.00298	0.007015	0.001912	IL1B/SNCA/TGFB1
GO:0050728	negative regulation of inflammatory response	3/32	169/18670	0.00298	0.007015	0.001912	IGF1/IL2/IL10
GO:0009161	ribonucleoside monophosphate metabolic process	4/32	354/18670	0.003004	0.007067	0.001926	PARP1/IGF1/SNCA/TGFB1
GO:0002762	negative regulation of myeloid leukocyte differentiation	2/32	48/18670	0.003056	0.007152	0.001949	CTNNB1/MYC
GO:0007595	lactation	2/32	48/18670	0.003056	0.007152	0.001949	CCND1/CAV1
GO:0035272	exocrine system development	2/32	48/18670	0.003056	0.007152	0.001949	TGFB1/TNF
GO:0045912	negative regulation of carbohydrate metabolic process	2/32	48/18670	0.003056	0.007152	0.001949	GSK3B/TGFB1
GO:0060324	face development	2/32	48/18670	0.003056	0.007152	0.001949	MAPK1/TGFB1
GO:0072604	interleukin-6 secretion	2/32	48/18670	0.003056	0.007152	0.001949	IL1B/TNF
GO:0090199	regulation of release of cytochrome c from mitochondria	2/32	48/18670	0.003056	0.007152	0.001949	AKT1/IGF1
GO:0009206	purine ribonucleoside triphosphate biosynthetic process	3/32	171/18670	0.003081	0.007199	0.001962	PARP1/IGF1/TGFB1

GO:0043901	negative regulation of multi-organism process	3/32	171/18670	0.003081	0.007199	0.001962	IGF1/JUN/TNF
GO:0009145	purine nucleoside triphosphate biosynthetic process	3/32	172/18670	0.003132	0.007313	0.001993	PARP1/IGF1/TGFB1
GO:0032757	positive regulation of interleukin-8 production	2/32	49/18670	0.003183	0.007405	0.002018	IL1B/TNF
GO:0043124	negative regulation of I-kappaB kinase/NF-kappaB signaling	2/32	49/18670	0.003183	0.007405	0.002018	ABL1/CASP8
GO:0048546	digestive tract morphogenesis	2/32	49/18670	0.003183	0.007405	0.002018	BCL2/CTNNB1
GO:0051205	protein insertion into membrane	2/32	49/18670	0.003183	0.007405	0.002018	BCL2/CASP8
GO:1990090	cellular response to nerve growth factor stimulus	2/32	49/18670	0.003183	0.007405	0.002018	AKT1/MAPT
GO:0009141	nucleoside triphosphate metabolic process	4/32	362/18670	0.003255	0.007568	0.002062	PARP1/IGF1/SNCA/TGFB1
GO:0002931	response to ischemia	2/32	50/18670	0.003312	0.007661	0.002088	BCL2/CAV1
GO:0009409	response to cold	2/32	50/18670	0.003312	0.007661	0.002088	CASP8/HSP90AA1
GO:0014009	glial cell proliferation	2/32	50/18670	0.003312	0.007661	0.002088	IL1B/TNF
GO:0032873	negative regulation of stress-activated MAPK cascade	2/32	50/18670	0.003312	0.007661	0.002088	AKT1/MYC
GO:0070303	negative regulation of stress-activated protein kinase signaling cascade	2/32	50/18670	0.003312	0.007661	0.002088	AKT1/MYC

GO:1900087	positive regulation of G1/S transition of mitotic cell cycle	2/32	50/18670	0.003312	0.007661	0.002088	AKT1/CCND1
GO:2000725	regulation of cardiac muscle cell differentiation	2/32	50/18670	0.003312	0.007661	0.002088	IGF1/TGFB1
GO:0032102	negative regulation of response to external stimulus	4/32	365/18670	0.003353	0.00775	0.002112	FGF2/IGF1/IL2/IL10
GO:0001959	regulation of cytokine-mediated signaling pathway	3/32	177/18670	0.003395	0.00783	0.002134	CASP8/CAV1/TNF
GO:0009201	ribonucleoside triphosphate biosynthetic process	3/32	177/18670	0.003395	0.00783	0.002134	PARP1/IGF1/TGFB1
GO:0060491	regulation of cell projection assembly	3/32	177/18670	0.003395	0.00783	0.002134	CAV1/ICAM1/SRC
GO:0007566	embryo implantation	2/32	51/18670	0.003443	0.007914	0.002157	IL1B/PTGS2
GO:0031103	axon regeneration	2/32	51/18670	0.003443	0.007914	0.002157	BCL2/JUN
GO:0032206	positive regulation of telomere maintenance	2/32	51/18670	0.003443	0.007914	0.002157	CTNNB1/MAPK1
GO:0045599	negative regulation of fat cell differentiation	2/32	51/18670	0.003443	0.007914	0.002157	TGFB1/TNF
GO:0051452	intracellular pH reduction	2/32	51/18670	0.003443	0.007914	0.002157	FASLG/BCL2
GO:0006611	protein export from nucleus	3/32	179/18670	0.003504	0.008029	0.002188	GSK3B/IL1B/TGFB1
GO:0030177	positive regulation of Wnt signaling pathway	3/32	179/18670	0.003504	0.008029	0.002188	ABL1/CAV1/SRC

GO:0071347	cellular response to interleukin-1	3/32	179/18670	0.003504	0.008029	0.002188	ICAM1/IL1A/IL1B
GO:1903034	regulation of response to wounding	3/32	179/18670	0.003504	0.008029	0.002188	CAV1/FGF2/IL10
GO:0031529	ruffle organization	2/32	52/18670	0.003577	0.008186	0.002231	CAV1/ICAM1
GO:1990089	response to nerve growth factor	2/32	52/18670	0.003577	0.008186	0.002231	AKT1/MAPT
GO:0009123	nucleoside monophosphate metabolic process	4/32	375/18670	0.003693	0.008444	0.002301	PARP1/IGF1/SNCA/TGFB1
GO:0032715	negative regulation of interleukin-6 production	2/32	53/18670	0.003714	0.008465	0.002307	IL10/TNF
GO:0043331	response to dsRNA	2/32	53/18670	0.003714	0.008465	0.002307	CAV1/MAPK1
GO:0045661	regulation of myoblast differentiation	2/32	53/18670	0.003714	0.008465	0.002307	TGFB1/TNF
GO:0045851	pH reduction	2/32	53/18670	0.003714	0.008465	0.002307	FASLG/BCL2
GO:0009127	purine nucleoside monophosphate biosynthetic process	3/32	183/18670	0.003728	0.008465	0.002307	PARP1/IGF1/TGFB1
GO:0009168	purine ribonucleoside monophosphate biosynthetic process	3/32	183/18670	0.003728	0.008465	0.002307	PARP1/IGF1/TGFB1
GO:0021953	central nervous system neuron differentiation	3/32	183/18670	0.003728	0.008465	0.002307	CTNNB1/HSP90AA1/MAPT
GO:0038061	NIK/NF-kappaB signaling	3/32	183/18670	0.003728	0.008465	0.002307	AKT1/IL1B/TNF

GO:0050770	regulation of axonogenesis	3/32	183/18670	0.003728	0.008465	0.002307	ABL1/GSK3B/MAPT
GO:0061136	regulation of proteasomal protein catabolic process	3/32	183/18670	0.003728	0.008465	0.002307	AKT1/CAV1/GSK3B
GO:0043401	steroid hormone mediated signaling pathway	3/32	184/18670	0.003785	0.008583	0.002339	PARP1/CTNNB1/SRC
GO:2000045	regulation of G1/S transition of mitotic cell cycle	3/32	184/18670	0.003785	0.008583	0.002339	AKT1/CCND1/BCL2
GO:0046328	regulation of JNK cascade	3/32	185/18670	0.003843	0.008708	0.002373	AKT1/IL1B/TNF
GO:0006636	unsaturated fatty acid biosynthetic process	2/32	54/18670	0.003852	0.008722	0.002377	IL1B/PTGS2
GO:0045089	positive regulation of innate immune response	4/32	381/18670	0.003908	0.008841	0.002409	CASP8/CAV1/IRF1/SRC
GO:0042304	regulation of fatty acid biosynthetic process	2/32	55/18670	0.003994	0.009023	0.002459	IL1B/PTGS2
GO:0046456	icosanoid biosynthetic process	2/32	55/18670	0.003994	0.009023	0.002459	IL1B/PTGS2
GO:0051271	negative regulation of cellular component movement	4/32	384/18670	0.004018	0.009071	0.002472	AKT1/BCL2/FGF2/TGFB1
GO:0009142	nucleoside triphosphate biosynthetic process	3/32	188/18670	0.004021	0.009071	0.002472	PARP1/IGF1/TGFB1
GO:1903078	positive regulation of protein localization to plasma membrane	2/32	56/18670	0.004137	0.009327	0.002542	AKT1/TNF
GO:0060759	regulation of response to cytokine stimulus	3/32	190/18670	0.004141	0.00933	0.002543	CASP8/CAV1/TNF

GO:0032970	regulation of actin filament-based process	4/32	388/18670	0.004169	0.009386	0.002558	ABL1/CAV1/ICAM1/TGFB1
GO:0042594	response to starvation	3/32	191/18670	0.004203	0.009455	0.002577	BCL2/JUN/MAPK1
GO:0060043	regulation of cardiac muscle cell proliferation	2/32	57/18670	0.004283	0.009622	0.002622	FGF2/MAPK1
GO:0071398	cellular response to fatty acid	2/32	57/18670	0.004283	0.009622	0.002622	AKT1/SRC
GO:0007219	Notch signaling pathway	3/32	193/18670	0.004327	0.009707	0.002645	AKT1/MYC/TGFB1
GO:0051224	negative regulation of protein transport	3/32	193/18670	0.004327	0.009707	0.002645	IL1B/IL10/TNF
GO:0051168	nuclear export	3/32	194/18670	0.004389	0.009834	0.00268	GSK3B/IL1B/TGFB1
GO:0099504	synaptic vesicle cycle	3/32	194/18670	0.004389	0.009834	0.00268	CTNNB1/GSK3B/SNCA
GO:0030520	intracellular estrogen receptor signaling pathway	2/32	58/18670	0.004431	0.009886	0.002694	PARP1/SRC
GO:0032722	positive regulation of chemokine production	2/32	58/18670	0.004431	0.009886	0.002694	IL1B/TNF
GO:0035306	positive regulation of dephosphorylation	2/32	58/18670	0.004431	0.009886	0.002694	SRC/TGFB1
GO:0042093	T-helper cell differentiation	2/32	58/18670	0.004431	0.009886	0.002694	IL2/JAK3
GO:0071385	cellular response to glucocorticoid stimulus	2/32	58/18670	0.004431	0.009886	0.002694	ICAM1/TGFB1

GO:1903749	positive regulation of establishment of protein localization to mitochondrion	2/32	58/18670	0.004431	0.009886	0.002694	BCL2/CASP8
GO:0040013	negative regulation of locomotion	4/32	396/18670	0.004482	0.009992	0.002723	AKT1/BCL2/FGF2/TGFB1
GO:0018205	peptidyl-lysine modification	4/32	397/18670	0.004522	0.010068	0.002744	CTNNB1/IL1B/SNCA/TGFB1
GO:0051604	protein maturation	4/32	397/18670	0.004522	0.010068	0.002744	PARP1/CASP8/IL1B/SRC
GO:0002221	pattern recognition receptor signaling pathway	3/32	197/18670	0.004581	0.01018	0.002774	CASP8/CAV1/IRF1
GO:0009156	ribonucleoside monophosphate biosynthetic process	3/32	197/18670	0.004581	0.01018	0.002774	PARP1/IGF1/TGFB1
GO:0031102	neuron projection regeneration	2/32	59/18670	0.004582	0.01018	0.002774	BCL2/JUN
GO:0007015	actin filament organization	4/32	400/18670	0.004644	0.010304	0.002808	ABL1/BCL2/ICAM1/SRC
GO:0010876	lipid localization	4/32	400/18670	0.004644	0.010304	0.002808	AKT1/CAV1/IL1B/TNF
GO:0034341	response to interferon-gamma	3/32	199/18670	0.004712	0.010448	0.002847	ICAM1/IRF1/SNCA
GO:0002294	CD4-positive, alpha-beta T cell differentiation involved in immune response	2/32	60/18670	0.004735	0.010476	0.002855	IL2/JAK3
GO:0032515	negative regulation of phosphoprotein phosphatase activity	2/32	60/18670	0.004735	0.010476	0.002855	GSK3B/TNF
GO:1902808	positive regulation of cell cycle G1/S phase transition	2/32	60/18670	0.004735	0.010476	0.002855	AKT1/CCND1

GO:0043547	positive regulation of GTPase activity	4/32	405/18670	0.004853	0.010729	0.002924	ERBB2/GSK3B/ICAM1/JUN
GO:0002287	alpha-beta T cell activation involved in immune response	2/32	61/18670	0.00489	0.01073	0.002924	IL2/JAK3
GO:0002293	alpha-beta T cell differentiation involved in immune response	2/32	61/18670	0.00489	0.01073	0.002924	IL2/JAK3
GO:0006027	glycosaminoglycan catabolic process	2/32	61/18670	0.00489	0.01073	0.002924	FGF2/TGFB1
GO:0007405	neuroblast proliferation	2/32	61/18670	0.00489	0.01073	0.002924	CTNNB1/TGFB1
GO:0010803	regulation of tumor necrosis factor-mediated signaling pathway	2/32	61/18670	0.00489	0.01073	0.002924	CASP8/TNF
GO:0045123	cellular extravasation	2/32	61/18670	0.00489	0.01073	0.002924	ICAM1/TNF
GO:0048645	animal organ formation	2/32	61/18670	0.00489	0.01073	0.002924	CTNNB1/MAPK1
GO:0071384	cellular response to corticosteroid stimulus	2/32	61/18670	0.00489	0.01073	0.002924	ICAM1/TGFB1
GO:1905953	negative regulation of lipid localization	2/32	61/18670	0.00489	0.01073	0.002924	AKT1/TNF
GO:2000514	regulation of CD4-positive, alpha-beta T cell activation	2/32	61/18670	0.00489	0.01073	0.002924	IL2/JAK3
GO:2001244	positive regulation of intrinsic apoptotic signaling pathway	2/32	61/18670	0.00489	0.01073	0.002924	BCL2/CAV1
GO:0009746	response to hexose	3/32	202/18670	0.004913	0.010765	0.002934	ICAM1/PTGS2/TGFB1

GO:1902806	regulation of cell cycle G1/S phase transition	3/32	202/18670	0.004913	0.010765	0.002934	AKT1/CCND1/BCL2
GO:0000280	nuclear division	4/32	407/18670	0.004938	0.010813	0.002947	IGF1/IL1A/IL1B/TGFB1
GO:0045453	bone resorption	2/32	62/18670	0.005048	0.011042	0.003009	CTNNB1/SRC
GO:1902905	positive regulation of supramolecular fiber organization	3/32	204/18670	0.005049	0.011042	0.003009	ABL1/ICAM1/MAPT
GO:0042982	amyloid precursor protein metabolic process	2/32	63/18670	0.005208	0.011365	0.003097	IGF1/TNF
GO:0046637	regulation of alpha-beta T cell differentiation	2/32	63/18670	0.005208	0.011365	0.003097	IL2/JAK3
GO:1904377	positive regulation of protein localization to cell periphery	2/32	63/18670	0.005208	0.011365	0.003097	AKT1/TNF
GO:0034284	response to monosaccharide	3/32	207/18670	0.005258	0.011459	0.003123	ICAM1/PTGS2/TGFB1
GO:0099003	vesicle-mediated transport in synapse	3/32	207/18670	0.005258	0.011459	0.003123	CTNNB1/GSK3B/SNCA
GO:0009124	nucleoside monophosphate biosynthetic process	3/32	208/18670	0.005329	0.011598	0.003161	PARP1/IGF1/TGFB1
GO:0050792	regulation of viral process	3/32	208/18670	0.005329	0.011598	0.003161	BCL2/JUN/TNF
GO:0045600	positive regulation of fat cell differentiation	2/32	64/18670	0.00537	0.011664	0.003179	AKT1/PTGS2
GO:0045669	positive regulation of osteoblast differentiation	2/32	64/18670	0.00537	0.011664	0.003179	CTNNB1/IGF1

GO:0045670	regulation of osteoclast differentiation	2/32	64/18670	0.00537	0.011664	0.003179	CTNNB1/TNF
GO:0051216	cartilage development	3/32	209/18670	0.0054	0.011721	0.003194	CTNNB1/FGF2/TGFB1
GO:0009100	glycoprotein metabolic process	4/32	419/18670	0.00547	0.011856	0.003231	BCL2/CTNNB1/IGF1/JAK3
GO:0043161	proteasome-mediated ubiquitin-dependent protein catabolic process	4/32	419/18670	0.00547	0.011856	0.003231	AKT1/CAV1/CTNNB1/GSK3B
GO:0006026	aminoglycan catabolic process	2/32	65/18670	0.005534	0.011972	0.003263	FGF2/TGFB1
GO:0006940	regulation of smooth muscle contraction	2/32	65/18670	0.005534	0.011972	0.003263	CAV1/PTGS2
GO:0035308	negative regulation of protein dephosphorylation	2/32	65/18670	0.005534	0.011972	0.003263	GSK3B/TNF
GO:0040014	regulation of multicellular organism growth	2/32	66/18670	0.005701	0.012294	0.00335	BCL2/IGF1
GO:0046626	regulation of insulin receptor signaling pathway	2/32	66/18670	0.005701	0.012294	0.00335	IL1B/SRC
GO:1904888	cranial skeletal system development	2/32	66/18670	0.005701	0.012294	0.00335	CTNNB1/TGFB1
GO:1905207	regulation of cardiocyte differentiation	2/32	66/18670	0.005701	0.012294	0.00335	IGF1/TGFB1
GO:0006417	regulation of translation	4/32	424/18670	0.005702	0.012294	0.00335	AKT1/ERBB2/MAPK1/TNF
GO:0007254	JNK cascade	3/32	214/18670	0.005765	0.012414	0.003383	AKT1/IL1B/TNF

GO:1903050	regulation of proteolysis involved in cellular protein catabolic process	3/32	214/18670	0.005765	0.012414	0.003383	AKT1/CAV1/GSK3B
GO:0030858	positive regulation of epithelial cell differentiation	2/32	67/18670	0.00587	0.012623	0.00344	CTNNB1/IL13
GO:0060038	cardiac muscle cell proliferation	2/32	67/18670	0.00587	0.012623	0.00344	FGF2/MAPK1
GO:0006644	phospholipid metabolic process	4/32	430/18670	0.00599	0.012849	0.003502	FGF2/SNCA/SRC/TGFB1
GO:0017157	regulation of exocytosis	3/32	217/18670	0.005992	0.012849	0.003502	GSK3B/IL13/SNCA
GO:0050920	regulation of chemotaxis	3/32	217/18670	0.005992	0.012849	0.003502	FGF2/KDR/TGFB1
GO:0060348	bone development	3/32	217/18670	0.005992	0.012849	0.003502	IGF1/SRC/TGFB1
GO:0002292	T cell differentiation involved in immune response	2/32	68/18670	0.006042	0.012913	0.003519	IL2/JAK3
GO:0035924	cellular response to vascular endothelial growth factor stimulus	2/32	68/18670	0.006042	0.012913	0.003519	AKT1/KDR
GO:0045806	negative regulation of endocytosis	2/32	68/18670	0.006042	0.012913	0.003519	CAV1/TGFB1
GO:0050918	positive chemotaxis	2/32	68/18670	0.006042	0.012913	0.003519	FGF2/KDR
GO:0071242	cellular response to ammonium ion	2/32	68/18670	0.006042	0.012913	0.003519	ABL1/MAPK1
GO:0043583	ear development	3/32	219/18670	0.006145	0.013126	0.003577	BCL2/MAPK1/TGFB1

GO:0030166	proteoglycan biosynthetic process	2/32	69/18670	0.006216	0.013258	0.003613	CTNNB1/IGF1
GO:2000243	positive regulation of reproductive process	2/32	69/18670	0.006216	0.013258	0.003613	CTNNB1/SRC
GO:0010256	endomembrane system organization	4/32	438/18670	0.006389	0.013579	0.003701	AKT1/FASLG/CAV1/MAPK1
GO:0032720	negative regulation of tumor necrosis factor production	2/32	70/18670	0.006392	0.013579	0.003701	IGF1/IL10
GO:0034121	regulation of toll-like receptor signaling pathway	2/32	70/18670	0.006392	0.013579	0.003701	CAV1/IRF1
GO:0050766	positive regulation of phagocytosis	2/32	70/18670	0.006392	0.013579	0.003701	IL1B/TNF
GO:0051966	regulation of synaptic transmission, glutamatergic	2/32	70/18670	0.006392	0.013579	0.003701	PTGS2/TNF
GO:0072091	regulation of stem cell proliferation	2/32	70/18670	0.006392	0.013579	0.003701	CTNNB1/TGFB1
GO:0043647	inositol phosphate metabolic process	2/32	71/18670	0.00657	0.01394	0.003799	FGF2/SNCA
GO:0051057	positive regulation of small GTPase mediated signal transduction	2/32	71/18670	0.00657	0.01394	0.003799	IGF1/SRC
GO:0051495	positive regulation of cytoskeleton organization	3/32	226/18670	0.006702	0.014211	0.003873	ABL1/ICAM1/MAPT
GO:0006305	DNA alkylation	2/32	72/18670	0.00675	0.014275	0.00389	PARP1/MYC
GO:0006306	DNA methylation	2/32	72/18670	0.00675	0.014275	0.00389	PARP1/MYC

GO:0061035	regulation of cartilage development	2/32	72/18670	0.00675	0.014275	0.00389	CTNNB1/TGFB1
GO:1903556	negative regulation of tumor necrosis factor superfamily cytokine production	2/32	72/18670	0.00675	0.014275	0.00389	IGF1/IL10
GO:0001894	tissue homeostasis	3/32	227/18670	0.006784	0.014337	0.003907	BCL2/CTNNB1/SRC
GO:0007265	Ras protein signal transduction	4/32	448/18670	0.006911	0.014597	0.003978	ABL1/FGF2/IGF1/JUN
GO:0045685	regulation of glial cell differentiation	2/32	73/18670	0.006933	0.014633	0.003988	CTNNB1/TGFB1
GO:0032507	maintenance of protein location in cell	2/32	74/18670	0.007118	0.014974	0.004081	AKT1/CAV1
GO:0043367	CD4-positive, alpha-beta T cell differentiation	2/32	74/18670	0.007118	0.014974	0.004081	IL2/JAK3
GO:0050710	negative regulation of cytokine secretion	2/32	74/18670	0.007118	0.014974	0.004081	IL10/TNF
GO:0051145	smooth muscle cell differentiation	2/32	74/18670	0.007118	0.014974	0.004081	CTNNB1/TGFB1
GO:1900076	regulation of cellular response to insulin stimulus	2/32	74/18670	0.007118	0.014974	0.004081	IL1B/SRC
GO:0045088	regulation of innate immune response	4/32	452/18670	0.007128	0.014986	0.004084	CASP8/CAV1/IRF1/SRC
GO:0016570	histone modification	4/32	454/18670	0.007238	0.015207	0.004144	CTNNB1/IL1B/SNCA/TGFB1
GO:0007422	peripheral nervous system development	2/32	75/18670	0.007305	0.015308	0.004172	AKT1/ERBB2

GO:0010611	regulation of cardiac muscle hypertrophy	2/32	75/18670	0.007305	0.015308	0.004172	PARP1/IGF1
GO:0046173	polyol biosynthetic process	2/32	75/18670	0.007305	0.015308	0.004172	FGF2/SNCA
GO:1902117	positive regulation of organelle assembly	2/32	75/18670	0.007305	0.015308	0.004172	SRC/TNF
GO:0009755	hormone-mediated signaling pathway	3/32	235/18670	0.007461	0.015624	0.004258	PARP1/CTNNB1/SRC
GO:0001937	negative regulation of endothelial cell proliferation	2/32	76/18670	0.007495	0.015674	0.004271	CAV1/TNF
GO:0007492	endoderm development	2/32	76/18670	0.007495	0.015674	0.004271	CTNNB1/TGFB1
GO:0032481	positive regulation of type I interferon production	2/32	77/18670	0.007687	0.01603	0.004368	CTNNB1/IRF1
GO:0048678	response to axon injury	2/32	77/18670	0.007687	0.01603	0.004368	BCL2/JUN
GO:1901224	positive regulation of NIK/NF-kappaB signaling	2/32	77/18670	0.007687	0.01603	0.004368	IL1B/TNF
GO:0023061	signal release	4/32	462/18670	0.00769	0.01603	0.004368	GSK3B/IL1B/SNCA/TNF
GO:0046394	carboxylic acid biosynthetic process	4/32	462/18670	0.00769	0.01603	0.004368	IGF1/IL1B/PTGS2/TGFB1
GO:0051607	defense response to virus	3/32	238/18670	0.007725	0.016092	0.004385	BCL2/IL1B/IRF1
GO:0016053	organic acid biosynthetic process	4/32	463/18670	0.007748	0.01613	0.004396	IGF1/IL1B/PTGS2/TGFB1

GO:0031348	negative regulation of defense response	3/32	239/18670	0.007814	0.016257	0.00443	IGF1/IL2/IL10
GO:0014743	regulation of muscle hypertrophy	2/32	78/18670	0.00788	0.016352	0.004456	PARP1/IGF1
GO:0021954	central nervous system neuron development	2/32	78/18670	0.00788	0.016352	0.004456	HSP90AA1/MAPT
GO:0033143	regulation of intracellular steroid hormone receptor signaling pathway	2/32	78/18670	0.00788	0.016352	0.004456	PARP1/SRC
GO:0043407	negative regulation of MAP kinase activity	2/32	78/18670	0.00788	0.016352	0.004456	CAV1/IL1B
GO:0003151	outflow tract morphogenesis	2/32	79/18670	0.008076	0.016738	0.004561	CTNNB1/JUN
GO:0043537	negative regulation of blood vessel endothelial cell migration	2/32	79/18670	0.008076	0.016738	0.004561	FGF2/TGFB1
GO:0002088	lens development in camera-type eye	2/32	80/18670	0.008275	0.017138	0.00467	CTNNB1/TGFB1
GO:0016569	covalent chromatin modification	4/32	474/18670	0.008402	0.01739	0.004739	CTNNB1/IL1B/SNCA/TGFB1
GO:0014855	striated muscle cell proliferation	2/32	81/18670	0.008475	0.017519	0.004774	FGF2/MAPK1
GO:0030512	negative regulation of transforming growth factor beta receptor signaling pathway	2/32	81/18670	0.008475	0.017519	0.004774	CAV1/TGFB1
GO:0010498	proteasomal protein catabolic process	4/32	477/18670	0.008587	0.017738	0.004834	AKT1/CAV1/CTNNB1/GSK3B
GO:0043087	regulation of GTPase activity	4/32	479/18670	0.008711	0.017984	0.004901	ERBB2/GSK3B/ICAM1/JUN

GO:0006352	DNA-templated transcription, initiation	3/32	249/18670	0.00874	0.01803	0.004914	CCND1/CTNNB1/JUN
GO:0006970	response to osmotic stress	2/32	83/18670	0.008883	0.01829	0.004984	PTGS2/TNF
GO:0045582	positive regulation of T cell differentiation	2/32	83/18670	0.008883	0.01829	0.004984	IL2/TGFB1
GO:1903845	negative regulation of cellular response to transforming growth factor beta stimulus	2/32	83/18670	0.008883	0.01829	0.004984	CAV1/TGFB1
GO:0051453	regulation of intracellular pH	2/32	84/18670	0.00909	0.018704	0.005097	FASLG/BCL2
GO:0002028	regulation of sodium ion transport	2/32	85/18670	0.009299	0.019098	0.005204	AKT1/TGFB1
GO:0014031	mesenchymal cell development	2/32	85/18670	0.009299	0.019098	0.005204	BCL2/MAPK1
GO:0045682	regulation of epidermis development	2/32	85/18670	0.009299	0.019098	0.005204	CTNNB1/TNF
GO:0030101	natural killer cell activation	2/32	86/18670	0.00951	0.019494	0.005313	CASP8/IL2
GO:0042058	regulation of epidermal growth factor receptor signaling pathway	2/32	86/18670	0.00951	0.019494	0.005313	AKT1/FASLG
GO:0042446	hormone biosynthetic process	2/32	86/18670	0.00951	0.019494	0.005313	IL1B/TNF
GO:0003007	heart morphogenesis	3/32	259/18670	0.009727	0.019926	0.00543	CTNNB1/JUN/TGFB1
GO:0072659	protein localization to plasma membrane	3/32	260/18670	0.009829	0.020123	0.005484	AKT1/TGFB1/TNF

GO:0009108	coenzyme biosynthetic process	3/32	261/18670	0.009932	0.02027	0.005524	IGF1/PTGS2/SNCA
GO:0009791	post-embryonic development	2/32	88/18670	0.009939	0.02027	0.005524	ABL1/BCL2
GO:0045921	positive regulation of exocytosis	2/32	88/18670	0.009939	0.02027	0.005524	IL13/SNCA
GO:0061097	regulation of protein tyrosine kinase activity	2/32	88/18670	0.009939	0.02027	0.005524	CAV1/SRC
GO:0097194	execution phase of apoptosis	2/32	88/18670	0.009939	0.02027	0.005524	AKT1/CASP8
GO:2000177	regulation of neural precursor cell proliferation	2/32	88/18670	0.009939	0.02027	0.005524	CTNNB1/TGFB1
GO:0032410	negative regulation of transporter activity	2/32	89/18670	0.010157	0.020688	0.005638	CAV1/SNCA
GO:0097306	cellular response to alcohol	2/32	89/18670	0.010157	0.020688	0.005638	AKT1/CTNNB1
GO:0001843	neural tube closure	2/32	90/18670	0.010377	0.021095	0.005749	ABL1/TGFB1
GO:0032651	regulation of interleukin-1 beta production	2/32	90/18670	0.010377	0.021095	0.005749	CASP8/IGF1
GO:0071674	mononuclear cell migration	2/32	90/18670	0.010377	0.021095	0.005749	TGFB1/TNF
GO:0045638	negative regulation of myeloid cell differentiation	2/32	91/18670	0.010598	0.021492	0.005857	CTNNB1/MYC
GO:0060333	interferon-gamma-mediated signaling pathway	2/32	91/18670	0.010598	0.021492	0.005857	ICAM1/IRF1

GO:0060606	tube closure	2/32	91/18670	0.010598	0.021492	0.005857	ABL1/TGFB1
GO:1904063	negative regulation of cation transmembrane transport	2/32	91/18670	0.010598	0.021492	0.005857	CAV1/TGFB1
GO:0022898	regulation of transmembrane transporter activity	3/32	268/18670	0.010669	0.021622	0.005892	BCL2/CAV1/SNCA
GO:0035710	CD4-positive, alpha-beta T cell activation	2/32	92/18670	0.010822	0.021918	0.005973	IL2/JAK3
GO:0045165	cell fate commitment	3/32	270/18670	0.010886	0.022033	0.006004	BCL2/CTNNB1/FGF2
GO:0006476	protein deacetylation	2/32	93/18670	0.011049	0.02232	0.006083	MAPT/TGFB1
GO:0007589	body fluid secretion	2/32	93/18670	0.011049	0.02232	0.006083	CCND1/CAV1
GO:0035249	synaptic transmission, glutamatergic	2/32	93/18670	0.011049	0.02232	0.006083	PTGS2/TNF
GO:0061448	connective tissue development	3/32	273/18670	0.011215	0.022643	0.00617	CTNNB1/FGF2/TGFB1
GO:0006029	proteoglycan metabolic process	2/32	94/18670	0.011277	0.022724	0.006193	CTNNB1/IGF1
GO:0044728	DNA methylation or demethylation	2/32	94/18670	0.011277	0.022724	0.006193	PARP1/MYC
GO:0045621	positive regulation of lymphocyte differentiation	2/32	94/18670	0.011277	0.022724	0.006193	IL2/TGFB1
GO:0016054	organic acid catabolic process	3/32	275/18670	0.011438	0.02302	0.006273	AKT1/FGF2/TGFB1

GO:0046395	carboxylic acid catabolic process	3/32	275/18670	0.011438	0.02302	0.006273	AKT1/FGF2/TGFB1
GO:0045069	regulation of viral genome replication	2/32	95/18670	0.011507	0.023101	0.006295	BCL2/TNF
GO:0050810	regulation of steroid biosynthetic process	2/32	95/18670	0.011507	0.023101	0.006295	IL1B/TNF
GO:0051196	regulation of coenzyme metabolic process	2/32	95/18670	0.011507	0.023101	0.006295	IGF1/SNCA
GO:0051651	maintenance of location in cell	2/32	95/18670	0.011507	0.023101	0.006295	AKT1/CAV1
GO:0007411	axon guidance	3/32	276/18670	0.01155	0.023173	0.006315	ERBB2/MAPK1/SRC
GO:0097485	neuron projection guidance	3/32	277/18670	0.011664	0.023385	0.006373	ERBB2/MAPK1/SRC
GO:0032755	positive regulation of interleukin-6 production	2/32	96/18670	0.011739	0.023508	0.006406	IL1B/TNF
GO:2001169	regulation of ATP biosynthetic process	2/32	96/18670	0.011739	0.023508	0.006406	PARP1/IGF1
GO:0010596	negative regulation of endothelial cell migration	2/32	97/18670	0.011974	0.023903	0.006514	FGF2/TGFB1
GO:0014020	primary neural tube formation	2/32	97/18670	0.011974	0.023903	0.006514	ABL1/TGFB1
GO:0015908	fatty acid transport	2/32	97/18670	0.011974	0.023903	0.006514	AKT1/IL1B
GO:0120162	positive regulation of cold-induced thermogenesis	2/32	97/18670	0.011974	0.023903	0.006514	CAV1/IL13

GO:2001022	positive regulation of response to DNA damage stimulus	2/32	97/18670	0.011974	0.023903	0.006514	PARP1/MYC
GO:0030038	contractile actin filament bundle assembly	2/32	98/18670	0.01221	0.02433	0.00663	ABL1/SRC
GO:0031341	regulation of cell killing	2/32	98/18670	0.01221	0.02433	0.00663	ICAM1/IL13
GO:0043149	stress fiber assembly	2/32	98/18670	0.01221	0.02433	0.00663	ABL1/SRC
GO:0032409	regulation of transporter activity	3/32	283/18670	0.012355	0.024603	0.006705	BCL2/CAV1/SNCA
GO:0001708	cell fate specification	2/32	99/18670	0.012449	0.024743	0.006743	CTNNB1/FGF2
GO:0048525	negative regulation of viral process	2/32	99/18670	0.012449	0.024743	0.006743	JUN/TNF
GO:1901570	fatty acid derivative biosynthetic process	2/32	99/18670	0.012449	0.024743	0.006743	IL1B/PTGS2
GO:0021700	developmental maturation	3/32	284/18670	0.012473	0.024775	0.006752	BCL2/CTNNB1/IGF1
GO:0034446	substrate adhesion-dependent cell spreading	2/32	100/18670	0.012689	0.025159	0.006856	ABL1/SRC
GO:0070498	interleukin-1-mediated signaling pathway	2/32	100/18670	0.012689	0.025159	0.006856	IL1A/IL1B
GO:2001023	regulation of response to drug	2/32	100/18670	0.012689	0.025159	0.006856	IL10/SNCA
GO:0031532	actin cytoskeleton reorganization	2/32	101/18670	0.012932	0.025624	0.006983	ABL1/TGFB1

GO:0098869	cellular oxidant detoxification	2/32	102/18670	0.013177	0.02609	0.00711	PTGS2/TNF
GO:0003015	heart process	3/32	290/18670	0.013191	0.02609	0.00711	CAV1/IL2/SRC
GO:0051650	establishment of vesicle localization	3/32	290/18670	0.013191	0.02609	0.00711	CTNNB1/GSK3B/SNCA
GO:0007229	integrin-mediated signaling pathway	2/32	103/18670	0.013423	0.026499	0.007222	ABL1/SRC
GO:0018958	phenol-containing compound metabolic process	2/32	103/18670	0.013423	0.026499	0.007222	BCL2/SNCA
GO:0035601	protein deacylation	2/32	103/18670	0.013423	0.026499	0.007222	MAPT/TGFB1
GO:0009410	response to xenobiotic stimulus	3/32	292/18670	0.013436	0.026508	0.007224	ICAM1/SNCA/TGFB1
GO:0010923	negative regulation of phosphatase activity	2/32	104/18670	0.013672	0.026908	0.007333	GSK3B/TNF
GO:0046928	regulation of neurotransmitter secretion	2/32	104/18670	0.013672	0.026908	0.007333	GSK3B/SNCA
GO:0090100	positive regulation of transmembrane receptor protein serine/threonine kinase signaling pathway	2/32	104/18670	0.013672	0.026908	0.007333	PARP1/TGFB1
GO:0098732	macromolecule deacylation	2/32	104/18670	0.013672	0.026908	0.007333	MAPT/TGFB1
GO:0016236	macroautophagy	3/32	295/18670	0.013808	0.027142	0.007397	AKT1/KDR/SRC
GO:0048511	rhythmic process	3/32	295/18670	0.013808	0.027142	0.007397	GSK3B/JUN/SRC

GO:0001841	neural tube formation	2/32	105/18670	0.013923	0.027334	0.007449	ABL1/TGFB1
GO:0007009	plasma membrane organization	2/32	105/18670	0.013923	0.027334	0.007449	AKT1/CAV1
GO:0002456	T cell mediated immunity	2/32	106/18670	0.014175	0.027796	0.007575	ICAM1/IL1B
GO:0003300	cardiac muscle hypertrophy	2/32	106/18670	0.014175	0.027796	0.007575	PARP1/IGF1
GO:0043502	regulation of muscle adaptation	2/32	107/18670	0.01443	0.028278	0.007706	PARP1/IGF1
GO:0010769	regulation of cell morphogenesis involved in differentiation	3/32	301/18670	0.01457	0.028534	0.007776	ABL1/GSK3B/MAPT
GO:0071156	regulation of cell cycle arrest	2/32	108/18670	0.014687	0.028746	0.007834	CCND1/TGFB1
GO:0014897	striated muscle hypertrophy	2/32	109/18670	0.014945	0.029234	0.007967	PARP1/IGF1
GO:0006024	glycosaminoglycan biosynthetic process	2/32	110/18670	0.015206	0.02969	0.008091	IL1B/TGFB1
GO:0006939	smooth muscle contraction	2/32	110/18670	0.015206	0.02969	0.008091	CAV1/PTGS2
GO:0033559	unsaturated fatty acid metabolic process	2/32	110/18670	0.015206	0.02969	0.008091	IL1B/PTGS2
GO:0051648	vesicle localization	3/32	306/18670	0.015223	0.029704	0.008095	CTNNB1/GSK3B/SNCA
GO:0014896	muscle hypertrophy	2/32	111/18670	0.015469	0.030092	0.008201	PARP1/IGF1

GO:0022037	metencephalon development	2/32	111/18670	0.015469	0.030092	0.008201	ABL1/BCL2
GO:0031623	receptor internalization	2/32	111/18670	0.015469	0.030092	0.008201	CAV1/SNCA
GO:0035305	negative regulation of dephosphorylation	2/32	111/18670	0.015469	0.030092	0.008201	GSK3B/TNF
GO:0072676	lymphocyte migration	2/32	111/18670	0.015469	0.030092	0.008201	AKT1/ICAM1
GO:0043414	macromolecule methylation	3/32	309/18670	0.015622	0.030373	0.008277	PARP1/CTNNB1/MYC
GO:0014902	myotube differentiation	2/32	112/18670	0.015733	0.030515	0.008316	BCL2/IGF1
GO:0043279	response to alkaloid	2/32	112/18670	0.015733	0.030515	0.008316	ICAM1/SNCA
GO:0098693	regulation of synaptic vesicle cycle	2/32	112/18670	0.015733	0.030515	0.008316	GSK3B/SNCA
GO:1990748	cellular detoxification	2/32	112/18670	0.015733	0.030515	0.008316	PTGS2/TNF
GO:0043200	response to amino acid	2/32	113/18670	0.016	0.031013	0.008451	ICAM1/TNF
GO:0006690	icosanoid metabolic process	2/32	114/18670	0.016268	0.031495	0.008583	IL1B/PTGS2
GO:0010633	negative regulation of epithelial cell migration	2/32	114/18670	0.016268	0.031495	0.008583	FGF2/TGFB1
GO:0043010	camera-type eye development	3/32	314/18670	0.016301	0.03154	0.008595	CTNNB1/JUN/TGFB1

GO:0006023	aminoglycan biosynthetic process	2/32	115/18670	0.016539	0.03184	0.008677	IL1B/TGFB1
GO:0006898	receptor-mediated endocytosis	3/32	316/18670	0.016578	0.03184	0.008677	CAV1/HSP90AA1/SNCA
GO:0050851	antigen receptor-mediated signaling pathway	3/32	316/18670	0.016578	0.03184	0.008677	ABL1/BCL2/MAPK1
GO:0006304	DNA modification	2/32	116/18670	0.016811	0.03184	0.008677	PARP1/MYC
GO:0021987	cerebral cortex development	2/32	116/18670	0.016811	0.03184	0.008677	CTNNB1/GSK3B
GO:0000012	single strand break repair	1/32	10/18670	0.017012	0.03184	0.008677	PARP1
GO:0001840	neural plate development	1/32	10/18670	0.017012	0.03184	0.008677	CTNNB1
GO:0002291	T cell activation via T cell receptor contact with antigen bound to MHC molecule on antigen presenting cell	1/32	10/18670	0.017012	0.03184	0.008677	ICAM1
GO:0007440	foregut morphogenesis	1/32	10/18670	0.017012	0.03184	0.008677	CTNNB1
GO:0010918	positive regulation of mitochondrial membrane potential	1/32	10/18670	0.017012	0.03184	0.008677	AKT1
GO:0014041	regulation of neuron maturation	1/32	10/18670	0.017012	0.03184	0.008677	BCL2
GO:0019062	virion attachment to host cell	1/32	10/18670	0.017012	0.03184	0.008677	ICAM1
GO:0021548	pons development	1/32	10/18670	0.017012	0.03184	0.008677	BCL2

GO:0032025	response to cobalt ion	1/32	10/18670	0.017012	0.03184	0.008677	CASP8
GO:0032070	regulation of deoxyribonuclease activity	1/32	10/18670	0.017012	0.03184	0.008677	AKT1
GO:0032308	positive regulation of prostaglandin secretion	1/32	10/18670	0.017012	0.03184	0.008677	IL1B
GO:0032463	negative regulation of protein homooligomerization	1/32	10/18670	0.017012	0.03184	0.008677	SRC
GO:0032621	interleukin-18 production	1/32	10/18670	0.017012	0.03184	0.008677	IL10
GO:0033483	gas homeostasis	1/32	10/18670	0.017012	0.03184	0.008677	CAV1
GO:0033690	positive regulation of osteoblast proliferation	1/32	10/18670	0.017012	0.03184	0.008677	ABL1
GO:0035768	endothelial cell chemotaxis to fibroblast growth factor	1/32	10/18670	0.017012	0.03184	0.008677	FGF2
GO:0042536	negative regulation of tumor necrosis factor biosynthetic process	1/32	10/18670	0.017012	0.03184	0.008677	IL10
GO:0044359	modulation of molecular function in other organism	1/32	10/18670	0.017012	0.03184	0.008677	CASP8
GO:0045348	positive regulation of MHC class II biosynthetic process	1/32	10/18670	0.017012	0.03184	0.008677	IL10
GO:0045625	regulation of T-helper 1 cell differentiation	1/32	10/18670	0.017012	0.03184	0.008677	JAK3
GO:0045657	positive regulation of monocyte differentiation	1/32	10/18670	0.017012	0.03184	0.008677	JUN

GO:0045792	negative regulation of cell size	1/32	10/18670	0.017012	0.03184	0.008677	AKT1
GO:0046322	negative regulation of fatty acid oxidation	1/32	10/18670	0.017012	0.03184	0.008677	AKT1
GO:0048262	determination of dorsal/ventral asymmetry	1/32	10/18670	0.017012	0.03184	0.008677	CTNNB1
GO:0048304	positive regulation of isotype switching to IgG isotypes	1/32	10/18670	0.017012	0.03184	0.008677	IL2
GO:0052205	modulation of molecular function in other organism involved in symbiotic interaction	1/32	10/18670	0.017012	0.03184	0.008677	CASP8
GO:0060281	regulation of oocyte development	1/32	10/18670	0.017012	0.03184	0.008677	IGF1
GO:0060768	regulation of epithelial cell proliferation involved in prostate gland development	1/32	10/18670	0.017012	0.03184	0.008677	CTNNB1
GO:0061140	lung secretory cell differentiation	1/32	10/18670	0.017012	0.03184	0.008677	IL13
GO:0071104	response to interleukin-9	1/32	10/18670	0.017012	0.03184	0.008677	JAK3
GO:0071609	chemokine (C-C motif) ligand 5 production	1/32	10/18670	0.017012	0.03184	0.008677	IL10
GO:0072203	cell proliferation involved in metanephros development	1/32	10/18670	0.017012	0.03184	0.008677	MYC
GO:0090154	positive regulation of sphingolipid biosynthetic process	1/32	10/18670	0.017012	0.03184	0.008677	TNF
GO:0090336	positive regulation of brown fat cell differentiation	1/32	10/18670	0.017012	0.03184	0.008677	PTGS2

GO:0090557	establishment of endothelial intestinal barrier	1/32	10/18670	0.017012	0.03184	0.008677	ICAM1
GO:0097278	complement-dependent cytotoxicity	1/32	10/18670	0.017012	0.03184	0.008677	IL13
GO:0106049	regulation of cellular response to osmotic stress	1/32	10/18670	0.017012	0.03184	0.008677	PTGS2
GO:0140052	cellular response to oxidised low-density lipoprotein particle stimulus	1/32	10/18670	0.017012	0.03184	0.008677	AKT1
GO:1900222	negative regulation of amyloid-beta clearance	1/32	10/18670	0.017012	0.03184	0.008677	TNF
GO:1901033	positive regulation of response to reactive oxygen species	1/32	10/18670	0.017012	0.03184	0.008677	TNF
GO:1903799	negative regulation of production of miRNAs involved in gene silencing by miRNA	1/32	10/18670	0.017012	0.03184	0.008677	TGFB1
GO:1903800	positive regulation of production of miRNAs involved in gene silencing by miRNA	1/32	10/18670	0.017012	0.03184	0.008677	TGFB1
GO:1904338	regulation of dopaminergic neuron differentiation	1/32	10/18670	0.017012	0.03184	0.008677	GSK3B
GO:1904526	regulation of microtubule binding	1/32	10/18670	0.017012	0.03184	0.008677	ABL1
GO:1904779	regulation of protein localization to centrosome	1/32	10/18670	0.017012	0.03184	0.008677	GSK3B
GO:1905879	regulation of oogenesis	1/32	10/18670	0.017012	0.03184	0.008677	IGF1
GO:1990416	cellular response to brain-derived neurotrophic factor stimulus	1/32	10/18670	0.017012	0.03184	0.008677	MAPT

GO:2000304	positive regulation of ceramide biosynthetic process	1/32	10/18670	0.017012	0.03184	0.008677	TNF
GO:2000317	negative regulation of T-helper 17 type immune response	1/32	10/18670	0.017012	0.03184	0.008677	IL2
GO:2000544	regulation of endothelial cell chemotaxis to fibroblast growth factor	1/32	10/18670	0.017012	0.03184	0.008677	FGF2
GO:2001214	positive regulation of vasculogenesis	1/32	10/18670	0.017012	0.03184	0.008677	KDR
GO:0007613	memory	2/32	117/18670	0.017085	0.031901	0.008694	MAPT/PTGS2
GO:0010508	positive regulation of autophagy	2/32	117/18670	0.017085	0.031901	0.008694	GSK3B/KDR
GO:0043666	regulation of phosphoprotein phosphatase activity	2/32	117/18670	0.017085	0.031901	0.008694	GSK3B/TNF
GO:1901222	regulation of NIK/NF-kappaB signaling	2/32	117/18670	0.017085	0.031901	0.008694	IL1B/TNF
GO:0016079	synaptic vesicle exocytosis	2/32	119/18670	0.017639	0.032898	0.008965	GSK3B/SNCA
GO:0051193	regulation of cofactor metabolic process	2/32	119/18670	0.017639	0.032898	0.008965	IGF1/SNCA
GO:0017015	regulation of transforming growth factor beta receptor signaling pathway	2/32	120/18670	0.017919	0.033401	0.009102	CAV1/TGFB1
GO:0051188	cofactor biosynthetic process	3/32	326/18670	0.017999	0.033529	0.009137	IGF1/PTGS2/SNCA
GO:0046718	viral entry into host cell	2/32	121/18670	0.018201	0.033887	0.009235	CAV1/ICAM1

GO:0019079	viral genome replication	2/32	122/18670	0.018485	0.033944	0.00925	BCL2/TNF
GO:1903844	regulation of cellular response to transforming growth factor beta stimulus	2/32	122/18670	0.018485	0.033944	0.00925	CAV1/TGFB1
GO:0000185	activation of MAPKKK activity	1/32	11/18670	0.018698	0.033944	0.00925	TNF
GO:0002730	regulation of dendritic cell cytokine production	1/32	11/18670	0.018698	0.033944	0.00925	JAK3
GO:0002923	regulation of humoral immune response mediated by circulating immunoglobulin	1/32	11/18670	0.018698	0.033944	0.00925	TNF
GO:0003264	regulation of cardioblast proliferation	1/32	11/18670	0.018698	0.033944	0.00925	CTNNB1
GO:0009950	dorsal/ventral axis specification	1/32	11/18670	0.018698	0.033944	0.00925	CTNNB1
GO:0016264	gap junction assembly	1/32	11/18670	0.018698	0.033944	0.00925	CAV1
GO:0019371	cyclooxygenase pathway	1/32	11/18670	0.018698	0.033944	0.00925	PTGS2
GO:0031915	positive regulation of synaptic plasticity	1/32	11/18670	0.018698	0.033944	0.00925	PTGS2
GO:0032306	regulation of prostaglandin secretion	1/32	11/18670	0.018698	0.033944	0.00925	IL1B
GO:0033148	positive regulation of intracellular estrogen receptor signaling pathway	1/32	11/18670	0.018698	0.033944	0.00925	PARP1
GO:0033234	negative regulation of protein sumoylation	1/32	11/18670	0.018698	0.033944	0.00925	CTNNB1

GO:0033327	Leydig cell differentiation	1/32	11/18670	0.018698	0.033944	0.00925	CCND1
GO:0033860	regulation of NAD(P)H oxidase activity	1/32	11/18670	0.018698	0.033944	0.00925	IL13
GO:0034115	negative regulation of heterotypic cell-cell adhesion	1/32	11/18670	0.018698	0.033944	0.00925	IL10
GO:0035766	cell chemotaxis to fibroblast growth factor	1/32	11/18670	0.018698	0.033944	0.00925	FGF2
GO:0036462	TRAIL-activated apoptotic signaling pathway	1/32	11/18670	0.018698	0.033944	0.00925	CASP8
GO:0042482	positive regulation of odontogenesis	1/32	11/18670	0.018698	0.033944	0.00925	TGFB1
GO:0043117	positive regulation of vascular permeability	1/32	11/18670	0.018698	0.033944	0.00925	TGFB1
GO:0045351	type I interferon biosynthetic process	1/32	11/18670	0.018698	0.033944	0.00925	IL10
GO:0048096	chromatin-mediated maintenance of transcription	1/32	11/18670	0.018698	0.033944	0.00925	CTNNB1
GO:0048548	regulation of pinocytosis	1/32	11/18670	0.018698	0.033944	0.00925	CAV1
GO:0048742	regulation of skeletal muscle fiber development	1/32	11/18670	0.018698	0.033944	0.00925	BCL2
GO:0051024	positive regulation of immunoglobulin secretion	1/32	11/18670	0.018698	0.033944	0.00925	IL2
GO:0051583	dopamine uptake involved in synaptic transmission	1/32	11/18670	0.018698	0.033944	0.00925	SNCA

GO:0051934	catecholamine uptake involved in synaptic transmission	1/32	11/18670	0.018698	0.033944	0.00925	SNCA
GO:0051974	negative regulation of telomerase activity	1/32	11/18670	0.018698	0.033944	0.00925	SRC
GO:0060272	embryonic skeletal joint morphogenesis	1/32	11/18670	0.018698	0.033944	0.00925	CTNNB1
GO:0060433	bronchus development	1/32	11/18670	0.018698	0.033944	0.00925	IL13
GO:0060767	epithelial cell proliferation involved in prostate gland development	1/32	11/18670	0.018698	0.033944	0.00925	CTNNB1
GO:0060856	establishment of blood-brain barrier	1/32	11/18670	0.018698	0.033944	0.00925	CTNNB1
GO:0061307	cardiac neural crest cell differentiation involved in heart development	1/32	11/18670	0.018698	0.033944	0.00925	MAPK1
GO:0061308	cardiac neural crest cell development involved in heart development	1/32	11/18670	0.018698	0.033944	0.00925	MAPK1
GO:0070213	protein auto-ADP-ribosylation	1/32	11/18670	0.018698	0.033944	0.00925	PARP1
GO:0070587	regulation of cell-cell adhesion involved in gastrulation	1/32	11/18670	0.018698	0.033944	0.00925	IL10
GO:0070673	response to interleukin-18	1/32	11/18670	0.018698	0.033944	0.00925	AKT1
GO:0072683	T cell extravasation	1/32	11/18670	0.018698	0.033944	0.00925	ICAM1
GO:0090042	tubulin deacetylation	1/32	11/18670	0.018698	0.033944	0.00925	MAPT

GO:1901201	regulation of extracellular matrix assembly	1/32	11/18670	0.018698	0.033944	0.00925	TGFB1
GO:1904847	regulation of cell chemotaxis to fibroblast growth factor	1/32	11/18670	0.018698	0.033944	0.00925	FGF2
GO:1905941	positive regulation of gonad development	1/32	11/18670	0.018698	0.033944	0.00925	SRC
GO:2000392	regulation of lamellipodium morphogenesis	1/32	11/18670	0.018698	0.033944	0.00925	SRC
GO:2001269	positive regulation of cysteine-type endopeptidase activity involved in apoptotic signaling pathway	1/32	11/18670	0.018698	0.033944	0.00925	CASP8
GO:0002062	chondrocyte differentiation	2/32	123/18670	0.018771	0.034057	0.009281	CTNNB1/TGFB1
GO:0015849	organic acid transport	3/32	333/18670	0.019033	0.034442	0.009386	AKT1/IL1B/SNCA
GO:0016042	lipid catabolic process	3/32	333/18670	0.019033	0.034442	0.009386	AKT1/IL1B/TNF
GO:0046942	carboxylic acid transport	3/32	333/18670	0.019033	0.034442	0.009386	AKT1/IL1B/SNCA
GO:0007043	cell-cell junction assembly	2/32	124/18670	0.019058	0.034442	0.009386	CAV1/TNF
GO:0019218	regulation of steroid metabolic process	2/32	124/18670	0.019058	0.034442	0.009386	IL1B/TNF
GO:0019751	polyol metabolic process	2/32	124/18670	0.019058	0.034442	0.009386	FGF2/SNCA
GO:0046717	acid secretion	2/32	124/18670	0.019058	0.034442	0.009386	IL1B/SNCA

GO:2000134	negative regulation of G1/S transition of mitotic cell cycle	2/32	125/18670	0.019347	0.034945	0.009523	CCND1/BCL2
GO:0051056	regulation of small GTPase mediated signal transduction	3/32	338/18670	0.019792	0.035728	0.009736	ABL1/IGF1/SRC
GO:0032368	regulation of lipid transport	2/32	127/18670	0.019932	0.03596	0.0098	AKT1/IL1B
GO:0002576	platelet degranulation	2/32	128/18670	0.020227	0.036001	0.009811	IGF1/TGFB1
GO:0002371	dendritic cell cytokine production	1/32	12/18670	0.020381	0.036001	0.009811	JAK3
GO:0002862	negative regulation of inflammatory response to antigenic stimulus	1/32	12/18670	0.020381	0.036001	0.009811	IL10
GO:0002863	positive regulation of inflammatory response to antigenic stimulus	1/32	12/18670	0.020381	0.036001	0.009811	TNF
GO:0002903	negative regulation of B cell apoptotic process	1/32	12/18670	0.020381	0.036001	0.009811	IL2
GO:0003337	mesenchymal to epithelial transition involved in metanephros morphogenesis	1/32	12/18670	0.020381	0.036001	0.009811	CTNNB1
GO:0006983	ER overload response	1/32	12/18670	0.020381	0.036001	0.009811	GSK3B
GO:0010649	regulation of cell communication by electrical coupling	1/32	12/18670	0.020381	0.036001	0.009811	CAV1
GO:0021819	layer formation in cerebral cortex	1/32	12/18670	0.020381	0.036001	0.009811	CTNNB1
GO:0030002	cellular anion homeostasis	1/32	12/18670	0.020381	0.036001	0.009811	FASLG

GO:0030320	cellular monovalent inorganic anion homeostasis	1/32	12/18670	0.020381	0.036001	0.009811	FASLG
GO:0033197	response to vitamin E	1/32	12/18670	0.020381	0.036001	0.009811	CCND1
GO:0035630	bone mineralization involved in bone maturation	1/32	12/18670	0.020381	0.036001	0.009811	IGF1
GO:0042416	dopamine biosynthetic process	1/32	12/18670	0.020381	0.036001	0.009811	SNCA
GO:0042659	regulation of cell fate specification	1/32	12/18670	0.020381	0.036001	0.009811	FGF2
GO:0043922	negative regulation by host of viral transcription	1/32	12/18670	0.020381	0.036001	0.009811	JUN
GO:0044650	adhesion of symbiont to host cell	1/32	12/18670	0.020381	0.036001	0.009811	ICAM1
GO:0045416	positive regulation of interleukin-8 biosynthetic process	1/32	12/18670	0.020381	0.036001	0.009811	TNF
GO:0048302	regulation of isotype switching to IgG isotypes	1/32	12/18670	0.020381	0.036001	0.009811	IL2
GO:0048642	negative regulation of skeletal muscle tissue development	1/32	12/18670	0.020381	0.036001	0.009811	TGFB1
GO:0051798	positive regulation of hair follicle development	1/32	12/18670	0.020381	0.036001	0.009811	TNF
GO:0060670	branching involved in labyrinthine layer morphogenesis	1/32	12/18670	0.020381	0.036001	0.009811	IL10
GO:0060742	epithelial cell differentiation involved in prostate gland development	1/32	12/18670	0.020381	0.036001	0.009811	CTNNB1

GO:0060788	ectodermal placode formation	1/32	12/18670	0.020381	0.036001	0.009811	CTNNB1
GO:0070243	regulation of thymocyte apoptotic process	1/32	12/18670	0.020381	0.036001	0.009811	JAK3
GO:0070486	leukocyte aggregation	1/32	12/18670	0.020381	0.036001	0.009811	IL1B
GO:0070493	thrombin-activated receptor signaling pathway	1/32	12/18670	0.020381	0.036001	0.009811	SNCA
GO:0070586	cell-cell adhesion involved in gastrulation	1/32	12/18670	0.020381	0.036001	0.009811	IL10
GO:0070601	centromeric sister chromatid cohesion	1/32	12/18670	0.020381	0.036001	0.009811	CTNNB1
GO:0071639	positive regulation of monocyte chemotactic protein-1 production	1/32	12/18670	0.020381	0.036001	0.009811	IL1B
GO:0071697	ectodermal placode morphogenesis	1/32	12/18670	0.020381	0.036001	0.009811	CTNNB1
GO:0097201	negative regulation of transcription from RNA polymerase II promoter in response to stress	1/32	12/18670	0.020381	0.036001	0.009811	JUN
GO:0098962	regulation of postsynaptic neurotransmitter receptor activity	1/32	12/18670	0.020381	0.036001	0.009811	SRC
GO:1903909	regulation of receptor clustering	1/32	12/18670	0.020381	0.036001	0.009811	TGFB1
GO:1904953	Wnt signaling pathway involved in midbrain dopaminergic neuron differentiation	1/32	12/18670	0.020381	0.036001	0.009811	CTNNB1
GO:1905244	regulation of modification of synaptic structure	1/32	12/18670	0.020381	0.036001	0.009811	ABL1

GO:2000121	regulation of removal of superoxide radicals	1/32	12/18670	0.020381	0.036001	0.009811	TNF
GO:2000341	regulation of chemokine (C-X-C motif) ligand 2 production	1/32	12/18670	0.020381	0.036001	0.009811	TNF
GO:0032956	regulation of actin cytoskeleton organization	3/32	343/18670	0.020567	0.03629	0.00989	ABL1/ICAM1/TGFB1
GO:0072330	monocarboxylic acid biosynthetic process	3/32	343/18670	0.020567	0.03629	0.00989	IGF1/IL1B/PTGS2
GO:0006638	neutral lipid metabolic process	2/32	130/18670	0.020822	0.036719	0.010007	CAV1/SNCA
GO:0098754	detoxification	2/32	131/18670	0.021123	0.037208	0.01014	PTGS2/TNF
GO:1902807	negative regulation of cell cycle G1/S phase transition	2/32	131/18670	0.021123	0.037208	0.01014	CCND1/BCL2
GO:0042177	negative regulation of protein catabolic process	2/32	132/18670	0.021425	0.037699	0.010274	IL10/SNCA
GO:0055007	cardiac muscle cell differentiation	2/32	132/18670	0.021425	0.037699	0.010274	IGF1/TGFB1
GO:0002920	regulation of humoral immune response	2/32	134/18670	0.022035	0.038067	0.010374	IL1B/TNF
GO:0006766	vitamin metabolic process	2/32	134/18670	0.022035	0.038067	0.010374	IL1B/TNF
GO:0001765	membrane raft assembly	1/32	13/18670	0.022061	0.038067	0.010374	CAV1
GO:0003306	Wnt signaling pathway involved in heart development	1/32	13/18670	0.022061	0.038067	0.010374	CTNNB1

GO:0010642	negative regulation of platelet-derived growth factor receptor signaling pathway	1/32	13/18670	0.022061	0.038067	0.010374	SNCA
GO:0014842	regulation of skeletal muscle satellite cell proliferation	1/32	13/18670	0.022061	0.038067	0.010374	FGF2
GO:0019896	axonal transport of mitochondrion	1/32	13/18670	0.022061	0.038067	0.010374	MAPT
GO:0031115	negative regulation of microtubule polymerization	1/32	13/18670	0.022061	0.038067	0.010374	SNCA
GO:0032488	Cdc42 protein signal transduction	1/32	13/18670	0.022061	0.038067	0.010374	ABL1
GO:0032725	positive regulation of granulocyte macrophage colony-stimulating factor production	1/32	13/18670	0.022061	0.038067	0.010374	IL1B
GO:0033127	regulation of histone phosphorylation	1/32	13/18670	0.022061	0.038067	0.010374	IL1B
GO:0033623	regulation of integrin activation	1/32	13/18670	0.022061	0.038067	0.010374	SRC
GO:0035723	interleukin-15-mediated signaling pathway	1/32	13/18670	0.022061	0.038067	0.010374	JAK3
GO:0042532	negative regulation of tyrosine phosphorylation of STAT protein	1/32	13/18670	0.022061	0.038067	0.010374	CAV1
GO:0042635	positive regulation of hair cycle	1/32	13/18670	0.022061	0.038067	0.010374	TNF
GO:0043568	positive regulation of insulin-like growth factor receptor signaling pathway	1/32	13/18670	0.022061	0.038067	0.010374	IGF1
GO:0047484	regulation of response to osmotic stress	1/32	13/18670	0.022061	0.038067	0.010374	PTGS2

GO:0048291	isotype switching to IgG isotypes	1/32	13/18670	0.022061	0.038067	0.010374	IL2
GO:0048715	negative regulation of oligodendrocyte differentiation	1/32	13/18670	0.022061	0.038067	0.010374	CTNNB1
GO:0051001	negative regulation of nitric-oxide synthase activity	1/32	13/18670	0.022061	0.038067	0.010374	CAV1
GO:0060312	regulation of blood vessel remodeling	1/32	13/18670	0.022061	0.038067	0.010374	TGFB1
GO:0060712	spongiotrophoblast layer development	1/32	13/18670	0.022061	0.038067	0.010374	AKT1
GO:0061029	eyelid development in camera-type eye	1/32	13/18670	0.022061	0.038067	0.010374	JUN
GO:0071350	cellular response to interleukin-15	1/32	13/18670	0.022061	0.038067	0.010374	JAK3
GO:0071696	ectodermal placode development	1/32	13/18670	0.022061	0.038067	0.010374	CTNNB1
GO:0071872	cellular response to epinephrine stimulus	1/32	13/18670	0.022061	0.038067	0.010374	SNCA
GO:0072182	regulation of nephron tubule epithelial cell differentiation	1/32	13/18670	0.022061	0.038067	0.010374	CTNNB1
GO:0098903	regulation of membrane repolarization during action potential	1/32	13/18670	0.022061	0.038067	0.010374	CAV1
GO:1900272	negative regulation of long-term synaptic potentiation	1/32	13/18670	0.022061	0.038067	0.010374	ABL1
GO:1902430	negative regulation of amyloid-beta formation	1/32	13/18670	0.022061	0.038067	0.010374	IGF1

GO:1902713	regulation of interferon-gamma secretion	1/32	13/18670	0.022061	0.038067	0.010374	ABL1
GO:1902947	regulation of tau-protein kinase activity	1/32	13/18670	0.022061	0.038067	0.010374	HSP90AA1
GO:1903960	negative regulation of anion transmembrane transport	1/32	13/18670	0.022061	0.038067	0.010374	AKT1
GO:1990403	embryonic brain development	1/32	13/18670	0.022061	0.038067	0.010374	CTNNB1
GO:2000052	positive regulation of non-canonical Wnt signaling pathway	1/32	13/18670	0.022061	0.038067	0.010374	ABL1
GO:2000194	regulation of female gonad development	1/32	13/18670	0.022061	0.038067	0.010374	SRC
GO:0030010	establishment of cell polarity	2/32	135/18670	0.022343	0.038532	0.0105	GSK3B/HSP90AA1
GO:0006959	humoral immune response	3/32	356/18670	0.022662	0.039061	0.010645	BCL2/IL1B/TNF
GO:0031644	regulation of neurological system process	2/32	137/18670	0.022963	0.03956	0.010781	IL10/SRC
GO:0051588	regulation of neurotransmitter transport	2/32	139/18670	0.023591	0.040161	0.010945	GSK3B/SNCA
GO:0010457	centriole-centriole cohesion	1/32	14/18670	0.023738	0.040161	0.010945	CTNNB1
GO:0010917	negative regulation of mitochondrial membrane potential	1/32	14/18670	0.023738	0.040161	0.010945	MAPT
GO:0010935	regulation of macrophage cytokine production	1/32	14/18670	0.023738	0.040161	0.010945	TGFB1

GO:0014841	skeletal muscle satellite cell proliferation	1/32	14/18670	0.023738	0.040161	0.010945	FGF2
GO:0014854	response to inactivity	1/32	14/18670	0.023738	0.040161	0.010945	IL10
GO:0014857	regulation of skeletal muscle cell proliferation	1/32	14/18670	0.023738	0.040161	0.010945	FGF2
GO:0021781	glial cell fate commitment	1/32	14/18670	0.023738	0.040161	0.010945	CTNNB1
GO:0032310	prostaglandin secretion	1/32	14/18670	0.023738	0.040161	0.010945	IL1B
GO:0034356	NAD biosynthesis via nicotinamide riboside salvage pathway	1/32	14/18670	0.023738	0.040161	0.010945	PTGS2
GO:0034392	negative regulation of smooth muscle cell apoptotic process	1/32	14/18670	0.023738	0.040161	0.010945	IGF1
GO:0035112	genitalia morphogenesis	1/32	14/18670	0.023738	0.040161	0.010945	CTNNB1
GO:0036295	cellular response to increased oxygen levels	1/32	14/18670	0.023738	0.040161	0.010945	CAV1
GO:0045346	regulation of MHC class II biosynthetic process	1/32	14/18670	0.023738	0.040161	0.010945	IL10
GO:0048308	organelle inheritance	1/32	14/18670	0.023738	0.040161	0.010945	MAPK1
GO:0048313	Golgi inheritance	1/32	14/18670	0.023738	0.040161	0.010945	MAPK1
GO:0055064	chloride ion homeostasis	1/32	14/18670	0.023738	0.040161	0.010945	FASLG

GO:0060009	Sertoli cell development	1/32	14/18670	0.023738	0.040161	0.010945	ICAM1
GO:0060397	JAK-STAT cascade involved in growth hormone signaling pathway	1/32	14/18670	0.023738	0.040161	0.010945	JAK3
GO:0060732	positive regulation of inositol phosphate biosynthetic process	1/32	14/18670	0.023738	0.040161	0.010945	SNCA
GO:0061051	positive regulation of cell growth involved in cardiac muscle cell development	1/32	14/18670	0.023738	0.040161	0.010945	IGF1
GO:0070672	response to interleukin-15	1/32	14/18670	0.023738	0.040161	0.010945	JAK3
GO:0070885	negative regulation of calcineurin-NFAT signaling cascade	1/32	14/18670	0.023738	0.040161	0.010945	GSK3B
GO:0072216	positive regulation of metanephros development	1/32	14/18670	0.023738	0.040161	0.010945	MYC
GO:0072498	embryonic skeletal joint development	1/32	14/18670	0.023738	0.040161	0.010945	CTNNB1
GO:0072567	chemokine (C-X-C motif) ligand 2 production	1/32	14/18670	0.023738	0.040161	0.010945	TNF
GO:0090151	establishment of protein localization to mitochondrial membrane	1/32	14/18670	0.023738	0.040161	0.010945	HSP90AA1
GO:0097202	activation of cysteine-type endopeptidase activity	1/32	14/18670	0.023738	0.040161	0.010945	CASP8
GO:0106057	negative regulation of calcineurin-mediated signaling	1/32	14/18670	0.023738	0.040161	0.010945	GSK3B
GO:1900121	negative regulation of receptor binding	1/32	14/18670	0.023738	0.040161	0.010945	IL10

GO:1900452	regulation of long-term synaptic depression	1/32	14/18670	0.023738	0.040161	0.010945	MAPT
GO:1901722	regulation of cell proliferation involved in kidney development	1/32	14/18670	0.023738	0.040161	0.010945	MYC
GO:1903054	negative regulation of extracellular matrix organization	1/32	14/18670	0.023738	0.040161	0.010945	TGFB1
GO:2001212	regulation of vasculogenesis	1/32	14/18670	0.023738	0.040161	0.010945	KDR
GO:0046330	positive regulation of JNK cascade	2/32	140/18670	0.023908	0.040426	0.011017	IL1B/TNF
GO:0006869	lipid transport	3/32	365/18670	0.024178	0.040862	0.011136	AKT1/CAV1/IL1B
GO:0032259	methylation	3/32	366/18670	0.02435	0.041131	0.011209	PARP1/CTNNB1/MYC
GO:0106106	cold-induced thermogenesis	2/32	143/18670	0.024868	0.041962	0.011435	CAV1/IL13
GO:0120161	regulation of cold-induced thermogenesis	2/32	143/18670	0.024868	0.041962	0.011435	CAV1/IL13
GO:0045666	positive regulation of neuron differentiation	3/32	371/18670	0.025219	0.042193	0.011498	BCL2/IL2/MAPT
GO:0001711	endodermal cell fate commitment	1/32	15/18670	0.025413	0.042193	0.011498	CTNNB1
GO:0001921	positive regulation of receptor recycling	1/32	15/18670	0.025413	0.042193	0.011498	SNCA
GO:0010455	positive regulation of cell fate commitment	1/32	15/18670	0.025413	0.042193	0.011498	FGF2

GO:0010561	negative regulation of glycoprotein biosynthetic process	1/32	15/18670	0.025413	0.042193	0.011498	JAK3
GO:0014856	skeletal muscle cell proliferation	1/32	15/18670	0.025413	0.042193	0.011498	FGF2
GO:0015671	oxygen transport	1/32	15/18670	0.025413	0.042193	0.011498	MYC
GO:0032645	regulation of granulocyte macrophage colony-stimulating factor production	1/32	15/18670	0.025413	0.042193	0.011498	IL1B
GO:0033008	positive regulation of mast cell activation involved in immune response	1/32	15/18670	0.025413	0.042193	0.011498	IL13
GO:0033689	negative regulation of osteoblast proliferation	1/32	15/18670	0.025413	0.042193	0.011498	BCL2
GO:0034616	response to laminar fluid shear stress	1/32	15/18670	0.025413	0.042193	0.011498	TGFB1
GO:0035067	negative regulation of histone acetylation	1/32	15/18670	0.025413	0.042193	0.011498	SNCA
GO:0042762	regulation of sulfur metabolic process	1/32	15/18670	0.025413	0.042193	0.011498	SNCA
GO:0043306	positive regulation of mast cell degranulation	1/32	15/18670	0.025413	0.042193	0.011498	IL13
GO:0044406	adhesion of symbiont to host	1/32	15/18670	0.025413	0.042193	0.011498	ICAM1
GO:0045342	MHC class II biosynthetic process	1/32	15/18670	0.025413	0.042193	0.011498	IL10
GO:0045837	negative regulation of membrane potential	1/32	15/18670	0.025413	0.042193	0.011498	MAPT

GO:0045838	positive regulation of membrane potential	1/32	15/18670	0.025413	0.042193	0.011498	AKT1
GO:0048070	regulation of developmental pigmentation	1/32	15/18670	0.025413	0.042193	0.011498	BCL2
GO:0051280	negative regulation of release of sequestered calcium ion into cytosol	1/32	15/18670	0.025413	0.042193	0.011498	TGFB1
GO:0051770	positive regulation of nitric-oxide synthase biosynthetic process	1/32	15/18670	0.025413	0.042193	0.011498	KDR
GO:0072075	metanephric mesenchyme development	1/32	15/18670	0.025413	0.042193	0.011498	MYC
GO:0072160	nephron tubule epithelial cell differentiation	1/32	15/18670	0.025413	0.042193	0.011498	CTNNB1
GO:0072283	metanephric renal vesicle morphogenesis	1/32	15/18670	0.025413	0.042193	0.011498	CTNNB1
GO:0090141	positive regulation of mitochondrial fission	1/32	15/18670	0.025413	0.042193	0.011498	KDR
GO:0090197	positive regulation of chemokine secretion	1/32	15/18670	0.025413	0.042193	0.011498	TNF
GO:0090494	dopamine uptake	1/32	15/18670	0.025413	0.042193	0.011498	SNCA
GO:1903358	regulation of Golgi organization	1/32	15/18670	0.025413	0.042193	0.011498	MAPK1
GO:2000095	regulation of Wnt signaling pathway, planar cell polarity pathway	1/32	15/18670	0.025413	0.042193	0.011498	ABL1
GO:2000402	negative regulation of lymphocyte migration	1/32	15/18670	0.025413	0.042193	0.011498	AKT1

GO:2001185	regulation of CD8-positive, alpha-beta T cell activation	1/32	15/18670	0.025413	0.042193	0.011498	IRF1
GO:0007612	learning	2/32	145/18670	0.025517	0.042344	0.011539	JUN/PTGS2
GO:0035264	multicellular organism growth	2/32	146/18670	0.025844	0.042864	0.011681	BCL2/IGF1
GO:0019359	nicotinamide nucleotide biosynthetic process	2/32	147/18670	0.026173	0.04332	0.011805	IGF1/PTGS2
GO:0019363	pyridine nucleotide biosynthetic process	2/32	147/18670	0.026173	0.04332	0.011805	IGF1/PTGS2
GO:0061351	neural precursor cell proliferation	2/32	147/18670	0.026173	0.04332	0.011805	CTNNB1/TGFB1
GO:0090263	positive regulation of canonical Wnt signaling pathway	2/32	147/18670	0.026173	0.04332	0.011805	CAV1/SRC
GO:0061041	regulation of wound healing	2/32	148/18670	0.026503	0.043844	0.011948	CAV1/FGF2
GO:0050773	regulation of dendrite development	2/32	149/18670	0.026836	0.043944	0.011975	GSK3B/IL2
GO:1903900	regulation of viral life cycle	2/32	149/18670	0.026836	0.043944	0.011975	BCL2/TNF
GO:0030900	forebrain development	3/32	381/18670	0.027008	0.043944	0.011975	CTNNB1/GSK3B/SRC
GO:0006206	pyrimidine nucleobase metabolic process	1/32	16/18670	0.027085	0.043944	0.011975	MAPK1
GO:0006837	serotonin transport	1/32	16/18670	0.027085	0.043944	0.011975	SNCA

GO:0010919	regulation of inositol phosphate biosynthetic process	1/32	16/18670	0.027085	0.043944	0.011975	SNCA
GO:0010934	macrophage cytokine production	1/32	16/18670	0.027085	0.043944	0.011975	TGFB1
GO:0014048	regulation of glutamate secretion	1/32	16/18670	0.027085	0.043944	0.011975	SNCA
GO:0016540	protein autoprocessing	1/32	16/18670	0.027085	0.043944	0.011975	PARP1
GO:0019372	lipoxygenase pathway	1/32	16/18670	0.027085	0.043944	0.011975	PTGS2
GO:0030889	negative regulation of B cell proliferation	1/32	16/18670	0.027085	0.043944	0.011975	IL10
GO:0032305	positive regulation of icosanoid secretion	1/32	16/18670	0.027085	0.043944	0.011975	IL1B
GO:0032604	granulocyte macrophage colony-stimulating factor production	1/32	16/18670	0.027085	0.043944	0.011975	IL1B
GO:0033033	negative regulation of myeloid cell apoptotic process	1/32	16/18670	0.027085	0.043944	0.011975	BCL2
GO:0043217	myelin maintenance	1/32	16/18670	0.027085	0.043944	0.011975	AKT1
GO:0045986	negative regulation of smooth muscle contraction	1/32	16/18670	0.027085	0.043944	0.011975	PTGS2
GO:0046325	negative regulation of glucose import	1/32	16/18670	0.027085	0.043944	0.011975	TNF
GO:0048820	hair follicle maturation	1/32	16/18670	0.027085	0.043944	0.011975	CTNNB1

GO:0051444	negative regulation of ubiquitin-protein transferase activity	1/32	16/18670	0.027085	0.043944	0.011975	ABL1
GO:0051571	positive regulation of histone H3-K4 methylation	1/32	16/18670	0.027085	0.043944	0.011975	CTNNB1
GO:0060572	morphogenesis of an epithelial bud	1/32	16/18670	0.027085	0.043944	0.011975	CTNNB1
GO:0060965	negative regulation of gene silencing by miRNA	1/32	16/18670	0.027085	0.043944	0.011975	TGFB1
GO:0061548	ganglion development	1/32	16/18670	0.027085	0.043944	0.011975	CTNNB1
GO:0071380	cellular response to prostaglandin E stimulus	1/32	16/18670	0.027085	0.043944	0.011975	AKT1
GO:0071871	response to epinephrine	1/32	16/18670	0.027085	0.043944	0.011975	SNCA
GO:0090153	regulation of sphingolipid biosynthetic process	1/32	16/18670	0.027085	0.043944	0.011975	TNF
GO:0090335	regulation of brown fat cell differentiation	1/32	16/18670	0.027085	0.043944	0.011975	PTGS2
GO:0090493	catecholamine uptake	1/32	16/18670	0.027085	0.043944	0.011975	SNCA
GO:0097091	synaptic vesicle clustering	1/32	16/18670	0.027085	0.043944	0.011975	CTNNB1
GO:1902001	fatty acid transmembrane transport	1/32	16/18670	0.027085	0.043944	0.011975	AKT1
GO:1902004	positive regulation of amyloid-beta formation	1/32	16/18670	0.027085	0.043944	0.011975	TNF

GO:1902166	negative regulation of intrinsic apoptotic signaling pathway in response to DNA damage by p53 class mediator	1/32	16/18670	0.027085	0.043944	0.011975	BCL2
GO:1902992	negative regulation of amyloid precursor protein catabolic process	1/32	16/18670	0.027085	0.043944	0.011975	IGF1
GO:1904294	positive regulation of ERAD pathway	1/32	16/18670	0.027085	0.043944	0.011975	CAV1
GO:1905038	regulation of membrane lipid metabolic process	1/32	16/18670	0.027085	0.043944	0.011975	TNF
GO:1990000	amyloid fibril formation	1/32	16/18670	0.027085	0.043944	0.011975	MAPT
GO:2000303	regulation of ceramide biosynthetic process	1/32	16/18670	0.027085	0.043944	0.011975	TNF
GO:2001170	negative regulation of ATP biosynthetic process	1/32	16/18670	0.027085	0.043944	0.011975	PARP1
GO:0008360	regulation of cell shape	2/32	150/18670	0.02717	0.044015	0.011995	ICAM1/KDR
GO:0035567	non-canonical Wnt signaling pathway	2/32	150/18670	0.02717	0.044015	0.011995	ABL1/CTNNB1
GO:0072525	pyridine-containing compound biosynthetic process	2/32	150/18670	0.02717	0.044015	0.011995	IGF1/PTGS2
GO:0031346	positive regulation of cell projection organization	3/32	383/18670	0.027374	0.044323	0.012079	IL2/MAPT/SRC
GO:0030168	platelet activation	2/32	153/18670	0.028182	0.045585	0.012423	MAPK1/SRC
GO:0051017	actin filament bundle assembly	2/32	153/18670	0.028182	0.045585	0.012423	ABL1/SRC

GO:0010770	positive regulation of cell morphogenesis involved in differentiation	2/32	154/18670	0.028522	0.045863	0.012498	ABL1/MAPT
GO:0017156	calcium ion regulated exocytosis	2/32	154/18670	0.028522	0.045863	0.012498	GSK3B/SNCA
GO:1990845	adaptive thermogenesis	2/32	154/18670	0.028522	0.045863	0.012498	CAV1/IL13
GO:0002693	positive regulation of cellular extravasation	1/32	17/18670	0.028754	0.045863	0.012498	ICAM1
GO:0010224	response to UV-B	1/32	17/18670	0.028754	0.045863	0.012498	BCL2
GO:0010715	regulation of extracellular matrix disassembly	1/32	17/18670	0.028754	0.045863	0.012498	TGFB1
GO:0015732	prostaglandin transport	1/32	17/18670	0.028754	0.045863	0.012498	IL1B
GO:0030011	maintenance of cell polarity	1/32	17/18670	0.028754	0.045863	0.012498	GSK3B
GO:0032460	negative regulation of protein oligomerization	1/32	17/18670	0.028754	0.045863	0.012498	SRC
GO:0033145	positive regulation of intracellular steroid hormone receptor signaling pathway	1/32	17/18670	0.028754	0.045863	0.012498	PARP1
GO:0033599	regulation of mammary gland epithelial cell proliferation	1/32	17/18670	0.028754	0.045863	0.012498	CCND1
GO:0035729	cellular response to hepatocyte growth factor stimulus	1/32	17/18670	0.028754	0.045863	0.012498	IL10
GO:0042268	regulation of cytolysis	1/32	17/18670	0.028754	0.045863	0.012498	TGFB1

GO:0042994	cytoplasmic sequestering of transcription factor	1/32	17/18670	0.028754	0.045863	0.012498	IL10
GO:0043923	positive regulation by host of viral transcription	1/32	17/18670	0.028754	0.045863	0.012498	JUN
GO:0048311	mitochondrion distribution	1/32	17/18670	0.028754	0.045863	0.012498	MAPT
GO:0048535	lymph node development	1/32	17/18670	0.028754	0.045863	0.012498	TGFB1
GO:0051284	positive regulation of sequestering of calcium ion	1/32	17/18670	0.028754	0.045863	0.012498	TGFB1
GO:0060644	mammary gland epithelial cell differentiation	1/32	17/18670	0.028754	0.045863	0.012498	AKT1
GO:0061323	cell proliferation involved in heart morphogenesis	1/32	17/18670	0.028754	0.045863	0.012498	CTNNB1
GO:0070242	thymocyte apoptotic process	1/32	17/18670	0.028754	0.045863	0.012498	JAK3
GO:0071360	cellular response to exogenous dsRNA	1/32	17/18670	0.028754	0.045863	0.012498	CAV1
GO:0072673	lamellipodium morphogenesis	1/32	17/18670	0.028754	0.045863	0.012498	SRC
GO:0090185	negative regulation of kidney development	1/32	17/18670	0.028754	0.045863	0.012498	CTNNB1
GO:1904355	positive regulation of telomere capping	1/32	17/18670	0.028754	0.045863	0.012498	MAPK1
GO:1904948	midbrain dopaminergic neuron differentiation	1/32	17/18670	0.028754	0.045863	0.012498	CTNNB1

GO:2000136	regulation of cell proliferation involved in heart morphogenesis	1/32	17/18670	0.028754	0.045863	0.012498	CTNNB1
GO:2001267	regulation of cysteine-type endopeptidase activity involved in apoptotic signaling pathway	1/32	17/18670	0.028754	0.045863	0.012498	CASP8
GO:0051053	negative regulation of DNA metabolic process	2/32	156/18670	0.029209	0.046565	0.01269	PARP1/SRC
GO:0019827	stem cell population maintenance	2/32	157/18670	0.029554	0.047069	0.012827	CTNNB1/FGF2
GO:0061572	actin filament bundle organization	2/32	157/18670	0.029554	0.047069	0.012827	ABL1/SRC
GO:0016482	cytosolic transport	2/32	158/18670	0.029902	0.047597	0.012971	MAPK1/SRC
GO:0007252	I-kappaB phosphorylation	1/32	18/18670	0.03042	0.047597	0.012971	AKT1
GO:0030809	negative regulation of nucleotide biosynthetic process	1/32	18/18670	0.03042	0.047597	0.012971	PARP1
GO:0031065	positive regulation of histone deacetylation	1/32	18/18670	0.03042	0.047597	0.012971	TGFB1
GO:0031293	membrane protein intracellular domain proteolysis	1/32	18/18670	0.03042	0.047597	0.012971	TGFB1
GO:0031998	regulation of fatty acid beta-oxidation	1/32	18/18670	0.03042	0.047597	0.012971	AKT1
GO:0032693	negative regulation of interleukin-10 production	1/32	18/18670	0.03042	0.047597	0.012971	JAK3
GO:0035994	response to muscle stretch	1/32	18/18670	0.03042	0.047597	0.012971	JUN

GO:0036035	osteoclast development	1/32	18/18670	0.03042	0.047597	0.012971	SRC
GO:0043011	myeloid dendritic cell differentiation	1/32	18/18670	0.03042	0.047597	0.012971	TGFB1
GO:0045056	transcytosis	1/32	18/18670	0.03042	0.047597	0.012971	SRC
GO:0051782	negative regulation of cell division	1/32	18/18670	0.03042	0.047597	0.012971	MYC
GO:0051895	negative regulation of focal adhesion assembly	1/32	18/18670	0.03042	0.047597	0.012971	SRC
GO:0060192	negative regulation of lipase activity	1/32	18/18670	0.03042	0.047597	0.012971	ABL1
GO:0060546	negative regulation of necroptotic process	1/32	18/18670	0.03042	0.047597	0.012971	CAV1
GO:0060749	mammary gland alveolus development	1/32	18/18670	0.03042	0.047597	0.012971	CCND1
GO:0061377	mammary gland lobule development	1/32	18/18670	0.03042	0.047597	0.012971	CCND1
GO:0070230	positive regulation of lymphocyte apoptotic process	1/32	18/18670	0.03042	0.047597	0.012971	IL10
GO:0070233	negative regulation of T cell apoptotic process	1/32	18/18670	0.03042	0.047597	0.012971	JAK3
GO:0071318	cellular response to ATP	1/32	18/18670	0.03042	0.047597	0.012971	PTGS2
GO:0071605	monocyte chemotactic protein-1 production	1/32	18/18670	0.03042	0.047597	0.012971	IL1B

GO:0071637	regulation of monocyte chemotactic protein-1 production	1/32	18/18670	0.03042	0.047597	0.012971	IL1B
GO:0072077	renal vesicle morphogenesis	1/32	18/18670	0.03042	0.047597	0.012971	CTNNB1
GO:0072079	nephron tubule formation	1/32	18/18670	0.03042	0.047597	0.012971	CTNNB1
GO:0090190	positive regulation of branching involved in ureteric bud morphogenesis	1/32	18/18670	0.03042	0.047597	0.012971	TGFB1
GO:0090196	regulation of chemokine secretion	1/32	18/18670	0.03042	0.047597	0.012971	TNF
GO:0097094	craniofacial suture morphogenesis	1/32	18/18670	0.03042	0.047597	0.012971	TGFB1
GO:0150079	negative regulation of neuroinflammatory response	1/32	18/18670	0.03042	0.047597	0.012971	IGF1
GO:1900221	regulation of amyloid-beta clearance	1/32	18/18670	0.03042	0.047597	0.012971	TNF
GO:1900372	negative regulation of purine nucleotide biosynthetic process	1/32	18/18670	0.03042	0.047597	0.012971	PARP1
GO:1902165	regulation of intrinsic apoptotic signaling pathway in response to DNA damage by p53 class mediator	1/32	18/18670	0.03042	0.047597	0.012971	BCL2
GO:1903019	negative regulation of glycoprotein metabolic process	1/32	18/18670	0.03042	0.047597	0.012971	JAK3
GO:2000193	positive regulation of fatty acid transport	1/32	18/18670	0.03042	0.047597	0.012971	IL1B
GO:2000319	regulation of T-helper 17 cell differentiation	1/32	18/18670	0.03042	0.047597	0.012971	IL2

GO:2000647	negative regulation of stem cell proliferation	1/32	18/18670	0.03042	0.047597	0.012971	TGFB1
GO:2000757	negative regulation of peptidyl-lysine acetylation	1/32	18/18670	0.03042	0.047597	0.012971	SNCA
GO:0021915	neural tube development	2/32	160/18670	0.030601	0.047811	0.013029	ABL1/TGFB1
GO:0032680	regulation of tumor necrosis factor production	2/32	160/18670	0.030601	0.047811	0.013029	IGF1/IL10
GO:1903305	regulation of regulated secretory pathway	2/32	160/18670	0.030601	0.047811	0.013029	GSK3B/IL13
GO:0006732	coenzyme metabolic process	3/32	403/18670	0.031179	0.04869	0.013269	IGF1/PTGS2/SNCA
GO:0015718	monocarboxylic acid transport	2/32	162/18670	0.031307	0.048866	0.013317	AKT1/IL1B
GO:0032640	tumor necrosis factor production	2/32	163/18670	0.031663	0.049215	0.013412	IGF1/IL10
GO:1903555	regulation of tumor necrosis factor superfamily cytokine production	2/32	163/18670	0.031663	0.049215	0.013412	IGF1/IL10
GO:0006633	fatty acid biosynthetic process	2/32	164/18670	0.03202	0.049215	0.013412	IL1B/PTGS2
GO:0001832	blastocyst growth	1/32	19/18670	0.032083	0.049215	0.013412	IGF1
GO:0002363	alpha-beta T cell lineage commitment	1/32	19/18670	0.032083	0.049215	0.013412	BCL2
GO:0002726	positive regulation of T cell cytokine production	1/32	19/18670	0.032083	0.049215	0.013412	IL1B

GO:0003159	morphogenesis of an endothelium	1/32	19/18670	0.032083	0.049215	0.013412	CTNNB1
GO:0007096	regulation of exit from mitosis	1/32	19/18670	0.032083	0.049215	0.013412	TGFB1
GO:0010888	negative regulation of lipid storage	1/32	19/18670	0.032083	0.049215	0.013412	TNF
GO:0015669	gas transport	1/32	19/18670	0.032083	0.049215	0.013412	MYC
GO:0016082	synaptic vesicle priming	1/32	19/18670	0.032083	0.049215	0.013412	SNCA
GO:0030949	positive regulation of vascular endothelial growth factor receptor signaling pathway	1/32	19/18670	0.032083	0.049215	0.013412	IL1B
GO:0032303	regulation of icosanoid secretion	1/32	19/18670	0.032083	0.049215	0.013412	IL1B
GO:0032700	negative regulation of interleukin-17 production	1/32	19/18670	0.032083	0.049215	0.013412	TGFB1
GO:0034063	stress granule assembly	1/32	19/18670	0.032083	0.049215	0.013412	MAPT
GO:0035493	SNARE complex assembly	1/32	19/18670	0.032083	0.049215	0.013412	SNCA
GO:0035728	response to hepatocyte growth factor	1/32	19/18670	0.032083	0.049215	0.013412	IL10
GO:0045063	T-helper 1 cell differentiation	1/32	19/18670	0.032083	0.049215	0.013412	JAK3
GO:0045414	regulation of interleukin-8 biosynthetic process	1/32	19/18670	0.032083	0.049215	0.013412	TNF

GO:0051131	chaperone-mediated protein complex assembly	1/32	19/18670	0.032083	0.049215	0.013412	HSP90AA1
GO:0060149	negative regulation of posttranscriptional gene silencing	1/32	19/18670	0.032083	0.049215	0.013412	TGFB1
GO:0060231	mesenchymal to epithelial transition	1/32	19/18670	0.032083	0.049215	0.013412	CTNNB1
GO:0060967	negative regulation of gene silencing by RNA	1/32	19/18670	0.032083	0.049215	0.013412	TGFB1
GO:0061154	endothelial tube morphogenesis	1/32	19/18670	0.032083	0.049215	0.013412	CTNNB1
GO:0072074	kidney mesenchyme development	1/32	19/18670	0.032083	0.049215	0.013412	MYC
GO:0072087	renal vesicle development	1/32	19/18670	0.032083	0.049215	0.013412	CTNNB1
GO:0098911	regulation of ventricular cardiac muscle cell action potential	1/32	19/18670	0.032083	0.049215	0.013412	CAV1
GO:1900409	positive regulation of cellular response to oxidative stress	1/32	19/18670	0.032083	0.049215	0.013412	TNF
GO:1902074	response to salt	1/32	19/18670	0.032083	0.049215	0.013412	TGFB1
GO:1902176	negative regulation of oxidative stress-induced intrinsic apoptotic signaling pathway	1/32	19/18670	0.032083	0.049215	0.013412	AKT1
GO:1903798	regulation of production of miRNAs involved in gene silencing by miRNA	1/32	19/18670	0.032083	0.049215	0.013412	TGFB1
GO:1905288	vascular associated smooth muscle cell apoptotic process	1/32	19/18670	0.032083	0.049215	0.013412	IGF1

GO:1905459	regulation of vascular associated smooth muscle cell apoptotic process	1/32	19/18670	0.032083	0.049215	0.013412	IGF1
GO:1905939	regulation of gonad development	1/32	19/18670	0.032083	0.049215	0.013412	SRC
GO:2000251	positive regulation of actin cytoskeleton reorganization	1/32	19/18670	0.032083	0.049215	0.013412	ABL1
GO:2000696	regulation of epithelial cell differentiation involved in kidney development	1/32	19/18670	0.032083	0.049215	0.013412	CTNNB1
GO:0007093	mitotic cell cycle checkpoint	2/32	165/18670	0.032379	0.049644	0.013529	CCND1/TGFB1

Supplementary Table S5-2: Datailed information of Cellular components(CC) enrichment of PPI network cluster 1 targets

ID	Description	GeneRatio	BgRatio	pvalue	p.adjust	qvalue	geneID	Count
GO:0045121	membrane raft	11/32	315/19717	1.39E-12	1.24E-10	6.97E-11	FASLG/CASP8/CAV1/CTNNB1/ICAM1/KDR/MAPT/MAPK1/PTGS2/SRC/TNF	11
GO:0098857	membrane microdomain	11/32	316/19717	1.44E-12	1.24E-10	6.97E-11	FASLG/CASP8/CAV1/CTNNB1/ICAM1/KDR/MAPT/MAPK1/PTGS2/SRC/TNF	11
GO:0098589	membrane region	11/32	328/19717	2.16E-12	1.24E-10	6.97E-11	FASLG/CASP8/CAV1/CTNNB1/ICAM1/KDR/MAPT/MAPK1/PTGS2/SRC/TNF	11
GO:0005901	caveola	6/32	80/19717	3.07E-09	1.32E-07	7.44E-08	FASLG/CAV1/CTNNB1/MAPK1/PTGS2/SRC	6
GO:0044853	plasma membrane raft	6/32	109/19717	2.00E-08	6.89E-07	3.88E-07	FASLG/CAV1/CTNNB1/MAPK1/PTGS2/SRC	6
GO:0043209	myelin sheath	3/32	49/19717	6.80E-05	0.001949	0.001098	BCL2/ERBB2/HSP90AA1	3
GO:0043025	neuronal cell body	6/32	497/19717	1.29E-04	0.003136	0.001766	ABL1/HSP90AA1/MAPT/MAPK1/SNCA/TGFB1	6
GO:0030877	beta-catenin destruction complex	2/32	12/19717	1.67E-04	0.003136	0.001766	CTNNB1/GSK3B	2
GO:1990909	Wnt signalosome	2/32	12/19717	1.67E-04	0.003136	0.001766	CTNNB1/GSK3B	2
GO:0060205	cytoplasmic vesicle lumen	5/32	338/19717	1.98E-04	0.003136	0.001766	FASLG/HSP90AA1/IGF1/MAPK1/TGFB1	5
GO:0031983	vesicle lumen	5/32	339/19717	2.01E-04	0.003136	0.001766	FASLG/HSP90AA1/IGF1/MAPK1/TGFB1	5
GO:0031091	platelet alpha granule	3/32	91/19717	4.28E-04	0.006136	0.003455	IGF1/SNCA/TGFB1	3

GO:0099091	postsynaptic specialization, intracellular component	2/32	21/19717	0.000526	0.006955	0.003916	CTNNB1/SRC	2
GO:0005719	nuclear euchromatin	2/32	30/19717	0.001079	0.013256	0.007464	CTNNB1/JUN	2
GO:0000791	euchromatin	2/32	38/19717	0.00173	0.0186	0.010473	CTNNB1/JUN	2
GO:0034774	secretory granule lumen	4/32	321/19717	0.00173	0.0186	0.010473	HSP90AA1/IGF1/MAPK1/TGFB1	4
GO:0030426	growth cone	3/32	171/19717	0.002643	0.025375	0.014287	HSP90AA1/MAPT/SNCA	3
GO:0005775	vacuolar lumen	3/32	172/19717	0.002687	0.025375	0.014287	FASLG/HSP90AA1/MAPK1	3
GO:0030427	site of polarized growth	3/32	176/19717	0.002867	0.025375	0.014287	HSP90AA1/MAPT/SNCA	3
GO:0005741	mitochondrial outer membrane	3/32	178/19717	0.00296	0.025375	0.014287	BCL2/CASP8/SNCA	3
GO:0000790	nuclear chromatin	4/32	377/19717	0.003098	0.025375	0.014287	CTNNB1/IRF1/JUN/MYC	4
GO:0009897	external side of plasma membrane	4/32	393/19717	0.003595	0.026296	0.014806	FASLG/ICAM1/IL13/TNF	4
GO:0031252	cell leading edge	4/32	403/19717	0.003932	0.026296	0.014806	ABL1/CTNNB1/MAPT/SRC	4
GO:0005925	focal adhesion	4/32	405/19717	0.004002	0.026296	0.014806	CAV1/CTNNB1/ICAM1/MAPK1	4
GO:0005924	cell-substrate adherens junction	4/32	408/19717	0.004108	0.026296	0.014806	CAV1/CTNNB1/ICAM1/MAPK1	4
GO:0031968	organelle outer membrane	3/32	201/19717	0.004164	0.026296	0.014806	BCL2/CASP8/SNCA	3
GO:0030055	cell-substrate junction	4/32	412/19717	0.004253	0.026296	0.014806	CAV1/CTNNB1/ICAM1/MAPK1	4
GO:0019867	outer membrane	3/32	203/19717	0.004281	0.026296	0.014806	BCL2/CASP8/SNCA	3
GO:0031093	platelet alpha granule lumen	2/32	67/19717	0.005283	0.031333	0.017642	IGF1/TGFB1	2
GO:0005635	nuclear envelope	4/32	464/19717	0.006461	0.037044	0.020857	ABL1/PARP1/BCL2/SNCA	4
GO:0016234	inclusion body	2/32	82/19717	0.007816	0.041421	0.023321	MAPT/SNCA	2
GO:0005902	microvillus	2/32	83/19717	0.008001	0.041421	0.023321	CTNNB1/TGFB1	2
GO:0017053	transcriptional repressor complex	2/32	84/19717	0.008188	0.041421	0.023321	CCND1/JUN	2
GO:0120111	neuron projection cytoplasm	2/32	84/19717	0.008188	0.041421	0.023321	MAPT/MAPK1	2

Supplementary Table S5-3: Datailed information of molecular functions(MF) enrichment of PPI network cluster 1 targets

ID	Description	GeneRatio	BgRatio	pvalue	p.adjust	qvalue	geneID	Count
GO:0005125	cytokine activity	9/32	220/17697	1.32E-10	2.71E-08	1.32E-08	FASLG/FGF2/IL1A/IL1B/IL2/IL10/IL13/TGFB1/TNF	9
GO:0048018	receptor ligand activity	11/32	482/17697	4.20E-10	4.33E-08	2.10E-08	FASLG/FGF2/IGF1/IL1A/IL1B/IL2/IL10/IL13/MAPT/TGFB1/TNF	11
GO:0005126	cytokine receptor binding	9/32	286/17697	1.34E-09	9.22E-08	4.48E-08	FASLG/CASP8/IL1A/IL1B/IL2/IL10/IL13/TGFB1/TNF	9
GO:0019902	phosphatase binding	7/32	185/17697	3.29E-08	1.69E-06	8.22E-07	AKT1/BCL2/CTNNB1/ERBB2/JAK3/MAPT/MAPK1	7
GO:0005178	integrin binding	6/32	132/17697	1.19E-07	4.46E-06	2.16E-06	FGF2/ICAM1/IGF1/IL1B/KDR/SRC	6
GO:0070851	growth factor receptor binding	6/32	134/17697	1.30E-07	4.46E-06	2.16E-06	FGF2/IL1A/IL1B/IL2/IL10/SRC	6
GO:0019903	protein phosphatase binding	6/32	140/17697	1.68E-07	4.96E-06	2.41E-06	AKT1/BCL2/CTNNB1/ERBB2/JAK3/MAPT	6
GO:0004713	protein tyrosine kinase activity	5/32	134/17697	3.95E-06	0.000102	4.93E-05	ABL1/ERBB2/JAK3/KDR/SRC	5
GO:0008083	growth factor activity	5/32	163/17697	1.03E-05	0.000235	0.000114	FGF2/IGF1/IL2/IL10/TGFB1	5
GO:0031625	ubiquitin protein ligase binding	6/32	290/17697	1.16E-05	0.00024	0.000116	BCL2/CASP8/GSK3B/HSP90AA1/JUN/SRC	6
GO:0051219	phosphoprotein binding	4/32	83/17697	1.46E-05	0.000274	0.000133	ABL1/MAPK1/SNCA/SRC	4
GO:0044389	ubiquitin-like protein ligase binding	6/32	308/17697	1.64E-05	0.000281	0.000137	BCL2/CASP8/GSK3B/HSP90AA1/JUN/SRC	6
GO:0005164	tumor necrosis factor receptor binding	3/32	31/17697	2.33E-05	0.000353	0.000171	FASLG/CASP8/TNF	3
GO:0050839	cell adhesion molecule binding	7/32	499/17697	2.48E-05	0.000353	0.000171	CTNNB1/FGF2/ICAM1/IGF1/IL1B/KDR/SRC	7
GO:0051721	protein phosphatase 2A binding	3/32	32/17697	2.57E-05	0.000353	0.000171	AKT1/BCL2/MAPT	3
GO:0030331	estrogen receptor binding	3/32	42/17697	5.88E-05	0.000757	0.000367	PARP1/CTNNB1/SRC	3
GO:0048156	tau protein binding	3/32	45/17697	7.24E-05	0.000838	0.000407	GSK3B/HSP90AA1/SNCA	3
GO:0004715	non-membrane spanning protein tyrosine kinase activity	3/32	46/17697	7.73E-05	0.000838	0.000407	ABL1/JAK3/SRC	3
GO:0032813	tumor necrosis factor receptor superfamily binding	3/32	46/17697	7.73E-05	0.000838	0.000407	FASLG/CASP8/TNF	3
GO:0005080	protein kinase C binding	3/32	53/17697	0.000118	0.001219	0.000592	ABL1/AKT1/SRC	3
GO:0097110	scaffold protein binding	3/32	59/17697	0.000163	0.001599	0.000776	CASP8/HSP90AA1/SRC	3
GO:0034452	dynactin binding	2/32	12/17697	0.000207	0.001936	0.00094	GSK3B/MAPT	2
GO:0008022	protein C-terminus binding	4/32	187/17697	0.000344	0.003085	0.001497	ABL1/CTNNB1/ERBB2/SRC	4
GO:0005149	interleukin-1 receptor binding	2/32	16/17697	0.000374	0.003211	0.001559	IL1A/IL1B	2

GO:0046332	SMAD binding	3/32	80/17697	0.000401	0.003308	0.001606	PARP1/CTNNB1/JUN	3
GO:0005123	death receptor binding	2/32	17/17697	0.000424	0.003356	0.001629	FASLG/CASP8	2
GO:0070064	proline-rich region binding	2/32	18/17697	0.000476	0.003529	0.001713	ABL1/CCND1	2
GO:0033613	activating transcription factor binding	3/32	85/17697	0.00048	0.003529	0.001713	CTNNB1/JUN/MYC	3
GO:0035258	steroid hormone receptor binding	3/32	92/17697	0.000605	0.004295	0.002085	PARP1/CTNNB1/SRC	3
GO:0005158	insulin receptor binding	2/32	22/17697	0.000715	0.004912	0.002385	IGF1/SRC	2
GO:0070412	R-SMAD binding	2/32	23/17697	0.000783	0.005201	0.002525	PARP1/JUN	2
GO:0047485	protein N-terminus binding	3/32	109/17697	0.00099	0.006372	0.003093	PARP1/SNCA/TGFB1	3
GO:0042826	histone deacetylase binding	3/32	111/17697	0.001043	0.006513	0.003162	PARP1/CCND1/HSP90AA1	3
GO:0046875	ephrin receptor binding	2/32	27/17697	0.001081	0.006549	0.003179	ABL1/SRC	2
GO:0031072	heat shock protein binding	3/32	119/17697	0.001275	0.007507	0.003644	KDR/MAPT/SNCA	3
GO:0044325	ion channel binding	3/32	124/17697	0.001436	0.008215	0.003988	CAV1/CTNNB1/SRC	3
GO:0002020	protease binding	3/32	128/17697	0.001573	0.008755	0.00425	BCL2/GSK3B/TNF	3
GO:0097718	disordered domain specific binding	2/32	33/17697	0.001615	0.008755	0.00425	CTNNB1/HSP90AA1	2
GO:0042169	SH2 domain binding	2/32	36/17697	0.00192	0.010144	0.004924	ABL1/SRC	2
GO:0016248	channel inhibitor activity	2/32	38/17697	0.002138	0.011012	0.005345	BCL2/CAV1	2
GO:0001784	phosphotyrosine residue binding	2/32	40/17697	0.002367	0.011893	0.005773	ABL1/MAPK1	2
GO:0051879	Hsp90 protein binding	2/32	41/17697	0.002486	0.011985	0.005818	KDR/MAPT	2
GO:0003713	transcription coactivator activity	4/32	319/17697	0.002502	0.011985	0.005818	ABL1/CTNNB1/FGF2/JUN	4
GO:0035257	nuclear hormone receptor binding	3/32	152/17697	0.002567	0.012018	0.005834	PARP1/CTNNB1/SRC	3
GO:0001085	RNA polymerase II transcription factor binding	3/32	155/17697	0.002713	0.01223	0.005937	CTNNB1/GSK3B/JUN	3
GO:0004712	protein serine/threonine/tyrosine kinase activity	2/32	43/17697	0.002731	0.01223	0.005937	AKT1/MAPK1	2
GO:0030674	protein binding, bridging	3/32	170/17697	0.00352	0.015429	0.00749	CAV1/MAPT/SRC	3
GO:0045309	protein phosphorylated amino acid binding	2/32	51/17697	0.003822	0.016401	0.007962	ABL1/MAPK1	2
GO:0001102	RNA polymerase II activating transcription factor binding	2/32	53/17697	0.004121	0.017325	0.00841	CTNNB1/JUN	2
GO:0051427	hormone receptor binding	3/32	185/17697	0.004461	0.018379	0.008922	PARP1/CTNNB1/SRC	3

GO:0005507	copper ion binding	2/32	59/17697	0.005082	0.020529	0.009966	IL1A/SNCA	2
GO:0004714	transmembrane receptor protein tyrosine kinase activity	2/32	62/17697	0.005598	0.022177	0.010766	ERBB2/KDR	2
GO:0019207	kinase regulator activity	3/32	207/17697	0.006094	0.023687	0.011498	CCND1/IL2/TGFB1	3
GO:0070491	repressing transcription factor binding	2/32	71/17697	0.007283	0.027782	0.013486	CTNNB1/MYC	2
GO:0001228	DNA-binding transcription activator activity, RNA polymerase II-specific	4/32	439/17697	0.007752	0.029035	0.014095	PARP1/IRF1/JUN/MYC	4
GO:0060090	molecular adaptor activity	3/32	237/17697	0.008834	0.032341	0.015699	CAV1/MAPT/SRC	3
GO:0019199	transmembrane receptor protein kinase activity	2/32	79/17697	0.008949	0.032341	0.015699	ERBB2/KDR	2
GO:0019209	kinase activator activity	2/32	86/17697	0.010533	0.037409	0.01816	IL2/TGFB1	2

Supplementary Table S5-4: Datailed information of KEGG pathways enrichment of PPI network cluster 1 targets

ID	Description	GeneRatio	BgRatio	pvalue	p.adjust	qvalue	geneID	Count
hsa05205	Proteoglycans in cancer	14/32	205/8085	9.20E-15	1.75E-12	3.68E-13	AKT1/FASLG/CCND1/CAV1/CTNNB1/ERBB2/FGF2/IGF1/KDR/MYC/MAPK1/SRC/TGFB1/TNF	14
hsa05418	Fluid shear stress and atherosclerosis	12/32	139/8085	6.95E-14	6.07E-12	1.28E-12	AKT1/BCL2/CAV1/CTNNB1/HSP90AA1/ICAM1/IL1A/IL1B/JUN/KDR/SRC/TNF	12
hsa04625	C-type lectin receptor signaling pathway	11/32	104/8085	9.59E-14	6.07E-12	1.28E-12	AKT1/CASP8/IL1B/IL2/IL10/IRF1/JUN/MAPK1/PTGS2/SRC/TNF	11
hsa04010	MAPK signaling pathway	14/32	294/8085	1.36E-12	6.47E-11	1.36E-11	AKT1/FASLG/ERBB2/FGF2/IGF1/IL1A/IL1B/JUN/KDR/MAPT/MYC/MAPK1/TGFB1/TNF	14
hsa05162	Measles	11/32	139/8085	2.47E-12	8.64E-11	1.82E-11	AKT1/FASLG/CCND1/BCL2/CASP8/GSK3B/IL1A/IL1B/IL2/JAK3/JUN	11
hsa04933	AGE-RAGE signaling pathway in diabetic complications	10/32	100/8085	2.73E-12	8.64E-11	1.82E-11	AKT1/CCND1/BCL2/ICAM1/IL1A/IL1B/JUN/MAPK1/TGFB1/TNF	10
hsa05142	Chagas disease	10/32	102/8085	3.34E-12	9.07E-11	1.91E-11	AKT1/FASLG/CASP8/IL1B/IL2/IL10/JUN/MAPK1/TGFB1/TNF	10
hsa05167	Kaposi sarcoma-associated herpesvirus infection	12/32	194/8085	3.85E-12	9.14E-11	1.92E-11	AKT1/CCND1/CASP8/CTNNB1/FGF2/GSK3B/ICAM1/JUN/MYC/MAPK1/PTGS2/SRC	12
hsa04510	Focal adhesion	12/32	201/8085	5.87E-12	1.24E-10	2.61E-11	AKT1/CCND1/BCL2/CAV1/CTNNB1/ERBB2/GSK3B/IGF1/JUN/KDR/MAPK1/SRC	12
hsa01521	EGFR tyrosine kinase inhibitor resistance	9/32	79/8085	1.19E-11	2.11E-10	4.45E-11	AKT1/BCL2/ERBB2/FGF2/GSK3B/IGF1/KDR/MAPK1/SRC	9
hsa05417	Lipid and atherosclerosis	12/32	215/8085	1.30E-11	2.11E-10	4.45E-11	AKT1/FASLG/BCL2/CASP8/GSK3B/HSP90AA1/ICAM1/IL1B/JUN/MAPK1/SRC/TNF	12
hsa05161	Hepatitis B	11/32	162/8085	1.33E-11	2.11E-10	4.45E-11	AKT1/FASLG/BCL2/CASP8/JAK3/JUN/MYC/MAPK1/SRC/TGFB1/TNF	11

hsa04151	PI3K-Akt signaling pathway	14/32	354/8085	1.70E-11	2.48E-10	5.23E-11	AKT1/FASLG/CCND1/BCL2/ERBB2/FGF2/GSK3B/HSP90AA1/IGF1/IL2/JAK3/KDR/MYC/MAPK1	14
hsa05163	Human cytomegalovirus infection	12/32	225/8085	2.23E-11	3.02E-10	6.36E-11	AKT1/FASLG/CCND1/CASP8/CTNNB1/GSK3B/IL1B/MYC/MAPK1/PTGS2/SRC/TNF	12
hsa05210	Colorectal cancer	9/32	86/8085	2.61E-11	3.31E-10	6.97E-11	AKT1/CCND1/BCL2/CTNNB1/GSK3B/JUN/MYC/MAPK1/TGFB1	9
hsa04657	IL-17 signaling pathway	9/32	94/8085	5.92E-11	7.03E-10	1.48E-10	CASP8/GSK3B/HSP90AA1/IL1B/IL13/JUN/MAPK1/PTGS2/TNF	9
hsa05215	Prostate cancer	9/32	97/8085	7.89E-11	8.82E-10	1.86E-10	AKT1/CCND1/BCL2/CTNNB1/ERBB2/GSK3B/HSP90AA1/IGF1/MAPK1	9
hsa05321	Inflammatory bowel disease	8/32	65/8085	1.01E-10	1.07E-09	2.25E-10	IL1A/IL1B/IL2/IL10/IL13/JUN/TGFB1/TNF	8
hsa05224	Breast cancer	10/32	147/8085	1.33E-10	1.33E-09	2.80E-10	AKT1/CCND1/CTNNB1/ERBB2/FGF2/GSK3B/IGF1/JUN/MYC/MAPK1	10
hsa05226	Gastric cancer	10/32	149/8085	1.52E-10	1.45E-09	3.04E-10	AKT1/CCND1/BCL2/CTNNB1/ERBB2/FGF2/GSK3B/MYC/MAPK1/TGFB1	10
hsa04668	TNF signaling pathway	9/32	112/8085	2.92E-10	2.64E-09	5.56E-10	AKT1/CASP8/ICAM1/IL1B/IRF1/JUN/MAPK1/PTGS2/TNF	9
hsa05140	Leishmaniasis	8/32	77/8085	4.08E-10	3.53E-09	7.43E-10	IL1A/IL1B/IL10/JUN/MAPK1/PTGS2/TGFB1/TNF	8
hsa04012	ErbB signaling pathway	8/32	85/8085	9.15E-10	7.55E-09	1.59E-09	ABL1/AKT1/ERBB2/GSK3B/JUN/MYC/MAPK1/SRC	8
hsa05152	Tuberculosis	10/32	180/8085	9.84E-10	7.79E-09	1.64E-09	AKT1/BCL2/CASP8/IL1A/IL1B/IL10/MAPK1/SRC/TGFB1/TNF	10
hsa05135	Yersinia infection	9/32	137/8085	1.78E-09	1.36E-08	2.85E-09	AKT1/GSK3B/IL1B/IL2/IL10/JUN/MAPK1/SRC/TNF	9
hsa05213	Endometrial cancer	7/32	58/8085	1.97E-09	1.44E-08	3.03E-09	AKT1/CCND1/CTNNB1/ERBB2/GSK3B/MYC/MAPK1	7
hsa01522	Endocrine resistance	8/32	98/8085	2.89E-09	2.03E-08	4.28E-09	AKT1/CCND1/BCL2/ERBB2/IGF1/JUN/MAPK1/SRC	8
hsa04932	Non-alcoholic fatty liver disease	9/32	155/8085	5.34E-09	3.62E-08	7.62E-09	AKT1/FASLG/CASP8/GSK3B/IL1A/IL1B/JUN/TGFB1/TNF	9
hsa05160	Hepatitis C	9/32	157/8085	5.98E-09	3.92E-08	8.24E-09	AKT1/FASLG/CCND1/CASP8/CTNNB1/GSK3B/MYC/MAPK1/TNF	9
hsa05166	Human T-cell leukemia virus 1 infection	10/32	219/8085	6.64E-09	4.10E-08	8.62E-09	AKT1/CCND1/ICAM1/IL2/JAK3/JUN/MYC/MAPK1/TGFB1/TNF	10
hsa04217	Necroptosis	9/32	159/8085	6.68E-09	4.10E-08	8.62E-09	PARP1/FASLG/BCL2/CASP8/HSP90AA1/IL1A/IL1B/JAK3/TNF	9
hsa05133	Pertussis	7/32	76/8085	1.37E-08	8.10E-08	1.71E-08	IL1A/IL1B/IL10/IRF1/JUN/MAPK1/TNF	7
hsa05132	Salmonella infection	10/32	249/8085	2.28E-08	1.31E-07	2.76E-08	AKT1/BCL2/CASP8/CTNNB1/HSP90AA1/IL1B/JUN/MYC/MAPK1/TNF	10
hsa04210	Apoptosis	8/32	136/8085	3.90E-08	2.18E-07	4.59E-08	PARP1/AKT1/FASLG/BCL2/CASP8/JUN/MAPK1/TNF	8
hsa05169	Epstein-Barr virus infection	9/32	202/8085	5.41E-08	2.94E-07	6.19E-08	AKT1/CCND1/BCL2/CASP8/ICAM1/JAK3/JUN/MYC/TNF	9
hsa04550	Signaling pathways regulating pluripotency of stem cells	8/32	143/8085	5.78E-08	3.05E-07	6.43E-08	AKT1/CTNNB1/FGF2/GSK3B/IGF1/JAK3/MYC/MAPK1	8
hsa05207	Chemical carcinogenesis - receptor activation	9/32	212/8085	8.22E-08	4.19E-07	8.82E-08	AKT1/CCND1/BCL2/FGF2/HSP90AA1/JUN/MYC/MAPK1/SRC	9
hsa05010	Alzheimer disease	11/32	369/8085	8.38E-08	4.19E-07	8.82E-08	AKT1/CASP8/CTNNB1/GSK3B/IL1A/IL1B/MAPT/MAPK1/PTGS2/SNCA/TNF	11

hsa05022	Pathways of neurodegeneration - multiple diseases	12/32	476/8085	1.16E-07	5.64E-07	1.19E-07	FASLG/BCL2/CASP8/CTNNB1/GSK3B/IL1A/IL1B/MAPT/MAPK1/PTGS2/SNCA/TNF	12
hsa04660	T cell receptor signaling pathway	7/32	104/8085	1.23E-07	5.84E-07	1.23E-07	AKT1/GSK3B/IL2/IL10/JUN/MAPK1/TNF	7
hsa04630	JAK-STAT signaling pathway	8/32	162/8085	1.53E-07	7.08E-07	1.49E-07	AKT1/CCND1/BCL2/IL2/IL10/IL13/JAK3/MYC	8
hsa04659	Th17 cell differentiation	7/32	108/8085	1.60E-07	7.22E-07	1.52E-07	HSP90AA1/IL1B/IL2/JAK3/JUN/MAPK1/TGFB1	7
hsa05145	Toxoplasmosis	7/32	112/8085	2.05E-07	9.06E-07	1.91E-07	AKT1/BCL2/CASP8/IL10/MAPK1/TGFB1/TNF	7
hsa05164	Influenza A	8/32	172/8085	2.43E-07	1.05E-06	2.21E-07	AKT1/FASLG/CASP8/ICAM1/IL1A/IL1B/MAPK1/TNF	8
hsa04917	Prolactin signaling pathway	6/32	70/8085	2.57E-07	1.09E-06	2.28E-07	AKT1/CCND1/GSK3B/IRF1/MAPK1/SRC	6
hsa05143	African trypanosomiasis	5/32	37/8085	2.79E-07	1.15E-06	2.43E-07	FASLG/ICAM1/IL1B/IL10/TNF	5
hsa04722	Neurotrophin signaling pathway	7/32	119/8085	3.11E-07	1.26E-06	2.65E-07	ABL1/AKT1/FASLG/BCL2/GSK3B/JUN/MAPK1	7
hsa01524	Platinum drug resistance	6/32	73/8085	3.31E-07	1.29E-06	2.72E-07	AKT1/FASLG/BCL2/CASP8/ERBB2/MAPK1	6
hsa05165	Human papillomavirus infection	10/32	331/8085	3.33E-07	1.29E-06	2.72E-07	AKT1/FASLG/CCND1/CASP8/CTNNB1/GSK3B/IRF1/MAPK1/PTGS2/TNF	10
hsa04919	Thyroid hormone signaling pathway	7/32	121/8085	3.49E-07	1.33E-06	2.79E-07	AKT1/CCND1/CTNNB1/GSK3B/MYC/MAPK1/SRC	7
hsa05220	Chronic myeloid leukemia	6/32	76/8085	4.22E-07	1.57E-06	3.31E-07	ABL1/AKT1/CCND1/MYC/MAPK1/TGFB1	6
hsa05219	Bladder cancer	5/32	41/8085	4.75E-07	1.73E-06	3.65E-07	CCND1/ERBB2/MYC/MAPK1/SRC	5
hsa04380	Osteoclast differentiation	7/32	128/8085	5.12E-07	1.84E-06	3.87E-07	AKT1/IL1A/IL1B/JUN/MAPK1/TGFB1/TNF	7
hsa05332	Graft-versus-host disease	5/32	42/8085	5.37E-07	1.89E-06	3.98E-07	FASLG/IL1A/IL1B/IL2/TNF	5
hsa04068	FoxO signaling pathway	7/32	131/8085	6.00E-07	2.06E-06	4.33E-07	AKT1/FASLG/CCND1/IGF1/IL10/MAPK1/TGFB1	7
hsa04940	Type I diabetes mellitus	5/32	43/8085	6.06E-07	2.06E-06	4.33E-07	FASLG/IL1A/IL1B/IL2/TNF	5
hsa05130	Pathogenic Escherichia coli infection	8/32	197/8085	6.86E-07	2.29E-06	4.82E-07	ABL1/FASLG/CASP8/IL1B/JUN/MAPK1/SRC/TNF	8
hsa05144	Malaria	5/32	50/8085	1.31E-06	4.29E-06	9.03E-07	ICAM1/IL1B/IL10/TGFB1/TNF	5
hsa05323	Rheumatoid arthritis	6/32	93/8085	1.40E-06	4.52E-06	9.51E-07	ICAM1/IL1A/IL1B/JUN/TGFB1/TNF	6
hsa04064	NF-kappa B signaling pathway	6/32	104/8085	2.71E-06	8.43E-06	1.78E-06	PARP1/BCL2/ICAM1/IL1B/PTGS2/TNF	6
hsa04620	Toll-like receptor signaling pathway	6/32	104/8085	2.71E-06	8.43E-06	1.78E-06	AKT1/CASP8/IL1B/JUN/MAPK1/TNF	6
hsa04370	VEGF signaling pathway	5/32	59/8085	3.02E-06	9.24E-06	1.95E-06	AKT1/KDR/MAPK1/PTGS2/SRC	5
hsa05225	Hepatocellular carcinoma	7/32	168/8085	3.21E-06	9.67E-06	2.04E-06	AKT1/CCND1/CTNNB1/GSK3B/MYC/MAPK1/TGFB1	7
hsa05416	Viral myocarditis	5/32	60/8085	3.28E-06	9.74E-06	2.05E-06	ABL1/CCND1/CASP8/CAV1/ICAM1	5

hsa05131	Shigellosis	8/32	247/8085	3.77E-06	1.10E-05	2.32E-06	AKT1/BCL2/GSK3B/IL1B/JUN/MAPK1/SRC/TNF	8
hsa04621	NOD-like receptor signaling pathway	7/32	186/8085	6.30E-06	1.81E-05	3.82E-06	BCL2/CASP8/HSP90AA1/IL1B/JUN/MAPK1/TNF	7
hsa05218	Melanoma	5/32	72/8085	8.13E-06	2.27E-05	4.78E-06	AKT1/CCND1/FGF2/IGF1/MAPK1	5
hsa05223	Non-small cell lung cancer	5/32	72/8085	8.13E-06	2.27E-05	4.78E-06	AKT1/CCND1/ERBB2/JAK3/MAPK1	5
hsa05212	Pancreatic cancer	5/32	76/8085	1.06E-05	2.92E-05	6.15E-06	AKT1/CCND1/ERBB2/MAPK1/TGFB1	5
hsa05216	Thyroid cancer	4/32	37/8085	1.22E-05	3.31E-05	6.96E-06	CCND1/CTNNB1/MYC/MAPK1	4
hsa05330	Allograft rejection	4/32	38/8085	1.36E-05	3.59E-05	7.56E-06	FASLG/IL2/IL10/TNF	4
hsa04915	Estrogen signaling pathway	6/32	138/8085	1.39E-05	3.59E-05	7.56E-06	AKT1/BCL2/HSP90AA1/JUN/MAPK1/SRC	6
hsa04015	Rap1 signaling pathway	7/32	210/8085	1.40E-05	3.59E-05	7.56E-06	AKT1/CTNNB1/FGF2/IGF1/KDR/MAPK1/SRC	7
hsa04060	Cytokine-cytokine receptor interaction	8/32	295/8085	1.40E-05	3.59E-05	7.56E-06	FASLG/IL1A/IL1B/IL2/IL10/IL13/TGFB1/TNF	8
hsa05170	Human immunodeficiency virus 1 infection	7/32	212/8085	1.49E-05	3.77E-05	7.93E-06	AKT1/FASLG/BCL2/CASP8/JUN/MAPK1/TNF	7
hsa04014	Ras signaling pathway	7/32	232/8085	2.67E-05	6.58E-05	1.39E-05	ABL1/AKT1/FASLG/FGF2/IGF1/KDR/MAPK1	7
hsa04658	Th1 and Th2 cell differentiation	5/32	92/8085	2.70E-05	6.58E-05	1.39E-05	IL2/IL13/JAK3/JUN/MAPK1	5
hsa05222	Small cell lung cancer	5/32	92/8085	2.70E-05	6.58E-05	1.39E-05	AKT1/CCND1/BCL2/MYC/PTGS2	5
hsa04218	Cellular senescence	6/32	156/8085	2.80E-05	6.74E-05	1.42E-05	AKT1/CCND1/IL1A/MYC/MAPK1/TGFB1	6
hsa04066	HIF-1 signaling pathway	5/32	109/8085	6.12E-05	0.000145	3.06E-05	AKT1/BCL2/ERBB2/IGF1/MAPK1	5
hsa04110	Cell cycle	5/32	124/8085	0.000113	0.000265	5.59E-05	ABL1/CCND1/GSK3B/MYC/TGFB1	5
hsa05203	Viral carcinogenesis	6/32	204/8085	0.000126	0.000291	6.12E-05	CCND1/CASP8/JAK3/JUN/MAPK1/SRC	6
hsa05221	Acute myeloid leukemia	4/32	67/8085	0.00013	0.000298	6.27E-05	AKT1/CCND1/MYC/MAPK1	4
hsa04926	Relaxin signaling pathway	5/32	129/8085	0.000136	0.000308	6.49E-05	AKT1/JUN/MAPK1/SRC/TGFB1	5
hsa04664	Fc epsilon RI signaling pathway	4/32	68/8085	0.000138	0.000308	6.49E-05	AKT1/IL13/MAPK1/TNF	4
hsa05211	Renal cell carcinoma	4/32	69/8085	0.000146	0.000322	6.79E-05	AKT1/JUN/MAPK1/TGFB1	4
hsa05230	Central carbon metabolism in cancer	4/32	70/8085	0.000154	0.000337	7.10E-05	AKT1/ERBB2/MYC/MAPK1	4
hsa04520	Adherens junction	4/32	71/8085	0.000163	0.000352	7.41E-05	CTNNB1/ERBB2/MAPK1/SRC	4
hsa05206	MicroRNAs in cancer	7/32	310/8085	0.000168	0.000358	7.53E-05	ABL1/CCND1/BCL2/ERBB2/MYC/MAPK1/PTGS2	7
hsa04115	p53 signaling pathway	4/32	73/8085	0.000182	0.000384	8.07E-05	CCND1/BCL2/CASP8/IGF1	4

hsa05214	Glioma	4/32	75/8085	0.000202	0.000421	8.87E-05	AKT1/CCND1/IGF1/MAPK1	4
hsa05310	Asthma	3/32	31/8085	0.000235	0.000485	0.000102	IL10/IL13/TNF	3
hsa04662	B cell receptor signaling pathway	4/32	82/8085	0.000285	0.000582	0.000122	AKT1/GSK3B/JUN/MAPK1	4
hsa04921	Oxytocin signaling pathway	5/32	154/8085	0.000312	0.000631	0.000133	CCND1/JUN/MAPK1/PTGS2/SRC	5
hsa04150	mTOR signaling pathway	5/32	155/8085	0.000322	0.000644	0.000135	AKT1/GSK3B/IGF1/MAPK1/TNF	5
hsa04390	Hippo signaling pathway	5/32	157/8085	0.000341	0.000676	0.000142	CCND1/CTNNB1/GSK3B/MYC/TGFB1	5
hsa04310	Wnt signaling pathway	5/32	166/8085	0.000442	0.000865	0.000182	CCND1/CTNNB1/GSK3B/JUN/MYC	5
hsa04350	TGF-beta signaling pathway	4/32	94/8085	0.00048	0.000931	0.000196	MYC/MAPK1/TGFB1/TNF	4
hsa05020	Prion disease	6/32	273/8085	0.000606	0.001154	0.000243	CAV1/GSK3B/IL1A/IL1B/MAPK1/TNF	6
hsa04914	Progesterone-mediated oocyte maturation	4/32	100/8085	0.000607	0.001154	0.000243	AKT1/HSP90AA1/IGF1/MAPK1	4
hsa05146	Amoebiasis	4/32	102/8085	0.000655	0.001231	0.000259	IL1B/IL10/TGFB1/TNF	4
hsa04062	Chemokine signaling pathway	5/32	192/8085	0.000857	0.001596	0.000336	AKT1/GSK3B/JAK3/MAPK1/SRC	5
hsa04672	Intestinal immune network for IgA production	3/32	49/8085	0.000917	0.001692	0.000356	IL2/IL10/TGFB1	3
hsa05320	Autoimmune thyroid disease	3/32	53/8085	0.001154	0.002091	0.00044	FASLG/IL2/IL10	3
hsa04071	Sphingolipid signaling pathway	4/32	119/8085	0.001167	0.002091	0.00044	AKT1/BCL2/MAPK1/TNF	4
hsa04935	Growth hormone synthesis, secretion and action	4/32	119/8085	0.001167	0.002091	0.00044	AKT1/GSK3B/IGF1/MAPK1	4
hsa04340	Hedgehog signaling pathway	3/32	56/8085	0.001354	0.002405	0.000506	CCND1/BCL2/GSK3B	3
hsa05134	Legionellosis	3/32	57/8085	0.001426	0.002508	0.000528	CASP8/IL1B/TNF	3
hsa04650	Natural killer cell mediated cytotoxicity	4/32	131/8085	0.001665	0.002889	0.000608	FASLG/ICAM1/MAPK1/TNF	4
hsa05208	Chemical carcinogenesis - reactive oxygen species	5/32	223/8085	0.001673	0.002889	0.000608	ABL1/AKT1/JUN/MAPK1/SRC	5
hsa05171	Coronavirus disease - COVID-19	5/32	232/8085	0.001992	0.003409	0.000718	IL1B/IL2/JUN/MAPK1/TNF	5
hsa05168	Herpes simplex virus 1 infection	7/32	498/8085	0.002816	0.004776	0.001006	AKT1/FASLG/BCL2/CASP8/IL1B/SRC/TNF	7
hsa04934	Cushing syndrome	4/32	155/8085	0.003076	0.005173	0.001089	CCND1/CTNNB1/GSK3B/MAPK1	4
hsa05100	Bacterial invasion of epithelial cells	3/32	77/8085	0.003378	0.00563	0.001185	CAV1/CTNNB1/SRC	3
hsa04530	Tight junction	4/32	169/8085	0.004196	0.006933	0.00146	CCND1/ERBB2/JUN/SRC	4

hsa05235	PD-L1 expression and PD-1 checkpoint pathway in cancer	3/32	89/8085	0.005078	0.008318	0.001751	AKT1/JUN/MAPK1	3
hsa05410	Hypertrophic cardiomyopathy	3/32	90/8085	0.005239	0.008508	0.001791	IGF1/TGFB1/TNF	3
hsa04360	Axon guidance	4/32	182/8085	0.005459	0.008789	0.00185	ABL1/GSK3B/MAPK1/SRC	4
hsa04912	GnRH signaling pathway	3/32	93/8085	0.005741	0.009166	0.00193	JUN/MAPK1/SRC	3
hsa05414	Dilated cardiomyopathy	3/32	96/8085	0.006271	0.009929	0.00209	IGF1/TGFB1/TNF	3
hsa01523	Antifolate resistance	2/32	31/8085	0.00657	0.010256	0.002159	IL1B/TNF	2
hsa04750	Inflammatory mediator regulation of TRP channels	3/32	98/8085	0.00664	0.010256	0.002159	IGF1/IL1B/SRC	3
hsa05231	Choline metabolism in cancer	3/32	98/8085	0.00664	0.010256	0.002159	AKT1/JUN/MAPK1	3
hsa04640	Hematopoietic cell lineage	3/32	99/8085	0.006829	0.010464	0.002203	IL1A/IL1B/TNF	3
hsa04215	Apoptosis - multiple species	2/32	32/8085	0.006991	0.010588	0.002229	BCL2/CASP8	2
hsa04061	Viral protein interaction with cytokine and cytokine receptor	3/32	100/8085	0.007021	0.010588	0.002229	IL2/IL10/TNF	3
hsa04916	Melanogenesis	3/32	101/8085	0.007217	0.010797	0.002273	CTNNB1/GSK3B/MAPK1	3
hsa05415	Diabetic cardiomyopathy	4/32	203/8085	0.008	0.011875	0.0025	PARP1/AKT1/GSK3B/TGFB1	4
hsa04931	Insulin resistance	3/32	108/8085	0.008677	0.012781	0.002691	AKT1/GSK3B/TNF	3
hsa04960	Aldosterone-regulated sodium reabsorption	2/32	37/8085	0.009272	0.013551	0.002853	IGF1/MAPK1	2
hsa04725	Cholinergic synapse	3/32	113/8085	0.00982	0.014242	0.002998	AKT1/BCL2/MAPK1	3
hsa04152	AMPK signaling pathway	3/32	120/8085	0.011561	0.01664	0.003503	AKT1/CCND1/IGF1	3
hsa04611	Platelet activation	3/32	124/8085	0.012631	0.018044	0.003799	AKT1/MAPK1/SRC	3
hsa04930	Type II diabetes mellitus	2/32	46/8085	0.014094	0.019984	0.004207	MAPK1/TNF	2
hsa04910	Insulin signaling pathway	3/32	137/8085	0.016493	0.023212	0.004887	AKT1/GSK3B/MAPK1	3
hsa04371	Apelin signaling pathway	3/32	138/8085	0.016815	0.023491	0.004946	AKT1/CCND1/MAPK1	3
hsa04913	Ovarian steroidogenesis	2/32	51/8085	0.01715	0.023785	0.005007	IGF1/PTGS2	2
hsa04140	Autophagy - animal	3/32	141/8085	0.017801	0.024509	0.00516	AKT1/BCL2/MAPK1	3
hsa04261	Adrenergic signaling in cardiomyocytes	3/32	150/8085	0.020952	0.02864	0.006029	AKT1/BCL2/MAPK1	3

hsa04923	Regulation of lipolysis in adipocytes	2/32	57/8085	0.021155	0.028711	0.006044	AKT1/PTGS2	2
hsa04730	Long-term depression	2/32	60/8085	0.02329	0.031384	0.006607	IGF1/MAPK1	2
hsa04213	Longevity regulating pathway - multiple species	2/32	62/8085	0.024761	0.033131	0.006975	AKT1/IGF1	2
hsa05217	Basal cell carcinoma	2/32	63/8085	0.02551	0.033895	0.007136	CTNNB1/GSK3B	2
hsa04929	GnRH secretion	2/32	64/8085	0.026269	0.03466	0.007297	AKT1/MAPK1	2
hsa04920	Adipocytokine signaling pathway	2/32	69/8085	0.030198	0.03957	0.00833	AKT1/TNF	2
hsa04622	RIG-I-like receptor signaling pathway	2/32	70/8085	0.03101	0.040082	0.008438	CASP8/TNF	2
hsa05120	Epithelial cell signaling in Helicobacter pylori infection	2/32	70/8085	0.03101	0.040082	0.008438	JUN/SRC	2
hsa04137	Mitophagy - animal	2/32	72/8085	0.032661	0.04193	0.008827	JUN/SRC	2
hsa04612	Antigen processing and presentation	2/32	78/8085	0.037816	0.048222	0.010152	HSP90AA1/TNF	2
hsa04613	Neutrophil extracellular trap formation	3/32	190/8085	0.038459	0.048715	0.010256	AKT1/MAPK1/SRC	3
