# Supplementary material for: Exploring Molecular Mechanisms of Aloe barbadmsis Miller on Diphenoxylate-Induced Constipation in Mice
Source: Evid Based Complement Alternat Med. 2022 May 6;2022:6225758. doi: 10.1155/2022/6225758 (PMC9106447; doi:10.1155/2022/6225758)
Supplement: Supplementary Materials — Table S1. Active ingredients of Aloe. Table S2. Potential targets related to active ingredients. Table S3. potential targets related to constipation. Table S4. Common targets related to active ingredients. Table S5. Table S5-1. Detailed information of BP enrichment of PPI network cluster 1 targets; Table S5-2. Detailed information of CC enrichment of PPI network cluster 1 targets; Table S5-3. Detailed information of MF enrichment of PPI network cluster 1 targets; Table S5-4. Detailed information of KEGG pathways enrichment of PPI network cluster 1 targets. Table S6. Table S6-1. Detailed information of BP enrichment of common targets; Table S6-2. Detailed information of CC enrichment of common targets; Table S6-3. Detailed information of MF enrichment of common targets; Table S6-4. Detailed information of KEGG pathways enrichment of common targets. Table S7. Original images of H&E staining in colon of three repeats in each group. Table S8. Raw data of 5-HT, SP, and VIP in serum and colon determined by ELISA kits. Table S9. Raw data of NF-κB p65, AKT, ERK, and JNK in colon determined by RT-PCR method. Table S10. Original images of ERK, JNK, AKT, and NF-κB p65 in colon of Western Blot, and its raw data quantification. [file 6225758.f1.zip › suppl table 1-10/Table S6 (1) (1).pdf]

Supplementary Table S6-1: Datailed information of biological process(BP)enrichment of common targets

ID	Description	GeneRatio	BgRatio	pvalue	p.adjust	qvalue	geneID	[illegible]
GO:0046677	response to antibiotic	39/149	327/18670	6.73E-35	3.01E-31	1.10E-31	MET/LEP/COL1A1/BCL2/CYP1A1/HTR3A/CASP8/OPRK1/NFE2L2/DRD2/GRIN2B/TRPA1/CCND1/CYP2E1/S100A8/IL13/STAT1/IL6/HSP90AA1/CASP3/HMGCR/HMOX1/MAOB/IL10/NQO1/SOD1/IL2/GRIN1/ICAM1/ABL1/DRD4/CFTR/SRC/TP53/CASP9/CAT/SPARC/JUN/AVP	
GO:0072593	reactive oxygen species metabolic process	33/149	284/18670	4.28E-29	9.58E-26	3.50E-26	NOS2/LEP/NOS1/BCL2/CYP1A1/DUOX2/CRP/MAPT/NFE2L2/TNF/MPO/F2/PTGS2/SNCA/TGFB1/TRPV1/HSP90AA1/INS/MAOB/IL10/EGFR/NQO1/SOD1/GRIN1/IFNG/ICAM1/CAV1/TPO/TP53/CDKN1A/CAT/AKT1/IL1B	
GO:0097305	response to alcohol	29/149	233/18670	1.83E-26	2.74E-23	1.00E-23	LEP/HTR3A/CASP8/OPRK1/PPARG/DRD2/GRIN2B/CTNNB1/CCND1/CYP2E1/TGFB1/S100A8/IL13/PARP1/HMGCR/MAOB/NQO1/SOD1/IL2/GRIN1/FOS/ICAM1/DRD4/CFTR/CDKN1A/CAT/AKT1/SPARC/AVP	
GO:0006979	response to oxidative stress	36/149	451/18670	5.82E-26	6.52E-23	2.39E-23	MMP2/MET/COL1A1/APOE/BCL2/DUOX2/MAPT/NFE2L2/TNF/MPO/TRPA1/PTGS2/CTNNB1/CYP2E1/SNCA/STAT1/IL6/CASP3/PARP1/HMOX1/INS/IL10/MAPK1/EGFR/NQO1/SOD1/FOS/ABL1/TPO/SRC/TP53/CAT/AKT1/PTGS1/JUN/MMP9	
GO:0032496	response to lipopolysaccharide	32/149	330/18670	1.04E-25	9.36E-23	3.42E-23	NOS2/NOS1/CYP1A1/CASP8/OPRK1/CXCL8/TNF/MPO/PTGS2/SNCA/TGFB1/S100A8/IL13/IL6/CASP3/FASLG/GGT1/MAOB/IL10/MAPK1/FOS/ICAM1/ABL1/SRC/CASP9/AKT1/NFKB1/IL1B/NFKBIA/SPARC/JUN/NOTCH1	
GO:0002237	response to molecule of bacterial origin	32/149	343/18670	3.53E-25	2.63E-22	9.63E-23	NOS2/NOS1/CYP1A1/CASP8/OPRK1/CXCL8/TNF/MPO/PTGS2/SNCA/TGFB1/S100A8/IL13/IL6/CASP3/FASLG/GGT1/MAOB/IL10/MAPK1/FOS/ICAM1/ABL1/SRC/CASP9/AKT1/NFKB1/IL1B/NFKBIA/SPARC/JUN/NOTCH1	
GO:0031667	response to nutrient levels	36/149	499/18670	1.91E-24	1.22E-21	4.48E-22	SLC6A4/LEP/COL1A1/APOE/FAS/PON1/BCHE/BCL2/CYP1A1/PPARG/NFE2L2/MPO/PTGS2/P2RY12/CCND1/TGFB1/STAT1/TRPV1/HMGCR/HMOX1/MAPK1/EGFR/NQO1/SOD1/GRIN1/ICAM1/SRC/TP53/CDKN1A/CAT/AKT1/IL1B/SPARC/JUN/SNAI2/CYP24A1	
GO:0000302	response to reactive oxygen species	27/149	232/18670	7.16E-24	4.01E-21	1.47E-21	MMP2/MET/COL1A1/APOE/BCL2/MAPT/NFE2L2/TNF/MPO/TRPA1/CYP2E1/STAT1/IL6/CASP3/HMOX1/IL10/MAPK1/EGFR/NQO1/SOD1/FOS/ABL1/SRC/CAT/AKT1/JUN/MMP9	
GO:0043270	positive regulation of ion transport	28/149	275/18670	4.08E-23	2.03E-20	7.43E-21	SLC6A4/LEP/NOS1/APOE/ADRA2A/HTR3A/OPRK1/ABCB1/PLA2G6/DRD2/F2/P2RY12/SNCA/SHANK3/IL13/KCNQ1/MYLK/GRIN1/IFNG/CAV1/ABL1/DRD4/CFTR/AKT1/IL1B/BAX/SCN5A/AVP	
GO:0035690	cellular response to drug	31/149	369/18670	5.07E-23	2.27E-20	8.31E-21	NOS2/MET/NFE2L2/SOX9/TNF/PTGS2/CTNNB1/P2RY12/TGFB1/MYC/IL6/KCNQ1/TRPV1/KDR/HMOX1/FMR1/IL10/MAPK1/EGFR/NQO1/SOD1/ICAM1/ABL1/CACNA1S/CFTR/SRC/TP53/CASP9/NFKB1/IL1B/CHRM3	
GO:2000379	positive regulation of reactive oxygen species metabolic process	20/149	102/18670	1.41E-22	5.75E-20	2.10E-20	LEP/CRP/MAPT/NFE2L2/TNF/F2/PTGS2/SNCA/TGFB1/TRPV1/HSP90AA1/EGFR/SOD1/GRIN1/IFNG/ICAM1/TP53/CDKN1A/AKT1/IL1B	
GO:1903409	reactive oxygen species biosynthetic process	21/149	122/18670	2.13E-22	7.95E-20	2.91E-20	NOS2/NOS1/CYP1A1/DUOX2/TNF/MPO/PTGS2/SNCA/TRPV1/HSP90AA1/INS/MAOB/IL10/NQO1/SOD1/GRIN1/IFNG/ICAM1/CAV1/AKT1/IL1B	
GO:0045471	response to ethanol	21/149	125/18670	3.64E-22	1.26E-19	4.60E-20	LEP/HTR3A/CASP8/OPRK1/DRD2/GRIN2B/CCND1/CYP2E1/S100A8/IL13/HMGCR/MAOB/NQO1/SOD1/IL2/GRIN1/ICAM1/DRD4/CAT/SPARC/AVP	
GO:0042391	regulation of membrane potential	32/149	434/18670	5.15E-22	1.65E-19	6.03E-20	BCL2/HTR3A/CACNA1B/MAPT/DRD2/GRIN2B/GABRG3/SNCA/SHANK3/NRXN1/GSK3B/KCNQ1/TRPV1/PARP1/CACNA1C/KDR/FMR1/SOD1/CACNA1A/GRIN1/GABRD/CAV1/ABL1/DRD4/CFTR/SRC/AKT1/SCN10A/JUN/GABRA3/BAX/SCN5A	
GO:2000377	regulation of reactive oxygen species metabolic process	24/149	195/18670	7.10E-22	2.12E-19	7.75E-20	LEP/BCL2/CRP/MAPT/NFE2L2/TNF/F2/PTGS2/SNCA/TGFB1/TRPV1/HSP90AA1/INS/IL10/EGFR/SOD1/GRIN1/IFNG/ICAM1/CAV1/TP53/CDKN1A/AKT1/IL1B	

GO:0003018	vascular process in circulatory system	23/149	173/18670	8.89E-22	2.49E-19	9.10E-20	SLC6A4/LEP/NOS1/APOE/ADRA2A/CRP/PLA2G6/PTGS2/TGFB1/AVPR2/HMGCR/HMOX1/INS/HTR1A/EGFR/SOD1/ICAM1/CAV1/SRC/AKT1/CHRM3/HTR2A/AVP
GO:0009612	response to mechanical stimulus	24/149	210/18670	4.25E-21	1.12E-18	4.10E-19	IRF1/COL1A1/FAS/CASP8/PPARG/SOX9/DRD2/MPO/TRPA1/PTGS2/TGFB1/SHANK3/NRXN1/IL13/STAT1/EGFR/FOS/SRC/AKT1/NFKB1/IL1B/NFKBIA/HTR2A/JUN
GO:0050673	epithelial cell proliferation	31/149	434/18670	6.30E-21	1.41E-18	5.15E-19	IGF1/LEP/APOE/PPARG/SOX9/TNF/CTNNB1/CCND1/ESR1/TGFB1/MYC/STAT1/KDR/HMOX1/IL10/MAPK1/EGFR/NODAL/ERBB2/FGF2/CAV1/RB1/PGR/AKT1/SPARC/IGFBP3/JUN/SNAI2/NOTCH1/BAX/SCN5A
GO:0010038	response to metal ion	29/149	364/18670	6.52E-21	1.41E-18	5.15E-19	BCL2/CYP1A1/CASP8/MAPT/NFE2L2/IL1A/DRD2/PTGS2/CCND1/SNCA/S100A8/CASP3/PARP1/HMOX1/MAOB/MAPK1/EGFR/NQO1/SOD1/FOS/ICAM1/CAV1/CASP9/CAT/AKT1/SPARC/JUN/SCN5A/MMP9
GO:0035296	regulation of tube diameter	21/149	143/18670	6.91E-21	1.41E-18	5.15E-19	SLC6A4/LEP/NOS1/APOE/ADRA2A/CRP/PLA2G6/PTGS2/AVPR2/HMGCR/HMOX1/INS/HTR1A/EGFR/SOD1/ICAM1/CAV1/AKT1/CHRM3/HTR2A/AVP
GO:0050880	regulation of blood vessel size	21/149	143/18670	6.91E-21	1.41E-18	5.15E-19	SLC6A4/LEP/NOS1/APOE/ADRA2A/CRP/PLA2G6/PTGS2/AVPR2/HMGCR/HMOX1/INS/HTR1A/EGFR/SOD1/ICAM1/CAV1/AKT1/CHRM3/HTR2A/AVP
GO:0097746	regulation of blood vessel diameter	21/149	143/18670	6.91E-21	1.41E-18	5.15E-19	SLC6A4/LEP/NOS1/APOE/ADRA2A/CRP/PLA2G6/PTGS2/AVPR2/HMGCR/HMOX1/INS/HTR1A/EGFR/SOD1/ICAM1/CAV1/AKT1/CHRM3/HTR2A/AVP
GO:0035150	regulation of tube size	21/149	144/18670	8.04E-21	1.57E-18	5.73E-19	SLC6A4/LEP/NOS1/APOE/ADRA2A/CRP/PLA2G6/PTGS2/AVPR2/HMGCR/HMOX1/INS/HTR1A/EGFR/SOD1/ICAM1/CAV1/AKT1/CHRM3/HTR2A/AVP
GO:0071496	cellular response to external stimulus	28/149	339/18670	1.25E-20	2.34E-18	8.56E-19	IRF1/LEP/COL1A1/FAS/BCL2/CASP8/PPARG/NFE2L2/SOX9/PTGS2/P2RY12/TGFB1/IL13/TRPV1/HMOX1/MAPK1/EGFR/SOD1/FOS/ICAM1/TP53/CDKN1A/AKT1/NFKB1/IL1B/JUN/SNAI2/CYP24A1
GO:0097191	extrinsic apoptotic signaling pathway	24/149	224/18670	2.00E-20	3.58E-18	1.31E-18	IGF1/FAS/BCL2/CASP8/RET/TNF/IL1A/TGFB1/GSK3B/CASP3/FASLG/HMOX1/IL2/IFNG/ICAM1/CAV1/SRC/ACVR1B/CASP9/AKT1/PIK3R1/IL1B/SNAI2/BAX
GO:0070997	neuron death	28/149	348/18670	2.54E-20	4.38E-18	1.60E-18	APOE/BCL2/THRB/CASP8/MAPT/TNF/GRIN2B/CTNNB1/SNCA/IL13/GSK3B/CASP3/PARP1/FASLG/HMOX1/IL10/NQO1/SOD1/GRIN1/IFNG/FOS/ABL1/RB1/TP53/CASP9/AKT1/JUN/BAX
GO:0048545	response to steroid hormone	29/149	385/18670	3.10E-20	5.14E-18	1.88E-18	COL1A1/BCHE/BCL2/THRB/PPARG/TNF/PTGS2/CTNNB1/CCND1/ESR1/TGFB1/IL6/CASP3/PARP1/MAOB/IL10/EGFR/NODAL/FOS/ICAM1/CAV1/RB1/SRC/PGR/THRA/CASP9/CDKN1A/SPARC/NOTCH1
GO:1901216	positive regulation of neuron death	18/149	94/18670	3.23E-20	5.17E-18	1.89E-18	CASP8/MAPT/TNF/GRIN2B/CTNNB1/SNCA/GSK3B/CASP3/PARP1/FASLG/NQO1/IFNG/FOS/ABL1/TP53/CASP9/JUN/BAX
GO:0001505	regulation of neurotransmitter levels	28/149	354/18670	4.02E-20	6.21E-18	2.27E-18	SLC6A2/NOS2/SLC6A4/NOS1/BCHE/CACNA1B/TNF/DRD2/PTGS2/SNCA/NRXN1/ACHE/GSK3B/TRPV1/HSP90AA1/HTR1A/FMR1/MAOB/IL10/NQO1/IFNG/ICAM1/CAV1/DRD4/AKT1/IL1B/HTR2A/PAH
GO:0006816	calcium ion transport	30/149	434/18670	7.39E-20	1.10E-17	4.04E-18	NOS1/BCL2/ADRA2A/CACNA1B/PLA2G6/DRD2/GRIN2B/F2/TRPA1/PTGS2/CTNNB1/P2RY12/SNCA/TGFB1/IL13/TRPV1/CACNA1C/FASLG/FMR1/MYLK/CACNA1A/GRIN1/FGF2/ICAM1/CAV1/ABL1/CACNA1S/DRD4/HTR2A/BAX
GO:0051235	maintenance of location	27/149	330/18670	8.38E-20	1.21E-17	4.43E-18	LEP/NOS1/APOE/PPARG/CRP/TNF/DRD2/F2/TRPA1/SNCA/TGFB1/S100A8/IL13/IL6/TRPV1/CACNA1C/FASLG/IL10/FGF2/CAV1/ABL1/AKT1/NFKB1/IL1B/NFKBIA/HTR2A/BAX
GO:0034599	cellular response to oxidative stress	26/149	302/18670	1.20E-19	1.68E-17	6.15E-18	MMP2/MET/BCL2/MAPT/NFE2L2/TNF/MPO/CTNNB1/SNCA/IL6/PARP1/HMOX1/INS/IL10/MAPK1/EGFR/NQO1/SOD1/FOS/ABL1/SRC/TP53/CAT/AKT1/JUN/MMP9
GO:2001233	regulation of apoptotic signaling pathway	29/149	406/18670	1.34E-19	1.82E-17	6.65E-18	IGF1/FAS/BCL2/CASP8/RET/NFE2L2/PLA2G6/TNF/IL1A/PTGS2/CTNNB1/S100A8/GSK3B/PARP1/FASLG/HMOX1/INS/SOD1/ICAM1/CAV1/RB1/SRC/TP53/AKT1/IL1B/SNAI2/BAX/AVP/MMP9

GO:0070838	divalent metal ion transport	31/149	483/18670	1.43E-19	1.88E-17	6.89E-18	NOS1/BCL2/ADRA2A/CACNA1B/PLA2G6/DRD2/GRIN2B/F2/TRPA1/PTGS2/CTNNB1/P2RY12/SNCA/TGFB1/IL13/TRPV1/CACNA1C/FASLG/FMR1/MYLK/CACNA1A/GRIN1/IFNG/FGF2/ICAM1/CAV1/ABL1/CACNA1S/DRD4/HTR2A/BAX
GO:0072511	divalent inorganic cation transport	31/149	489/18670	2.04E-19	2.59E-17	9.47E-18	NOS1/BCL2/ADRA2A/CACNA1B/PLA2G6/DRD2/GRIN2B/F2/TRPA1/PTGS2/CTNNB1/P2RY12/SNCA/TGFB1/IL13/TRPV1/CACNA1C/FASLG/FMR1/MYLK/CACNA1A/GRIN1/IFNG/FGF2/ICAM1/CAV1/ABL1/CACNA1S/DRD4/HTR2A/BAX
GO:0007584	response to nutrient	23/149	219/18670	2.08E-19	2.59E-17	9.47E-18	SLC6A4/LEP/COL1A1/BCHE/CYP1A1/PPARG/PTGS2/P2RY12/CCND1/TGFB1/STAT1/TRPV1/HMGCR/HMOX1/EGFR/NQO1/SOD1/GRIN1/CAT/IL1B/SPARC/SNAI2/CYP24A1
GO:0050678	regulation of epithelial cell proliferation	28/149	378/18670	2.33E-19	2.82E-17	1.03E-17	IGF1/LEP/APOE/PPARG/SOX9/TNF/CTNNB1/CCND1/TGFB1/MYC/STAT1/KDR/HMOX1/IL10/EGFR/NODAL/ERBB2/FGF2/CAV1/RB1/PGR/AKT1/SPARC/JUN/SNAI2/NOTCH1/BAX/SCN5A
GO:1901214	regulation of neuron death	26/149	313/18670	2.95E-19	3.48E-17	1.27E-17	APOE/BCL2/CASP8/MAPT/TNF/GRIN2B/CTNNB1/SNCA/IL13/GSK3B/CASP3/PARP1/FASLG/HMOX1/IL10/NQO1/SOD1/GRIN1/IFNG/FOS/ABL1/TP53/CASP9/AKT1/JUN/BAX
GO:0010959	regulation of metal ion transport	28/149	394/18670	7.00E-19	8.04E-17	2.94E-17	NOS1/BCL2/ADRA2A/OPRK1/CACNA1B/PLA2G6/DRD2/F2/PTGS2/CTNNB1/P2RY12/SNCA/TGFB1/IL13/KCNQ1/CACNA1C/FMR1/MYLK/GRIN1/IFNG/ICAM1/CAV1/ABL1/DRD4/AKT1/HTR2A/BAX/SCN5A
GO:0048732	gland development	29/149	434/18670	8.30E-19	9.29E-17	3.40E-17	MET/BCL2/THRB/CYP1A1/SOX9/TNF/DRD2/CTNNB1/CCND1/ESR1/TGFB1/NKX2-1/HMOX1/IL10/MAPK1/IGF2/EGFR/NODAL/SOD1/CAV1/ABL1/SRC/PGR/THRA/AKT1/JUN/SNAI2/NOTCH1/BAX
GO:0034765	regulation of ion transmembrane transport	30/149	483/18670	1.49E-18	1.63E-16	5.97E-17	NOS1/ADRA2A/HTR3A/OPRK1/CACNA1B/ABCB1/PLA2G6/DRD2/GRIN2B/F2/SNCA/TGFB1/SHANK3/IL13/KCNQ1/CACNA1C/FMR1/CACNA1A/GRIN1/IFNG/CAV1/ABL1/CACNA1S/DRD4/CFTR/AKT1/SCN10A/BAX/SCN5A/MMP9
GO:0019216	regulation of lipid metabolic process	28/149	410/18670	2.00E-18	2.14E-16	7.82E-17	LEP/APOE/ADRA2A/CYP1A1/PPARG/PLA2G6/TNF/F2/PTGS2/P2RY12/SNCA/TGFB1/HMGCR/INS/SOD1/IFNG/FGF2/CAV1/RB1/SRC/THRA/AKT1/PIK3R1/NFKB1/IL1B/HTR2A/SNAI2/AVP
GO:0031960	response to corticosteroid	20/149	162/18670	2.14E-18	2.23E-16	8.15E-17	COL1A1/BCHE/BCL2/TNF/PTGS2/CCND1/TGFB1/IL6/CASP3/PARP1/MAOB/IL10/EGFR/FOS/ICAM1/SRC/CASP9/CDKN1A/SPARC/NOTCH1
GO:0002791	regulation of peptide secretion	30/149	500/18670	3.91E-18	3.98E-16	1.46E-16	IGF1/NOS2/LEP/DPP4/APOE/ADRA2A/CRP/PLA2G6/TNF/IL1A/DRD2/TGFB1/S100A8/IL13/ACHE/IL6/HMGCR/CACNA1C/INS/IL10/EGFR/SLC25A4/IL2/CACNA1A/IFNG/ABL1/DRD4/CFTR/SRC/IL1B
GO:0097756	negative regulation of blood vessel diameter	16/149	84/18670	4.78E-18	4.76E-16	1.74E-16	SLC6A4/LEP/ADRA2A/CRP/PTGS2/AVPR2/HMGCR/INS/HTR1A/EGFR/ICAM1/CAV1/AKT1/CHRM3/HTR2A/AVP
GO:0048660	regulation of smooth muscle cell proliferation	20/149	169/18670	5.01E-18	4.88E-16	1.79E-16	MMP2/IGF1/PPARG/TNF/PTGS2/CTNNB1/IL13/STAT1/IL6/HMGCR/HMOX1/IL10/EGFR/IFNG/FGF2/CDKN1A/AKT1/IGFBP3/JUN/MMP9
GO:0048659	smooth muscle cell proliferation	20/149	171/18670	6.35E-18	6.05E-16	2.21E-16	MMP2/IGF1/PPARG/TNF/PTGS2/CTNNB1/IL13/STAT1/IL6/HMGCR/HMOX1/IL10/EGFR/IFNG/FGF2/CDKN1A/AKT1/IGFBP3/JUN/MMP9
GO:0070482	response to oxygen levels	27/149	394/18670	7.98E-18	7.35E-16	2.69E-16	MMP2/PLAU/NOS2/SLC6A4/LEP/NOS1/COL1A1/DPP4/FAS/BCL2/CYP1A1/PPARG/NFE2L2/DRD2/PTGS2/TGFB1/MYC/CASP3/HMOX1/ICAM1/CAV1/SRC/TP53/CDKN1A/CAT/AKT1/NOTCH1
GO:0050708	regulation of protein secretion	29/149	472/18670	8.04E-18	7.35E-16	2.69E-16	IGF1/NOS2/LEP/DPP4/APOE/ADRA2A/CRP/PLA2G6/TNF/IL1A/DRD2/TGFB1/IL13/ACHE/IL6/HMGCR/CACNA1C/INS/IL10/EGFR/SLC25A4/IL2/CACNA1A/IFNG/ABL1/DRD4/CFTR/SRC/IL1B
GO:0045862	positive regulation of proteolysis	26/149	363/18670	1.17E-17	1.05E-15	3.84E-16	APOE/FAS/ADRA2A/CASP8/PPARG/MAPT/NFE2L2/TNF/GRIN2B/SNCA/CTSD/MYC/S100A8/GSK3B/FASLG/FMR1/NODAL/GRIN1/IFNG/CAV1/SRC/CASP9/AKT1/IL1B/F12/BAX
GO:0042133	neurotransmitter metabolic process	19/149	153/18670	1.42E-17	1.25E-15	4.57E-16	NOS2/SLC6A4/NOS1/BCHE/TNF/PTGS2/ACHE/TRPV1/HSP90AA1/HTR1A/MAOB/IL10/NQO1/IFNG/ICAM1/CAV1/AKT1/IL1B/PAH

GO:1903522	regulation of blood circulation	24/149	297/18670	1.49E-17	1.29E-15	4.70E-16	LEP/NOS1/THRB/ADRA2A/CACNA1B/DRD2/PTGS2/AVPR2/KCNQ1/TRPV1/ABCC9/CACNA1C/EGFR/IL2/ICAM1/CAV1/CACNA1S/THRA/AKT1/CHRM3/HTR2A/SCN10A/SCN5A/AVP
GO:0033002	muscle cell proliferation	22/149	239/18670	2.32E-17	1.96E-15	7.16E-16	MMP2/IGF1/PPARG/TNF/PTGS2/CTNNB1/IL13/STAT1/IL6/HMGCR/HMOX1/IL10/MAPK1/EGFR/IFNG/FGF2/CDKN1A/AKT1/IGFBP3/JUN/NOTCH1/MMP9
GO:0062012	regulation of small molecule metabolic process	28/149	459/18670	3.83E-17	3.18E-15	1.16E-15	IGF1/NOS2/LEP/NOS1/APOE/PPARG/TNF/PTGS2/SNCA/TGFB1/GSK3B/PARP1/HMGCR/INS/IGF2/NQO1/SOD1/IFNG/CAV1/SRC/TP53/AKT1/NFKB1/IL1B/IGFBP3/HTR2A/SNAI2/AVP
GO:0070588	calcium ion transmembrane transport	24/149	315/18670	5.76E-17	4.69E-15	1.72E-15	NOS1/ADRA2A/CACNA1B/PLA2G6/DRD2/GRIN2B/F2/TRPA1/P2RY12/SNCA/TGFB1/IL13/TRPV1/CACNA1C/FASLG/FMR1/CACNA1A/GRIN1/FGF2/ABL1/CACNA1S/DRD4/HTR2A/BAX
GO:0051924	regulation of calcium ion transport	22/149	254/18670	8.50E-17	6.80E-15	2.49E-15	NOS1/BCL2/ADRA2A/CACNA1B/PLA2G6/DRD2/F2/PTGS2/CTNNB1/P2RY12/SNCA/TGFB1/IL13/CACNA1C/FMR1/MYLK/GRIN1/ICAM1/CAV1/ABL1/DRD4/BAX
GO:0007568	aging	24/149	321/18670	8.86E-17	6.97E-15	2.55E-15	LEP/BCL2/CYP1A1/SLC12A2/NFE2L2/MPO/PTGS2/SNCA/TGFB1/HMGCR/IL10/MAPK1/NQO1/SOD1/FOS/ICAM1/ABL1/TP53/CASP9/CDKN1A/CAT/AKT1/HTR2A/JUN
GO:2001234	negative regulation of apoptotic signaling pathway	21/149	230/18670	1.53E-16	1.18E-14	4.33E-15	IGF1/FAS/BCL2/CASP8/NFE2L2/TNF/IL1A/PTGS2/CTNNB1/FASLG/HMOX1/INS/ICAM1/RB1/SRC/AKT1/IL1B/SNAI2/BAX/AVP/MMP9
GO:0048871	multicellular organismal homeostasis	28/149	485/18670	1.59E-16	1.20E-14	4.41E-15	MET/LEP/PRDM16/BCL2/SOX9/TNF/IL1A/DRD2/PTGS2/CTNNB1/AVPR2/IL13/ACHE/IL6/TRPV1/EGFR/SOD1/CAV1/RB1/CFTR/SRC/THRA/IL1B/HTR2A/GATM/NOTCH1/BAX/AVP
GO:0050890	cognition	23/149	296/18670	1.77E-16	1.29E-14	4.71E-15	SLC6A4/APOE/BCHE/OPRK1/MAPT/PLA2G6/TNF/DRD2/GRIN2B/PTGS2/SHANK3/NRXN1/CASP3/HMGCR/INS/MAPK1/EGFR/GRIN1/FOS/THRA/HTR2A/JUN/GATM
GO:0071214	cellular response to abiotic stimulus	24/149	331/18670	1.78E-16	1.29E-14	4.71E-15	IRF1/COL1A1/FAS/CASP8/SOX9/PTGS2/TGFB1/MYC/IL13/TRPV1/CASP3/PARP1/FMR1/EGFR/MYLK/TP53/CASP9/CDKN1A/AKT1/PIK3R1/NFKB1/IL1B/SNAI2/BAX
GO:0104004	cellular response to environmental stimulus	24/149	331/18670	1.78E-16	1.29E-14	4.71E-15	IRF1/COL1A1/FAS/CASP8/SOX9/PTGS2/TGFB1/MYC/IL13/TRPV1/CASP3/PARP1/FMR1/EGFR/MYLK/TP53/CASP9/CDKN1A/AKT1/PIK3R1/NFKB1/IL1B/SNAI2/BAX
GO:0042136	neurotransmitter biosynthetic process	16/149	106/18670	2.38E-16	1.69E-14	6.19E-15	NOS2/SLC6A4/NOS1/TNF/PTGS2/ACHE/TRPV1/HSP90AA1/IL10/NQO1/IFNG/ICAM1/CAV1/AKT1/IL1B/PAH
GO:1904951	positive regulation of establishment of protein localization	27/149	456/18670	3.14E-16	2.20E-14	8.05E-15	IGF1/LEP/BCL2/CASP8/PLA2G6/TNF/IL1A/DRD2/PTGS2/TGFB1/IL13/ACHE/GSK3B/IL6/INS/IL10/MAPK1/EGFR/IL2/ERBB2/IFNG/ABL1/CFTR/SRC/TP53/PIK3R1/IL1B
GO:0042110	T cell activation	27/149	464/18670	4.84E-16	3.33E-14	1.22E-14	IGF1/IRF1/LEP/DPP4/BCL2/CASP8/CTNNB1/TGFB1/IL6/CASP3/INS/IL10/IGF2/SOD1/IL2/ERBB2/IFNG/ICAM1/CAV1/ABL1/SRC/TP53/JAK3/AKT1/PIK3R1/IL1B/BAX
GO:0060402	calcium ion transport into cytosol	18/149	158/18670	5.03E-16	3.41E-14	1.25E-14	NOS1/BCL2/DRD2/GRIN2B/F2/TRPA1/SNCA/TGFB1/IL13/TRPV1/CACNA1C/FASLG/GRIN1/FGF2/CAV1/ABL1/HTR2A/BAX
GO:0070371	ERK1 and ERK2 cascade	23/149	317/18670	7.90E-16	5.28E-14	1.93E-14	IGF1/PLA2G2A/APOE/SOX9/TNF/DRD2/TGFB1/MYC/HMGCR/KDR/MAPK1/EGFR/NODAL/ERBB2/FGF2/ICAM1/ABL1/SRC/IL1B/HTR2A/JUN/NOTCH1/AVP

GO:0007204	positive regulation of cytosolic calcium ion concentration	23/149	319/18670	9.06E-16	5.97E-14	2.18E-14	NOS1/BCL2/PLA2G6/DRD2/GRIN2B/F2/TRPA1/ESR1/SNCA/TGFB1/IL13/TRPV1/CACNA1C/FASLG/IL2/CACNA1A/GRIN1/FGF2/CAV1/ABL1/HTR2A/BAX/AVP
GO:0050804	modulation of chemical synaptic transmission	26/149	436/18670	1.00E-15	6.49E-14	2.38E-14	SLC6A4/APOE/BCHE/CACNA1B/MAPT/PLA2G6/TNF/DRD2/GRIN2B/PTGS2/SNCA/SHANK3/NRXN1/ACHE/GSK3B/INS/FMR1/MAPK1/EGFR/CACNA1A/GRIN1/ABL1/DRD4/SRC/IL1B/HTR2A
GO:0099177	regulation of trans-synaptic signaling	26/149	437/18670	1.06E-15	6.76E-14	2.47E-14	SLC6A4/APOE/BCHE/CACNA1B/MAPT/PLA2G6/TNF/DRD2/GRIN2B/PTGS2/SNCA/SHANK3/NRXN1/ACHE/GSK3B/INS/FMR1/MAPK1/EGFR/CACNA1A/GRIN1/ABL1/DRD4/SRC/IL1B/HTR2A
GO:0007611	learning or memory	21/149	256/18670	1.35E-15	8.51E-14	3.12E-14	SLC6A4/APOE/BCHE/OPRK1/MAPT/PLA2G6/DRD2/GRIN2B/PTGS2/SHANK3/NRXN1/CASP3/HMGCR/MAPK1/EGFR/GRIN1/FOS/THRA/HTR2A/JUN/GATM
GO:0034614	cellular response to reactive oxygen species	18/149	168/18670	1.51E-15	9.40E-14	3.44E-14	MMP2/MET/MAPT/NFE2L2/TNF/MPO/IL6/IL10/MAPK1/EGFR/NQO1/SOD1/FOS/ABL1/SRC/AKT1/JUN/MMP9
GO:0009410	response to xenobiotic stimulus	22/149	292/18670	1.61E-15	9.88E-14	3.62E-14	BCHE/CYP1A1/HTR3A/OPRK1/PPARG/DRD2/CYP2E1/SNCA/TGFB1/GGT1/EGFR/NQO1/SOD1/GRIN1/ICAM1/CACNA1S/DRD4/RB1/CASP9/PTGS1/HTR2A/CYP3A4
GO:0060401	cytosolic calcium ion transport	18/149	171/18670	2.07E-15	1.25E-13	4.59E-14	NOS1/BCL2/DRD2/GRIN2B/F2/TRPA1/SNCA/TGFB1/IL13/TRPV1/CACNA1C/FASLG/GRIN1/FGF2/CAV1/ABL1/HTR2A/BAX
GO:0048661	positive regulation of smooth muscle cell proliferation	15/149	101/18670	2.74E-15	1.64E-13	5.98E-14	MMP2/IGF1/TNF/PTGS2/IL13/STAT1/IL6/HMGCR/HMOX1/IL10/EGFR/FGF2/AKT1/JUN/MMP9
GO:0034764	positive regulation of transmembrane transport	19/149	204/18670	3.23E-15	1.90E-13	6.97E-14	IGF1/NOS1/HTR3A/OPRK1/ABCB1/NFE2L2/F2/SNCA/SHANK3/IL13/KCNQ1/INS/IFNG/ABL1/DRD4/CFTR/AKT1/PIK3R1/BAX
GO:0051222	positive regulation of protein transport	25/149	418/18670	3.53E-15	2.06E-13	7.52E-14	IGF1/LEP/PLA2G6/TNF/IL1A/DRD2/PTGS2/TGFB1/IL13/ACHE/GSK3B/IL6/INS/IL10/MAPK1/EGFR/IL2/ERBB2/IFNG/ABL1/CFTR/SRC/TP53/PIK3R1/IL1B
GO:0043491	protein kinase B signaling	21/149	269/18670	3.65E-15	2.10E-13	7.68E-14	IGF1/MET/LEP/RET/SOX9/TNF/DRD2/P2RY12/ESR1/TGFB1/HSP90AA1/KDR/INS/IGF2/EGFR/ERBB2/FGF2/SRC/AKT1/PIK3R1/IL1B
GO:2000116	regulation of cysteine-type endopeptidase activity	20/149	239/18670	4.58E-15	2.60E-13	9.51E-14	FAS/CASP8/PPARG/MAPT/TNF/GRIN2B/PTGS2/SNCA/CTSD/MYC/S100A8/FASLG/NODAL/GRIN1/SRC/CASP9/AKT1/BAX/AVP/MMP9
GO:0051047	positive regulation of secretion	25/149	428/18670	6.08E-15	3.41E-13	1.25E-13	IGF1/SLC6A4/LEP/OPRK1/PLA2G6/TNF/IL1A/DRD2/SNCA/TGFB1/S100A8/IL13/ACHE/IL6/TRPV1/INS/IL10/EGFR/IL2/IFNG/ABL1/CFTR/SRC/IL1B/AVP
GO:0009266	response to temperature stimulus	20/149	243/18670	6.31E-15	3.49E-13	1.28E-13	IGF1/NOS1/CASP8/PPARG/MAPT/IL1A/TRPA1/PTGS2/GSK3B/TRPV1/HSP90AA1/HMOX1/MAPK1/SOD1/FOS/THRA/CDKN1A/AKT1/NFKBIA/HTR2A
GO:0097237	cellular response to toxic substance	20/149	247/18670	8.63E-15	4.71E-13	1.72E-13	MET/APOE/DUOX2/NFE2L2/TNF/MPO/PTGS2/IL6/KDR/HMOX1/IL10/NQO1/SOD1/ABL1/TPO/SRC/IYD/CAT/NFKB1/PTGS1
GO:0051480	regulation of cytosolic calcium ion concentration	23/149	357/18670	1.03E-14	5.54E-13	2.03E-13	NOS1/BCL2/PLA2G6/DRD2/GRIN2B/F2/TRPA1/ESR1/SNCA/TGFB1/IL13/TRPV1/CACNA1C/FASLG/IL2/CACNA1A/GRIN1/FGF2/CAV1/ABL1/HTR2A/BAX/AVP

GO:0032355	response to estradiol	16/149	134/18670	1.09E-14	5.79E-13	2.12E-13	SLC6A4/LEP/COL1A1/CASP8/PTGS2/CTNNB1/CCND1/ESR1/TGFB1/CASP3/GGT1/IL10/EGFR/NQO1/CASP9/CAT
GO:0010001	glial cell differentiation	19/149	218/18670	1.10E-14	5.79E-13	2.12E-13	PPARG/MAPT/SOX9/TNF/F2/CTNNB1/TGFB1/S100A8/NKX2-1/IL6/MAPK1/EGFR/SOD1/ERBB2/IFNG/ABL1/AKT1/IL1B/NOTCH1
GO:0001666	response to hypoxia	23/149	359/18670	1.16E-14	6.02E-13	2.20E-13	MMP2/PLAU/NOS2/SLC6A4/LEP/NOS1/DPP4/BCL2/CYP1A1/NFE2L2/DRD2/PTGS2/TGFB1/MYC/CASP3/HMOX1/ICAM1/CAV1/SRC/TP53/CAT/AKT1/NOTCH1
GO:0045785	positive regulation of cell adhesion	24/149	403/18670	1.46E-14	7.45E-13	2.73E-13	IGF1/LEP/DPP4/RET/TNF/P2RY12/TGFB1/GSK3B/IL6/KDR/IL10/IGF2/NODAL/IL2/ERBB2/IFNG/ICAM1/CAV1/ABL1/SRC/JAK3/AKT1/PIK3R1/IL1B
GO:0018108	peptidyl-tyrosine phosphorylation	23/149	363/18670	1.46E-14	7.45E-13	2.73E-13	IGF1/MET/LEP/ALK/ADRA2A/RET/TNF/TGFB1/IL13/IL6/KDR/IGF2/EGFR/IL2/ERBB2/IFNG/ICAM1/CAV1/ABL1/SRC/TP53/JAK3/HTR2A
GO:0097193	intrinsic apoptotic signaling pathway	21/149	289/18670	1.53E-14	7.68E-13	2.81E-13	BCL2/NFE2L2/TNF/PTGS2/S100A8/CASP3/PARP1/HMOX1/INS/SOD1/CAV1/ABL1/SRC/TP53/CASP9/CDKN1A/AKT1/PIK3R1/SNAI2/BAX/MMP9
GO:0009314	response to radiation	25/149	448/18670	1.73E-14	8.59E-13	3.14E-13	BCL2/OPRK1/DRD2/PTGS2/CCND1/TGFB1/MYC/CASP3/PARP1/HMGCR/FMR1/EGFR/GRIN1/FOS/ICAM1/FECH/TP53/CASP9/CDKN1A/CAT/AKT1/PIK3R1/JUN/SNAI2/BAX
GO:0018212	peptidyl-tyrosine modification	23/149	366/18670	1.75E-14	8.59E-13	3.14E-13	IGF1/MET/LEP/ALK/ADRA2A/RET/TNF/TGFB1/IL13/IL6/KDR/IGF2/EGFR/IL2/ERBB2/IFNG/ICAM1/CAV1/ABL1/SRC/TP53/JAK3/HTR2A
GO:0072503	cellular divalent inorganic cation homeostasis	26/149	493/18670	1.84E-14	8.98E-13	3.28E-13	NOS1/APOE/BCL2/PLA2G6/DRD2/GRIN2B/F2/TRPA1/ESR1/SNCA/TGFB1/S100A8/IL13/TRPV1/CACNA1C/FASLG/IL2/CACNA1A/GRIN1/FGF2/CAV1/ABL1/DRD4/HTR2A/BAX/AVP
GO:0097553	calcium ion transmembrane import into cytosol	16/149	140/18670	2.19E-14	1.05E-12	3.84E-13	NOS1/DRD2/GRIN2B/F2/TRPA1/SNCA/TGFB1/IL13/TRPV1/CACNA1C/FASLG/GRIN1/FGF2/ABL1/HTR2A/BAX
GO:0036293	response to decreased oxygen levels	23/149	370/18670	2.20E-14	1.05E-12	3.84E-13	MMP2/PLAU/NOS2/SLC6A4/LEP/NOS1/DPP4/BCL2/CYP1A1/NFE2L2/DRD2/PTGS2/TGFB1/MYC/CASP3/HMOX1/ICAM1/CAV1/SRC/TP53/CAT/AKT1/NOTCH1
GO:0150076	neuroinflammatory response	13/149	75/18670	2.59E-14	1.22E-12	4.46E-13	IGF1/MAPT/TNF/PTGS2/SNCA/IL13/IL6/TRPV1/EGFR/IFNG/IL1B/JUN/MMP9
GO:0051098	regulation of binding	23/149	373/18670	2.61E-14	1.22E-12	4.46E-13	IGF1/MET/APOE/PON1/BCL2/PPARG/CTNNB1/TGFB1/GSK3B/PARP1/HMOX1/FMR1/IL10/IFNG/CAV1/ABL1/RB1/SRC/AKT1/NFKBIA/JUN/BAX/MMP9
GO:0015850	organic hydroxy compound transport	20/149	262/18670	2.66E-14	1.23E-12	4.49E-13	SLC6A2/SLC6A4/LEP/NOS1/APOE/PON1/ADRA2A/OPRK1/PPARG/DRD2/P2RY12/SNCA/HTR1A/MAOB/CAV1/DRD4/CFTR/NFKB1/NFKBIA/HTR2A
GO:0006874	cellular calcium ion homeostasis	25/149	458/18670	2.85E-14	1.30E-12	4.77E-13	NOS1/APOE/BCL2/PLA2G6/DRD2/GRIN2B/F2/TRPA1/ESR1/SNCA/TGFB1/IL13/TRPV1/CACNA1C/FASLG/IL2/CACNA1A/GRIN1/FGF2/CAV1/ABL1/DRD4/HTR2A/BAX/AVP
GO:0042310	vasoconstriction	13/149	76/18670	3.11E-14	1.41E-12	5.14E-13	SLC6A4/LEP/ADRA2A/PTGS2/AVPR2/HTR1A/EGFR/ICAM1/CAV1/AKT1/CHRM3/HTR2A/AVP
GO:0023061	signal release	25/149	462/18670	3.47E-14	1.56E-12	5.69E-13	NOS2/LEP/DPP4/ADRA2A/OPRK1/CACNA1B/PLA2G6/TNF/DRD2/SNCA/NRXN1/GSK3B/IL6/HMGCR/CACNA1C/INS/HTR1A/FMR1/EGFR/SLC25A4/CACNA1A/IFNG/CFTR/IL1B/HTR2A

GO:0006809	nitric oxide biosynthetic process	13/149	77/18670	3.71E-14	1.65E-12	6.03E-13	NOS2/NOS1/TNF/PTGS2/TRPV1/HSP90AA1/IL10/NQO1/IFNG/ICAM1/CAV1/AKT1/IL1B
GO:0001819	positive regulation of cytokine production	25/149	464/18670	3.83E-14	1.68E-12	6.16E-13	IRF1/LEP/ADRA2A/CASP8/TNF/IL1A/DRD2/PTGS2/CTNNB1/TGFB1/IL13/STAT1/IL6/HMOX1/INS/IL10/NODAL/SOD1/IL2/IFNG/ABL1/SRC/PIK3R1/NFKB1/IL1B
GO:1904062	regulation of cation transmembrane transport	22/149	342/18670	4.23E-14	1.81E-12	6.61E-13	NOS1/ADRA2A/OPRK1/PLA2G6/DRD2/GRIN2B/F2/SNCA/TGFB1/SHANK3/IL13/KCNQ1/CACNA1C/FMR1/GRIN1/IFNG/CAV1/ABL1/DRD4/BAX/SCN5A/MMP9
GO:0042542	response to hydrogen peroxide	16/149	146/18670	4.27E-14	1.81E-12	6.61E-13	MET/COL1A1/BCL2/NFE2L2/TRPA1/STAT1/IL6/CASP3/HMOX1/IL10/NQO1/SOD1/ABL1/SRC/CAT/JUN
GO:0045834	positive regulation of lipid metabolic process	16/149	146/18670	4.27E-14	1.81E-12	6.61E-13	APOE/PPARG/PLA2G6/TNF/F2/PTGS2/P2RY12/TGFB1/INS/IFNG/FGF2/SRC/AKT1/IL1B/HTR2A/AVP
GO:0051384	response to glucocorticoid	16/149	146/18670	4.27E-14	1.81E-12	6.61E-13	BCHE/BCL2/TNF/PTGS2/CCND1/TGFB1/IL6/CASP3/MAOB/IL10/EGFR/FOS/ICAM1/CASP9/CDKN1A/SPARC
GO:0001101	response to acid chemical	22/149	343/18670	4.49E-14	1.88E-12	6.88E-13	MMP2/SLC6A4/LEP/COL1A1/PON1/BCHE/PPARG/RET/SOX9/TNF/PTGS2/CYP2E1/CASP3/KDR/EGFR/NQO1/GRIN1/ICAM1/SRC/CAT/AKT1/SPARC
GO:0055074	calcium ion homeostasis	25/149	471/18670	5.38E-14	2.23E-12	8.16E-13	NOS1/APOE/BCL2/PLA2G6/DRD2/GRIN2B/F2/TRPA1/ESR1/SNCA/TGFB1/IL13/TRPV1/CACNA1C/FASLG/IL2/CACNA1A/GRIN1/FGF2/CAV1/ABL1/DRD4/HTR2A/BAX/AVP
GO:0014074	response to purine-containing compound	16/149	149/18670	5.90E-14	2.43E-12	8.88E-13	SLC6A4/COL1A1/DUOX2/PPARG/PTGS2/P2RY12/STAT1/KCNQ1/TRPV1/SOD1/FOS/CACNA1S/CFTR/IL1B/SPARC/JUN
GO:0051090	regulation of DNA-binding transcription factor activity	24/149	432/18670	6.73E-14	2.74E-12	1.00E-12	ALK/PPARG/TNF/CTNNB1/ESR1/TGFB1/S100A8/IL6/HMOX1/INS/IL10/MAPK1/NODAL/FOS/ICAM1/CAV1/RB1/TCF3/CAT/AKT1/NFKB1/IL1B/NFKBIA/JUN
GO:0050863	regulation of T cell activation	21/149	314/18670	7.85E-14	3.17E-12	1.16E-12	IGF1/IRF1/LEP/DPP4/CTNNB1/TGFB1/IL6/CASP3/IL10/IGF2/SOD1/IL2/ERBB2/IFNG/CAV1/ABL1/SRC/JAK3/AKT1/PIK3R1/IL1B
GO:0043434	response to peptide hormone	24/149	436/18670	8.22E-14	3.29E-12	1.20E-12	LEP/COL1A1/OPRK1/PPARG/NFE2L2/PTGS2/TGFB1/GSK3B/STAT1/TRPV1/PARP1/INS/IL10/IGF2/ICAM1/CAV1/SRC/CAT/JAK3/AKT1/PIK3R1/NFKB1/IL1B/SPARC
GO:0046209	nitric oxide metabolic process	13/149	82/18670	8.71E-14	3.45E-12	1.26E-12	NOS2/NOS1/TNF/PTGS2/TRPV1/HSP90AA1/IL10/NQO1/IFNG/ICAM1/CAV1/AKT1/IL1B
GO:0062013	positive regulation of small molecule metabolic process	16/149	153/18670	8.98E-14	3.53E-12	1.29E-12	IGF1/NOS2/NOS1/PPARG/TNF/PTGS2/SNCA/INS/IGF2/IFNG/SRC/AKT1/NFKB1/IL1B/HTR2A/AVP
GO:0032768	regulation of monooxygenase activity	12/149	64/18670	9.80E-14	3.82E-12	1.40E-12	LEP/APOE/TNF/SNCA/HSP90AA1/INS/EGFR/IFNG/CAV1/AKT1/NFKB1/IL1B
GO:0001503	ossification	23/149	398/18670	1.03E-13	3.98E-12	1.45E-12	MMP2/IGF1/LEP/COL1A1/BCL2/SOX9/TNF/PTGS2/CTNNB1/TGFB1/ACHE/IL6/MAPK1/IGF2/EGFR/THRA/CAT/AKT1/SPARC/IGFBP3/SNAI2/NOTCH1/CYP24A1
GO:0050727	regulation of inflammatory response	25/149	485/18670	1.04E-13	3.98E-12	1.46E-12	IGF1/NOS2/LEP/PLA2G2A/APOE/PPARG/TNF/F2/PTGS2/ESR1/SNCA/S100A8/IL6/GGT1/INS/IL10/EGFR/SOD1/IL2/RB1/NFKB1/IL1B/F12/NFKBIA/MMP9

GO:1903532	positive regulation of secretion by cell	23/149	399/18670	1.09E-13	4.12E-12	1.51E-12	IGF1/SLC6A4/LEP/OPRK1/PLA2G6/TNF/IL1A/DRD2/SNCA/TGFB1/IL13/ACHE/IL6/INS/IL10/EGFR/IL2/IFNG/ABL1/CFTR/SRC/IL1B/AVP
GO:0033138	positive regulation of peptidyl-serine phosphorylation	14/149	105/18670	1.13E-13	4.24E-12	1.55E-12	NOS1/BCL2/RET/TNF/PTGS2/SNCA/TGFB1/IL6/HSP90AA1/EGFR/IFNG/CAV1/AKT1/AVP
GO:0032409	regulation of transporter activity	20/149	283/18670	1.14E-13	4.27E-12	1.56E-12	NOS1/PON1/BCL2/ADRA2A/HTR3A/PPARG/ABCB1/PLA2G6/DRD2/GRIN2B/SNCA/SHANK3/INS/FMR1/GRIN1/IFNG/CAV1/DRD4/CFTR/MMP9
GO:0043281	regulation of cysteine-type endopeptidase activity involved in apoptotic process	18/149	215/18670	1.16E-13	4.28E-12	1.56E-12	FAS/CASP8/PPARG/MAPT/TNF/PTGS2/SNCA/CTSD/MYC/S100A8/FASLG/NODAL/SRC/CASP9/AKT1/BAX/AVP/MMP9
GO:0030857	negative regulation of epithelial cell differentiation	11/149	49/18670	1.29E-13	4.75E-12	1.74E-12	SOX9/CTNNB1/CCND1/IL13/GSK3B/STAT1/NODAL/IFNG/CAV1/NOTCH1/MMP9
GO:0022407	regulation of cell-cell adhesion	23/149	403/18670	1.34E-13	4.87E-12	1.78E-12	IGF1/IRF1/LEP/DPP4/TNF/TGFB1/IL6/CASP3/IL10/IGF2/NODAL/IL2/ERBB2/IFNG/ICAM1/CAV1/ABL1/SRC/JAK3/AKT1/PIK3R1/IL1B/SNAI2
GO:0043271	negative regulation of ion transport	16/149	157/18670	1.35E-13	4.87E-12	1.78E-12	LEP/NOS1/BCL2/ADRA2A/DRD2/PTGS2/SNCA/TGFB1/FMR1/MAOB/ICAM1/CAV1/DRD4/AKT1/HTR2A/MMP9
GO:2001057	reactive nitrogen species metabolic process	13/149	85/18670	1.41E-13	5.07E-12	1.85E-12	NOS2/NOS1/TNF/PTGS2/TRPV1/HSP90AA1/IL10/NQO1/IFNG/ICAM1/CAV1/AKT1/IL1B
GO:0051341	regulation of oxidoreductase activity	14/149	107/18670	1.47E-13	5.24E-12	1.92E-12	LEP/APOE/TNF/SNCA/IL13/HSP90AA1/INS/EGFR/IFNG/CAV1/ABL1/AKT1/NFKB1/IL1B
GO:0042063	gliogenesis	20/149	290/18670	1.81E-13	6.38E-12	2.34E-12	PPARG/MAPT/SOX9/TNF/F2/CTNNB1/P2RY12/TGFB1/S100A8/NKX2-1/IL6/MAPK1/EGFR/SOD1/ERBB2/IFNG/ABL1/AKT1/IL1B/NOTCH1
GO:0044706	multi-multicellular organism process	18/149	222/18670	2.01E-13	7.03E-12	2.57E-12	MMP2/SLC6A4/LEP/BCL2/CYP1A1/PLA2G6/PTGS2/ESR1/TGFB1/MAPK1/NODAL/SOD1/FOS/PGR/ACVR1B/AKT1/IL1B/MMP9
GO:0007159	leukocyte cell-cell adhesion	21/149	337/18670	3.12E-13	1.08E-11	3.96E-12	IGF1/IRF1/LEP/DPP4/TNF/TGFB1/S100A8/IL6/CASP3/IL10/IGF2/IL2/ERBB2/IFNG/ICAM1/CAV1/SRC/JAK3/AKT1/PIK3R1/IL1B
GO:0001763	morphogenesis of a branching structure	17/149	196/18670	3.24E-13	1.12E-11	4.08E-12	MET/BCL2/SOX9/TNF/DRD2/CTNNB1/ESR1/TGFB1/MYC/NKX2-1/IL10/FGF2/ABL1/SRC/PGR/SNAI2/NOTCH1
GO:0003012	muscle system process	24/149	465/18670	3.32E-13	1.14E-11	4.15E-12	IGF1/LEP/NOS1/ADRA2A/PLA2G6/DRD2/PTGS2/KCNQ1/TRPV1/PARP1/CACNA1C/HMOX1/MYLK/SOD1/CAV1/SLC6A8/CACNA1S/IL1B/CHRM3/HTR2A/SCN10A/GATM/NOTCH1/SCN5A
GO:0033135	regulation of peptidyl-serine phosphorylation	15/149	139/18670	3.47E-13	1.18E-11	4.31E-12	NOS1/BCL2/RET/TNF/PTGS2/SNCA/TGFB1/IL6/HSP90AA1/EGFR/IFNG/CAV1/AKT1/BAX/AVP
GO:0050806	positive regulation of synaptic transmission	16/149	168/18670	3.91E-13	1.32E-11	4.81E-12	APOE/TNF/DRD2/GRIN2B/PTGS2/SNCA/SHANK3/NRXN1/GSK3B/INS/FMR1/MAPK1/EGFR/GRIN1/ABL1/DRD4

GO:0009411	response to UV	15/149	141/18670	4.30E-13	1.42E-11	5.19E-12	BCL2/PTGS2/CCND1/MYC/CASP3/PARP1/FMR1/EGFR/TP53/CASP9/CDKN1A/CAT/AKT1/PIK3R1/BAX
GO:0038034	signal transduction in absence of ligand	12/149	72/18670	4.34E-13	1.42E-11	5.19E-12	BCL2/RET/TNF/IL1A/GSK3B/CASP3/IL2/CASP9/AKT1/IL1B/SNAI2/BAX
GO:0050805	negative regulation of synaptic transmission	12/149	72/18670	4.34E-13	1.42E-11	5.19E-12	SLC6A4/BCHE/MAPT/PLA2G6/DRD2/PTGS2/SNCA/SHANK3/ACHE/FMR1/IL1B/HTR2A
GO:0097192	extrinsic apoptotic signaling pathway in absence of ligand	12/149	72/18670	4.34E-13	1.42E-11	5.19E-12	BCL2/RET/TNF/IL1A/GSK3B/CASP3/IL2/CASP9/AKT1/IL1B/SNAI2/BAX
GO:1903037	regulation of leukocyte cell-cell adhesion	20/149	304/18670	4.37E-13	1.42E-11	5.19E-12	IGF1/IRF1/LEP/DPP4/TNF/TGFB1/IL6/CASP3/IL10/IGF2/IL2/ERBB2/IFNG/ICAM1/CAV1/SRC/JAK3/AKT1/PIK3R1/IL1B
GO:0031668	cellular response to extracellular stimulus	19/149	268/18670	4.64E-13	1.50E-11	5.47E-12	LEP/COL1A1/FAS/BCL2/PPARG/NFE2L2/PTGS2/P2RY12/TRPV1/HMOX1/MAPK1/SOD1/FOS/ICAM1/TP53/CDKN1A/JUN/SNAI2/CYP24A1
GO:0033273	response to vitamin	13/149	93/18670	4.70E-13	1.51E-11	5.51E-12	LEP/COL1A1/BCHE/CYP1A1/PPARG/PTGS2/CCND1/TGFB1/EGFR/CAT/SPARC/SNAI2/CYP24A1
GO:0048608	reproductive structure development	23/149	431/18670	5.44E-13	1.73E-11	6.33E-12	LEP/BCL2/CASP8/PPARG/SOX9/PTGS2/CTNNB1/CCND1/ESR1/NKX2-1/CASP3/IL10/MAPK1/IGF2/EGFR/NODAL/SOD1/ICAM1/SRC/PGR/AKT1/NOTCH1/BAX
GO:0046651	lymphocyte proliferation	19/149	272/18670	6.05E-13	1.91E-11	6.98E-12	IGF1/IRF1/LEP/BCL2/CTNNB1/TGFB1/IL13/IL6/CASP3/IL10/IGF2/IL2/ERBB2/ABL1/TP53/CDKN1A/JAK3/IL1B/BAX
GO:0031669	cellular response to nutrient levels	18/149	237/18670	6.17E-13	1.92E-11	7.02E-12	LEP/COL1A1/FAS/BCL2/PPARG/NFE2L2/PTGS2/P2RY12/TRPV1/HMOX1/MAPK1/SOD1/ICAM1/TP53/CDKN1A/JUN/SNAI2/CYP24A1
GO:0001659	temperature homeostasis	16/149	173/18670	6.17E-13	1.92E-11	7.02E-12	LEP/PRDM16/TNF/IL1A/DRD2/PTGS2/IL13/ACHE/TRPV1/CAV1/RB1/THRA/IL1B/HTR2A/GATM/NOTCH1
GO:0061458	reproductive system development	23/149	434/18670	6.28E-13	1.94E-11	7.10E-12	LEP/BCL2/CASP8/PPARG/SOX9/PTGS2/CTNNB1/CCND1/ESR1/NKX2-1/CASP3/IL10/MAPK1/IGF2/EGFR/NODAL/SOD1/ICAM1/SRC/PGR/AKT1/NOTCH1/BAX
GO:0051048	negative regulation of secretion	18/149	238/18670	6.63E-13	2.03E-11	7.44E-12	LEP/APOE/ADRA2A/OPRK1/TNF/DRD2/P2RY12/SNCA/AVPR2/HMGCR/HMOX1/INS/FMR1/MAOB/IL10/DRD4/IL1B/NOTCH1
GO:0032943	mononuclear cell proliferation	19/149	274/18670	6.89E-13	2.10E-11	7.68E-12	IGF1/IRF1/LEP/BCL2/CTNNB1/TGFB1/IL13/IL6/CASP3/IL10/IGF2/IL2/ERBB2/ABL1/TP53/CDKN1A/JAK3/IL1B/BAX
GO:0051402	neuron apoptotic process	18/149	239/18670	7.12E-13	2.16E-11	7.89E-12	APOE/BCL2/THRB/TNF/CTNNB1/SNCA/CASP3/PARP1/FASLG/HMOX1/NQO1/SOD1/GRIN1/RB1/TP53/CASP9/JUN/BAX
GO:0050679	positive regulation of epithelial cell proliferation	17/149	206/18670	7.31E-13	2.20E-11	8.04E-12	IGF1/SOX9/CTNNB1/CCND1/TGFB1/MYC/KDR/HMOX1/IL10/EGFR/NODAL/ERBB2/FGF2/AKT1/JUN/NOTCH1/SCN5A
GO:0016049	cell growth	24/149	484/18670	7.85E-13	2.34E-11	8.58E-12	IGF1/APOE/BCL2/PPARG/MAPT/SOX9/F2/CTNNB1/TGFB1/S100A8/GSK3B/HSP90AA1/INS/EGFR/IL2/ERBB2/ABL1/RB1/ACVR1B/TP53/CDKN1A/AKT1/IGFBP3/AVP

GO:0009416	response to light stimulus	20/149	314/18670	7.97E-13	2.36E-11	8.65E-12	BCL2/DRD2/PTGS2/CCND1/MYC/CASP3/PARP1/HMGCR/FMR1/EGFR/GRIN1/FOS/FECH/TP53/CASP9/CDKN1A/CAT/AKT1/PIK3R1/BAX
GO:0051249	regulation of lymphocyte activation	24/149	485/18670	8.20E-13	2.42E-11	8.85E-12	IGF1/IRF1/LEP/DPP4/BCL2/CTNNB1/TGFB1/IL13/IL6/CASP3/IL10/IGF2/SOD1/IL2/ERBB2/IFNG/CAV1/ABL1/SRC/CDKN1A/JAK3/AKT1/PIK3R1/IL1B
GO:0014065	phosphatidylinositol 3-kinase signaling	15/149	148/18670	8.80E-13	2.58E-11	9.43E-12	IGF1/LEP/SOX9/TNF/F2/KDR/INS/MAPK1/EGFR/ERBB2/SRC/CAT/AKT1/PIK3R1/HTR2A
GO:0019229	regulation of vasoconstriction	11/149	58/18670	9.51E-13	2.76E-11	1.01E-11	LEP/ADRA2A/PTGS2/AVPR2/EGFR/ICAM1/CAV1/AKT1/CHRM3/HTR2A/AVP
GO:2001056	positive regulation of cysteine-type endopeptidase activity	15/149	149/18670	9.72E-13	2.81E-11	1.03E-11	FAS/CASP8/PPARG/MAPT/TNF/GRIN2B/SNCA/CTSD/MYC/S100A8/FASLG/NODAL/GRIN1/CASP9/BAX
GO:1903531	negative regulation of secretion by cell	17/149	211/18670	1.08E-12	3.10E-11	1.14E-11	LEP/APOE/ADRA2A/OPRK1/TNF/DRD2/P2RY12/SNCA/HMGCR/HMOX1/INS/FMR1/MAOB/IL10/DRD4/IL1B/NOTCH1
GO:0051209	release of sequestered calcium ion into cytosol	14/149	124/18670	1.17E-12	3.35E-11	1.23E-11	NOS1/DRD2/F2/TRPA1/SNCA/TGFB1/IL13/TRPV1/CACNA1C/FASLG/FGF2/ABL1/HTR2A/BAX
GO:0061138	morphogenesis of a branching epithelium	16/149	182/18670	1.36E-12	3.84E-11	1.41E-11	MET/BCL2/SOX9/TNF/CTNNB1/ESR1/TGFB1/MYC/NKX2-1/IL10/FGF2/ABL1/SRC/PGR/SNAI2/NOTCH1
GO:0071260	cellular response to mechanical stimulus	12/149	79/18670	1.38E-12	3.89E-11	1.42E-11	IRF1/COL1A1/FAS/CASP8/SOX9/PTGS2/TGFB1/IL13/EGFR/AKT1/NFKB1/IL1B
GO:1903426	regulation of reactive oxygen species biosynthetic process	13/149	101/18670	1.40E-12	3.92E-11	1.43E-11	TNF/PTGS2/SNCA/TRPV1/HSP90AA1/INS/IL10/GRIN1/IFNG/ICAM1/CAV1/AKT1/IL1B
GO:1903829	positive regulation of cellular protein localization	20/149	324/18670	1.42E-12	3.96E-11	1.45E-11	LEP/BCL2/CASP8/MAPT/TNF/F2/PTGS2/TGFB1/GSK3B/PARP1/INS/MAPK1/EGFR/ERBB2/IFNG/SRC/TP53/AKT1/PIK3R1/IL1B
GO:0032102	negative regulation of response to external stimulus	21/149	365/18670	1.45E-12	4.02E-11	1.47E-11	IGF1/PLAU/LEP/APOE/PPARG/DRD2/F2/NRXN1/HMGCR/INS/IL10/SOD1/IL2/GRIN1/FGF2/RB1/CDKN1A/NFKB1/F12/SNAI2/NOTCH1
GO:0051283	negative regulation of sequestering of calcium ion	14/149	126/18670	1.47E-12	4.03E-11	1.47E-11	NOS1/DRD2/F2/TRPA1/SNCA/TGFB1/IL13/TRPV1/CACNA1C/FASLG/FGF2/ABL1/HTR2A/BAX
GO:0042098	T cell proliferation	16/149	184/18670	1.61E-12	4.38E-11	1.60E-11	IGF1/IRF1/LEP/CTNNB1/TGFB1/IL6/CASP3/IL10/IGF2/IL2/ERBB2/ABL1/TP53/JAK3/IL1B/BAX
GO:0002793	positive regulation of peptide secretion	19/149	288/18670	1.67E-12	4.53E-11	1.66E-11	IGF1/PLA2G6/TNF/IL1A/DRD2/TGFB1/S100A8/IL13/ACHE/IL6/INS/IL10/EGFR/IL2/IFNG/ABL1/CFTR/SRC/IL1B

GO:2001236	regulation of extrinsic apoptotic signaling pathway	15/149	155/18670	1.74E-12	4.69E-11	1.71E-11	IGF1/FAS/BCL2/CASP8/RET/TNF/IL1A/FASLG/HMOX1/ICAM1/CAV1/SRC/AKT1/IL1B/SNAI2
GO:0051282	regulation of sequestering of calcium ion	14/149	128/18670	1.83E-12	4.90E-11	1.79E-11	NOS1/DRD2/F2/TRPA1/SNCA/TGFB1/IL13/TRPV1/CACNA1C/FASLG/FGF2/ABL1/HTR2A/BAX
GO:0048708	astrocyte differentiation	12/149	81/18670	1.88E-12	5.01E-11	1.83E-11	MAPT/SOX9/TNF/F2/S100A8/IL6/MAPK1/EGFR/IFNG/ABL1/IL1B/NOTCH1
GO:2001237	negative regulation of extrinsic apoptotic signaling pathway	13/149	104/18670	2.06E-12	5.45E-11	2.00E-11	IGF1/FAS/BCL2/CASP8/TNF/IL1A/FASLG/HMOX1/ICAM1/SRC/AKT1/IL1B/SNAI2
GO:0022409	positive regulation of cell-cell adhesion	18/149	255/18670	2.14E-12	5.64E-11	2.06E-11	IGF1/LEP/DPP4/TNF/TGFB1/IL6/IL10/IGF2/NODAL/IL2/IFNG/ICAM1/CAV1/SRC/JAK3/AKT1/PIK3R1/IL1B
GO:0001667	ameboidal-type cell migration	23/149	461/18670	2.18E-12	5.72E-11	2.09E-11	MET/DPP4/APOE/PPARG/RET/NFE2L2/SOX9/PTGS2/P2RY12/TGFB1/KDR/HMOX1/NODAL/IFNG/FGF2/ABL1/SRC/ACVR1B/AKT1/SPARC/JUN/NOTCH1/MMP9
GO:0045907	positive regulation of vasoconstriction	9/149	32/18670	2.47E-12	6.43E-11	2.35E-11	PTGS2/AVPR2/EGFR/ICAM1/CAV1/AKT1/CHRM3/HTR2A/AVP
GO:0071902	positive regulation of protein serine/threonine kinase activity	20/149	334/18670	2.49E-12	6.45E-11	2.36E-11	IGF1/ALK/ADRA2A/RET/TNF/CCND1/SNCA/TGFB1/MAPK1/IGF2/EGFR/SOD1/ERBB2/IFNG/FGF2/DRD4/SRC/AKT1/IL1B/HTR2A
GO:0051208	sequestering of calcium ion	14/149	131/18670	2.52E-12	6.48E-11	2.37E-11	NOS1/DRD2/F2/TRPA1/SNCA/TGFB1/IL13/TRPV1/CACNA1C/FASLG/FGF2/ABL1/HTR2A/BAX
GO:0048145	regulation of fibroblast proliferation	12/149	83/18670	2.54E-12	6.50E-11	2.38E-11	IGF1/PPARG/CTNNB1/ESR1/TGFB1/MYC/IL13/EGFR/TP53/CDKN1A/JUN/BAX
GO:0043254	regulation of protein complex assembly	23/149	467/18670	2.85E-12	7.24E-11	2.65E-11	MET/APOE/MAPT/TNF/CTNNB1/P2RY12/ESR1/SNCA/TGFB1/GSK3B/MMP1/HSP90AA1/PARP1/INS/IFNG/ICAM1/ABL1/RB1/SRC/THRA/TP53/JUN/BAX
GO:0048144	fibroblast proliferation	12/149	84/18670	2.94E-12	7.45E-11	2.73E-11	IGF1/PPARG/CTNNB1/ESR1/TGFB1/MYC/IL13/EGFR/TP53/CDKN1A/JUN/BAX
GO:0070661	leukocyte proliferation	19/149	298/18670	3.05E-12	7.67E-11	2.81E-11	IGF1/IRF1/LEP/BCL2/CTNNB1/TGFB1/IL13/IL6/CASP3/IL10/IGF2/IL2/ERBB2/ABL1/TP53/CDKN1A/JAK3/IL1B/BAX
GO:0007565	female pregnancy	16/149	192/18670	3.09E-12	7.74E-11	2.83E-11	MMP2/LEP/BCL2/PLA2G6/PTGS2/ESR1/TGFB1/MAPK1/NODAL/SOD1/FOS/PGR/ACVR1B/AKT1/IL1B/MMP9
GO:0051091	positive regulation of DNA-binding transcription factor activity	18/149	261/18670	3.17E-12	7.89E-11	2.89E-11	ALK/PPARG/TNF/CTNNB1/ESR1/TGFB1/S100A8/IL6/INS/IL10/NODAL/ICAM1/CAV1/TCF3/CAT/AKT1/NFKB1/IL1B

GO:0046683	response to organophosphorus	14/149	134/18670	3.45E-12	8.53E-11	3.12E-11	SLC6A4/COL1A1/DUOX2/PTGS2/P2RY12/STAT1/KCNQ1/TRPV1/SOD1/FOS/CFTR/IL1B/SPARC/JUN
GO:0042359	vitamin D metabolic process	8/149	22/18670	3.96E-12	9.75E-11	3.57E-11	CYP1A1/TNF/IFNG/NFKB1/IL1B/SNAI2/CYP24A1/CYP3A4
GO:0046883	regulation of hormone secretion	18/149	266/18670	4.37E-12	1.07E-10	3.91E-11	NOS2/LEP/DPP4/ADRA2A/OPRK1/PLA2G6/TNF/DRD2/HMGCR/CACNA1C/INS/HTR1A/EGFR/SLC25A4/CACNA1A/IFNG/CFTR/IL1B
GO:0010952	positive regulation of peptidase activity	16/149	197/18670	4.59E-12	1.12E-10	4.09E-11	FAS/CASP8/PPARG/MAPT/TNF/GRIN2B/SNCA/CTSD/MYC/S100A8/FASLG/NODAL/GRIN1/CAV1/CASP9/BAX
GO:0022898	regulation of transmembrane transporter activity	18/149	268/18670	4.95E-12	1.19E-10	4.36E-11	NOS1/BCL2/ADRA2A/HTR3A/ABCB1/PLA2G6/DRD2/GRIN2B/SNCA/SHANK3/INS/FMR1/GRIN1/IFNG/CAV1/DRD4/CFTR/MMP9
GO:0050714	positive regulation of protein secretion	18/149	268/18670	4.95E-12	1.19E-10	4.36E-11	IGF1/PLA2G6/TNF/IL1A/DRD2/TGFB1/IL13/ACHE/IL6/INS/IL10/EGFR/IL2/IFNG/ABL1/CFTR/SRC/IL1B
GO:0043279	response to alkaloid	13/149	112/18670	5.41E-12	1.30E-10	4.74E-11	BCHE/HTR3A/OPRK1/PPARG/DRD2/SNCA/TRPV1/CASP3/GRIN1/ICAM1/CACNA1S/DRD4/HTR2A
GO:0071216	cellular response to biotic stimulus	17/149	236/18670	6.58E-12	1.57E-10	5.74E-11	NOS2/OPRK1/CXCL8/TNF/TGFB1/GSK3B/IL6/IL10/MAPK1/ICAM1/ABL1/SRC/TP53/AKT1/NFKB1/IL1B/NFKBIA
GO:0046879	hormone secretion	19/149	312/18670	6.82E-12	1.61E-10	5.91E-11	NOS2/LEP/DPP4/ADRA2A/OPRK1/PLA2G6/TNF/DRD2/IL6/HMGCR/CACNA1C/INS/HTR1A/EGFR/SLC25A4/CACNA1A/IFNG/CFTR/IL1B
GO:0015844	monoamine transport	12/149	90/18670	6.85E-12	1.61E-10	5.91E-11	SLC6A2/SLC6A4/NOS1/ADRA2A/OPRK1/DRD2/P2RY12/SNCA/HTR1A/MAOB/DRD4/HTR2A
GO:0010634	positive regulation of epithelial cell migration	15/149	171/18670	7.28E-12	1.71E-10	6.24E-11	MET/NFE2L2/SOX9/PTGS2/TGFB1/KDR/HMOX1/IFNG/FGF2/ABL1/SRC/AKT1/SPARC/JUN/MMP9
GO:0051899	membrane depolarization	12/149	91/18670	7.84E-12	1.83E-10	6.69E-11	BCL2/CACNA1B/PARP1/CACNA1C/KDR/CACNA1A/CAV1/ABL1/SRC/SCN10A/JUN/SCN5A
GO:0050670	regulation of lymphocyte proliferation	16/149	208/18670	1.05E-11	2.43E-10	8.88E-11	IGF1/IRF1/LEP/BCL2/CTNNB1/TGFB1/IL13/IL6/CASP3/IL10/IGF2/IL2/ERBB2/CDKN1A/JAK3/IL1B
GO:0090276	regulation of peptide hormone secretion	16/149	208/18670	1.05E-11	2.43E-10	8.88E-11	NOS2/LEP/DPP4/ADRA2A/PLA2G6/TNF/DRD2/HMGCR/CACNA1C/INS/EGFR/SLC25A4/CACNA1A/IFNG/CFTR/IL1B
GO:0051897	positive regulation of protein kinase B signaling	15/149	176/18670	1.10E-11	2.54E-10	9.28E-11	MET/LEP/RET/TNF/P2RY12/ESR1/TGFB1/HSP90AA1/INS/IGF2/EGFR/ERBB2/FGF2/SRC/PIK3R1
GO:0052547	regulation of peptidase activity	22/149	452/18670	1.12E-11	2.55E-10	9.33E-11	FAS/CASP8/PPARG/MAPT/TNF/GRIN2B/PTGS2/SNCA/CTSD/MYC/S100A8/FASLG/SERPINH1/NODAL/GRIN1/CAV1/SRC/CASP9/AKT1/BAX/AVP/MMP9
GO:0051896	regulation of protein kinase B signaling	17/149	244/18670	1.12E-11	2.55E-10	9.33E-11	MET/LEP/RET/TNF/DRD2/P2RY12/ESR1/TGFB1/HSP90AA1/INS/IGF2/EGFR/ERBB2/FGF2/SRC/AKT1/PIK3R1

GO:0032944	regulation of mononuclear cell proliferation	16/149	209/18670	1.13E-11	2.56E-10	9.35E-11	IGF1/IRF1/LEP/BCL2/CTNNB1/TGFB1/IL13/IL6/CASP3/IL10/IGF2/IL2/ERBB2/CDKN1A/JAK3/IL1B
GO:0009914	hormone transport	19/149	322/18670	1.18E-11	2.65E-10	9.69E-11	NOS2/LEP/DPP4/ADRA2A/OPRK1/PLA2G6/TNF/DRD2/IL6/HMGCR/CACNA1C/INS/HTR1A/EGFR/SLC25A4/CACNA1A/IFNG/CFTR/IL1B
GO:0018209	peptidyl-serine modification	19/149	322/18670	1.18E-11	2.65E-10	9.69E-11	NOS1/BCL2/RET/TNF/PTGS2/SNCA/TGFB1/GSK3B/IL6/HSP90AA1/PARP1/MAPK1/EGFR/IFNG/CAV1/SRC/AKT1/BAX/AVP
GO:0043523	regulation of neuron apoptotic process	16/149	210/18670	1.21E-11	2.71E-10	9.91E-11	APOE/BCL2/TNF/CTNNB1/SNCA/CASP3/PARP1/FASLG/HMOX1/NQO1/SOD1/GRIN1/TP53/CASP9/JUN/BAX
GO:0048872	homeostasis of number of cells	17/149	246/18670	1.28E-11	2.83E-10	1.04E-10	BCL2/SOX9/TGFB1/STAT1/IL6/CASP3/HMOX1/SOD1/IL2/ABL1/RB1/THRA/ACVR1B/JAK3/AKT1/NOTCH1/BAX
GO:0010950	positive regulation of endopeptidase activity	15/149	178/18670	1.30E-11	2.87E-10	1.05E-10	FAS/CASP8/PPARG/MAPT/TNF/GRIN2B/SNCA/CTSD/MYC/S100A8/FASLG/NODAL/GRIN1/CASP9/BAX
GO:0006801	superoxide metabolic process	11/149	73/18670	1.34E-11	2.93E-10	1.07E-10	NOS2/DUOX2/CRP/MAPT/NFE2L2/TNF/MPO/TGFB1/EGFR/NQO1/SOD1
GO:0033555	multicellular organismal response to stress	11/149	73/18670	1.34E-11	2.93E-10	1.07E-10	SLC6A2/NOS1/DPP4/APOE/BCL2/CACNA1B/RET/TRPA1/TRPV1/HTR1A/DRD4
GO:0051099	positive regulation of binding	15/149	179/18670	1.41E-11	3.06E-10	1.12E-10	IGF1/MET/APOE/PON1/PPARG/CTNNB1/TGFB1/GSK3B/PARP1/FMR1/IFNG/CAV1/ABL1/RB1/MMP9
GO:1900182	positive regulation of protein localization to nucleus	11/149	74/18670	1.57E-11	3.39E-10	1.24E-10	LEP/F2/PTGS2/TGFB1/PARP1/INS/MAPK1/IFNG/SRC/AKT1/PIK3R1
GO:0048754	branching morphogenesis of an epithelial tube	14/149	150/18670	1.62E-11	3.49E-10	1.28E-10	MET/BCL2/SOX9/TNF/CTNNB1/ESR1/TGFB1/MYC/NKX2-1/FGF2/ABL1/SRC/PGR/NOTCH1
GO:0030072	peptide hormone secretion	17/149	250/18670	1.65E-11	3.53E-10	1.29E-10	NOS2/LEP/DPP4/ADRA2A/PLA2G6/TNF/DRD2/IL6/HMGCR/CACNA1C/INS/EGFR/SLC25A4/CACNA1A/IFNG/CFTR/IL1B
GO:0048015	phosphatidylinositol-mediated signaling	15/149	181/18670	1.65E-11	3.53E-10	1.29E-10	IGF1/LEP/SOX9/TNF/F2/KDR/INS/MAPK1/EGFR/ERBB2/SRC/CAT/AKT1/PIK3R1/HTR2A
GO:0001558	regulation of cell growth	21/149	416/18670	1.74E-11	3.65E-10	1.34E-10	IGF1/APOE/BCL2/PPARG/MAPT/F2/TGFB1/S100A8/GSK3B/INS/EGFR/IL2/ERBB2/ABL1/RB1/ACVR1B/TP53/CDKN1A/AKT1/IGFBP3/AVP
GO:0030099	myeloid cell differentiation	21/149	416/18670	1.74E-11	3.65E-10	1.34E-10	CASP8/PPARG/TNF/CTNNB1/TGFB1/MYC/STAT1/CASP3/PARP1/IFNG/FOS/RB1/SRC/THRA/ACVR1B/CASP9/JAK3/PIK3R1/NFKBIA/JUN/MMP9
GO:0070374	positive regulation of ERK1 and ERK2 cascade	16/149	215/18670	1.74E-11	3.65E-10	1.34E-10	PLA2G2A/APOE/TNF/DRD2/TGFB1/HMGCR/KDR/EGFR/NODAL/FGF2/ICAM1/ABL1/SRC/HTR2A/JUN/NOTCH1
GO:0010632	regulation of epithelial cell migration	18/149	291/18670	1.96E-11	4.10E-10	1.50E-10	MET/APOE/PPARG/NFE2L2/SOX9/PTGS2/TGFB1/KDR/HMOX1/IFNG/FGF2/ABL1/SRC/AKT1/SPARC/JUN/NOTCH1/MMP9

GO:0071241	cellular response to inorganic substance	16/149	217/18670	2.00E-11	4.16E-10	1.52E-10	CYP1A1/NFE2L2/PTGS2/SNCA/PARP1/HMOX1/FMR1/MAPK1/EGFR/NQO1/SOD1/FOS/AKT1/JUN/SCN5A/MMP9
GO:0048017	inositol lipid-mediated signaling	15/149	184/18670	2.09E-11	4.34E-10	1.59E-10	IGF1/LEP/SOX9/TNF/F2/KDR/INS/MAPK1/EGFR/ERBB2/SRC/CAT/AKT1/PIK3R1/HTR2A
GO:1903039	positive regulation of leukocyte cell-cell adhesion	16/149	218/18670	2.14E-11	4.42E-10	1.62E-10	IGF1/LEP/DPP4/TNF/TGFB1/IL6/IGF2/IL2/IFNG/ICAM1/CAV1/SRC/JAK3/AKT1/PIK3R1/IL1B
GO:0050730	regulation of peptidyl-tyrosine phosphorylation	17/149	256/18670	2.40E-11	4.94E-10	1.81E-10	IGF1/LEP/ADRA2A/TNF/TGFB1/IL13/IL6/IGF2/EGFR/IL2/IFNG/ICAM1/CAV1/ABL1/SRC/TP53/HTR2A
GO:0052548	regulation of endopeptidase activity	21/149	425/18670	2.59E-11	5.30E-10	1.94E-10	FAS/CASP8/PPARG/MAPT/TNF/GRIN2B/PTGS2/SNCA/CTSD/MYC/S100A8/FASLG/SERPINH1/NODAL/GRIN1/SRC/CASP9/AKT1/BAX/AVP/MMP9
GO:0007259	JAK-STAT cascade	14/149	156/18670	2.76E-11	5.59E-10	2.05E-10	IGF1/LEP/RET/TNF/F2/IL13/STAT1/IL6/IL10/IL2/IFNG/CAV1/JAK3/NOTCH1
GO:0034767	positive regulation of ion transmembrane transport	14/149	156/18670	2.76E-11	5.59E-10	2.05E-10	NOS1/HTR3A/OPRK1/ABCB1/F2/SNCA/SHANK3/IL13/KCNQ1/IFNG/ABL1/DRD4/CFTR/BAX
GO:0070663	regulation of leukocyte proliferation	16/149	222/18670	2.82E-11	5.68E-10	2.08E-10	IGF1/IRF1/LEP/BCL2/CTNNB1/TGFB1/IL13/IL6/CASP3/IL10/IGF2/IL2/ERBB2/CDKN1A/JAK3/IL1B
GO:0061900	glial cell activation	10/149	58/18670	2.91E-11	5.84E-10	2.14E-10	MAPT/TNF/SNCA/IL13/IL6/TRPV1/EGFR/IFNG/IL1B/JUN
GO:1903706	regulation of hemopoiesis	22/149	475/18670	2.93E-11	5.86E-10	2.15E-10	IRF1/CASP8/NFE2L2/TNF/CTNNB1/TGFB1/MYC/STAT1/SOD1/IL2/ERBB2/IFNG/FOS/ABL1/RB1/TCF3/ACVR1B/JAK3/PIK3R1/NFKBIA/JUN/NOTCH1
GO:0045765	regulation of angiogenesis	20/149	383/18670	2.99E-11	5.95E-10	2.18E-10	LEP/PPARG/CXCL8/NFE2L2/IL1A/PTGS2/CTNNB1/STAT1/IL6/FASLG/KDR/HMOX1/IL10/NODAL/ERBB2/FGF2/ABL1/IL1B/SPARC/NOTCH1
GO:0018105	peptidyl-serine phosphorylation	18/149	299/18670	3.07E-11	6.08E-10	2.22E-10	NOS1/BCL2/RET/TNF/PTGS2/SNCA/TGFB1/GSK3B/IL6/HSP90AA1/MAPK1/EGFR/IFNG/CAV1/SRC/AKT1/BAX/AVP
GO:0098869	cellular oxidant detoxification	12/149	102/18670	3.11E-11	6.14E-10	2.25E-10	APOE/DUOX2/NFE2L2/TNF/MPO/PTGS2/NQO1/SOD1/TPO/IYD/CAT/PTGS1
GO:0070372	regulation of ERK1 and ERK2 cascade	18/149	300/18670	3.24E-11	6.36E-10	2.33E-10	PLA2G2A/APOE/TNF/DRD2/TGFB1/HMGCR/KDR/EGFR/NODAL/ERBB2/FGF2/ICAM1/ABL1/SRC/IL1B/HTR2A/JUN/NOTCH1
GO:1901653	cellular response to peptide	20/149	385/18670	3.28E-11	6.42E-10	2.35E-10	IGF1/LEP/PPARG/NFE2L2/TGFB1/GSK3B/STAT1/PARP1/INS/IGF2/CACNA1A/ICAM1/CAV1/SRC/TP53/JAK3/AKT1/PIK3R1/NFKB1/IL1B
GO:0071248	cellular response to metal ion	15/149	190/18670	3.32E-11	6.46E-10	2.36E-10	CYP1A1/NFE2L2/PTGS2/SNCA/PARP1/HMOX1/MAPK1/EGFR/NQO1/SOD1/FOS/AKT1/JUN/SCN5A/MMP9
GO:0034504	protein localization to nucleus	17/149	262/18670	3.46E-11	6.72E-10	2.46E-10	LEP/COL1A1/SOX9/F2/PTGS2/TGFB1/GSK3B/PARP1/INS/MAPK1/IFNG/SRC/TP53/CDKN1A/AKT1/PIK3R1/NFKBIA

GO:0050731	positive regulation of peptidyl-tyrosine phosphorylation	15/149	192/18670	3.85E-11	7.43E-10	2.72E-10	IGF1/LEP/ADRA2A/TNF/TGFB1/IL13/IL6/IGF2/IL2/IFNG/ICAM1/ABL1/SRC/TP53/HTR2A
GO:1901654	response to ketone	15/149	193/18670	4.15E-11	7.97E-10	2.92E-10	PPARG/CCND1/TGFB1/PARP1/MAOB/EGFR/FOS/ICAM1/CAV1/CFTR/SRC/CASP9/CDKN1A/AKT1/AVP
GO:0043280	positive regulation of cysteine-type endopeptidase activity involved in apoptotic process	13/149	132/18670	4.48E-11	8.57E-10	3.14E-10	FAS/CASP8/PPARG/MAPT/TNF/SNCA/CTSD/MYC/S100A8/FASLG/NODAL/CASP9/BAX
GO:0031334	positive regulation of protein complex assembly	17/149	268/18670	4.95E-11	9.43E-10	3.45E-10	MET/MAPT/TNF/CTNNB1/P2RY12/ESR1/TGFB1/GSK3B/MMP1/HSP90AA1/PARP1/IFNG/ICAM1/SRC/TP53/JUN/BAX
GO:0010631	epithelial cell migration	19/149	351/18670	5.24E-11	9.95E-10	3.64E-10	MET/DPP4/APOE/PPARG/NFE2L2/SOX9/PTGS2/TGFB1/KDR/HMOX1/IFNG/FGF2/ABL1/SRC/AKT1/SPARC/JUN/NOTCH1/MMP9
GO:0045927	positive regulation of growth	17/149	270/18670	5.56E-11	1.05E-09	3.85E-10	IGF1/LEP/BCL2/MAPT/DRD2/F2/S100A8/INS/MAPK1/IGF2/EGFR/IL2/ERBB2/FGF2/AKT1/NOTCH1/AVP
GO:2001242	regulation of intrinsic apoptotic signaling pathway	14/149	165/18670	5.89E-11	1.11E-09	4.06E-10	BCL2/NFE2L2/PTGS2/S100A8/PARP1/INS/SOD1/CAV1/SRC/TP53/AKT1/SNAI2/BAX/MMP9
GO:0046890	regulation of lipid biosynthetic process	15/149	198/18670	5.97E-11	1.12E-09	4.09E-10	LEP/APOE/PLA2G6/TNF/PTGS2/HMGCR/INS/SOD1/IFNG/AKT1/NFKB1/IL1B/HTR2A/SNAI2/AVP
GO:0090132	epithelium migration	19/149	354/18670	6.06E-11	1.13E-09	4.14E-10	MET/DPP4/APOE/PPARG/NFE2L2/SOX9/PTGS2/TGFB1/KDR/HMOX1/IFNG/FGF2/ABL1/SRC/AKT1/SPARC/JUN/NOTCH1/MMP9
GO:0015696	ammonium transport	12/149	108/18670	6.17E-11	1.15E-09	4.20E-10	SLC6A2/SLC6A4/NOS1/OPRK1/SLC12A2/DRD2/SNCA/HTR1A/MAOB/SLC6A8/DRD4/HTR2A
GO:0097696	STAT cascade	14/149	166/18670	6.39E-11	1.18E-09	4.33E-10	IGF1/LEP/RET/TNF/F2/IL13/STAT1/IL6/IL10/IL2/IFNG/CAV1/JAK3/NOTCH1
GO:0046686	response to cadmium ion	10/149	63/18670	6.88E-11	1.27E-09	4.64E-10	HMOX1/MAPK1/EGFR/SOD1/FOS/CAT/AKT1/SPARC/JUN/MMP9
GO:0046425	regulation of JAK-STAT cascade	13/149	137/18670	7.18E-11	1.32E-09	4.82E-10	IGF1/LEP/RET/TNF/F2/IL13/IL6/IL10/IL2/IFNG/CAV1/JAK3/NOTCH1
GO:0090130	tissue migration	19/149	360/18670	8.08E-11	1.48E-09	5.41E-10	MET/DPP4/APOE/PPARG/NFE2L2/SOX9/PTGS2/TGFB1/KDR/HMOX1/IFNG/FGF2/ABL1/SRC/AKT1/SPARC/JUN/NOTCH1/MMP9
GO:0031331	positive regulation of cellular catabolic process	19/149	361/18670	8.48E-11	1.54E-09	5.65E-10	IGF1/APOE/ADRA2A/NFE2L2/TNF/SNCA/GSK3B/IL6/HSP90AA1/KDR/HMOX1/INS/FMR1/IFNG/CAV1/AKT1/IL1B/HTR2A/BAX

GO:0060078	regulation of postsynaptic membrane potential	13/149	140/18670	9.45E-11	1.71E-09	6.27E-10	DRD2/GRIN2B/GABRG3/SNCA/SHANK3/NRXN1/GSK3B/TRPV1/GRIN1/GABRD/DRD4/AKT1/GABRA3
GO:1990748	cellular detoxification	12/149	112/18670	9.51E-11	1.72E-09	6.28E-10	APOE/DUOX2/NFE2L2/TNF/MPO/PTGS2/NQO1/SOD1/TPO/IYD/CAT/PTGS1
GO:0071222	cellular response to lipopolysaccharide	15/149	205/18670	9.77E-11	1.76E-09	6.43E-10	NOS2/OPRK1/CXCL8/TNF/TGFB1/IL6/IL10/MAPK1/ICAM1/ABL1/SRC/AKT1/NFKB1/IL1B/NFKBIA
GO:0006953	acute-phase response	9/149	47/18670	1.08E-10	1.94E-09	7.10E-10	CRP/TNF/IL1A/F2/PTGS2/IL6/TRPV1/INS/IL1B
GO:0032103	positive regulation of response to external stimulus	18/149	323/18670	1.09E-10	1.94E-09	7.11E-10	MET/PLA2G2A/CXCL8/TNF/F2/PTGS2/P2RY12/SNCA/TGFB1/S100A8/IL6/KDR/EGFR/IL2/FGF2/IL1B/F12/NFKBIA
GO:0045428	regulation of nitric oxide biosynthetic process	10/149	66/18670	1.11E-10	1.98E-09	7.24E-10	TNF/PTGS2/TRPV1/HSP90AA1/IL10/IFNG/ICAM1/CAV1/AKT1/IL1B
GO:0038127	ERBB signaling pathway	13/149	142/18670	1.13E-10	2.00E-09	7.32E-10	ADRA2A/SOX9/TGFB1/HSP90AA1/FASLG/MAPK1/EGFR/ERBB2/ABL1/SRC/AKT1/PIK3R1/MMP9
GO:0050796	regulation of insulin secretion	14/149	176/18670	1.40E-10	2.47E-09	9.03E-10	NOS2/LEP/DPP4/ADRA2A/PLA2G6/TNF/DRD2/HMGCR/CACNA1C/SLC25A4/CACNA1A/IFNG/CFTR/IL1B
GO:1900180	regulation of protein localization to nucleus	12/149	116/18670	1.44E-10	2.53E-09	9.26E-10	LEP/F2/PTGS2/TGFB1/GSK3B/PARP1/INS/MAPK1/IFNG/SRC/AKT1/PIK3R1
GO:0019915	lipid storage	10/149	68/18670	1.51E-10	2.65E-09	9.69E-10	LEP/APOE/PPARG/CRP/TNF/IL6/CAV1/NFKB1/IL1B/NFKBIA
GO:0071219	cellular response to molecule of bacterial origin	15/149	212/18670	1.57E-10	2.73E-09	1.00E-09	NOS2/OPRK1/CXCL8/TNF/TGFB1/IL6/IL10/MAPK1/ICAM1/ABL1/SRC/AKT1/NFKB1/IL1B/NFKBIA
GO:0055123	digestive system development	13/149	146/18670	1.60E-10	2.77E-09	1.01E-09	BCL2/CYP1A1/RET/CXCL8/TNF/CTNNB1/TGFB1/IGF2/EGFR/NODAL/RB1/CDKN1A/NOTCH1
GO:1904892	regulation of STAT cascade	13/149	146/18670	1.60E-10	2.77E-09	1.01E-09	IGF1/LEP/RET/TNF/F2/IL13/IL6/IL10/IL2/IFNG/CAV1/JAK3/NOTCH1
GO:0035094	response to nicotine	9/149	49/18670	1.61E-10	2.78E-09	1.02E-09	BCL2/TNF/DRD2/IL13/CASP3/HMOX1/MAPK1/NFKB1/AVP
GO:0043062	extracellular structure organization	20/149	422/18670	1.68E-10	2.87E-09	1.05E-09	MMP2/COL1A1/DPP4/PLA2G2A/APOE/SOX9/TNF/MPO/TGFB1/MMP1/IL6/KDR/SERPINH1/FGF2/ICAM1/ABL1/RB1/SPARC/NOTCH1/MMP9
GO:1901342	regulation of vasculature development	20/149	422/18670	1.68E-10	2.87E-09	1.05E-09	LEP/PPARG/CXCL8/NFE2L2/IL1A/PTGS2/CTNNB1/STAT1/IL6/FASLG/KDR/HMOX1/IL10/NODAL/ERBB2/FGF2/ABL1/IL1B/SPARC/NOTCH1
GO:0009896	positive regulation of catabolic process	20/149	423/18670	1.75E-10	2.98E-09	1.09E-09	IGF1/APOE/ADRA2A/NFE2L2/SOX9/TNF/SNCA/GSK3B/IL6/HSP90AA1/KDR/HMOX1/INS/FMR1/IFNG/CAV1/AKT1/IL1B/HTR2A/BAX

GO:0071466	cellular response to xenobiotic stimulus	14/149	180/18670	1.89E-10	3.20E-09	1.17E-09	BCHE/CYP1A1/CYP2E1/TGFB1/GGT1/EGFR/NQO1/GRIN1/ICAM1/CACNA1S/RB1/CASP9/PTGS1/CYP3A4
GO:0055093	response to hyperoxia	7/149	21/18670	1.89E-10	3.20E-09	1.17E-09	COL1A1/FAS/CYP1A1/PPARG/CAV1/CDKN1A/CAT
GO:0042368	vitamin D biosynthetic process	6/149	12/18670	2.07E-10	3.49E-09	1.28E-09	TNF/IFNG/NFKB1/IL1B/SNAI2/CYP3A4
GO:0022612	gland morphogenesis	12/149	120/18670	2.15E-10	3.61E-09	1.32E-09	BCL2/SOX9/TNF/ESR1/TGFB1/EGFR/CAV1/SRC/PGR/SNAI2/NOTCH1/BAX
GO:0043405	regulation of MAP kinase activity	18/149	337/18670	2.17E-10	3.63E-09	1.33E-09	IGF1/ALK/APOE/ADRA2A/RET/TNF/TGFB1/HMGCR/MAPK1/EGFR/SOD1/ERBB2/FGF2/CAV1/DRD4/SRC/IL1B/HTR2A
GO:0050999	regulation of nitric-oxide synthase activity	9/149	51/18670	2.35E-10	3.92E-09	1.43E-09	LEP/APOE/TNF/HSP90AA1/INS/EGFR/CAV1/AKT1/IL1B
GO:1903169	regulation of calcium ion transmembrane transport	13/149	151/18670	2.45E-10	4.06E-09	1.49E-09	NOS1/ADRA2A/PLA2G6/DRD2/F2/SNCA/TGFB1/IL13/CACNA1C/FMR1/ABL1/DRD4/BAX
GO:0051881	regulation of mitochondrial membrane potential	10/149	72/18670	2.72E-10	4.49E-09	1.64E-09	BCL2/MAPT/TRPV1/PARP1/KDR/SOD1/ABL1/SRC/AKT1/BAX
GO:0045787	positive regulation of cell cycle	19/149	389/18670	3.01E-10	4.95E-09	1.81E-09	IGF1/SLC6A4/CYP1A1/IL1A/DRD2/CCND1/TGFB1/INS/IL10/IGF2/EGFR/ABL1/RB1/SRC/TP53/CDKN1A/AKT1/IL1B/BAX
GO:1903524	positive regulation of blood circulation	10/149	73/18670	3.13E-10	5.13E-09	1.88E-09	PTGS2/AVPR2/KCNQ1/EGFR/ICAM1/CAV1/AKT1/CHRM3/HTR2A/AVP
GO:0008637	apoptotic mitochondrial changes	12/149	124/18670	3.16E-10	5.15E-09	1.88E-09	IGF1/BCL2/CASP8/PLA2G6/GSK3B/SLC25A4/TP53/AKT1/JUN/BAX/AVP/MMP9
GO:0014066	regulation of phosphatidylinositol 3-kinase signaling	12/149	124/18670	3.16E-10	5.15E-09	1.88E-09	IGF1/LEP/SOX9/TNF/F2/KDR/INS/MAPK1/EGFR/SRC/CAT/PIK3R1
GO:0031670	cellular response to nutrient	10/149	74/18670	3.59E-10	5.82E-09	2.13E-09	LEP/COL1A1/PPARG/PTGS2/P2RY12/TRPV1/HMOX1/SOD1/SNAI2/CYP24A1
GO:0030856	regulation of epithelial cell differentiation	13/149	156/18670	3.68E-10	5.92E-09	2.17E-09	SOX9/TNF/CTNNB1/CCND1/IL13/GSK3B/STAT1/NODAL/IFNG/CAV1/IL1B/NOTCH1/MMP9
GO:0042129	regulation of T cell proliferation	13/149	156/18670	3.68E-10	5.92E-09	2.17E-09	IGF1/IRF1/LEP/CTNNB1/TGFB1/IL6/CASP3/IL10/IGF2/IL2/ERBB2/JAK3/IL1B
GO:0071276	cellular response to cadmium ion	8/149	37/18670	4.32E-10	6.94E-09	2.54E-09	HMOX1/MAPK1/EGFR/SOD1/FOS/AKT1/JUN/MMP9
GO:0010876	lipid localization	19/149	400/18670	4.80E-10	7.68E-09	2.81E-09	NOS2/LEP/PLA2G2A/APOE/PON1/PPARG/CRP/ABCB1/PLA2G6/TNF/DRD2/IL6/CAV1/DRD4/CFTR/AKT1/NFKB1/IL1B/NFKBIA

GO:0010522	regulation of calcium ion transport into cytosol	11/149	102/18670	5.51E-10	8.79E-09	3.22E-09	NOS1/BCL2/F2/SNCA/TGFB1/IL13/CACNA1C/GRIN1/CAV1/ABL1/BAX
GO:1902105	regulation of leukocyte differentiation	16/149	272/18670	5.74E-10	9.12E-09	3.34E-09	IRF1/CASP8/TNF/CTNNB1/TGFB1/MYC/SOD1/IL2/ERBB2/IFNG/FOS/ABL1/RB1/JAK3/PIK3R1/JUN
GO:0098754	detoxification	12/149	131/18670	6.00E-10	9.50E-09	3.48E-09	APOE/DUOX2/NFE2L2/TNF/MPO/PTGS2/NQO1/SOD1/TPO/IYD/CAT/PTGS1
GO:0018958	phenol-containing compound metabolic process	11/149	103/18670	6.13E-10	9.67E-09	3.54E-09	BCL2/DUOX2/DRD2/CYP2E1/SNCA/HTR1A/MAOB/TPO/DRD4/IYD/PAH
GO:0042743	hydrogen peroxide metabolic process	9/149	57/18670	6.69E-10	1.04E-08	3.82E-09	CYP1A1/DUOX2/MPO/SNCA/MAOB/EGFR/SOD1/TPO/CAT
GO:0043525	positive regulation of neuron apoptotic process	9/149	57/18670	6.69E-10	1.04E-08	3.82E-09	TNF/CTNNB1/CASP3/FASLG/NQO1/TP53/CASP9/JUN/BAX
GO:1903428	positive regulation of reactive oxygen species biosynthetic process	9/149	57/18670	6.69E-10	1.04E-08	3.82E-09	TNF/PTGS2/TRPV1/HSP90AA1/GRIN1/IFNG/ICAM1/AKT1/IL1B
GO:0048565	digestive tract development	12/149	134/18670	7.81E-10	1.21E-08	4.44E-09	BCL2/CYP1A1/RET/CXCL8/TNF/CTNNB1/TGFB1/EGFR/NODAL/RB1/CDKN1A/NOTCH1
GO:0060485	mesenchyme development	16/149	278/18670	7.89E-10	1.22E-08	4.48E-09	COL1A1/BCL2/RET/SOX9/CTNNB1/TGFB1/MYC/NKX2-1/GSK3B/STAT1/IL6/MAPK1/NODAL/IL1B/SNAI2/NOTCH1
GO:0002683	negative regulation of immune system process	20/149	463/18670	8.49E-10	1.31E-08	4.79E-09	IRF1/PPARG/NFE2L2/SOX9/TNF/DRD2/CTNNB1/TGFB1/MYC/CASP3/HMOX1/INS/IL10/IL2/ERBB2/JAK3/AKT1/PIK3R1/NFKBIA/NOTCH1
GO:0060562	epithelial tube morphogenesis	17/149	322/18670	8.51E-10	1.31E-08	4.79E-09	MET/BCL2/RET/SOX9/TNF/CTNNB1/ESR1/TGFB1/MYC/NKX2-1/CASP3/NODAL/FGF2/ABL1/SRC/PGR/NOTCH1
GO:0050870	positive regulation of T cell activation	14/149	202/18670	8.65E-10	1.33E-08	4.86E-09	IGF1/LEP/DPP4/TGFB1/IL6/IGF2/IL2/IFNG/CAV1/SRC/JAK3/AKT1/PIK3R1/IL1B
GO:0001836	release of cytochrome c from mitochondria	9/149	59/18670	9.21E-10	1.40E-08	5.14E-09	IGF1/BCL2/PLA2G6/TP53/AKT1/JUN/BAX/AVP/MMP9
GO:0033619	membrane protein proteolysis	9/149	59/18670	9.21E-10	1.40E-08	5.14E-09	APOE/ADRA2A/RET/TNF/TGFB1/IL10/IFNG/NFKB1/IL1B
GO:0002573	myeloid leukocyte differentiation	14/149	204/18670	9.84E-10	1.49E-08	5.47E-09	CASP8/PPARG/TNF/CTNNB1/TGFB1/MYC/PARP1/IFNG/FOS/RB1/SRC/PIK3R1/JUN/MMP9
GO:0042362	fat-soluble vitamin biosynthetic process	6/149	15/18670	1.10E-09	1.67E-08	6.10E-09	TNF/IFNG/NFKB1/IL1B/SNAI2/CYP3A4

GO:0006109	regulation of carbohydrate metabolic process	14/149	206/18670	1.12E-09	1.69E-08	6.17E-09	IGF1/LEP/SNCA/TGFB1/GSK3B/INS/IGF2/IFNG/SRC/TP53/AKT1/NFKB1/IGFBP3/HTR2A
GO:0006970	response to osmotic stress	10/149	83/18670	1.14E-09	1.72E-08	6.29E-09	SLC12A2/ABCB1/TNF/PTGS2/CASP3/EGFR/MYLK/TP53/BAX/AVP
GO:0030073	insulin secretion	14/149	207/18670	1.19E-09	1.78E-08	6.53E-09	NOS2/LEP/DPP4/ADRA2A/PLA2G6/TNF/DRD2/HMGCR/CACNA1C/SLC25A4/CACNA1A/IFNG/CFTR/IL1B
GO:0030324	lung development	13/149	172/18670	1.23E-09	1.84E-08	6.73E-09	THRB/SOX9/TNF/CTNNB1/NKX2-1/IL13/MAPK1/EGFR/NODAL/PGR/THRA/SPARC/NOTCH1
GO:0050769	positive regulation of neurogenesis	20/149	474/18670	1.27E-09	1.90E-08	6.94E-09	ALK/APOE/BCL2/PPARG/RET/MAPT/NFE2L2/TNF/DRD2/CTNNB1/P2RY12/TGFB1/SHANK3/IL6/FMR1/IL2/IFNG/TCF3/IL1B/NOTCH1
GO:0046889	positive regulation of lipid biosynthetic process	10/149	84/18670	1.29E-09	1.91E-08	6.99E-09	APOE/PLA2G6/TNF/PTGS2/INS/IFNG/AKT1/IL1B/HTR2A/AVP
GO:0008202	steroid metabolic process	17/149	331/18670	1.29E-09	1.91E-08	7.00E-09	LEP/APOE/PON1/CYP1A1/TNF/CYP2E1/ESR1/HMGCR/SOD1/IFNG/CFTR/CAT/NFKB1/IL1B/SNAI2/CYP24A1/CYP3A4
GO:1904019	epithelial cell apoptotic process	11/149	111/18670	1.38E-09	2.03E-08	7.43E-09	NFE2L2/TNF/IL13/IL6/FASLG/KDR/HMOX1/IL10/ICAM1/ABL1/RB1
GO:0032147	activation of protein kinase activity	17/149	333/18670	1.42E-09	2.08E-08	7.62E-09	IGF1/LEP/ALK/ADRA2A/RET/TNF/DRD2/INS/MAPK1/EGFR/SOD1/FGF2/ABL1/DRD4/SRC/AKT1/IL1B
GO:1904705	regulation of vascular smooth muscle cell proliferation	10/149	85/18670	1.45E-09	2.11E-08	7.73E-09	MMP2/IGF1/PPARG/TNF/HMOX1/IL10/FGF2/CDKN1A/JUN/MMP9
GO:1990874	vascular smooth muscle cell proliferation	10/149	85/18670	1.45E-09	2.11E-08	7.73E-09	MMP2/IGF1/PPARG/TNF/HMOX1/IL10/FGF2/CDKN1A/JUN/MMP9
GO:0046824	positive regulation of nucleocytoplasmic transport	9/149	62/18670	1.46E-09	2.11E-08	7.73E-09	LEP/PTGS2/TGFB1/GSK3B/MAPK1/IFNG/TP53/PIK3R1/IL1B
GO:0003015	heart process	16/149	290/18670	1.46E-09	2.11E-08	7.73E-09	NOS1/THRB/CACNA1B/DRD2/KCNQ1/TRPV1/ABCC9/CACNA1C/SOD1/IL2/CAV1/CACNA1S/SRC/THRA/SCN10A/SCN5A
GO:0051251	positive regulation of lymphocyte activation	17/149	334/18670	1.48E-09	2.14E-08	7.84E-09	IGF1/LEP/DPP4/BCL2/TGFB1/IL13/IL6/IGF2/IL2/IFNG/CAV1/SRC/CDKN1A/JAK3/AKT1/PIK3R1/IL1B
GO:0002696	positive regulation of leukocyte activation	18/149	380/18670	1.49E-09	2.14E-08	7.84E-09	IGF1/LEP/DPP4/BCL2/TGFB1/IL13/IL6/IL10/IGF2/IL2/IFNG/CAV1/SRC/CDKN1A/JAK3/AKT1/PIK3R1/IL1B
GO:0042176	regulation of protein catabolic process	18/149	381/18670	1.55E-09	2.22E-08	8.14E-09	NOS2/APOE/ADRA2A/NFE2L2/SOX9/TNF/SNCA/GSK3B/HSP90AA1/HMGCR/INS/FMR1/IL10/EGFR/IFNG/CAV1/AKT1/IL1B

GO:0006775	fat-soluble vitamin metabolic process	8/149	43/18670	1.56E-09	2.22E-08	8.14E-09	CYP1A1/TNF/IFNG/NFKB1/IL1B/SNAI2/CYP24A1/CYP3A4
GO:0045429	positive regulation of nitric oxide biosynthetic process	8/149	43/18670	1.56E-09	2.22E-08	8.14E-09	TNF/PTGS2/TRPV1/HSP90AA1/IFNG/ICAM1/AKT1/IL1B
GO:0006919	activation of cysteine-type endopeptidase activity involved in apoptotic process	10/149	86/18670	1.63E-09	2.30E-08	8.42E-09	FAS/CASP8/PPARG/MAPT/TNF/SNCA/S100A8/FASLG/CASP9/BAX
GO:0001936	regulation of endothelial cell proliferation	13/149	176/18670	1.63E-09	2.30E-08	8.42E-09	LEP/APOE/PPARG/TNF/STAT1/KDR/HMOX1/IL10/FGF2/CAV1/AKT1/SPARC/JUN
GO:0009408	response to heat	13/149	176/18670	1.63E-09	2.30E-08	8.42E-09	IGF1/NOS1/MAPT/IL1A/PTGS2/GSK3B/TRPV1/HSP90AA1/HMOX1/MAPK1/SOD1/CDKN1A/AKT1
GO:0030323	respiratory tube development	13/149	176/18670	1.63E-09	2.30E-08	8.42E-09	THRB/SOX9/TNF/CTNNB1/NKX2-1/IL13/MAPK1/EGFR/NODAL/PGR/THRA/SPARC/NOTCH1
GO:0014068	positive regulation of phosphatidylinositol 3-kinase signaling	10/149	87/18670	1.83E-09	2.57E-08	9.40E-09	IGF1/LEP/SOX9/TNF/F2/KDR/INS/SRC/CAT/PIK3R1
GO:0036296	response to increased oxygen levels	7/149	28/18670	1.84E-09	2.58E-08	9.43E-09	COL1A1/FAS/CYP1A1/PPARG/CAV1/CDKN1A/CAT
GO:0048511	rhythmic process	16/149	295/18670	1.87E-09	2.60E-08	9.53E-09	NOS2/SLC6A4/LEP/OPRK1/PPARG/DRD2/ESR1/NKX2-1/GSK3B/CASP3/EGFR/DRD4/SRC/PGR/TP53/JUN
GO:1904407	positive regulation of nitric oxide metabolic process	8/149	44/18670	1.89E-09	2.63E-08	9.64E-09	TNF/PTGS2/TRPV1/HSP90AA1/IFNG/ICAM1/AKT1/IL1B
GO:1903034	regulation of response to wounding	13/149	179/18670	2.01E-09	2.79E-08	1.02E-08	PLAU/APOE/ADRA2A/DUOX2/NFE2L2/F2/HMGCR/IL10/MYLK/FGF2/CAV1/CDKN1A/F12
GO:0046427	positive regulation of JAK-STAT cascade	10/149	89/18670	2.29E-09	3.17E-08	1.16E-08	IGF1/LEP/TNF/F2/IL13/IL6/IL10/IL2/IFNG/NOTCH1
GO:0010721	negative regulation of cell development	17/149	344/18670	2.32E-09	3.19E-08	1.17E-08	IGF1/SLC6A4/APOE/BCL2/THRB/SOX9/TNF/F2/CTNNB1/TGFB1/GSK3B/IL6/TRPV1/NODAL/TP53/IL1B/NOTCH1
GO:0043406	positive regulation of MAP kinase activity	15/149	258/18670	2.39E-09	3.29E-08	1.20E-08	IGF1/ALK/ADRA2A/RET/TNF/TGFB1/MAPK1/EGFR/SOD1/ERBB2/FGF2/DRD4/SRC/IL1B/HTR2A
GO:0061041	regulation of wound healing	12/149	148/18670	2.46E-09	3.36E-08	1.23E-08	PLAU/APOE/ADRA2A/DUOX2/NFE2L2/F2/HMGCR/MYLK/FGF2/CAV1/CDKN1A/F12

GO:0008217	regulation of blood pressure	13/149	182/18670	2.46E-09	3.36E-08	1.23E-08	NOS2/LEP/NOS1/PPARG/CACNA1B/DRD2/PTGS2/AVPR2/TRPV1/HMOX1/SOD1/PTGS1/AVP
GO:0048762	mesenchymal cell differentiation	14/149	219/18670	2.48E-09	3.37E-08	1.23E-08	COL1A1/BCL2/RET/SOX9/CTNNB1/TGFB1/NKX2-1/GSK3B/STAT1/IL6/MAPK1/IL1B/SNAI2/NOTCH1
GO:0051926	negative regulation of calcium ion transport	9/149	66/18670	2.59E-09	3.51E-08	1.29E-08	NOS1/BCL2/ADRA2A/DRD2/PTGS2/TGFB1/FMR1/ICAM1/DRD4
GO:0050867	positive regulation of cell activation	18/149	394/18670	2.63E-09	3.57E-08	1.30E-08	IGF1/LEP/DPP4/BCL2/TGFB1/IL13/IL6/IL10/IGF2/IL2/IFNG/CAV1/SRC/CDKN1A/JAK3/AKT1/PIK3R1/IL1B
GO:0032412	regulation of ion transmembrane transporter activity	15/149	260/18670	2.66E-09	3.59E-08	1.31E-08	NOS1/ADRA2A/HTR3A/ABCB1/PLA2G6/DRD2/GRIN2B/SHANK3/FMR1/GRIN1/IFNG/CAV1/DRD4/CFTR/MMP9
GO:0050900	leukocyte migration	20/149	499/18670	3.07E-09	4.13E-08	1.51E-08	LEP/COL1A1/RET/CXCL8/SLC12A2/TNF/F2/P2RY12/TGFB1/S100A8/MMP1/IL6/HMOX1/IL10/ICAM1/CAV1/SRC/AKT1/PIK3R1/IL1B
GO:1904894	positive regulation of STAT cascade	10/149	92/18670	3.19E-09	4.28E-08	1.56E-08	IGF1/LEP/TNF/F2/IL13/IL6/IL10/IL2/IFNG/NOTCH1
GO:0030098	lymphocyte differentiation	17/149	353/18670	3.42E-09	4.57E-08	1.67E-08	IRF1/LEP/BCL2/CTNNB1/TGFB1/IL6/IL10/SOD1/IL2/ERBB2/IFNG/ABL1/TCF3/TP53/JAK3/PIK3R1/BAX
GO:0048167	regulation of synaptic plasticity	13/149	187/18670	3.43E-09	4.57E-08	1.67E-08	APOE/MAPT/DRD2/GRIN2B/PTGS2/SNCA/SHANK3/GSK3B/INS/FMR1/MAPK1/GRIN1/ABL1
GO:0009895	negative regulation of catabolic process	16/149	308/18670	3.47E-09	4.61E-08	1.69E-08	NOS2/MET/LEP/BCL2/ADRA2A/TNF/SNCA/HMGCR/HMOX1/INS/FMR1/IL10/EGFR/TP53/AKT1/IL1B
GO:0001774	microglial cell activation	8/149	48/18670	3.92E-09	5.18E-08	1.90E-08	MAPT/TNF/SNCA/IL13/IL6/TRPV1/IFNG/JUN
GO:0002269	leukocyte activation involved in inflammatory response	8/149	48/18670	3.92E-09	5.18E-08	1.90E-08	MAPT/TNF/SNCA/IL13/IL6/TRPV1/IFNG/JUN
GO:0051054	positive regulation of DNA metabolic process	14/149	228/18670	4.17E-09	5.49E-08	2.01E-08	CTNNB1/TGFB1/MYC/IL6/HSP90AA1/PARP1/MAPK1/EGFR/IL2/FGF2/SRC/AKT1/JUN/BAX
GO:0001935	endothelial cell proliferation	13/149	191/18670	4.43E-09	5.82E-08	2.13E-08	LEP/APOE/PPARG/TNF/STAT1/KDR/HMOX1/IL10/FGF2/CAV1/AKT1/SPARC/JUN
GO:0046717	acid secretion	11/149	124/18670	4.51E-09	5.89E-08	2.15E-08	NOS2/LEP/PLA2G2A/PLA2G6/DRD2/SNCA/KCNQ1/TRPV1/DRD4/IL1B/AVP
GO:0051101	regulation of DNA binding	11/149	124/18670	4.51E-09	5.89E-08	2.15E-08	IGF1/PPARG/CTNNB1/TGFB1/PARP1/HMOX1/IFNG/RB1/NFKBIA/JUN/MMP9
GO:0006936	muscle contraction	17/149	360/18670	4.59E-09	5.97E-08	2.19E-08	NOS1/ADRA2A/PLA2G6/DRD2/PTGS2/KCNQ1/TRPV1/CACNA1C/MYLK/SOD1/CAV1/SLC6A8/CACNA1S/CHRM3/HTR2A/SCN10A/SCN5A

GO:0097300	programmed necrotic cell death	8/149	49/18670	4.66E-09	6.05E-08	2.21E-08	FAS/CASP8/TNF/FASLG/SLC25A4/CAV1/TP53/BAX
GO:1901617	organic hydroxy compound biosynthetic process	15/149	271/18670	4.68E-09	6.05E-08	2.22E-08	LEP/APOE/TNF/SNCA/HMGCR/SOD1/IFNG/FGF2/CFTR/TP53/NFKB1/IL1B/SNAI2/CYP3A4/PAH
GO:0043112	receptor metabolic process	13/149	192/18670	4.72E-09	6.09E-08	2.23E-08	APOE/PPARG/CXCL8/TNF/DRD2/SNCA/TGFB1/ACHE/FMR1/IL10/IFNG/CAV1/DRD4
GO:1905952	regulation of lipid localization	12/149	157/18670	4.82E-09	6.21E-08	2.27E-08	LEP/APOE/PON1/PPARG/CRP/PLA2G6/TNF/IL6/AKT1/NFKB1/IL1B/NFKBIA
GO:0032868	response to insulin	15/149	272/18670	4.92E-09	6.31E-08	2.31E-08	LEP/OPRK1/PPARG/GSK3B/STAT1/PARP1/INS/IL10/IGF2/ICAM1/SRC/CAT/AKT1/PIK3R1/IL1B
GO:0015908	fatty acid transport	10/149	97/18670	5.37E-09	6.88E-08	2.52E-08	NOS2/LEP/PLA2G2A/APOE/PPARG/PLA2G6/DRD2/DRD4/AKT1/IL1B
GO:0006066	alcohol metabolic process	17/149	364/18670	5.41E-09	6.91E-08	2.53E-08	IGF1/LEP/APOE/PON1/CYP1A1/TNF/ADH1C/SNCA/HMGCR/SOD1/IFNG/FGF2/CFTR/CAT/NFKB1/IL1B/CYP3A4
GO:0010888	negative regulation of lipid storage	6/149	19/18670	5.82E-09	7.40E-08	2.71E-08	LEP/PPARG/CRP/TNF/IL6/NFKBIA
GO:0071375	cellular response to peptide hormone stimulus	16/149	321/18670	6.25E-09	7.93E-08	2.90E-08	LEP/PPARG/NFE2L2/TGFB1/GSK3B/STAT1/PARP1/INS/IGF2/CAV1/SRC/JAK3/AKT1/PIK3R1/NFKB1/IL1B
GO:0034763	negative regulation of transmembrane transport	11/149	128/18670	6.31E-09	7.98E-08	2.92E-08	LEP/ADRA2A/TNF/DRD2/TGFB1/FMR1/CAV1/DRD4/AKT1/IL1B/MMP9
GO:0030198	extracellular matrix organization	17/149	368/18670	6.37E-09	8.04E-08	2.94E-08	MMP2/COL1A1/DPP4/SOX9/TNF/TGFB1/MMP1/IL6/KDR/SERPINH1/FGF2/ICAM1/ABL1/RB1/SPARC/NOTCH1/MMP9
GO:0042326	negative regulation of phosphorylation	19/149	468/18670	6.40E-09	8.05E-08	2.95E-08	APOE/MAPT/DRD2/SNCA/TGFB1/MYC/CASP3/HMGCR/IL2/IFNG/CAV1/ABL1/RB1/CDKN1A/AKT1/IL1B/IGFBP3/JUN/BAX
GO:0048146	positive regulation of fibroblast proliferation	8/149	51/18670	6.49E-09	8.14E-08	2.98E-08	IGF1/ESR1/TGFB1/MYC/IL13/EGFR/CDKN1A/JUN
GO:0060541	respiratory system development	13/149	198/18670	6.85E-09	8.57E-08	3.14E-08	THRB/SOX9/TNF/CTNNB1/NKX2-1/IL13/MAPK1/EGFR/NODAL/PGR/THRA/SPARC/NOTCH1
GO:0030522	intracellular receptor signaling pathway	15/149	280/18670	7.27E-09	9.05E-08	3.31E-08	LEP/THRB/CASP8/PPARG/CTNNB1/ESR1/PARP1/NODAL/RB1/SRC/PGR/THRA/NFKBIA/SNAI2/CYP24A1
GO:0060047	heart contraction	15/149	280/18670	7.27E-09	9.05E-08	3.31E-08	NOS1/THRB/CACNA1B/DRD2/KCNQ1/TRPV1/ABCC9/CACNA1C/SOD1/IL2/CAV1/CACNA1S/THRA/SCN10A/SCN5A
GO:0045931	positive regulation of mitotic cell cycle	12/149	163/18670	7.38E-09	9.14E-08	3.34E-08	IGF1/CYP1A1/IL1A/CCND1/TGFB1/INS/IGF2/EGFR/ABL1/RB1/AKT1/IL1B

GO:0001660	fever generation	5/149	10/18670	7.39E-09	9.14E-08	3.34E-08	TNF/IL1A/PTGS2/TRPV1/IL1B
GO:0046545	development of primary female sexual characteristics	10/149	101/18670	7.99E-09	9.86E-08	3.61E-08	LEP/BCL2/ESR1/CASP3/SOD1/ICAM1/SRC/PGR/ACVR1B/BAX
GO:0045598	regulation of fat cell differentiation	11/149	132/18670	8.73E-09	1.07E-07	3.93E-08	LEP/PRDM16/PPARG/TNF/PTGS2/TGFB1/IL6/INS/AKT1/HTR2A/SNAI2
GO:0051348	negative regulation of transferase activity	15/149	285/18670	9.23E-09	1.13E-07	4.15E-08	APOE/PPARG/MAPT/GSK3B/CASP3/HMGCR/IFNG/CAV1/ABL1/RB1/SRC/TP53/CDKN1A/AKT1/IL1B
GO:0001933	negative regulation of protein phosphorylation	18/149	429/18670	9.94E-09	1.21E-07	4.44E-08	APOE/DRD2/SNCA/TGFB1/MYC/CASP3/HMGCR/IL2/IFNG/CAV1/ABL1/RB1/CDKN1A/AKT1/IL1B/IGFBP3/JUN/BAX
GO:0051052	regulation of DNA metabolic process	18/149	429/18670	9.94E-09	1.21E-07	4.44E-08	PPARG/CTNNB1/TGFB1/MYC/IL6/HSP90AA1/PARP1/IL10/MAPK1/EGFR/IL2/FGF2/SRC/TP53/CDKN1A/AKT1/JUN/BAX
GO:0051353	positive regulation of oxidoreductase activity	8/149	54/18670	1.04E-08	1.26E-07	4.62E-08	APOE/TNF/SNCA/INS/IFNG/ABL1/AKT1/IL1B
GO:1904645	response to amyloid-beta	8/149	54/18670	1.04E-08	1.26E-07	4.62E-08	MMP2/IGF1/CACNA1B/GSK3B/PARP1/CACNA1A/ICAM1/MMP9
GO:0008630	intrinsic apoptotic signaling pathway in response to DNA damage	10/149	104/18670	1.06E-08	1.28E-07	4.70E-08	BCL2/TNF/HMOX1/ABL1/TP53/CASP9/CDKN1A/PIK3R1/SNAI2/BAX
GO:0071887	leukocyte apoptotic process	10/149	104/18670	1.06E-08	1.28E-07	4.70E-08	IL6/CASP3/FASLG/IL10/IL2/TP53/CASP9/JAK3/AKT1/BAX
GO:0001889	liver development	11/149	135/18670	1.11E-08	1.33E-07	4.86E-08	MET/CYP1A1/SOX9/CCND1/TGFB1/HMOX1/IL10/EGFR/NODAL/JUN/NOTCH1
GO:0060359	response to ammonium ion	11/149	135/18670	1.11E-08	1.33E-07	4.86E-08	HTR3A/OPRK1/DRD2/SNCA/CASP3/MAPK1/GRIN1/ABL1/DRD4/CHRM3/HTR2A
GO:0010742	macrophage derived foam cell differentiation	7/149	36/18670	1.23E-08	1.47E-07	5.36E-08	PLA2G2A/PPARG/CRP/TGFB1/STAT1/NFKB1/NFKBIA
GO:0090077	foam cell differentiation	7/149	36/18670	1.23E-08	1.47E-07	5.36E-08	PLA2G2A/PPARG/CRP/TGFB1/STAT1/NFKB1/NFKBIA
GO:0090322	regulation of superoxide metabolic process	7/149	36/18670	1.23E-08	1.47E-07	5.36E-08	CRP/MAPT/NFE2L2/TNF/TGFB1/EGFR/SOD1
GO:1901215	negative regulation of neuron death	13/149	208/18670	1.24E-08	1.47E-07	5.38E-08	APOE/BCL2/CTNNB1/SNCA/IL13/GSK3B/HMOX1/IL10/SOD1/GRIN1/AKT1/JUN/BAX
GO:0071383	cellular response to steroid hormone stimulus	14/149	250/18670	1.35E-08	1.60E-07	5.86E-08	THRB/PPARG/CTNNB1/ESR1/TGFB1/PARP1/EGFR/NODAL/ICAM1/RB1/SRC/PGR/THRA/CASP9

GO:0061008	hepaticobiliary system development	11/149	138/18670	1.39E-08	1.65E-07	6.03E-08	MET/CYP1A1/SOX9/CCND1/TGFB1/HMOX1/IL10/EGFR/NODAL/JUN/NOTCH1
GO:0062014	negative regulation of small molecule metabolic process	10/149	107/18670	1.40E-08	1.65E-07	6.05E-08	APOE/SNCA/TGFB1/PARP1/INS/SOD1/TP53/AKT1/NFKB1/SNAI2
GO:0019932	second-messenger-mediated signaling	18/149	439/18670	1.42E-08	1.67E-07	6.10E-08	IGF1/NOS2/NOS1/APOE/ADRA2A/MAPT/CXCL8/SOX9/TNF/DRD2/GRIN2B/P2RY12/GSK3B/CACNA1C/KDR/INS/EGFR/GRIN1
GO:0008016	regulation of heart contraction	14/149	251/18670	1.42E-08	1.67E-07	6.10E-08	NOS1/THRB/CACNA1B/DRD2/KCNQ1/TRPV1/ABCC9/CACNA1C/IL2/CAV1/CACNA1S/THRA/SCN10A/SCN5A
GO:0032885	regulation of polysaccharide biosynthetic process	7/149	37/18670	1.51E-08	1.76E-07	6.44E-08	IGF1/TGFB1/GSK3B/INS/IGF2/AKT1/NFKB1
GO:0043029	T cell homeostasis	7/149	37/18670	1.51E-08	1.76E-07	6.44E-08	BCL2/TGFB1/CASP3/IL2/JAK3/AKT1/BAX
GO:0099565	chemical synaptic transmission, postsynaptic	10/149	108/18670	1.54E-08	1.79E-07	6.54E-08	DRD2/GRIN2B/SNCA/SHANK3/NRXN1/GSK3B/TRPV1/GRIN1/DRD4/AKT1
GO:0042737	drug catabolic process	11/149	140/18670	1.62E-08	1.88E-07	6.88E-08	NOS1/DUOX2/MPO/CYP2E1/SNCA/ACHE/MAOB/TPO/CAT/CYP3A4/PAH
GO:0046165	alcohol biosynthetic process	12/149	175/18670	1.64E-08	1.90E-07	6.95E-08	LEP/APOE/TNF/SNCA/HMGCR/SOD1/IFNG/FGF2/CFTR/NFKB1/IL1B/CYP3A4
GO:0090068	positive regulation of cell cycle process	15/149	298/18670	1.68E-08	1.94E-07	7.09E-08	IGF1/CYP1A1/IL1A/DRD2/CCND1/TGFB1/INS/IGF2/EGFR/RB1/TP53/CDKN1A/AKT1/IL1B/BAX
GO:0006939	smooth muscle contraction	10/149	110/18670	1.83E-08	2.11E-07	7.73E-08	ADRA2A/PLA2G6/DRD2/PTGS2/TRPV1/MYLK/SOD1/CAV1/CHRM3/HTR2A
GO:0034644	cellular response to UV	9/149	82/18670	1.84E-08	2.11E-07	7.73E-08	PTGS2/MYC/PARP1/FMR1/TP53/CASP9/CDKN1A/PIK3R1/BAX
GO:0048638	regulation of developmental growth	16/149	347/18670	1.88E-08	2.15E-07	7.86E-08	IGF1/SLC6A4/LEP/APOE/BCL2/MAPT/DRD2/GSK3B/MAPK1/IGF2/SOD1/FGF2/ABL1/CDKN1A/AKT1/NOTCH1
GO:0045913	positive regulation of carbohydrate metabolic process	9/149	83/18670	2.05E-08	2.34E-07	8.57E-08	IGF1/SNCA/INS/IGF2/IFNG/SRC/AKT1/NFKB1/HTR2A
GO:0051043	regulation of membrane protein ectodomain proteolysis	6/149	23/18670	2.11E-08	2.39E-07	8.75E-08	APOE/ADRA2A/TNF/IL10/IFNG/IL1B

GO:0060479	lung cell differentiation	6/149	23/18670	2.11E-08	2.39E-07	8.75E-08	THRB/SOX9/CTNNB1/NKX2-1/IL13/THRA
GO:0048771	tissue remodeling	12/149	179/18670	2.11E-08	2.39E-07	8.75E-08	MMP2/LEP/IL1A/CTNNB1/TGFB1/IL6/EGFR/IL2/CAV1/SRC/TP53/BAX
GO:2001235	positive regulation of apoptotic signaling pathway	12/149	179/18670	2.11E-08	2.39E-07	8.75E-08	FAS/BCL2/CASP8/RET/PLA2G6/S100A8/GSK3B/SOD1/CAV1/TP53/BAX/MMP9
GO:0043388	positive regulation of DNA binding	8/149	59/18670	2.14E-08	2.41E-07	8.82E-08	IGF1/PPARG/CTNNB1/TGFB1/PARP1/IFNG/RB1/MMP9
GO:0097755	positive regulation of blood vessel diameter	8/149	59/18670	2.14E-08	2.41E-07	8.82E-08	LEP/NOS1/APOE/PLA2G6/HMOX1/INS/EGFR/SOD1
GO:0050807	regulation of synapse organization	13/149	218/18670	2.17E-08	2.43E-07	8.91E-08	APOE/TNF/DRD2/GRIN2B/SNCA/SHANK3/NRXN1/INS/FMR1/IL10/GRIN1/ABL1/SPARC
GO:1904064	positive regulation of cation transmembrane transport	11/149	144/18670	2.17E-08	2.44E-07	8.91E-08	NOS1/OPRK1/F2/SNCA/SHANK3/IL13/KCNQ1/IFNG/ABL1/DRD4/BAX
GO:0010623	programmed cell death involved in cell development	5/149	12/18670	2.29E-08	2.55E-07	9.34E-08	BCL2/IL1A/FASLG/IL1B/BAX
GO:0033197	response to vitamin E	5/149	12/18670	2.29E-08	2.55E-07	9.34E-08	LEP/COL1A1/PPARG/CCND1/CAT
GO:0002526	acute inflammatory response	13/149	220/18670	2.42E-08	2.69E-07	9.83E-08	PPARG/CRP/TNF/IL1A/F2/PTGS2/S100A8/IL6/TRPV1/INS/ICAM1/IL1B/F12
GO:0010821	regulation of mitochondrion organization	12/149	182/18670	2.55E-08	2.82E-07	1.03E-07	IGF1/BCL2/CASP8/MAPT/PLA2G6/GSK3B/KDR/TP53/AKT1/BAX/AVP/MMP9
GO:0010212	response to ionizing radiation	11/149	147/18670	2.70E-08	2.98E-07	1.09E-07	BCL2/CCND1/TGFB1/MYC/CASP3/PARP1/ICAM1/TP53/CDKN1A/SNAI2/BAX
GO:0009110	vitamin biosynthetic process	6/149	24/18670	2.79E-08	3.06E-07	1.12E-07	TNF/IFNG/NFKB1/IL1B/SNAI2/CYP3A4
GO:1905953	negative regulation of lipid localization	8/149	61/18670	2.81E-08	3.06E-07	1.12E-07	LEP/PPARG/CRP/TNF/IL6/AKT1/NFKB1/NFKBIA
GO:0001776	leukocyte homeostasis	9/149	86/18670	2.81E-08	3.06E-07	1.12E-07	BCL2/TGFB1/IL6/CASP3/IL2/ABL1/JAK3/AKT1/BAX
GO:0007260	tyrosine phosphorylation of STAT protein	9/149	86/18670	2.81E-08	3.06E-07	1.12E-07	IGF1/LEP/TNF/IL13/IL6/IL2/IFNG/CAV1/JAK3

GO:0008625	extrinsic apoptotic signaling pathway via death domain receptors	9/149	86/18670	2.81E-08	3.06E-07	1.12E-07	FAS/BCL2/CASP8/TNF/FASLG/HMOX1/ICAM1/PIK3R1/BAX
GO:0060291	long-term synaptic potentiation	9/149	86/18670	2.81E-08	3.06E-07	1.12E-07	APOE/DRD2/GRIN2B/SNCA/SHANK3/GSK3B/INS/MAPK1/ABL1
GO:0032963	collagen metabolic process	10/149	115/18670	2.82E-08	3.06E-07	1.12E-07	MMP2/COL1A1/PPARG/F2/CTSD/TGFB1/MMP1/IL6/SERPINH1/MMP9
GO:0046660	female sex differentiation	10/149	115/18670	2.82E-08	3.06E-07	1.12E-07	LEP/BCL2/ESR1/CASP3/SOD1/ICAM1/SRC/PGR/ACVR1B/BAX
GO:0042113	B cell activation	15/149	310/18670	2.84E-08	3.06E-07	1.12E-07	BCL2/CASP8/TGFB1/IL13/IL6/CASP3/IL10/IL2/ABL1/TCF3/TP53/CDKN1A/JAK3/PIK3R1/BAX
GO:0045137	development of primary sexual characteristics	13/149	223/18670	2.84E-08	3.06E-07	1.12E-07	LEP/BCL2/SOX9/CCND1/ESR1/NKX2-1/CASP3/SOD1/ICAM1/SRC/PGR/ACVR1B/BAX
GO:0050808	synapse organization	17/149	408/18670	2.91E-08	3.14E-07	1.15E-07	APOE/MAPT/TNF/DRD2/GRIN2B/CTNNB1/SNCA/SHANK3/NRXN1/ACHE/INS/FMR1/IL10/ERBB2/GRIN1/ABL1/SPARC
GO:0021782	glial cell development	10/149	116/18670	3.06E-08	3.29E-07	1.20E-07	MAPT/TNF/TGFB1/S100A8/IL6/EGFR/SOD1/IFNG/AKT1/IL1B
GO:0034612	response to tumor necrosis factor	15/149	312/18670	3.09E-08	3.31E-07	1.21E-07	COL1A1/CASP8/CXCL8/NFE2L2/TNF/PTGS2/STAT1/TRPV1/CASP3/GGT1/MAPK1/ICAM1/AKT1/NFKB1/NFKBIA
GO:0001649	osteoblast differentiation	13/149	225/18670	3.15E-08	3.37E-07	1.23E-07	IGF1/COL1A1/TNF/CTNNB1/ACHE/IL6/IGF2/CAT/AKT1/IGFBP3/SNAI2/NOTCH1/CYP24A1
GO:0002260	lymphocyte homeostasis	8/149	62/18670	3.20E-08	3.40E-07	1.25E-07	BCL2/TGFB1/CASP3/IL2/ABL1/JAK3/AKT1/BAX
GO:0070265	necrotic cell death	8/149	62/18670	3.20E-08	3.40E-07	1.25E-07	FAS/CASP8/TNF/FASLG/SLC25A4/CAV1/TP53/BAX
GO:1903053	regulation of extracellular matrix organization	7/149	41/18670	3.21E-08	3.40E-07	1.25E-07	DPP4/SOX9/TGFB1/IL6/ABL1/RB1/NOTCH1
GO:0002761	regulation of myeloid leukocyte differentiation	10/149	117/18670	3.33E-08	3.52E-07	1.29E-07	CASP8/TNF/CTNNB1/TGFB1/MYC/IFNG/FOS/RB1/PIK3R1/JUN
GO:0006836	neurotransmitter transport	14/149	269/18670	3.40E-08	3.59E-07	1.31E-07	SLC6A2/SLC6A4/NOS1/CACNA1B/DRD2/SNCA/NRXN1/GSK3B/HTR1A/FMR1/MAOB/SLC6A8/DRD4/HTR2A
GO:1900407	regulation of cellular response to oxidative stress	9/149	88/18670	3.44E-08	3.62E-07	1.32E-07	MET/NFE2L2/TNF/CTNNB1/PARP1/INS/IL10/SOD1/AKT1
GO:1904035	regulation of epithelial cell apoptotic process	9/149	88/18670	3.44E-08	3.62E-07	1.32E-07	NFE2L2/TNF/IL13/IL6/FASLG/KDR/HMOX1/ICAM1/ABL1

GO:0050803	regulation of synapse structure or activity	13/149	227/18670	3.50E-08	3.67E-07	1.34E-07	APOE/TNF/DRD2/GRIN2B/SNCA/SHANK3/NRXN1/INS/FMR1/IL10/GRIN1/ABL1/SPARC
GO:0007548	sex differentiation	14/149	270/18670	3.56E-08	3.73E-07	1.36E-07	LEP/BCL2/SOX9/CTNNB1/CCND1/ESR1/NKX2-1/CASP3/SOD1/ICAM1/SRC/PGR/ACVR1B/BAX
GO:2000679	positive regulation of transcription regulatory region DNA binding	6/149	25/18670	3.65E-08	3.81E-07	1.39E-07	IGF1/CTNNB1/TGFB1/PARP1/IFNG/RB1
GO:0030656	regulation of vitamin metabolic process	5/149	13/18670	3.70E-08	3.85E-07	1.41E-07	TNF/IFNG/NFKB1/IL1B/SNAI2
GO:0006869	lipid transport	16/149	365/18670	3.79E-08	3.94E-07	1.44E-07	NOS2/LEP/PLA2G2A/APOE/PON1/PPARG/ABCB1/PLA2G6/DRD2/CAV1/DRD4/CFTR/AKT1/NFKB1/IL1B/NFKBIA
GO:0001890	placenta development	11/149	152/18670	3.81E-08	3.95E-07	1.45E-07	LEP/CASP8/PPARG/PTGS2/IL10/MAPK1/IGF2/EGFR/NODAL/SOD1/AKT1
GO:0010594	regulation of endothelial cell migration	13/149	229/18670	3.88E-08	4.02E-07	1.47E-07	MET/APOE/PPARG/NFE2L2/PTGS2/TGFB1/KDR/HMOX1/FGF2/ABL1/AKT1/SPARC/NOTCH1
GO:0007173	epidermal growth factor receptor signaling pathway	10/149	119/18670	3.92E-08	4.04E-07	1.48E-07	ADRA2A/SOX9/TGFB1/FASLG/EGFR/ABL1/SRC/AKT1/PIK3R1/MMP9
GO:0050729	positive regulation of inflammatory response	11/149	153/18670	4.08E-08	4.19E-07	1.53E-07	PLA2G2A/TNF/PTGS2/SNCA/S100A8/IL6/EGFR/IL2/IL1B/F12/NFKBIA
GO:0009743	response to carbohydrate	13/149	230/18670	4.09E-08	4.19E-07	1.53E-07	LEP/ADRA2A/OPRK1/PLA2G6/PTGS2/TGFB1/CASP3/HMGCR/ICAM1/CFTR/CAT/IL1B/SPARC
GO:0043542	endothelial cell migration	14/149	273/18670	4.09E-08	4.19E-07	1.53E-07	MET/DPP4/APOE/PPARG/NFE2L2/PTGS2/TGFB1/KDR/HMOX1/FGF2/ABL1/AKT1/SPARC/NOTCH1
GO:0042445	hormone metabolic process	13/149	232/18670	4.53E-08	4.63E-07	1.69E-07	LEP/CYP1A1/DUOX2/TNF/ADH1C/ESR1/ACHE/IFNG/TPO/IYD/NFKB1/IL1B/CYP3A4
GO:0032881	regulation of polysaccharide metabolic process	7/149	43/18670	4.53E-08	4.63E-07	1.69E-07	IGF1/TGFB1/GSK3B/INS/IGF2/AKT1/NFKB1
GO:0045639	positive regulation of myeloid cell differentiation	9/149	91/18670	4.63E-08	4.71E-07	1.72E-07	CASP8/TNF/TGFB1/STAT1/IFNG/FOS/RB1/ACVR1B/JUN
GO:0072577	endothelial cell apoptotic process	8/149	65/18670	4.68E-08	4.75E-07	1.74E-07	NFE2L2/TNF/IL13/FASLG/KDR/IL10/ICAM1/ABL1

GO:1905477	positive regulation of protein localization to membrane	10/149	122/18670	4.97E-08	5.04E-07	1.84E-07	BCL2/CASP8/TNF/TGFB1/EGFR/ERBB2/IFNG/TP53/AKT1/PIK3R1
GO:0043535	regulation of blood vessel endothelial cell migration	11/149	156/18670	4.99E-08	5.04E-07	1.84E-07	APOE/PPARG/NFE2L2/PTGS2/TGFB1/KDR/HMOX1/FGF2/ABL1/AKT1/NOTCH1
GO:0071453	cellular response to oxygen levels	13/149	234/18670	5.01E-08	5.05E-07	1.85E-07	FAS/BCL2/PPARG/NFE2L2/PTGS2/MYC/HMOX1/ICAM1/CAV1/SRC/TP53/AKT1/NOTCH1
GO:0045833	negative regulation of lipid metabolic process	9/149	92/18670	5.09E-08	5.13E-07	1.88E-07	APOE/ADRA2A/TNF/INS/SOD1/AKT1/NFKB1/IL1B/SNAI2
GO:0014002	astrocyte development	7/149	44/18670	5.36E-08	5.37E-07	1.96E-07	MAPT/TNF/S100A8/IL6/EGFR/IFNG/IL1B
GO:0070266	necroptotic process	7/149	44/18670	5.36E-08	5.37E-07	1.96E-07	FAS/CASP8/TNF/FASLG/SLC25A4/CAV1/TP53
GO:0035249	synaptic transmission, glutamatergic	9/149	93/18670	5.60E-08	5.59E-07	2.05E-07	PLA2G6/TNF/DRD2/PTGS2/SHANK3/NRXN1/EGFR/GRIN1/HTR2A
GO:0036473	cell death in response to oxidative stress	9/149	93/18670	5.60E-08	5.59E-07	2.05E-07	MET/BCL2/NFE2L2/CTNNB1/PARP1/INS/IL10/SOD1/AKT1
GO:0043467	regulation of generation of precursor metabolites and energy	11/149	158/18670	5.68E-08	5.66E-07	2.07E-07	IGF1/NOS2/PRDM16/SNCA/GSK3B/INS/IGF2/IFNG/TP53/AKT1/HTR2A
GO:0048568	embryonic organ development	17/149	428/18670	5.83E-08	5.79E-07	2.12E-07	CASP8/CXCL8/SOX9/TNF/CTNNB1/TGFB1/KDR/IL10/MAPK1/IGF2/EGFR/NODAL/SOD1/TPO/TP53/AKT1/NOTCH1
GO:0030100	regulation of endocytosis	14/149	281/18670	5.86E-08	5.81E-07	2.13E-07	APOE/PPARG/TNF/DRD2/SNCA/TGFB1/FMR1/SOD1/IFNG/CAV1/ABL1/DRD4/SRC/IL1B
GO:0048143	astrocyte activation	6/149	27/18670	6.02E-08	5.94E-07	2.17E-07	MAPT/TNF/IL6/EGFR/IFNG/IL1B
GO:0098810	neurotransmitter reuptake	6/149	27/18670	6.02E-08	5.94E-07	2.17E-07	SLC6A2/SLC6A4/NOS1/DRD2/SNCA/DRD4
GO:0032309	icosanoid secretion	7/149	45/18670	6.30E-08	6.20E-07	2.27E-07	NOS2/LEP/PLA2G2A/PLA2G6/DRD2/DRD4/IL1B
GO:0007626	locomotory behavior	12/149	198/18670	6.47E-08	6.35E-07	2.32E-07	DPP4/ALK/APOE/OPRK1/CACNA1B/DRD2/SNCA/NKX2-1/SOD1/GRIN1/DRD4/AVP
GO:0030217	T cell differentiation	13/149	240/18670	6.74E-08	6.55E-07	2.40E-07	IRF1/LEP/BCL2/CTNNB1/TGFB1/IL6/SOD1/IL2/ERBB2/IFNG/ABL1/TP53/JAK3
GO:0050663	cytokine secretion	13/149	240/18670	6.74E-08	6.55E-07	2.40E-07	NOS2/LEP/CRP/TNF/IL1A/DRD2/INS/IL10/IFNG/ABL1/SRC/IL1B/NOTCH1

GO:0042116	macrophage activation	9/149	95/18670	6.75E-08	6.55E-07	2.40E-07	MAPT/TNF/SNCA/IL13/IL6/TRPV1/IL10/IFNG/JUN
GO:0044070	regulation of anion transport	9/149	95/18670	6.75E-08	6.55E-07	2.40E-07	LEP/APOE/ABCB1/PLA2G6/SNCA/CFTR/AKT1/IL1B/AVP
GO:0050810	regulation of steroid biosynthetic process	9/149	95/18670	6.75E-08	6.55E-07	2.40E-07	LEP/APOE/TNF/HMGCR/SOD1/IFNG/NFKB1/IL1B/SNAI2
GO:0051952	regulation of amine transport	9/149	95/18670	6.75E-08	6.55E-07	2.40E-07	LEP/ADRA2A/OPRK1/DRD2/P2RY12/SNCA/DRD4/HTR2A/AVP
GO:0017038	protein import	12/149	199/18670	6.84E-08	6.61E-07	2.42E-07	LEP/APOE/PTGS2/TGFB1/HSP90AA1/MAPK1/IFNG/TP53/CDKN1A/AKT1/PIK3R1/NFKBIA
GO:0010883	regulation of lipid storage	7/149	46/18670	7.38E-08	7.13E-07	2.61E-07	LEP/PPARG/CRP/TNF/IL6/NFKB1/NFKBIA
GO:0008585	female gonad development	9/149	96/18670	7.40E-08	7.13E-07	2.61E-07	LEP/BCL2/ESR1/CASP3/SOD1/ICAM1/SRC/PGR/BAX
GO:0032770	positive regulation of monooxygenase activity	6/149	28/18670	7.61E-08	7.32E-07	2.68E-07	APOE/TNF/INS/IFNG/AKT1/IL1B
GO:0010595	positive regulation of endothelial cell migration	10/149	128/18670	7.86E-08	7.53E-07	2.76E-07	MET/NFE2L2/PTGS2/TGFB1/KDR/HMOX1/FGF2/ABL1/AKT1/SPARC
GO:0043255	regulation of carbohydrate biosynthetic process	9/149	97/18670	8.10E-08	7.74E-07	2.83E-07	IGF1/LEP/SNCA/TGFB1/GSK3B/INS/IGF2/AKT1/NFKB1
GO:1902882	regulation of response to oxidative stress	9/149	97/18670	8.10E-08	7.74E-07	2.83E-07	MET/NFE2L2/TNF/CTNNB1/PARP1/INS/IL10/SOD1/AKT1
GO:0007162	negative regulation of cell adhesion	14/149	289/18670	8.30E-08	7.91E-07	2.89E-07	IRF1/COL1A1/TGFB1/CASP3/IL10/IL2/ERBB2/ABL1/SRC/JAK3/AKT1/PIK3R1/SNAI2/NOTCH1
GO:0051966	regulation of synaptic transmission, glutamatergic	8/149	70/18670	8.47E-08	8.05E-07	2.95E-07	PLA2G6/TNF/DRD2/PTGS2/SHANK3/NRXN1/EGFR/HTR2A
GO:0035635	entry of bacterium into host cell	5/149	15/18670	8.52E-08	8.05E-07	2.95E-07	MET/CXCL8/CTNNB1/CAV1/SRC
GO:0045073	regulation of chemokine biosynthetic process	5/149	15/18670	8.52E-08	8.05E-07	2.95E-07	TNF/IL6/HMOX1/IFNG/IL1B
GO:0051044	positive regulation of membrane protein ectodomain proteolysis	5/149	15/18670	8.52E-08	8.05E-07	2.95E-07	APOE/ADRA2A/TNF/IFNG/IL1B

GO:0061614	pri-miRNA transcription by RNA polymerase II	7/149	47/18670	8.62E-08	8.09E-07	2.96E-07	PPARG/SOX9/TGFB1/IL10/FOS/TP53/JUN
GO:0070849	response to epidermal growth factor	7/149	47/18670	8.62E-08	8.09E-07	2.96E-07	COL1A1/SOX9/MAPK1/EGFR/ERBB2/AKT1/SNAI2
GO:2001239	regulation of extrinsic apoptotic signaling pathway in absence of ligand	7/149	47/18670	8.62E-08	8.09E-07	2.96E-07	BCL2/RET/TNF/IL1A/AKT1/IL1B/SNAI2
GO:0006914	autophagy	18/149	496/18670	9.05E-08	8.46E-07	3.10E-07	MET/LEP/BCL2/MAPT/DRD2/SNCA/S100A8/GSK3B/HSP90AA1/CASP3/KDR/HMOX1/IL10/IFNG/ABL1/SRC/TP53/AKT1
GO:0061919	process utilizing autophagic mechanism	18/149	496/18670	9.05E-08	8.46E-07	3.10E-07	MET/LEP/BCL2/MAPT/DRD2/SNCA/S100A8/GSK3B/HSP90AA1/CASP3/KDR/HMOX1/IL10/IFNG/ABL1/SRC/TP53/AKT1
GO:0050671	positive regulation of lymphocyte proliferation	10/149	130/18670	9.10E-08	8.49E-07	3.11E-07	IGF1/LEP/BCL2/IL13/IL6/IGF2/IL2/CDKN1A/JAK3/IL1B
GO:0010575	positive regulation of vascular endothelial growth factor production	6/149	29/18670	9.53E-08	8.88E-07	3.25E-07	IL1A/PTGS2/TGFB1/IL6/NODAL/IL1B
GO:0032946	positive regulation of mononuclear cell proliferation	10/149	131/18670	9.79E-08	9.09E-07	3.33E-07	IGF1/LEP/BCL2/IL13/IL6/IGF2/IL2/CDKN1A/JAK3/IL1B
GO:0007599	hemostasis	15/149	341/18670	9.90E-08	9.18E-07	3.36E-07	PLAU/IRF1/COL1A1/APOE/ADRA2A/NFE2L2/F2/P2RY12/AVPR2/IL6/MAPK1/CAV1/SRC/PIK3R1/F12
GO:0051972	regulation of telomerase activity	7/149	48/18670	1.00E-07	9.25E-07	3.39E-07	PPARG/CTNNB1/MYC/HSP90AA1/MAPK1/SRC/TP53
GO:0090199	regulation of release of cytochrome c from mitochondria	7/149	48/18670	1.00E-07	9.25E-07	3.39E-07	IGF1/PLA2G6/TP53/AKT1/BAX/AVP/MMP9
GO:0033044	regulation of chromosome organization	15/149	342/18670	1.03E-07	9.48E-07	3.47E-07	NOS1/MAPT/CTNNB1/SNCA/TGFB1/MYC/PARP1/FMR1/MAPK1/IGF2/RB1/SRC/TP53/IL1B/SNAI2
GO:0006352	DNA-templated transcription, initiation	13/149	249/18670	1.04E-07	9.50E-07	3.48E-07	THRB/PPARG/SOX9/CTNNB1/CCND1/ESR1/PGR/THRA/TP53/CDKN1A/JUN/NOTCH1/BAX
GO:0071560	cellular response to transforming growth factor beta stimulus	13/149	249/18670	1.04E-07	9.50E-07	3.48E-07	COL1A1/PRDM16/SOX9/TGFB1/NKX2-1/PARP1/NODAL/FOS/CAV1/SRC/ACVR1B/TP53/JUN
GO:0002064	epithelial cell development	12/149	207/18670	1.05E-07	9.62E-07	3.52E-07	MET/SOX9/TNF/CTNNB1/ESR1/GSK3B/SOD1/ICAM1/PGR/CDKN1A/IL1B/NOTCH1

GO:0034284	response to monosaccharide	12/149	207/18670	1.05E-07	9.62E-07	3.52E-07	LEP/ADRA2A/OPRK1/PLA2G6/PTGS2/TGFB1/CASP3/HMGCR/ICAM1/CFTR/CAT/SPARC
GO:0060079	excitatory postsynaptic potential	9/149	100/18670	1.06E-07	9.64E-07	3.53E-07	DRD2/GRIN2B/SNCA/SHANK3/NRXN1/GSK3B/TRPV1/GRIN1/AKT1
GO:0061180	mammary gland epithelium development	8/149	72/18670	1.06E-07	9.64E-07	3.53E-07	CCND1/ESR1/TGFB1/MAPK1/SRC/PGR/AKT1/BAX
GO:0030307	positive regulation of cell growth	11/149	169/18670	1.13E-07	1.03E-06	3.76E-07	IGF1/BCL2/MAPT/F2/S100A8/INS/EGFR/IL2/ERBB2/AKT1/AVP
GO:0021537	telencephalon development	13/149	251/18670	1.14E-07	1.03E-06	3.76E-07	ALK/DRD2/CTNNB1/P2RY12/SHANK3/AVPR2/NKX2-1/GSK3B/CASP3/EGFR/GRIN1/BAX/SCN5A
GO:0045637	regulation of myeloid cell differentiation	13/149	251/18670	1.14E-07	1.03E-06	3.76E-07	CASP8/TNF/CTNNB1/TGFB1/MYC/STAT1/IFNG/FOS/RB1/ACVR1B/PIK3R1/NFKBIA/JUN
GO:0071715	icosanoid transport	7/149	49/18670	1.16E-07	1.04E-06	3.82E-07	NOS2/LEP/PLA2G2A/PLA2G6/DRD2/DRD4/IL1B
GO:1901571	fatty acid derivative transport	7/149	49/18670	1.16E-07	1.04E-06	3.82E-07	NOS2/LEP/PLA2G2A/PLA2G6/DRD2/DRD4/IL1B
GO:1904707	positive regulation of vascular smooth muscle cell proliferation	7/149	49/18670	1.16E-07	1.04E-06	3.82E-07	MMP2/IGF1/TNF/IL10/FGF2/JUN/MMP9
GO:0071229	cellular response to acid chemical	12/149	209/18670	1.17E-07	1.05E-06	3.84E-07	MMP2/SLC6A4/LEP/COL1A1/PPARG/RET/SOX9/TNF/KDR/EGFR/SRC/AKT1
GO:0031100	animal organ regeneration	8/149	73/18670	1.18E-07	1.06E-06	3.87E-07	PPARG/CCND1/TGFB1/HMOX1/IL10/EGFR/CDKN1A/NOTCH1
GO:0006837	serotonin transport	5/149	16/18670	1.23E-07	1.09E-06	4.01E-07	SLC6A4/NOS1/SNCA/HTR1A/MAOB
GO:0032225	regulation of synaptic transmission, dopaminergic	5/149	16/18670	1.23E-07	1.09E-06	4.01E-07	SLC6A4/DRD2/PTGS2/SNCA/DRD4
GO:0042033	chemokine biosynthetic process	5/149	16/18670	1.23E-07	1.09E-06	4.01E-07	TNF/IL6/HMOX1/IFNG/IL1B
GO:0050755	chemokine metabolic process	5/149	16/18670	1.23E-07	1.09E-06	4.01E-07	TNF/IL6/HMOX1/IFNG/IL1B
GO:2000027	regulation of animal organ morphogenesis	13/149	253/18670	1.25E-07	1.11E-06	4.04E-07	BCL2/THRB/SOX9/TNF/CTNNB1/ESR1/TGFB1/MYC/STAT1/ABL1/SNAI2/NOTCH1/BAX
GO:0015837	amine transport	9/149	102/18670	1.26E-07	1.11E-06	4.07E-07	LEP/ADRA2A/OPRK1/DRD2/P2RY12/SNCA/DRD4/HTR2A/AVP

GO:0009409	response to cold	7/149	50/18670	1.34E-07	1.19E-06	4.34E-07	CASP8/PPARG/TRPA1/HSP90AA1/FOS/THRA/NFKBIA
GO:0071559	response to transforming growth factor beta	13/149	255/18670	1.37E-07	1.20E-06	4.40E-07	COL1A1/PRDM16/SOX9/TGFB1/NKX2-1/PARP1/NODAL/FOS/CAV1/SRC/ACVR1B/TP53/JUN
GO:0001963	synaptic transmission, dopaminergic	6/149	31/18670	1.46E-07	1.28E-06	4.70E-07	SLC6A2/SLC6A4/DRD2/PTGS2/SNCA/DRD4
GO:1903201	regulation of oxidative stress-induced cell death	8/149	75/18670	1.46E-07	1.28E-06	4.70E-07	MET/NFE2L2/CTNNB1/PARP1/INS/IL10/SOD1/AKT1
GO:0019233	sensory perception of pain	9/149	104/18670	1.49E-07	1.30E-06	4.76E-07	OPRK1/TRPA1/PTGS2/TRPV1/IL10/MAPK1/GRIN1/HTR2A/SCN10A
GO:0046822	regulation of nucleocytoplasmic transport	9/149	104/18670	1.49E-07	1.30E-06	4.76E-07	LEP/PTGS2/TGFB1/GSK3B/MAPK1/IFNG/TP53/PIK3R1/IL1B
GO:0045732	positive regulation of protein catabolic process	12/149	214/18670	1.51E-07	1.32E-06	4.83E-07	APOE/ADRA2A/NFE2L2/SOX9/TNF/GSK3B/HSP90AA1/FMR1/IFNG/CAV1/AKT1/IL1B
GO:0007566	embryo implantation	7/149	51/18670	1.54E-07	1.35E-06	4.92E-07	MMP2/PTGS2/NODAL/SOD1/ACVR1B/IL1B/MMP9
GO:0090257	regulation of muscle system process	13/149	259/18670	1.63E-07	1.42E-06	5.20E-07	IGF1/NOS1/ADRA2A/PTGS2/KCNQ1/PARP1/CACNA1C/SOD1/CAV1/CHRM3/SCN10A/NOTCH1/SCN5A
GO:0051588	regulation of neurotransmitter transport	10/149	139/18670	1.71E-07	1.48E-06	5.43E-07	SLC6A4/NOS1/DRD2/SNCA/GSK3B/HTR1A/FMR1/MAOB/DRD4/HTR2A
GO:0070665	positive regulation of leukocyte proliferation	10/149	139/18670	1.71E-07	1.48E-06	5.43E-07	IGF1/LEP/BCL2/IL13/IL6/IGF2/IL2/CDKN1A/JAK3/IL1B
GO:0031649	heat generation	5/149	17/18670	1.73E-07	1.50E-06	5.47E-07	TNF/IL1A/PTGS2/TRPV1/IL1B
GO:2000811	negative regulation of anoikis	5/149	17/18670	1.73E-07	1.50E-06	5.47E-07	BCL2/CAV1/SRC/SNAI2/NOTCH1
GO:0046394	carboxylic acid biosynthetic process	17/149	462/18670	1.74E-07	1.50E-06	5.48E-07	IGF1/CYP1A1/PTGS2/CYP2E1/TGFB1/GGT1/INS/IFNG/TP53/NFKB1/IL1B/PTGS1/HTR2A/GATM/CYP3A4/PAH/AVP
GO:0008406	gonad development	12/149	217/18670	1.76E-07	1.51E-06	5.52E-07	LEP/BCL2/SOX9/CCND1/ESR1/NKX2-1/CASP3/SOD1/ICAM1/SRC/PGR/BAX
GO:0017157	regulation of exocytosis	12/149	217/18670	1.76E-07	1.51E-06	5.52E-07	ADRA2A/PLA2G6/DRD2/SNCA/IL13/GSK3B/HMOX1/FMR1/IFNG/CFTR/HTR2A/NOTCH1
GO:0051385	response to mineralocorticoid	6/149	32/18670	1.78E-07	1.53E-06	5.59E-07	CCND1/PARP1/MAOB/FOS/SRC/CDKN1A

GO:0016053	organic acid biosynthetic process	17/149	463/18670	1.79E-07	1.53E-06	5.61E-07	IGF1/CYP1A1/PTGS2/CYP2E1/TGFB1/GGT1/INS/IFNG/TP53/NFKB1/IL1B/PTGS1/HTR2A/GATM/CYP3A4/PAH/AVP
GO:0051937	catecholamine transport	8/149	77/18670	1.80E-07	1.54E-06	5.62E-07	SLC6A2/ADRA2A/OPRK1/DRD2/P2RY12/SNCA/DRD4/HTR2A
GO:0001837	epithelial to mesenchymal transition	10/149	140/18670	1.83E-07	1.56E-06	5.69E-07	COL1A1/SOX9/CTNNB1/TGFB1/NKX2-1/GSK3B/IL6/IL1B/SNAI2/NOTCH1
GO:0072073	kidney epithelium development	10/149	140/18670	1.83E-07	1.56E-06	5.69E-07	BCL2/RET/SOX9/CTNNB1/TGFB1/MYC/STAT1/FGF2/CAT/NOTCH1
GO:0019722	calcium-mediated signaling	12/149	218/18670	1.85E-07	1.57E-06	5.74E-07	IGF1/NOS1/MAPT/CXCL8/TNF/GRIN2B/P2RY12/GSK3B/CACNA1C/KDR/EGFR/GRIN1
GO:2001257	regulation of cation channel activity	11/149	178/18670	1.92E-07	1.62E-06	5.94E-07	NOS1/PLA2G6/DRD2/GRIN2B/SHANK3/FMR1/GRIN1/IFNG/CAV1/DRD4/MMP9
GO:0010827	regulation of glucose transmembrane transport	8/149	78/18670	1.99E-07	1.68E-06	6.16E-07	IGF1/LEP/NFE2L2/TNF/INS/AKT1/PIK3R1/IL1B
GO:0045840	positive regulation of mitotic nuclear division	7/149	53/18670	2.03E-07	1.71E-06	6.26E-07	IGF1/IL1A/TGFB1/INS/IGF2/RB1/IL1B
GO:2000278	regulation of DNA biosynthetic process	9/149	108/18670	2.06E-07	1.73E-06	6.34E-07	PPARG/CTNNB1/MYC/HSP90AA1/MAPK1/FGF2/SRC/TP53/CDKN1A
GO:0007409	axonogenesis	17/149	468/18670	2.09E-07	1.75E-06	6.42E-07	APOE/BCL2/RET/MAPT/DRD2/SHANK3/NRXN1/NKX2-1/GSK3B/HSP90AA1/MAPK1/ERBB2/GRIN1/ABL1/SRC/PIK3R1/NOTCH1
GO:0010675	regulation of cellular carbohydrate metabolic process	10/149	142/18670	2.09E-07	1.75E-06	6.42E-07	IGF1/LEP/SNCA/GSK3B/INS/IGF2/SRC/TP53/AKT1/IGFBP3
GO:0043534	blood vessel endothelial cell migration	11/149	180/18670	2.15E-07	1.80E-06	6.58E-07	APOE/PPARG/NFE2L2/PTGS2/TGFB1/KDR/HMOX1/FGF2/ABL1/AKT1/NOTCH1
GO:0010039	response to iron ion	6/149	33/18670	2.17E-07	1.81E-06	6.61E-07	BCL2/CYP1A1/DRD2/CCND1/SNCA/HMOX1
GO:0051194	positive regulation of cofactor metabolic process	6/149	33/18670	2.17E-07	1.81E-06	6.61E-07	IGF1/NFE2L2/SNCA/INS/IFNG/HTR2A
GO:0043536	positive regulation of blood vessel endothelial cell migration	8/149	79/18670	2.20E-07	1.83E-06	6.71E-07	NFE2L2/PTGS2/TGFB1/KDR/HMOX1/FGF2/ABL1/AKT1
GO:0006606	protein import into nucleus	10/149	143/18670	2.23E-07	1.85E-06	6.78E-07	LEP/PTGS2/TGFB1/MAPK1/IFNG/TP53/CDKN1A/AKT1/PIK3R1/NFKBIA

GO:0030879	mammary gland development	10/149	143/18670	2.23E-07	1.85E-06	6.78E-07	SOX9/CCND1/ESR1/TGFB1/MAPK1/CAV1/SRC/PGR/AKT1/BAX
GO:0002763	positive regulation of myeloid leukocyte differentiation	7/149	54/18670	2.32E-07	1.91E-06	7.00E-07	CASP8/TNF/TGFB1/IFNG/FOS/RB1/JUN
GO:0010524	positive regulation of calcium ion transport into cytosol	7/149	54/18670	2.32E-07	1.91E-06	7.00E-07	F2/SNCA/IL13/GRIN1/CAV1/ABL1/BAX
GO:0045444	fat cell differentiation	12/149	223/18670	2.36E-07	1.95E-06	7.13E-07	LEP/PRDM16/PPARG/TNF/PTGS2/CCND1/TGFB1/IL6/INS/AKT1/HTR2A/SNAI2
GO:0030534	adult behavior	10/149	144/18670	2.38E-07	1.96E-06	7.18E-07	LEP/ALK/OPRK1/DRD2/SNCA/SHANK3/NRXN1/GRIN1/DRD4/HTR2A
GO:0007612	learning	10/149	145/18670	2.54E-07	2.09E-06	7.65E-07	BCHE/OPRK1/DRD2/PTGS2/SHANK3/NRXN1/HMGCR/GRIN1/FOS/JUN
GO:0010574	regulation of vascular endothelial growth factor production	6/149	34/18670	2.61E-07	2.14E-06	7.83E-07	IL1A/PTGS2/TGFB1/IL6/NODAL/IL1B
GO:0043276	anoikis	6/149	34/18670	2.61E-07	2.14E-06	7.83E-07	BCL2/CAV1/SRC/AKT1/SNAI2/NOTCH1
GO:1902930	regulation of alcohol biosynthetic process	8/149	81/18670	2.68E-07	2.19E-06	8.01E-07	APOE/TNF/SNCA/HMGCR/SOD1/IFNG/NFKB1/IL1B
GO:0006909	phagocytosis	15/149	369/18670	2.74E-07	2.23E-06	8.18E-07	MET/LEP/PPARG/CRP/PLA2G6/TNF/TGFB1/HSP90AA1/MAPK1/SOD1/IFNG/ABL1/SRC/PIK3R1/IL1B
GO:0032386	regulation of intracellular transport	16/149	423/18670	2.84E-07	2.31E-06	8.46E-07	LEP/DRD2/PTGS2/TGFB1/IL13/GSK3B/HMOX1/FMR1/MAPK1/ERBB2/IFNG/SRC/TP53/PIK3R1/IL1B/HTR2A
GO:0002262	myeloid cell homeostasis	10/149	147/18670	2.89E-07	2.35E-06	8.60E-07	STAT1/IL6/CASP3/HMOX1/SOD1/RB1/THRA/ACVR1B/JAK3/BAX
GO:0051279	regulation of release of sequestered calcium ion into cytosol	8/149	82/18670	2.95E-07	2.39E-06	8.76E-07	NOS1/F2/SNCA/TGFB1/IL13/CACNA1C/ABL1/BAX
GO:0060688	regulation of morphogenesis of a branching structure	7/149	56/18670	2.99E-07	2.42E-06	8.85E-07	SOX9/TNF/CTNNB1/ESR1/TGFB1/ABL1/SNAI2
GO:0090183	regulation of kidney development	7/149	56/18670	2.99E-07	2.42E-06	8.85E-07	RET/SOX9/CTNNB1/TGFB1/MYC/STAT1/MMP9

GO:0002792	negative regulation of peptide secretion	10/149	148/18670	3.08E-07	2.48E-06	9.09E-07	LEP/APOE/ADRA2A/TNF/DRD2/HMGCR/INS/IL10/DRD4/IL1B
GO:0110111	negative regulation of animal organ morphogenesis	6/149	35/18670	3.13E-07	2.52E-06	9.23E-07	BCL2/THRB/TNF/CTNNB1/STAT1/NOTCH1
GO:1905475	regulation of protein localization to membrane	11/149	187/18670	3.15E-07	2.53E-06	9.27E-07	BCL2/CASP8/TNF/TGFB1/INS/EGFR/ERBB2/IFNG/TP53/AKT1/PIK3R1
GO:0015711	organic anion transport	17/149	482/18670	3.16E-07	2.54E-06	9.29E-07	NOS2/LEP/PLA2G2A/APOE/PPARG/ABCB1/PLA2G6/DRD2/SNCA/TRPV1/SLC25A4/SLC6A8/DRD4/CFTR/AKT1/IL1B/AVP
GO:0032930	positive regulation of superoxide anion generation	5/149	19/18670	3.22E-07	2.58E-06	9.43E-07	CRP/MAPT/TGFB1/EGFR/SOD1
GO:0042509	regulation of tyrosine phosphorylation of STAT protein	8/149	83/18670	3.24E-07	2.59E-06	9.49E-07	IGF1/LEP/TNF/IL13/IL6/IL2/IFNG/CAV1
GO:0042035	regulation of cytokine biosynthetic process	9/149	114/18670	3.28E-07	2.61E-06	9.56E-07	IRF1/TNF/IL1A/IL6/HMOX1/IL10/IFNG/NFKB1/IL1B
GO:0051092	positive regulation of NF-kappaB transcription factor activity	10/149	149/18670	3.28E-07	2.61E-06	9.56E-07	ALK/TNF/TGFB1/S100A8/INS/ICAM1/CAV1/CAT/NFKB1/IL1B
GO:1904018	positive regulation of vasculature development	12/149	230/18670	3.29E-07	2.62E-06	9.59E-07	CXCL8/NFE2L2/IL1A/PTGS2/KDR/HMOX1/IL10/NODAL/FGF2/ABL1/IL1B/NOTCH1
GO:0006367	transcription initiation from RNA polymerase II promoter	11/149	188/18670	3.32E-07	2.64E-06	9.66E-07	THRB/PPARG/SOX9/CCND1/ESR1/PGR/THRA/TP53/CDKN1A/NOTCH1/BAX
GO:0010507	negative regulation of autophagy	8/149	84/18670	3.56E-07	2.82E-06	1.03E-06	MET/LEP/BCL2/SNCA/HMOX1/IL10/TP53/AKT1
GO:0045445	myoblast differentiation	8/149	84/18670	3.56E-07	2.82E-06	1.03E-06	IGF1/SOX9/TNF/TGFB1/HMGCR/RB1/IGFBP3/NOTCH1
GO:0051188	cofactor biosynthetic process	14/149	326/18670	3.61E-07	2.86E-06	1.04E-06	IGF1/CYP1A1/DUOX2/NFE2L2/PTGS2/SNCA/GGT1/INS/MAOB/SOD1/IFNG/FECH/TP53/HTR2A
GO:0001822	kidney development	13/149	278/18670	3.68E-07	2.90E-06	1.06E-06	BCL2/RET/SOX9/CTNNB1/TGFB1/MYC/STAT1/FGF2/CASP9/CAT/NOTCH1/BAX/MMP9
GO:0016999	antibiotic metabolic process	10/149	151/18670	3.71E-07	2.92E-06	1.07E-06	CYP1A1/DUOX2/MPO/ADH1C/SNCA/MAOB/EGFR/SOD1/TPO/CAT

GO:0010573	vascular endothelial growth factor production	6/149	36/18670	3.73E-07	2.92E-06	1.07E-06	IL1A/PTGS2/TGFB1/IL6/NODAL/IL1B
GO:0042554	superoxide anion generation	6/149	36/18670	3.73E-07	2.92E-06	1.07E-06	DUOX2/CRP/MAPT/TGFB1/EGFR/SOD1
GO:1901099	negative regulation of signal transduction in absence of ligand	6/149	36/18670	3.73E-07	2.92E-06	1.07E-06	BCL2/TNF/IL1A/AKT1/IL1B/SNAI2
GO:2001240	negative regulation of extrinsic apoptotic signaling pathway in absence of ligand	6/149	36/18670	3.73E-07	2.92E-06	1.07E-06	BCL2/TNF/IL1A/AKT1/IL1B/SNAI2
GO:0010506	regulation of autophagy	14/149	327/18670	3.75E-07	2.93E-06	1.07E-06	MET/LEP/BCL2/MAPT/SNCA/GSK3B/CASP3/KDR/HMOX1/IL10/IFNG/ABL1/TP53/AKT1
GO:0071478	cellular response to radiation	11/149	191/18670	3.90E-07	3.03E-06	1.11E-06	PTGS2/TGFB1/MYC/PARP1/FMR1/TP53/CASP9/CDKN1A/PIK3R1/SNAI2/BAX
GO:0007613	memory	9/149	117/18670	4.09E-07	3.17E-06	1.16E-06	SLC6A4/APOE/MAPT/PLA2G6/DRD2/PTGS2/SHANK3/GRIN1/HTR2A
GO:0010906	regulation of glucose metabolic process	9/149	117/18670	4.09E-07	3.17E-06	1.16E-06	IGF1/LEP/GSK3B/INS/IGF2/SRC/TP53/AKT1/IGFBP3
GO:1900371	regulation of purine nucleotide biosynthetic process	9/149	117/18670	4.09E-07	3.17E-06	1.16E-06	IGF1/NOS2/NOS1/SNCA/PARP1/INS/IFNG/TP53/HTR2A
GO:0030900	forebrain development	15/149	381/18670	4.12E-07	3.18E-06	1.17E-06	ALK/DRD2/CTNNB1/P2RY12/SHANK3/AVPR2/NKX2-1/GSK3B/CASP3/EGFR/GRIN1/SRC/NOTCH1/BAX/SCN5A
GO:0046777	protein autophosphorylation	12/149	235/18670	4.15E-07	3.20E-06	1.17E-06	ALK/GSK3B/KDR/INS/EGFR/ERBB2/CAV1/ABL1/SRC/ACVR1B/AKT1/JUN
GO:0001655	urogenital system development	14/149	330/18670	4.19E-07	3.23E-06	1.18E-06	BCL2/RET/SOX9/CTNNB1/ESR1/TGFB1/MYC/STAT1/FGF2/CASP9/CAT/NOTCH1/BAX/MMP9
GO:0090316	positive regulation of intracellular protein transport	10/149	153/18670	4.19E-07	3.23E-06	1.18E-06	LEP/PTGS2/TGFB1/GSK3B/MAPK1/ERBB2/IFNG/TP53/PIK3R1/IL1B
GO:0048148	behavioral response to cocaine	5/149	20/18670	4.26E-07	3.27E-06	1.20E-06	OPRK1/DRD2/SNCA/DRD4/HTR2A
GO:2000351	regulation of endothelial cell apoptotic process	7/149	59/18670	4.32E-07	3.31E-06	1.21E-06	NFE2L2/TNF/IL13/FASLG/KDR/ICAM1/ABL1

GO:0060249	anatomical structure homeostasis	16/149	437/18670	4.38E-07	3.36E-06	1.23E-06	SLC6A2/BCL2/SOX9/CTNNB1/TGFB1/MYC/IL6/HSP90AA1/PARP1/MAPK1/EGFR/SOD1/RB1/SRC/NOTCH1/BAX
GO:0030808	regulation of nucleotide biosynthetic process	9/149	118/18670	4.40E-07	3.36E-06	1.23E-06	IGF1/NOS2/NOS1/SNCA/PARP1/INS/IFNG/TP53/HTR2A
GO:0006631	fatty acid metabolic process	15/149	383/18670	4.40E-07	3.36E-06	1.23E-06	LEP/PON1/CYP1A1/PPARG/PTGS2/CYP2E1/SNCA/GGT1/INS/CAV1/AKT1/IL1B/PTGS1/CYP3A4/AVP
GO:0042307	positive regulation of protein import into nucleus	6/149	37/18670	4.43E-07	3.37E-06	1.23E-06	LEP/PTGS2/TGFB1/MAPK1/IFNG/PIK3R1
GO:1990845	adaptive thermogenesis	10/149	154/18670	4.45E-07	3.39E-06	1.24E-06	LEP/PRDM16/IL13/ACHE/TRPV1/CAV1/RB1/THRA/GATM/NOTCH1
GO:0007050	cell cycle arrest	12/149	237/18670	4.54E-07	3.45E-06	1.26E-06	IRF1/CXCL8/CCND1/TGFB1/MYC/IFNG/ABL1/RB1/TP53/CDKN1A/NOTCH1/BAX
GO:0015849	organic acid transport	14/149	333/18670	4.67E-07	3.53E-06	1.29E-06	NOS2/LEP/PLA2G2A/APOE/PPARG/PLA2G6/DRD2/SNCA/TRPV1/SLC6A8/DRD4/AKT1/IL1B/AVP
GO:0046942	carboxylic acid transport	14/149	333/18670	4.67E-07	3.53E-06	1.29E-06	NOS2/LEP/PLA2G2A/APOE/PPARG/PLA2G6/DRD2/SNCA/TRPV1/SLC6A8/DRD4/AKT1/IL1B/AVP
GO:0045844	positive regulation of striated muscle tissue development	8/149	87/18670	4.68E-07	3.53E-06	1.29E-06	IGF1/BCL2/CTNNB1/TGFB1/HMGCR/MAPK1/FGF2/NOTCH1
GO:0048636	positive regulation of muscle organ development	8/149	87/18670	4.68E-07	3.53E-06	1.29E-06	IGF1/BCL2/CTNNB1/TGFB1/HMGCR/MAPK1/FGF2/NOTCH1
GO:0021700	developmental maturation	13/149	284/18670	4.69E-07	3.53E-06	1.29E-06	MMP2/IGF1/LEP/BCL2/PPARG/RET/CTNNB1/NRXN1/GRIN1/RB1/CFTR/PGR/CDKN1A
GO:1903707	negative regulation of hemopoiesis	10/149	155/18670	4.73E-07	3.55E-06	1.30E-06	IRF1/NFE2L2/CTNNB1/MYC/IL2/ERBB2/JAK3/PIK3R1/NFKBIA/NOTCH1
GO:0046324	regulation of glucose import	7/149	60/18670	4.85E-07	3.64E-06	1.33E-06	IGF1/LEP/NFE2L2/TNF/INS/AKT1/PIK3R1
GO:0015980	energy derivation by oxidation of organic compounds	13/149	285/18670	4.88E-07	3.66E-06	1.34E-06	IGF1/NOS2/LEP/PRDM16/SNCA/MYC/GSK3B/INS/IGF2/IFNG/TP53/CAT/AKT1
GO:0006694	steroid biosynthetic process	11/149	196/18670	5.04E-07	3.77E-06	1.38E-06	LEP/APOE/TNF/HMGCR/SOD1/IFNG/CFTR/NFKB1/IL1B/SNAI2/CYP3A4
GO:0034103	regulation of tissue remodeling	8/149	88/18670	5.11E-07	3.81E-06	1.39E-06	LEP/TGFB1/IL6/EGFR/IL2/SRC/TP53/BAX

GO:1901863	positive regulation of muscle tissue development	8/149	88/18670	5.11E-07	3.81E-06	1.39E-06	IGF1/BCL2/CTNNB1/TGFB1/HMGCR/MAPK1/FGF2/NOTCH1
GO:0007596	blood coagulation	14/149	336/18670	5.20E-07	3.87E-06	1.42E-06	PLAU/IRF1/COL1A1/APOE/ADRA2A/NFE2L2/F2/P2RY12/IL6/MAPK1/CAV1/SRC/PIK3R1/F12
GO:0032602	chemokine production	8/149	89/18670	5.58E-07	4.14E-06	1.51E-06	TNF/S100A8/IL6/HMOX1/IL10/IFNG/IL1B/SNAI2
GO:0031099	regeneration	11/149	198/18670	5.58E-07	4.14E-06	1.51E-06	IGF1/BCL2/PPARG/CCND1/TGFB1/HMOX1/IL10/EGFR/CDKN1A/JUN/NOTCH1
GO:0007179	transforming growth factor beta receptor signaling pathway	11/149	199/18670	5.86E-07	4.34E-06	1.59E-06	PRDM16/TGFB1/NKX2-1/PARP1/NODAL/FOS/CAV1/SRC/ACVR1B/TP53/JUN
GO:1904591	positive regulation of protein import	6/149	39/18670	6.13E-07	4.53E-06	1.66E-06	LEP/PTGS2/TGFB1/MAPK1/IFNG/PIK3R1
GO:0042089	cytokine biosynthetic process	9/149	123/18670	6.26E-07	4.62E-06	1.69E-06	IRF1/TNF/IL1A/IL6/HMOX1/IL10/IFNG/NFKB1/IL1B
GO:0050817	coagulation	14/149	342/18670	6.43E-07	4.73E-06	1.73E-06	PLAU/IRF1/COL1A1/APOE/ADRA2A/NFE2L2/F2/P2RY12/IL6/MAPK1/CAV1/SRC/PIK3R1/F12
GO:0072001	renal system development	13/149	293/18670	6.68E-07	4.91E-06	1.80E-06	BCL2/RET/SOX9/CTNNB1/TGFB1/MYC/STAT1/FGF2/CASP9/CAT/NOTCH1/BAX/MMP9
GO:0014013	regulation of gliogenesis	9/149	124/18670	6.70E-07	4.91E-06	1.80E-06	PPARG/TNF/F2/CTNNB1/P2RY12/TGFB1/IL6/IL1B/NOTCH1
GO:0019218	regulation of steroid metabolic process	9/149	124/18670	6.70E-07	4.91E-06	1.80E-06	LEP/APOE/TNF/HMGCR/SOD1/IFNG/NFKB1/IL1B/SNAI2
GO:0042107	cytokine metabolic process	9/149	124/18670	6.70E-07	4.91E-06	1.80E-06	IRF1/TNF/IL1A/IL6/HMOX1/IL10/IFNG/NFKB1/IL1B
GO:0015718	monocarboxylic acid transport	10/149	162/18670	7.11E-07	5.19E-06	1.90E-06	NOS2/LEP/PLA2G2A/APOE/PPARG/PLA2G6/DRD2/DRD4/AKT1/IL1B
GO:0032928	regulation of superoxide anion generation	5/149	22/18670	7.14E-07	5.20E-06	1.90E-06	CRP/MAPT/TGFB1/EGFR/SOD1
GO:0060487	lung epithelial cell differentiation	5/149	22/18670	7.14E-07	5.20E-06	1.90E-06	THRB/SOX9/NKX2-1/IL13/THRA
GO:0050768	negative regulation of neurogenesis	13/149	295/18670	7.21E-07	5.24E-06	1.92E-06	SLC6A4/APOE/THRB/SOX9/TNF/F2/CTNNB1/TGFB1/GSK3B/IL6/TP53/IL1B/NOTCH1
GO:0035265	organ growth	11/149	204/18670	7.50E-07	5.43E-06	1.99E-06	IGF1/SLC6A4/LEP/BCL2/SOX9/ESR1/MAPK1/SOD1/FGF2/AKT1/NOTCH1

GO:0035637	multicellular organismal signaling	11/149	204/18670	7.50E-07	5.43E-06	1.99E-06	NOS1/KCNQ1/ABCC9/CACNA1C/FMR1/SOD1/CAV1/CACNA1S/SCN10A/SCN5A/AVP
GO:0045766	positive regulation of angiogenesis	11/149	204/18670	7.50E-07	5.43E-06	1.99E-06	CXCL8/NFE2L2/IL1A/PTGS2/KDR/HMOX1/IL10/NODAL/FGF2/ABL1/IL1B
GO:0051170	import into nucleus	10/149	163/18670	7.52E-07	5.43E-06	1.99E-06	LEP/PTGS2/TGFB1/MAPK1/IFNG/TP53/CDKN1A/AKT1/PIK3R1/NFKBIA
GO:0043550	regulation of lipid kinase activity	7/149	64/18670	7.60E-07	5.46E-06	2.00E-06	F2/P2RY12/TGFB1/FGF2/RB1/SRC/PIK3R1
GO:0045600	positive regulation of fat cell differentiation	7/149	64/18670	7.60E-07	5.46E-06	2.00E-06	PRDM16/PPARG/PTGS2/INS/AKT1/HTR2A/SNAI2
GO:0060135	maternal process involved in female pregnancy	7/149	64/18670	7.60E-07	5.46E-06	2.00E-06	PLA2G6/PTGS2/ESR1/MAPK1/NODAL/PGR/AKT1
GO:0072080	nephron tubule development	8/149	93/18670	7.83E-07	5.61E-06	2.05E-06	BCL2/SOX9/CTNNB1/TGFB1/MYC/STAT1/FGF2/NOTCH1
GO:1901655	cellular response to ketone	8/149	93/18670	7.83E-07	5.61E-06	2.05E-06	PPARG/TGFB1/EGFR/ICAM1/CFTR/SRC/CASP9/AKT1
GO:0032368	regulation of lipid transport	9/149	127/18670	8.20E-07	5.86E-06	2.14E-06	LEP/APOE/PON1/PPARG/PLA2G6/AKT1/NFKB1/IL1B/NFKBIA
GO:0035270	endocrine system development	9/149	127/18670	8.20E-07	5.86E-06	2.14E-06	THRB/SOX9/DRD2/NKX2-1/GSK3B/IL6/MAPK1/THRA/AKT1
GO:0150077	regulation of neuroinflammatory response	6/149	41/18670	8.34E-07	5.94E-06	2.17E-06	IGF1/TNF/PTGS2/IL6/IL1B/MMP9
GO:1902893	regulation of pri-miRNA transcription by RNA polymerase II	6/149	41/18670	8.34E-07	5.94E-06	2.17E-06	SOX9/TGFB1/IL10/FOS/TP53/JUN
GO:0050680	negative regulation of epithelial cell proliferation	10/149	165/18670	8.41E-07	5.96E-06	2.18E-06	APOE/PPARG/SOX9/TNF/TGFB1/STAT1/CAV1/RB1/SPARC/SNAI2
GO:0050954	sensory perception of mechanical stimulus	10/149	165/18670	8.41E-07	5.96E-06	2.18E-06	COL1A1/THRB/TRPA1/KCNQ1/TRPV1/CASP3/SOD1/ICAM1/HTR2A/SNAI2
GO:0071695	anatomical structure maturation	10/149	165/18670	8.41E-07	5.96E-06	2.18E-06	MMP2/IGF1/LEP/BCL2/RET/CTNNB1/GRIN1/RB1/PGR/CDKN1A
GO:0051187	cofactor catabolic process	7/149	65/18670	8.46E-07	5.99E-06	2.19E-06	DUOX2/MPO/SNCA/GGT1/HMOX1/TPO/CAT

GO:0043900	regulation of multi-organism process	15/149	405/18670	8.89E-07	6.28E-06	2.30E-06	IGF1/NOS2/APOE/BCL2/CXCL8/TNF/STAT1/FMR1/NODAL/IFNG/CAV1/ACVR1B/IL1B/JUN/NOTCH1
GO:0050995	negative regulation of lipid catabolic process	5/149	23/18670	9.07E-07	6.39E-06	2.34E-06	ADRA2A/TNF/INS/AKT1/IL1B
GO:0051882	mitochondrial depolarization	5/149	23/18670	9.07E-07	6.39E-06	2.34E-06	BCL2/PARP1/KDR/ABL1/SRC
GO:0007623	circadian rhythm	11/149	208/18670	9.09E-07	6.39E-06	2.34E-06	NOS2/SLC6A4/LEP/PPARG/DRD2/NKX2-1/GSK3B/EGFR/DRD4/TP53/JUN
GO:0042100	B cell proliferation	8/149	95/18670	9.22E-07	6.46E-06	2.36E-06	BCL2/IL13/CASP3/IL10/IL2/ABL1/CDKN1A/BAX
GO:0061326	renal tubule development	8/149	95/18670	9.22E-07	6.46E-06	2.36E-06	BCL2/SOX9/CTNNB1/TGFB1/MYC/STAT1/FGF2/NOTCH1
GO:0071482	cellular response to light stimulus	9/149	129/18670	9.36E-07	6.55E-06	2.40E-06	PTGS2/MYC/PARP1/FMR1/TP53/CASP9/CDKN1A/PIK3R1/BAX
GO:0051785	positive regulation of nuclear division	7/149	66/18670	9.40E-07	6.57E-06	2.40E-06	IGF1/IL1A/TGFB1/INS/IGF2/RB1/IL1B
GO:0051701	interaction with host	11/149	209/18670	9.53E-07	6.65E-06	2.43E-06	MET/DPP4/CASP8/CXCL8/CTNNB1/TGFB1/EGFR/ICAM1/CAV1/SRC/HTR2A
GO:0006509	membrane protein ectodomain proteolysis	6/149	42/18670	9.67E-07	6.73E-06	2.46E-06	APOE/ADRA2A/TNF/IL10/IFNG/IL1B
GO:0019217	regulation of fatty acid metabolic process	8/149	96/18670	9.99E-07	6.95E-06	2.54E-06	PPARG/PTGS2/SNCA/INS/CAV1/AKT1/IL1B/AVP
GO:0042108	positive regulation of cytokine biosynthetic process	7/149	67/18670	1.04E-06	7.24E-06	2.65E-06	IRF1/TNF/IL1A/IL6/HMOX1/IFNG/IL1B
GO:0050728	negative regulation of inflammatory response	10/149	169/18670	1.05E-06	7.26E-06	2.66E-06	IGF1/APOE/PPARG/F2/INS/IL10/SOD1/IL2/RB1/NFKB1
GO:0001657	ureteric bud development	8/149	97/18670	1.08E-06	7.47E-06	2.73E-06	BCL2/RET/SOX9/CTNNB1/TGFB1/MYC/FGF2/CAT
GO:0051591	response to cAMP	8/149	97/18670	1.08E-06	7.47E-06	2.73E-06	COL1A1/DUOX2/STAT1/KCNQ1/FOS/CFTR/SPARC/JUN
GO:0008631	intrinsic apoptotic signaling pathway in response to oxidative stress	6/149	43/18670	1.12E-06	7.68E-06	2.81E-06	BCL2/NFE2L2/PARP1/INS/SOD1/AKT1

GO:0032459	regulation of protein oligomerization	6/149	43/18670	1.12E-06	7.68E-06	2.81E-06	APOE/MMP1/INS/SRC/TP53/BAX
GO:0071364	cellular response to epidermal growth factor stimulus	6/149	43/18670	1.12E-06	7.68E-06	2.81E-06	COL1A1/SOX9/EGFR/ERBB2/AKT1/SNAI2
GO:2000209	regulation of anoikis	5/149	24/18670	1.14E-06	7.82E-06	2.86E-06	BCL2/CAV1/SRC/SNAI2/NOTCH1
GO:0046323	glucose import	7/149	68/18670	1.15E-06	7.92E-06	2.90E-06	IGF1/LEP/NFE2L2/TNF/INS/AKT1/PIK3R1
GO:0006937	regulation of muscle contraction	10/149	171/18670	1.17E-06	7.97E-06	2.92E-06	NOS1/ADRA2A/PTGS2/KCNQ1/CACNA1C/SOD1/CAV1/CHRM3/SCN10A/SCN5A
GO:0072163	mesonephric epithelium development	8/149	98/18670	1.17E-06	7.97E-06	2.92E-06	BCL2/RET/SOX9/CTNNB1/TGFB1/MYC/FGF2/CAT
GO:0072164	mesonephric tubule development	8/149	98/18670	1.17E-06	7.97E-06	2.92E-06	BCL2/RET/SOX9/CTNNB1/TGFB1/MYC/FGF2/CAT
GO:2001243	negative regulation of intrinsic apoptotic signaling pathway	8/149	98/18670	1.17E-06	7.97E-06	2.92E-06	BCL2/NFE2L2/PTGS2/INS/SRC/AKT1/SNAI2/MMP9
GO:0051583	dopamine uptake involved in synaptic transmission	4/149	11/18670	1.23E-06	8.37E-06	3.06E-06	SLC6A2/DRD2/SNCA/DRD4
GO:0051934	catecholamine uptake involved in synaptic transmission	4/149	11/18670	1.23E-06	8.37E-06	3.06E-06	SLC6A2/DRD2/SNCA/DRD4
GO:0032388	positive regulation of intracellular transport	11/149	215/18670	1.26E-06	8.54E-06	3.12E-06	LEP/PTGS2/TGFB1/IL13/GSK3B/MAPK1/ERBB2/IFNG/TP53/PIK3R1/IL1B
GO:0015909	long-chain fatty acid transport	7/149	69/18670	1.28E-06	8.63E-06	3.16E-06	PLA2G2A/APOE/PPARG/PLA2G6/DRD2/DRD4/AKT1
GO:0042698	ovulation cycle	7/149	69/18670	1.28E-06	8.63E-06	3.16E-06	LEP/OPRK1/ESR1/CASP3/EGFR/SRC/PGR
GO:0070227	lymphocyte apoptotic process	7/149	69/18670	1.28E-06	8.63E-06	3.16E-06	FASLG/IL10/IL2/TP53/JAK3/AKT1/BAX
GO:0046688	response to copper ion	6/149	44/18670	1.28E-06	8.64E-06	3.16E-06	CYP1A1/NFE2L2/IL1A/SNCA/SOD1/ICAM1
GO:0030260	entry into host cell	9/149	134/18670	1.29E-06	8.64E-06	3.16E-06	MET/DPP4/CXCL8/CTNNB1/EGFR/ICAM1/CAV1/SRC/HTR2A

GO:0044409	entry into host	9/149	134/18670	1.29E-06	8.64E-06	3.16E-06	MET/DPP4/CXCL8/CTNNB1/EGFR/ICAM1/CAV1/SRC/HTR2A
GO:0051806	entry into cell of other organism involved in symbiotic interaction	9/149	134/18670	1.29E-06	8.64E-06	3.16E-06	MET/DPP4/CXCL8/CTNNB1/EGFR/ICAM1/CAV1/SRC/HTR2A
GO:0051828	entry into other organism involved in symbiotic interaction	9/149	134/18670	1.29E-06	8.64E-06	3.16E-06	MET/DPP4/CXCL8/CTNNB1/EGFR/ICAM1/CAV1/SRC/HTR2A
GO:0032869	cellular response to insulin stimulus	11/149	216/18670	1.32E-06	8.81E-06	3.23E-06	LEP/PPARG/GSK3B/STAT1/PARP1/INS/IGF2/SRC/AKT1/PIK3R1/IL1B
GO:0030301	cholesterol transport	8/149	100/18670	1.36E-06	9.11E-06	3.33E-06	LEP/APOE/PON1/PPARG/CAV1/CFTR/NFKB1/NFKBIA
GO:2001023	regulation of response to drug	8/149	100/18670	1.36E-06	9.11E-06	3.33E-06	MET/OPRK1/NFE2L2/DRD2/SNCA/IL10/DRD4/HTR2A
GO:0043393	regulation of protein binding	11/149	217/18670	1.38E-06	9.18E-06	3.36E-06	MET/APOE/BCL2/GSK3B/IL10/CAV1/ABL1/SRC/AKT1/BAX/MMP9
GO:0010517	regulation of phospholipase activity	7/149	70/18670	1.41E-06	9.38E-06	3.43E-06	P2RY12/ESR1/SNCA/EGFR/FGF2/ABL1/HTR2A
GO:0002719	negative regulation of cytokine production involved in immune response	5/149	25/18670	1.41E-06	9.38E-06	3.43E-06	TNF/TGFB1/HMOX1/IL10/JAK3
GO:0019430	removal of superoxide radicals	5/149	25/18670	1.41E-06	9.38E-06	3.43E-06	NFE2L2/TNF/MPO/NQO1/SOD1
GO:0150063	visual system development	14/149	366/18670	1.44E-06	9.51E-06	3.48E-06	BCL2/THRB/CYP1A1/RET/SOX9/CTNNB1/P2RY12/TGFB1/ACHE/CACNA1C/FASLG/EGFR/JUN/BAX
GO:1900271	regulation of long-term synaptic potentiation	6/149	45/18670	1.47E-06	9.72E-06	3.56E-06	APOE/DRD2/SHANK3/GSK3B/INS/ABL1
GO:1900274	regulation of phospholipase C activity	6/149	45/18670	1.47E-06	9.72E-06	3.56E-06	P2RY12/ESR1/EGFR/FGF2/ABL1/HTR2A
GO:0051961	negative regulation of nervous system development	13/149	315/18670	1.50E-06	9.91E-06	3.63E-06	SLC6A4/APOE/THRB/SOX9/TNF/F2/CTNNB1/TGFB1/GSK3B/IL6/TP53/IL1B/NOTCH1
GO:0034605	cellular response to heat	9/149	137/18670	1.55E-06	1.02E-05	3.74E-06	MAPT/IL1A/PTGS2/GSK3B/TRPV1/HSP90AA1/HMOX1/MAPK1/CDKN1A

GO:0042531	positive regulation of tyrosine phosphorylation of STAT protein	7/149	71/18670	1.55E-06	1.02E-05	3.74E-06	IGF1/LEP/TNF/IL13/IL6/IL2/IFNG
GO:0001823	mesonephros development	8/149	102/18670	1.59E-06	1.04E-05	3.81E-06	BCL2/RET/SOX9/CTNNB1/TGFB1/MYC/FGF2/CAT
GO:0001504	neurotransmitter uptake	6/149	46/18670	1.68E-06	1.10E-05	4.01E-06	SLC6A2/SLC6A4/NOS1/DRD2/SNCA/DRD4
GO:0030810	positive regulation of nucleotide biosynthetic process	6/149	46/18670	1.68E-06	1.10E-05	4.01E-06	IGF1/NOS2/NOS1/INS/IFNG/HTR2A
GO:0060443	mammary gland morphogenesis	6/149	46/18670	1.68E-06	1.10E-05	4.01E-06	ESR1/TGFB1/CAV1/SRC/PGR/BAX
GO:1900373	positive regulation of purine nucleotide biosynthetic process	6/149	46/18670	1.68E-06	1.10E-05	4.01E-06	IGF1/NOS2/NOS1/INS/IFNG/HTR2A
GO:1904036	negative regulation of epithelial cell apoptotic process	6/149	46/18670	1.68E-06	1.10E-05	4.01E-06	NFE2L2/IL13/KDR/HMOX1/ICAM1/ABL1
GO:0048880	sensory system development	14/149	371/18670	1.68E-06	1.10E-05	4.01E-06	BCL2/THRB/CYP1A1/RET/SOX9/CTNNB1/P2RY12/TGFB1/ACHE/CACNA1C/FASLG/EGFR/JUN/BAX
GO:0034766	negative regulation of ion transmembrane transport	8/149	103/18670	1.71E-06	1.11E-05	4.06E-06	ADRA2A/DRD2/TGFB1/FMR1/CAV1/DRD4/AKT1/MMP9
GO:0007249	I-kappaB kinase/NF-kappaB signaling	12/149	269/18670	1.73E-06	1.12E-05	4.10E-06	CASP8/TNF/CTNNB1/ESR1/AVPR2/STAT1/FASLG/HMOX1/ABL1/AKT1/IL1B/NFKBIA
GO:0032800	receptor biosynthetic process	5/149	26/18670	1.74E-06	1.13E-05	4.13E-06	PPARG/TNF/ACHE/IL10/IFNG
GO:0002700	regulation of production of molecular mediator of immune response	9/149	139/18670	1.75E-06	1.13E-05	4.14E-06	TNF/TGFB1/IL13/IL6/HMOX1/IL10/IL2/JAK3/IL1B
GO:0050715	positive regulation of cytokine secretion	9/149	139/18670	1.75E-06	1.13E-05	4.14E-06	TNF/IL1A/DRD2/INS/IL10/IFNG/ABL1/SRC/IL1B
GO:0045165	cell fate commitment	12/149	270/18670	1.80E-06	1.16E-05	4.24E-06	BCL2/PPARG/SOX9/CTNNB1/NKX2-1/IL6/CASP3/NODAL/FGF2/TCF3/TP53/NOTCH1
GO:0045080	positive regulation of chemokine biosynthetic process	4/149	12/18670	1.84E-06	1.18E-05	4.33E-06	TNF/HMOX1/IFNG/IL1B

GO:0043627	response to estrogen	7/149	73/18670	1.88E-06	1.21E-05	4.42E-06	OPRK1/PPARG/CCND1/ESR1/HMOX1/MAPK1/CAV1
GO:0022408	negative regulation of cell-cell adhesion	10/149	181/18670	1.95E-06	1.25E-05	4.59E-06	IRF1/TGFB1/CASP3/IL10/IL2/ERBB2/ABL1/JAK3/AKT1/SNAI2
GO:0050709	negative regulation of protein secretion	9/149	141/18670	1.97E-06	1.26E-05	4.63E-06	APOE/ADRA2A/TNF/DRD2/HMGCR/INS/IL10/DRD4/IL1B
GO:0014015	positive regulation of gliogenesis	7/149	74/18670	2.06E-06	1.32E-05	4.83E-06	PPARG/TNF/P2RY12/TGFB1/IL6/IL1B/NOTCH1
GO:0072006	nephron development	9/149	142/18670	2.09E-06	1.33E-05	4.88E-06	BCL2/RET/SOX9/CTNNB1/TGFB1/MYC/STAT1/FGF2/NOTCH1
GO:1900542	regulation of purine nucleotide metabolic process	9/149	142/18670	2.09E-06	1.33E-05	4.88E-06	IGF1/NOS2/NOS1/SNCA/PARP1/INS/IFNG/TP53/HTR2A
GO:0051953	negative regulation of amine transport	5/149	27/18670	2.12E-06	1.35E-05	4.93E-06	LEP/ADRA2A/DRD2/P2RY12/SNCA
GO:0071450	cellular response to oxygen radical	5/149	27/18670	2.12E-06	1.35E-05	4.93E-06	NFE2L2/TNF/MPO/NQO1/SOD1
GO:0071451	cellular response to superoxide	5/149	27/18670	2.12E-06	1.35E-05	4.93E-06	NFE2L2/TNF/MPO/NQO1/SOD1
GO:1902175	regulation of oxidative stress-induced intrinsic apoptotic signaling pathway	5/149	27/18670	2.12E-06	1.35E-05	4.93E-06	NFE2L2/PARP1/INS/SOD1/AKT1
GO:0035272	exocrine system development	6/149	48/18670	2.17E-06	1.38E-05	5.05E-06	SOX9/TNF/TGFB1/IGF2/EGFR/SNAI2
GO:0009615	response to virus	13/149	326/18670	2.20E-06	1.39E-05	5.09E-06	IRF1/BCL2/CYP1A1/DUOX2/OPRK1/TNF/STAT1/IL6/ABCC9/FMR1/IFNG/SRC/IL1B
GO:0031056	regulation of histone modification	9/149	143/18670	2.21E-06	1.40E-05	5.11E-06	NOS1/CTNNB1/SNCA/TGFB1/FMR1/IGF2/TP53/IL1B/SNAI2
GO:0106106	cold-induced thermogenesis	9/149	143/18670	2.21E-06	1.40E-05	5.11E-06	LEP/PRDM16/IL13/ACHE/CAV1/RB1/THRA/GATM/NOTCH1
GO:0120161	regulation of cold-induced thermogenesis	9/149	143/18670	2.21E-06	1.40E-05	5.11E-06	LEP/PRDM16/IL13/ACHE/CAV1/RB1/THRA/GATM/NOTCH1
GO:0043401	steroid hormone mediated signaling pathway	10/149	184/18670	2.26E-06	1.42E-05	5.21E-06	THRB/PPARG/CTNNB1/ESR1/PARP1/NODAL/RB1/SRC/PGR/THRA

GO:0048639	positive regulation of developmental growth	10/149	184/18670	2.26E-06	1.42E-05	5.21E-06	IGF1/LEP/BCL2/MAPT/DRD2/MAPK1/IGF2/FGF2/AKT1/NOTCH1
GO:1903708	positive regulation of hemopoiesis	10/149	185/18670	2.38E-06	1.49E-05	5.46E-06	CASP8/TNF/TGFB1/STAT1/IL2/IFNG/FOS/RB1/ACVR1B/JUN
GO:1904659	glucose transmembrane transport	8/149	108/18670	2.45E-06	1.53E-05	5.61E-06	IGF1/LEP/NFE2L2/TNF/INS/AKT1/PIK3R1/IL1B
GO:0003254	regulation of membrane depolarization	6/149	49/18670	2.46E-06	1.54E-05	5.64E-06	BCL2/PARP1/KDR/SRC/SCN10A/SCN5A
GO:0007492	endoderm development	7/149	76/18670	2.47E-06	1.54E-05	5.64E-06	MMP2/CTNNB1/TGFB1/NKX2-1/NODAL/NOTCH1/MMP9
GO:0061045	negative regulation of wound healing	7/149	76/18670	2.47E-06	1.54E-05	5.64E-06	PLAU/APOE/F2/HMGCR/FGF2/CDKN1A/F12
GO:0072088	nephron epithelium morphogenesis	7/149	76/18670	2.47E-06	1.54E-05	5.64E-06	BCL2/SOX9/CTNNB1/TGFB1/MYC/STAT1/FGF2
GO:0071901	negative regulation of protein serine/threonine kinase activity	9/149	145/18670	2.48E-06	1.55E-05	5.66E-06	APOE/CASP3/HMGCR/CAV1/ABL1/RB1/CDKN1A/AKT1/IL1B
GO:0003180	aortic valve morphogenesis	5/149	28/18670	2.57E-06	1.59E-05	5.82E-06	SOX9/TGFB1/RB1/SNAI2/NOTCH1
GO:1902003	regulation of amyloid-beta formation	5/149	28/18670	2.57E-06	1.59E-05	5.82E-06	IGF1/APOE/TNF/CASP3/IFNG
GO:1990776	response to angiotensin	5/149	28/18670	2.57E-06	1.59E-05	5.82E-06	NFE2L2/PTGS2/CAV1/SRC/NFKB1
GO:0072009	nephron epithelium development	8/149	109/18670	2.62E-06	1.62E-05	5.94E-06	BCL2/SOX9/CTNNB1/TGFB1/MYC/STAT1/FGF2/NOTCH1
GO:0006140	regulation of nucleotide metabolic process	9/149	146/18670	2.63E-06	1.63E-05	5.95E-06	IGF1/NOS2/NOS1/SNCA/PARP1/INS/IFNG/TP53/HTR2A
GO:0010718	positive regulation of epithelial to mesenchymal transition	6/149	50/18670	2.78E-06	1.71E-05	6.26E-06	COL1A1/CTNNB1/TGFB1/IL6/IL1B/NOTCH1
GO:0060425	lung morphogenesis	6/149	50/18670	2.78E-06	1.71E-05	6.26E-06	SOX9/TNF/CTNNB1/NKX2-1/MAPK1/NODAL
GO:1903793	positive regulation of anion transport	6/149	50/18670	2.78E-06	1.71E-05	6.26E-06	APOE/ABCB1/PLA2G6/CFTR/IL1B/AVP
GO:0071236	cellular response to antibiotic	9/149	147/18670	2.78E-06	1.71E-05	6.26E-06	MET/NFE2L2/IL6/IL10/NQO1/ABL1/CFTR/SRC/TP53

GO:0030336	negative regulation of cell migration	13/149	334/18670	2.87E-06	1.76E-05	6.45E-06	APOE/BCL2/PPARG/NFE2L2/DRD2/TGFB1/NKX2-1/HMOX1/NODAL/FGF2/AKT1/IGFBP3/NOTCH1
GO:0000271	polysaccharide biosynthetic process	7/149	78/18670	2.94E-06	1.80E-05	6.58E-06	IGF1/TGFB1/GSK3B/INS/IGF2/AKT1/NFKB1
GO:0031016	pancreas development	7/149	78/18670	2.94E-06	1.80E-05	6.58E-06	MET/SOX9/CTNNB1/GSK3B/IL6/IGF2/AKT1
GO:0072028	nephron morphogenesis	7/149	78/18670	2.94E-06	1.80E-05	6.58E-06	BCL2/SOX9/CTNNB1/TGFB1/MYC/STAT1/FGF2
GO:0072347	response to anesthetic	7/149	78/18670	2.94E-06	1.80E-05	6.58E-06	HTR3A/OPRK1/DRD2/SNCA/GRIN1/DRD4/HTR2A
GO:0000303	response to superoxide	5/149	29/18670	3.08E-06	1.87E-05	6.85E-06	NFE2L2/TNF/MPO/NQO1/SOD1
GO:0005979	regulation of glycogen biosynthetic process	5/149	29/18670	3.08E-06	1.87E-05	6.85E-06	IGF1/GSK3B/INS/IGF2/AKT1
GO:0007263	nitric oxide mediated signal transduction	5/149	29/18670	3.08E-06	1.87E-05	6.85E-06	NOS2/NOS1/APOE/INS/EGFR
GO:0010962	regulation of glucan biosynthetic process	5/149	29/18670	3.08E-06	1.87E-05	6.85E-06	IGF1/GSK3B/INS/IGF2/AKT1
GO:0034976	response to endoplasmic reticulum stress	12/149	285/18670	3.14E-06	1.91E-05	6.98E-06	BCL2/CXCL8/NFE2L2/PLA2G6/CCND1/GSK3B/CAV1/CFTR/TP53/PIK3R1/JUN/BAX
GO:0008645	hexose transmembrane transport	8/149	112/18670	3.22E-06	1.94E-05	7.10E-06	IGF1/LEP/NFE2L2/TNF/INS/AKT1/PIK3R1/IL1B
GO:0015918	sterol transport	8/149	112/18670	3.22E-06	1.94E-05	7.10E-06	LEP/APOE/PON1/PPARG/CAV1/CFTR/NFKB1/NFKBIA
GO:0042303	molting cycle	8/149	112/18670	3.22E-06	1.94E-05	7.10E-06	BCL2/SOX9/TNF/PTGS2/CTNNB1/EGFR/ACVR1B/NOTCH1
GO:0042633	hair cycle	8/149	112/18670	3.22E-06	1.94E-05	7.10E-06	BCL2/SOX9/TNF/PTGS2/CTNNB1/EGFR/ACVR1B/NOTCH1
GO:0050777	negative regulation of immune response	9/149	150/18670	3.29E-06	1.98E-05	7.25E-06	PPARG/TNF/DRD2/TGFB1/HMOX1/INS/IL10/IL2/JAK3
GO:0045088	regulation of innate immune response	15/149	452/18670	3.44E-06	2.07E-05	7.59E-06	IRF1/LEP/APOE/CASP8/PPARG/DRD2/ESR1/S100A8/STAT1/INS/IFNG/CAV1/SRC/NFKB1/NFKBIA
GO:0015872	dopamine transport	6/149	52/18670	3.51E-06	2.10E-05	7.69E-06	SLC6A2/OPRK1/DRD2/SNCA/DRD4/HTR2A
GO:0035176	social behavior	6/149	52/18670	3.51E-06	2.10E-05	7.69E-06	SLC6A4/SHANK3/NRXN1/GRIN1/DRD4/AVP

GO:0051703	intraspecies interaction between organisms	6/149	52/18670	3.51E-06	2.10E-05	7.69E-06	SLC6A4/SHANK3/NRXN1/GRIN1/DRD4/AVP
GO:2000677	regulation of transcription regulatory region DNA binding	6/149	52/18670	3.51E-06	2.10E-05	7.69E-06	IGF1/CTNNB1/TGFB1/PARP1/IFNG/RB1
GO:0031348	negative regulation of defense response	11/149	239/18670	3.51E-06	2.10E-05	7.69E-06	IGF1/APOE/PPARG/DRD2/F2/INS/IL10/SOD1/IL2/RB1/NFKB1
GO:0006813	potassium ion transport	11/149	240/18670	3.66E-06	2.17E-05	7.94E-06	NOS1/ADRA2A/OPRK1/SLC12A2/DRD2/P2RY12/KCNQ1/ABCC9/SLC12A1/CAV1/HTR2A
GO:0014854	response to inactivity	4/149	14/18670	3.66E-06	2.17E-05	7.94E-06	DRD2/IL10/CAT/SCN5A
GO:0000187	activation of MAPK activity	9/149	152/18670	3.67E-06	2.17E-05	7.94E-06	IGF1/ALK/RET/TNF/MAPK1/SOD1/FGF2/DRD4/IL1B
GO:0000305	response to oxygen radical	5/149	30/18670	3.67E-06	2.17E-05	7.94E-06	NFE2L2/TNF/MPO/NQO1/SOD1
GO:0010743	regulation of macrophage derived foam cell differentiation	5/149	30/18670	3.67E-06	2.17E-05	7.94E-06	PLA2G2A/PPARG/CRP/NFKB1/NFKBIA
GO:0048265	response to pain	5/149	30/18670	3.67E-06	2.17E-05	7.94E-06	SLC6A2/CACNA1B/RET/TRPA1/TRPV1
GO:0097421	liver regeneration	5/149	30/18670	3.67E-06	2.17E-05	7.94E-06	CCND1/TGFB1/HMOX1/IL10/EGFR
GO:0006690	icosanoid metabolic process	8/149	114/18670	3.67E-06	2.17E-05	7.94E-06	PON1/CYP1A1/PTGS2/CYP2E1/GGT1/IL1B/PTGS1/AVP
GO:0015749	monosaccharide transmembrane transport	8/149	114/18670	3.67E-06	2.17E-05	7.94E-06	IGF1/LEP/NFE2L2/TNF/INS/AKT1/PIK3R1/IL1B
GO:0042593	glucose homeostasis	11/149	241/18670	3.81E-06	2.25E-05	8.22E-06	LEP/ADRA2A/OPRK1/PPARG/PLA2G6/HMGCR/INS/ICAM1/CFTR/AKT1/PIK3R1
GO:0030168	platelet activation	9/149	153/18670	3.87E-06	2.27E-05	8.32E-06	COL1A1/APOE/ADRA2A/F2/P2RY12/IL6/MAPK1/SRC/PIK3R1
GO:0034250	positive regulation of cellular amide metabolic process	9/149	153/18670	3.87E-06	2.27E-05	8.32E-06	NFE2L2/PLA2G6/TNF/IL6/CASP3/FMR1/MAPK1/ERBB2/IFNG
GO:0045807	positive regulation of endocytosis	9/149	153/18670	3.87E-06	2.27E-05	8.32E-06	APOE/PPARG/TNF/DRD2/SNCA/FMR1/SOD1/IFNG/IL1B

GO:0071356	cellular response to tumor necrosis factor	12/149	291/18670	3.89E-06	2.29E-05	8.37E-06	COL1A1/CASP8/CXCL8/NFE2L2/TNF/STAT1/TRPV1/MAPK1/ICAM1/AKT1/NFKB1/NFKBIA
GO:0032964	collagen biosynthetic process	6/149	53/18670	3.94E-06	2.31E-05	8.44E-06	COL1A1/PPARG/F2/TGFB1/IL6/SERPINH1
GO:0033500	carbohydrate homeostasis	11/149	242/18670	3.96E-06	2.32E-05	8.49E-06	LEP/ADRA2A/OPRK1/PPARG/PLA2G6/HMGCR/INS/ICAM1/CFTR/AKT1/PIK3R1
GO:0055067	monovalent inorganic cation homeostasis	9/149	154/18670	4.08E-06	2.39E-05	8.73E-06	BCL2/SLC12A2/DRD2/AVPR2/FASLG/MAPK1/SLC12A1/CFTR/AVP
GO:0032642	regulation of chemokine production	7/149	82/18670	4.13E-06	2.41E-05	8.82E-06	TNF/IL6/HMOX1/IL10/IFNG/IL1B/SNAI2
GO:1904950	negative regulation of establishment of protein localization	10/149	197/18670	4.17E-06	2.43E-05	8.90E-06	APOE/ADRA2A/MAPT/TNF/DRD2/HMGCR/INS/IL10/DRD4/IL1B
GO:0034219	carbohydrate transmembrane transport	8/149	116/18670	4.18E-06	2.44E-05	8.92E-06	IGF1/LEP/NFE2L2/TNF/INS/AKT1/PIK3R1/IL1B
GO:0002675	positive regulation of acute inflammatory response	5/149	31/18670	4.35E-06	2.52E-05	9.22E-06	TNF/PTGS2/IL6/IL1B/F12
GO:0019098	reproductive behavior	5/149	31/18670	4.35E-06	2.52E-05	9.22E-06	THRB/OPRK1/GRIN1/THRA/AVP
GO:0033198	response to ATP	5/149	31/18670	4.35E-06	2.52E-05	9.22E-06	PTGS2/P2RY12/TRPV1/SOD1/IL1B
GO:1902895	positive regulation of pri-miRNA transcription by RNA polymerase II	5/149	31/18670	4.35E-06	2.52E-05	9.22E-06	TGFB1/IL10/FOS/TP53/JUN
GO:0006584	catecholamine metabolic process	6/149	54/18670	4.40E-06	2.54E-05	9.29E-06	DRD2/SNCA/HTR1A/MAOB/DRD4/PAH
GO:0009712	catechol-containing compound metabolic process	6/149	54/18670	4.40E-06	2.54E-05	9.29E-06	DRD2/SNCA/HTR1A/MAOB/DRD4/PAH
GO:0050994	regulation of lipid catabolic process	6/149	54/18670	4.40E-06	2.54E-05	9.29E-06	ADRA2A/TNF/INS/THRA/AKT1/IL1B
GO:0010822	positive regulation of mitochondrion organization	8/149	117/18670	4.46E-06	2.57E-05	9.41E-06	BCL2/CASP8/PLA2G6/GSK3B/KDR/TP53/BAX/MMP9

GO:0050866	negative regulation of cell activation	10/149	199/18670	4.56E-06	2.63E-05	9.61E-06	IRF1/APOE/F2/TGFB1/CASP3/HMOX1/IL10/IL2/ERBB2/JAK3
GO:0007178	transmembrane receptor protein serine/threonine kinase signaling pathway	13/149	349/18670	4.63E-06	2.66E-05	9.73E-06	PRDM16/TGFB1/NKX2-1/PARP1/NODAL/FOS/CAV1/ABL1/SRC/ACVR1B/TP53/JUN/NOTCH1
GO:2000146	negative regulation of cell motility	13/149	349/18670	4.63E-06	2.66E-05	9.73E-06	APOE/BCL2/PPARG/NFE2L2/DRD2/TGFB1/NKX2-1/HMOX1/NODAL/FGF2/AKT1/IGFBP3/NOTCH1
GO:0002718	regulation of cytokine production involved in immune response	7/149	84/18670	4.85E-06	2.78E-05	1.02E-05	TNF/TGFB1/IL6/HMOX1/IL10/JAK3/IL1B
GO:0001541	ovarian follicle development	6/149	55/18670	4.91E-06	2.80E-05	1.03E-05	BCL2/ESR1/SOD1/ICAM1/SRC/BAX
GO:0042220	response to cocaine	6/149	55/18670	4.91E-06	2.80E-05	1.03E-05	HTR3A/OPRK1/DRD2/SNCA/DRD4/HTR2A
GO:0042306	regulation of protein import into nucleus	6/149	55/18670	4.91E-06	2.80E-05	1.03E-05	LEP/PTGS2/TGFB1/MAPK1/IFNG/PIK3R1
GO:0034349	glial cell apoptotic process	4/149	15/18670	4.97E-06	2.83E-05	1.04E-05	CASP3/RB1/TP53/CASP9
GO:0090494	dopamine uptake	4/149	15/18670	4.97E-06	2.83E-05	1.04E-05	SLC6A2/DRD2/SNCA/DRD4
GO:0002703	regulation of leukocyte mediated immunity	10/149	201/18670	4.99E-06	2.84E-05	1.04E-05	LEP/TNF/TGFB1/IL13/IL6/HMOX1/IL10/IL2/JAK3/IL1B
GO:0001975	response to amphetamine	5/149	32/18670	5.12E-06	2.90E-05	1.06E-05	DRD2/SOD1/GRIN1/ICAM1/DRD4
GO:0003176	aortic valve development	5/149	32/18670	5.12E-06	2.90E-05	1.06E-05	SOX9/TGFB1/RB1/SNAI2/NOTCH1
GO:0038128	ERBB2 signaling pathway	5/149	32/18670	5.12E-06	2.90E-05	1.06E-05	HSP90AA1/EGFR/ERBB2/SRC/PIK3R1
GO:0042744	hydrogen peroxide catabolic process	5/149	32/18670	5.12E-06	2.90E-05	1.06E-05	DUOX2/MPO/SNCA/TPO/CAT
GO:0071295	cellular response to vitamin	5/149	32/18670	5.12E-06	2.90E-05	1.06E-05	LEP/COL1A1/PPARG/SNAI2/CYP24A1
GO:0072089	stem cell proliferation	8/149	120/18670	5.39E-06	3.04E-05	1.11E-05	ABCB1/DRD2/CTNNB1/TGFB1/FGF2/TP53/SNAI2/NOTCH1
GO:0030278	regulation of ossification	10/149	203/18670	5.44E-06	3.07E-05	1.12E-05	IGF1/BCL2/SOX9/TNF/CTNNB1/TGFB1/IL6/MAPK1/SNAI2/NOTCH1

GO:0010332	response to gamma radiation	6/149	56/18670	5.46E-06	3.07E-05	1.12E-05	BCL2/MYC/PARP1/TP53/CDKN1A/BAX
GO:0032615	interleukin-12 production	6/149	56/18670	5.46E-06	3.07E-05	1.12E-05	IRF1/LEP/IL10/IFNG/JAK3/NFKB1
GO:1903202	negative regulation of oxidative stress-induced cell death	6/149	56/18670	5.46E-06	3.07E-05	1.12E-05	MET/NFE2L2/CTNNB1/INS/IL10/AKT1
GO:0001942	hair follicle development	7/149	86/18670	5.68E-06	3.18E-05	1.16E-05	BCL2/SOX9/TNF/CTNNB1/EGFR/ACVR1B/NOTCH1
GO:0042446	hormone biosynthetic process	7/149	86/18670	5.68E-06	3.18E-05	1.16E-05	DUOX2/TNF/IFNG/TPO/NFKB1/IL1B/CYP3A4
GO:0097164	ammonium ion metabolic process	10/149	205/18670	5.94E-06	3.32E-05	1.22E-05	PLA2G2A/PON1/BCHE/PLA2G6/DRD2/SNCA/ACHE/HTR1A/MAOB/DRD4
GO:1902692	regulation of neuroblast proliferation	5/149	33/18670	6.00E-06	3.35E-05	1.22E-05	DRD2/CTNNB1/TGFB1/TP53/NOTCH1
GO:2000352	negative regulation of endothelial cell apoptotic process	5/149	33/18670	6.00E-06	3.35E-05	1.22E-05	NFE2L2/IL13/KDR/ICAM1/ABL1
GO:2001024	negative regulation of response to drug	5/149	33/18670	6.00E-06	3.35E-05	1.22E-05	MET/NFE2L2/DRD2/SNCA/IL10
GO:0045981	positive regulation of nucleotide metabolic process	6/149	57/18670	6.06E-06	3.37E-05	1.23E-05	IGF1/NOS2/NOS1/INS/IFNG/HTR2A
GO:1900408	negative regulation of cellular response to oxidative stress	6/149	57/18670	6.06E-06	3.37E-05	1.23E-05	MET/NFE2L2/CTNNB1/INS/IL10/AKT1
GO:1900544	positive regulation of purine nucleotide metabolic process	6/149	57/18670	6.06E-06	3.37E-05	1.23E-05	IGF1/NOS2/NOS1/INS/IFNG/HTR2A
GO:0001892	embryonic placenta development	7/149	87/18670	6.14E-06	3.40E-05	1.24E-05	CASP8/IL10/MAPK1/IGF2/EGFR/NODAL/AKT1
GO:0006112	energy reserve metabolic process	7/149	87/18670	6.14E-06	3.40E-05	1.24E-05	IGF1/LEP/MYC/GSK3B/INS/IGF2/AKT1
GO:0051781	positive regulation of cell division	7/149	87/18670	6.14E-06	3.40E-05	1.24E-05	IL1A/DRD2/TGFB1/IGF2/FGF2/CAT/IL1B

GO:0070555	response to interleukin-1	10/149	207/18670	6.47E-06	3.57E-05	1.31E-05	CXCL8/SOX9/IL1A/SNCA/IL6/ICAM1/SRC/NFKB1/IL1B/NFKBIA
GO:0071456	cellular response to hypoxia	10/149	207/18670	6.47E-06	3.57E-05	1.31E-05	BCL2/NFE2L2/PTGS2/MYC/HMOX1/ICAM1/SRC/TP53/AKT1/NOTCH1
GO:0051928	positive regulation of calcium ion transport	8/149	123/18670	6.47E-06	3.57E-05	1.31E-05	F2/SNCA/IL13/MYLK/GRIN1/CAV1/ABL1/BAX
GO:0060965	negative regulation of gene silencing by miRNA	4/149	16/18670	6.58E-06	3.62E-05	1.32E-05	PPARG/ESR1/TGFB1/TP53
GO:0090335	regulation of brown fat cell differentiation	4/149	16/18670	6.58E-06	3.62E-05	1.32E-05	LEP/PRDM16/PTGS2/INS
GO:0090493	catecholamine uptake	4/149	16/18670	6.58E-06	3.62E-05	1.32E-05	SLC6A2/DRD2/SNCA/DRD4
GO:0022404	molting cycle process	7/149	88/18670	6.62E-06	3.62E-05	1.32E-05	BCL2/SOX9/TNF/CTNNB1/EGFR/ACVR1B/NOTCH1
GO:0022405	hair cycle process	7/149	88/18670	6.62E-06	3.62E-05	1.32E-05	BCL2/SOX9/TNF/CTNNB1/EGFR/ACVR1B/NOTCH1
GO:0098773	skin epidermis development	7/149	88/18670	6.62E-06	3.62E-05	1.32E-05	BCL2/SOX9/TNF/CTNNB1/EGFR/ACVR1B/NOTCH1
GO:1901888	regulation of cell junction assembly	7/149	88/18670	6.62E-06	3.62E-05	1.32E-05	TNF/KDR/GPBAR1/CAV1/ABL1/SRC/SNAI2
GO:0031663	lipopolysaccharide-mediated signaling pathway	6/149	58/18670	6.72E-06	3.66E-05	1.34E-05	TNF/TGFB1/MAPK1/AKT1/IL1B/NFKBIA
GO:1904589	regulation of protein import	6/149	58/18670	6.72E-06	3.66E-05	1.34E-05	LEP/PTGS2/TGFB1/MAPK1/IFNG/PIK3R1
GO:0001654	eye development	13/149	362/18670	6.87E-06	3.75E-05	1.37E-05	BCL2/THRB/CYP1A1/RET/SOX9/CTNNB1/TGFB1/ACHE/CACNA1C/FASLG/EGFR/JUN/BAX
GO:0032148	activation of protein kinase B activity	5/149	34/18670	6.99E-06	3.79E-05	1.39E-05	IGF1/ADRA2A/INS/SRC/AKT1
GO:0034205	amyloid-beta formation	5/149	34/18670	6.99E-06	3.79E-05	1.39E-05	IGF1/APOE/TNF/CASP3/IFNG
GO:0045920	negative regulation of exocytosis	5/149	34/18670	6.99E-06	3.79E-05	1.39E-05	ADRA2A/SNCA/HMOX1/FMR1/NOTCH1
GO:0033673	negative regulation of kinase activity	11/149	257/18670	7.03E-06	3.81E-05	1.39E-05	APOE/MAPT/CASP3/HMGCR/IFNG/CAV1/ABL1/RB1/CDKN1A/AKT1/IL1B

GO:0048863	stem cell differentiation	11/149	257/18670	7.03E-06	3.81E-05	1.39E-05	BCHE/RET/NFE2L2/SOX9/ESR1/MAPK1/ABL1/TCF3/TP53/SNAI2/NOTCH1
GO:0006006	glucose metabolic process	10/149	209/18670	7.04E-06	3.81E-05	1.39E-05	IGF1/LEP/TNF/GSK3B/INS/IGF2/SRC/TP53/AKT1/IGFBP3
GO:0030111	regulation of Wnt signaling pathway	13/149	363/18670	7.08E-06	3.83E-05	1.40E-05	COL1A1/APOE/SOX9/CTNNB1/ESR1/GSK3B/EGFR/CAV1/ABL1/SRC/NFKB1/SNAI2/NOTCH1
GO:0007193	adenylate cyclase-inhibiting G protein-coupled receptor signaling pathway	7/149	89/18670	7.14E-06	3.85E-05	1.41E-05	ADRA2A/OPRK1/DRD2/P2RY12/HTR1A/DRD4/CHRM3
GO:0032410	negative regulation of transporter activity	7/149	89/18670	7.14E-06	3.85E-05	1.41E-05	ADRA2A/DRD2/SNCA/FMR1/CAV1/DRD4/MMP9
GO:0006805	xenobiotic metabolic process	8/149	125/18670	7.29E-06	3.93E-05	1.44E-05	BCHE/CYP1A1/CYP2E1/GGT1/NQO1/GRIN1/PTGS1/CYP3A4
GO:0050707	regulation of cytokine secretion	10/149	210/18670	7.34E-06	3.95E-05	1.44E-05	CRP/TNF/IL1A/DRD2/INS/IL10/IFNG/ABL1/SRC/IL1B
GO:0001658	branching involved in ureteric bud morphogenesis	6/149	59/18670	7.43E-06	3.98E-05	1.46E-05	BCL2/SOX9/CTNNB1/TGFB1/MYC/FGF2
GO:0032890	regulation of organic acid transport	6/149	59/18670	7.43E-06	3.98E-05	1.46E-05	LEP/PLA2G6/SNCA/AKT1/IL1B/AVP
GO:1902883	negative regulation of response to oxidative stress	6/149	59/18670	7.43E-06	3.98E-05	1.46E-05	MET/NFE2L2/CTNNB1/INS/IL10/AKT1
GO:0010717	regulation of epithelial to mesenchymal transition	7/149	90/18670	7.69E-06	4.11E-05	1.50E-05	COL1A1/CTNNB1/TGFB1/NKX2-1/IL6/IL1B/NOTCH1
GO:0031058	positive regulation of histone modification	7/149	90/18670	7.69E-06	4.11E-05	1.50E-05	NOS1/CTNNB1/TGFB1/FMR1/TP53/IL1B/SNAI2
GO:1903035	negative regulation of response to wounding	7/149	90/18670	7.69E-06	4.11E-05	1.50E-05	PLAU/APOE/F2/HMGCR/FGF2/CDKN1A/F12
GO:0002701	negative regulation of production of molecular mediator of immune response	5/149	35/18670	8.10E-06	4.29E-05	1.57E-05	TNF/TGFB1/HMOX1/IL10/JAK3
GO:0051354	negative regulation of oxidoreductase activity	5/149	35/18670	8.10E-06	4.29E-05	1.57E-05	SNCA/IL13/INS/CAV1/NFKB1

GO:0070873	regulation of glycogen metabolic process	5/149	35/18670	8.10E-06	4.29E-05	1.57E-05	IGF1/GSK3B/INS/IGF2/AKT1
GO:1901030	positive regulation of mitochondrial outer membrane permeabilization involved in apoptotic signaling pathway	5/149	35/18670	8.10E-06	4.29E-05	1.57E-05	BCL2/CASP8/GSK3B/TP53/BAX
GO:1902991	regulation of amyloid precursor protein catabolic process	5/149	35/18670	8.10E-06	4.29E-05	1.57E-05	IGF1/APOE/TNF/CASP3/IFNG
GO:1904031	positive regulation of cyclin-dependent protein kinase activity	5/149	35/18670	8.10E-06	4.29E-05	1.57E-05	CCND1/EGFR/SRC/CDKN1A/AKT1
GO:1904063	negative regulation of cation transmembrane transport	7/149	91/18670	8.28E-06	4.38E-05	1.60E-05	ADRA2A/DRD2/TGFB1/FMR1/CAV1/DRD4/MMP9
GO:0045725	positive regulation of glycogen biosynthetic process	4/149	17/18670	8.55E-06	4.51E-05	1.65E-05	IGF1/INS/IGF2/AKT1
GO:0050665	hydrogen peroxide biosynthetic process	4/149	17/18670	8.55E-06	4.51E-05	1.65E-05	CYP1A1/DUOX2/MAOB/SOD1
GO:2001267	regulation of cysteine-type endopeptidase activity involved in apoptotic signaling pathway	4/149	17/18670	8.55E-06	4.51E-05	1.65E-05	FAS/CASP8/BAX/MMP9
GO:2001020	regulation of response to DNA damage stimulus	10/149	214/18670	8.67E-06	4.57E-05	1.67E-05	BCL2/MAPT/MYC/PARP1/FMR1/EGFR/ABL1/TP53/CASP9/SNAI2
GO:0051100	negative regulation of binding	9/149	169/18670	8.70E-06	4.58E-05	1.68E-05	MET/GSK3B/HMOX1/IL10/CAV1/AKT1/NFKBIA/JUN/BAX
GO:0030888	regulation of B cell proliferation	6/149	61/18670	9.03E-06	4.74E-05	1.73E-05	BCL2/IL13/CASP3/IL10/IL2/CDKN1A
GO:0090303	positive regulation of wound healing	6/149	61/18670	9.03E-06	4.74E-05	1.73E-05	ADRA2A/DUOX2/NFE2L2/F2/MYLK/F12

GO:2001244	positive regulation of intrinsic apoptotic signaling pathway	6/149	61/18670	9.03E-06	4.74E-05	1.73E-05	BCL2/S100A8/SOD1/CAV1/TP53/BAX
GO:1903038	negative regulation of leukocyte cell-cell adhesion	8/149	129/18670	9.20E-06	4.82E-05	1.76E-05	IRF1/TGFB1/CASP3/IL10/IL2/ERBB2/JAK3/AKT1
GO:0060428	lung epithelium development	5/149	36/18670	9.35E-06	4.89E-05	1.79E-05	THRB/SOX9/NKX2-1/IL13/THRA
GO:0090218	positive regulation of lipid kinase activity	5/149	36/18670	9.35E-06	4.89E-05	1.79E-05	F2/P2RY12/TGFB1/FGF2/SRC
GO:0036294	cellular response to decreased oxygen levels	10/149	217/18670	9.79E-06	5.11E-05	1.87E-05	BCL2/NFE2L2/PTGS2/MYC/HMOX1/ICAM1/SRC/TP53/AKT1/NOTCH1
GO:0010676	positive regulation of cellular carbohydrate metabolic process	6/149	62/18670	9.94E-06	5.17E-05	1.89E-05	IGF1/SNCA/INS/IGF2/SRC/AKT1
GO:0050433	regulation of catecholamine secretion	6/149	62/18670	9.94E-06	5.17E-05	1.89E-05	ADRA2A/OPRK1/DRD2/P2RY12/SNCA/HTR2A
GO:0021543	pallium development	9/149	172/18670	1.00E-05	5.21E-05	1.91E-05	ALK/CTNNB1/P2RY12/NKX2-1/GSK3B/CASP3/EGFR/GRIN1/BAX
GO:0051262	protein tetramerization	9/149	172/18670	1.00E-05	5.21E-05	1.91E-05	GRIN2B/TRPA1/SNCA/ACHE/TRPV1/HMGCR/GRIN1/TP53/CAT
GO:0006814	sodium ion transport	10/149	218/18670	1.02E-05	5.29E-05	1.94E-05	NOS1/SLC12A2/DRD2/TGFB1/SLC12A1/SLC6A8/DRD4/AKT1/SCN10A/SCN5A
GO:0060993	kidney morphogenesis	7/149	94/18670	1.03E-05	5.32E-05	1.95E-05	BCL2/SOX9/CTNNB1/TGFB1/MYC/STAT1/FGF2
GO:0030183	B cell differentiation	8/149	131/18670	1.03E-05	5.33E-05	1.95E-05	BCL2/IL10/ABL1/TCF3/TP53/JAK3/PIK3R1/BAX
GO:0001662	behavioral fear response	5/149	37/18670	1.07E-05	5.52E-05	2.02E-05	DPP4/APOE/BCL2/HTR1A/DRD4
GO:0030212	hyaluronan metabolic process	5/149	37/18670	1.07E-05	5.52E-05	2.02E-05	TGFB1/FGF2/AKT1/NFKB1/IL1B
GO:0034405	response to fluid shear stress	5/149	37/18670	1.07E-05	5.52E-05	2.02E-05	NFE2L2/PTGS2/TGFB1/SRC/AKT1
GO:0038083	peptidyl-tyrosine autophosphorylation	5/149	37/18670	1.07E-05	5.52E-05	2.02E-05	KDR/EGFR/CAV1/ABL1/SRC

GO:0090050	positive regulation of cell migration involved in sprouting angiogenesis	5/149	37/18670	1.07E-05	5.52E-05	2.02E-05	PTGS2/KDR/HMOX1/FGF2/ABL1
GO:1905314	semi-lunar valve development	5/149	37/18670	1.07E-05	5.52E-05	2.02E-05	SOX9/TGFB1/RB1/SNAI2/NOTCH1
GO:2000142	regulation of DNA-templated transcription, initiation	5/149	37/18670	1.07E-05	5.52E-05	2.02E-05	CTNNB1/ESR1/THRA/TP53/JUN
GO:0002637	regulation of immunoglobulin production	6/149	63/18670	1.09E-05	5.56E-05	2.04E-05	TNF/TGFB1/IL13/IL6/IL10/IL2
GO:0032371	regulation of sterol transport	6/149	63/18670	1.09E-05	5.56E-05	2.04E-05	LEP/APOE/PON1/PPARG/NFKB1/NFKBIA
GO:0032374	regulation of cholesterol transport	6/149	63/18670	1.09E-05	5.56E-05	2.04E-05	LEP/APOE/PON1/PPARG/NFKB1/NFKBIA
GO:0042982	amyloid precursor protein metabolic process	6/149	63/18670	1.09E-05	5.56E-05	2.04E-05	IGF1/APOE/TNF/ACHE/CASP3/IFNG
GO:0035994	response to muscle stretch	4/149	18/18670	1.09E-05	5.56E-05	2.04E-05	FOS/NFKB1/NFKBIA/JUN
GO:0070875	positive regulation of glycogen metabolic process	4/149	18/18670	1.09E-05	5.56E-05	2.04E-05	IGF1/INS/IGF2/AKT1
GO:0071318	cellular response to ATP	4/149	18/18670	1.09E-05	5.56E-05	2.04E-05	PTGS2/P2RY12/TRPV1/SOD1
GO:1900221	regulation of amyloid-beta clearance	4/149	18/18670	1.09E-05	5.56E-05	2.04E-05	APOE/TNF/HMGCR/IFNG
GO:0042102	positive regulation of T cell proliferation	7/149	95/18670	1.10E-05	5.58E-05	2.04E-05	IGF1/LEP/IL6/IGF2/IL2/JAK3/IL1B
GO:1903076	regulation of protein localization to plasma membrane	7/149	95/18670	1.10E-05	5.58E-05	2.04E-05	TNF/TGFB1/INS/EGFR/IFNG/AKT1/PIK3R1
GO:2001252	positive regulation of chromosome organization	9/149	174/18670	1.10E-05	5.58E-05	2.04E-05	NOS1/CTNNB1/TGFB1/FMR1/MAPK1/RB1/TP53/IL1B/SNAI2
GO:0010660	regulation of muscle cell apoptotic process	7/149	96/18670	1.18E-05	5.97E-05	2.18E-05	IGF1/PPARG/NFE2L2/HMGCR/HMOX1/IFNG/TP53

GO:0097327	response to antineoplastic agent	7/149	96/18670	1.18E-05	5.97E-05	2.18E-05	CTNNB1/TGFB1/HMOX1/FMR1/EGFR/ICAM1/CASP9
GO:0050432	catecholamine secretion	6/149	64/18670	1.20E-05	6.04E-05	2.21E-05	ADRA2A/OPRK1/DRD2/P2RY12/SNCA/HTR2A
GO:1903672	positive regulation of sprouting angiogenesis	6/149	64/18670	1.20E-05	6.04E-05	2.21E-05	PTGS2/KDR/HMOX1/IL10/FGF2/ABL1
GO:0006766	vitamin metabolic process	8/149	134/18670	1.22E-05	6.13E-05	2.24E-05	CYP1A1/TNF/IFNG/NFKB1/IL1B/SNAI2/CYP24A1/CYP3A4
GO:0002209	behavioral defense response	5/149	38/18670	1.23E-05	6.18E-05	2.26E-05	DPP4/APOE/BCL2/HTR1A/DRD4
GO:0046326	positive regulation of glucose import	5/149	38/18670	1.23E-05	6.18E-05	2.26E-05	IGF1/NFE2L2/INS/AKT1/PIK3R1
GO:0030316	osteoclast differentiation	7/149	97/18670	1.26E-05	6.33E-05	2.32E-05	TNF/CTNNB1/TGFB1/IFNG/FOS/SRC/PIK3R1
GO:0048709	oligodendrocyte differentiation	7/149	97/18670	1.26E-05	6.33E-05	2.32E-05	PPARG/SOX9/CTNNB1/TGFB1/NKX2-1/ERBB2/NOTCH1
GO:0120162	positive regulation of cold-induced thermogenesis	7/149	97/18670	1.26E-05	6.33E-05	2.32E-05	LEP/PRDM16/IL13/ACHE/CAV1/THRA/GATM
GO:0051271	negative regulation of cellular component movement	13/149	384/18670	1.29E-05	6.47E-05	2.37E-05	APOE/BCL2/PPARG/NFE2L2/DRD2/TGFB1/NKX2-1/HMOX1/NODAL/FGF2/AKT1/IGFBP3/NOTCH1
GO:0060675	ureteric bud morphogenesis	6/149	65/18670	1.31E-05	6.55E-05	2.40E-05	BCL2/SOX9/CTNNB1/TGFB1/MYC/FGF2
GO:0042692	muscle cell differentiation	13/149	385/18670	1.33E-05	6.63E-05	2.43E-05	IGF1/NOS1/BCL2/SOX9/CTNNB1/TGFB1/CASP3/IGF2/ABL1/RB1/TCF3/AKT1/NOTCH1
GO:0033157	regulation of intracellular protein transport	10/149	225/18670	1.34E-05	6.70E-05	2.45E-05	LEP/PTGS2/TGFB1/GSK3B/MAPK1/ERBB2/IFNG/TP53/PIK3R1/IL1B
GO:0002042	cell migration involved in sprouting angiogenesis	7/149	98/18670	1.35E-05	6.73E-05	2.46E-05	PTGS2/KDR/HMOX1/FGF2/ABL1/AKT1/NOTCH1
GO:0060191	regulation of lipase activity	7/149	98/18670	1.35E-05	6.73E-05	2.46E-05	P2RY12/ESR1/SNCA/EGFR/FGF2/ABL1/HTR2A
GO:0060149	negative regulation of posttranscriptional gene silencing	4/149	19/18670	1.38E-05	6.83E-05	2.50E-05	PPARG/ESR1/TGFB1/TP53

GO:0060967	negative regulation of gene silencing by RNA	4/149	19/18670	1.38E-05	6.83E-05	2.50E-05	PPARG/ESR1/TGFB1/TP53
GO:1903798	regulation of production of miRNAs involved in gene silencing by miRNA	4/149	19/18670	1.38E-05	6.83E-05	2.50E-05	ESR1/TGFB1/EGFR/TP53
GO:0008361	regulation of cell size	9/149	179/18670	1.38E-05	6.85E-05	2.51E-05	APOE/RET/MAPT/SLC12A2/SHANK3/GSK3B/SLC12A1/ABL1/AKT1
GO:0007210	serotonin receptor signaling pathway	5/149	39/18670	1.40E-05	6.92E-05	2.53E-05	HTR3A/HTR1A/DRD4/CHRM3/HTR2A
GO:0042417	dopamine metabolic process	5/149	39/18670	1.40E-05	6.92E-05	2.53E-05	DRD2/SNCA/HTR1A/MAOB/DRD4
GO:1902042	negative regulation of extrinsic apoptotic signaling pathway via death domain receptors	5/149	39/18670	1.40E-05	6.92E-05	2.53E-05	FAS/CASP8/FASLG/HMOX1/ICAM1
GO:0031644	regulation of neurological system process	8/149	137/18670	1.43E-05	7.04E-05	2.58E-05	OPRK1/SHANK3/NRXN1/FMR1/IL10/GRIN1/SRC/AVP
GO:0046626	regulation of insulin receptor signaling pathway	6/149	66/18670	1.43E-05	7.04E-05	2.58E-05	LEP/INS/IGF2/SRC/PIK3R1/IL1B
GO:0046888	negative regulation of hormone secretion	6/149	66/18670	1.43E-05	7.04E-05	2.58E-05	LEP/ADRA2A/OPRK1/DRD2/HMGCR/IL1B
GO:0072171	mesonephric tubule morphogenesis	6/149	66/18670	1.43E-05	7.04E-05	2.58E-05	BCL2/SOX9/CTNNB1/TGFB1/MYC/FGF2
GO:0070301	cellular response to hydrogen peroxide	7/149	99/18670	1.44E-05	7.10E-05	2.60E-05	MET/NFE2L2/IL6/IL10/NQO1/ABL1/SRC
GO:0001894	tissue homeostasis	10/149	227/18670	1.45E-05	7.12E-05	2.60E-05	BCL2/SOX9/CTNNB1/IL6/EGFR/SOD1/RB1/SRC/NOTCH1/BAX
GO:0046631	alpha-beta T cell activation	8/149	138/18670	1.51E-05	7.39E-05	2.70E-05	IRF1/BCL2/IL6/INS/IL2/IFNG/ABL1/JAK3
GO:0002285	lymphocyte activation involved in immune response	9/149	181/18670	1.51E-05	7.39E-05	2.70E-05	TGFB1/IL6/IL10/IL2/IFNG/ICAM1/ABL1/TP53/JAK3
GO:0010565	regulation of cellular ketone metabolic process	9/149	181/18670	1.51E-05	7.39E-05	2.70E-05	PPARG/PTGS2/SNCA/INS/NQO1/CAV1/AKT1/IL1B/AVP

GO:0016042	lipid catabolic process	12/149	333/18670	1.52E-05	7.42E-05	2.72E-05	LEP/PLA2G2A/APOE/ADRA2A/PLA2G6/TNF/INS/THRA/AKT1/IL1B/CYP24A1/CYP3A4
GO:0000082	G1/S transition of mitotic cell cycle	11/149	279/18670	1.52E-05	7.42E-05	2.72E-05	BCL2/CYP1A1/CCND1/MYC/EGFR/RB1/ACVR1B/TP53/CDKN1A/AKT1/BAX
GO:0007200	phospholipase C-activating G protein-coupled receptor signaling pathway	7/149	100/18670	1.54E-05	7.51E-05	2.75E-05	ADRA2A/OPRK1/DRD2/F2/P2RY12/ESR1/HTR2A
GO:0010657	muscle cell apoptotic process	7/149	100/18670	1.54E-05	7.51E-05	2.75E-05	IGF1/PPARG/NFE2L2/HMGCR/HMOX1/IFNG/TP53
GO:0022600	digestive system process	7/149	100/18670	1.54E-05	7.51E-05	2.75E-05	LEP/ADRA2A/OPRK1/SOX9/KCNQ1/TRPV1/CHRM3
GO:0048662	negative regulation of smooth muscle cell proliferation	6/149	67/18670	1.56E-05	7.57E-05	2.77E-05	PPARG/HMOX1/IL10/IFNG/CDKN1A/IGFBP3
GO:0051705	multi-organism behavior	6/149	67/18670	1.56E-05	7.57E-05	2.77E-05	SLC6A4/SHANK3/NRXN1/GRIN1/DRD4/AVP
GO:2000573	positive regulation of DNA biosynthetic process	6/149	67/18670	1.56E-05	7.57E-05	2.77E-05	CTNNB1/MYC/HSP90AA1/MAPK1/FGF2/SRC
GO:0045580	regulation of T cell differentiation	8/149	139/18670	1.59E-05	7.68E-05	2.81E-05	IRF1/TGFB1/SOD1/IL2/ERBB2/IFNG/ABL1/JAK3
GO:0007618	mating	5/149	40/18670	1.59E-05	7.68E-05	2.81E-05	SLC6A4/THRB/GRIN1/THRA/AVP
GO:0042596	fear response	5/149	40/18670	1.59E-05	7.68E-05	2.81E-05	DPP4/APOE/BCL2/HTR1A/DRD4
GO:0051281	positive regulation of release of sequestered calcium ion into cytosol	5/149	40/18670	1.59E-05	7.68E-05	2.81E-05	F2/SNCA/IL13/ABL1/BAX
GO:0002027	regulation of heart rate	7/149	101/18670	1.65E-05	7.93E-05	2.90E-05	DRD2/KCNQ1/TRPV1/CACNA1C/CAV1/SCN10A/SCN5A
GO:0046632	alpha-beta T cell differentiation	7/149	101/18670	1.65E-05	7.93E-05	2.90E-05	IRF1/BCL2/IL6/IL2/IFNG/ABL1/JAK3
GO:0033627	cell adhesion mediated by integrin	6/149	68/18670	1.70E-05	8.19E-05	3.00E-05	PLAU/DPP4/RET/P2RY12/ICAM1/SNAI2

GO:0042053	regulation of dopamine metabolic process	4/149	20/18670	1.71E-05	8.20E-05	3.00E-05	SNCA/HTR1A/MAOB/DRD4
GO:0042069	regulation of catecholamine metabolic process	4/149	20/18670	1.71E-05	8.20E-05	3.00E-05	SNCA/HTR1A/MAOB/DRD4
GO:0051580	regulation of neurotransmitter uptake	4/149	20/18670	1.71E-05	8.20E-05	3.00E-05	NOS1/DRD2/SNCA/DRD4
GO:2000045	regulation of G1/S transition of mitotic cell cycle	9/149	184/18670	1.72E-05	8.25E-05	3.02E-05	BCL2/CYP1A1/CCND1/EGFR/RB1/TP53/CDKN1A/AKT1/BAX
GO:0002367	cytokine production involved in immune response	7/149	102/18670	1.76E-05	8.41E-05	3.08E-05	TNF/TGFB1/IL6/HMOX1/IL10/JAK3/IL1B
GO:0045930	negative regulation of mitotic cell cycle	12/149	338/18670	1.76E-05	8.43E-05	3.08E-05	BCL2/TNF/CTNNB1/CCND1/TGFB1/IL10/EGFR/ABL1/RB1/TP53/CDKN1A/BAX
GO:0008286	insulin receptor signaling pathway	8/149	141/18670	1.76E-05	8.43E-05	3.08E-05	LEP/GSK3B/INS/IGF2/SRC/AKT1/PIK3R1/IL1B
GO:0040013	negative regulation of locomotion	13/149	396/18670	1.79E-05	8.52E-05	3.12E-05	APOE/BCL2/PPARG/NFE2L2/DRD2/TGFB1/NKX2-1/HMOX1/NODAL/FGF2/AKT1/IGFBP3/NOTCH1
GO:1902275	regulation of chromatin organization	9/149	185/18670	1.80E-05	8.56E-05	3.13E-05	NOS1/CTNNB1/SNCA/TGFB1/FMR1/IGF2/TP53/IL1B/SNAI2
GO:0050798	activated T cell proliferation	5/149	41/18670	1.80E-05	8.56E-05	3.13E-05	IGF1/CASP3/IGF2/IL2/ABL1
GO:1903170	negative regulation of calcium ion transmembrane transport	5/149	41/18670	1.80E-05	8.56E-05	3.13E-05	ADRA2A/DRD2/TGFB1/FMR1/DRD4
GO:0071300	cellular response to retinoic acid	6/149	69/18670	1.85E-05	8.80E-05	3.22E-05	SLC6A4/LEP/COL1A1/PPARG/RET/SOX9
GO:0002697	regulation of immune effector process	14/149	458/18670	1.86E-05	8.85E-05	3.24E-05	LEP/TNF/F2/TGFB1/IL13/STAT1/IL6/HMOX1/INS/IL10/IL2/IFNG/JAK3/IL1B
GO:1902106	negative regulation of leukocyte differentiation	7/149	103/18670	1.87E-05	8.87E-05	3.24E-05	IRF1/CTNNB1/MYC/IL2/ERBB2/JAK3/PIK3R1
GO:1905269	positive regulation of chromatin organization	7/149	103/18670	1.87E-05	8.87E-05	3.24E-05	NOS1/CTNNB1/TGFB1/FMR1/TP53/IL1B/SNAI2
GO:0051403	stress-activated MAPK cascade	11/149	286/18670	1.91E-05	9.05E-05	3.31E-05	LEP/FAS/OPRK1/TNF/MYC/HMGCR/MAPK1/EGFR/AKT1/NFKB1/IL1B

GO:0060828	regulation of canonical Wnt signaling pathway	11/149	286/18670	1.91E-05	9.05E-05	3.31E-05	COL1A1/APOE/SOX9/CTNNB1/GSK3B/EGFR/CAV1/SRC/NFKB1/SNAI2/NOTCH1
GO:0006469	negative regulation of protein kinase activity	10/149	235/18670	1.95E-05	9.21E-05	3.37E-05	APOE/CASP3/HMGCR/IFNG/CAV1/ABL1/RB1/CDKN1A/AKT1/IL1B
GO:0009755	hormone-mediated signaling pathway	10/149	235/18670	1.95E-05	9.21E-05	3.37E-05	THRB/PPARG/CTNNB1/ESR1/PARP1/NODAL/RB1/SRC/PGR/THRA
GO:0060560	developmental growth involved in morphogenesis	10/149	235/18670	1.95E-05	9.21E-05	3.37E-05	APOE/MAPT/SOX9/CTNNB1/ESR1/TGFB1/GSK3B/HSP90AA1/ABL1/NOTCH1
GO:0072091	regulation of stem cell proliferation	6/149	70/18670	2.01E-05	9.47E-05	3.47E-05	DRD2/CTNNB1/TGFB1/TP53/SNAI2/NOTCH1
GO:0010907	positive regulation of glucose metabolic process	5/149	42/18670	2.03E-05	9.55E-05	3.49E-05	IGF1/INS/IGF2/SRC/AKT1
GO:0006913	nucleocytoplasmic transport	12/149	343/18670	2.04E-05	9.56E-05	3.50E-05	LEP/PTGS2/TGFB1/GSK3B/MAPK1/IFNG/TP53/CDKN1A/AKT1/PIK3R1/IL1B/NFKBIA
GO:0072330	monocarboxylic acid biosynthetic process	12/149	343/18670	2.04E-05	9.56E-05	3.50E-05	IGF1/CYP1A1/PTGS2/CYP2E1/INS/IFNG/TP53/IL1B/PTGS1/HTR2A/CYP3A4/AVP
GO:1902107	positive regulation of leukocyte differentiation	8/149	144/18670	2.05E-05	9.63E-05	3.52E-05	CASP8/TNF/TGFB1/IL2/IFNG/FOS/RB1/JUN
GO:0015874	norepinephrine transport	4/149	21/18670	2.10E-05	9.77E-05	3.57E-05	SLC6A2/ADRA2A/P2RY12/SNCA
GO:0017000	antibiotic biosynthetic process	4/149	21/18670	2.10E-05	9.77E-05	3.57E-05	CYP1A1/DUOX2/MAOB/SOD1
GO:0050951	sensory perception of temperature stimulus	4/149	21/18670	2.10E-05	9.77E-05	3.57E-05	OPRK1/TRPA1/TRPV1/HTR2A
GO:0051412	response to corticosterone	4/149	21/18670	2.10E-05	9.77E-05	3.57E-05	CCND1/MAOB/FOS/CDKN1A
GO:0051900	regulation of mitochondrial depolarization	4/149	21/18670	2.10E-05	9.77E-05	3.57E-05	BCL2/PARP1/KDR/SRC
GO:0070920	regulation of production of small RNA involved in gene silencing by RNA	4/149	21/18670	2.10E-05	9.77E-05	3.57E-05	ESR1/TGFB1/EGFR/TP53

GO:1903055	positive regulation of extracellular matrix organization	4/149	21/18670	2.10E-05	9.77E-05	3.57E-05	SOX9/TGFB1/IL6/RB1
GO:0032872	regulation of stress-activated MAPK cascade	10/149	237/18670	2.10E-05	9.77E-05	3.57E-05	LEP/FAS/OPRK1/TNF/MYC/HMGCR/MAPK1/EGFR/AKT1/IL1B
GO:0043122	regulation of I-kappaB kinase/NF-kappaB signaling	10/149	237/18670	2.10E-05	9.77E-05	3.57E-05	CASP8/TNF/CTNNB1/ESR1/STAT1/FASLG/HMOX1/ABL1/AKT1/IL1B
GO:0043266	regulation of potassium ion transport	7/149	105/18670	2.12E-05	9.84E-05	3.60E-05	NOS1/ADRA2A/OPRK1/DRD2/KCNQ1/CAV1/HTR2A
GO:0050795	regulation of behavior	6/149	71/18670	2.18E-05	0.000101	3.70E-05	APOE/OPRK1/DRD2/NRXN1/INS/HTR1A
GO:0051169	nuclear transport	12/149	346/18670	2.22E-05	0.000103	3.76E-05	LEP/PTGS2/TGFB1/GSK3B/MAPK1/IFNG/TP53/CDKN1A/AKT1/PIK3R1/IL1B/NFKBIA
GO:0070302	regulation of stress-activated protein kinase signaling cascade	10/149	239/18670	2.26E-05	0.000105	3.83E-05	LEP/FAS/OPRK1/TNF/MYC/HMGCR/MAPK1/EGFR/AKT1/IL1B
GO:0061337	cardiac conduction	8/149	146/18670	2.27E-05	0.000105	3.83E-05	NOS1/KCNQ1/ABCC9/CACNA1C/CAV1/CACNA1S/SCN10A/SCN5A
GO:2000241	regulation of reproductive process	8/149	146/18670	2.27E-05	0.000105	3.83E-05	IGF1/SOX9/CTNNB1/ESR1/NODAL/SRC/ACVR1B/NOTCH1
GO:0010863	positive regulation of phospholipase C activity	5/149	43/18670	2.28E-05	0.000105	3.85E-05	P2RY12/ESR1/EGFR/FGF2/HTR2A
GO:1904646	cellular response to amyloid-beta	5/149	43/18670	2.28E-05	0.000105	3.85E-05	IGF1/GSK3B/PARP1/CACNA1A/ICAM1
GO:0043524	negative regulation of neuron apoptotic process	8/149	147/18670	2.38E-05	0.00011	4.01E-05	APOE/BCL2/SNCA/HMOX1/SOD1/GRIN1/JUN/BAX
GO:0090092	regulation of transmembrane receptor protein serine/threonine kinase signaling pathway	10/149	241/18670	2.43E-05	0.000112	4.08E-05	PRDM16/TGFB1/NKX2-1/PARP1/NODAL/CAV1/ABL1/ACVR1B/TP53/NOTCH1
GO:0060537	muscle tissue development	13/149	408/18670	2.44E-05	0.000112	4.10E-05	IGF1/BCL2/SOX9/CTNNB1/TGFB1/HMGCR/MAPK1/MYLK/FOS/FGF2/CAV1/RB1/NOTCH1
GO:0008643	carbohydrate transport	8/149	148/18670	2.50E-05	0.000115	4.20E-05	IGF1/LEP/NFE2L2/TNF/INS/AKT1/PIK3R1/IL1B

GO:0035148	tube formation	8/149	148/18670	2.50E-05	0.000115	4.20E-05	RET/SOX9/CTNNB1/TGFB1/CASP3/NODAL/ABL1/NOTCH1
GO:0007219	Notch signaling pathway	9/149	193/18670	2.51E-05	0.000115	4.21E-05	SOX9/TGFB1/MYC/STAT1/EGFR/AKT1/NFKBIA/SNAI2/NOTCH1
GO:0051224	negative regulation of protein transport	9/149	193/18670	2.51E-05	0.000115	4.21E-05	APOE/ADRA2A/TNF/DRD2/HMGCR/INS/IL10/DRD4/IL1B
GO:0006821	chloride transport	7/149	108/18670	2.55E-05	0.000116	4.24E-05	SLC12A2/ABCB1/GABRG3/SLC12A1/GABRD/CFTR/GABRA3
GO:0030878	thyroid gland development	4/149	22/18670	2.55E-05	0.000116	4.24E-05	THRB/NKX2-1/MAPK1/THRA
GO:0046628	positive regulation of insulin receptor signaling pathway	4/149	22/18670	2.55E-05	0.000116	4.24E-05	LEP/INS/IGF2/SRC
GO:0051000	positive regulation of nitric-oxide synthase activity	4/149	22/18670	2.55E-05	0.000116	4.24E-05	APOE/TNF/INS/AKT1
GO:0010828	positive regulation of glucose transmembrane transport	5/149	44/18670	2.56E-05	0.000116	4.24E-05	IGF1/NFE2L2/INS/AKT1/PIK3R1
GO:0030225	macrophage differentiation	5/149	44/18670	2.56E-05	0.000116	4.24E-05	CASP8/TGFB1/PARP1/RB1/MMP9
GO:0042987	amyloid precursor protein catabolic process	5/149	44/18670	2.56E-05	0.000116	4.24E-05	IGF1/APOE/TNF/CASP3/IFNG
GO:1901028	regulation of mitochondrial outer membrane permeabilization involved in apoptotic signaling pathway	5/149	44/18670	2.56E-05	0.000116	4.24E-05	BCL2/CASP8/GSK3B/TP53/BAX
GO:0045685	regulation of glial cell differentiation	6/149	73/18670	2.56E-05	0.000116	4.24E-05	PPARG/F2/CTNNB1/TGFB1/IL6/NOTCH1
GO:1901983	regulation of protein acetylation	6/149	73/18670	2.56E-05	0.000116	4.24E-05	NOS1/SNCA/TGFB1/GSK3B/IL1B/SNAI2
GO:0007517	muscle organ development	13/149	410/18670	2.57E-05	0.000116	4.25E-05	IGF1/BCL2/CTNNB1/TGFB1/ACHE/HMGCR/MAPK1/MYLK/FOS/FGF2/CAV1/RB1/NOTCH1

GO:0001818	negative regulation of cytokine production	11/149	296/18670	2.63E-05	0.000119	4.34E-05	IGF1/TNF/F2/TGFB1/IL13/IL6/HMOX1/IL10/IFNG/JAK3/NFKB1
GO:0072078	nephron tubule morphogenesis	6/149	74/18670	2.77E-05	0.000125	4.56E-05	BCL2/SOX9/CTNNB1/TGFB1/MYC/FGF2
GO:1900076	regulation of cellular response to insulin stimulus	6/149	74/18670	2.77E-05	0.000125	4.56E-05	LEP/INS/IGF2/SRC/PIK3R1/IL1B
GO:1903036	positive regulation of response to wounding	6/149	74/18670	2.77E-05	0.000125	4.56E-05	ADRA2A/DUOX2/NFE2L2/F2/MYLK/F12
GO:0044843	cell cycle G1/S phase transition	11/149	298/18670	2.79E-05	0.000126	4.60E-05	BCL2/CYP1A1/CCND1/MYC/EGFR/RB1/ACVR1B/TP53/CDKN1A/AKT1/BAX
GO:0071897	DNA biosynthetic process	9/149	196/18670	2.84E-05	0.000128	4.67E-05	PPARG/CTNNB1/MYC/HSP90AA1/MAPK1/FGF2/SRC/TP53/CDKN1A
GO:0048538	thymus development	5/149	45/18670	2.86E-05	0.000128	4.70E-05	BCL2/CTNNB1/MAPK1/SOD1/ABL1
GO:0006959	humoral immune response	12/149	356/18670	2.93E-05	0.000132	4.82E-05	PLA2G2A/BCL2/CRP/CXCL8/PLA2G6/TNF/F2/S100A8/IL6/IFNG/IL1B/NOTCH1
GO:0016202	regulation of striated muscle tissue development	8/149	152/18670	3.03E-05	0.000136	4.97E-05	IGF1/BCL2/CTNNB1/TGFB1/HMGCR/MAPK1/FGF2/NOTCH1
GO:0031623	receptor internalization	7/149	111/18670	3.04E-05	0.000136	4.98E-05	CXCL8/DRD2/SNCA/ACHE/FMR1/CAV1/DRD4
GO:0007617	mating behavior	4/149	23/18670	3.07E-05	0.000137	5.00E-05	THRB/GRIN1/THRA/AVP
GO:0045821	positive regulation of glycolytic process	4/149	23/18670	3.07E-05	0.000137	5.00E-05	IGF1/INS/IFNG/HTR2A
GO:0051589	negative regulation of neurotransmitter transport	4/149	23/18670	3.07E-05	0.000137	5.00E-05	NOS1/SNCA/FMR1/MAOB
GO:0072215	regulation of metanephros development	4/149	23/18670	3.07E-05	0.000137	5.00E-05	RET/CTNNB1/MYC/STAT1
GO:2000637	positive regulation of gene silencing by miRNA	4/149	23/18670	3.07E-05	0.000137	5.00E-05	TGFB1/FMR1/EGFR/TP53
GO:0042180	cellular ketone metabolic process	10/149	248/18670	3.10E-05	0.000138	5.05E-05	PPARG/PTGS2/SNCA/HMGCR/INS/NQO1/CAV1/AKT1/IL1B/AVP

GO:0005978	glycogen biosynthetic process	5/149	46/18670	3.19E-05	0.000141	5.17E-05	IGF1/GSK3B/INS/IGF2/AKT1
GO:0009250	glucan biosynthetic process	5/149	46/18670	3.19E-05	0.000141	5.17E-05	IGF1/GSK3B/INS/IGF2/AKT1
GO:0014075	response to amine	5/149	46/18670	3.19E-05	0.000141	5.17E-05	DRD2/SOD1/GRIN1/ICAM1/DRD4
GO:0022602	ovulation cycle process	5/149	46/18670	3.19E-05	0.000141	5.17E-05	LEP/ESR1/CASP3/SRC/PGR
GO:0033628	regulation of cell adhesion mediated by integrin	5/149	46/18670	3.19E-05	0.000141	5.17E-05	PLAU/DPP4/RET/P2RY12/SNAI2
GO:0019318	hexose metabolic process	10/149	249/18670	3.21E-05	0.000142	5.19E-05	IGF1/LEP/TNF/GSK3B/INS/IGF2/SRC/TP53/AKT1/IGFBP3
GO:0002065	columnar/cuboidal epithelial cell differentiation	7/149	112/18670	3.22E-05	0.000142	5.20E-05	IL13/GSK3B/NODAL/SOD1/ABL1/CDKN1A/NOTCH1
GO:0032411	positive regulation of transporter activity	7/149	112/18670	3.22E-05	0.000142	5.20E-05	PON1/HTR3A/ABCB1/SHANK3/IFNG/DRD4/CFTR
GO:0050868	negative regulation of T cell activation	7/149	112/18670	3.22E-05	0.000142	5.20E-05	IRF1/TGFB1/CASP3/IL10/IL2/ERBB2/JAK3
GO:0001937	negative regulation of endothelial cell proliferation	6/149	76/18670	3.22E-05	0.000142	5.20E-05	APOE/PPARG/TNF/STAT1/CAV1/SPARC
GO:0043200	response to amino acid	7/149	113/18670	3.41E-05	0.00015	5.49E-05	MMP2/COL1A1/TNF/CASP3/EGFR/GRIN1/ICAM1
GO:0046620	regulation of organ growth	7/149	113/18670	3.41E-05	0.00015	5.49E-05	IGF1/SLC6A4/MAPK1/SOD1/FGF2/AKT1/NOTCH1
GO:0048678	response to axon injury	6/149	77/18670	3.47E-05	0.000153	5.59E-05	BCL2/DRD2/P2RY12/SOD1/JUN/BAX
GO:1901861	regulation of muscle tissue development	8/149	155/18670	3.49E-05	0.000153	5.60E-05	IGF1/BCL2/CTNNB1/TGFB1/HMGCR/MAPK1/FGF2/NOTCH1
GO:1902652	secondary alcohol metabolic process	8/149	155/18670	3.49E-05	0.000153	5.60E-05	LEP/APOE/PON1/HMGCR/SOD1/CFTR/CAT/CYP3A4
GO:0050435	amyloid-beta metabolic process	5/149	47/18670	3.54E-05	0.000155	5.68E-05	IGF1/APOE/TNF/CASP3/IFNG

GO:0070231	T cell apoptotic process	5/149	47/18670	3.54E-05	0.000155	5.68E-05	FASLG/TP53/JAK3/AKT1/BAX
GO:0006575	cellular modified amino acid metabolic process	9/149	202/18670	3.59E-05	0.000157	5.74E-05	PLA2G2A/DUOX2/NFE2L2/GGT1/SOD1/SLC6A8/TPO/IYD/GATM
GO:0009746	response to hexose	9/149	202/18670	3.59E-05	0.000157	5.74E-05	ADRA2A/OPRK1/PLA2G6/PTGS2/TGFB1/CASP3/HMGCR/ICAM1/CFTR
GO:1902806	regulation of cell cycle G1/S phase transition	9/149	202/18670	3.59E-05	0.000157	5.74E-05	BCL2/CYP1A1/CCND1/EGFR/RB1/TP53/CDKN1A/AKT1/BAX
GO:0005976	polysaccharide metabolic process	7/149	114/18670	3.61E-05	0.000157	5.76E-05	IGF1/TGFB1/GSK3B/INS/IGF2/AKT1/NFKB1
GO:0045446	endothelial cell differentiation	7/149	114/18670	3.61E-05	0.000157	5.76E-05	MET/TNF/CTNNB1/KDR/ICAM1/IL1B/NOTCH1
GO:0048634	regulation of muscle organ development	8/149	156/18670	3.65E-05	0.000159	5.80E-05	IGF1/BCL2/CTNNB1/TGFB1/HMGCR/MAPK1/FGF2/NOTCH1
GO:0010288	response to lead ion	4/149	24/18670	3.66E-05	0.000159	5.80E-05	MAPT/PTGS2/CAT/SPARC
GO:0060148	positive regulation of posttranscriptional gene silencing	4/149	24/18670	3.66E-05	0.000159	5.80E-05	TGFB1/FMR1/EGFR/TP53
GO:1900078	positive regulation of cellular response to insulin stimulus	4/149	24/18670	3.66E-05	0.000159	5.80E-05	LEP/INS/IGF2/SRC
GO:2001026	regulation of endothelial cell chemotaxis	4/149	24/18670	3.66E-05	0.000159	5.80E-05	MET/KDR/FGF2/NOTCH1
GO:0032413	negative regulation of ion transmembrane transporter activity	6/149	78/18670	3.74E-05	0.000162	5.92E-05	ADRA2A/DRD2/FMR1/CAV1/DRD4/MMP9
GO:0061333	renal tubule morphogenesis	6/149	78/18670	3.74E-05	0.000162	5.92E-05	BCL2/SOX9/CTNNB1/TGFB1/MYC/FGF2
GO:1904375	regulation of protein localization to cell periphery	7/149	115/18670	3.82E-05	0.000165	6.04E-05	TNF/TGFB1/INS/EGFR/IFNG/AKT1/PIK3R1
GO:1903727	positive regulation of phospholipid metabolic process	5/149	48/18670	3.93E-05	0.00017	6.21E-05	P2RY12/TGFB1/FGF2/SRC/HTR2A

GO:0030193	regulation of blood coagulation	6/149	79/18670	4.02E-05	0.000173	6.35E-05	PLAU/APOE/NFE2L2/F2/CAV1/F12
GO:0021987	cerebral cortex development	7/149	116/18670	4.04E-05	0.000174	6.37E-05	CTNNB1/P2RY12/NKX2-1/GSK3B/EGFR/GRIN1/BAX
GO:0007187	G protein-coupled receptor signaling pathway, coupled to cyclic nucleotide second messenger	10/149	256/18670	4.06E-05	0.000175	6.39E-05	NOS1/ADRA2A/OPRK1/DRD2/P2RY12/AVPR2/HTR1A/DRD4/CHRM3/HTR2A
GO:0002673	regulation of acute inflammatory response	8/149	159/18670	4.18E-05	0.00018	6.58E-05	PPARG/TNF/F2/PTGS2/IL6/INS/IL1B/F12
GO:0022617	extracellular matrix disassembly	6/149	80/18670	4.32E-05	0.000184	6.75E-05	MMP2/DPP4/TGFB1/MMP1/IL6/MMP9
GO:0070509	calcium ion import	6/149	80/18670	4.32E-05	0.000184	6.75E-05	CACNA1B/CTNNB1/TRPV1/CACNA1C/CACNA1A/CACNA1S
GO:1900046	regulation of hemostasis	6/149	80/18670	4.32E-05	0.000184	6.75E-05	PLAU/APOE/NFE2L2/F2/CAV1/F12
GO:0002053	positive regulation of mesenchymal cell proliferation	4/149	25/18670	4.33E-05	0.000184	6.75E-05	SOX9/CTNNB1/MYC/STAT1
GO:0030813	positive regulation of nucleotide catabolic process	4/149	25/18670	4.33E-05	0.000184	6.75E-05	IGF1/INS/IFNG/HTR2A
GO:0045662	negative regulation of myoblast differentiation	4/149	25/18670	4.33E-05	0.000184	6.75E-05	SOX9/TNF/TGFB1/NOTCH1
GO:0051197	positive regulation of coenzyme metabolic process	4/149	25/18670	4.33E-05	0.000184	6.75E-05	IGF1/INS/IFNG/HTR2A
GO:0060444	branching involved in mammary gland duct morphogenesis	4/149	25/18670	4.33E-05	0.000184	6.75E-05	ESR1/TGFB1/SRC/PGR
GO:1903306	negative regulation of regulated secretory pathway	4/149	25/18670	4.33E-05	0.000184	6.75E-05	ADRA2A/HMOX1/FMR1/NOTCH1
GO:1904385	cellular response to angiotensin	4/149	25/18670	4.33E-05	0.000184	6.75E-05	NFE2L2/CAV1/SRC/NFKB1

GO:0048546	digestive tract morphogenesis	5/149	49/18670	4.35E-05	0.000185	6.77E-05	BCL2/CTNNB1/EGFR/NODAL/NOTCH1
GO:0051205	protein insertion into membrane	5/149	49/18670	4.35E-05	0.000185	6.77E-05	BCL2/CASP8/EGFR/TP53/BAX
GO:0002819	regulation of adaptive immune response	8/149	160/18670	4.37E-05	0.000185	6.78E-05	IRF1/TNF/TGFB1/IL6/IL10/IL2/JAK3/IL1B
GO:0007519	skeletal muscle tissue development	8/149	160/18670	4.37E-05	0.000185	6.78E-05	BCL2/CTNNB1/TGFB1/HMGCR/FOS/CAV1/RB1/NOTCH1
GO:0046661	male sex differentiation	8/149	160/18670	4.37E-05	0.000185	6.78E-05	BCL2/SOX9/CTNNB1/CCND1/ESR1/NKX2-1/ICAM1/BAX
GO:1903305	regulation of regulated secretory pathway	8/149	160/18670	4.37E-05	0.000185	6.78E-05	ADRA2A/DRD2/IL13/GSK3B/HMOX1/FMR1/HTR2A/NOTCH1
GO:0043010	camera-type eye development	11/149	314/18670	4.50E-05	0.00019	6.97E-05	THRB/CYP1A1/RET/SOX9/CTNNB1/TGFB1/ACHE/CACNA1C/EGFR/JUN/BAX
GO:0001701	in utero embryonic development	12/149	373/18670	4.61E-05	0.000195	7.14E-05	IGF1/CASP8/CTNNB1/IL10/MAPK1/IGF2/EGFR/NODAL/ACVR1B/TP53/AKT1/NOTCH1
GO:0031098	stress-activated protein kinase signaling cascade	11/149	315/18670	4.63E-05	0.000195	7.15E-05	LEP/FAS/OPRK1/TNF/MYC/HMGCR/MAPK1/EGFR/AKT1/NFKB1/IL1B
GO:0033238	regulation of cellular amine metabolic process	6/149	81/18670	4.63E-05	0.000195	7.15E-05	SNCA/INS/HTR1A/MAOB/NQO1/DRD4
GO:0110110	positive regulation of animal organ morphogenesis	6/149	81/18670	4.63E-05	0.000195	7.15E-05	SOX9/CTNNB1/TGFB1/MYC/NOTCH1/BAX
GO:0051216	cartilage development	9/149	209/18670	4.69E-05	0.000198	7.23E-05	LEP/COL1A1/SOX9/CTNNB1/TGFB1/SERPINH1/FGF2/THRA/SNAI2
GO:0001952	regulation of cell-matrix adhesion	7/149	119/18670	4.76E-05	0.0002	7.32E-05	PLAU/BCL2/GSK3B/KDR/ABL1/SRC/PIK3R1
GO:0051193	regulation of cofactor metabolic process	7/149	119/18670	4.76E-05	0.0002	7.32E-05	IGF1/NFE2L2/SNCA/INS/IFNG/TP53/HTR2A
GO:0032637	interleukin-8 production	6/149	82/18670	4.96E-05	0.000208	7.61E-05	NOS2/LEP/CRP/TNF/IL10/IL1B
GO:0055117	regulation of cardiac muscle contraction	6/149	82/18670	4.96E-05	0.000208	7.61E-05	NOS1/KCNQ1/CACNA1C/CAV1/SCN10A/SCN5A
GO:0090049	regulation of cell migration involved in sprouting angiogenesis	6/149	82/18670	4.96E-05	0.000208	7.61E-05	PTGS2/KDR/HMOX1/FGF2/ABL1/NOTCH1

GO:1905954	positive regulation of lipid localization	6/149	82/18670	4.96E-05	0.000208	7.61E-05	APOE/PON1/PLA2G6/NFKB1/IL1B/NFKBIA
GO:0002698	negative regulation of immune effector process	7/149	120/18670	5.02E-05	0.00021	7.68E-05	TNF/TGFB1/HMOX1/INS/IL10/IL2/JAK3
GO:0048169	regulation of long-term neuronal synaptic plasticity	4/149	26/18670	5.08E-05	0.000212	7.77E-05	DRD2/SNCA/SHANK3/GRIN1
GO:0035821	modification of morphology or physiology of other organism	8/149	164/18670	5.21E-05	0.000218	7.97E-05	NOS2/APOE/CASP8/F2/TGFB1/FMR1/IFNG/JUN
GO:0031330	negative regulation of cellular catabolic process	10/149	264/18670	5.25E-05	0.000219	8.03E-05	MET/LEP/BCL2/SNCA/HMOX1/INS/FMR1/IL10/TP53/AKT1
GO:0097366	response to bronchodilator	5/149	51/18670	5.29E-05	0.00022	8.07E-05	DRD2/SOD1/GRIN1/ICAM1/DRD4
GO:2001258	negative regulation of cation channel activity	5/149	51/18670	5.29E-05	0.00022	8.07E-05	DRD2/FMR1/CAV1/DRD4/MMP9
GO:0034101	erythrocyte homeostasis	7/149	122/18670	5.58E-05	0.000232	8.48E-05	STAT1/CASP3/HMOX1/RB1/THRA/ACVR1B/JAK3
GO:1903578	regulation of ATP metabolic process	7/149	122/18670	5.58E-05	0.000232	8.48E-05	IGF1/SNCA/PARP1/INS/IFNG/TP53/HTR2A
GO:0015893	drug transport	9/149	214/18670	5.63E-05	0.000234	8.55E-05	SLC6A2/OPRK1/DRD2/SNCA/MYC/SLC25A4/SLC6A8/DRD4/HTR2A
GO:0016051	carbohydrate biosynthetic process	9/149	214/18670	5.63E-05	0.000234	8.55E-05	IGF1/LEP/SNCA/TGFB1/GSK3B/INS/IGF2/AKT1/NFKB1
GO:0014910	regulation of smooth muscle cell migration	6/149	84/18670	5.69E-05	0.000235	8.62E-05	IGF1/PLAU/BCL2/NFE2L2/SRC/IGFBP3
GO:0043154	negative regulation of cysteine-type endopeptidase activity involved in apoptotic process	6/149	84/18670	5.69E-05	0.000235	8.62E-05	PTGS2/SNCA/SRC/AKT1/AVP/MMP9
GO:0050818	regulation of coagulation	6/149	84/18670	5.69E-05	0.000235	8.62E-05	PLAU/APOE/NFE2L2/F2/CAV1/F12
GO:0001820	serotonin secretion	3/149	10/18670	5.74E-05	0.000236	8.63E-05	SLC6A4/HTR1A/MAOB

GO:0032025	response to cobalt ion	3/149	10/18670	5.74E-05	0.000236	8.63E-05	CASP8/CASP3/CASP9
GO:0046532	regulation of photoreceptor cell differentiation	3/149	10/18670	5.74E-05	0.000236	8.63E-05	THRB/SOX9/NOTCH1
GO:0090336	positive regulation of brown fat cell differentiation	3/149	10/18670	5.74E-05	0.000236	8.63E-05	PRDM16/PTGS2/INS
GO:1900222	negative regulation of amyloid-beta clearance	3/149	10/18670	5.74E-05	0.000236	8.63E-05	TNF/HMGCR/IFNG
GO:1903799	negative regulation of production of miRNAs involved in gene silencing by miRNA	3/149	10/18670	5.74E-05	0.000236	8.63E-05	ESR1/TGFB1/TP53
GO:1903800	positive regulation of production of miRNAs involved in gene silencing by miRNA	3/149	10/18670	5.74E-05	0.000236	8.63E-05	TGFB1/EGFR/TP53
GO:0003179	heart valve morphogenesis	5/149	52/18670	5.82E-05	0.000239	8.73E-05	SOX9/TGFB1/RB1/SNAI2/NOTCH1
GO:0048168	regulation of neuronal synaptic plasticity	5/149	52/18670	5.82E-05	0.000239	8.73E-05	APOE/DRD2/SNCA/SHANK3/GRIN1
GO:0055078	sodium ion homeostasis	5/149	52/18670	5.82E-05	0.000239	8.73E-05	SLC12A2/DRD2/AVPR2/SLC12A1/AVP
GO:0010810	regulation of cell-substrate adhesion	9/149	215/18670	5.84E-05	0.000239	8.75E-05	PLAU/COL1A1/BCL2/GSK3B/KDR/ABL1/SRC/PIK3R1/NOTCH1
GO:1901568	fatty acid derivative metabolic process	8/149	167/18670	5.92E-05	0.000242	8.85E-05	PON1/CYP1A1/PTGS2/CYP2E1/GGT1/IL1B/PTGS1/AVP
GO:0009651	response to salt stress	4/149	27/18670	5.93E-05	0.000242	8.85E-05	TNF/TP53/BAX/AVP
GO:0035902	response to immobilization stress	4/149	27/18670	5.93E-05	0.000242	8.85E-05	CYP1A1/PPARG/TGFB1/FOS
GO:0046885	regulation of hormone biosynthetic process	4/149	27/18670	5.93E-05	0.000242	8.85E-05	TNF/IFNG/NFKB1/IL1B

GO:0200144	positive regulation of DNA-templated transcription, initiation	4/149	27/18670	5.93E-05	0.000242	8.85E-05	CTNNB1/ESR1/TP53/JUN
GO:0002028	regulation of sodium ion transport	6/149	85/18670	6.08E-05	0.000248	9.06E-05	NOS1/DRD2/TGFB1/DRD4/AKT1/SCN5A
GO:0014031	mesenchymal cell development	6/149	85/18670	6.08E-05	0.000248	9.06E-05	BCL2/RET/SOX9/MAPK1/SNAI2/NOTCH1
GO:0051302	regulation of cell division	8/149	168/18670	6.18E-05	0.000251	9.20E-05	IL1A/DRD2/TGFB1/MYC/IGF2/FGF2/CAT/IL1B
GO:0001838	embryonic epithelial tube formation	7/149	124/18670	6.18E-05	0.000251	9.20E-05	RET/SOX9/CTNNB1/TGFB1/CASP3/NODAL/ABL1
GO:0060348	bone development	9/149	217/18670	6.27E-05	0.000255	9.32E-05	IGF1/LEP/COL1A1/SOX9/TGFB1/SERPINH1/SRC/TP53/SPARC
GO:0035065	regulation of histone acetylation	5/149	53/18670	6.38E-05	0.000258	9.45E-05	NOS1/SNCA/TGFB1/IL1B/SNAI2
GO:0045661	regulation of myoblast differentiation	5/149	53/18670	6.38E-05	0.000258	9.45E-05	SOX9/TNF/TGFB1/IGFBP3/NOTCH1
GO:0070228	regulation of lymphocyte apoptotic process	5/149	53/18670	6.38E-05	0.000258	9.45E-05	IL10/IL2/TP53/JAK3/BAX
GO:0009165	nucleotide biosynthetic process	12/149	386/18670	6.41E-05	0.000259	9.49E-05	IGF1/NOS2/NOS1/PTGS2/SNCA/TGFB1/PARP1/INS/MAPK1/IFNG/TP53/HTR2A
GO:0045619	regulation of lymphocyte differentiation	8/149	169/18670	6.44E-05	0.00026	9.52E-05	IRF1/TGFB1/SOD1/IL2/ERBB2/IFNG/ABL1/JAK3
GO:0060538	skeletal muscle organ development	8/149	169/18670	6.44E-05	0.00026	9.52E-05	BCL2/CTNNB1/TGFB1/HMGCR/FOS/CAV1/RB1/NOTCH1
GO:0034637	cellular carbohydrate biosynthetic process	6/149	86/18670	6.49E-05	0.000262	9.58E-05	IGF1/LEP/GSK3B/INS/IGF2/AKT1
GO:0070542	response to fatty acid	6/149	86/18670	6.49E-05	0.000262	9.58E-05	PON1/PPARG/PTGS2/SRC/CAT/AKT1
GO:0019058	viral life cycle	11/149	328/18670	6.66E-05	0.000268	9.82E-05	DPP4/APOE/BCL2/CXCL8/TNF/FMR1/EGFR/ICAM1/CAV1/HTR2A/NOTCH1
GO:0090101	negative regulation of transmembrane receptor protein serine/threonine kinase signaling pathway	7/149	126/18670	6.84E-05	0.000275	0.000101	PRDM16/TGFB1/NKX2-1/CAV1/ABL1/TP53/NOTCH1

GO:0001516	prostaglandin biosynthetic process	4/149	28/18670	6.87E-05	0.000275	0.000101	PTGS2/IL1B/PTGS1/AVP
GO:0010894	negative regulation of steroid biosynthetic process	4/149	28/18670	6.87E-05	0.000275	0.000101	APOE/SOD1/NFKB1/SNAI2
GO:0033598	mammary gland epithelial cell proliferation	4/149	28/18670	6.87E-05	0.000275	0.000101	CCND1/ESR1/MAPK1/BAX
GO:0046457	prostanoid biosynthetic process	4/149	28/18670	6.87E-05	0.000275	0.000101	PTGS2/IL1B/PTGS1/AVP
GO:1902932	positive regulation of alcohol biosynthetic process	4/149	28/18670	6.87E-05	0.000275	0.000101	TNF/SNCA/IFNG/IL1B
GO:0061448	connective tissue development	10/149	273/18670	6.95E-05	0.000278	0.000102	LEP/COL1A1/SOX9/CTNNB1/TGFB1/SERPINH1/FGF2/THRA/SNAI2/NOTCH1
GO:0032655	regulation of interleukin-12 production	5/149	54/18670	6.99E-05	0.000279	0.000102	IRF1/IL10/IFNG/JAK3/NFKB1
GO:0097345	mitochondrial outer membrane permeabilization	5/149	54/18670	6.99E-05	0.000279	0.000102	BCL2/CASP8/GSK3B/TP53/BAX
GO:1901293	nucleoside phosphate biosynthetic process	12/149	390/18670	7.07E-05	0.000282	0.000103	IGF1/NOS2/NOS1/PTGS2/SNCA/TGFB1/PARP1/INS/MAPK1/IFNG/TP53/HTR2A
GO:0007188	adenylate cyclase-modulating G protein-coupled receptor signaling pathway	9/149	221/18670	7.22E-05	0.000288	0.000105	NOS1/ADRA2A/OPRK1/DRD2/P2RY12/AVPR2/HTR1A/DRD4/CHRM3
GO:0097194	execution phase of apoptosis	6/149	88/18670	7.39E-05	0.000294	0.000107	CASP8/IL6/CASP3/TP53/AKT1/BAX
GO:1902476	chloride transmembrane transport	6/149	88/18670	7.39E-05	0.000294	0.000107	SLC12A2/GABRG3/SLC12A1/GABRD/CFTR/GABRA3
GO:1903725	regulation of phospholipid metabolic process	6/149	88/18670	7.39E-05	0.000294	0.000107	P2RY12/TGFB1/FGF2/SRC/PIK3R1/HTR2A
GO:2000177	regulation of neural precursor cell proliferation	6/149	88/18670	7.39E-05	0.000294	0.000107	SLC6A4/DRD2/CTNNB1/TGFB1/TP53/NOTCH1

GO:0043903	regulation of symbiosis, encompassing mutualism through parasitism	9/149	222/18670	7.47E-05	0.000297	0.000109	APOE/BCL2/CXCL8/TNF/STAT1/FMR1/CAV1/JUN/NOTCH1
GO:0010469	regulation of signaling receptor activity	8/149	173/18670	7.59E-05	0.000301	0.00011	PLAU/ADRA2A/GRIN2B/SHANK3/IL10/GRIN1/IFNG/SRC
GO:0010823	negative regulation of mitochondrion organization	5/149	55/18670	7.64E-05	0.000302	0.000111	IGF1/MAPT/TP53/AKT1/AVP
GO:0043551	regulation of phosphatidylinositol 3-kinase activity	5/149	55/18670	7.64E-05	0.000302	0.000111	P2RY12/TGFB1/FGF2/SRC/PIK3R1
GO:0046456	icosanoid biosynthetic process	5/149	55/18670	7.64E-05	0.000302	0.000111	PTGS2/GGT1/IL1B/PTGS1/AVP
GO:0031652	positive regulation of heat generation	3/149	11/18670	7.84E-05	0.000308	0.000113	TNF/PTGS2/IL1B
GO:0046886	positive regulation of hormone biosynthetic process	3/149	11/18670	7.84E-05	0.000308	0.000113	TNF/IFNG/IL1B
GO:0051974	negative regulation of telomerase activity	3/149	11/18670	7.84E-05	0.000308	0.000113	PPARG/SRC/TP53
GO:0060767	epithelial cell proliferation involved in prostate gland development	3/149	11/18670	7.84E-05	0.000308	0.000113	SOX9/CTNNB1/NOTCH1
GO:0072584	caveolin-mediated endocytosis	3/149	11/18670	7.84E-05	0.000308	0.000113	MAPK1/CAV1/SRC
GO:0086016	AV node cell action potential	3/149	11/18670	7.84E-05	0.000308	0.000113	CACNA1C/SCN10A/SCN5A
GO:0086027	AV node cell to bundle of His cell signaling	3/149	11/18670	7.84E-05	0.000308	0.000113	CACNA1C/SCN10A/SCN5A
GO:1901201	regulation of extracellular matrix assembly	3/149	11/18670	7.84E-05	0.000308	0.000113	SOX9/TGFB1/NOTCH1
GO:2001269	positive regulation of cysteine-type endopeptidase activity	3/149	11/18670	7.84E-05	0.000308	0.000113	FAS/CASP8/BAX

	involved in apoptotic signaling pathway						
GO:1901019	regulation of calcium ion transmembrane transporter activity	6/149	89/18670	7.87E-05	0.000309	0.000113	NOS1/ADRA2A/PLA2G6/DRD2/FMR1/DRD4
GO:0007271	synaptic transmission, cholinergic	4/149	29/18670	7.92E-05	0.00031	0.000114	APOE/ACHE/NQO1/CHRM3
GO:0060441	epithelial tube branching involved in lung morphogenesis	4/149	29/18670	7.92E-05	0.00031	0.000114	SOX9/TNF/CTNNB1/NKX2-1
GO:0060070	canonical Wnt signaling pathway	11/149	335/18670	8.04E-05	0.000315	0.000115	COL1A1/APOE/SOX9/CTNNB1/GSK3B/EGFR/CAV1/SRC/NFKB1/SNAI2/NOTCH1
GO:0002695	negative regulation of leukocyte activation	8/149	175/18670	8.23E-05	0.000322	0.000118	IRF1/TGFB1/CASP3/HMOX1/IL10/IL2/ERBB2/JAK3
GO:0022029	telencephalon cell migration	5/149	56/18670	8.33E-05	0.000324	0.000119	DRD2/CTNNB1/P2RY12/NKX2-1/EGFR
GO:0031295	T cell costimulation	5/149	56/18670	8.33E-05	0.000324	0.000119	DPP4/CAV1/SRC/AKT1/PIK3R1
GO:0086065	cell communication involved in cardiac conduction	5/149	56/18670	8.33E-05	0.000324	0.000119	KCNQ1/CACNA1C/CAV1/SCN10A/SCN5A
GO:1903078	positive regulation of protein localization to plasma membrane	5/149	56/18670	8.33E-05	0.000324	0.000119	TNF/EGFR/IFNG/AKT1/PIK3R1
GO:0001656	metanephros development	6/149	90/18670	8.38E-05	0.000326	0.000119	BCL2/RET/SOX9/CTNNB1/MYC/STAT1
GO:0046849	bone remodeling	6/149	90/18670	8.38E-05	0.000326	0.000119	LEP/CTNNB1/TGFB1/IL6/EGFR/SRC
GO:0048469	cell maturation	8/149	177/18670	8.91E-05	0.000345	0.000126	BCL2/PPARG/RET/CTNNB1/RB1/CFTR/PGR/CDKN1A
GO:0014909	smooth muscle cell migration	6/149	91/18670	8.91E-05	0.000345	0.000126	IGF1/PLAU/BCL2/NFE2L2/SRC/IGFBP3
GO:0060333	interferon-gamma-mediated signaling pathway	6/149	91/18670	8.91E-05	0.000345	0.000126	IRF1/PPARG/STAT1/IFNG/ICAM1/TP53

GO:1901992	positive regulation of mitotic cell cycle phase transition	6/149	91/18670	8.91E-05	0.000345	0.000126	CYP1A1/CCND1/TGFB1/EGFR/RB1/AKT1
GO:0031294	lymphocyte costimulation	5/149	57/18670	9.07E-05	0.000348	0.000127	DPP4/CAV1/SRC/AKT1/PIK3R1
GO:0001782	B cell homeostasis	4/149	30/18670	9.08E-05	0.000348	0.000127	BCL2/CASP3/ABL1/BAX
GO:0001844	protein insertion into mitochondrial membrane involved in apoptotic signaling pathway	4/149	30/18670	9.08E-05	0.000348	0.000127	BCL2/CASP8/TP53/BAX
GO:0035066	positive regulation of histone acetylation	4/149	30/18670	9.08E-05	0.000348	0.000127	NOS1/TGFB1/IL1B/SNAI2
GO:0042759	long-chain fatty acid biosynthetic process	4/149	30/18670	9.08E-05	0.000348	0.000127	CYP1A1/PTGS2/CYP2E1/CYP3A4
GO:0045939	negative regulation of steroid metabolic process	4/149	30/18670	9.08E-05	0.000348	0.000127	APOE/SOD1/NFKB1/SNAI2
GO:0045940	positive regulation of steroid metabolic process	4/149	30/18670	9.08E-05	0.000348	0.000127	APOE/TNF/IFNG/IL1B
GO:0048147	negative regulation of fibroblast proliferation	4/149	30/18670	9.08E-05	0.000348	0.000127	PPARG/MYC/TP53/BAX
GO:0048873	homeostasis of number of cells within a tissue	4/149	30/18670	9.08E-05	0.000348	0.000127	BCL2/SOX9/NOTCH1/BAX
GO:0061037	negative regulation of cartilage development	4/149	30/18670	9.08E-05	0.000348	0.000127	LEP/SOX9/CTNNB1/SNAI2
GO:0071549	cellular response to dexamethasone stimulus	4/149	30/18670	9.08E-05	0.000348	0.000127	TGFB1/EGFR/ICAM1/CASP9
GO:0090200	positive regulation of release of cytochrome c from mitochondria	4/149	30/18670	9.08E-05	0.000348	0.000127	PLA2G6/TP53/BAX/MMP9
GO:0003158	endothelium development	7/149	132/18670	9.18E-05	0.000351	0.000128	MET/TNF/CTNNB1/KDR/ICAM1/IL1B/NOTCH1
GO:0030518	intracellular steroid hormone receptor signaling pathway	7/149	132/18670	9.18E-05	0.000351	0.000128	CTNNB1/ESR1/PARP1/NODAL/RB1/SRC/PGR

GO:0042476	odontogenesis	7/149	132/18670	9.18E-05	0.000351	0.000128	COL1A1/CTNNB1/TGFB1/SRC/SCN10A/BAX/SCN5A
GO:0072175	epithelial tube formation	7/149	132/18670	9.18E-05	0.000351	0.000128	RET/SOX9/CTNNB1/TGFB1/CASP3/NODAL/ABL1
GO:2000117	negative regulation of cysteine-type endopeptidase activity	6/149	92/18670	9.47E-05	0.000362	0.000132	PTGS2/SNCA/SRC/AKT1/AVP/MMP9
GO:0071347	cellular response to interleukin-1	8/149	179/18670	9.63E-05	0.000368	0.000134	CXCL8/SOX9/IL1A/IL6/ICAM1/NFKB1/IL1B/NFKBIA
GO:0031647	regulation of protein stability	10/149	284/18670	9.64E-05	0.000368	0.000135	IGF1/BCL2/SNCA/HSP90AA1/CASP3/MAPK1/SRC/TP53/CDKN1A/PIK3R1
GO:0017001	antibiotic catabolic process	5/149	58/18670	9.87E-05	0.000375	0.000137	DUOX2/MPO/SNCA/TPO/CAT
GO:0032722	positive regulation of chemokine production	5/149	58/18670	9.87E-05	0.000375	0.000137	TNF/IL6/HMOX1/IFNG/IL1B
GO:0055081	anion homeostasis	5/149	58/18670	9.87E-05	0.000375	0.000137	APOE/SLC12A2/FASLG/INS/SLC12A1
GO:1902041	regulation of extrinsic apoptotic signaling pathway via death domain receptors	5/149	58/18670	9.87E-05	0.000375	0.000137	FAS/CASP8/FASLG/HMOX1/ICAM1
GO:1905330	regulation of morphogenesis of an epithelium	8/149	180/18670	0.0001	0.00038	0.000139	SOX9/TNF/CTNNB1/ESR1/TGFB1/STAT1/ABL1/SNAI2
GO:1901184	regulation of ERBB signaling pathway	6/149	93/18670	0.000101	0.000381	0.00014	ADRA2A/FASLG/EGFR/ERBB2/AKT1/MMP9
GO:0007006	mitochondrial membrane organization	7/149	134/18670	0.000101	0.000382	0.00014	BCL2/CASP8/SNCA/GSK3B/HSP90AA1/TP53/BAX
GO:0002440	production of molecular mediator of immune response	10/149	286/18670	0.000102	0.000387	0.000141	TNF/TGFB1/IL13/IL6/HMOX1/IL10/IL2/TCF3/JAK3/IL1B
GO:0044262	cellular carbohydrate metabolic process	10/149	286/18670	0.000102	0.000387	0.000141	IGF1/LEP/SNCA/GSK3B/INS/IGF2/SRC/TP53/AKT1/IGFBP3
GO:0010165	response to X-ray	4/149	31/18670	0.000104	0.000389	0.000142	CCND1/CASP3/TP53/CDKN1A

GO:0035767	endothelial cell chemotaxis	4/149	31/18670	0.000104	0.000389	0.000142	MET/KDR/FGF2/NOTCH1
GO:0043457	regulation of cellular respiration	4/149	31/18670	0.000104	0.000389	0.000142	NOS2/PRDM16/SNCA/IFNG
GO:0045737	positive regulation of cyclin-dependent protein serine/threonine kinase activity	4/149	31/18670	0.000104	0.000389	0.000142	CCND1/EGFR/SRC/AKT1
GO:0045742	positive regulation of epidermal growth factor receptor signaling pathway	4/149	31/18670	0.000104	0.000389	0.000142	ADRA2A/FASLG/AKT1/MMP9
GO:0050482	arachidonic acid secretion	4/149	31/18670	0.000104	0.000389	0.000142	PLA2G2A/PLA2G6/DRD2/DRD4
GO:1903963	arachidonate transport	4/149	31/18670	0.000104	0.000389	0.000142	PLA2G2A/PLA2G6/DRD2/DRD4
GO:0031392	regulation of prostaglandin biosynthetic process	3/149	12/18670	0.000104	0.000389	0.000142	PTGS2/IL1B/AVP
GO:0045955	negative regulation of calcium ion-dependent exocytosis	3/149	12/18670	0.000104	0.000389	0.000142	ADRA2A/FMR1/NOTCH1
GO:0060391	positive regulation of SMAD protein signal transduction	3/149	12/18670	0.000104	0.000389	0.000142	TGFB1/PARP1/NODAL
GO:0086067	AV node cell to bundle of His cell communication	3/149	12/18670	0.000104	0.000389	0.000142	CACNA1C/SCN10A/SCN5A
GO:1902950	regulation of dendritic spine maintenance	3/149	12/18670	0.000104	0.000389	0.000142	APOE/GRIN2B/INS
GO:0051147	regulation of muscle cell differentiation	8/149	181/18670	0.000104	0.000389	0.000142	IGF1/BCL2/CTNNB1/TGFB1/IGF2/ABL1/TCF3/NOTCH1
GO:0050921	positive regulation of chemotaxis	7/149	135/18670	0.000106	0.000395	0.000145	MET/CXCL8/P2RY12/TGFB1/IL6/KDR/FGF2
GO:0001885	endothelial cell development	5/149	59/18670	0.000107	0.000399	0.000146	MET/TNF/CTNNB1/ICAM1/IL1B

GO:0010518	positive regulation of phospholipase activity	5/149	59/18670	0.000107	0.000399	0.000146	P2RY12/ESR1/EGFR/FGF2/HTR2A
GO:0021885	forebrain cell migration	5/149	59/18670	0.000107	0.000399	0.000146	DRD2/CTNNB1/P2RY12/NKX2-1/EGFR
GO:2000756	regulation of peptidyl-lysine acetylation	5/149	59/18670	0.000107	0.000399	0.000146	NOS1/SNCA/TGFB1/IL1B/SNAI2
GO:0007292	female gamete generation	7/149	136/18670	0.000111	0.000412	0.000151	IGF1/LEP/BCL2/PTGS2/CTNNB1/SRC/PGR
GO:0071326	cellular response to monosaccharide stimulus	7/149	136/18670	0.000111	0.000412	0.000151	LEP/ADRA2A/OPRK1/PLA2G6/HMGCR/ICAM1/CFTR
GO:0002040	sprouting angiogenesis	8/149	183/18670	0.000112	0.000417	0.000153	PTGS2/KDR/HMOX1/IL10/FGF2/ABL1/AKT1/NOTCH1
GO:0043123	positive regulation of I-kappaB kinase/NF-kappaB signaling	8/149	183/18670	0.000112	0.000417	0.000153	CASP8/TNF/CTNNB1/FASLG/HMOX1/ABL1/AKT1/IL1B
GO:0051196	regulation of coenzyme metabolic process	6/149	95/18670	0.000113	0.00042	0.000154	IGF1/SNCA/INS/IFNG/TP53/HTR2A
GO:0034330	cell junction organization	10/149	290/18670	0.000115	0.000425	0.000155	BCL2/TNF/CTNNB1/TGFB1/KDR/GPBAR1/CAV1/ABL1/SRC/SNAI2
GO:0072655	establishment of protein localization to mitochondrion	7/149	137/18670	0.000116	0.000429	0.000157	BCL2/CASP8/MAPT/HSP90AA1/TP53/AKT1/BAX
GO:1903670	regulation of sprouting angiogenesis	7/149	137/18670	0.000116	0.000429	0.000157	PTGS2/KDR/HMOX1/IL10/FGF2/ABL1/NOTCH1
GO:1902110	positive regulation of mitochondrial membrane permeability involved in apoptotic process	5/149	60/18670	0.000116	0.000429	0.000157	BCL2/CASP8/GSK3B/TP53/BAX
GO:0050864	regulation of B cell activation	8/149	184/18670	0.000117	0.000431	0.000158	BCL2/TGFB1/IL13/IL6/CASP3/IL10/IL2/CDKN1A
GO:0043552	positive regulation of phosphatidylinositol 3-kinase activity	4/149	32/18670	0.000118	0.000433	0.000159	P2RY12/TGFB1/FGF2/SRC
GO:0045589	regulation of regulatory T cell differentiation	4/149	32/18670	0.000118	0.000433	0.000159	IRF1/TGFB1/IL2/IFNG

GO:0051968	positive regulation of synaptic transmission, glutamatergic	4/149	32/18670	0.000118	0.000433	0.000159	PTGS2/SHANK3/NRXN1/EGFR
GO:0061311	cell surface receptor signaling pathway involved in heart development	4/149	32/18670	0.000118	0.000433	0.000159	CTNNB1/TGFB1/SNAI2/NOTCH1
GO:0086019	cell-cell signaling involved in cardiac conduction	4/149	32/18670	0.000118	0.000433	0.000159	KCNQ1/CACNA1C/SCN10A/SCN5A
GO:2001169	regulation of ATP biosynthetic process	6/149	96/18670	0.00012	0.000441	0.000161	IGF1/PARP1/INS/IFNG/TP53/HTR2A
GO:0005996	monosaccharide metabolic process	10/149	292/18670	0.000121	0.000445	0.000163	IGF1/LEP/TNF/GSK3B/INS/IGF2/SRC/TP53/AKT1/IGFBP3
GO:0090287	regulation of cellular response to growth factor stimulus	10/149	292/18670	0.000121	0.000445	0.000163	PRDM16/CTNNB1/TGFB1/NKX2-1/FGF2/CAV1/ABL1/TP53/IL1B/NOTCH1
GO:0008584	male gonad development	7/149	138/18670	0.000121	0.000445	0.000163	BCL2/SOX9/CCND1/ESR1/NKX2-1/ICAM1/BAX
GO:0009308	amine metabolic process	7/149	138/18670	0.000121	0.000445	0.000163	CYP1A1/SNCA/INS/HTR1A/MAOB/NQO1/DRD4
GO:0003170	heart valve development	5/149	61/18670	0.000126	0.000459	0.000168	SOX9/TGFB1/RB1/SNAI2/NOTCH1
GO:0007405	neuroblast proliferation	5/149	61/18670	0.000126	0.000459	0.000168	DRD2/CTNNB1/TGFB1/TP53/NOTCH1
GO:0032370	positive regulation of lipid transport	5/149	61/18670	0.000126	0.000459	0.000168	APOE/PON1/PLA2G6/IL1B/NFKBIA
GO:0006720	isoprenoid metabolic process	7/149	139/18670	0.000127	0.000463	0.000169	APOE/CYP1A1/ADH1C/CYP2E1/HMGCR/EGFR/CYP3A4
GO:0007586	digestion	7/149	139/18670	0.000127	0.000463	0.000169	LEP/ADRA2A/OPRK1/SOX9/KCNQ1/TRPV1/CHRM3
GO:0046546	development of primary male sexual characteristics	7/149	139/18670	0.000127	0.000463	0.000169	BCL2/SOX9/CCND1/ESR1/NKX2-1/ICAM1/BAX
GO:0006942	regulation of striated muscle contraction	6/149	97/18670	0.000127	0.000463	0.000169	NOS1/KCNQ1/CACNA1C/CAV1/SCN10A/SCN5A

GO:1903364	positive regulation of cellular protein catabolic process	7/149	140/18670	0.000133	0.000483	0.000177	APOE/NFE2L2/GSK3B/HSP90AA1/FMR1/CAV1/AKT1
GO:0010464	regulation of mesenchymal cell proliferation	4/149	33/18670	0.000133	0.000483	0.000177	SOX9/CTNNB1/MYC/STAT1
GO:0051204	protein insertion into mitochondrial membrane	4/149	33/18670	0.000133	0.000483	0.000177	BCL2/CASP8/TP53/BAX
GO:0060603	mammary gland duct morphogenesis	4/149	33/18670	0.000133	0.000483	0.000177	ESR1/TGFB1/SRC/PGR
GO:1901186	positive regulation of ERBB signaling pathway	4/149	33/18670	0.000133	0.000483	0.000177	ADRA2A/FASLG/AKT1/MMP9
GO:2000758	positive regulation of peptidyl-lysine acetylation	4/149	33/18670	0.000133	0.000483	0.000177	NOS1/TGFB1/IL1B/SNAI2
GO:0010745	negative regulation of macrophage derived foam cell differentiation	3/149	13/18670	0.000134	0.000484	0.000177	PPARG/CRP/NFKBIA
GO:0031223	auditory behavior	3/149	13/18670	0.000134	0.000484	0.000177	DRD2/SHANK3/NRXN1
GO:0031650	regulation of heat generation	3/149	13/18670	0.000134	0.000484	0.000177	TNF/PTGS2/IL1B
GO:0034374	low-density lipoprotein particle remodeling	3/149	13/18670	0.000134	0.000484	0.000177	PLA2G2A/APOE/MPO
GO:0050872	white fat cell differentiation	3/149	13/18670	0.000134	0.000484	0.000177	PRDM16/PPARG/SNAI2
GO:0090594	inflammatory response to wounding	3/149	13/18670	0.000134	0.000484	0.000177	IL1A/TGFB1/HMOX1
GO:1902947	regulation of tau-protein kinase activity	3/149	13/18670	0.000134	0.000484	0.000177	HSP90AA1/IFNG/RB1
GO:0050764	regulation of phagocytosis	6/149	98/18670	0.000134	0.000484	0.000177	PPARG/TNF/TGFB1/SOD1/IFNG/IL1B
GO:0034248	regulation of cellular amide metabolic process	13/149	483/18670	0.000135	0.000487	0.000178	IGF1/APOE/NFE2L2/PLA2G6/TNF/SNCA/IL6/CASP3/FMR1/MAPK1/ERBB2/IFNG/AKT1

GO:0055025	positive regulation of cardiac muscle tissue development	5/149	62/18670	0.000136	0.000488	0.000179	IGF1/TGFB1/MAPK1/FGF2/NOTCH1
GO:1902686	mitochondrial outer membrane permeabilization involved in programmed cell death	5/149	62/18670	0.000136	0.000488	0.000179	BCL2/CASP8/GSK3B/TP53/BAX
GO:0022604	regulation of cell morphogenesis	13/149	484/18670	0.000138	0.000496	0.000182	APOE/RET/MAPT/F2/SHANK3/GSK3B/KDR/FMR1/GRIN1/ICAM1/ABL1/SRC/SPARC
GO:0070585	protein localization to mitochondrion	7/149	141/18670	0.000139	0.000498	0.000182	BCL2/CASP8/MAPT/HSP90AA1/TP53/AKT1/BAX
GO:0034329	cell junction assembly	9/149	241/18670	0.000139	0.0005	0.000183	BCL2/TNF/CTNNB1/KDR/GPBAR1/CAV1/ABL1/SRC/SNAI2
GO:0038093	Fc receptor signaling pathway	9/149	241/18670	0.000139	0.0005	0.000183	PLA2G6/HSP90AA1/MAPK1/FOS/ABL1/SRC/PIK3R1/NFKB1/JUN
GO:0007588	excretion	5/149	63/18670	0.000147	0.000524	0.000192	DRD2/AVPR2/TRPV1/HMOX1/AVP
GO:0046622	positive regulation of organ growth	5/149	63/18670	0.000147	0.000524	0.000192	IGF1/MAPK1/FGF2/AKT1/NOTCH1
GO:1904377	positive regulation of protein localization to cell periphery	5/149	63/18670	0.000147	0.000524	0.000192	TNF/EGFR/IFNG/AKT1/PIK3R1
GO:0007435	salivary gland morphogenesis	4/149	34/18670	0.00015	0.000533	0.000195	TNF/TGFB1/EGFR/SNAI2
GO:0010765	positive regulation of sodium ion transport	4/149	34/18670	0.00015	0.000533	0.000195	NOS1/DRD4/AKT1/SCN5A
GO:0032660	regulation of interleukin-17 production	4/149	34/18670	0.00015	0.000533	0.000195	TGFB1/IL6/IL2/IFNG
GO:0045066	regulatory T cell differentiation	4/149	34/18670	0.00015	0.000533	0.000195	IRF1/TGFB1/IL2/IFNG
GO:0071868	cellular response to monoamine stimulus	4/149	34/18670	0.00015	0.000533	0.000195	SNCA/KCNQ1/MAPK1/ABL1
GO:0071870	cellular response to catecholamine stimulus	4/149	34/18670	0.00015	0.000533	0.000195	SNCA/KCNQ1/MAPK1/ABL1

GO:0098664	G protein-coupled serotonin receptor signaling pathway	4/149	34/18670	0.00015	0.000533	0.000195	HTR1A/DRD4/CHRM3/HTR2A
GO:0042594	response to starvation	8/149	191/18670	0.000151	0.000536	0.000196	FAS/BCL2/PPARG/NFE2L2/MAPK1/TP53/CDKN1A/JUN
GO:0006164	purine nucleotide biosynthetic process	10/149	300/18670	0.000151	0.000536	0.000196	IGF1/NOS2/NOS1/SNCA/TGFB1/PARP1/INS/IFNG/TP53/HTR2A
GO:0035794	positive regulation of mitochondrial membrane permeability	5/149	64/18670	0.000158	0.000559	0.000205	BCL2/CASP8/GSK3B/TP53/BAX
GO:0045670	regulation of osteoclast differentiation	5/149	64/18670	0.000158	0.000559	0.000205	TNF/CTNNB1/IFNG/FOS/PIK3R1
GO:2000378	negative regulation of reactive oxygen species metabolic process	5/149	64/18670	0.000158	0.000559	0.000205	BCL2/INS/IL10/CAV1/TP53
GO:0071322	cellular response to carbohydrate stimulus	7/149	144/18670	0.000158	0.000559	0.000205	LEP/ADRA2A/OPRK1/PLA2G6/HMGCR/ICAM1/CFTR
GO:0007631	feeding behavior	6/149	101/18670	0.000159	0.000561	0.000205	LEP/OPRK1/DRD2/INS/GRIN1/FOS
GO:0045861	negative regulation of proteolysis	11/149	363/18670	0.000163	0.000575	0.000211	F2/PTGS2/SNCA/INS/SERPINH1/IL10/SRC/TP53/AKT1/AVP/MMP9
GO:0002822	regulation of adaptive immune response based on somatic recombination of immune receptors built from immunoglobulin superfamily domains	7/149	145/18670	0.000165	0.000582	0.000213	TNF/TGFB1/IL6/IL10/IL2/JAK3/IL1B
GO:0007605	sensory perception of sound	7/149	145/18670	0.000165	0.000582	0.000213	COL1A1/THRB/KCNQ1/CASP3/SOD1/ICAM1/SNAI2
GO:0000079	regulation of cyclin-dependent protein serine/threonine kinase activity	6/149	102/18670	0.000168	0.00059	0.000216	CCND1/CASP3/EGFR/SRC/CDKN1A/AKT1
GO:0060326	cell chemotaxis	10/149	304/18670	0.000168	0.00059	0.000216	MET/CXCL8/SLC12A2/S100A8/IL6/KDR/IL10/FGF2/IL1B/NOTCH1

GO:0001893	maternal placenta development	4/149	35/18670	0.000168	0.00059	0.000216	PTGS2/MAPK1/NODAL/AKT1
GO:0051954	positive regulation of amine transport	4/149	35/18670	0.000168	0.00059	0.000216	OPRK1/DRD2/DRD4/AVP
GO:0060251	regulation of glial cell proliferation	4/149	35/18670	0.000168	0.00059	0.000216	TNF/IL6/IL1B/NOTCH1
GO:0071312	cellular response to alkaloid	4/149	35/18670	0.000168	0.00059	0.000216	TRPV1/CASP3/ICAM1/CACNA1S
GO:1905332	positive regulation of morphogenesis of an epithelium	4/149	35/18670	0.000168	0.00059	0.000216	SOX9/CTNNB1/TGFB1/ABL1
GO:0010917	negative regulation of mitochondrial membrane potential	3/149	14/18670	0.00017	0.000591	0.000216	MAPT/TRPV1/BAX
GO:0030213	hyaluronan biosynthetic process	3/149	14/18670	0.00017	0.000591	0.000216	TGFB1/NFKB1/IL1B
GO:0032310	prostaglandin secretion	3/149	14/18670	0.00017	0.000591	0.000216	NOS2/LEP/IL1B
GO:0036295	cellular response to increased oxygen levels	3/149	14/18670	0.00017	0.000591	0.000216	FAS/PPARG/CAV1
GO:0045591	positive regulation of regulatory T cell differentiation	3/149	14/18670	0.00017	0.000591	0.000216	TGFB1/IL2/IFNG
GO:0045651	positive regulation of macrophage differentiation	3/149	14/18670	0.00017	0.000591	0.000216	CASP8/TGFB1/RB1
GO:0055064	chloride ion homeostasis	3/149	14/18670	0.00017	0.000591	0.000216	SLC12A2/FASLG/SLC12A1
GO:1900452	regulation of long-term synaptic depression	3/149	14/18670	0.00017	0.000591	0.000216	MAPT/SHANK3/FMR1
GO:1901386	negative regulation of voltage-gated calcium channel activity	3/149	14/18670	0.00017	0.000591	0.000216	DRD2/FMR1/DRD4
GO:1903054	negative regulation of extracellular matrix organization	3/149	14/18670	0.00017	0.000591	0.000216	DPP4/TGFB1/NOTCH1

GO:2001279	regulation of unsaturated fatty acid biosynthetic process	3/149	14/18670	0.00017	0.000591	0.000216	PTGS2/IL1B/AVP
GO:0006940	regulation of smooth muscle contraction	5/149	65/18670	0.00017	0.000591	0.000216	ADRA2A/PTGS2/SOD1/CAV1/CHRM3
GO:0051250	negative regulation of lymphocyte activation	7/149	146/18670	0.000172	0.000598	0.000219	IRF1/TGFB1/CASP3/IL10/IL2/ERBB2/JAK3
GO:0006644	phospholipid metabolic process	12/149	430/18670	0.000177	0.000613	0.000224	PLA2G2A/PON1/PLA2G6/P2RY12/SNCA/TGFB1/NKX2-1/ACHE/FGF2/SRC/PIK3R1/HTR2A
GO:0032091	negative regulation of protein binding	6/149	103/18670	0.000177	0.000613	0.000224	MET/GSK3B/IL10/CAV1/AKT1/BAX
GO:0002685	regulation of leukocyte migration	8/149	196/18670	0.00018	0.000624	0.000228	CXCL8/TNF/P2RY12/TGFB1/IL6/HMOX1/ICAM1/AKT1
GO:0040014	regulation of multicellular organism growth	5/149	66/18670	0.000183	0.000632	0.000231	IGF1/BCL2/DRD2/IGF2/SOD1
GO:1902108	regulation of mitochondrial membrane permeability involved in apoptotic process	5/149	66/18670	0.000183	0.000632	0.000231	BCL2/CASP8/GSK3B/TP53/BAX
GO:1905710	positive regulation of membrane permeability	5/149	66/18670	0.000183	0.000632	0.000231	BCL2/CASP8/GSK3B/TP53/BAX
GO:0014812	muscle cell migration	6/149	104/18670	0.000186	0.000643	0.000235	IGF1/PLAU/BCL2/NFE2L2/SRC/IGFBP3
GO:0032414	positive regulation of ion transmembrane transporter activity	6/149	104/18670	0.000186	0.000643	0.000235	HTR3A/ABCB1/SHANK3/IFNG/DRD4/CFTR
GO:0009749	response to glucose	8/149	197/18670	0.000187	0.000643	0.000235	ADRA2A/OPRK1/PLA2G6/TGFB1/CASP3/HMGCR/ICAM1/CFTR
GO:0051592	response to calcium ion	7/149	148/18670	0.000187	0.000645	0.000236	CCND1/EGFR/FOS/CAV1/SPARC/JUN/SCN5A
GO:0051973	positive regulation of telomerase activity	4/149	36/18670	0.000188	0.000646	0.000236	CTNNB1/MYC/HSP90AA1/MAPK1
GO:0097242	amyloid-beta clearance	4/149	36/18670	0.000188	0.000646	0.000236	APOE/TNF/HMGCR/IFNG

GO:1901020	negative regulation of calcium ion transmembrane transporter activity	4/149	36/18670	0.000188	0.000646	0.000236	ADRA2A/DRD2/FMR1/DRD4
GO:2001171	positive regulation of ATP biosynthetic process	4/149	36/18670	0.000188	0.000646	0.000236	IGF1/INS/IFNG/HTR2A
GO:0002706	regulation of lymphocyte mediated immunity	7/149	149/18670	0.000195	0.000668	0.000245	LEP/TNF/TGFB1/IL6/IL10/IL2/IL1B
GO:0008277	regulation of G protein-coupled receptor signaling pathway	7/149	149/18670	0.000195	0.000668	0.000245	MET/NOS1/ALK/CXCL8/DRD2/F2/SNCA
GO:0009267	cellular response to starvation	7/149	149/18670	0.000195	0.000668	0.000245	FAS/BCL2/NFE2L2/MAPK1/TP53/CDKN1A/JUN
GO:0050773	regulation of dendrite development	7/149	149/18670	0.000195	0.000668	0.000245	ALK/APOE/SHANK3/GSK3B/FMR1/IL2/GRIN1
GO:0034341	response to interferon-gamma	8/149	199/18670	0.0002	0.000683	0.00025	NOS2/IRF1/PPARG/SNCA/STAT1/IFNG/ICAM1/TP53
GO:0035966	response to topologically incorrect protein	8/149	199/18670	0.0002	0.000683	0.00025	CXCL8/NFE2L2/CCND1/HSP90AA1/SERPINH1/PIK3R1/F12/BAX
GO:0008203	cholesterol metabolic process	7/149	150/18670	0.000203	0.000694	0.000254	LEP/APOE/PON1/HMGCR/SOD1/CFTR/CAT
GO:0016331	morphogenesis of embryonic epithelium	7/149	150/18670	0.000203	0.000694	0.000254	RET/SOX9/CTNNB1/TGFB1/CASP3/NODAL/ABL1
GO:0002286	T cell activation involved in immune response	6/149	106/18670	0.000207	0.000704	0.000258	IL6/IL2/IFNG/ICAM1/TP53/JAK3
GO:1901989	positive regulation of cell cycle phase transition	6/149	106/18670	0.000207	0.000704	0.000258	CYP1A1/CCND1/TGFB1/EGFR/RB1/AKT1
GO:1904029	regulation of cyclin-dependent protein kinase activity	6/149	106/18670	0.000207	0.000704	0.000258	CCND1/CASP3/EGFR/SRC/CDKN1A/AKT1
GO:0007431	salivary gland development	4/149	37/18670	0.00021	0.000711	0.00026	TNF/TGFB1/EGFR/SNAI2
GO:0033280	response to vitamin D	4/149	37/18670	0.00021	0.000711	0.00026	PTGS2/TGFB1/SNAI2/CYP24A1

GO:0045923	positive regulation of fatty acid metabolic process	4/149	37/18670	0.00021	0.000711	0.00026	PPARG/PTGS2/IL1B/AVP
GO:0046006	regulation of activated T cell proliferation	4/149	37/18670	0.00021	0.000711	0.00026	IGF1/CASP3/IGF2/IL2
GO:0007638	mechanosensory behavior	3/149	15/18670	0.000211	0.000711	0.00026	DRD2/SHANK3/NRXN1
GO:0017014	protein nitrosylation	3/149	15/18670	0.000211	0.000711	0.00026	NOS2/NOS1/S100A8
GO:0018119	peptidyl-cysteine S-nitrosylation	3/149	15/18670	0.000211	0.000711	0.00026	NOS2/NOS1/S100A8
GO:0030730	sequestering of triglyceride	3/149	15/18670	0.000211	0.000711	0.00026	PPARG/TNF/IL1B
GO:0032352	positive regulation of hormone metabolic process	3/149	15/18670	0.000211	0.000711	0.00026	TNF/IFNG/IL1B
GO:0033604	negative regulation of catecholamine secretion	3/149	15/18670	0.000211	0.000711	0.00026	ADRA2A/DRD2/P2RY12
GO:0045837	negative regulation of membrane potential	3/149	15/18670	0.000211	0.000711	0.00026	MAPT/TRPV1/BAX
GO:0045898	regulation of RNA polymerase II transcriptional preinitiation complex assembly	3/149	15/18670	0.000211	0.000711	0.00026	ESR1/THRA/TP53
GO:0051770	positive regulation of nitric-oxide synthase biosynthetic process	3/149	15/18670	0.000211	0.000711	0.00026	STAT1/KDR/IFNG
GO:0060134	prepulse inhibition	3/149	15/18670	0.000211	0.000711	0.00026	DRD2/NRXN1/GRIN1
GO:1900119	positive regulation of execution phase of apoptosis	3/149	15/18670	0.000211	0.000711	0.00026	IL6/TP53/BAX
GO:2001028	positive regulation of endothelial cell chemotaxis	3/149	15/18670	0.000211	0.000711	0.00026	MET/KDR/FGF2

GO:0072522	purine-containing compound biosynthetic process	10/149	313/18670	0.000213	0.000715	0.000262	IGF1/NOS2/NOS1/SNCA/TGFB1/PARP1/INS/IFNG/TP53/HTR2A
GO:0001678	cellular glucose homeostasis	7/149	152/18670	0.000221	0.000741	0.000271	ADRA2A/OPRK1/PLA2G6/HMGCR/ICAM1/CFTR/PIK3R1
GO:0030902	hindbrain development	7/149	152/18670	0.000221	0.000741	0.000271	SLC6A4/BCL2/CTNNB1/GRIN1/ABL1/TP53/SCN5A
GO:0032526	response to retinoic acid	6/149	108/18670	0.000229	0.000768	0.000281	SLC6A4/LEP/COL1A1/PPARG/RET/SOX9
GO:0006898	receptor-mediated endocytosis	10/149	316/18670	0.00023	0.00077	0.000282	APOE/CXCL8/DRD2/SNCA/ACHE/HSP90AA1/FMR1/CAV1/DRD4/SPARC
GO:0032094	response to food	4/149	38/18670	0.000233	0.000774	0.000283	LEP/CYP1A1/MPO/AKT1
GO:0032350	regulation of hormone metabolic process	4/149	38/18670	0.000233	0.000774	0.000283	TNF/IFNG/NFKB1/IL1B
GO:0032620	interleukin-17 production	4/149	38/18670	0.000233	0.000774	0.000283	TGFB1/IL6/IL2/IFNG
GO:0048009	insulin-like growth factor receptor signaling pathway	4/149	38/18670	0.000233	0.000774	0.000283	IGF1/AKT1/PIK3R1/IGFBP3
GO:0051590	positive regulation of neurotransmitter transport	4/149	38/18670	0.000233	0.000774	0.000283	SLC6A4/DRD2/SNCA/DRD4
GO:0071867	response to monoamine	4/149	38/18670	0.000233	0.000774	0.000283	SNCA/KCNQ1/MAPK1/ABL1
GO:0071869	response to catecholamine	4/149	38/18670	0.000233	0.000774	0.000283	SNCA/KCNQ1/MAPK1/ABL1
GO:0086005	ventricular cardiac muscle cell action potential	4/149	38/18670	0.000233	0.000774	0.000283	KCNQ1/CACNA1C/CAV1/SCN5A
GO:0086091	regulation of heart rate by cardiac conduction	4/149	38/18670	0.000233	0.000774	0.000283	KCNQ1/CACNA1C/CAV1/SCN5A
GO:1904706	negative regulation of vascular smooth muscle cell proliferation	4/149	38/18670	0.000233	0.000774	0.000283	PPARG/HMOX1/IL10/CDKN1A

GO:1905898	positive regulation of response to endoplasmic reticulum stress	4/149	38/18670	0.000233	0.000774	0.000283	NFE2L2/CAV1/PIK3R1/BAX
GO:0017156	calcium ion regulated exocytosis	7/149	154/18670	0.000239	0.000794	0.000291	ADRA2A/DRD2/SNCA/GSK3B/FMR1/HTR2A/NOTCH1
GO:0021761	limbic system development	6/149	109/18670	0.000241	0.000799	0.000292	ALK/DRD2/NKX2-1/GSK3B/CASP3/BAX
GO:0051149	positive regulation of muscle cell differentiation	6/149	109/18670	0.000241	0.000799	0.000292	IGF1/BCL2/CTNNB1/TGFB1/ABL1/TCF3
GO:0033077	T cell differentiation in thymus	5/149	70/18670	0.000241	0.000799	0.000292	BCL2/CTNNB1/SOD1/ERBB2/TP53
GO:0050766	positive regulation of phagocytosis	5/149	70/18670	0.000241	0.000799	0.000292	PPARG/TNF/SOD1/IFNG/IL1B
GO:0060395	SMAD protein signal transduction	5/149	70/18670	0.000241	0.000799	0.000292	TGFB1/PARP1/NODAL/FOS/JUN
GO:0044282	small molecule catabolic process	12/149	445/18670	0.000242	0.000801	0.000293	NOS2/LEP/NOS1/APOE/PON1/CYP1A1/TGFB1/FGF2/TP53/AKT1/CYP24A1/PAH
GO:0033559	unsaturated fatty acid metabolic process	6/149	110/18670	0.000253	0.000836	0.000306	CYP1A1/PTGS2/CYP2E1/IL1B/PTGS1/AVP
GO:0010661	positive regulation of muscle cell apoptotic process	4/149	39/18670	0.000258	0.000849	0.000311	PPARG/HMGCR/IFNG/TP53
GO:0060969	negative regulation of gene silencing	4/149	39/18670	0.000258	0.000849	0.000311	PPARG/ESR1/TGFB1/TP53
GO:0033692	cellular polysaccharide biosynthetic process	5/149	71/18670	0.000258	0.000849	0.000311	IGF1/GSK3B/INS/IGF2/AKT1
GO:1904427	positive regulation of calcium ion transmembrane transport	5/149	71/18670	0.000258	0.000849	0.000311	F2/SNCA/IL13/ABL1/BAX
GO:0010875	positive regulation of cholesterol efflux	3/149	16/18670	0.000258	0.000849	0.000311	APOE/PON1/NFKBIA
GO:0032695	negative regulation of interleukin-12 production	3/149	16/18670	0.000258	0.000849	0.000311	IL10/JAK3/NFKB1

GO:0034116	positive regulation of heterotypic cell-cell adhesion	3/149	16/18670	0.000258	0.000849	0.000311	TNF/IL10/IL1B
GO:0086103	G protein-coupled receptor signaling pathway involved in heart process	3/149	16/18670	0.000258	0.000849	0.000311	NOS1/CAV1/SRC
GO:1902004	positive regulation of amyloid-beta formation	3/149	16/18670	0.000258	0.000849	0.000311	TNF/CASP3/IFNG
GO:0031346	positive regulation of cell projection organization	11/149	383/18670	0.000259	0.000849	0.000311	ALK/APOE/RET/MAPT/NFE2L2/P2RY12/SHANK3/INS/FMR1/IL2/SRC
GO:0098661	inorganic anion transmembrane transport	6/149	111/18670	0.000266	0.000871	0.000319	SLC12A2/GABRG3/SLC12A1/GABRD/CFTR/GABRA3
GO:0050792	regulation of viral process	8/149	208/18670	0.00027	0.000884	0.000323	APOE/BCL2/CXCL8/TNF/STAT1/FMR1/JUN/NOTCH1
GO:0019226	transmission of nerve impulse	5/149	72/18670	0.000275	0.000901	0.00033	FMR1/SOD1/SCN10A/SCN5A/AVP
GO:0060193	positive regulation of lipase activity	5/149	72/18670	0.000275	0.000901	0.00033	P2RY12/ESR1/EGFR/FGF2/HTR2A
GO:0061035	regulation of cartilage development	5/149	72/18670	0.000275	0.000901	0.00033	LEP/SOX9/CTNNB1/TGFB1/SNAI2
GO:0001938	positive regulation of endothelial cell proliferation	6/149	112/18670	0.000279	0.000909	0.000333	KDR/HMOX1/IL10/FGF2/AKT1/JUN
GO:0017158	regulation of calcium ion-dependent exocytosis	6/149	112/18670	0.000279	0.000909	0.000333	ADRA2A/DRD2/GSK3B/FMR1/HTR2A/NOTCH1
GO:0051817	modification of morphology or physiology of other organism involved in symbiotic interaction	6/149	112/18670	0.000279	0.000909	0.000333	APOE/CASP8/F2/TGFB1/FMR1/JUN
GO:0098693	regulation of synaptic vesicle cycle	6/149	112/18670	0.000279	0.000909	0.000333	DRD2/SNCA/NRXN1/GSK3B/FMR1/HTR2A
GO:0014046	dopamine secretion	4/149	40/18670	0.000285	0.000924	0.000338	OPRK1/DRD2/SNCA/HTR2A

GO:0014059	regulation of dopamine secretion	4/149	40/18670	0.000285	0.000924	0.000338	OPRK1/DRD2/SNCA/HTR2A
GO:0044058	regulation of digestive system process	4/149	40/18670	0.000285	0.000924	0.000338	LEP/OPRK1/KCNQ1/TRPV1
GO:0071548	response to dexamethasone	4/149	40/18670	0.000285	0.000924	0.000338	TGFB1/EGFR/ICAM1/CASP9
GO:0072210	metanephric nephron development	4/149	40/18670	0.000285	0.000924	0.000338	RET/SOX9/CTNNB1/STAT1
GO:0090184	positive regulation of kidney development	4/149	40/18670	0.000285	0.000924	0.000338	RET/SOX9/TGFB1/MYC
GO:0098727	maintenance of cell number	7/149	159/18670	0.00029	0.000942	0.000345	IGF1/PRDM16/SOX9/CTNNB1/NODAL/FGF2/NOTCH1
GO:0032609	interferon-gamma production	6/149	113/18670	0.000293	0.000948	0.000347	TNF/AVPR2/IL10/IL2/ABL1/IL1B
GO:0060964	regulation of gene silencing by miRNA	6/149	113/18670	0.000293	0.000948	0.000347	PPARG/ESR1/TGFB1/FMR1/EGFR/TP53
GO:0032945	negative regulation of mononuclear cell proliferation	5/149	73/18670	0.000294	0.00095	0.000348	TGFB1/CASP3/IL10/IL2/ERBB2
GO:0050672	negative regulation of lymphocyte proliferation	5/149	73/18670	0.000294	0.00095	0.000348	TGFB1/CASP3/IL10/IL2/ERBB2
GO:0006754	ATP biosynthetic process	7/149	160/18670	0.000302	0.000975	0.000357	IGF1/TGFB1/PARP1/INS/IFNG/TP53/HTR2A
GO:0014706	striated muscle tissue development	11/149	390/18670	0.000302	0.000975	0.000357	IGF1/BCL2/CTNNB1/TGFB1/HMGCR/MAPK1/FOS/FGF2/CAV1/RB1/NOTCH1
GO:0030218	erythrocyte differentiation	6/149	114/18670	0.000307	0.000989	0.000362	STAT1/CASP3/RB1/THRA/ACVR1B/JAK3
GO:0042752	regulation of circadian rhythm	6/149	114/18670	0.000307	0.000989	0.000362	PPARG/DRD2/NKX2-1/GSK3B/DRD4/TP53
GO:0006590	thyroid hormone generation	3/149	17/18670	0.000312	0.000999	0.000366	DUOX2/TPO/IYD
GO:0010715	regulation of extracellular matrix disassembly	3/149	17/18670	0.000312	0.000999	0.000366	DPP4/TGFB1/IL6
GO:0015732	prostaglandin transport	3/149	17/18670	0.000312	0.000999	0.000366	NOS2/LEP/IL1B

GO:0032740	positive regulation of interleukin-17 production	3/149	17/18670	0.000312	0.000999	0.000366	TGFB1/IL6/IL2
GO:0061323	cell proliferation involved in heart morphogenesis	3/149	17/18670	0.000312	0.000999	0.000366	SOX9/CTNNB1/NOTCH1
GO:0070242	thymocyte apoptotic process	3/149	17/18670	0.000312	0.000999	0.000366	TP53/JAK3/BAX
GO:0090185	negative regulation of kidney development	3/149	17/18670	0.000312	0.000999	0.000366	CTNNB1/STAT1/MMP9
GO:1902931	negative regulation of alcohol biosynthetic process	3/149	17/18670	0.000312	0.000999	0.000366	APOE/SOD1/NFKB1
GO:1905331	negative regulation of morphogenesis of an epithelium	3/149	17/18670	0.000312	0.000999	0.000366	TNF/CTNNB1/STAT1
GO:2000136	regulation of cell proliferation involved in heart morphogenesis	3/149	17/18670	0.000312	0.000999	0.000366	SOX9/CTNNB1/NOTCH1
GO:0005977	glycogen metabolic process	5/149	74/18670	0.000313	0.000999	0.000366	IGF1/GSK3B/INS/IGF2/AKT1
GO:0086001	cardiac muscle cell action potential	5/149	74/18670	0.000313	0.000999	0.000366	KCNQ1/CACNA1C/CAV1/SCN10A/SCN5A
GO:0099601	regulation of neurotransmitter receptor activity	5/149	74/18670	0.000313	0.000999	0.000366	GRIN2B/SHANK3/GRIN1/IFNG/SRC
GO:1900006	positive regulation of dendrite development	5/149	74/18670	0.000313	0.000999	0.000366	ALK/APOE/SHANK3/FMR1/IL2
GO:0030890	positive regulation of B cell proliferation	4/149	41/18670	0.000314	0.000999	0.000366	BCL2/IL13/IL2/CDKN1A
GO:0050873	brown fat cell differentiation	4/149	41/18670	0.000314	0.000999	0.000366	LEP/PRDM16/PTGS2/INS
GO:0061028	establishment of endothelial barrier	4/149	41/18670	0.000314	0.000999	0.000366	TNF/CTNNB1/ICAM1/IL1B
GO:0042742	defense response to bacterium	10/149	330/18670	0.000324	0.001033	0.000378	NOS2/PLA2G2A/CRP/PLA2G6/TNF/MPO/F2/S100A8/IL6/IL10

GO:0006073	cellular glucan metabolic process	5/149	75/18670	0.000333	0.00106	0.000388	IGF1/GSK3B/INS/IGF2/AKT1
GO:0044042	glucan metabolic process	5/149	75/18670	0.000333	0.00106	0.000388	IGF1/GSK3B/INS/IGF2/AKT1
GO:0007569	cell aging	6/149	116/18670	0.000337	0.00107	0.000392	BCL2/SOD1/ICAM1/ABL1/TP53/CDKN1A
GO:0031214	biomineral tissue development	7/149	163/18670	0.000338	0.001072	0.000392	IGF1/LEP/COL1A1/SOX9/PTGS2/TGFB1/NOTCH1
GO:0002639	positive regulation of immunoglobulin production	4/149	42/18670	0.000344	0.001091	0.000399	TGFB1/IL13/IL6/IL2
GO:0010559	regulation of glycoprotein biosynthetic process	4/149	42/18670	0.000344	0.001091	0.000399	IGF1/BCL2/CTNNB1/JAK3
GO:1901031	regulation of response to reactive oxygen species	4/149	42/18670	0.000344	0.001091	0.000399	MET/NFE2L2/TNF/IL10
GO:0002699	positive regulation of immune effector process	8/149	216/18670	0.000348	0.001101	0.000403	TNF/TGFB1/IL13/IL6/HMOX1/IL2/IFNG/IL1B
GO:0006633	fatty acid biosynthetic process	7/149	164/18670	0.000351	0.001108	0.000405	CYP1A1/PTGS2/CYP2E1/IL1B/PTGS1/CYP3A4/AVP
GO:0007088	regulation of mitotic nuclear division	7/149	164/18670	0.000351	0.001108	0.000405	IGF1/IL1A/TGFB1/INS/IGF2/RB1/IL1B
GO:0060147	regulation of posttranscriptional gene silencing	6/149	117/18670	0.000353	0.001114	0.000408	PPARG/ESR1/TGFB1/FMR1/EGFR/TP53
GO:0060966	regulation of gene silencing by RNA	6/149	117/18670	0.000353	0.001114	0.000408	PPARG/ESR1/TGFB1/FMR1/EGFR/TP53
GO:0046902	regulation of mitochondrial membrane permeability	5/149	76/18670	0.000355	0.001118	0.000409	BCL2/CASP8/GSK3B/TP53/BAX
GO:0050920	regulation of chemotaxis	8/149	217/18670	0.000359	0.00113	0.000414	MET/CXCL8/P2RY12/TGFB1/IL6/KDR/FGF2/NOTCH1
GO:0051346	negative regulation of hydrolase activity	12/149	466/18670	0.000367	0.001158	0.000424	NOS1/TNF/PTGS2/SNCA/GSK3B/SERPINH1/ABL1/SRC/TP53/AKT1/AVP/MMP9
GO:0002902	regulation of B cell apoptotic process	3/149	18/18670	0.000372	0.001168	0.000427	IL10/IL2/BAX

GO:00510 23	regulation of immunoglobulin secretion	3/149	18/18670	0.00037 2	0.00116 8	0.00042 7	TNF/IL6/IL2
GO:00702 30	positive regulation of lymphocyte apoptotic process	3/149	18/18670	0.00037 2	0.00116 8	0.00042 7	IL10/TP53/BAX
GO:00720 77	renal vesicle morphogenesis	3/149	18/18670	0.00037 2	0.00116 8	0.00042 7	SOX9/CTNNB1/STAT1
GO:01500 78	positive regulation of neuroinflammatory response	3/149	18/18670	0.00037 2	0.00116 8	0.00042 7	TNF/IL6/IL1B
GO:20006 47	negative regulation of stem cell proliferation	3/149	18/18670	0.00037 2	0.00116 8	0.00042 7	TGFB1/TP53/SNAI2
GO:00022 44	hematopoietic progenitor cell differentiation	7/149	166/18670	0.00037 7	0.00117 8	0.00043 1	BCL2/NFE2L2/TGFB1/ABL1/TCF3/TP53/NOTCH1
GO:00161 25	sterol metabolic process	7/149	166/18670	0.00037 7	0.00117 8	0.00043 1	LEP/APOE/PON1/HMGCR/SOD1/CFTR/CAT
GO:00902 88	negative regulation of cellular response to growth factor stimulus	7/149	166/18670	0.00037 7	0.00117 8	0.00043 1	PRDM16/TGFB1/NKX2-1/CAV1/ABL1/TP53/NOTCH1
GO:00066 92	prostanoid metabolic process	4/149	43/18670	0.00037 7	0.00117 8	0.00043 1	PTGS2/IL1B/PTGS1/AVP
GO:00066 93	prostaglandin metabolic process	4/149	43/18670	0.00037 7	0.00117 8	0.00043 1	PTGS2/IL1B/PTGS1/AVP
GO:00217 95	cerebral cortex cell migration	4/149	43/18670	0.00037 7	0.00117 8	0.00043 1	CTNNB1/P2RY12/NKX2-1/EGFR
GO:19019 85	positive regulation of protein acetylation	4/149	43/18670	0.00037 7	0.00117 8	0.00043 1	NOS1/TGFB1/IL1B/SNAI2
GO:00435 83	ear development	8/149	219/18670	0.00038 1	0.00118 9	0.00043 5	BCL2/SOX9/TGFB1/KCNQ1/MAPK1/SOD1/SPARC/NOTCH1
GO:00083 06	associative learning	5/149	78/18670	0.0004	0.00124 6	0.00045 6	OPRK1/DRD2/HMGCR/GRIN1/FOS
GO:00706 64	negative regulation of leukocyte proliferation	5/149	78/18670	0.0004	0.00124 6	0.00045 6	TGFB1/CASP3/IL10/IL2/ERBB2
GO:00030 14	renal system process	6/149	120/18670	0.00040 4	0.00125 7	0.00046	BCL2/DRD2/AVPR2/KCNQ1/TRPV1/AVP

GO:0006721	terpenoid metabolic process	6/149	120/18670	0.000404	0.001257	0.00046	APOE/CYP1A1/ADH1C/CYP2E1/EGFR/CYP3A4
GO:0007212	dopamine receptor signaling pathway	4/149	44/18670	0.000413	0.001277	0.000467	ALK/DRD2/GSK3B/DRD4
GO:0010463	mesenchymal cell proliferation	4/149	44/18670	0.000413	0.001277	0.000467	SOX9/CTNNB1/MYC/STAT1
GO:0032965	regulation of collagen biosynthetic process	4/149	44/18670	0.000413	0.001277	0.000467	PPARG/F2/TGFB1/IL6
GO:0035850	epithelial cell differentiation involved in kidney development	4/149	44/18670	0.000413	0.001277	0.000467	CTNNB1/STAT1/NOTCH1/MMP9
GO:0045601	regulation of endothelial cell differentiation	4/149	44/18670	0.000413	0.001277	0.000467	TNF/CTNNB1/IL1B/NOTCH1
GO:0060999	positive regulation of dendritic spine development	4/149	44/18670	0.000413	0.001277	0.000467	APOE/SHANK3/FMR1/IL2
GO:0090311	regulation of protein deacetylation	4/149	44/18670	0.000413	0.001277	0.000467	MAPT/TGFB1/IFNG/TP53
GO:0007269	neurotransmitter secretion	7/149	169/18670	0.00042	0.001299	0.000475	CACNA1B/DRD2/SNCA/NRXN1/GSK3B/FMR1/HTR2A
GO:0015698	inorganic anion transport	7/149	169/18670	0.00042	0.001299	0.000475	SLC12A2/ABCB1/GABRG3/SLC12A1/GABRD/CFTR/GABRA3
GO:0001570	vasculogenesis	5/149	79/18670	0.000424	0.001309	0.000479	CTNNB1/TGFB1/KDR/CAV1/NOTCH1
GO:0006110	regulation of glycolytic process	5/149	79/18670	0.000424	0.001309	0.000479	IGF1/INS/IFNG/TP53/HTR2A
GO:0043537	negative regulation of blood vessel endothelial cell migration	5/149	79/18670	0.000424	0.001309	0.000479	APOE/PPARG/TGFB1/FGF2/NOTCH1
GO:0099643	signal release from synapse	7/149	170/18670	0.000435	0.001342	0.000491	CACNA1B/DRD2/SNCA/NRXN1/GSK3B/FMR1/HTR2A
GO:0002544	chronic inflammatory response	3/149	19/18670	0.000439	0.001346	0.000492	TNF/S100A8/IL10
GO:0007625	grooming behavior	3/149	19/18670	0.000439	0.001346	0.000492	DRD2/NRXN1/AVP

GO:0010523	negative regulation of calcium ion transport into cytosol	3/149	19/18670	0.000439	0.001346	0.000492	NOS1/BCL2/TGFB1
GO:0033189	response to vitamin A	3/149	19/18670	0.000439	0.001346	0.000492	CYP1A1/PPARG/CAT
GO:0060252	positive regulation of glial cell proliferation	3/149	19/18670	0.000439	0.001346	0.000492	TNF/IL6/IL1B
GO:0060438	trachea development	3/149	19/18670	0.000439	0.001346	0.000492	SOX9/CTNNB1/MAPK1
GO:0072087	renal vesicle development	3/149	19/18670	0.000439	0.001346	0.000492	SOX9/CTNNB1/STAT1
GO:0090201	negative regulation of release of cytochrome c from mitochondria	3/149	19/18670	0.000439	0.001346	0.000492	IGF1/AKT1/AVP
GO:1902176	negative regulation of oxidative stress-induced intrinsic apoptotic signaling pathway	3/149	19/18670	0.000439	0.001346	0.000492	NFE2L2/INS/AKT1
GO:2000696	regulation of epithelial cell differentiation involved in kidney development	3/149	19/18670	0.000439	0.001346	0.000492	CTNNB1/STAT1/MMP9
GO:0043500	muscle adaptation	6/149	122/18670	0.000441	0.001351	0.000494	IGF1/PARP1/HMOX1/IL1B/GATM/NOTCH1
GO:0031018	endocrine pancreas development	4/149	45/18670	0.00045	0.001372	0.000502	SOX9/GSK3B/IL6/AKT1
GO:0032570	response to progesterone	4/149	45/18670	0.00045	0.001372	0.000502	TGFB1/FOS/CAV1/SRC
GO:0035987	endodermal cell differentiation	4/149	45/18670	0.00045	0.001372	0.000502	MMP2/CTNNB1/NODAL/MMP9
GO:0045581	negative regulation of T cell differentiation	4/149	45/18670	0.00045	0.001372	0.000502	IRF1/IL2/ERBB2/JAK3
GO:0090278	negative regulation of peptide hormone secretion	4/149	45/18670	0.00045	0.001372	0.000502	LEP/ADRA2A/DRD2/HMGCR

GO:0009206	purine ribonucleoside triphosphate biosynthetic process	7/149	171/18670	0.000451	0.001374	0.000503	IGF1/TGFB1/PARP1/INS/IFNG/TP53/HTR2A
GO:0007160	cell-matrix adhesion	8/149	225/18670	0.000456	0.001389	0.000508	PLAU/BCL2/CTNNB1/GSK3B/KDR/ABL1/SRC/PIK3R1
GO:0009145	purine nucleoside triphosphate biosynthetic process	7/149	172/18670	0.000467	0.001421	0.00052	IGF1/TGFB1/PARP1/INS/IFNG/TP53/HTR2A
GO:0030512	negative regulation of transforming growth factor beta receptor signaling pathway	5/149	81/18670	0.000476	0.001446	0.000529	PRDM16/TGFB1/NKX2-1/CAV1/TP53
GO:0030811	regulation of nucleotide catabolic process	5/149	81/18670	0.000476	0.001446	0.000529	IGF1/INS/IFNG/TP53/HTR2A
GO:0032204	regulation of telomere maintenance	5/149	81/18670	0.000476	0.001446	0.000529	CTNNB1/MYC/PARP1/MAPK1/SRC
GO:0002437	inflammatory response to antigenic stimulus	4/149	46/18670	0.00049	0.001482	0.000542	TNF/IL10/ICAM1/NOTCH1
GO:0006984	ER-nucleus signaling pathway	4/149	46/18670	0.00049	0.001482	0.000542	CXCL8/NFE2L2/GSK3B/TP53
GO:0035196	production of miRNAs involved in gene silencing by miRNA	4/149	46/18670	0.00049	0.001482	0.000542	ESR1/TGFB1/EGFR/TP53
GO:0045776	negative regulation of blood pressure	4/149	46/18670	0.00049	0.001482	0.000542	NOS2/NOS1/DRD2/TRPV1
GO:0060711	labyrinthine layer development	4/149	46/18670	0.00049	0.001482	0.000542	CASP8/IL10/MAPK1/AKT1
GO:0071354	cellular response to interleukin-6	4/149	46/18670	0.00049	0.001482	0.000542	STAT1/IL6/ICAM1/NFKB1
GO:0046486	glycerolipid metabolic process	11/149	414/18670	0.000499	0.001508	0.000552	PLA2G2A/APOE/PON1/PLA2G6/CYP2E1/ACHE/FGF2/CAV1/CAT/PIK3R1/HTR2A
GO:2000134	negative regulation of G1/S transition of mitotic cell cycle	6/149	125/18670	0.000502	0.001517	0.000555	BCL2/CCND1/RB1/TP53/CDKN1A/BAX
GO:0010833	telomere maintenance via telomere lengthening	5/149	82/18670	0.000504	0.00152	0.000556	CTNNB1/HSP90AA1/PARP1/MAPK1/SRC

GO:0030279	negative regulation of ossification	5/149	82/18670	0.000504	0.00152	0.000556	BCL2/SOX9/TNF/TGFB1/NOTCH1
GO:0032769	negative regulation of monooxygenase activity	3/149	20/18670	0.000514	0.00154	0.000564	SNCA/CAV1/NFKB1
GO:0043586	tongue development	3/149	20/18670	0.000514	0.00154	0.000564	PRDM16/CTNNB1/EGFR
GO:0051767	nitric-oxide synthase biosynthetic process	3/149	20/18670	0.000514	0.00154	0.000564	STAT1/KDR/IFNG
GO:0051769	regulation of nitric-oxide synthase biosynthetic process	3/149	20/18670	0.000514	0.00154	0.000564	STAT1/KDR/IFNG
GO:0060602	branch elongation of an epithelium	3/149	20/18670	0.000514	0.00154	0.000564	SOX9/ESR1/TGFB1
GO:0086014	atrial cardiac muscle cell action potential	3/149	20/18670	0.000514	0.00154	0.000564	KCNQ1/CACNA1C/SCN5A
GO:0086026	atrial cardiac muscle cell to AV node cell signaling	3/149	20/18670	0.000514	0.00154	0.000564	KCNQ1/CACNA1C/SCN5A
GO:0086066	atrial cardiac muscle cell to AV node cell communication	3/149	20/18670	0.000514	0.00154	0.000564	KCNQ1/CACNA1C/SCN5A
GO:0097062	dendritic spine maintenance	3/149	20/18670	0.000514	0.00154	0.000564	APOE/GRIN2B/INS
GO:1902993	positive regulation of amyloid precursor protein catabolic process	3/149	20/18670	0.000514	0.00154	0.000564	TNF/CASP3/IFNG
GO:0098656	anion transmembrane transport	9/149	288/18670	0.000518	0.001552	0.000568	SLC12A2/ABCB1/GABRG3/SLC12A1/GABRD/SLC6A8/CFTR/AKT1/GABRA3
GO:0018107	peptidyl-threonine phosphorylation	6/149	126/18670	0.000524	0.001567	0.000573	BCL2/TGFB1/GSK3B/MAPK1/ACVR1B/AKT1
GO:0045667	regulation of osteoblast differentiation	6/149	126/18670	0.000524	0.001567	0.000573	IGF1/TNF/CTNNB1/IL6/SNAI2/NOTCH1
GO:0051260	protein homooligomerization	10/149	351/18670	0.000526	0.001572	0.000575	SLC6A4/APOE/TRPA1/TRPV1/HMOX1/CAV1/SRC/TP53/CAT/BAX
GO:0030574	collagen catabolic process	4/149	47/18670	0.000532	0.001585	0.00058	MMP2/CTSD/MMP1/MMP9

GO:0031952	regulation of protein autophosphorylation	4/149	47/18670	0.000532	0.001585	0.00058	INS/CAV1/SRC/JUN
GO:1903580	positive regulation of ATP metabolic process	4/149	47/18670	0.000532	0.001585	0.00058	IGF1/INS/IFNG/HTR2A
GO:0098659	inorganic cation import across plasma membrane	5/149	83/18670	0.000533	0.001585	0.00058	SLC12A2/TRPV1/ABCC9/IFNG/SLC12A1
GO:0099587	inorganic ion import across plasma membrane	5/149	83/18670	0.000533	0.001585	0.00058	SLC12A2/TRPV1/ABCC9/IFNG/SLC12A1
GO:1903845	negative regulation of cellular response to transforming growth factor beta stimulus	5/149	83/18670	0.000533	0.001585	0.00058	PRDM16/TGFB1/NKX2-1/CAV1/TP53
GO:2000106	regulation of leukocyte apoptotic process	5/149	83/18670	0.000533	0.001585	0.00058	IL10/IL2/TP53/JAK3/BAX
GO:0006986	response to unfolded protein	7/149	176/18670	0.000536	0.001592	0.000583	CXCL8/NFE2L2/CCND1/HSP90AA1/SERPINH1/PIK3R1/BAX
GO:0001959	regulation of cytokine-mediated signaling pathway	7/149	177/18670	0.000554	0.001644	0.000602	CASP8/PPARG/TNF/STAT1/IL6/IFNG/CAV1
GO:0009201	ribonucleoside triphosphate biosynthetic process	7/149	177/18670	0.000554	0.001644	0.000602	IGF1/TGFB1/PARP1/INS/IFNG/TP53/HTR2A
GO:0031589	cell-substrate adhesion	10/149	354/18670	0.000562	0.001667	0.00061	PLAU/COL1A1/BCL2/CTNNB1/GSK3B/KDR/ABL1/SRC/PIK3R1/NOTCH1
GO:0002687	positive regulation of leukocyte migration	6/149	128/18670	0.00057	0.001689	0.000618	CXCL8/TNF/P2RY12/TGFB1/IL6/ICAM1
GO:0030850	prostate gland development	4/149	48/18670	0.000577	0.001704	0.000623	SOX9/CTNNB1/ESR1/NOTCH1
GO:0045912	negative regulation of carbohydrate metabolic process	4/149	48/18670	0.000577	0.001704	0.000623	TGFB1/GSK3B/INS/TP53
GO:0046850	regulation of bone remodeling	4/149	48/18670	0.000577	0.001704	0.000623	LEP/IL6/EGFR/SRC
GO:0055023	positive regulation of cardiac muscle tissue growth	4/149	48/18670	0.000577	0.001704	0.000623	IGF1/MAPK1/FGF2/NOTCH1

GO:0060324	face development	4/149	48/18670	0.000577	0.001704	0.000623	MMP2/COL1A1/TGFB1/MAPK1
GO:0072604	interleukin-6 secretion	4/149	48/18670	0.000577	0.001704	0.000623	NOS2/LEP/TNF/IL1B
GO:0051146	striated muscle cell differentiation	9/149	293/18670	0.000586	0.00173	0.000633	IGF1/NOS1/BCL2/TGFB1/CASP3/IGF2/RB1/AKT1/NOTCH1
GO:0048588	developmental cell growth	8/149	234/18670	0.000591	0.001743	0.000638	IGF1/APOE/MAPT/SOX9/CTNNB1/GSK3B/HSP90AA1/ABL1
GO:0044106	cellular amine metabolic process	6/149	129/18670	0.000594	0.001745	0.000639	SNCA/INS/HTR1A/MAOB/NQO1/DRD4
GO:0010829	negative regulation of glucose transmembrane transport	3/149	21/18670	0.000596	0.001745	0.000639	LEP/TNF/IL1B
GO:0010893	positive regulation of steroid biosynthetic process	3/149	21/18670	0.000596	0.001745	0.000639	TNF/IFNG/IL1B
GO:0030728	ovulation	3/149	21/18670	0.000596	0.001745	0.000639	LEP/PTGS2/PGR
GO:0035813	regulation of renal sodium excretion	3/149	21/18670	0.000596	0.001745	0.000639	DRD2/AVPR2/AVP
GO:0042403	thyroid hormone metabolic process	3/149	21/18670	0.000596	0.001745	0.000639	DUOX2/TPO/IYD
GO:0046827	positive regulation of protein export from nucleus	3/149	21/18670	0.000596	0.001745	0.000639	GSK3B/TP53/IL1B
GO:0071498	cellular response to fluid shear stress	3/149	21/18670	0.000596	0.001745	0.000639	NFE2L2/PTGS2/SRC
GO:1900273	positive regulation of long-term synaptic potentiation	3/149	21/18670	0.000596	0.001745	0.000639	DRD2/SHANK3/INS
GO:1904886	beta-catenin destruction complex disassembly	3/149	21/18670	0.000596	0.001745	0.000639	CTNNB1/GSK3B/CAV1
GO:2000810	regulation of bicellular tight junction assembly	3/149	21/18670	0.000596	0.001745	0.000639	TNF/GPBAR1/SNAI2
GO:0071346	cellular response to interferon-gamma	7/149	180/18670	0.000612	0.001791	0.000655	NOS2/IRF1/PPARG/STAT1/IFNG/ICAM1/TP53

GO:0010712	regulation of collagen metabolic process	4/149	49/18670	0.000624	0.001823	0.000667	PPARG/F2/TGFB1/IL6
GO:0043124	negative regulation of I-kappaB kinase/NF-kappaB signaling	4/149	49/18670	0.000624	0.001823	0.000667	CASP8/ESR1/STAT1/ABL1
GO:1903018	regulation of glycoprotein metabolic process	4/149	49/18670	0.000624	0.001823	0.000667	IGF1/BCL2/CTNNB1/JAK3
GO:0042058	regulation of epidermal growth factor receptor signaling pathway	5/149	86/18670	0.000627	0.001827	0.000669	ADRA2A/FASLG/EGFR/AKT1/MMP9
GO:0048013	ephrin receptor signaling pathway	5/149	86/18670	0.000627	0.001827	0.000669	MMP2/GRIN2B/GRIN1/SRC/MMP9
GO:0090559	regulation of membrane permeability	5/149	86/18670	0.000627	0.001827	0.000669	BCL2/CASP8/GSK3B/TP53/BAX
GO:0046887	positive regulation of hormone secretion	6/149	131/18670	0.000644	0.001873	0.000686	LEP/PLA2G6/DRD2/INS/EGFR/CFTR
GO:1902807	negative regulation of cell cycle G1/S phase transition	6/149	131/18670	0.000644	0.001873	0.000686	BCL2/CCND1/RB1/TP53/CDKN1A/BAX
GO:0030258	lipid modification	8/149	238/18670	0.00066	0.00192	0.000702	LEP/APOE/CYP1A1/PPARG/CYP2E1/AKT1/PIK3R1/CYP3A4
GO:0051607	defense response to virus	8/149	238/18670	0.00066	0.00192	0.000702	IRF1/BCL2/OPRK1/STAT1/IL6/ABCC9/IFNG/IL1B
GO:0042177	negative regulation of protein catabolic process	6/149	132/18670	0.00067	0.001945	0.000712	NOS2/SNCA/HMGCR/INS/IL10/EGFR
GO:0045995	regulation of embryonic development	6/149	132/18670	0.00067	0.001945	0.000712	IGF1/NFE2L2/CTNNB1/IL10/NODAL/NOTCH1
GO:0010975	regulation of neuron projection development	12/149	499/18670	0.000674	0.001947	0.000713	ALK/APOE/RET/MAPT/NFE2L2/SHANK3/GSK3B/FMR1/IL2/GRIN1/ABL1/AKT1
GO:0001706	endoderm formation	4/149	50/18670	0.000675	0.001947	0.000713	MMP2/CTNNB1/NODAL/MMP9
GO:0002931	response to ischemia	4/149	50/18670	0.000675	0.001947	0.000713	BCL2/CAV1/TP53/CASP9
GO:0014009	glial cell proliferation	4/149	50/18670	0.000675	0.001947	0.000713	TNF/IL6/IL1B/NOTCH1

GO:0031113	regulation of microtubule polymerization	4/149	50/18670	0.000675	0.001947	0.000713	MET/MAPT/SNCA/ABL1
GO:0070741	response to interleukin-6	4/149	50/18670	0.000675	0.001947	0.000713	STAT1/IL6/ICAM1/NFKB1
GO:1900087	positive regulation of G1/S transition of mitotic cell cycle	4/149	50/18670	0.000675	0.001947	0.000713	CYP1A1/CCND1/EGFR/AKT1
GO:0009127	purine nucleoside monophosphate biosynthetic process	7/149	183/18670	0.000675	0.001947	0.000713	IGF1/TGFB1/PARP1/INS/IFNG/TP53/HTR2A
GO:0009168	purine ribonucleoside monophosphate biosynthetic process	7/149	183/18670	0.000675	0.001947	0.000713	IGF1/TGFB1/PARP1/INS/IFNG/TP53/HTR2A
GO:0021953	central nervous system neuron differentiation	7/149	183/18670	0.000675	0.001947	0.000713	LEP/MAPT/DRD2/CTNNB1/SHANK3/NKX2-1/HSP90AA1
GO:0048705	skeletal system morphogenesis	8/149	239/18670	0.000679	0.001956	0.000716	MMP2/COL1A1/SOX9/CTNNB1/TGFB1/SERPINH1/NODAL/THRA
GO:0002052	positive regulation of neuroblast proliferation	3/149	22/18670	0.000686	0.001968	0.00072	DRD2/CTNNB1/NOTCH1
GO:0031281	positive regulation of cyclase activity	3/149	22/18670	0.000686	0.001968	0.00072	NOS2/NOS1/CACNA1C
GO:0045649	regulation of macrophage differentiation	3/149	22/18670	0.000686	0.001968	0.00072	CASP8/TGFB1/RB1
GO:0045723	positive regulation of fatty acid biosynthetic process	3/149	22/18670	0.000686	0.001968	0.00072	PTGS2/IL1B/AVP
GO:0048305	immunoglobulin secretion	3/149	22/18670	0.000686	0.001968	0.00072	TNF/IL6/IL2
GO:0060390	regulation of SMAD protein signal transduction	3/149	22/18670	0.000686	0.001968	0.00072	TGFB1/PARP1/NODAL
GO:0090312	positive regulation of protein deacetylation	3/149	22/18670	0.000686	0.001968	0.00072	TGFB1/IFNG/TP53
GO:1901522	positive regulation of transcription from RNA polymerase II promoter	3/149	22/18670	0.000686	0.001968	0.00072	NFE2L2/TP53/NOTCH1

	involved in cellular response to chemical stimulus						
GO:0043470	regulation of carbohydrate catabolic process	5/149	88/18670	0.000697	0.001989	0.000728	IGF1/INS/IFNG/TP53/HTR2A
GO:0045921	positive regulation of exocytosis	5/149	88/18670	0.000697	0.001989	0.000728	PLA2G6/SNCA/IL13/IFNG/CFTR
GO:0106027	neuron projection organization	5/149	88/18670	0.000697	0.001989	0.000728	APOE/GRIN2B/SHANK3/GSK3B/INS
GO:0001508	action potential	6/149	133/18670	0.000697	0.001989	0.000728	KCNQ1/CACNA1C/FMR1/CAV1/SCN10A/SCN5A
GO:0002705	positive regulation of leukocyte mediated immunity	6/149	133/18670	0.000697	0.001989	0.000728	TNF/TGFB1/IL13/IL6/IL2/IL1B
GO:0042770	signal transduction in response to DNA damage	6/149	133/18670	0.000697	0.001989	0.000728	ABL1/TP53/CASP9/CDKN1A/SNAI2/BAX
GO:0071333	cellular response to glucose stimulus	6/149	133/18670	0.000697	0.001989	0.000728	ADRA2A/OPRK1/PLA2G6/HMGCR/ICAM1/CFTR
GO:0043409	negative regulation of MAPK cascade	7/149	184/18670	0.000697	0.001989	0.000728	APOE/MYC/HMGCR/CAV1/ABL1/AKT1/IL1B
GO:0018210	peptidyl-threonine modification	6/149	134/18670	0.000725	0.002066	0.000756	BCL2/TGFB1/GSK3B/MAPK1/ACVR1B/AKT1
GO:0002712	regulation of B cell mediated immunity	4/149	51/18670	0.000728	0.002066	0.000756	TNF/TGFB1/IL10/IL2
GO:0002889	regulation of immunoglobulin mediated immune response	4/149	51/18670	0.000728	0.002066	0.000756	TNF/TGFB1/IL10/IL2
GO:0019369	arachidonic acid metabolic process	4/149	51/18670	0.000728	0.002066	0.000756	CYP1A1/PTGS2/CYP2E1/PTGS1
GO:0031050	dsRNA processing	4/149	51/18670	0.000728	0.002066	0.000756	ESR1/TGFB1/EGFR/TP53
GO:0048641	regulation of skeletal muscle tissue development	4/149	51/18670	0.000728	0.002066	0.000756	BCL2/CTNNB1/TGFB1/HMGCR

GO:00709 18	production of small RNA involved in gene silencing by RNA	4/149	51/18670	0.00072 8	0.00206 6	0.00075 6	ESR1/TGFB1/EGFR/TP53
GO:00713 31	cellular response to hexose stimulus	6/149	135/18670	0.00075 4	0.00214	0.00078 3	ADRA2A/OPRK1/PLA2G6/HMGCR/ICAM1/CFTR
GO:00306 41	regulation of cellular pH	5/149	90/18670	0.00077 1	0.00218 7	0.0008	BCL2/FASLG/MAPK1/CFTR/AVP
GO:00343 33	adherens junction assembly	5/149	90/18670	0.00077 1	0.00218 7	0.0008	BCL2/CTNNB1/KDR/ABL1/SRC
GO:00164 47	somatic recombination of immunoglobulin gene segments	4/149	52/18670	0.00078 3	0.00220 9	0.00080 8	TGFB1/IL10/IL2/TCF3
GO:00604 21	positive regulation of heart growth	4/149	52/18670	0.00078 3	0.00220 9	0.00080 8	IGF1/MAPK1/FGF2/NOTCH1
GO:00721 32	mesenchyme morphogenesis	4/149	52/18670	0.00078 3	0.00220 9	0.00080 8	SOX9/MYC/SNAI2/NOTCH1
GO:00323 31	negative regulation of chondrocyte differentiation	3/149	23/18670	0.00078 5	0.00220 9	0.00080 8	SOX9/CTNNB1/SNAI2
GO:00323 73	positive regulation of sterol transport	3/149	23/18670	0.00078 5	0.00220 9	0.00080 8	APOE/PON1/NFKBIA
GO:00323 76	positive regulation of cholesterol transport	3/149	23/18670	0.00078 5	0.00220 9	0.00080 8	APOE/PON1/NFKBIA
GO:00358 12	renal sodium excretion	3/149	23/18670	0.00078 5	0.00220 9	0.00080 8	DRD2/AVPR2/AVP
GO:00513 49	positive regulation of lyase activity	3/149	23/18670	0.00078 5	0.00220 9	0.00080 8	NOS2/NOS1/CACNA1C
GO:19010 32	negative regulation of response to reactive oxygen species	3/149	23/18670	0.00078 5	0.00220 9	0.00080 8	MET/NFE2L2/IL10
GO:19032 06	negative regulation of hydrogen peroxide-induced cell death	3/149	23/18670	0.00078 5	0.00220 9	0.00080 8	MET/NFE2L2/IL10
GO:20010 39	negative regulation of cellular response to drug	3/149	23/18670	0.00078 5	0.00220 9	0.00080 8	MET/NFE2L2/IL10

GO:0032535	regulation of cellular component size	10/149	370/18670	0.000789	0.00222	0.000812	APOE/RET/MAPT/SLC12A2/SHANK3/GSK3B/SLC12A1/ICAM1/ABL1/AKT1
GO:0009142	nucleoside triphosphate biosynthetic process	7/149	188/18670	0.000791	0.002222	0.000813	IGF1/TGFB1/PARP1/INS/IFNG/TP53/HTR2A
GO:0043902	positive regulation of multi-organism process	7/149	188/18670	0.000791	0.002222	0.000813	NOS2/APOE/FMR1/IFNG/ACVR1B/JUN/NOTCH1
GO:0051783	regulation of nuclear division	7/149	188/18670	0.000791	0.002222	0.000813	IGF1/IL1A/TGFB1/INS/IGF2/RB1/IL1B
GO:0045666	positive regulation of neuron differentiation	10/149	371/18670	0.000806	0.002261	0.000827	ALK/APOE/BCL2/RET/MAPT/NFE2L2/SHANK3/FMR1/IL2/TCF3
GO:0060048	cardiac muscle contraction	6/149	137/18670	0.000814	0.002283	0.000836	NOS1/KCNQ1/CACNA1C/CAV1/SCN10A/SCN5A
GO:0030195	negative regulation of blood coagulation	4/149	53/18670	0.000842	0.002353	0.000861	PLAU/APOE/F2/F12
GO:0031638	zymogen activation	4/149	53/18670	0.000842	0.002353	0.000861	PLAU/CASP8/GGT1/F12
GO:0043331	response to dsRNA	4/149	53/18670	0.000842	0.002353	0.000861	MAPK1/CAV1/NFKB1/NFKBIA
GO:0045744	negative regulation of G protein-coupled receptor signaling pathway	4/149	53/18670	0.000842	0.002353	0.000861	MET/CXCL8/DRD2/SNCA
GO:0060563	neuroepithelial cell differentiation	4/149	53/18670	0.000842	0.002353	0.000861	NODAL/SOD1/ABL1/NOTCH1
GO:0060759	regulation of response to cytokine stimulus	7/149	190/18670	0.000842	0.002353	0.000861	CASP8/PPARG/TNF/STAT1/IL6/IFNG/CAV1
GO:0002433	immune response-regulating cell surface receptor signaling pathway involved in phagocytosis	6/149	139/18670	0.000878	0.00245	0.000897	PLA2G6/HSP90AA1/MAPK1/ABL1/SRC/PIK3R1
GO:0038096	Fc-gamma receptor signaling pathway involved in phagocytosis	6/149	139/18670	0.000878	0.00245	0.000897	PLA2G6/HSP90AA1/MAPK1/ABL1/SRC/PIK3R1
GO:0045926	negative regulation of growth	8/149	249/18670	0.000886	0.00247	0.000904	SLC6A4/BCL2/PPARG/TGFB1/ACVR1B/TP53/CDKN1A/NOTCH1

GO:1901990	regulation of mitotic cell cycle phase transition	11/149	444/18670	0.000887	0.002473	0.000905	BCL2/CYP1A1/CCND1/TGFB1/HSP90AA1/EGFR/RB1/TP53/CDKN1A/AKT1/BAX
GO:0001783	B cell apoptotic process	3/149	24/18670	0.000892	0.002477	0.000906	IL10/IL2/BAX
GO:0042104	positive regulation of activated T cell proliferation	3/149	24/18670	0.000892	0.002477	0.000906	IGF1/IGF2/IL2
GO:0042738	exogenous drug catabolic process	3/149	24/18670	0.000892	0.002477	0.000906	NOS1/CYP2E1/CYP3A4
GO:0044062	regulation of excretion	3/149	24/18670	0.000892	0.002477	0.000906	DRD2/AVPR2/AVP
GO:2000178	negative regulation of neural precursor cell proliferation	3/149	24/18670	0.000892	0.002477	0.000906	SLC6A4/TGFB1/TP53
GO:0032092	positive regulation of protein binding	5/149	93/18670	0.000895	0.002484	0.000909	APOE/GSK3B/CAV1/ABL1/MMP9
GO:1990778	protein localization to cell periphery	9/149	311/18670	0.000895	0.002484	0.000909	TNF/TGFB1/INS/EGFR/GRIN1/IFNG/CAV1/AKT1/PIK3R1
GO:0006636	unsaturated fatty acid biosynthetic process	4/149	54/18670	0.000904	0.002504	0.000916	PTGS2/IL1B/PTGS1/AVP
GO:0061005	cell differentiation involved in kidney development	4/149	54/18670	0.000904	0.002504	0.000916	CTNNB1/STAT1/NOTCH1/MMP9
GO:1900047	negative regulation of hemostasis	4/149	54/18670	0.000904	0.002504	0.000916	PLAU/APOE/F2/F12
GO:0035725	sodium ion transmembrane transport	6/149	140/18670	0.000911	0.002523	0.000923	NOS1/SLC12A2/SLC12A1/DRD4/SCN10A/SCN5A
GO:0002377	immunoglobulin production	7/149	193/18670	0.000923	0.002553	0.000934	TNF/TGFB1/IL13/IL6/IL10/IL2/TCF3
GO:0099504	synaptic vesicle cycle	7/149	194/18670	0.000951	0.00263	0.000962	DRD2/CTNNB1/SNCA/NRXN1/GSK3B/FMR1/HTR2A
GO:0007265	Ras protein signal transduction	11/149	448/18670	0.000954	0.002636	0.000965	IGF1/MET/APOE/ADRA2A/FGF2/ABL1/RB1/TP53/CDKN1A/JUN/NOTCH1
GO:0043030	regulation of macrophage activation	4/149	55/18670	0.000969	0.00267	0.000977	SNCA/IL13/IL6/IL10

GO:0043113	receptor clustering	4/149	55/18670	0.000969	0.00267	0.000977	APOE/TGFB1/SHANK3/NRXN1
GO:0045620	negative regulation of lymphocyte differentiation	4/149	55/18670	0.000969	0.00267	0.000977	IRF1/IL2/ERBB2/JAK3
GO:0098900	regulation of action potential	4/149	55/18670	0.000969	0.00267	0.000977	CACNA1C/FMR1/CAV1/SCN5A
GO:0038094	Fc-gamma receptor signaling pathway	6/149	142/18670	0.000981	0.002701	0.000988	PLA2G6/HSP90AA1/MAPK1/ABL1/SRC/PIK3R1
GO:0050871	positive regulation of B cell activation	6/149	142/18670	0.000981	0.002701	0.000988	BCL2/TGFB1/IL13/IL6/IL2/CDKN1A
GO:0002702	positive regulation of production of molecular mediator of immune response	5/149	95/18670	0.000985	0.002709	0.000991	TGFB1/IL13/IL6/IL2/IL1B
GO:0045069	regulation of viral genome replication	5/149	95/18670	0.000985	0.002709	0.000991	BCL2/CXCL8/TNF/FMR1/NOTCH1
GO:0007214	gamma-aminobutyric acid signaling pathway	3/149	25/18670	0.001007	0.002756	0.001008	SLC12A2/GABRG3/GABRA3
GO:0031069	hair follicle morphogenesis	3/149	25/18670	0.001007	0.002756	0.001008	BCL2/CTNNB1/NOTCH1
GO:0032461	positive regulation of protein oligomerization	3/149	25/18670	0.001007	0.002756	0.001008	MMP1/TP53/BAX
GO:0045672	positive regulation of osteoclast differentiation	3/149	25/18670	0.001007	0.002756	0.001008	TNF/IFNG/FOS
GO:0060330	regulation of response to interferon-gamma	3/149	25/18670	0.001007	0.002756	0.001008	PPARG/STAT1/IFNG
GO:0060334	regulation of interferon-gamma-mediated signaling pathway	3/149	25/18670	0.001007	0.002756	0.001008	PPARG/STAT1/IFNG
GO:0060571	morphogenesis of an epithelial fold	3/149	25/18670	0.001007	0.002756	0.001008	CTNNB1/EGFR/NODAL
GO:0072273	metanephric nephron morphogenesis	3/149	25/18670	0.001007	0.002756	0.001008	SOX9/CTNNB1/STAT1

GO:0044344	cellular response to fibroblast growth factor stimulus	6/149	143/18670	0.001017	0.002782	0.001018	COL1A1/CXCL8/CTNNB1/SNCA/MAPK1/FGF2
GO:0048010	vascular endothelial growth factor receptor signaling pathway	5/149	96/18670	0.001033	0.002821	0.001032	HSP90AA1/KDR/SRC/PIK3R1/IL1B
GO:0090277	positive regulation of peptide hormone secretion	5/149	96/18670	0.001033	0.002821	0.001032	PLA2G6/DRD2/INS/EGFR/CFTR
GO:0086002	cardiac muscle cell action potential involved in contraction	4/149	56/18670	0.001037	0.00283	0.001036	KCNQ1/CACNA1C/CAV1/SCN5A
GO:0009156	ribonucleoside monophosphate biosynthetic process	7/149	197/18670	0.00104	0.002837	0.001038	IGF1/TGFB1/PARP1/INS/IFNG/TP53/HTR2A
GO:0090596	sensory organ morphogenesis	8/149	256/18670	0.001059	0.002886	0.001056	BCL2/THRB/SOX9/CTNNB1/FASLG/MAPK1/SOD1/BAX
GO:0010596	negative regulation of endothelial cell migration	5/149	97/18670	0.001082	0.002948	0.001079	APOE/PPARG/TGFB1/FGF2/NOTCH1
GO:0002431	Fc receptor mediated stimulatory signaling pathway	6/149	145/18670	0.001093	0.002977	0.001089	PLA2G6/HSP90AA1/MAPK1/ABL1/SRC/PIK3R1
GO:0050819	negative regulation of coagulation	4/149	57/18670	0.001108	0.003012	0.001102	PLAU/APOE/F2/F12
GO:0051055	negative regulation of lipid biosynthetic process	4/149	57/18670	0.001108	0.003012	0.001102	APOE/SOD1/NFKB1/SNAI2
GO:0061178	regulation of insulin secretion involved in cellular response to glucose stimulus	4/149	57/18670	0.001108	0.003012	0.001102	ADRA2A/PLA2G6/HMGCR/CFTR
GO:0002360	T cell lineage commitment	3/149	26/18670	0.001132	0.003039	0.001112	BCL2/IL6/TP53
GO:0006309	apoptotic DNA fragmentation	3/149	26/18670	0.001132	0.003039	0.001112	IL6/CASP3/BAX
GO:0010996	response to auditory stimulus	3/149	26/18670	0.001132	0.003039	0.001112	DRD2/SHANK3/NRXN1

GO:0030194	positive regulation of blood coagulation	3/149	26/18670	0.001132	0.003039	0.001112	NFE2L2/F2/F12
GO:0034114	regulation of heterotypic cell-cell adhesion	3/149	26/18670	0.001132	0.003039	0.001112	TNF/IL10/IL1B
GO:0035640	exploration behavior	3/149	26/18670	0.001132	0.003039	0.001112	DPP4/APOE/HTR1A
GO:0055083	monovalent inorganic anion homeostasis	3/149	26/18670	0.001132	0.003039	0.001112	SLC12A2/FASLG/SLC12A1
GO:0060544	regulation of necroptotic process	3/149	26/18670	0.001132	0.003039	0.001112	CASP8/SLC25A4/CAV1
GO:0060740	prostate gland epithelium morphogenesis	3/149	26/18670	0.001132	0.003039	0.001112	SOX9/ESR1/NOTCH1
GO:1900048	positive regulation of hemostasis	3/149	26/18670	0.001132	0.003039	0.001112	NFE2L2/F2/F12
GO:1900739	regulation of protein insertion into mitochondrial membrane involved in apoptotic signaling pathway	3/149	26/18670	0.001132	0.003039	0.001112	BCL2/CASP8/TP53
GO:1900740	positive regulation of protein insertion into mitochondrial membrane involved in apoptotic signaling pathway	3/149	26/18670	0.001132	0.003039	0.001112	BCL2/CASP8/TP53
GO:1903203	regulation of oxidative stress-induced neuron death	3/149	26/18670	0.001132	0.003039	0.001112	CTNNB1/PARP1/IL10
GO:1903579	negative regulation of ATP metabolic process	3/149	26/18670	0.001132	0.003039	0.001112	SNCA/PARP1/TP53
GO:1903959	regulation of anion transmembrane transport	3/149	26/18670	0.001132	0.003039	0.001112	ABCB1/CFTR/AKT1
GO:0002224	toll-like receptor signaling pathway	6/149	146/18670	0.001133	0.003039	0.001112	IRF1/CASP8/ESR1/S100A8/CAV1/NFKBIA
GO:0035264	multicellular organism growth	6/149	146/18670	0.001133	0.003039	0.001112	IGF1/BCL2/DRD2/IGF2/SOD1/TP53

GO:0006885	regulation of pH	5/149	98/18670	0.001133	0.003039	0.001112	BCL2/FASLG/MAPK1/CFTR/AVP
GO:0031341	regulation of cell killing	5/149	98/18670	0.001133	0.003039	0.001112	NOS2/LEP/IL13/IFNG/ICAM1
GO:0043473	pigmentation	5/149	98/18670	0.001133	0.003039	0.001112	BCL2/DRD2/SPARC/SNAI2/BAX
GO:0050848	regulation of calcium-mediated signaling	5/149	98/18670	0.001133	0.003039	0.001112	IGF1/NOS1/MAPT/TNF/GSK3B
GO:0099175	regulation of postsynapse organization	5/149	98/18670	0.001133	0.003039	0.001112	APOE/GRIN2B/SHANK3/NRXN1/INS
GO:0003007	heart morphogenesis	8/149	259/18670	0.001141	0.003057	0.001119	SOX9/CTNNB1/TGFB1/NODAL/TP53/JUN/SNAI2/NOTCH1
GO:0019359	nicotinamide nucleotide biosynthetic process	6/149	147/18670	0.001173	0.003139	0.001149	IGF1/PTGS2/INS/IFNG/TP53/HTR2A
GO:0019363	pyridine nucleotide biosynthetic process	6/149	147/18670	0.001173	0.003139	0.001149	IGF1/PTGS2/INS/IFNG/TP53/HTR2A
GO:0061351	neural precursor cell proliferation	6/149	147/18670	0.001173	0.003139	0.001149	SLC6A4/DRD2/CTNNB1/TGFB1/TP53/NOTCH1
GO:0071385	cellular response to glucocorticoid stimulus	4/149	58/18670	0.001183	0.003163	0.001158	TGFB1/EGFR/ICAM1/CASP9
GO:0001708	cell fate specification	5/149	99/18670	0.001186	0.003167	0.001159	SOX9/CTNNB1/NODAL/FGF2/NOTCH1
GO:1901570	fatty acid derivative biosynthetic process	5/149	99/18670	0.001186	0.003167	0.001159	PTGS2/GGT1/IL1B/PTGS1/AVP
GO:0002824	positive regulation of adaptive immune response based on somatic recombination of immune receptors built from immunoglobulin superfamily domains	5/149	100/18670	0.00124	0.003304	0.001209	TNF/TGFB1/IL6/IL2/IL1B
GO:0007215	glutamate receptor signaling pathway	5/149	100/18670	0.00124	0.003304	0.001209	GRIN2B/SHANK3/FMR1/GRIN1/IFNG
GO:0055024	regulation of cardiac muscle tissue development	5/149	100/18670	0.00124	0.003304	0.001209	IGF1/TGFB1/MAPK1/FGF2/NOTCH1

GO:0070498	interleukin-1-mediated signaling pathway	5/149	100/18670	0.00124	0.003304	0.001209	IL1A/IL6/NFKB1/IL1B/NFKBIA
GO:0048592	eye morphogenesis	6/149	149/18670	0.001258	0.003349	0.001226	BCL2/THRB/SOX9/CTNNB1/FASLG/BAX
GO:0010656	negative regulation of muscle cell apoptotic process	4/149	59/18670	0.001261	0.00335	0.001226	IGF1/NFE2L2/HMGCR/HMOX1
GO:0042130	negative regulation of T cell proliferation	4/149	59/18670	0.001261	0.00335	0.001226	TGFB1/CASP3/IL10/ERBB2
GO:0001964	startle response	3/149	27/18670	0.001266	0.00335	0.001226	DRD2/NRXN1/GRIN1
GO:0002021	response to dietary excess	3/149	27/18670	0.001266	0.00335	0.001226	LEP/APOE/TRPV1
GO:0006706	steroid catabolic process	3/149	27/18670	0.001266	0.00335	0.001226	APOE/CYP24A1/CYP3A4
GO:0006884	cell volume homeostasis	3/149	27/18670	0.001266	0.00335	0.001226	SLC12A2/SHANK3/SLC12A1
GO:0036475	neuron death in response to oxidative stress	3/149	27/18670	0.001266	0.00335	0.001226	CTNNB1/PARP1/IL10
GO:0048643	positive regulation of skeletal muscle tissue development	3/149	27/18670	0.001266	0.00335	0.001226	BCL2/CTNNB1/HMGCR
GO:0048799	animal organ maturation	3/149	27/18670	0.001266	0.00335	0.001226	IGF1/LEP/RET
GO:0050820	positive regulation of coagulation	3/149	27/18670	0.001266	0.00335	0.001226	NFE2L2/F2/F12
GO:2000191	regulation of fatty acid transport	3/149	27/18670	0.001266	0.00335	0.001226	PLA2G6/AKT1/IL1B
GO:0006473	protein acetylation	7/149	204/18670	0.001273	0.003366	0.001232	NOS1/MAPT/SNCA/TGFB1/GSK3B/IL1B/SNAI2
GO:0010639	negative regulation of organelle organization	10/149	395/18670	0.001292	0.003415	0.00125	IGF1/MET/MAPT/SNCA/SHANK3/PARP1/SRC/TP53/AKT1/AVP
GO:0032649	regulation of interferon-gamma production	5/149	101/18670	0.001296	0.003424	0.001253	TNF/IL10/IL2/ABL1/IL1B
GO:0016485	protein processing	9/149	328/18670	0.001298	0.003425	0.001253	PLAU/CASP8/F2/CASP3/PARP1/GGT1/SRC/IL1B/F12

GO:0071774	response to fibroblast growth factor	6/149	150/18670	0.001302	0.003431	0.001256	COL1A1/CXCL8/CTNNB1/SNCA/MAPK1/FGF2
GO:0072525	pyridine-containing compound biosynthetic process	6/149	150/18670	0.001302	0.003431	0.001256	IGF1/PTGS2/INS/IFNG/TP53/HTR2A
GO:0051604	protein maturation	10/149	397/18670	0.001342	0.003534	0.001293	PLAU/CASP8/F2/CASP3/PARP1/GGT1/SERPINH1/SRC/IL1B/F12
GO:0002294	CD4-positive, alpha-beta T cell differentiation involved in immune response	4/149	60/18670	0.001343	0.003534	0.001293	IL6/IL2/IFNG/JAK3
GO:1902808	positive regulation of cell cycle G1/S phase transition	4/149	60/18670	0.001343	0.003534	0.001293	CYP1A1/CCND1/EGFR/AKT1
GO:0006733	oxidoreduction coenzyme metabolic process	7/149	207/18670	0.001385	0.003639	0.001332	IGF1/PTGS2/HMGCR/INS/IFNG/TP53/HTR2A
GO:0099003	vesicle-mediated transport in synapse	7/149	207/18670	0.001385	0.003639	0.001332	DRD2/CTNNB1/SNCA/GSK3B/FMR1/DRD4/HTR2A
GO:0048489	synaptic vesicle transport	6/149	152/18670	0.001393	0.003657	0.001338	DRD2/CTNNB1/SNCA/GSK3B/FMR1/HTR2A
GO:0097480	establishment of synaptic vesicle localization	6/149	152/18670	0.001393	0.003657	0.001338	DRD2/CTNNB1/SNCA/GSK3B/FMR1/HTR2A
GO:0034368	protein-lipid complex remodeling	3/149	28/18670	0.00141	0.003675	0.001345	PLA2G2A/APOE/MPO
GO:0034369	plasma lipoprotein particle remodeling	3/149	28/18670	0.00141	0.003675	0.001345	PLA2G2A/APOE/MPO
GO:0042730	fibrinolysis	3/149	28/18670	0.00141	0.003675	0.001345	PLAU/F2/F12
GO:0045686	negative regulation of glial cell differentiation	3/149	28/18670	0.00141	0.003675	0.001345	F2/CTNNB1/NOTCH1
GO:0051123	RNA polymerase II preinitiation complex assembly	3/149	28/18670	0.00141	0.003675	0.001345	ESR1/THRA/TP53
GO:0060260	regulation of transcription initiation	3/149	28/18670	0.00141	0.003675	0.001345	ESR1/THRA/TP53

	from RNA polymerase II promoter						
GO:0060512	prostate gland morphogenesis	3/149	28/18670	0.00141	0.003675	0.001345	SOX9/ESR1/NOTCH1
GO:0085029	extracellular matrix assembly	3/149	28/18670	0.00141	0.003675	0.001345	SOX9/TGFB1/NOTCH1
GO:1900543	negative regulation of purine nucleotide metabolic process	3/149	28/18670	0.00141	0.003675	0.001345	SNCA/PARP1/TP53
GO:2000108	positive regulation of leukocyte apoptotic process	3/149	28/18670	0.00141	0.003675	0.001345	IL10/TP53/BAX
GO:2000463	positive regulation of excitatory postsynaptic potential	3/149	28/18670	0.00141	0.003675	0.001345	SHANK3/NRXN1/GRIN1
GO:2001025	positive regulation of response to drug	3/149	28/18670	0.00141	0.003675	0.001345	OPRK1/DRD2/DRD4
GO:0044264	cellular polysaccharide metabolic process	5/149	103/18670	0.001415	0.003686	0.001349	IGF1/GSK3B/INS/IGF2/AKT1
GO:0009124	nucleoside monophosphate biosynthetic process	7/149	208/18670	0.001423	0.003706	0.001356	IGF1/TGFB1/PARP1/INS/IFNG/TP53/HTR2A
GO:0002287	alpha-beta T cell activation involved in immune response	4/149	61/18670	0.001428	0.003713	0.001359	IL6/IL2/IFNG/JAK3
GO:0002293	alpha-beta T cell differentiation involved in immune response	4/149	61/18670	0.001428	0.003713	0.001359	IL6/IL2/IFNG/JAK3
GO:0071384	cellular response to corticosteroid stimulus	4/149	61/18670	0.001428	0.003713	0.001359	TGFB1/EGFR/ICAM1/CASP9
GO:0035303	regulation of dephosphorylation	7/149	209/18670	0.001463	0.003801	0.001391	TNF/DRD2/TGFB1/GSK3B/IFNG/SRC/IGFBP3
GO:0046928	regulation of neurotransmitter secretion	5/149	104/18670	0.001477	0.00383	0.001401	DRD2/SNCA/GSK3B/FMR1/HTR2A

GO:0090100	positive regulation of transmembrane receptor protein serine/threonine kinase signaling pathway	5/149	104/18670	0.001477	0.00383	0.001401	TGFB1/PARP1/NODAL/ACVR1B/NOTCH1
GO:1901800	positive regulation of proteasomal protein catabolic process	5/149	104/18670	0.001477	0.00383	0.001401	NFE2L2/GSK3B/FMR1/CAV1/AKT1
GO:0002562	somatic diversification of immune receptors via germline recombination within a single locus	4/149	62/18670	0.001518	0.003927	0.001437	TGFB1/IL10/IL2/TCF3
GO:0016444	somatic cell DNA recombination	4/149	62/18670	0.001518	0.003927	0.001437	TGFB1/IL10/IL2/TCF3
GO:0045453	bone resorption	4/149	62/18670	0.001518	0.003927	0.001437	CTNNB1/IL6/EGFR/SRC
GO:1904356	regulation of telomere maintenance via telomere lengthening	4/149	62/18670	0.001518	0.003927	0.001437	CTNNB1/PARP1/MAPK1/SRC
GO:0060968	regulation of gene silencing	6/149	155/18670	0.001539	0.00398	0.001456	PPARG/ESR1/TGFB1/FMR1/EGFR/TP53
GO:0002708	positive regulation of lymphocyte mediated immunity	5/149	105/18670	0.001541	0.00398	0.001456	TNF/TGFB1/IL6/IL2/IL1B
GO:0002821	positive regulation of adaptive immune response	5/149	105/18670	0.001541	0.00398	0.001456	TNF/TGFB1/IL6/IL2/IL1B
GO:0010954	positive regulation of protein processing	3/149	29/18670	0.001563	0.004022	0.001472	SRC/IL1B/F12
GO:0034367	protein-containing complex remodeling	3/149	29/18670	0.001563	0.004022	0.001472	PLA2G2A/APOE/MPO
GO:0045980	negative regulation of nucleotide metabolic process	3/149	29/18670	0.001563	0.004022	0.001472	SNCA/PARP1/TP53
GO:0048730	epidermis morphogenesis	3/149	29/18670	0.001563	0.004022	0.001472	BCL2/CTNNB1/NOTCH1

GO:0071280	cellular response to copper ion	3/149	29/18670	0.001563	0.004022	0.001472	CYP1A1/NFE2L2/SNCA
GO:0072606	interleukin-8 secretion	3/149	29/18670	0.001563	0.004022	0.001472	NOS2/LEP/CRP
GO:1900027	regulation of ruffle assembly	3/149	29/18670	0.001563	0.004022	0.001472	P2RY12/ICAM1/CAV1
GO:0031571	mitotic G1 DNA damage checkpoint	4/149	63/18670	0.001611	0.004139	0.001514	CCND1/TP53/CDKN1A/BAX
GO:0044819	mitotic G1/S transition checkpoint	4/149	63/18670	0.001611	0.004139	0.001514	CCND1/TP53/CDKN1A/BAX
GO:0019827	stem cell population maintenance	6/149	157/18670	0.001643	0.004219	0.001544	PRDM16/SOX9/CTNNB1/NODAL/FGF2/NOTCH1
GO:1901987	regulation of cell cycle phase transition	11/149	480/18670	0.001655	0.004248	0.001554	BCL2/CYP1A1/CCND1/TGFB1/HSP90AA1/EGFR/RB1/TP53/CDKN1A/AKT1/BAX
GO:0016054	organic acid catabolic process	8/149	275/18670	0.001667	0.004274	0.001564	NOS2/LEP/NOS1/PON1/TGFB1/FGF2/AKT1/PAH
GO:0046395	carboxylic acid catabolic process	8/149	275/18670	0.001667	0.004274	0.001564	NOS2/LEP/NOS1/PON1/TGFB1/FGF2/AKT1/PAH
GO:0007411	axon guidance	8/149	276/18670	0.001706	0.004365	0.001597	RET/SHANK3/NRXN1/NKX2-1/MAPK1/ERBB2/SRC/PIK3R1
GO:0016445	somatic diversification of immunoglobulins	4/149	64/18670	0.001707	0.004365	0.001597	TGFB1/IL10/IL2/TCF3
GO:0035773	insulin secretion involved in cellular response to glucose stimulus	4/149	64/18670	0.001707	0.004365	0.001597	ADRA2A/PLA2G6/HMGCR/CFTR
GO:0044783	G1 DNA damage checkpoint	4/149	64/18670	0.001707	0.004365	0.001597	CCND1/TP53/CDKN1A/BAX
GO:1900449	regulation of glutamate receptor signaling pathway	4/149	64/18670	0.001707	0.004365	0.001597	GRIN2B/SHANK3/GRIN1/IFNG
GO:0000002	mitochondrial genome maintenance	3/149	30/18670	0.001727	0.004405	0.001612	PARP1/SLC25A4/TP53
GO:0032743	positive regulation of interleukin-2 production	3/149	30/18670	0.001727	0.004405	0.001612	IL1A/ABL1/IL1B

GO:0070168	negative regulation of biomineral tissue development	3/149	30/18670	0.001727	0.004405	0.001612	SOX9/TGFB1/NOTCH1
GO:1903205	regulation of hydrogen peroxide-induced cell death	3/149	30/18670	0.001727	0.004405	0.001612	MET/NFE2L2/IL10
GO:0097485	neuron projection guidance	8/149	277/18670	0.001745	0.004444	0.001626	RET/SHANK3/NRXN1/NKX2-1/MAPK1/ERBB2/SRC/PIK3R1
GO:0030004	cellular monovalent inorganic cation homeostasis	5/149	108/18670	0.001745	0.004444	0.001626	BCL2/FASLG/MAPK1/CFTR/AVP
GO:0071156	regulation of cell cycle arrest	5/149	108/18670	0.001745	0.004444	0.001626	CCND1/TGFB1/TP53/CDKN1A/BAX
GO:0032729	positive regulation of interferon-gamma production	4/149	65/18670	0.001808	0.004602	0.001684	TNF/IL2/ABL1/IL1B
GO:0071804	cellular potassium ion transport	7/149	217/18670	0.001812	0.004607	0.001686	OPRK1/SLC12A2/P2RY12/KCNQ1/ABCC9/SLC12A1/CAV1
GO:0071805	potassium ion transmembrane transport	7/149	217/18670	0.001812	0.004607	0.001686	OPRK1/SLC12A2/P2RY12/KCNQ1/ABCC9/SLC12A1/CAV1
GO:0001676	long-chain fatty acid metabolic process	5/149	109/18670	0.001817	0.004617	0.00169	CYP1A1/PTGS2/CYP2E1/PTGS1/CYP3A4
GO:0032886	regulation of microtubule-based process	7/149	218/18670	0.00186	0.004723	0.001728	MET/MAPT/CTNNB1/SNCA/GSK3B/ERBB2/ABL1
GO:0032635	interleukin-6 production	6/149	161/18670	0.001866	0.004733	0.001732	NOS2/LEP/TNF/IL6/IL10/IL1B
GO:0009152	purine ribonucleotide biosynthetic process	8/149	280/18670	0.001866	0.004733	0.001732	IGF1/SNCA/TGFB1/PARP1/INS/IFNG/TP53/HTR2A
GO:0016101	diterpenoid metabolic process	5/149	110/18670	0.001892	0.004791	0.001753	APOE/CYP1A1/ADH1C/EGFR/CYP3A4
GO:0098739	import across plasma membrane	5/149	110/18670	0.001892	0.004791	0.001753	SLC12A2/TRPV1/ABCC9/IFNG/SLC12A1
GO:0003401	axis elongation	3/149	31/18670	0.001901	0.004791	0.001753	SOX9/ESR1/TGFB1

GO:0007202	activation of phospholipase C activity	3/149	31/18670	0.001901	0.004791	0.001753	P2RY12/EGFR/HTR2A
GO:0010762	regulation of fibroblast migration	3/149	31/18670	0.001901	0.004791	0.001753	TGFB1/FGF2/AKT1
GO:0030431	sleep	3/149	31/18670	0.001901	0.004791	0.001753	DRD2/FOS/HTR2A
GO:0040018	positive regulation of multicellular organism growth	3/149	31/18670	0.001901	0.004791	0.001753	BCL2/DRD2/IGF2
GO:0048710	regulation of astrocyte differentiation	3/149	31/18670	0.001901	0.004791	0.001753	F2/IL6/NOTCH1
GO:0060292	long-term synaptic depression	3/149	31/18670	0.001901	0.004791	0.001753	MAPT/SHANK3/FMR1
GO:0071880	adenylate cyclase-activating adrenergic receptor signaling pathway	3/149	31/18670	0.001901	0.004791	0.001753	NOS1/ADRA2A/DRD2
GO:1903319	positive regulation of protein maturation	3/149	31/18670	0.001901	0.004791	0.001753	SRC/IL1B/F12
GO:0010976	positive regulation of neuron projection development	8/149	281/18670	0.001908	0.004807	0.001759	ALK/APOE/RET/MAPT/NFE2L2/SHANK3/FMR1/IL2
GO:0000723	telomere maintenance	6/149	162/18670	0.001925	0.004848	0.001774	CTNNB1/MYC/HSP90AA1/PARP1/MAPK1/SRC
GO:0022037	metencephalon development	5/149	111/18670	0.001969	0.004954	0.001813	BCL2/GRIN1/ABL1/TP53/SCN5A
GO:0097479	synaptic vesicle localization	6/149	163/18670	0.001986	0.004995	0.001828	DRD2/CTNNB1/SNCA/GSK3B/FMR1/HTR2A
GO:0043588	skin development	10/149	419/18670	0.001996	0.005018	0.001836	MET/COL1A1/BCL2/SOX9/TNF/CTNNB1/CASP3/EGFR/ACVR1B/NOTCH1
GO:0030858	positive regulation of epithelial cell differentiation	4/149	67/18670	0.002022	0.00508	0.001859	SOX9/CTNNB1/IL13/NOTCH1
GO:1903510	mucopolysaccharide metabolic process	5/149	112/18670	0.002048	0.005141	0.001881	TGFB1/FGF2/AKT1/NFKB1/IL1B

GO:1901605	alpha-amino acid metabolic process	7/149	222/18670	0.002061	0.005173	0.001893	NOS2/NOS1/GGT1/SLC6A8/IYD/GATM/PAH
GO:0003338	metanephros morphogenesis	3/149	32/18670	0.002085	0.005206	0.001905	SOX9/CTNNB1/STAT1
GO:0007616	long-term memory	3/149	32/18670	0.002085	0.005206	0.001905	APOE/DRD2/GRIN1
GO:0042311	vasodilation	3/149	32/18670	0.002085	0.005206	0.001905	NOS1/APOE/SOD1
GO:0042573	retinoic acid metabolic process	3/149	32/18670	0.002085	0.005206	0.001905	CYP1A1/ADH1C/CYP3A4
GO:0045745	positive regulation of G protein-coupled receptor signaling pathway	3/149	32/18670	0.002085	0.005206	0.001905	NOS1/DRD2/F2
GO:0046685	response to arsenic-containing substance	3/149	32/18670	0.002085	0.005206	0.001905	CYP1A1/HMOX1/CDKN1A
GO:0055094	response to lipoprotein particle	3/149	32/18670	0.002085	0.005206	0.001905	APOE/PPARG/AKT1
GO:0060325	face morphogenesis	3/149	32/18670	0.002085	0.005206	0.001905	MMP2/COL1A1/TGFB1
GO:1900745	positive regulation of p38MAPK cascade	3/149	32/18670	0.002085	0.005206	0.001905	LEP/OPRK1/IL1B
GO:0002449	lymphocyte mediated immunity	9/149	352/18670	0.002102	0.005243	0.001918	LEP/CRP/TNF/TGFB1/IL6/IL10/IL2/ICAM1/IL1B
GO:1902903	regulation of supramolecular fiber organization	9/149	352/18670	0.002102	0.005243	0.001918	MET/APOE/MAPT/SNCA/SHANK3/ICAM1/ABL1/RB1/PIK3R1
GO:0007093	mitotic cell cycle checkpoint	6/149	165/18670	0.002112	0.005264	0.001926	CCND1/TGFB1/RB1/TP53/CDKN1A/BAX
GO:0002292	T cell differentiation involved in immune response	4/149	68/18670	0.002135	0.005305	0.001941	IL6/IL2/IFNG/JAK3
GO:0010812	negative regulation of cell-substrate adhesion	4/149	68/18670	0.002135	0.005305	0.001941	COL1A1/SRC/PIK3R1/NOTCH1
GO:0050891	multicellular organismal water homeostasis	4/149	68/18670	0.002135	0.005305	0.001941	MET/AVPR2/CFTR/AVP

GO:0050918	positive chemotaxis	4/149	68/18670	0.002135	0.005305	0.001941	MET/CXCL8/KDR/FGF2
GO:0071230	cellular response to amino acid stimulus	4/149	68/18670	0.002135	0.005305	0.001941	MMP2/COL1A1/TNF/EGFR
GO:0071242	cellular response to ammonium ion	4/149	68/18670	0.002135	0.005305	0.001941	CASP3/MAPK1/ABL1/CHRM3
GO:0006475	internal protein amino acid acetylation	6/149	166/18670	0.002177	0.005405	0.001978	NOS1/MAPT/SNCA/TGFB1/IL1B/SNAI2
GO:0010633	negative regulation of epithelial cell migration	5/149	114/18670	0.002212	0.005487	0.002008	APOE/PPARG/TGFB1/FGF2/NOTCH1
GO:0030282	bone mineralization	5/149	114/18670	0.002212	0.005487	0.002008	IGF1/LEP/SOX9/PTGS2/TGFB1
GO:0002446	neutrophil mediated immunity	11/149	499/18670	0.002241	0.005554	0.002032	PLAU/MPO/F2/CTSD/S100A8/IL6/HSP90AA1/MAPK1/CAT/NFKB1/MMP9
GO:0086003	cardiac muscle cell contraction	4/149	69/18670	0.002253	0.005578	0.002041	KCNQ1/CACNA1C/CAV1/SCN5A
GO:2000243	positive regulation of reproductive process	4/149	69/18670	0.002253	0.005578	0.002041	SOX9/CTNNB1/SRC/ACVR1B
GO:0014072	response to isoquinoline alkaloid	3/149	33/18670	0.00228	0.005618	0.002056	OPRK1/DRD2/GRIN1
GO:0016242	negative regulation of macroautophagy	3/149	33/18670	0.00228	0.005618	0.002056	HMOX1/TP53/AKT1
GO:0033028	myeloid cell apoptotic process	3/149	33/18670	0.00228	0.005618	0.002056	BCL2/IL6/THRA
GO:0036003	positive regulation of transcription from RNA polymerase II promoter in response to stress	3/149	33/18670	0.00228	0.005618	0.002056	NFE2L2/TP53/NOTCH1
GO:0043278	response to morphine	3/149	33/18670	0.00228	0.005618	0.002056	OPRK1/DRD2/GRIN1
GO:0045191	regulation of isotype switching	3/149	33/18670	0.00228	0.005618	0.002056	TGFB1/IL10/IL2
GO:0051955	regulation of amino acid transport	3/149	33/18670	0.00228	0.005618	0.002056	LEP/SNCA/AVP
GO:1901890	positive regulation of cell junction assembly	3/149	33/18670	0.00228	0.005618	0.002056	KDR/CAV1/ABL1

GO:2001038	regulation of cellular response to drug	3/149	33/18670	0.00228	0.005618	0.002056	MET/NFE2L2/IL10
GO:0006096	glycolytic process	5/149	115/18670	0.002298	0.005656	0.00207	IGF1/INS/IFNG/TP53/HTR2A
GO:0032612	interleukin-1 production	5/149	115/18670	0.002298	0.005656	0.00207	IGF1/CASP8/IL10/IFNG/IL1B
GO:0001906	cell killing	6/149	168/18670	0.002312	0.005682	0.002079	NOS2/LEP/F2/IL13/IFNG/ICAM1
GO:1990138	neuron projection extension	6/149	168/18670	0.002312	0.005682	0.002079	APOE/MAPT/CTNNB1/GSK3B/HSP90AA1/ABL1
GO:0007004	telomere maintenance via telomerase	4/149	70/18670	0.002375	0.005835	0.002135	CTNNB1/HSP90AA1/MAPK1/SRC
GO:0006757	ATP generation from ADP	5/149	116/18670	0.002387	0.005858	0.002143	IGF1/INS/IFNG/TP53/HTR2A
GO:2000736	regulation of stem cell differentiation	5/149	116/18670	0.002387	0.005858	0.002143	NFE2L2/SOX9/ABL1/TCF3/NOTCH1
GO:0043433	negative regulation of DNA-binding transcription factor activity	6/149	170/18670	0.002452	0.006015	0.002201	ESR1/HMOX1/IL10/RB1/CAT/NFKBIA
GO:0009260	ribonucleotide biosynthetic process	8/149	293/18670	0.002472	0.006061	0.002218	IGF1/SNCA/TGFB1/PARP1/INS/IFNG/TP53/HTR2A
GO:0000737	DNA catabolic process, endonucleolytic	3/149	34/18670	0.002487	0.006066	0.00222	IL6/CASP3/BAX
GO:0019228	neuronal action potential	3/149	34/18670	0.002487	0.006066	0.00222	FMR1/SCN10A/SCN5A
GO:0031128	developmental induction	3/149	34/18670	0.002487	0.006066	0.00222	SOX9/CTNNB1/NKX2-1
GO:0032892	positive regulation of organic acid transport	3/149	34/18670	0.002487	0.006066	0.00222	PLA2G6/IL1B/AVP
GO:0036474	cell death in response to hydrogen peroxide	3/149	34/18670	0.002487	0.006066	0.00222	MET/NFE2L2/IL10
GO:0070884	regulation of calcineurin-NFAT signaling cascade	3/149	34/18670	0.002487	0.006066	0.00222	IGF1/TNF/GSK3B

GO:0071402	cellular response to lipoprotein particle stimulus	3/149	34/18670	0.002487	0.006066	0.00222	APOE/PPARG/AKT1
GO:0071875	adrenergic receptor signaling pathway	3/149	34/18670	0.002487	0.006066	0.00222	NOS1/ADRA2A/DRD2
GO:0106056	regulation of calcineurin-mediated signaling	3/149	34/18670	0.002487	0.006066	0.00222	IGF1/TNF/GSK3B
GO:0002460	adaptive immune response based on somatic recombination of immune receptors built from immunoglobulin superfamily domains	9/149	361/18670	0.002491	0.006073	0.002222	CRP/TNF/TGFB1/IL6/IL10/IL2/ICAM1/JAK3/IL1B
GO:0071479	cellular response to ionizing radiation	4/149	71/18670	0.002501	0.006095	0.00223	TGFB1/TP53/CDKN1A/SNAI2
GO:0002532	production of molecular mediator involved in inflammatory response	4/149	72/18670	0.002632	0.006407	0.002344	NOS2/LEP/F2/INS
GO:1903747	regulation of establishment of protein localization to mitochondrion	4/149	72/18670	0.002632	0.006407	0.002344	BCL2/CASP8/MAPT/TP53
GO:0016079	synaptic vesicle exocytosis	5/149	119/18670	0.002667	0.006484	0.002373	DRD2/SNCA/GSK3B/FMR1/HTR2A
GO:0042866	pyruvate biosynthetic process	5/149	119/18670	0.002667	0.006484	0.002373	IGF1/INS/IFNG/TP53/HTR2A
GO:0016525	negative regulation of angiogenesis	6/149	173/18670	0.002676	0.006502	0.002379	PPARG/CTNNB1/STAT1/FASLG/SPARC/NOTCH1
GO:0016358	dendrite development	7/149	233/18670	0.002703	0.006536	0.002392	ALK/APOE/SHANK3/GSK3B/FMR1/IL2/GRIN1
GO:0003203	endocardial cushion morphogenesis	3/149	35/18670	0.002704	0.006536	0.002392	SOX9/SNAI2/NOTCH1
GO:0030262	apoptotic nuclear changes	3/149	35/18670	0.002704	0.006536	0.002392	IL6/CASP3/BAX
GO:0098801	regulation of renal system process	3/149	35/18670	0.002704	0.006536	0.002392	DRD2/AVPR2/AVP

GO:1901385	regulation of voltage-gated calcium channel activity	3/149	35/18670	0.002704	0.006536	0.002392	DRD2/FMR1/DRD4
GO:0006570	tyrosine metabolic process	2/149	10/18670	0.00273	0.006536	0.002392	IYD/PAH
GO:0007440	foregut morphogenesis	2/149	10/18670	0.00273	0.006536	0.002392	CTNNB1/NOTCH1
GO:0010749	regulation of nitric oxide mediated signal transduction	2/149	10/18670	0.00273	0.006536	0.002392	INS/EGFR
GO:0014041	regulation of neuron maturation	2/149	10/18670	0.00273	0.006536	0.002392	BCL2/RET
GO:0021548	pons development	2/149	10/18670	0.00273	0.006536	0.002392	BCL2/GRIN1
GO:0031284	positive regulation of guanylate cyclase activity	2/149	10/18670	0.00273	0.006536	0.002392	NOS2/NOS1
GO:0032275	luteinizing hormone secretion	2/149	10/18670	0.00273	0.006536	0.002392	LEP/OPRK1
GO:0045348	positive regulation of MHC class II biosynthetic process	2/149	10/18670	0.00273	0.006536	0.002392	IL10/IFNG
GO:0048149	behavioral response to ethanol	2/149	10/18670	0.00273	0.006536	0.002392	DRD2/DRD4
GO:0051901	positive regulation of mitochondrial depolarization	2/149	10/18670	0.00273	0.006536	0.002392	PARP1/KDR
GO:0060068	vagina development	2/149	10/18670	0.00273	0.006536	0.002392	ESR1/BAX
GO:0060346	bone trabecula formation	2/149	10/18670	0.00273	0.006536	0.002392	MMP2/COL1A1
GO:0060371	regulation of atrial cardiac muscle cell membrane depolarization	2/149	10/18670	0.00273	0.006536	0.002392	SCN10A/SCN5A
GO:0060768	regulation of epithelial cell proliferation	2/149	10/18670	0.00273	0.006536	0.002392	CTNNB1/NOTCH1

	involved in prostate gland development						
GO:0061140	lung secretory cell differentiation	2/149	10/18670	0.00273	0.006536	0.002392	NKX2-1/IL13
GO:0070091	glucagon secretion	2/149	10/18670	0.00273	0.006536	0.002392	LEP/IL6
GO:0071104	response to interleukin-9	2/149	10/18670	0.00273	0.006536	0.002392	STAT1/JAK3
GO:0072203	cell proliferation involved in metanephros development	2/149	10/18670	0.00273	0.006536	0.002392	MYC/STAT1
GO:0090154	positive regulation of sphingolipid biosynthetic process	2/149	10/18670	0.00273	0.006536	0.002392	PLA2G6/TNF
GO:0098598	learned vocalization behavior or vocal learning	2/149	10/18670	0.00273	0.006536	0.002392	SHANK3/NRXN1
GO:2000304	positive regulation of ceramide biosynthetic process	2/149	10/18670	0.00273	0.006536	0.002392	PLA2G6/TNF
GO:2001225	regulation of chloride transport	2/149	10/18670	0.00273	0.006536	0.002392	ABCB1/CFTR
GO:0006520	cellular amino acid metabolic process	9/149	366/18670	0.00273	0.006536	0.002392	NOS2/NOS1/GGT1/INS/NQO1/SLC6A8/IYD/GATM/PAH
GO:0002758	innate immune response-activating signal transduction	8/149	298/18670	0.002743	0.006563	0.002402	IRF1/CASP8/ESR1/S100A8/CAV1/SRC/NFKB1/NFKBIA
GO:0006941	striated muscle contraction	6/149	174/18670	0.002753	0.006584	0.002409	NOS1/KCNQ1/CACNA1C/CAV1/SCN10A/SCN5A
GO:0017015	regulation of transforming growth factor beta receptor signaling pathway	5/149	120/18670	0.002765	0.006605	0.002417	PRDM16/TGFB1/NKX2-1/CAV1/TP53
GO:1903052	positive regulation of proteolysis involved in	5/149	120/18670	0.002765	0.006605	0.002417	NFE2L2/GSK3B/FMR1/CAV1/AKT1

	cellular protein catabolic process						
GO:0002312	B cell activation involved in immune response	4/149	73/18670	0.002768	0.006608	0.002418	TGFB1/IL10/IL2/ABL1
GO:0032200	telomere organization	6/149	175/18670	0.002833	0.006756	0.002472	CTNNB1/MYC/HSP90AA1/PARP1/MAPK1/SRC
GO:2000181	negative regulation of blood vessel morphogenesis	6/149	175/18670	0.002833	0.006756	0.002472	PPARG/CTNNB1/STAT1/FASLG/SPARC/NOTCH1
GO:0046390	ribose phosphate biosynthetic process	8/149	300/18670	0.002857	0.006811	0.002492	IGF1/SNCA/TGFB1/PARP1/INS/IFNG/TP53/HTR2A
GO:0030968	endoplasmic reticulum unfolded protein response	5/149	121/18670	0.002866	0.006817	0.002495	CXCL8/NFE2L2/CCND1/PIK3R1/BAX
GO:0043618	regulation of transcription from RNA polymerase II promoter in response to stress	5/149	121/18670	0.002866	0.006817	0.002495	NFE2L2/HMOX1/TP53/JUN/NOTCH1
GO:0046718	viral entry into host cell	5/149	121/18670	0.002866	0.006817	0.002495	DPP4/EGFR/ICAM1/CAV1/HTR2A
GO:0048675	axon extension	5/149	121/18670	0.002866	0.006817	0.002495	APOE/MAPT/GSK3B/HSP90AA1/ABL1
GO:0030104	water homeostasis	4/149	74/18670	0.002908	0.006899	0.002524	MET/AVPR2/CFTR/AVP
GO:0032677	regulation of interleukin-8 production	4/149	74/18670	0.002908	0.006899	0.002524	CRP/TNF/IL10/IL1B
GO:0043367	CD4-positive, alpha-beta T cell differentiation	4/149	74/18670	0.002908	0.006899	0.002524	IL6/IL2/IFNG/JAK3
GO:0051145	smooth muscle cell differentiation	4/149	74/18670	0.002908	0.006899	0.002524	SOX9/CTNNB1/TGFB1/NOTCH1
GO:0060998	regulation of dendritic spine development	4/149	74/18670	0.002908	0.006899	0.002524	APOE/SHANK3/FMR1/IL2
GO:0010769	regulation of cell morphogenesis involved in differentiation	8/149	301/18670	0.002916	0.006914	0.00253	APOE/RET/MAPT/SHANK3/GSK3B/FMR1/GRIN1/ABL1
GO:0030224	monocyte differentiation	3/149	36/18670	0.002933	0.006932	0.002536	PPARG/MYC/JUN

GO:0034390	smooth muscle cell apoptotic process	3/149	36/18670	0.002933	0.006932	0.002536	IGF1/PPARG/IFNG
GO:0034391	regulation of smooth muscle cell apoptotic process	3/149	36/18670	0.002933	0.006932	0.002536	IGF1/PPARG/IFNG
GO:0071392	cellular response to estradiol stimulus	3/149	36/18670	0.002933	0.006932	0.002536	ESR1/IL10/EGFR
GO:1903131	mononuclear cell differentiation	3/149	36/18670	0.002933	0.006932	0.002536	PPARG/MYC/JUN
GO:2000310	regulation of NMDA receptor activity	3/149	36/18670	0.002933	0.006932	0.002536	GRIN2B/GRIN1/IFNG
GO:0019079	viral genome replication	5/149	122/18670	0.00297	0.007008	0.002564	BCL2/CXCL8/TNF/FMR1/NOTCH1
GO:0019730	antimicrobial humoral response	5/149	122/18670	0.00297	0.007008	0.002564	PLA2G2A/CXCL8/PLA2G6/F2/S100A8
GO:1903844	regulation of cellular response to transforming growth factor beta stimulus	5/149	122/18670	0.00297	0.007008	0.002564	PRDM16/TGFB1/NKX2-1/CAV1/TP53
GO:0007416	synapse assembly	6/149	177/18670	0.002996	0.007068	0.002586	DRD2/SNCA/SHANK3/NRXN1/ACHE/GRIN1
GO:0002200	somatic diversification of immune receptors	4/149	75/18670	0.003053	0.007189	0.002631	TGFB1/IL10/IL2/TCF3
GO:0006695	cholesterol biosynthetic process	4/149	75/18670	0.003053	0.007189	0.002631	APOE/HMGCR/SOD1/CFTR
GO:0046173	polyol biosynthetic process	4/149	75/18670	0.003053	0.007189	0.002631	LEP/SNCA/FGF2/CYP3A4
GO:0002062	chondrocyte differentiation	5/149	123/18670	0.003076	0.00724	0.002649	SOX9/CTNNB1/TGFB1/SERPINH1/SNAI2
GO:0090090	negative regulation of canonical Wnt signaling pathway	6/149	178/18670	0.003081	0.007248	0.002652	APOE/SOX9/GSK3B/CAV1/SNAI2/NOTCH1
GO:0046034	ATP metabolic process	8/149	305/18670	0.00316	0.007429	0.002718	IGF1/SNCA/TGFB1/PARP1/INS/IFNG/TP53/HTR2A
GO:0030177	positive regulation of Wnt signaling pathway	6/149	179/18670	0.003167	0.007429	0.002718	COL1A1/EGFR/CAV1/ABL1/SRC/NFKB1

GO:0002714	positive regulation of B cell mediated immunity	3/149	37/18670	0.003173	0.007429	0.002718	TNF/TGFB1/IL2
GO:0002891	positive regulation of immunoglobulin mediated immune response	3/149	37/18670	0.003173	0.007429	0.002718	TNF/TGFB1/IL2
GO:0006921	cellular component disassembly involved in execution phase of apoptosis	3/149	37/18670	0.003173	0.007429	0.002718	IL6/CASP3/BAX
GO:0060323	head morphogenesis	3/149	37/18670	0.003173	0.007429	0.002718	MMP2/COL1A1/TGFB1
GO:0086010	membrane depolarization during action potential	3/149	37/18670	0.003173	0.007429	0.002718	CACNA1C/SCN10A/SCN5A
GO:1903427	negative regulation of reactive oxygen species biosynthetic process	3/149	37/18670	0.003173	0.007429	0.002718	INS/IL10/CAV1
GO:2000273	positive regulation of signaling receptor activity	3/149	37/18670	0.003173	0.007429	0.002718	ADRA2A/SHANK3/IFNG
GO:0045454	cell redox homeostasis	4/149	76/18670	0.003203	0.007487	0.00274	NOS2/NOS1/NFE2L2/MPO
GO:0046785	microtubule polymerization	4/149	76/18670	0.003203	0.007487	0.00274	MET/MAPT/SNCA/ABL1
GO:1902653	secondary alcohol biosynthetic process	4/149	76/18670	0.003203	0.007487	0.00274	APOE/HMGCR/SOD1/CFTR
GO:0003264	regulation of cardioblast proliferation	2/149	11/18670	0.003319	0.007667	0.002806	CTNNB1/NOTCH1
GO:0007195	adenylate cyclase-inhibiting dopamine receptor signaling pathway	2/149	11/18670	0.003319	0.007667	0.002806	DRD2/DRD4
GO:0009629	response to gravity	2/149	11/18670	0.003319	0.007667	0.002806	FOS/SPARC
GO:0014820	tonic smooth muscle contraction	2/149	11/18670	0.003319	0.007667	0.002806	MYLK/HTR2A

GO:0019371	cyclooxygenase pathway	2/149	11/18670	0.003319	0.007667	0.002806	PTGS2/PTGS1
GO:0021892	cerebral cortex GABAergic interneuron differentiation	2/149	11/18670	0.003319	0.007667	0.002806	DRD2/NKX2-1
GO:0032276	regulation of gonadotropin secretion	2/149	11/18670	0.003319	0.007667	0.002806	LEP/OPRK1
GO:0033327	Leydig cell differentiation	2/149	11/18670	0.003319	0.007667	0.002806	CCND1/NKX2-1
GO:0033860	regulation of NAD(P)H oxidase activity	2/149	11/18670	0.003319	0.007667	0.002806	IL13/INS
GO:0034776	response to histamine	2/149	11/18670	0.003319	0.007667	0.002806	DRD2/DRD4
GO:0043619	regulation of transcription from RNA polymerase II promoter in response to oxidative stress	2/149	11/18670	0.003319	0.007667	0.002806	NFE2L2/HMOX1
GO:0045899	positive regulation of RNA polymerase II transcriptional preinitiation complex assembly	2/149	11/18670	0.003319	0.007667	0.002806	ESR1/TP53
GO:0051024	positive regulation of immunoglobulin secretion	2/149	11/18670	0.003319	0.007667	0.002806	IL6/IL2
GO:0060159	regulation of dopamine receptor signaling pathway	2/149	11/18670	0.003319	0.007667	0.002806	ALK/DRD2
GO:0060433	bronchus development	2/149	11/18670	0.003319	0.007667	0.002806	SOX9/IL13
GO:0060439	trachea morphogenesis	2/149	11/18670	0.003319	0.007667	0.002806	CTNNB1/MAPK1
GO:0060525	prostate glandular acinus development	2/149	11/18670	0.003319	0.007667	0.002806	ESR1/NOTCH1

GO:00607<br>36	prostate gland growth	2/149	11/18670	0.00331<br>9	0.00766<br>7	0.00280<br>6	SOX9/ESR1
GO:00608<br>56	establishment of blood-<br>brain barrier	2/149	11/18670	0.00331<br>9	0.00766<br>7	0.00280<br>6	CTNNB1/TRPV1
GO:00613<br>14	Notch signaling involved<br>in heart development	2/149	11/18670	0.00331<br>9	0.00766<br>7	0.00280<br>6	SNAI2/NOTCH1
GO:00718<br>03	positive regulation of<br>podosome assembly	2/149	11/18670	0.00331<br>9	0.00766<br>7	0.00280<br>6	TNF/SRC
GO:00996<br>24	atrial cardiac muscle cell<br>membrane repolarization	2/149	11/18670	0.00331<br>9	0.00766<br>7	0.00280<br>6	KCNQ1/SCN5A
GO:19059<br>41	positive regulation of<br>gonad development	2/149	11/18670	0.00331<br>9	0.00766<br>7	0.00280<br>6	SOX9/SRC
GO:00062<br>78	RNA-dependent DNA<br>biosynthetic process	4/149	77/18670	0.00335<br>8	0.00774	0.00283<br>2	CTNNB1/HSP90AA1/MAPK1/SRC
GO:00083<br>44	adult locomotory<br>behavior	4/149	77/18670	0.00335<br>8	0.00774	0.00283<br>2	DRD2/SNCA/GRIN1/DRD4
GO:00324<br>81	positive regulation of<br>type I interferon<br>production	4/149	77/18670	0.00335<br>8	0.00774	0.00283<br>2	IRF1/CTNNB1/STAT1/NFKB1
GO:00518<br>51	modification by host of<br>symbiont morphology or<br>physiology	4/149	77/18670	0.00335<br>8	0.00774	0.00283<br>2	APOE/F2/FMR1/JUN
GO:00324<br>79	regulation of type I<br>interferon production	5/149	126/18670	0.00341<br>1	0.00785<br>1	0.00287<br>3	IRF1/CTNNB1/STAT1/IL10/NFKB1
GO:00460<br>31	ADP metabolic process	5/149	126/18670	0.00341<br>1	0.00785<br>1	0.00287<br>3	IGF1/INS/IFNG/TP53/HTR2A
GO:00067<br>78	porphyrin-containing<br>compound metabolic<br>process	3/149	38/18670	0.00342<br>5	0.00785<br>1	0.00287<br>3	CYP1A1/HMOX1/FECH
GO:00109<br>39	regulation of necrotic cell<br>death	3/149	38/18670	0.00342<br>5	0.00785<br>1	0.00287<br>3	CASP8/SLC25A4/CAV1
GO:00466<br>76	negative regulation of<br>insulin secretion	3/149	38/18670	0.00342<br>5	0.00785<br>1	0.00287<br>3	ADRA2A/DRD2/HMGCR
GO:00468<br>25	regulation of protein<br>export from nucleus	3/149	38/18670	0.00342<br>5	0.00785<br>1	0.00287<br>3	GSK3B/TP53/IL1B

GO:0050892	intestinal absorption	3/149	38/18670	0.003425	0.007851	0.002873	LEP/ADRA2A/KCNQ1
GO:0060045	positive regulation of cardiac muscle cell proliferation	3/149	38/18670	0.003425	0.007851	0.002873	MAPK1/FGF2/NOTCH1
GO:0060416	response to growth hormone	3/149	38/18670	0.003425	0.007851	0.002873	JAK3/AKT1/PIK3R1
GO:1903523	negative regulation of blood circulation	3/149	38/18670	0.003425	0.007851	0.002873	LEP/TRPV1/IL2
GO:2000279	negative regulation of DNA biosynthetic process	3/149	38/18670	0.003425	0.007851	0.002873	PPARG/SRC/TP53
GO:0021954	central nervous system neuron development	4/149	78/18670	0.003518	0.008038	0.002941	LEP/MAPT/DRD2/HSP90AA1
GO:0033143	regulation of intracellular steroid hormone receptor signaling pathway	4/149	78/18670	0.003518	0.008038	0.002941	ESR1/PARP1/NODAL/SRC
GO:0043407	negative regulation of MAP kinase activity	4/149	78/18670	0.003518	0.008038	0.002941	APOE/HMGCR/CAV1/IL1B
GO:0055021	regulation of cardiac muscle tissue growth	4/149	78/18670	0.003518	0.008038	0.002941	IGF1/MAPK1/FGF2/NOTCH1
GO:0072332	intrinsic apoptotic signaling pathway by p53 class mediator	4/149	78/18670	0.003518	0.008038	0.002941	BCL2/TP53/CDKN1A/BAX
GO:2000300	regulation of synaptic vesicle exocytosis	4/149	78/18670	0.003518	0.008038	0.002941	DRD2/GSK3B/FMR1/HTR2A
GO:0043620	regulation of DNA-templated transcription in response to stress	5/149	127/18670	0.003529	0.008048	0.002945	NFE2L2/HMOX1/TP53/JUN/NOTCH1
GO:0045727	positive regulation of translation	5/149	127/18670	0.003529	0.008048	0.002945	TNF/IL6/FMR1/MAPK1/ERBB2
GO:0038061	NIK/NF-kappaB signaling	6/149	183/18670	0.003531	0.008048	0.002945	ALK/TNF/EGFR/AKT1/IL1B/NFKBIA
GO:0050770	regulation of axonogenesis	6/149	183/18670	0.003531	0.008048	0.002945	APOE/RET/MAPT/GSK3B/GRIN1/ABL1

GO:0061136	regulation of proteasomal protein catabolic process	6/149	183/18670	0.003531	0.008048	0.002945	APOE/NFE2L2/GSK3B/FMR1/CAV1/AKT1
GO:0016570	histone modification	10/149	454/18670	0.003549	0.008086	0.002959	NOS1/PRDM16/CTNNB1/SNCA/TGFB1/FMR1/IGF2/TP53/IL1B/SNAI2
GO:0002576	platelet degranulation	5/149	128/18670	0.003649	0.008305	0.003039	IGF1/TGFB1/IGF2/SOD1/SPARC
GO:0032606	type I interferon production	5/149	128/18670	0.003649	0.008305	0.003039	IRF1/CTNNB1/STAT1/IL10/NFKB1
GO:0031110	regulation of microtubule polymerization or depolymerization	4/149	79/18670	0.003683	0.008353	0.003057	MET/MAPT/SNCA/ABL1
GO:1900034	regulation of cellular response to heat	4/149	79/18670	0.003683	0.008353	0.003057	MAPT/GSK3B/HSP90AA1/MAPK1
GO:0010761	fibroblast migration	3/149	39/18670	0.003689	0.008353	0.003057	TGFB1/FGF2/AKT1
GO:0010874	regulation of cholesterol efflux	3/149	39/18670	0.003689	0.008353	0.003057	APOE/PON1/NFKBIA
GO:0033146	regulation of intracellular estrogen receptor signaling pathway	3/149	39/18670	0.003689	0.008353	0.003057	ESR1/PARP1/SRC
GO:0045911	positive regulation of DNA recombination	3/149	39/18670	0.003689	0.008353	0.003057	TGFB1/PARP1/IL2
GO:0048713	regulation of oligodendrocyte differentiation	3/149	39/18670	0.003689	0.008353	0.003057	PPARG/CTNNB1/NOTCH1
GO:0060306	regulation of membrane repolarization	3/149	39/18670	0.003689	0.008353	0.003057	KCNQ1/CAV1/SCN5A
GO:2000648	positive regulation of stem cell proliferation	3/149	39/18670	0.003689	0.008353	0.003057	DRD2/CTNNB1/NOTCH1
GO:2000826	regulation of heart morphogenesis	3/149	39/18670	0.003689	0.008353	0.003057	SOX9/CTNNB1/NOTCH1
GO:0007369	gastrulation	6/149	185/18670	0.003724	0.008428	0.003084	MMP2/CTNNB1/IL10/NODAL/TP53/MMP9
GO:1903362	regulation of cellular protein catabolic process	7/149	247/18670	0.003729	0.008435	0.003087	APOE/NFE2L2/GSK3B/HSP90AA1/FMR1/CAV1/AKT1

GO:0043543	protein acylation	7/149	248/18670	0.003812	0.008619	0.003154	NOS1/MAPT/SNCA/TGFB1/GSK3B/IL1B/SNAI2
GO:0030308	negative regulation of cell growth	6/149	186/18670	0.003823	0.008635	0.00316	BCL2/PPARG/TGFB1/ACVR1B/TP53/CDKN1A
GO:0070507	regulation of microtubule cytoskeleton organization	6/149	186/18670	0.003823	0.008635	0.00316	MET/MAPT/CTNNB1/SNCA/GSK3B/ABL1
GO:0097061	dendritic spine organization	4/149	80/18670	0.003853	0.008698	0.003183	APOE/GRIN2B/SHANK3/INS
GO:0006638	neutral lipid metabolic process	5/149	130/18670	0.003898	0.008796	0.003219	APOE/CYP2E1/SNCA/CAV1/CAT
GO:1901343	negative regulation of vasculature development	6/149	187/18670	0.003924	0.008818	0.003227	PPARG/CTNNB1/STAT1/FASLG/SPARC/NOTCH1
GO:0002674	negative regulation of acute inflammatory response	2/149	12/18670	0.003962	0.008818	0.003227	PPARG/INS
GO:0003337	mesenchymal to epithelial transition involved in metanephros morphogenesis	2/149	12/18670	0.003962	0.008818	0.003227	CTNNB1/STAT1
GO:0006527	arginine catabolic process	2/149	12/18670	0.003962	0.008818	0.003227	NOS2/NOS1
GO:0006983	ER overload response	2/149	12/18670	0.003962	0.008818	0.003227	GSK3B/TP53
GO:0031282	regulation of guanylate cyclase activity	2/149	12/18670	0.003962	0.008818	0.003227	NOS2/NOS1
GO:0035630	bone mineralization involved in bone maturation	2/149	12/18670	0.003962	0.008818	0.003227	IGF1/LEP
GO:0038110	interleukin-2-mediated signaling pathway	2/149	12/18670	0.003962	0.008818	0.003227	IL2/JAK3
GO:0046541	saliva secretion	2/149	12/18670	0.003962	0.008818	0.003227	OPRK1/CHRM3
GO:0060453	regulation of gastric acid secretion	2/149	12/18670	0.003962	0.008818	0.003227	KCNQ1/TRPV1
GO:0060742	epithelial cell differentiation involved	2/149	12/18670	0.003962	0.008818	0.003227	CTNNB1/NOTCH1

	in prostate gland development						
GO:0061450	trophoblast cell migration	2/149	12/18670	0.003962	0.008818	0.003227	NODAL/ACVR1B
GO:0070243	regulation of thymocyte apoptotic process	2/149	12/18670	0.003962	0.008818	0.003227	TP53/JAK3
GO:0070486	leukocyte aggregation	2/149	12/18670	0.003962	0.008818	0.003227	S100A8/IL1B
GO:0070493	thrombin-activated receptor signaling pathway	2/149	12/18670	0.003962	0.008818	0.003227	MET/SNCA
GO:0072578	neurotransmitter-gated ion channel clustering	2/149	12/18670	0.003962	0.008818	0.003227	APOE/SHANK3
GO:1901163	regulation of trophoblast cell migration	2/149	12/18670	0.003962	0.008818	0.003227	NODAL/ACVR1B
GO:1902337	regulation of apoptotic process involved in morphogenesis	2/149	12/18670	0.003962	0.008818	0.003227	NOTCH1/BAX
GO:1902510	regulation of apoptotic DNA fragmentation	2/149	12/18670	0.003962	0.008818	0.003227	IL6/BAX
GO:1904181	positive regulation of membrane depolarization	2/149	12/18670	0.003962	0.008818	0.003227	PARP1/KDR
GO:1905244	regulation of modification of synaptic structure	2/149	12/18670	0.003962	0.008818	0.003227	FMR1/ABL1
GO:2000121	regulation of removal of superoxide radicals	2/149	12/18670	0.003962	0.008818	0.003227	NFE2L2/TNF
GO:0001941	postsynaptic membrane organization	3/149	40/18670	0.003965	0.008818	0.003227	APOE/SHANK3/NRXN1
GO:0035315	hair cell differentiation	3/149	40/18670	0.003965	0.008818	0.003227	CTNNB1/SOD1/NOTCH1
GO:0042036	negative regulation of cytokine biosynthetic process	3/149	40/18670	0.003965	0.008818	0.003227	IL6/IL10/NFKB1
GO:0045124	regulation of bone resorption	3/149	40/18670	0.003965	0.008818	0.003227	IL6/EGFR/SRC

GO:0050832	defense response to fungus	3/149	40/18670	0.003965	0.008818	0.003227	MPO/TGFB1/S100A8
GO:0071470	cellular response to osmotic stress	3/149	40/18670	0.003965	0.008818	0.003227	PTGS2/CASP3/MYLK
GO:2000008	regulation of protein localization to cell surface	3/149	40/18670	0.003965	0.008818	0.003227	TNF/CTNNB1/AKT1
GO:0010951	negative regulation of endopeptidase activity	7/149	250/18670	0.003983	0.008853	0.003239	PTGS2/SNCA/SERPINH1/SRC/AKT1/AVP/MMP9
GO:0032970	regulation of actin filament-based process	9/149	388/18670	0.004008	0.008906	0.003259	MET/TGFB1/SHANK3/CACNA1C/ICAM1/CAV1/ABL1/PIK3R1/SCN5A
GO:0007045	cell-substrate adherens junction assembly	4/149	81/18670	0.004028	0.008923	0.003265	BCL2/KDR/ABL1/SRC
GO:0014032	neural crest cell development	4/149	81/18670	0.004028	0.008923	0.003265	RET/SOX9/MAPK1/SNAI2
GO:0016126	sterol biosynthetic process	4/149	81/18670	0.004028	0.008923	0.003265	APOE/HMGCR/SOD1/CFTR
GO:0021766	hippocampus development	4/149	81/18670	0.004028	0.008923	0.003265	ALK/NKX2-1/GSK3B/CASP3
GO:0032436	positive regulation of proteasomal ubiquitin-dependent protein catabolic process	4/149	81/18670	0.004028	0.008923	0.003265	NFE2L2/GSK3B/CAV1/AKT1
GO:0048041	focal adhesion assembly	4/149	81/18670	0.004028	0.008923	0.003265	BCL2/KDR/ABL1/SRC
GO:0019362	pyridine nucleotide metabolic process	6/149	189/18670	0.004133	0.009146	0.003347	IGF1/PTGS2/INS/IFNG/TP53/HTR2A
GO:0046496	nicotinamide nucleotide metabolic process	6/149	189/18670	0.004133	0.009146	0.003347	IGF1/PTGS2/INS/IFNG/TP53/HTR2A
GO:0002218	activation of innate immune response	8/149	319/18670	0.004141	0.009159	0.003352	IRF1/CASP8/ESR1/S100A8/CAV1/SRC/NFKB1/NFKBIA
GO:0042552	myelination	5/149	132/18670	0.00416	0.009196	0.003365	CTNNB1/TGFB1/SOD1/ERBB2/AKT1
GO:0051702	interaction with symbiont	4/149	82/18670	0.004209	0.009295	0.003401	APOE/F2/FMR1/JUN

GO:0071158	positive regulation of cell cycle arrest	4/149	82/18670	0.004209	0.009295	0.003401	TGFB1/TP53/CDKN1A/BAX
GO:0006308	DNA catabolic process	3/149	41/18670	0.004253	0.009366	0.003427	IL6/CASP3/BAX
GO:0042572	retinol metabolic process	3/149	41/18670	0.004253	0.009366	0.003427	CYP1A1/ADH1C/CYP3A4
GO:0043267	negative regulation of potassium ion transport	3/149	41/18670	0.004253	0.009366	0.003427	NOS1/CAV1/HTR2A
GO:0097178	ruffle assembly	3/149	41/18670	0.004253	0.009366	0.003427	P2RY12/ICAM1/CAV1
GO:0098815	modulation of excitatory postsynaptic potential	3/149	41/18670	0.004253	0.009366	0.003427	SHANK3/NRXN1/GRIN1
GO:1900117	regulation of execution phase of apoptosis	3/149	41/18670	0.004253	0.009366	0.003427	IL6/TP53/BAX
GO:0046470	phosphatidylcholine metabolic process	4/149	83/18670	0.004395	0.009668	0.003538	PLA2G2A/PON1/PLA2G6/ACHE
GO:0060218	hematopoietic stem cell differentiation	4/149	83/18670	0.004395	0.009668	0.003538	NFE2L2/ABL1/TCF3/TP53
GO:0006165	nucleoside diphosphate phosphorylation	5/149	134/18670	0.004433	0.009738	0.003563	IGF1/INS/IFNG/TP53/HTR2A
GO:0007272	ensheathment of neurons	5/149	134/18670	0.004433	0.009738	0.003563	CTNNB1/TGFB1/SOD1/ERBB2/AKT1
GO:0008366	axon ensheathment	5/149	134/18670	0.004433	0.009738	0.003563	CTNNB1/TGFB1/SOD1/ERBB2/AKT1
GO:0048839	inner ear development	6/149	192/18670	0.004461	0.009795	0.003584	SOX9/TGFB1/KCNQ1/SOD1/SPARC/NOTCH1
GO:0042149	cellular response to glucose starvation	3/149	42/18670	0.004554	0.009993	0.003657	BCL2/NFE2L2/TP53
GO:0050886	endocrine process	4/149	84/18670	0.004587	0.010041	0.003674	LEP/OPRK1/AVPR2/IL1B
GO:0051453	regulation of intracellular pH	4/149	84/18670	0.004587	0.010041	0.003674	BCL2/FASLG/CFTR/AVP
GO:0060420	regulation of heart growth	4/149	84/18670	0.004587	0.010041	0.003674	IGF1/MAPK1/FGF2/NOTCH1
GO:1902803	regulation of synaptic vesicle transport	4/149	84/18670	0.004587	0.010041	0.003674	DRD2/GSK3B/FMR1/HTR2A

GO:1905897	regulation of response to endoplasmic reticulum stress	4/149	84/18670	0.004587	0.010041	0.003674	NFE2L2/CAV1/PIK3R1/BAX
GO:0002679	respiratory burst involved in defense response	2/149	13/18670	0.004658	0.010055	0.003679	MPO/INS
GO:0003084	positive regulation of systemic arterial blood pressure	2/149	13/18670	0.004658	0.010055	0.003679	AVPR2/AVP
GO:0007183	SMAD protein complex assembly	2/149	13/18670	0.004658	0.010055	0.003679	TGFB1/PARP1
GO:0007494	midgut development	2/149	13/18670	0.004658	0.010055	0.003679	RET/EGFR
GO:0010763	positive regulation of fibroblast migration	2/149	13/18670	0.004658	0.010055	0.003679	TGFB1/AKT1
GO:0031953	negative regulation of protein autophosphorylation	2/149	13/18670	0.004658	0.010055	0.003679	CAV1/JUN
GO:0032488	Cdc42 protein signal transduction	2/149	13/18670	0.004658	0.010055	0.003679	APOE/ABL1
GO:0033127	regulation of histone phosphorylation	2/149	13/18670	0.004658	0.010055	0.003679	FMR1/IL1B
GO:0033623	regulation of integrin activation	2/149	13/18670	0.004658	0.010055	0.003679	P2RY12/SRC
GO:0035641	locomotory exploration behavior	2/149	13/18670	0.004658	0.010055	0.003679	DPP4/APOE
GO:0042711	maternal behavior	2/149	13/18670	0.004658	0.010055	0.003679	OPRK1/AVP
GO:0043568	positive regulation of insulin-like growth factor receptor signaling pathway	2/149	13/18670	0.004658	0.010055	0.003679	IGF1/IGFBP3
GO:0045086	positive regulation of interleukin-2 biosynthetic process	2/149	13/18670	0.004658	0.010055	0.003679	IL1A/IL1B
GO:0047484	regulation of response to osmotic stress	2/149	13/18670	0.004658	0.010055	0.003679	ABCB1/PTGS2

GO:0048505	regulation of timing of cell differentiation	2/149	13/18670	0.004658	0.010055	0.003679	NODAL/NOTCH1
GO:0048715	negative regulation of oligodendrocyte differentiation	2/149	13/18670	0.004658	0.010055	0.003679	CTNNB1/NOTCH1
GO:0050966	detection of mechanical stimulus involved in sensory perception of pain	2/149	13/18670	0.004658	0.010055	0.003679	TRPA1/HTR2A
GO:0060746	parental behavior	2/149	13/18670	0.004658	0.010055	0.003679	OPRK1/AVP
GO:0061029	eyelid development in camera-type eye	2/149	13/18670	0.004658	0.010055	0.003679	EGFR/JUN
GO:0061430	bone trabecula morphogenesis	2/149	13/18670	0.004658	0.010055	0.003679	MMP2/COL1A1
GO:0071352	cellular response to interleukin-2	2/149	13/18670	0.004658	0.010055	0.003679	IL2/JAK3
GO:0071872	cellular response to epinephrine stimulus	2/149	13/18670	0.004658	0.010055	0.003679	SNCA/KCNQ1
GO:0072182	regulation of nephron tubule epithelial cell differentiation	2/149	13/18670	0.004658	0.010055	0.003679	CTNNB1/STAT1
GO:0090399	replicative senescence	2/149	13/18670	0.004658	0.010055	0.003679	TP53/CDKN1A
GO:1900103	positive regulation of endoplasmic reticulum unfolded protein response	2/149	13/18670	0.004658	0.010055	0.003679	PIK3R1/BAX
GO:1900272	negative regulation of long-term synaptic potentiation	2/149	13/18670	0.004658	0.010055	0.003679	APOE/ABL1
GO:1901550	regulation of endothelial cell development	2/149	13/18670	0.004658	0.010055	0.003679	TNF/IL1B
GO:1902430	negative regulation of amyloid-beta formation	2/149	13/18670	0.004658	0.010055	0.003679	IGF1/APOE

GO:1903140	regulation of establishment of endothelial barrier	2/149	13/18670	0.004658	0.010055	0.003679	TNF/IL1B
GO:0046939	nucleotide phosphorylation	5/149	136/18670	0.004719	0.010182	0.003726	IGF1/INS/IFNG/TP53/HTR2A
GO:0034308	primary alcohol metabolic process	4/149	85/18670	0.004784	0.010306	0.003771	IGF1/CYP1A1/ADH1C/CYP3A4
GO:0048864	stem cell development	4/149	85/18670	0.004784	0.010306	0.003771	RET/SOX9/MAPK1/SNAI2
GO:1901532	regulation of hematopoietic progenitor cell differentiation	4/149	85/18670	0.004784	0.010306	0.003771	NFE2L2/ABL1/TCF3/NOTCH1
GO:0016569	covalent chromatin modification	10/149	474/18670	0.004796	0.010327	0.003779	NOS1/PRDM16/CTNNB1/SNCA/TGFB1/FMR1/IGF2/TP53/IL1B/SNAI2
GO:0072524	pyridine-containing compound metabolic process	6/149	195/18670	0.004808	0.010348	0.003787	IGF1/PTGS2/INS/IFNG/TP53/HTR2A
GO:0031279	regulation of cyclase activity	3/149	43/18670	0.004867	0.01044	0.00382	NOS2/NOS1/CACNA1C
GO:0033173	calcineurin-NFAT signaling cascade	3/149	43/18670	0.004867	0.01044	0.00382	IGF1/TNF/GSK3B
GO:0034198	cellular response to amino acid starvation	3/149	43/18670	0.004867	0.01044	0.00382	FAS/MAPK1/CDKN1A
GO:0045687	positive regulation of glial cell differentiation	3/149	43/18670	0.004867	0.01044	0.00382	PPARG/TGFB1/NOTCH1
GO:0045746	negative regulation of Notch signaling pathway	3/149	43/18670	0.004867	0.01044	0.00382	EGFR/AKT1/NFKBIA
GO:0086004	regulation of cardiac muscle cell contraction	3/149	43/18670	0.004867	0.01044	0.00382	CACNA1C/CAV1/SCN5A
GO:1990573	potassium ion import across plasma membrane	3/149	43/18670	0.004867	0.01044	0.00382	SLC12A2/ABCC9/SLC12A1
GO:0072659	protein localization to plasma membrane	7/149	260/18670	0.004925	0.01056	0.003864	TNF/TGFB1/INS/EGFR/IFNG/AKT1/PIK3R1
GO:0009135	purine nucleoside diphosphate metabolic process	5/149	138/18670	0.005019	0.01075	0.003934	IGF1/INS/IFNG/TP53/HTR2A

GO:0009179	purine ribonucleoside diphosphate metabolic process	5/149	138/18670	0.005019	0.01075	0.003934	IGF1/INS/IFNG/TP53/HTR2A
GO:0009108	coenzyme biosynthetic process	7/149	261/18670	0.005028	0.010764	0.003939	IGF1/PTGS2/SNCA/INS/IFNG/TP53/HTR2A
GO:0002221	pattern recognition receptor signaling pathway	6/149	197/18670	0.00505	0.010807	0.003954	IRF1/CASP8/ESR1/S100A8/CAV1/NFKBIA
GO:0007281	germ cell development	7/149	262/18670	0.005132	0.010972	0.004015	IGF1/BCL2/CTNNB1/CFTR/SRC/AKT1/BAX
GO:0010466	negative regulation of peptidase activity	7/149	262/18670	0.005132	0.010972	0.004015	PTGS2/SNCA/SERPINH1/SRC/AKT1/AVP/MMP9
GO:0031333	negative regulation of protein complex assembly	5/149	139/18670	0.005173	0.011049	0.004043	SNCA/GSK3B/INS/SRC/THRA
GO:0035304	regulation of protein dephosphorylation	5/149	139/18670	0.005173	0.011049	0.004043	TNF/DRD2/TGFB1/GSK3B/IGFBP3
GO:0003197	endocardial cushion development	3/149	44/18670	0.005193	0.011059	0.004047	SOX9/SNAI2/NOTCH1
GO:0014047	glutamate secretion	3/149	44/18670	0.005193	0.011059	0.004047	SNCA/TRPV1/AVP
GO:0034105	positive regulation of tissue remodeling	3/149	44/18670	0.005193	0.011059	0.004047	EGFR/IL2/BAX
GO:0044060	regulation of endocrine process	3/149	44/18670	0.005193	0.011059	0.004047	LEP/OPRK1/IL1B
GO:0061756	leukocyte adhesion to vascular endothelial cell	3/149	44/18670	0.005193	0.011059	0.004047	LEP/TNF/ICAM1
GO:0070897	transcription preinitiation complex assembly	3/149	44/18670	0.005193	0.011059	0.004047	ESR1/THRA/TP53
GO:0048477	oogenesis	4/149	87/18670	0.005195	0.011059	0.004047	IGF1/BCL2/CTNNB1/SRC
GO:0009185	ribonucleoside diphosphate metabolic process	5/149	140/18670	0.005331	0.011338	0.004149	IGF1/INS/IFNG/TP53/HTR2A
GO:0034620	cellular response to unfolded protein	5/149	140/18670	0.005331	0.011338	0.004149	CXCL8/NFE2L2/CCND1/PIK3R1/BAX

GO:0140014	mitotic nuclear division	7/149	264/18670	0.005346	0.011363	0.004158	IGF1/IL1A/TGFB1/INS/IGF2/RB1/IL1B
GO:0003188	heart valve formation	2/149	14/18670	0.005406	0.0114	0.004172	SOX9/NOTCH1
GO:0021781	glial cell fate commitment	2/149	14/18670	0.005406	0.0114	0.004172	SOX9/CTNNB1
GO:0035791	platelet-derived growth factor receptor-beta signaling pathway	2/149	14/18670	0.005406	0.0114	0.004172	ABL1/SRC
GO:0038166	angiotensin-activated signaling pathway	2/149	14/18670	0.005406	0.0114	0.004172	CAV1/SRC
GO:0040034	regulation of development, heterochronic	2/149	14/18670	0.005406	0.0114	0.004172	NODAL/NOTCH1
GO:0043374	CD8-positive, alpha-beta T cell differentiation	2/149	14/18670	0.005406	0.0114	0.004172	IRF1/BCL2
GO:0045346	regulation of MHC class II biosynthetic process	2/149	14/18670	0.005406	0.0114	0.004172	IL10/IFNG
GO:0051712	positive regulation of killing of cells of other organism	2/149	14/18670	0.005406	0.0114	0.004172	NOS2/IFNG
GO:0051917	regulation of fibrinolysis	2/149	14/18670	0.005406	0.0114	0.004172	F2/F12
GO:0060009	Sertoli cell development	2/149	14/18670	0.005406	0.0114	0.004172	SOX9/ICAM1
GO:0070669	response to interleukin-2	2/149	14/18670	0.005406	0.0114	0.004172	IL2/JAK3
GO:0072216	positive regulation of metanephros development	2/149	14/18670	0.005406	0.0114	0.004172	RET/MYC
GO:0086069	bundle of His cell to Purkinje myocyte communication	2/149	14/18670	0.005406	0.0114	0.004172	SCN10A/SCN5A
GO:0097154	GABAergic neuron differentiation	2/149	14/18670	0.005406	0.0114	0.004172	DRD2/NKX2-1

GO:1903351	cellular response to dopamine	2/149	14/18670	0.005406	0.0114	0.004172	MAPK1/ABL1
GO:1903624	regulation of DNA catabolic process	2/149	14/18670	0.005406	0.0114	0.004172	IL6/BAX
GO:1904748	regulation of apoptotic process involved in development	2/149	14/18670	0.005406	0.0114	0.004172	NOTCH1/BAX
GO:1903321	negative regulation of protein modification by small protein conjugation or removal	4/149	88/18670	0.005409	0.011401	0.004172	CTNNB1/CAV1/ABL1/AKT1
GO:0014014	negative regulation of gliogenesis	3/149	45/18670	0.005532	0.011636	0.004258	F2/CTNNB1/NOTCH1
GO:0042771	intrinsic apoptotic signaling pathway in response to DNA damage by p53 class mediator	3/149	45/18670	0.005532	0.011636	0.004258	BCL2/TP53/CDKN1A
GO:0051932	synaptic transmission, GABAergic	3/149	45/18670	0.005532	0.011636	0.004258	DRD2/GABRG3/GABRA3
GO:0071827	plasma lipoprotein particle organization	3/149	45/18670	0.005532	0.011636	0.004258	PLA2G2A/APOE/MPO
GO:0009205	purine ribonucleoside triphosphate metabolic process	8/149	335/18670	0.005534	0.011636	0.004258	IGF1/SNCA/TGFB1/PARP1/INS/IFNG/TP53/HTR2A
GO:0097306	cellular response to alcohol	4/149	89/18670	0.005629	0.011831	0.004329	PPARG/CTNNB1/CFTR/AKT1
GO:0034332	adherens junction organization	5/149	142/18670	0.005657	0.011884	0.004349	BCL2/CTNNB1/KDR/ABL1/SRC
GO:0014033	neural crest cell differentiation	4/149	90/18670	0.005855	0.012283	0.004495	RET/SOX9/MAPK1/SNAI2
GO:0042475	odontogenesis of dentin-containing tooth	4/149	90/18670	0.005855	0.012283	0.004495	CTNNB1/SCN10A/BAX/SCN5A
GO:0045778	positive regulation of ossification	4/149	90/18670	0.005855	0.012283	0.004495	IGF1/CTNNB1/TGFB1/IL6
GO:0002067	glandular epithelial cell differentiation	3/149	46/18670	0.005884	0.012303	0.004502	IL13/GSK3B/NOTCH1

GO:0043330	response to exogenous dsRNA	3/149	46/18670	0.005884	0.012303	0.004502	MAPK1/CAV1/NFKBIA
GO:0048599	oocyte development	3/149	46/18670	0.005884	0.012303	0.004502	IGF1/BCL2/CTNNB1
GO:0051339	regulation of lyase activity	3/149	46/18670	0.005884	0.012303	0.004502	NOS2/NOS1/CACNA1C
GO:0097720	calcineurin-mediated signaling	3/149	46/18670	0.005884	0.012303	0.004502	IGF1/TNF/GSK3B
GO:1900015	regulation of cytokine production involved in inflammatory response	3/149	46/18670	0.005884	0.012303	0.004502	NOS2/LEP/F2
GO:1990928	response to amino acid starvation	3/149	46/18670	0.005884	0.012303	0.004502	FAS/MAPK1/CDKN1A
GO:1902905	positive regulation of supramolecular fiber organization	6/149	204/18670	0.005968	0.012474	0.004564	MET/APOE/MAPT/ICAM1/ABL1/RB1
GO:0060041	retina development in camera-type eye	5/149	144/18670	0.005996	0.012527	0.004584	THRB/RET/SOX9/ACHE/BAX
GO:0009167	purine ribonucleoside monophosphate metabolic process	8/149	340/18670	0.006035	0.012602	0.004611	IGF1/SNCA/TGFB1/PARP1/INS/IFNG/TP53/HTR2A
GO:0045638	negative regulation of myeloid cell differentiation	4/149	91/18670	0.006087	0.012698	0.004647	CTNNB1/MYC/PIK3R1/NFKBIA
GO:0048814	regulation of dendrite morphogenesis	4/149	91/18670	0.006087	0.012698	0.004647	SHANK3/GSK3B/FMR1/GRIN1
GO:0009126	purine nucleoside monophosphate metabolic process	8/149	341/18670	0.006139	0.012795	0.004682	IGF1/SNCA/TGFB1/PARP1/INS/IFNG/TP53/HTR2A
GO:0009199	ribonucleoside triphosphate metabolic process	8/149	341/18670	0.006139	0.012795	0.004682	IGF1/SNCA/TGFB1/PARP1/INS/IFNG/TP53/HTR2A
GO:0006865	amino acid transport	5/149	145/18670	0.006172	0.012826	0.004693	LEP/SNCA/TRPV1/SLC6A8/AVP

GO:0032105	negative regulation of response to extracellular stimulus	2/149	15/18670	0.006205	0.012826	0.004693	LEP/SNAI2
GO:0032108	negative regulation of response to nutrient levels	2/149	15/18670	0.006205	0.012826	0.004693	LEP/SNAI2
GO:0032274	gonadotropin secretion	2/149	15/18670	0.006205	0.012826	0.004693	LEP/OPRK1
GO:0034616	response to laminar fluid shear stress	2/149	15/18670	0.006205	0.012826	0.004693	NFE2L2/TGFB1
GO:0042159	lipoprotein catabolic process	2/149	15/18670	0.006205	0.012826	0.004693	APOE/CTSD
GO:0042762	regulation of sulfur metabolic process	2/149	15/18670	0.006205	0.012826	0.004693	NFE2L2/SNCA
GO:0045342	MHC class II biosynthetic process	2/149	15/18670	0.006205	0.012826	0.004693	IL10/IFNG
GO:0045410	positive regulation of interleukin-6 biosynthetic process	2/149	15/18670	0.006205	0.012826	0.004693	TNF/IL1B
GO:0048070	regulation of developmental pigmentation	2/149	15/18670	0.006205	0.012826	0.004693	BCL2/BAX
GO:0050961	detection of temperature stimulus involved in sensory perception	2/149	15/18670	0.006205	0.012826	0.004693	TRPV1/HTR2A
GO:0050965	detection of temperature stimulus involved in sensory perception of pain	2/149	15/18670	0.006205	0.012826	0.004693	TRPV1/HTR2A
GO:0055089	fatty acid homeostasis	2/149	15/18670	0.006205	0.012826	0.004693	APOE/INS
GO:0071801	regulation of podosome assembly	2/149	15/18670	0.006205	0.012826	0.004693	TNF/SRC
GO:0072075	metanephric mesenchyme development	2/149	15/18670	0.006205	0.012826	0.004693	MYC/STAT1

GO:0072160	nephron tubule epithelial cell differentiation	2/149	15/18670	0.006205	0.012826	0.004693	CTNNB1/STAT1
GO:0072283	metanephric renal vesicle morphogenesis	2/149	15/18670	0.006205	0.012826	0.004693	CTNNB1/STAT1
GO:1903350	response to dopamine	2/149	15/18670	0.006205	0.012826	0.004693	MAPK1/ABL1
GO:0009144	purine nucleoside triphosphate metabolic process	8/149	342/18670	0.006245	0.012826	0.004693	IGF1/SNCA/TGFB1/PARP1/INS/IFNG/TP53/HTR2A
GO:0002204	somatic recombination of immunoglobulin genes involved in immune response	3/149	47/18670	0.006248	0.012826	0.004693	TGFB1/IL10/IL2
GO:0002208	somatic diversification of immunoglobulins involved in immune response	3/149	47/18670	0.006248	0.012826	0.004693	TGFB1/IL10/IL2
GO:0014911	positive regulation of smooth muscle cell migration	3/149	47/18670	0.006248	0.012826	0.004693	IGF1/BCL2/SRC
GO:0018198	peptidyl-cysteine modification	3/149	47/18670	0.006248	0.012826	0.004693	NOS2/NOS1/S100A8
GO:0035722	interleukin-12-mediated signaling pathway	3/149	47/18670	0.006248	0.012826	0.004693	IL10/SOD1/IFNG
GO:0042551	neuron maturation	3/149	47/18670	0.006248	0.012826	0.004693	BCL2/RET/RB1
GO:0043370	regulation of CD4-positive, alpha-beta T cell differentiation	3/149	47/18670	0.006248	0.012826	0.004693	IL2/IFNG/JAK3
GO:0045190	isotype switching	3/149	47/18670	0.006248	0.012826	0.004693	TGFB1/IL10/IL2
GO:0048806	genitalia development	3/149	47/18670	0.006248	0.012826	0.004693	CTNNB1/ESR1/BAX
GO:0060351	cartilage development involved in endochondral bone morphogenesis	3/149	47/18670	0.006248	0.012826	0.004693	COL1A1/SOX9/SERPINH1

GO:0060986	endocrine hormone secretion	3/149	47/18670	0.006248	0.012826	0.004693	LEP/OPRK1/IL1B
GO:0061383	trabecula morphogenesis	3/149	47/18670	0.006248	0.012826	0.004693	MMP2/COL1A1/NOTCH1
GO:1900744	regulation of p38MAPK cascade	3/149	47/18670	0.006248	0.012826	0.004693	LEP/OPRK1/IL1B
GO:1903115	regulation of actin filament-based movement	3/149	47/18670	0.006248	0.012826	0.004693	CACNA1C/CAV1/SCN5A
GO:0035710	CD4-positive, alpha-beta T cell activation	4/149	92/18670	0.006325	0.012977	0.004749	IL6/IL2/IFNG/JAK3
GO:0090263	positive regulation of canonical Wnt signaling pathway	5/149	147/18670	0.006533	0.013397	0.004902	COL1A1/EGFR/CAV1/SRC/NFKB1
GO:0003073	regulation of systemic arterial blood pressure	4/149	93/18670	0.006569	0.013447	0.004921	DRD2/AVPR2/TRPV1/AVP
GO:0006476	protein deacetylation	4/149	93/18670	0.006569	0.013447	0.004921	MAPT/TGFB1/IFNG/TP53
GO:0007589	body fluid secretion	4/149	93/18670	0.006569	0.013447	0.004921	OPRK1/CCND1/CAV1/CHRM3
GO:0046634	regulation of alpha-beta T cell activation	4/149	93/18670	0.006569	0.013447	0.004921	IRF1/IL2/IFNG/JAK3
GO:0002762	negative regulation of myeloid leukocyte differentiation	3/149	48/18670	0.006626	0.013528	0.00495	CTNNB1/MYC/PIK3R1
GO:0008542	visual learning	3/149	48/18670	0.006626	0.013528	0.00495	DRD2/HMGCR/GRIN1
GO:0010107	potassium ion import	3/149	48/18670	0.006626	0.013528	0.00495	SLC12A2/ABCC9/SLC12A1
GO:0010862	positive regulation of pathway-restricted SMAD protein phosphorylation	3/149	48/18670	0.006626	0.013528	0.00495	TGFB1/NODAL/ACVR1B
GO:0043268	positive regulation of potassium ion transport	3/149	48/18670	0.006626	0.013528	0.00495	ADRA2A/OPRK1/KCNQ1
GO:0045933	positive regulation of muscle contraction	3/149	48/18670	0.006626	0.013528	0.00495	PTGS2/KCNQ1/CHRM3

GO:00351	appendage			0.00671	0.01369	0.00501	
07	morphogenesis	5/149	148/18670	8	7	2	SOX9/CTNNB1/CACNA1C/NOTCH1/BAX
GO:00351				0.00671	0.01369	0.00501	
08	limb morphogenesis	5/149	148/18670	8	7	2	SOX9/CTNNB1/CACNA1C/NOTCH1/BAX
GO:00516	localization within			0.00671	0.01369	0.00501	
68	membrane	5/149	148/18670	8	7	2	APOE/TGFB1/SHANK3/NRXN1/DRD4
GO:00421				0.00672	0.01369	0.00501	
19	neutrophil activation	10/149	498/18670	2	7	2	PLAU/CXCL8/MPO/CTSD/S100A8/HSP90AA1/MAPK1/CAT/NFKB1/MMP9
GO:00512	protein			0.00681	0.01388	0.00508	
89	homotetramerization	4/149	94/18670	9	9	2	TRPA1/TRPV1/TP53/CAT
GO:00301	negative regulation of			0.00684	0.01394	0.00510	
78	Wnt signaling pathway	6/149	210/18670	7	1	1	APOE/SOX9/GSK3B/CAV1/SNAI2/NOTCH1
GO:19039	regulation of viral life			0.00690	0.01405	0.00514	
00	cycle	5/149	149/18670	8	8	4	BCL2/CXCL8/TNF/FMR1/NOTCH1
	cytokine production						
GO:00025	involved in inflammatory			0.00701	0.01414	0.00517	
34	response	3/149	49/18670	8	5	6	NOS2/LEP/F2
GO:00713	cellular response to			0.00701	0.01414	0.00517	
49	interleukin-12	3/149	49/18670	8	5	6	IL10/SOD1/IFNG
GO:00718	protein-lipid complex			0.00701	0.01414	0.00517	
25	subunit organization	3/149	49/18670	8	5	6	PLA2G2A/APOE/MPO
GO:19900	cellular response to nerve			0.00701	0.01414	0.00517	
90	growth factor stimulus	3/149	49/18670	8	5	6	MAPT/TRPV1/AKT1
GO:00020				0.00705	0.01414	0.00517	
70	epithelial cell maturation	2/149	16/18670	5	5	6	PGR/CDKN1A
	regulation of cytokine						
GO:00027	secretion involved in			0.00705	0.01414	0.00517	
39	immune response	2/149	16/18670	5	5	6	TNF/IL10
GO:00071				0.00705	0.01414	0.00517	
58	neuron cell-cell adhesion	2/149	16/18670	5	5	6	RET/NRXN1
GO:00075				0.00705	0.01414	0.00517	
67	parturition	2/149	16/18670	5	5	6	CYP1A1/NODAL
GO:00140	regulation of glutamate			0.00705	0.01414	0.00517	
48	secretion	2/149	16/18670	5	5	6	SNCA/AVP
GO:00140	regulation of			0.00705	0.01414	0.00517	
61	norepinephrine secretion	2/149	16/18670	5	5	6	ADRA2A/P2RY12

GO:0016540	protein autoprocessing	2/149	16/18670	0.007055	0.014145	0.005176	PARP1/F12
GO:0019372	lipoxygenase pathway	2/149	16/18670	0.007055	0.014145	0.005176	PON1/PTGS2
GO:0030214	hyaluronan catabolic process	2/149	16/18670	0.007055	0.014145	0.005176	TGFB1/FGF2
GO:0030889	negative regulation of B cell proliferation	2/149	16/18670	0.007055	0.014145	0.005176	CASP3/IL10
GO:0032305	positive regulation of icosanoid secretion	2/149	16/18670	0.007055	0.014145	0.005176	PLA2G6/IL1B
GO:0043217	myelin maintenance	2/149	16/18670	0.007055	0.014145	0.005176	SOD1/AKT1
GO:0045623	negative regulation of T-helper cell differentiation	2/149	16/18670	0.007055	0.014145	0.005176	IL2/JAK3
GO:0045986	negative regulation of smooth muscle contraction	2/149	16/18670	0.007055	0.014145	0.005176	PTGS2/SOD1
GO:0046325	negative regulation of glucose import	2/149	16/18670	0.007055	0.014145	0.005176	LEP/TNF
GO:0061684	chaperone-mediated autophagy	2/149	16/18670	0.007055	0.014145	0.005176	SNCA/HSP90AA1
GO:0070886	positive regulation of calcineurin-NFAT signaling cascade	2/149	16/18670	0.007055	0.014145	0.005176	IGF1/TNF
GO:0071380	cellular response to prostaglandin E stimulus	2/149	16/18670	0.007055	0.014145	0.005176	PPARG/AKT1
GO:0071871	response to epinephrine	2/149	16/18670	0.007055	0.014145	0.005176	SNCA/KCNQ1
GO:0072189	ureter development	2/149	16/18670	0.007055	0.014145	0.005176	RET/SOX9
GO:0090153	regulation of sphingolipid biosynthetic process	2/149	16/18670	0.007055	0.014145	0.005176	PLA2G6/TNF
GO:0099170	postsynaptic modulation of chemical synaptic transmission	2/149	16/18670	0.007055	0.014145	0.005176	DRD2/FMR1

GO:0106058	positive regulation of calcineurin-mediated signaling	2/149	16/18670	0.007055	0.014145	0.005176	IGF1/TNF
GO:1900451	positive regulation of glutamate receptor signaling pathway	2/149	16/18670	0.007055	0.014145	0.005176	SHANK3/IFNG
GO:1902992	negative regulation of amyloid precursor protein catabolic process	2/149	16/18670	0.007055	0.014145	0.005176	IGF1/APOE
GO:1904294	positive regulation of ERAD pathway	2/149	16/18670	0.007055	0.014145	0.005176	NFE2L2/CAV1
GO:1905038	regulation of membrane lipid metabolic process	2/149	16/18670	0.007055	0.014145	0.005176	PLA2G6/TNF
GO:1990000	amyloid fibril formation	2/149	16/18670	0.007055	0.014145	0.005176	APOE/MAPT
GO:2000303	regulation of ceramide biosynthetic process	2/149	16/18670	0.007055	0.014145	0.005176	PLA2G6/TNF
GO:2000060	positive regulation of ubiquitin-dependent protein catabolic process	4/149	95/18670	0.007076	0.01418	0.005189	NFE2L2/GSK3B/CAV1/AKT1
GO:0030516	regulation of axon extension	4/149	96/18670	0.007338	0.0147	0.005379	APOE/MAPT/GSK3B/ABL1
GO:0010171	body morphogenesis	3/149	50/18670	0.007423	0.014829	0.005426	MMP2/COL1A1/TGFB1
GO:0042398	cellular modified amino acid biosynthetic process	3/149	50/18670	0.007423	0.014829	0.005426	NFE2L2/GGT1/GATM
GO:0045540	regulation of cholesterol biosynthetic process	3/149	50/18670	0.007423	0.014829	0.005426	APOE/HMGCR/SOD1
GO:0070671	response to interleukin-12	3/149	50/18670	0.007423	0.014829	0.005426	IL10/SOD1/IFNG
GO:0086009	membrane repolarization	3/149	50/18670	0.007423	0.014829	0.005426	KCNQ1/CAV1/SCN5A
GO:0106118	regulation of sterol biosynthetic process	3/149	50/18670	0.007423	0.014829	0.005426	APOE/HMGCR/SOD1

GO:1903050	regulation of proteolysis involved in cellular protein catabolic process	6/149	214/18670	0.007483	0.014943	0.005468	APOE/NFE2L2/GSK3B/FMR1/CAV1/AKT1
GO:0007044	cell-substrate junction assembly	4/149	97/18670	0.007607	0.01515	0.005544	BCL2/KDR/ABL1/SRC
GO:0014020	primary neural tube formation	4/149	97/18670	0.007607	0.01515	0.005544	TGFB1/CASP3/NODAL/ABL1
GO:0032231	regulation of actin filament bundle assembly	4/149	97/18670	0.007607	0.01515	0.005544	MET/SHANK3/ABL1/PIK3R1
GO:0044773	mitotic DNA damage checkpoint	4/149	97/18670	0.007607	0.01515	0.005544	CCND1/TP53/CDKN1A/BAX
GO:0060996	dendritic spine development	4/149	97/18670	0.007607	0.01515	0.005544	APOE/SHANK3/FMR1/IL2
GO:2001022	positive regulation of response to DNA damage stimulus	4/149	97/18670	0.007607	0.01515	0.005544	MYC/PARP1/FMR1/EGFR
GO:0009161	ribonucleoside monophosphate metabolic process	8/149	354/18670	0.007623	0.015175	0.005553	IGF1/SNCA/TGFB1/PARP1/INS/IFNG/TP53/HTR2A
GO:0051017	actin filament bundle assembly	5/149	153/18670	0.007704	0.015329	0.005609	MET/SHANK3/ABL1/SRC/PIK3R1
GO:0000075	cell cycle checkpoint	6/149	216/18670	0.007817	0.015547	0.005689	CCND1/TGFB1/RB1/TP53/CDKN1A/BAX
GO:0001954	positive regulation of cell-matrix adhesion	3/149	51/18670	0.007841	0.015568	0.005697	GSK3B/KDR/ABL1
GO:0009994	oocyte differentiation	3/149	51/18670	0.007841	0.015568	0.005697	IGF1/BCL2/CTNNB1
GO:0045599	negative regulation of fat cell differentiation	3/149	51/18670	0.007841	0.015568	0.005697	TNF/TGFB1/IL6
GO:0051452	intracellular pH reduction	3/149	51/18670	0.007841	0.015568	0.005697	BCL2/FASLG/AVP
GO:0030038	contractile actin filament bundle assembly	4/149	98/18670	0.007883	0.015605	0.00571	MET/ABL1/SRC/PIK3R1
GO:0043149	stress fiber assembly	4/149	98/18670	0.007883	0.015605	0.00571	MET/ABL1/SRC/PIK3R1

GO:0006090	pyruvate metabolic process	5/149	154/18670	0.007913	0.015605	0.00571	IGF1/INS/IFNG/TP53/HTR2A
GO:0003198	epithelial to mesenchymal transition involved in endocardial cushion formation	2/149	17/18670	0.007954	0.015605	0.00571	SNAI2/NOTCH1
GO:0006750	glutathione biosynthetic process	2/149	17/18670	0.007954	0.015605	0.00571	NFE2L2/GGT1
GO:0006978	DNA damage response, signal transduction by p53 class mediator resulting in transcription of p21 class mediator	2/149	17/18670	0.007954	0.015605	0.00571	TP53/CDKN1A
GO:0007635	chemosensory behavior	2/149	17/18670	0.007954	0.015605	0.00571	TRPV1/GRIN1
GO:0008340	determination of adult lifespan	2/149	17/18670	0.007954	0.015605	0.00571	LEP/TP53
GO:0010224	response to UV-B	2/149	17/18670	0.007954	0.015605	0.00571	BCL2/CDKN1A
GO:0010744	positive regulation of macrophage derived foam cell differentiation	2/149	17/18670	0.007954	0.015605	0.00571	PLA2G2A/NFKB1
GO:0030540	female genitalia development	2/149	17/18670	0.007954	0.015605	0.00571	ESR1/BAX
GO:0031000	response to caffeine	2/149	17/18670	0.007954	0.015605	0.00571	PPARG/CACNA1S
GO:0032460	negative regulation of protein oligomerization	2/149	17/18670	0.007954	0.015605	0.00571	INS/SRC
GO:0032966	negative regulation of collagen biosynthetic process	2/149	17/18670	0.007954	0.015605	0.00571	PPARG/IL6
GO:0033599	regulation of mammary gland epithelial cell proliferation	2/149	17/18670	0.007954	0.015605	0.00571	CCND1/BAX
GO:0036270	response to diuretic	2/149	17/18670	0.007954	0.015605	0.00571	PPARG/CACNA1S

GO:0038092	nodal signaling pathway	2/149	17/18670	0.007954	0.015605	0.00571	NODAL/ACVR1B
GO:0042994	cytoplasmic sequestering of transcription factor	2/149	17/18670	0.007954	0.015605	0.00571	IL10/NFKBIA
GO:0048243	norepinephrine secretion	2/149	17/18670	0.007954	0.015605	0.00571	ADRA2A/P2RY12
GO:0048521	negative regulation of behavior	2/149	17/18670	0.007954	0.015605	0.00571	DRD2/INS
GO:0051709	regulation of killing of cells of other organism	2/149	17/18670	0.007954	0.015605	0.00571	NOS2/IFNG
GO:0051969	regulation of transmission of nerve impulse	2/149	17/18670	0.007954	0.015605	0.00571	FMR1/AVP
GO:0061577	calcium ion transmembrane transport via high voltage-gated calcium channel	2/149	17/18670	0.007954	0.015605	0.00571	NOS1/CACNA1C
GO:0071850	mitotic cell cycle arrest	2/149	17/18670	0.007954	0.015605	0.00571	TP53/CDKN1A
GO:0097107	postsynaptic density assembly	2/149	17/18670	0.007954	0.015605	0.00571	SHANK3/NRXN1
GO:1904996	positive regulation of leukocyte adhesion to vascular endothelial cell	2/149	17/18670	0.007954	0.015605	0.00571	TNF/ICAM1
GO:2000641	regulation of early endosome to late endosome transport	2/149	17/18670	0.007954	0.015605	0.00571	MAPK1/SRC
GO:0046474	glycerophospholipid biosynthetic process	6/149	217/18670	0.007988	0.015664	0.005732	PLA2G2A/PLA2G6/ACHE/FGF2/PIK3R1/HTR2A
GO:0022412	cellular process involved in reproduction in multicellular organism	8/149	357/18670	0.008001	0.015684	0.005739	IGF1/BCL2/CTNNB1/TGFB1/CFTR/SRC/AKT1/BAX
GO:0032271	regulation of protein polymerization	6/149	218/18670	0.008161	0.01599	0.005851	MET/MAPT/SNCA/HSP90AA1/ICAM1/ABL1
GO:0009620	response to fungus	3/149	52/18670	0.008273	0.016175	0.005919	MPO/TGFB1/S100A8

GO:00315				0.00827	0.01617	0.00591	
29	ruffle organization	3/149	52/18670	3	5	9	P2RY12/ICAM1/CAV1
GO:00323	regulation of chondrocyte			0.00827	0.01617	0.00591	
30	differentiation	3/149	52/18670	3	5	9	SOX9/CTNNB1/SNAI2
GO:19900	response to nerve growth			0.00827	0.01617	0.00591	
89	factor	3/149	52/18670	3	5	9	MAPT/TRPV1/AKT1
	positive regulation of						
GO:20001	neural precursor cell			0.00827	0.01617	0.00591	
79	proliferation	3/149	52/18670	3	5	9	DRD2/CTNNB1/NOTCH1
GO:00165				0.00834	0.01629	0.00596	
73	histone acetylation	5/149	156/18670	1	4	2	NOS1/SNCA/TGFB1/IL1B/SNAI2
GO:00452	cell-cell junction			0.00834	0.01629	0.00596	
16	organization	5/149	156/18670	1	4	2	TNF/TGFB1/GPBAR1/CAV1/SNAI2
GO:00608				0.00845	0.01650		
40	artery development	4/149	100/18670	3	5	0.00604	LEP/APOE/MYLK/NOTCH1
GO:00718	protein-DNA complex			0.00845	0.01650	0.00604	
24	subunit organization	7/149	288/18670	9	9	1	SOX9/ESR1/MYC/PARP1/RB1/THRA/TP53
GO:00615	actin filament bundle			0.00856	0.01670	0.00611	
72	organization	5/149	157/18670	2	2	2	MET/SHANK3/ABL1/SRC/PIK3R1
GO:00091	nucleoside triphosphate			0.00866		0.00618	
41	metabolic process	8/149	362/18670	2	0.01689	1	IGF1/SNCA/TGFB1/PARP1/INS/IFNG/TP53/HTR2A
GO:00076				0.00871	0.01697		
32	visual behavior	3/149	53/18670	9	2	0.00621	DRD2/HMGCR/GRIN1
GO:00218	forebrain neuron			0.00871	0.01697		
79	differentiation	3/149	53/18670	9	2	0.00621	DRD2/SHANK3/NKX2-1
GO:00380				0.00871	0.01697		
66	p38MAPK cascade	3/149	53/18670	9	2	0.00621	LEP/OPRK1/IL1B
GO:00458				0.00871	0.01697		
51	pH reduction	3/149	53/18670	9	2	0.00621	BCL2/FASLG/AVP
GO:00000	regulation of DNA			0.00874		0.00622	
18	recombination	4/149	101/18670	9	0.017	1	TGFB1/PARP1/IL10/IL2
GO:00326	interleukin-1 beta			0.00874		0.00622	
11	production	4/149	101/18670	9	0.017	1	IGF1/CASP8/IFNG/IL1B
GO:00508	defense response to			0.00874		0.00622	
30	Gram-positive bacterium	4/149	101/18670	9	0.017	1	PLA2G2A/CRP/TNF/IL6

GO:0097006	regulation of plasma lipoprotein particle levels	4/149	101/18670	0.008749	0.017	0.006221	PLA2G2A/APOE/MPO/HMOX1
GO:0009132	nucleoside diphosphate metabolic process	5/149	158/18670	0.008786	0.017065	0.006245	IGF1/INS/IFNG/TP53/HTR2A
GO:0006349	regulation of gene expression by genetic imprinting	2/149	18/18670	0.008902	0.017171	0.006283	KCNQ1/IGF2
GO:0007221	positive regulation of transcription of Notch receptor target	2/149	18/18670	0.008902	0.017171	0.006283	STAT1/NOTCH1
GO:0007597	blood coagulation, intrinsic pathway	2/149	18/18670	0.008902	0.017171	0.006283	F2/F12
GO:0010713	negative regulation of collagen metabolic process	2/149	18/18670	0.008902	0.017171	0.006283	PPARG/IL6
GO:0021756	striatum development	2/149	18/18670	0.008902	0.017171	0.006283	DRD2/SHANK3
GO:0031065	positive regulation of histone deacetylation	2/149	18/18670	0.008902	0.017171	0.006283	TGFB1/TP53
GO:0031293	membrane protein intracellular domain proteolysis	2/149	18/18670	0.008902	0.017171	0.006283	TGFB1/NFKB1
GO:0042772	DNA damage response, signal transduction resulting in transcription	2/149	18/18670	0.008902	0.017171	0.006283	TP53/CDKN1A
GO:0060546	negative regulation of necroptotic process	2/149	18/18670	0.008902	0.017171	0.006283	SLC25A4/CAV1
GO:0060749	mammary gland alveolus development	2/149	18/18670	0.008902	0.017171	0.006283	CCND1/ESR1
GO:0061377	mammary gland lobule development	2/149	18/18670	0.008902	0.017171	0.006283	CCND1/ESR1
GO:0070633	transepithelial transport	2/149	18/18670	0.008902	0.017171	0.006283	SLC12A2/CFTR
GO:0071625	vocalization behavior	2/149	18/18670	0.008902	0.017171	0.006283	SHANK3/NRXN1

GO:0072079	nephron tubule formation	2/149	18/18670	0.008902	0.017171	0.006283	SOX9/CTNNB1
GO:0090190	positive regulation of branching involved in ureteric bud morphogenesis	2/149	18/18670	0.008902	0.017171	0.006283	SOX9/TGFB1
GO:2000193	positive regulation of fatty acid transport	2/149	18/18670	0.008902	0.017171	0.006283	PLA2G6/IL1B
GO:0030199	collagen fibril organization	3/149	54/18670	0.009179	0.017675	0.006468	COL1A1/SERPINH1/RB1
GO:0032210	regulation of telomere maintenance via telomerase	3/149	54/18670	0.009179	0.017675	0.006468	CTNNB1/MAPK1/SRC
GO:0032663	regulation of interleukin-2 production	3/149	54/18670	0.009179	0.017675	0.006468	IL1A/ABL1/IL1B
GO:0043392	negative regulation of DNA binding	3/149	54/18670	0.009179	0.017675	0.006468	HMOX1/NFKBIA/JUN
GO:0021915	neural tube development	5/149	160/18670	0.009247	0.017783	0.006507	TGFB1/CASP3/NODAL/ABL1/NOTCH1
GO:0030203	glycosaminoglycan metabolic process	5/149	160/18670	0.009247	0.017783	0.006507	TGFB1/FGF2/AKT1/NFKB1/IL1B
GO:0032680	regulation of tumor necrosis factor production	5/149	160/18670	0.009247	0.017783	0.006507	IGF1/LEP/IL10/IFNG/PIK3R1
GO:0030595	leukocyte chemotaxis	6/149	224/18670	0.009259	0.017799	0.006513	CXCL8/SLC12A2/S100A8/IL6/IL10/IL1B
GO:0032652	regulation of interleukin-1 production	4/149	103/18670	0.009359	0.017975	0.006577	IGF1/CASP8/IL10/IFNG
GO:0035601	protein deacylation	4/149	103/18670	0.009359	0.017975	0.006577	MAPT/TGFB1/IFNG/TP53
GO:0045665	negative regulation of neuron differentiation	6/149	225/18670	0.009452	0.018146	0.00664	SLC6A4/APOE/THRB/SOX9/GSK3B/NOTCH1
GO:0018393	internal peptidyl-lysine acetylation	5/149	161/18670	0.009484	0.018191	0.006657	NOS1/SNCA/TGFB1/IL1B/SNAI2

GO:0035967	cellular response to topologically incorrect protein	5/149	161/18670	0.009484	0.018191	0.006657	CXCL8/NFE2L2/CCND1/PIK3R1/BAX
GO:0002090	regulation of receptor internalization	3/149	55/18670	0.009653	0.018476	0.006761	DRD2/FMR1/DRD4
GO:0042304	regulation of fatty acid biosynthetic process	3/149	55/18670	0.009653	0.018476	0.006761	PTGS2/IL1B/AVP
GO:0043954	cellular component maintenance	3/149	55/18670	0.009653	0.018476	0.006761	APOE/GRIN2B/INS
GO:0048016	inositol phosphate-mediated signaling	3/149	55/18670	0.009653	0.018476	0.006761	IGF1/TNF/GSK3B
GO:0070830	bicellular tight junction assembly	3/149	55/18670	0.009653	0.018476	0.006761	TNF/GPBAR1/SNAI2
GO:0001523	retinoid metabolic process	4/149	104/18670	0.009674	0.018501	0.00677	APOE/CYP1A1/ADH1C/CYP3A4
GO:0098732	macromolecule deacylation	4/149	104/18670	0.009674	0.018501	0.00677	MAPT/TGFB1/IFNG/TP53
GO:0001696	gastric acid secretion	2/149	19/18670	0.009897	0.018712	0.006847	KCNQ1/TRPV1
GO:0002363	alpha-beta T cell lineage commitment	2/149	19/18670	0.009897	0.018712	0.006847	BCL2/IL6
GO:0002726	positive regulation of T cell cytokine production	2/149	19/18670	0.009897	0.018712	0.006847	IL6/IL1B
GO:0002922	positive regulation of humoral immune response	2/149	19/18670	0.009897	0.018712	0.006847	TNF/IL1B
GO:0006833	water transport	2/149	19/18670	0.009897	0.018712	0.006847	CFTR/AVP
GO:0010544	negative regulation of platelet activation	2/149	19/18670	0.009897	0.018712	0.006847	APOE/F2
GO:0010663	positive regulation of striated muscle cell apoptotic process	2/149	19/18670	0.009897	0.018712	0.006847	HMGCR/TP53
GO:0010666	positive regulation of cardiac muscle cell apoptotic process	2/149	19/18670	0.009897	0.018712	0.006847	HMGCR/TP53

GO:0016048	detection of temperature stimulus	2/149	19/18670	0.009897	0.018712	0.006847	TRPV1/HTR2A
GO:0019184	nonribosomal peptide biosynthetic process	2/149	19/18670	0.009897	0.018712	0.006847	NFE2L2/GGT1
GO:0032026	response to magnesium ion	2/149	19/18670	0.009897	0.018712	0.006847	CCND1/SNCA
GO:0032303	regulation of icosanoid secretion	2/149	19/18670	0.009897	0.018712	0.006847	PLA2G6/IL1B
GO:0032700	negative regulation of interleukin-17 production	2/149	19/18670	0.009897	0.018712	0.006847	TGFB1/IFNG
GO:0034138	toll-like receptor 3 signaling pathway	2/149	19/18670	0.009897	0.018712	0.006847	CASP8/CAV1
GO:0045076	regulation of interleukin-2 biosynthetic process	2/149	19/18670	0.009897	0.018712	0.006847	IL1A/IL1B
GO:0060219	camera-type eye photoreceptor cell differentiation	2/149	19/18670	0.009897	0.018712	0.006847	THRB/SOX9
GO:0060231	mesenchymal to epithelial transition	2/149	19/18670	0.009897	0.018712	0.006847	CTNNB1/STAT1
GO:0060716	labyrinthine layer blood vessel development	2/149	19/18670	0.009897	0.018712	0.006847	MAPK1/AKT1
GO:0060973	cell migration involved in heart development	2/149	19/18670	0.009897	0.018712	0.006847	SNAI2/NOTCH1
GO:0071800	podosome assembly	2/149	19/18670	0.009897	0.018712	0.006847	TNF/SRC
GO:0072074	kidney mesenchyme development	2/149	19/18670	0.009897	0.018712	0.006847	MYC/STAT1
GO:0098911	regulation of ventricular cardiac muscle cell action potential	2/149	19/18670	0.009897	0.018712	0.006847	CACNA1C/CAV1
GO:1900409	positive regulation of cellular response to oxidative stress	2/149	19/18670	0.009897	0.018712	0.006847	TNF/SOD1
GO:1905288	vascular associated smooth muscle cell apoptotic process	2/149	19/18670	0.009897	0.018712	0.006847	IGF1/PPARG

GO:1905459	regulation of vascular associated smooth muscle cell apoptotic process	2/149	19/18670	0.009897	0.018712	0.006847	IGF1/PPARG
GO:1905939	regulation of gonad development	2/149	19/18670	0.009897	0.018712	0.006847	SOX9/SRC
GO:2000010	positive regulation of protein localization to cell surface	2/149	19/18670	0.009897	0.018712	0.006847	TNF/AKT1
GO:0032640	tumor necrosis factor production	5/149	163/18670	0.009969	0.018824	0.006888	IGF1/LEP/IL10/IFNG/PIK3R1
GO:0099173	postsynapse organization	5/149	163/18670	0.009969	0.018824	0.006888	APOE/GRIN2B/SHANK3/NRXN1/INS
GO:1903555	regulation of tumor necrosis factor superfamily cytokine production	5/149	163/18670	0.009969	0.018824	0.006888	IGF1/LEP/IL10/IFNG/PIK3R1
GO:0001841	neural tube formation	4/149	105/18670	0.009996	0.018836	0.006892	TGFB1/CASP3/NODAL/ABL1
GO:0006641	triglyceride metabolic process	4/149	105/18670	0.009996	0.018836	0.006892	APOE/CYP2E1/CAV1/CAT
GO:0008593	regulation of Notch signaling pathway	4/149	105/18670	0.009996	0.018836	0.006892	EGFR/AKT1/NFKBIA/NOTCH1
GO:0045185	maintenance of protein location	4/149	105/18670	0.009996	0.018836	0.006892	IL10/CAV1/AKT1/NFKBIA
GO:0055017	cardiac muscle tissue growth	4/149	105/18670	0.009996	0.018836	0.006892	IGF1/MAPK1/FGF2/NOTCH1
GO:0048285	organelle fission	9/149	449/18670	0.010064	0.018956	0.006936	IGF1/MAPT/IL1A/TGFB1/KDR/INS/IGF2/RB1/IL1B
GO:0002381	immunoglobulin production involved in immunoglobulin mediated immune response	3/149	56/18670	0.01014	0.019067	0.006977	TGFB1/IL10/IL2
GO:0006749	glutathione metabolic process	3/149	56/18670	0.01014	0.019067	0.006977	NFE2L2/GGT1/SOD1

GO:0006977	DNA damage response, signal transduction by p53 class mediator resulting in cell cycle arrest	3/149	56/18670	0.01014	0.019067	0.006977	TP53/CDKN1A/BAX
GO:0120192	tight junction assembly	3/149	56/18670	0.01014	0.019067	0.006977	TNF/GPBAR1/SNAI2
GO:0003300	cardiac muscle hypertrophy	4/149	106/18670	0.010325	0.019399	0.007098	IGF1/LEP/PARP1/NOTCH1
GO:0044774	mitotic DNA integrity checkpoint	4/149	106/18670	0.010325	0.019399	0.007098	CCND1/TP53/CDKN1A/BAX
GO:0009123	nucleoside monophosphate metabolic process	8/149	375/18670	0.010572	0.019854	0.007265	IGF1/SNCA/TGFB1/PARP1/INS/IFNG/TP53/HTR2A
GO:0002066	columnar/cuboidal epithelial cell development	3/149	57/18670	0.010642	0.019913	0.007287	GSK3B/SOD1/CDKN1A
GO:0010665	regulation of cardiac muscle cell apoptotic process	3/149	57/18670	0.010642	0.019913	0.007287	NFE2L2/HMGCR/TP53
GO:0033344	cholesterol efflux	3/149	57/18670	0.010642	0.019913	0.007287	APOE/PON1/NFKBIA
GO:0048008	platelet-derived growth factor receptor signaling pathway	3/149	57/18670	0.010642	0.019913	0.007287	SNCA/ABL1/SRC
GO:0060043	regulation of cardiac muscle cell proliferation	3/149	57/18670	0.010642	0.019913	0.007287	MAPK1/FGF2/NOTCH1
GO:0071398	cellular response to fatty acid	3/149	57/18670	0.010642	0.019913	0.007287	PPARG/SRC/AKT1
GO:0072431	signal transduction involved in mitotic G1 DNA damage checkpoint	3/149	57/18670	0.010642	0.019913	0.007287	TP53/CDKN1A/BAX
GO:1902400	intracellular signal transduction involved in G1 DNA damage checkpoint	3/149	57/18670	0.010642	0.019913	0.007287	TP53/CDKN1A/BAX

GO:0001909	leukocyte mediated cytotoxicity	4/149	107/18670	0.010661	0.019913	0.007287	NOS2/LEP/F2/ICAM1
GO:0030330	DNA damage response, signal transduction by p53 class mediator	4/149	107/18670	0.010661	0.019913	0.007287	TP53/CDKN1A/SNAI2/BAX
GO:0043502	regulation of muscle adaptation	4/149	107/18670	0.010661	0.019913	0.007287	IGF1/PARP1/NOTCH1/SCN5A
GO:0048524	positive regulation of viral process	4/149	107/18670	0.010661	0.019913	0.007287	APOE/FMR1/JUN/NOTCH1
GO:0050905	neuromuscular process	4/149	107/18670	0.010661	0.019913	0.007287	DRD2/NRXN1/GRIN1/ABL1
GO:1903320	regulation of protein modification by small protein conjugation or removal	6/149	231/18670	0.010669	0.01992	0.007289	CTNNB1/AVPR2/HSP90AA1/CAV1/ABL1/AKT1
GO:0001502	cartilage condensation	2/149	20/18670	0.01094	0.020189	0.007388	SOX9/THRA
GO:0002320	lymphoid progenitor cell differentiation	2/149	20/18670	0.01094	0.020189	0.007388	BCL2/NOTCH1
GO:0002374	cytokine secretion involved in immune response	2/149	20/18670	0.01094	0.020189	0.007388	TNF/IL10
GO:0006525	arginine metabolic process	2/149	20/18670	0.01094	0.020189	0.007388	NOS2/NOS1
GO:0007620	copulation	2/149	20/18670	0.01094	0.020189	0.007388	SLC6A4/AVP
GO:0010560	positive regulation of glycoprotein biosynthetic process	2/149	20/18670	0.01094	0.020189	0.007388	IGF1/CTNNB1
GO:0019373	epoxygenase P450 pathway	2/149	20/18670	0.01094	0.020189	0.007388	CYP1A1/CYP2E1
GO:0030220	platelet formation	2/149	20/18670	0.01094	0.020189	0.007388	CASP3/CASP9
GO:0032042	mitochondrial DNA metabolic process	2/149	20/18670	0.01094	0.020189	0.007388	PARP1/TP53

GO:00324 62	regulation of protein homooligomerization	2/149	20/18670	0.01094	0.02018 9	0.00738 8	APOE/SRC
GO:00324 95	response to muramyl dipeptide	2/149	20/18670	0.01094	0.02018 9	0.00738 8	NFKBIA/NOTCH1
GO:00336 30	positive regulation of cell adhesion mediated by integrin	2/149	20/18670	0.01094	0.02018 9	0.00738 8	RET/P2RY12
GO:00343 93	positive regulation of smooth muscle cell apoptotic process	2/149	20/18670	0.01094	0.02018 9	0.00738 8	PPARG/IFNG
GO:00354 58	cellular response to interferon-beta	2/149	20/18670	0.01094	0.02018 9	0.00738 8	IRF1/STAT1
GO:00433 69	CD4-positive or CD8-positive, alpha-beta T cell lineage commitment	2/149	20/18670	0.01094	0.02018 9	0.00738 8	BCL2/IL6
GO:00433 71	negative regulation of CD4-positive, alpha-beta T cell differentiation	2/149	20/18670	0.01094	0.02018 9	0.00738 8	IL2/JAK3
GO:00439 31	ossification involved in bone maturation	2/149	20/18670	0.01094	0.02018 9	0.00738 8	IGF1/LEP
GO:00450 19	negative regulation of nitric oxide biosynthetic process	2/149	20/18670	0.01094	0.02018 9	0.00738 8	IL10/CAV1
GO:00456 55	regulation of monocyte differentiation	2/149	20/18670	0.01094	0.02018 9	0.00738 8	MYC/JUN
GO:00487 45	smooth muscle tissue development	2/149	20/18670	0.01094	0.02018 9	0.00738 8	SOX9/MYLK
GO:00517 97	regulation of hair follicle development	2/149	20/18670	0.01094	0.02018 9	0.00738 8	TNF/CTNNB1
GO:00600 08	Sertoli cell differentiation	2/149	20/18670	0.01094	0.02018 9	0.00738 8	SOX9/ICAM1
GO:00602 61	positive regulation of transcription initiation from RNA polymerase II promoter	2/149	20/18670	0.01094	0.02018 9	0.00738 8	ESR1/TP53

GO:0072234	metanephric nephron tubule development	2/149	20/18670	0.01094	0.020189	0.007388	SOX9/STAT1
GO:0097709	connective tissue replacement	2/149	20/18670	0.01094	0.020189	0.007388	IL1A/TGFB1
GO:0098698	postsynaptic specialization assembly	2/149	20/18670	0.01094	0.020189	0.007388	SHANK3/NRXN1
GO:1903204	negative regulation of oxidative stress-induced neuron death	2/149	20/18670	0.01094	0.020189	0.007388	CTNNB1/IL10
GO:1904406	negative regulation of nitric oxide metabolic process	2/149	20/18670	0.01094	0.020189	0.007388	IL10/CAV1
GO:0035051	cardiocyte differentiation	5/149	167/18670	0.010991	0.020275	0.007419	IGF1/TGFB1/MAPK1/EGFR/NOTCH1
GO:0046916	cellular transition metal ion homeostasis	4/149	108/18670	0.011004	0.020291	0.007425	MYC/S100A8/HMOX1/SOD1
GO:0030520	intracellular estrogen receptor signaling pathway	3/149	58/18670	0.011158	0.020524	0.00751	ESR1/PARP1/SRC
GO:0035306	positive regulation of dephosphorylation	3/149	58/18670	0.011158	0.020524	0.00751	TGFB1/IFNG/SRC
GO:0035904	aorta development	3/149	58/18670	0.011158	0.020524	0.00751	LEP/MYLK/NOTCH1
GO:0042093	T-helper cell differentiation	3/149	58/18670	0.011158	0.020524	0.00751	IL6/IL2/JAK3
GO:0098930	axonal transport	3/149	58/18670	0.011158	0.020524	0.00751	MAPT/FMR1/SOD1
GO:1903749	positive regulation of establishment of protein localization to mitochondrion	3/149	58/18670	0.011158	0.020524	0.00751	BCL2/CASP8/TP53
GO:0071706	tumor necrosis factor superfamily cytokine production	5/149	168/18670	0.011257	0.020698	0.007574	IGF1/LEP/IL10/IFNG/PIK3R1
GO:0014897	striated muscle hypertrophy	4/149	109/18670	0.011354	0.020868	0.007636	IGF1/LEP/PARP1/NOTCH1

GO:0018394	peptidyl-lysine acetylation	5/149	169/18670	0.011528	0.02117	0.007746	NOS1/SNCA/TGFB1/IL1B/SNAI2
GO:0038095	Fc-epsilon receptor signaling pathway	5/149	169/18670	0.011528	0.02117	0.007746	MAPK1/FOS/PIK3R1/NFKB1/JUN
GO:0045089	positive regulation of innate immune response	8/149	381/18670	0.011552	0.021205	0.007759	IRF1/CASP8/ESR1/S100A8/CAV1/SRC/NFKB1/NFKBIA
GO:0010662	regulation of striated muscle cell apoptotic process	3/149	59/18670	0.011688	0.021412	0.007835	NFE2L2/HMGCR/TP53
GO:0045824	negative regulation of innate immune response	3/149	59/18670	0.011688	0.021412	0.007835	PPARG/DRD2/INS
GO:0072413	signal transduction involved in mitotic cell cycle checkpoint	3/149	59/18670	0.011688	0.021412	0.007835	TP53/CDKN1A/BAX
GO:1902402	signal transduction involved in mitotic DNA damage checkpoint	3/149	59/18670	0.011688	0.021412	0.007835	TP53/CDKN1A/BAX
GO:1902403	signal transduction involved in mitotic DNA integrity checkpoint	3/149	59/18670	0.011688	0.021412	0.007835	TP53/CDKN1A/BAX
GO:0061387	regulation of extent of cell growth	4/149	110/18670	0.011711	0.021445	0.007847	APOE/MAPT/GSK3B/ABL1
GO:0006022	aminoglycan metabolic process	5/149	170/18670	0.011803	0.021604	0.007905	TGFB1/FGF2/AKT1/NFKB1/IL1B
GO:0009713	catechol-containing compound biosynthetic process	2/149	21/18670	0.012029	0.021856	0.007998	SNCA/PAH
GO:0032682	negative regulation of chemokine production	2/149	21/18670	0.012029	0.021856	0.007998	IL6/IL10
GO:0032891	negative regulation of organic acid transport	2/149	21/18670	0.012029	0.021856	0.007998	LEP/AKT1
GO:0033141	positive regulation of peptidyl-serine phosphorylation of STAT protein	2/149	21/18670	0.012029	0.021856	0.007998	RET/IFNG

GO:0035162	embryonic hemopoiesis	2/149	21/18670	0.012029	0.021856	0.007998	KDR/TPO
GO:0035357	peroxisome proliferator activated receptor signaling pathway	2/149	21/18670	0.012029	0.021856	0.007998	LEP/PPARG
GO:0035743	CD4-positive, alpha-beta T cell cytokine production	2/149	21/18670	0.012029	0.021856	0.007998	IL6/IL1B
GO:0036344	platelet morphogenesis	2/149	21/18670	0.012029	0.021856	0.007998	CASP3/CASP9
GO:0036499	PERK-mediated unfolded protein response	2/149	21/18670	0.012029	0.021856	0.007998	CXCL8/NFE2L2
GO:0042135	neurotransmitter catabolic process	2/149	21/18670	0.012029	0.021856	0.007998	ACHE/MAOB
GO:0042423	catecholamine biosynthetic process	2/149	21/18670	0.012029	0.021856	0.007998	SNCA/PAH
GO:0046716	muscle cell cellular homeostasis	2/149	21/18670	0.012029	0.021856	0.007998	TGFB1/SOD1
GO:0061213	positive regulation of mesonephros development	2/149	21/18670	0.012029	0.021856	0.007998	SOX9/TGFB1
GO:0072111	cell proliferation involved in kidney development	2/149	21/18670	0.012029	0.021856	0.007998	MYC/STAT1
GO:0098743	cell aggregation	2/149	21/18670	0.012029	0.021856	0.007998	SOX9/THRA
GO:1902307	positive regulation of sodium ion transmembrane transport	2/149	21/18670	0.012029	0.021856	0.007998	NOS1/DRD4
GO:1902884	positive regulation of response to oxidative stress	2/149	21/18670	0.012029	0.021856	0.007998	TNF/SOD1
GO:2000737	negative regulation of stem cell differentiation	2/149	21/18670	0.012029	0.021856	0.007998	NFE2L2/NOTCH1
GO:0014896	muscle hypertrophy	4/149	111/18670	0.012075	0.021918	0.00802	IGF1/LEP/PARP1/NOTCH1

GO:0072676	lymphocyte migration	4/149	111/18670	0.012075	0.021918	0.00802	RET/SLC12A2/ICAM1/AKT1
GO:0016241	regulation of macroautophagy	5/149	171/18670	0.012082	0.021918	0.00802	CASP3/KDR/HMOX1/TP53/AKT1
GO:0043901	negative regulation of multi-organism process	5/149	171/18670	0.012082	0.021918	0.00802	IGF1/TNF/STAT1/NODAL/JUN
GO:0010659	cardiac muscle cell apoptotic process	3/149	60/18670	0.012233	0.022146	0.008104	NFE2L2/HMGCR/TP53
GO:0033013	tetrapyrrole metabolic process	3/149	60/18670	0.012233	0.022146	0.008104	CYP1A1/HMOX1/FECH
GO:0051893	regulation of focal adhesion assembly	3/149	60/18670	0.012233	0.022146	0.008104	KDR/ABL1/SRC
GO:0090109	regulation of cell-substrate junction assembly	3/149	60/18670	0.012233	0.022146	0.008104	KDR/ABL1/SRC
GO:0120193	tight junction organization	3/149	60/18670	0.012233	0.022146	0.008104	TNF/GPBAR1/SNAI2
GO:0008544	epidermis development	9/149	464/18670	0.012276	0.022217	0.00813	BCL2/SOX9/TNF/CTNNB1/CASP3/EGFR/SOD1/ACVR1B/NOTCH1
GO:0032874	positive regulation of stress-activated MAPK cascade	5/149	172/18670	0.012366	0.02237	0.008186	LEP/OPRK1/TNF/HMGCR/IL1B
GO:0014902	myotube differentiation	4/149	112/18670	0.012447	0.022479	0.008226	IGF1/NOS1/BCL2/NOTCH1
GO:0031109	microtubule polymerization or depolymerization	4/149	112/18670	0.012447	0.022479	0.008226	MET/MAPT/SNCA/ABL1
GO:0048593	camera-type eye morphogenesis	4/149	112/18670	0.012447	0.022479	0.008226	THRB/SOX9/CTNNB1/BAX
GO:0060419	heart growth	4/149	112/18670	0.012447	0.022479	0.008226	IGF1/MAPK1/FGF2/NOTCH1
GO:0070304	positive regulation of stress-activated protein kinase signaling cascade	5/149	173/18670	0.012655	0.022846	0.00836	LEP/OPRK1/TNF/HMGCR/IL1B
GO:0006879	cellular iron ion homeostasis	3/149	61/18670	0.012792	0.023037	0.00843	MYC/HMOX1/SOD1

GO:0030521	androgen receptor signaling pathway	3/149	61/18670	0.012792	0.023037	0.00843	CTNNB1/NODAL/RB1
GO:0034113	heterotypic cell-cell adhesion	3/149	61/18670	0.012792	0.023037	0.00843	TNF/IL10/IL1B
GO:0042733	embryonic digit morphogenesis	3/149	61/18670	0.012792	0.023037	0.00843	CTNNB1/NOTCH1/BAX
GO:0045123	cellular extravasation	3/149	61/18670	0.012792	0.023037	0.00843	LEP/TNF/ICAM1
GO:2000514	regulation of CD4-positive, alpha-beta T cell activation	3/149	61/18670	0.012792	0.023037	0.00843	IL2/IFNG/JAK3
GO:0006925	inflammatory cell apoptotic process	2/149	22/18670	0.013163	0.023535	0.008612	IL6/FASLG
GO:0007063	regulation of sister chromatid cohesion	2/149	22/18670	0.013163	0.023535	0.008612	CTNNB1/RB1
GO:0010155	regulation of proton transport	2/149	22/18670	0.013163	0.023535	0.008612	IL13/DRD4
GO:0010869	regulation of receptor biosynthetic process	2/149	22/18670	0.013163	0.023535	0.008612	PPARG/IFNG
GO:0021801	cerebral cortex radial glia guided migration	2/149	22/18670	0.013163	0.023535	0.008612	CTNNB1/P2RY12
GO:0022030	telencephalon glial cell migration	2/149	22/18670	0.013163	0.023535	0.008612	CTNNB1/P2RY12
GO:0032469	endoplasmic reticulum calcium ion homeostasis	2/149	22/18670	0.013163	0.023535	0.008612	BCL2/BAX
GO:0033622	integrin activation	2/149	22/18670	0.013163	0.023535	0.008612	P2RY12/SRC
GO:0035809	regulation of urine volume	2/149	22/18670	0.013163	0.023535	0.008612	DRD2/AVPR2
GO:0042094	interleukin-2 biosynthetic process	2/149	22/18670	0.013163	0.023535	0.008612	IL1A/IL1B
GO:0043496	regulation of protein homodimerization activity	2/149	22/18670	0.013163	0.023535	0.008612	BCL2/BAX
GO:0044321	response to leptin	2/149	22/18670	0.013163	0.023535	0.008612	LEP/CCND1

GO:00600 65	uterus development	2/149	22/18670	0.01316 3	0.02353 5	0.00861 2	ESR1/SRC
GO:00605 47	negative regulation of necrotic cell death	2/149	22/18670	0.01316 3	0.02353 5	0.00861 2	SLC25A4/CAV1
GO:00713 59	cellular response to dsRNA	2/149	22/18670	0.01316 3	0.02353 5	0.00861 2	CAV1/NFKB1
GO:00713 79	cellular response to prostaglandin stimulus	2/149	22/18670	0.01316 3	0.02353 5	0.00861 2	PPARG/AKT1
GO:00901 89	regulation of branching involved in ureteric bud morphogenesis	2/149	22/18670	0.01316 3	0.02353 5	0.00861 2	SOX9/TGFB1
GO:19019 84	negative regulation of protein acetylation	2/149	22/18670	0.01316 3	0.02353 5	0.00861 2	SNCA/GSK3B
GO:00603 49	bone morphogenesis	4/149	114/18670	0.01321 1	0.02361 3	0.00864	COL1A1/SOX9/TGFB1/SERPINH1
GO:00109 21	regulation of phosphatase activity	5/149	175/18670	0.01324 5	0.02366 4	0.00865 9	TNF/DRD2/GSK3B/IFNG/IGFBP3
GO:00106 58	striated muscle cell apoptotic process	3/149	62/18670	0.01336 5	0.02379 2	0.00870 6	NFE2L2/HMGCR/TP53
GO:00316 40	killing of cells of other organism	3/149	62/18670	0.01336 5	0.02379 2	0.00870 6	NOS2/F2/IFNG
GO:00326 23	interleukin-2 production	3/149	62/18670	0.01336 5	0.02379 2	0.00870 6	IL1A/ABL1/IL1B
GO:00328 35	glomerulus development	3/149	62/18670	0.01336 5	0.02379 2	0.00870 6	BCL2/RET/NOTCH1
GO:00443 64	disruption of cells of other organism	3/149	62/18670	0.01336 5	0.02379 2	0.00870 6	NOS2/F2/IFNG
GO:00488 57	neural nucleus development	3/149	62/18670	0.01336 5	0.02379 2	0.00870 6	BCL2/NKX2-1/MAOB
GO:00603 93	regulation of pathway-restricted SMAD protein phosphorylation	3/149	62/18670	0.01336 5	0.02379 2	0.00870 6	TGFB1/NODAL/ACVR1B
GO:00700 59	intrinsic apoptotic signaling pathway in response to endoplasmic reticulum stress	3/149	62/18670	0.01336 5	0.02379 2	0.00870 6	BCL2/TP53/BAX

GO:1902305	regulation of sodium ion transmembrane transport	3/149	62/18670	0.013365	0.023792	0.008706	NOS1/DRD4/SCN5A
GO:0002429	immune response-activating cell surface receptor signaling pathway	9/149	473/18670	0.013768	0.0245	0.008965	BCL2/PLA2G6/HSP90AA1/MAPK1/ABL1/SRC/PIK3R1/NFKB1/BAX
GO:0060491	regulation of cell projection assembly	5/149	177/18670	0.013854	0.024643	0.009017	P2RY12/FMR1/ICAM1/CAV1/SRC
GO:0031646	positive regulation of neurological system process	3/149	63/18670	0.013953	0.02476	0.00906	SHANK3/NRXN1/GRIN1
GO:0034394	protein localization to cell surface	3/149	63/18670	0.013953	0.02476	0.00906	TNF/CTNNB1/AKT1
GO:0035418	protein localization to synapse	3/149	63/18670	0.013953	0.02476	0.00906	MAPT/NRXN1/GRIN1
GO:0046530	photoreceptor cell differentiation	3/149	63/18670	0.013953	0.02476	0.00906	THRB/SOX9/NOTCH1
GO:0046637	regulation of alpha-beta T cell differentiation	3/149	63/18670	0.013953	0.02476	0.00906	IL2/IFNG/JAK3
GO:0090181	regulation of cholesterol metabolic process	3/149	63/18670	0.013953	0.02476	0.00906	APOE/HMGCR/SOD1
GO:0070252	actin-mediated cell contraction	4/149	116/18670	0.014005	0.024843	0.009091	KCNQ1/CACNA1C/CAV1/SCN5A
GO:0050821	protein stabilization	5/149	178/18670	0.014165	0.025117	0.009191	IGF1/HSP90AA1/TP53/CDKN1A/PIK3R1
GO:0006650	glycerophospholipid metabolic process	7/149	319/18670	0.014241	0.025241	0.009236	PLA2G2A/PON1/PLA2G6/ACHE/FGF2/PIK3R1/HTR2A
GO:0010447	response to acidic pH	2/149	23/18670	0.014342	0.02528	0.00925	TRPV1/SRC
GO:0032799	low-density lipoprotein receptor particle metabolic process	2/149	23/18670	0.014342	0.02528	0.00925	APOE/PPARG
GO:0033139	regulation of peptidyl-serine phosphorylation of STAT protein	2/149	23/18670	0.014342	0.02528	0.00925	RET/IFNG

GO:0034695	response to prostaglandin E	2/149	23/18670	0.014342	0.02528	0.00925	PPARG/AKT1
GO:0036037	CD8-positive, alpha-beta T cell activation	2/149	23/18670	0.014342	0.02528	0.00925	IRF1/BCL2
GO:0060445	branching involved in salivary gland morphogenesis	2/149	23/18670	0.014342	0.02528	0.00925	TNF/SNAI2
GO:0070977	bone maturation	2/149	23/18670	0.014342	0.02528	0.00925	IGF1/LEP
GO:0071305	cellular response to vitamin D	2/149	23/18670	0.014342	0.02528	0.00925	SNAI2/CYP24A1
GO:0072170	metanephric tubule development	2/149	23/18670	0.014342	0.02528	0.00925	SOX9/STAT1
GO:0072243	metanephric nephron epithelium development	2/149	23/18670	0.014342	0.02528	0.00925	SOX9/STAT1
GO:0086012	membrane depolarization during cardiac muscle cell action potential	2/149	23/18670	0.014342	0.02528	0.00925	CACNA1C/SCN5A
GO:0090140	regulation of mitochondrial fission	2/149	23/18670	0.014342	0.02528	0.00925	MAPT/KDR
GO:1901889	negative regulation of cell junction assembly	2/149	23/18670	0.014342	0.02528	0.00925	TNF/SRC
GO:1903429	regulation of cell maturation	2/149	23/18670	0.014342	0.02528	0.00925	BCL2/RET
GO:0010508	positive regulation of autophagy	4/149	117/18670	0.014413	0.025366	0.009282	GSK3B/KDR/HMOX1/IFNG
GO:0043666	regulation of phosphoprotein phosphatase activity	4/149	117/18670	0.014413	0.025366	0.009282	TNF/DRD2/GSK3B/IGFBP3
GO:0051153	regulation of striated muscle cell differentiation	4/149	117/18670	0.014413	0.025366	0.009282	IGF1/BCL2/TGFB1/NOTCH1
GO:1901222	regulation of NIK/NF-kappaB signaling	4/149	117/18670	0.014413	0.025366	0.009282	TNF/EGFR/IL1B/NFKBIA
GO:0048736	appendage development	5/149	179/18670	0.014481	0.025466	0.009318	SOX9/CTNNB1/CACNA1C/NOTCH1/BAX

GO:0060173	limb development	5/149	179/18670	0.014481	0.025466	0.009318	SOX9/CTNNB1/CACNA1C/NOTCH1/BAX
GO:0043297	apical junction assembly	3/149	64/18670	0.014555	0.025575	0.009358	TNF/GPBAR1/SNAI2
GO:0045669	positive regulation of osteoblast differentiation	3/149	64/18670	0.014555	0.025575	0.009358	IGF1/CTNNB1/IL6
GO:0016311	dephosphorylation	9/149	478/18670	0.014652	0.025736	0.009417	PON1/BCL2/TNF/DRD2/TGFB1/GSK3B/IFNG/SRC/IGFBP3
GO:0046434	organophosphate catabolic process	6/149	248/18670	0.014717	0.025819	0.009448	IGF1/PON1/INS/IFNG/TP53/HTR2A
GO:0065004	protein-DNA complex assembly	6/149	248/18670	0.014717	0.025819	0.009448	SOX9/ESR1/PARP1/RB1/THRA/TP53
GO:1901991	negative regulation of mitotic cell cycle phase transition	6/149	248/18670	0.014717	0.025819	0.009448	BCL2/CCND1/RB1/TP53/CDKN1A/BAX
GO:0014823	response to activity	3/149	65/18670	0.015171	0.026596	0.009732	LEP/IL10/CAT
GO:0060389	pathway-restricted SMAD protein phosphorylation	3/149	65/18670	0.015171	0.026596	0.009732	TGFB1/NODAL/ACVR1B
GO:0006338	chromatin remodeling	5/149	182/18670	0.015458	0.027031	0.009891	SOX9/CTNNB1/ESR1/MYC/RB1
GO:0009166	nucleotide catabolic process	5/149	182/18670	0.015458	0.027031	0.009891	IGF1/INS/IFNG/TP53/HTR2A
GO:0045017	glycerolipid biosynthetic process	6/149	251/18670	0.015528	0.027031	0.009891	PLA2G2A/PLA2G6/ACHE/FGF2/PIK3R1/HTR2A
GO:0001919	regulation of receptor recycling	2/149	24/18670	0.015564	0.027031	0.009891	SNCA/ACHE
GO:0002092	positive regulation of receptor internalization	2/149	24/18670	0.015564	0.027031	0.009891	DRD2/FMR1
GO:0002861	regulation of inflammatory response to antigenic stimulus	2/149	24/18670	0.015564	0.027031	0.009891	TNF/IL10
GO:0003272	endocardial cushion formation	2/149	24/18670	0.015564	0.027031	0.009891	SNAI2/NOTCH1

GO:0010640	regulation of platelet-derived growth factor receptor signaling pathway	2/149	24/18670	0.015564	0.027031	0.009891	SNCA/SRC
GO:0021544	subpallium development	2/149	24/18670	0.015564	0.027031	0.009891	DRD2/SHANK3
GO:0021895	cerebral cortex neuron differentiation	2/149	24/18670	0.015564	0.027031	0.009891	DRD2/NKX2-1
GO:0032925	regulation of activin receptor signaling pathway	2/149	24/18670	0.015564	0.027031	0.009891	NODAL/ACVR1B
GO:0033081	regulation of T cell differentiation in thymus	2/149	24/18670	0.015564	0.027031	0.009891	SOD1/ERBB2
GO:0044346	fibroblast apoptotic process	2/149	24/18670	0.015564	0.027031	0.009891	MYC/TP53
GO:0045603	positive regulation of endothelial cell differentiation	2/149	24/18670	0.015564	0.027031	0.009891	CTNNB1/NOTCH1
GO:0046639	negative regulation of alpha-beta T cell differentiation	2/149	24/18670	0.015564	0.027031	0.009891	IL2/JAK3
GO:0046697	decidualization	2/149	24/18670	0.015564	0.027031	0.009891	PTGS2/MAPK1
GO:0051220	cytoplasmic sequestering of protein	2/149	24/18670	0.015564	0.027031	0.009891	IL10/NFKBIA
GO:0060343	trabecula formation	2/149	24/18670	0.015564	0.027031	0.009891	MMP2/COL1A1
GO:0060396	growth hormone receptor signaling pathway	2/149	24/18670	0.015564	0.027031	0.009891	JAK3/PIK3R1
GO:0071677	positive regulation of mononuclear cell migration	2/149	24/18670	0.015564	0.027031	0.009891	TNF/TGFB1
GO:0086064	cell communication by electrical coupling involved in cardiac conduction	2/149	24/18670	0.015564	0.027031	0.009891	CACNA1C/CAV1

GO:0099563	modification of synaptic structure	2/149	24/18670	0.015564	0.027031	0.009891	FMR1/ABL1
GO:1903020	positive regulation of glycoprotein metabolic process	2/149	24/18670	0.015564	0.027031	0.009891	IGF1/CTNNB1
GO:1903649	regulation of cytoplasmic transport	2/149	24/18670	0.015564	0.027031	0.009891	MAPK1/SRC
GO:0006732	coenzyme metabolic process	8/149	403/18670	0.015725	0.0273	0.009989	IGF1/PTGS2/SNCA/HMGCR/INS/IFNG/TP53/HTR2A
GO:0015800	acidic amino acid transport	3/149	66/18670	0.015803	0.027381	0.010019	SNCA/TRPV1/AVP
GO:0021872	forebrain generation of neurons	3/149	66/18670	0.015803	0.027381	0.010019	DRD2/SHANK3/NKX2-1
GO:1901607	alpha-amino acid biosynthetic process	3/149	66/18670	0.015803	0.027381	0.010019	GGT1/GATM/PAH
GO:1904888	cranial skeletal system development	3/149	66/18670	0.015803	0.027381	0.010019	CTNNB1/TGFB1/NODAL
GO:1905207	regulation of cardiocyte differentiation	3/149	66/18670	0.015803	0.027381	0.010019	IGF1/TGFB1/EGFR
GO:0043312	neutrophil degranulation	9/149	485/18670	0.015961	0.027644	0.010115	PLAU/MPO/CTSD/S100A8/HSP90AA1/MAPK1/CAT/NFKB1/MMP9
GO:0001704	formation of primary germ layer	4/149	121/18670	0.016121	0.02791	0.010213	MMP2/CTNNB1/NODAL/MMP9
GO:0019748	secondary metabolic process	3/149	67/18670	0.016449	0.028446	0.010409	BCL2/CYP1A1/NFE2L2
GO:0060038	cardiac muscle cell proliferation	3/149	67/18670	0.016449	0.028446	0.010409	MAPK1/FGF2/NOTCH1
GO:1903391	regulation of adherens junction organization	3/149	67/18670	0.016449	0.028446	0.010409	KDR/ABL1/SRC
GO:0002283	neutrophil activation involved in immune response	9/149	488/18670	0.016547	0.028604	0.010467	PLAU/MPO/CTSD/S100A8/HSP90AA1/MAPK1/CAT/NFKB1/MMP9
GO:0032434	regulation of proteasomal ubiquitin-dependent protein catabolic process	4/149	122/18670	0.016566	0.028627	0.010475	NFE2L2/GSK3B/CAV1/AKT1

GO:0009404	toxin metabolic process	2/149	25/18670	0.01683	0.028893	0.010573	CYP1A1/NFE2L2
GO:0014829	vascular smooth muscle contraction	2/149	25/18670	0.01683	0.028893	0.010573	CHRM3/HTR2A
GO:0031639	plasminogen activation	2/149	25/18670	0.01683	0.028893	0.010573	PLAU/F12
GO:0032104	regulation of response to extracellular stimulus	2/149	25/18670	0.01683	0.028893	0.010573	LEP/SNAI2
GO:0032107	regulation of response to nutrient levels	2/149	25/18670	0.01683	0.028893	0.010573	LEP/SNAI2
GO:0035235	ionotropic glutamate receptor signaling pathway	2/149	25/18670	0.01683	0.028893	0.010573	GRIN2B/GRIN1
GO:0045830	positive regulation of isotype switching	2/149	25/18670	0.01683	0.028893	0.010573	TGFB1/IL2
GO:0050901	leukocyte tethering or rolling	2/149	25/18670	0.01683	0.028893	0.010573	LEP/TNF
GO:0050927	positive regulation of positive chemotaxis	2/149	25/18670	0.01683	0.028893	0.010573	CXCL8/KDR
GO:0051894	positive regulation of focal adhesion assembly	2/149	25/18670	0.01683	0.028893	0.010573	KDR/ABL1
GO:0060561	apoptotic process involved in morphogenesis	2/149	25/18670	0.01683	0.028893	0.010573	NOTCH1/BAX
GO:0061217	regulation of mesonephros development	2/149	25/18670	0.01683	0.028893	0.010573	SOX9/TGFB1
GO:0071378	cellular response to growth hormone stimulus	2/149	25/18670	0.01683	0.028893	0.010573	JAK3/PIK3R1
GO:0072202	cell differentiation involved in metanephros development	2/149	25/18670	0.01683	0.028893	0.010573	CTNNB1/STAT1
GO:1900101	regulation of endoplasmic reticulum unfolded protein response	2/149	25/18670	0.01683	0.028893	0.010573	PIK3R1/BAX

GO:1904861	excitatory synapse assembly	2/149	25/18670	0.01683	0.028893	0.010573	SHANK3/NRXN1
GO:1905809	negative regulation of synapse organization	2/149	25/18670	0.01683	0.028893	0.010573	APOE/GRIN2B
GO:0006839	mitochondrial transport	6/149	256/18670	0.016949	0.029086	0.010643	BCL2/CASP8/GSK3B/HSP90AA1/TP53/BAX
GO:0035914	skeletal muscle cell differentiation	3/149	68/18670	0.017109	0.029339	0.010736	FOS/RB1/NOTCH1
GO:0035924	cellular response to vascular endothelial growth factor stimulus	3/149	68/18670	0.017109	0.029339	0.010736	KDR/AKT1/NOTCH1
GO:0090150	establishment of protein localization to membrane	7/149	332/18670	0.017355	0.029748	0.010885	BCL2/CASP8/HSP90AA1/EGFR/ERBB2/TP53/BAX
GO:0007043	cell-cell junction assembly	4/149	124/18670	0.017481	0.029942	0.010956	TNF/GPBAR1/CAV1/SNAI2
GO:0019751	polyol metabolic process	4/149	124/18670	0.017481	0.029942	0.010956	LEP/SNCA/FGF2/CYP3A4
GO:1901292	nucleoside phosphate catabolic process	5/149	188/18670	0.017541	0.030032	0.010989	IGF1/INS/IFNG/TP53/HTR2A
GO:0008088	axo-dendritic transport	3/149	69/18670	0.017785	0.030439	0.011138	MAPT/FMR1/SOD1
GO:0030326	embryonic limb morphogenesis	4/149	125/18670	0.01795	0.030698	0.011233	CTNNB1/CACNA1C/NOTCH1/BAX
GO:0035113	embryonic appendage morphogenesis	4/149	125/18670	0.01795	0.030698	0.011233	CTNNB1/CACNA1C/NOTCH1/BAX
GO:0022011	myelination in peripheral nervous system	2/149	26/18670	0.018138	0.030867	0.011295	SOD1/AKT1
GO:0031645	negative regulation of neurological system process	2/149	26/18670	0.018138	0.030867	0.011295	IL10/AVP
GO:0031954	positive regulation of protein autophosphorylation	2/149	26/18670	0.018138	0.030867	0.011295	INS/SRC
GO:0032292	peripheral nervous system axon ensheathment	2/149	26/18670	0.018138	0.030867	0.011295	SOD1/AKT1

GO:0032967	positive regulation of collagen biosynthetic process	2/149	26/18670	0.018138	0.030867	0.011295	F2/TGFB1
GO:0036152	phosphatidylethanolamine acyl-chain remodeling	2/149	26/18670	0.018138	0.030867	0.011295	PLA2G2A/PLA2G6
GO:0042044	fluid transport	2/149	26/18670	0.018138	0.030867	0.011295	CFTR/AVP
GO:0043567	regulation of insulin-like growth factor receptor signaling pathway	2/149	26/18670	0.018138	0.030867	0.011295	IGF1/IGFBP3
GO:0050926	regulation of positive chemotaxis	2/149	26/18670	0.018138	0.030867	0.011295	CXCL8/KDR
GO:0060669	embryonic placenta morphogenesis	2/149	26/18670	0.018138	0.030867	0.011295	IL10/IGF2
GO:0060706	cell differentiation involved in embryonic placenta development	2/149	26/18670	0.018138	0.030867	0.011295	CASP8/AKT1
GO:0072539	T-helper 17 cell differentiation	2/149	26/18670	0.018138	0.030867	0.011295	IL6/IL2
GO:1900120	regulation of receptor binding	2/149	26/18670	0.018138	0.030867	0.011295	IL10/MMP9
GO:0008654	phospholipid biosynthetic process	6/149	260/18670	0.018148	0.030872	0.011297	PLA2G2A/PLA2G6/ACHE/FGF2/PIK3R1/HTR2A
GO:0034121	regulation of toll-like receptor signaling pathway	3/149	70/18670	0.018475	0.031415	0.011495	IRF1/ESR1/CAV1
GO:0042440	pigment metabolic process	3/149	71/18670	0.019179	0.032589	0.011925	BCL2/HMOX1/FECH
GO:0051057	positive regulation of small GTPase mediated signal transduction	3/149	71/18670	0.019179	0.032589	0.011925	IGF1/SRC/NOTCH1
GO:0045333	cellular respiration	5/149	193/18670	0.019412	0.032926	0.012048	NOS2/PRDM16/SNCA/IFNG/CAT
GO:0002026	regulation of the force of heart contraction	2/149	27/18670	0.019488	0.032926	0.012048	NOS1/CAV1

GO:0002825	regulation of T-helper 1 type immune response	2/149	27/18670	0.019488	0.032926	0.012048	JAK3/IL1B
GO:0010714	positive regulation of collagen metabolic process	2/149	27/18670	0.019488	0.032926	0.012048	F2/TGFB1
GO:0036151	phosphatidylcholine acyl-chain remodeling	2/149	27/18670	0.019488	0.032926	0.012048	PLA2G2A/PLA2G6
GO:0042501	serine phosphorylation of STAT protein	2/149	27/18670	0.019488	0.032926	0.012048	RET/IFNG
GO:0043032	positive regulation of macrophage activation	2/149	27/18670	0.019488	0.032926	0.012048	IL13/IL10
GO:0045671	negative regulation of osteoclast differentiation	2/149	27/18670	0.019488	0.032926	0.012048	CTNNB1/PIK3R1
GO:0045932	negative regulation of muscle contraction	2/149	27/18670	0.019488	0.032926	0.012048	PTGS2/SOD1
GO:0060259	regulation of feeding behavior	2/149	27/18670	0.019488	0.032926	0.012048	OPRK1/INS
GO:0060307	regulation of ventricular cardiac muscle cell membrane repolarization	2/149	27/18670	0.019488	0.032926	0.012048	KCNQ1/SCN5A
GO:0071514	genetic imprinting	2/149	27/18670	0.019488	0.032926	0.012048	KCNQ1/IGF2
GO:0072207	metanephric epithelium development	2/149	27/18670	0.019488	0.032926	0.012048	SOX9/STAT1
GO:0072378	blood coagulation, fibrin clot formation	2/149	27/18670	0.019488	0.032926	0.012048	F2/F12
GO:1904357	negative regulation of telomere maintenance via telomere lengthening	2/149	27/18670	0.019488	0.032926	0.012048	PARP1/SRC
GO:0006305	DNA alkylation	3/149	72/18670	0.019899	0.033527	0.012268	MYC/PARP1/FOS
GO:0006306	DNA methylation	3/149	72/18670	0.019899	0.033527	0.012268	MYC/PARP1/FOS
GO:0035019	somatic stem cell population maintenance	3/149	72/18670	0.019899	0.033527	0.012268	PRDM16/SOX9/FGF2

GO:0051155	positive regulation of striated muscle cell differentiation	3/149	72/18670	0.019899	0.033527	0.012268	IGF1/BCL2/TGFB1
GO:1902036	regulation of hematopoietic stem cell differentiation	3/149	72/18670	0.019899	0.033527	0.012268	NFE2L2/ABL1/TCF3
GO:0006639	acylglycerol metabolic process	4/149	129/18670	0.019904	0.033527	0.012268	APOE/CYP2E1/CAV1/CAT
GO:0034754	cellular hormone metabolic process	4/149	129/18670	0.019904	0.033527	0.012268	CYP1A1/ADH1C/ESR1/CYP3A4
GO:0050853	B cell receptor signaling pathway	4/149	129/18670	0.019904	0.033527	0.012268	BCL2/MAPK1/ABL1/BAX
GO:0072331	signal transduction by p53 class mediator	6/149	267/18670	0.020385	0.034313	0.012556	BCL2/TP53/CDKN1A/AKT1/SNAI2/BAX
GO:1901988	negative regulation of cell cycle phase transition	6/149	267/18670	0.020385	0.034313	0.012556	BCL2/CCND1/RB1/TP53/CDKN1A/BAX
GO:0060350	endochondral bone morphogenesis	3/149	73/18670	0.020633	0.034678	0.012689	COL1A1/SOX9/SERPINH1
GO:0072401	signal transduction involved in DNA integrity checkpoint	3/149	73/18670	0.020633	0.034678	0.012689	TP53/CDKN1A/BAX
GO:0072422	signal transduction involved in DNA damage checkpoint	3/149	73/18670	0.020633	0.034678	0.012689	TP53/CDKN1A/BAX
GO:1903779	regulation of cardiac conduction	3/149	73/18670	0.020633	0.034678	0.012689	NOS1/ABCC9/CAV1
GO:0001702	gastrulation with mouth forming second	2/149	28/18670	0.020879	0.034881	0.012764	CTNNB1/NODAL
GO:0001773	myeloid dendritic cell activation	2/149	28/18670	0.020879	0.034881	0.012764	TGFB1/IL10
GO:0006972	hyperosmotic response	2/149	28/18670	0.020879	0.034881	0.012764	SLC12A2/AVP
GO:0008209	androgen metabolic process	2/149	28/18670	0.020879	0.034881	0.012764	ESR1/CYP3A4
GO:0009065	glutamine family amino acid catabolic process	2/149	28/18670	0.020879	0.034881	0.012764	NOS2/NOS1

GO:0032647	regulation of interferon-alpha production	2/149	28/18670	0.020879	0.034881	0.012764	STAT1/IL10
GO:0033137	negative regulation of peptidyl-serine phosphorylation	2/149	28/18670	0.020879	0.034881	0.012764	CAV1/BAX
GO:0033688	regulation of osteoblast proliferation	2/149	28/18670	0.020879	0.034881	0.012764	BCL2/ABL1
GO:0034698	response to gonadotropin	2/149	28/18670	0.020879	0.034881	0.012764	ICAM1/NOTCH1
GO:0035116	embryonic hindlimb morphogenesis	2/149	28/18670	0.020879	0.034881	0.012764	CTNNB1/NOTCH1
GO:0035456	response to interferon-beta	2/149	28/18670	0.020879	0.034881	0.012764	IRF1/STAT1
GO:0042634	regulation of hair cycle	2/149	28/18670	0.020879	0.034881	0.012764	TNF/CTNNB1
GO:0045408	regulation of interleukin-6 biosynthetic process	2/149	28/18670	0.020879	0.034881	0.012764	TNF/IL1B
GO:0071168	protein localization to chromatin	2/149	28/18670	0.020879	0.034881	0.012764	ESR1/RB1
GO:1903792	negative regulation of anion transport	2/149	28/18670	0.020879	0.034881	0.012764	LEP/AKT1
GO:1904994	regulation of leukocyte adhesion to vascular endothelial cell	2/149	28/18670	0.020879	0.034881	0.012764	TNF/ICAM1
GO:0055076	transition metal ion homeostasis	4/149	131/18670	0.020928	0.03495	0.012789	MYC/S100A8/HMOX1/SOD1
GO:0009064	glutamine family amino acid metabolic process	3/149	74/18670	0.021382	0.035669	0.013052	NOS2/NOS1/GGT1
GO:0072395	signal transduction involved in cell cycle checkpoint	3/149	74/18670	0.021382	0.035669	0.013052	TP53/CDKN1A/BAX
GO:1901616	organic hydroxy compound catabolic process	3/149	74/18670	0.021382	0.035669	0.013052	APOE/MAOB/CYP24A1
GO:0016052	carbohydrate catabolic process	5/149	199/18670	0.021826	0.036396	0.013318	IGF1/INS/IFNG/TP53/HTR2A

GO:0001960	negative regulation of cytokine-mediated signaling pathway	3/149	75/18670	0.022146	0.036846	0.013482	PPARG/IL6/CAV1
GO:0007422	peripheral nervous system development	3/149	75/18670	0.022146	0.036846	0.013482	SOD1/ERBB2/AKT1
GO:0010611	regulation of cardiac muscle hypertrophy	3/149	75/18670	0.022146	0.036846	0.013482	IGF1/PARP1/NOTCH1
GO:0021536	diencephalon development	3/149	75/18670	0.022146	0.036846	0.013482	DRD2/NKX2-1/BAX
GO:0006929	substrate-dependent cell migration	2/149	29/18670	0.02231	0.036846	0.013482	P2RY12/SNAI2
GO:0008299	isoprenoid biosynthetic process	2/149	29/18670	0.02231	0.036846	0.013482	CYP1A1/HMGCR
GO:0010644	cell communication by electrical coupling	2/149	29/18670	0.02231	0.036846	0.013482	CACNA1C/CAV1
GO:0010800	positive regulation of peptidyl-threonine phosphorylation	2/149	29/18670	0.02231	0.036846	0.013482	TGFB1/MAPK1
GO:0014044	Schwann cell development	2/149	29/18670	0.02231	0.036846	0.013482	SOD1/AKT1
GO:0021799	cerebral cortex radially oriented cell migration	2/149	29/18670	0.02231	0.036846	0.013482	CTNNB1/P2RY12
GO:0031063	regulation of histone deacetylation	2/149	29/18670	0.02231	0.036846	0.013482	TGFB1/TP53
GO:0031116	positive regulation of microtubule polymerization	2/149	29/18670	0.02231	0.036846	0.013482	MET/MAPT
GO:0032232	negative regulation of actin filament bundle assembly	2/149	29/18670	0.02231	0.036846	0.013482	MET/SHANK3
GO:0033032	regulation of myeloid cell apoptotic process	2/149	29/18670	0.02231	0.036846	0.013482	BCL2/THRA
GO:0042226	interleukin-6 biosynthetic process	2/149	29/18670	0.02231	0.036846	0.013482	TNF/IL1B
GO:0044030	regulation of DNA methylation	2/149	29/18670	0.02231	0.036846	0.013482	MYC/PARP1

GO:0044788	modulation by host of viral process	2/149	29/18670	0.02231	0.036846	0.013482	APOE/FMR1
GO:0045822	negative regulation of heart contraction	2/149	29/18670	0.02231	0.036846	0.013482	TRPV1/IL2
GO:0050974	detection of mechanical stimulus involved in sensory perception	2/149	29/18670	0.02231	0.036846	0.013482	TRPA1/HTR2A
GO:0055075	potassium ion homeostasis	2/149	29/18670	0.02231	0.036846	0.013482	SLC12A2/SLC12A1
GO:0070102	interleukin-6-mediated signaling pathway	2/149	29/18670	0.02231	0.036846	0.013482	STAT1/IL6
GO:0070229	negative regulation of lymphocyte apoptotic process	2/149	29/18670	0.02231	0.036846	0.013482	IL2/JAK3
GO:0070528	protein kinase C signaling	2/149	29/18670	0.02231	0.036846	0.013482	PLA2G6/AVP
GO:0072538	T-helper 17 type immune response	2/149	29/18670	0.02231	0.036846	0.013482	IL6/IL2
GO:2000515	negative regulation of CD4-positive, alpha-beta T cell activation	2/149	29/18670	0.02231	0.036846	0.013482	IL2/JAK3
GO:2000727	positive regulation of cardiac muscle cell differentiation	2/149	29/18670	0.02231	0.036846	0.013482	IGF1/TGFB1
GO:0032273	positive regulation of protein polymerization	4/149	134/18670	0.022524	0.037185	0.013607	MET/MAPT/HSP90AA1/ICAM1
GO:0048844	artery morphogenesis	3/149	76/18670	0.022924	0.037833	0.013844	APOE/MYLK/NOTCH1
GO:0031397	negative regulation of protein ubiquitination	3/149	77/18670	0.023717	0.039043	0.014287	CAV1/ABL1/AKT1
GO:0035050	embryonic heart tube development	3/149	77/18670	0.023717	0.039043	0.014287	CTNNB1/NODAL/NOTCH1
GO:0061418	regulation of transcription from RNA polymerase II promoter in response to hypoxia	3/149	77/18670	0.023717	0.039043	0.014287	NFE2L2/TP53/NOTCH1

GO:1901224	positive regulation of NIK/NF-kappaB signaling	3/149	77/18670	0.023717	0.039043	0.014287	TNF/EGFR/IL1B
GO:0003413	chondrocyte differentiation involved in endochondral bone morphogenesis	2/149	30/18670	0.02378	0.039043	0.014287	SOX9/SERPINH1
GO:0032607	interferon-alpha production	2/149	30/18670	0.02378	0.039043	0.014287	STAT1/IL10
GO:0046320	regulation of fatty acid oxidation	2/149	30/18670	0.02378	0.039043	0.014287	PPARG/AKT1
GO:0050869	negative regulation of B cell activation	2/149	30/18670	0.02378	0.039043	0.014287	CASP3/IL10
GO:0061082	myeloid leukocyte cytokine production	2/149	30/18670	0.02378	0.039043	0.014287	TGFB1/HMOX1
GO:0071480	cellular response to gamma radiation	2/149	30/18670	0.02378	0.039043	0.014287	TP53/CDKN1A
GO:0099068	postsynapse assembly	2/149	30/18670	0.02378	0.039043	0.014287	SHANK3/NRXN1
GO:1904292	regulation of ERAD pathway	2/149	30/18670	0.02378	0.039043	0.014287	NFE2L2/CAV1
GO:1904837	beta-catenin-TCF complex assembly	2/149	30/18670	0.02378	0.039043	0.014287	CTNNB1/MYC
GO:1905606	regulation of presynapse assembly	2/149	30/18670	0.02378	0.039043	0.014287	SNCA/NRXN1
GO:0031396	regulation of protein ubiquitination	5/149	204/18670	0.023981	0.039359	0.014402	AVPR2/HSP90AA1/CAV1/ABL1/AKT1
GO:0001910	regulation of leukocyte mediated cytotoxicity	3/149	78/18670	0.024525	0.040194	0.014708	NOS2/LEP/ICAM1
GO:0014743	regulation of muscle hypertrophy	3/149	78/18670	0.024525	0.040194	0.014708	IGF1/PARP1/NOTCH1
GO:0042246	tissue regeneration	3/149	78/18670	0.024525	0.040194	0.014708	IGF1/CDKN1A/NOTCH1
GO:0090398	cellular senescence	3/149	78/18670	0.024525	0.040194	0.014708	ABL1/TP53/CDKN1A

GO:0030048	actin filament-based movement	4/149	138/18670	0.024764	0.040571	0.014845	KCNQ1/CACNA1C/CAV1/SCN5A
GO:0002724	regulation of T cell cytokine production	2/149	31/18670	0.025289	0.041189	0.015072	IL6/IL1B
GO:0009954	proximal/distal pattern formation	2/149	31/18670	0.025289	0.041189	0.015072	CTNNB1/NODAL
GO:0010543	regulation of platelet activation	2/149	31/18670	0.025289	0.041189	0.015072	APOE/F2
GO:0033687	osteoblast proliferation	2/149	31/18670	0.025289	0.041189	0.015072	BCL2/ABL1
GO:0034694	response to prostaglandin	2/149	31/18670	0.025289	0.041189	0.015072	PPARG/AKT1
GO:0042168	heme metabolic process	2/149	31/18670	0.025289	0.041189	0.015072	HMOX1/FECH
GO:0043304	regulation of mast cell degranulation	2/149	31/18670	0.025289	0.041189	0.015072	IL13/HMOX1
GO:0045648	positive regulation of erythrocyte differentiation	2/149	31/18670	0.025289	0.041189	0.015072	STAT1/ACVR1B
GO:0046471	phosphatidylglycerol metabolic process	2/149	31/18670	0.025289	0.041189	0.015072	PLA2G2A/PLA2G6
GO:0086011	membrane repolarization during action potential	2/149	31/18670	0.025289	0.041189	0.015072	KCNQ1/CAV1
GO:0097106	postsynaptic density organization	2/149	31/18670	0.025289	0.041189	0.015072	SHANK3/NRXN1
GO:0099174	regulation of presynapse organization	2/149	31/18670	0.025289	0.041189	0.015072	SNCA/NRXN1
GO:0099625	ventricular cardiac muscle cell membrane repolarization	2/149	31/18670	0.025289	0.041189	0.015072	KCNQ1/SCN5A
GO:1902230	negative regulation of intrinsic apoptotic signaling pathway in response to DNA damage	2/149	31/18670	0.025289	0.041189	0.015072	BCL2/SNAI2
GO:1902253	regulation of intrinsic apoptotic signaling	2/149	31/18670	0.025289	0.041189	0.015072	BCL2/TP53

	pathway by p53 class mediator						
GO:1903393	positive regulation of adherens junction organization	2/149	31/18670	0.025289	0.041189	0.015072	KDR/ABL1
GO:0003151	outflow tract morphogenesis	3/149	79/18670	0.025348	0.041255	0.015096	CTNNB1/JUN/NOTCH1
GO:0055072	iron ion homeostasis	3/149	79/18670	0.025348	0.041255	0.015096	MYC/HMOX1/SOD1
GO:0051258	protein polymerization	6/149	283/18670	0.026186	0.042604	0.01559	MET/MAPT/SNCA/HSP90AA1/ICAM1/ABL1
GO:0097529	myeloid leukocyte migration	5/149	210/18670	0.026742	0.043471	0.015907	CXCL8/P2RY12/S100A8/IL6/IL1B
GO:0001569	branching involved in blood vessel morphogenesis	2/149	32/18670	0.026836	0.043471	0.015907	CTNNB1/ABL1
GO:0008210	estrogen metabolic process	2/149	32/18670	0.026836	0.043471	0.015907	CYP1A1/CYP3A4
GO:0033006	regulation of mast cell activation involved in immune response	2/149	32/18670	0.026836	0.043471	0.015907	IL13/HMOX1
GO:0039694	viral RNA genome replication	2/149	32/18670	0.026836	0.043471	0.015907	CXCL8/FMR1
GO:0042491	inner ear auditory receptor cell differentiation	2/149	32/18670	0.026836	0.043471	0.015907	SOD1/NOTCH1
GO:0045922	negative regulation of fatty acid metabolic process	2/149	32/18670	0.026836	0.043471	0.015907	INS/AKT1
GO:0045987	positive regulation of smooth muscle contraction	2/149	32/18670	0.026836	0.043471	0.015907	PTGS2/CHRM3
GO:0060317	cardiac epithelial to mesenchymal transition	2/149	32/18670	0.026836	0.043471	0.015907	SNAI2/NOTCH1
GO:0060674	placenta blood vessel development	2/149	32/18670	0.026836	0.043471	0.015907	MAPK1/AKT1

GO:0099623	regulation of cardiac muscle cell membrane repolarization	2/149	32/18670	0.026836	0.043471	0.015907	KCNQ1/SCN5A
GO:2000778	positive regulation of interleukin-6 secretion	2/149	32/18670	0.026836	0.043471	0.015907	TNF/IL1B
GO:0014855	striated muscle cell proliferation	3/149	81/18670	0.027038	0.043767	0.016015	MAPK1/FGF2/NOTCH1
GO:0060761	negative regulation of response to cytokine stimulus	3/149	81/18670	0.027038	0.043767	0.016015	PPARG/IL6/CAV1
GO:1902904	negative regulation of supramolecular fiber organization	4/149	142/18670	0.027135	0.043909	0.016067	MET/APOE/SNCA/SHANK3
GO:0048813	dendrite morphogenesis	4/149	143/18670	0.027749	0.044885	0.016424	SHANK3/GSK3B/FMR1/GRIN1
GO:0060415	muscle tissue morphogenesis	3/149	82/18670	0.027905	0.045121	0.016511	TGFB1/MYLK/NOTCH1
GO:0048562	embryonic organ morphogenesis	6/149	288/18670	0.028203	0.045587	0.016681	SOX9/CTNNB1/MAPK1/NODAL/SOD1/NOTCH1
GO:0006471	protein ADP-ribosylation	2/149	33/18670	0.02842	0.045739	0.016737	PARP1/IFNG
GO:0031112	positive regulation of microtubule polymerization or depolymerization	2/149	33/18670	0.02842	0.045739	0.016737	MET/MAPT
GO:0035115	embryonic forelimb morphogenesis	2/149	33/18670	0.02842	0.045739	0.016737	CTNNB1/CACNA1C
GO:0035774	positive regulation of insulin secretion involved in cellular response to glucose stimulus	2/149	33/18670	0.02842	0.045739	0.016737	PLA2G6/CFTR
GO:0042755	eating behavior	2/149	33/18670	0.02842	0.045739	0.016737	LEP/OPRK1
GO:0045736	negative regulation of cyclin-dependent protein	2/149	33/18670	0.02842	0.045739	0.016737	CASP3/CDKN1A

	serine/threonine kinase activity						
GO:0048011	neurotrophin TRK receptor signaling pathway	2/149	33/18670	0.02842	0.045739	0.016737	CASP3/SRC
GO:0048536	spleen development	2/149	33/18670	0.02842	0.045739	0.016737	BCL2/ABL1
GO:0048854	brain morphogenesis	2/149	33/18670	0.02842	0.045739	0.016737	SLC6A4/SHANK3
GO:0070232	regulation of T cell apoptotic process	2/149	33/18670	0.02842	0.045739	0.016737	TP53/JAK3
GO:0099084	postsynaptic specialization organization	2/149	33/18670	0.02842	0.045739	0.016737	SHANK3/NRXN1
GO:1901797	negative regulation of signal transduction by p53 class mediator	2/149	33/18670	0.02842	0.045739	0.016737	BCL2/SNAI2
GO:0008652	cellular amino acid biosynthetic process	3/149	83/18670	0.028786	0.046279	0.016934	GGT1/GATM/PAH
GO:0045582	positive regulation of T cell differentiation	3/149	83/18670	0.028786	0.046279	0.016934	TGFB1/IL2/IFNG
GO:0071277	cellular response to calcium ion	3/149	83/18670	0.028786	0.046279	0.016934	FOS/JUN/SCN5A
GO:0000077	DNA damage checkpoint	4/149	145/18670	0.029	0.046606	0.017054	CCND1/TP53/CDKN1A/BAX
GO:0051650	establishment of vesicle localization	6/149	290/18670	0.029038	0.04665	0.01707	DRD2/CTNNB1/SNCA/GSK3B/FMR1/HTR2A
GO:0019935	cyclic-nucleotide-mediated signaling	5/149	215/18670	0.029192	0.046882	0.017155	NOS1/APOE/ADRA2A/SOX9/DRD2
GO:0051492	regulation of stress fiber assembly	3/149	84/18670	0.029683	0.047652	0.017437	MET/ABL1/PIK3R1
GO:0032212	positive regulation of telomere maintenance via telomerase	2/149	34/18670	0.030041	0.048054	0.017584	CTNNB1/MAPK1
GO:0042533	tumor necrosis factor biosynthetic process	2/149	34/18670	0.030041	0.048054	0.017584	IL10/IFNG

GO:0042534	regulation of tumor necrosis factor biosynthetic process	2/149	34/18670	0.030041	0.048054	0.017584	IL10/IFNG
GO:0043368	positive T cell selection	2/149	34/18670	0.030041	0.048054	0.017584	BCL2/IL6
GO:0043516	regulation of DNA damage response, signal transduction by p53 class mediator	2/149	34/18670	0.030041	0.048054	0.017584	TP53/SNAI2
GO:0048566	embryonic digestive tract development	2/149	34/18670	0.030041	0.048054	0.017584	CXCL8/TNF
GO:0060795	cell fate commitment involved in formation of primary germ layer	2/149	34/18670	0.030041	0.048054	0.017584	CTNNB1/NODAL
GO:0070050	neuron cellular homeostasis	2/149	34/18670	0.030041	0.048054	0.017584	SLC6A2/IL6
GO:0098901	regulation of cardiac muscle cell action potential	2/149	34/18670	0.030041	0.048054	0.017584	CACNA1C/CAV1
GO:1904030	negative regulation of cyclin-dependent protein kinase activity	2/149	34/18670	0.030041	0.048054	0.017584	CASP3/CDKN1A
GO:0009798	axis specification	3/149	85/18670	0.030594	0.048904	0.017895	CTNNB1/NODAL/NOTCH1
GO:0045682	regulation of epidermis development	3/149	85/18670	0.030594	0.048904	0.017895	TNF/CTNNB1/NOTCH1
GO:0016064	immunoglobulin mediated immune response	5/149	218/18670	0.030729	0.049102	0.017967	CRP/TNF/TGFB1/IL10/IL2
GO:0016236	macroautophagy	6/149	295/18670	0.031196	0.049831	0.018234	CASP3/KDR/HMOX1/SRC/TP53/AKT1

Supplementary Table S6-2: Datailed information of Cellular components(CC) enrichment of common targets

ID	Description	GeneRatio	BgRatio	pvalue	p.adjust	qvalue	geneID	Count
GO:0045121	membrane raft	26/149	315/19717	9.24E-20	1.71E-17	1.08E-17	SLC6A2/SLC6A4/NOS1/DPP4/FAS/CASP8/RET/MAPT/TNF/PTGS2/CTNNB1/P2RY12/CTSD/KCNQ1/CASP3/FASLG/KDR/HMOX1/SERPINH1/MAPK1/EGFR/ICAM1/CAV1/SRC/HTR2A/SCN5A	26

GO:0098857	membrane microdomain	26/149	316/19717	1.00E-19	1.71E-17	1.08E-17	SLC6A2/SLC6A4/NOS1/DPP4/FAS/CASP8/RET/MAPT/TNF/PTGS2/CTNNB1/P2RY12/CTSD/KCNQ1/CASP3/FASLG/KDR/HMOX1/SERPINH1/MAPK1/EGFR/ICAM1/CAV1/SRC/HTR2A/SCN5A	26
GO:0098589	membrane region	26/149	328/19717	2.55E-19	2.91E-17	1.83E-17	SLC6A2/SLC6A4/NOS1/DPP4/FAS/CASP8/RET/MAPT/TNF/PTGS2/CTNNB1/P2RY12/CTSD/KCNQ1/CASP3/FASLG/KDR/HMOX1/SERPINH1/MAPK1/EGFR/ICAM1/CAV1/SRC/HTR2A/SCN5A	26
GO:0005901	caveola	11/149	80/19717	2.12E-11	1.71E-09	1.08E-09	NOS1/PTGS2/CTNNB1/P2RY12/FASLG/HMOX1/MAPK1/CAV1/SRC/HTR2A/SCN5A	11
GO:0043025	neuronal cell body	22/149	497/19717	2.50E-11	1.71E-09	1.08E-09	SLC6A2/APOE/HTR3A/OPRK1/CACNA1B/RET/MAPT/DRD2/SNCA/TGFB1/NRXN1/TRPV1/HSP90AA1/CASP3/CACNA1C/FMR1/MAPK1/NQO1/SOD1/CACNA1A/ABL1/HTR2A	22
GO:0034702	ion channel complex	17/149	301/19717	1.32E-10	7.54E-09	4.76E-09	NOS1/HTR3A/CACNA1B/GRIN2B/GABRG3/SHANK3/KCNQ1/ABCC9/CACNA1C/CACNA1A/GRIN1/GABRD/CACNA1S/CFTR/SCN10A/GABRA3/SCN5A	17
GO:1902495	transmembrane transporter complex	17/149	324/19717	4.13E-10	2.02E-08	1.27E-08	NOS1/HTR3A/CACNA1B/GRIN2B/GABRG3/SHANK3/KCNQ1/ABCC9/CACNA1C/CACNA1A/GRIN1/GABRD/CACNA1S/CFTR/SCN10A/GABRA3/SCN5A	17
GO:1990351	transporter complex	17/149	332/19717	6.00E-10	2.43E-08	1.53E-08	NOS1/HTR3A/CACNA1B/GRIN2B/GABRG3/SHANK3/KCNQ1/ABCC9/CACNA1C/CACNA1A/GRIN1/GABRD/CACNA1S/CFTR/SCN10A/GABRA3/SCN5A	17
GO:0044853	plasma membrane raft	11/149	109/19717	6.44E-10	2.43E-08	1.53E-08	NOS1/PTGS2/CTNNB1/P2RY12/FASLG/HMOX1/MAPK1/CAV1/SRC/HTR2A/SCN5A	11
GO:0097060	synaptic membrane	19/149	432/19717	7.10E-10	2.43E-08	1.53E-08	SLC6A2/SLC6A4/HTR3A/OPRK1/DRD2/GRIN2B/CTNNB1/GABRG3/SHANK3/NRXN1/TRPV1/CACNA1C/FMR1/GRIN1/GABRD/CHRM3/HTR2A/SCN10A/GABRA3	19
GO:0099699	integral component of synaptic membrane	12/149	152/19717	1.82E-09	5.67E-08	3.58E-08	SLC6A4/HTR3A/OPRK1/DRD2/GABRG3/NRXN1/GRIN1/GABRD/CHRM3/HTR2A/SCN10A/GABRA3	12
GO:0045211	postsynaptic membrane	16/149	323/19717	3.19E-09	9.08E-08	5.73E-08	SLC6A4/HTR3A/OPRK1/DRD2/GRIN2B/CTNNB1/GABRG3/SHANK3/TRPV1/CACNA1C/FMR1/GRIN1/GABRD/CHRM3/HTR2A/GABRA3	16
GO:0099240	intrinsic component of synaptic membrane	12/149	164/19717	4.34E-09	1.14E-07	7.21E-08	SLC6A4/HTR3A/OPRK1/DRD2/GABRG3/NRXN1/GRIN1/GABRD/CHRM3/HTR2A/SCN10A/GABRA3	12
GO:0042734	presynaptic membrane	11/149	161/19717	4.00E-08	9.76E-07	6.16E-07	SLC6A2/SLC6A4/HTR3A/OPRK1/DRD2/CTNNB1/NRXN1/FMR1/CHRM3/HTR2A/SCN10A	11
GO:0031983	vesicle lumen	15/149	339/19717	4.55E-08	1.04E-06	6.55E-07	IGF1/APOE/MPO/CTSD/TGFB1/S100A8/HSP90AA1/FASLG/INS/MAPK1/IGF2/EGFR/CAT/NFKB1/SPARC	15
GO:0099056	integral component of presynaptic membrane	8/149	73/19717	7.82E-08	1.67E-06	1.05E-06	SLC6A4/HTR3A/OPRK1/DRD2/NRXN1/CHRM3/HTR2A/SCN10A	8
GO:0034703	cation channel complex	12/149	220/19717	1.14E-07	2.30E-06	1.45E-06	NOS1/HTR3A/CACNA1B/GRIN2B/KCNQ1/ABCC9/CACNA1C/CACNA1A/GRIN1/CACNA1S/SCN10A/SCN5A	12
GO:0098889	intrinsic component of presynaptic membrane	8/149	82/19717	1.96E-07	3.72E-06	2.35E-06	SLC6A4/HTR3A/OPRK1/DRD2/NRXN1/CHRM3/HTR2A/SCN10A	8
GO:0099055	integral component of postsynaptic membrane	9/149	117/19717	2.60E-07	4.68E-06	2.96E-06	SLC6A4/HTR3A/OPRK1/DRD2/GRIN1/GABRD/CHRM3/HTR2A/GABRA3	9

GO:0060205	cytoplasmic vesicle lumen	14/149	338/19717	2.92E-07	5.00E-06	3.16E-06	IGF1/APOE/MPO/CTSD/TGFB1/S100A8/HSP90AA1/FASLG/INS/MAPK1/IGF2/CAT/NFKB1/SPARC	14
GO:0098936	intrinsic component of postsynaptic membrane	9/149	122/19717	3.72E-07	6.06E-06	3.82E-06	SLC6A4/HTR3A/OPRK1/DRD2/GRIN1/GABRD/CHRM3/HTR2A/GABRA3	9
GO:0098978	glutamatergic synapse	13/149	349/19717	2.57E-06	4.00E-05	2.52E-05	APOE/HTR3A/DRD2/NRXN1/GSK3B/GRIN1/GABRD/DRD4/SRC/CHRM3/SPARC/HTR2A/SCN10A	13
GO:0044798	nuclear transcription factor complex	10/149	201/19717	3.10E-06	4.60E-05	2.90E-05	THRB/PPARG/SOX9/CTNNB1/FOS/RB1/THRA/TCF3/TP53/JUN	10
GO:0090575	RNA polymerase II transcription factor complex	9/149	163/19717	4.20E-06	5.98E-05	3.77E-05	THRB/PPARG/CTNNB1/FOS/RB1/THRA/TCF3/TP53/JUN	9
GO:0098793	presynapse	15/149	491/19717	4.88E-06	6.68E-05	4.21E-05	SLC6A2/SLC6A4/NOS1/HTR3A/OPRK1/CACNA1B/DRD2/CTNNB1/SNCA/NRXN1/FMR1/GRIN1/CHRM3/HTR2A/SCN10A	15
GO:0034774	secretory granule lumen	12/149	321/19717	6.12E-06	8.05E-05	5.08E-05	IGF1/MPO/CTSD/TGFB1/S100A8/HSP90AA1/INS/MAPK1/IGF2/CAT/NFKB1/SPARC	12
GO:0062023	collagen-containing extracellular matrix	13/149	406/19717	1.31E-05	0.000163	0.000103	MMP2/COL1A1/APOE/F2/CTSD/TGFB1/S100A8/ACHE/SERPINH1/ICAM1/F12/SPARC/MMP9	13
GO:0099572	postsynaptic specialization	12/149	348/19717	1.38E-05	0.000163	0.000103	NOS1/DRD2/GRIN2B/CTNNB1/SHANK3/CACNA1C/FMR1/MAPK1/GRIN1/SRC/CHRM3/GABRA3	12
GO:0071682	endocytic vesicle lumen	4/149	20/19717	1.38E-05	0.000163	0.000103	APOE/MPO/HSP90AA1/SPARC	4
GO:0005891	voltage-gated calcium channel complex	5/149	43/19717	1.76E-05	0.000201	0.000127	NOS1/CACNA1B/CACNA1C/CACNA1A/CACNA1S	5
GO:0005667	transcription factor complex	12/149	365/19717	2.21E-05	0.000244	0.000154	THRB/PPARG/SOX9/CTNNB1/NKX2-1/PARP1/FOS/RB1/THRA/TCF3/TP53/JUN	12
GO:0043679	axon terminus	7/149	119/19717	3.38E-05	0.000361	0.000228	NOS1/OPRK1/DRD2/SNCA/FMR1/GRIN1/CHRM3	7
GO:0014069	postsynaptic density	11/149	324/19717	3.67E-05	0.00038	0.00024	NOS1/DRD2/GRIN2B/CTNNB1/SHANK3/CACNA1C/FMR1/MAPK1/GRIN1/SRC/CHRM3	11
GO:0032279	asymmetric synapse	11/149	328/19717	4.10E-05	0.000412	0.00026	NOS1/DRD2/GRIN2B/CTNNB1/SHANK3/CACNA1C/FMR1/MAPK1/GRIN1/SRC/CHRM3	11
GO:0098984	neuron to neuron synapse	11/149	350/19717	7.34E-05	0.000718	0.000453	NOS1/DRD2/GRIN2B/CTNNB1/SHANK3/CACNA1C/FMR1/MAPK1/GRIN1/SRC/CHRM3	11
GO:0044306	neuron projection terminus	7/149	138/19717	8.67E-05	0.000824	0.00052	NOS1/OPRK1/DRD2/SNCA/FMR1/GRIN1/CHRM3	7
GO:0009925	basal plasma membrane	4/149	34/19717	0.000122	0.001125	0.00071	MET/P2RY12/EGFR/ERBB2	4

GO:0000790	nuclear chromatin	11/149	377/19717	0.000141	0.001238	0.000781	IRF1/THRB/CTNNB1/ESR1/MYC/STAT1/RB1/TCF3/TP53/JUN/SNAI2	11
GO:0034704	calcium channel complex	5/149	66/19717	0.000142	0.001238	0.000781	NOS1/CACNA1B/CACNA1C/CACNA1A/CACNA1S	5
GO:0043083	synaptic cleft	3/149	14/19717	0.000145	0.001238	0.000781	APOE/ACHE/GRIN1	3
GO:0098982	GABA-ergic synapse	5/149	71/19717	0.000201	0.001675	0.001057	DRD2/GABRG3/NRXN1/GABRD/GABRA3	5
GO:0016323	basolateral plasma membrane	8/149	217/19717	0.00025	0.002033	0.001283	MET/ADRA2A/CTNNB1/P2RY12/KCNQ1/EGFR/ERBB2/CHRM3	8
GO:0031253	cell projection membrane	10/149	345/19717	0.000301	0.002359	0.001489	DPP4/MAPT/DRD2/CTNNB1/P2RY12/GABRG3/SHANK3/TRPV1/SRC/GABRA3	10
GO:0043197	dendritic spine	7/149	169/19717	0.000304	0.002359	0.001489	NOS1/MAPT/DRD2/SHANK3/TRPV1/FMR1/GRIN1	7
GO:0150034	distal axon	9/149	285/19717	0.000325	0.002424	0.001529	NOS1/OPRK1/MAPT/DRD2/SNCA/HSP90AA1/FMR1/GRIN1/CHRM3	9
GO:0044309	neuron spine	7/149	171/19717	0.000326	0.002424	0.001529	NOS1/MAPT/DRD2/SHANK3/TRPV1/FMR1/GRIN1	7
GO:1902711	GABA-A receptor complex	3/149	19/19717	0.000375	0.002729	0.001722	GABRG3/GABRD/GABRA3	3
GO:0005741	mitochondrial outer membrane	7/149	178/19717	0.000415	0.002959	0.001867	BCL2/CASP8/SNCA/MAOB/PGR/BAX/CYP24A1	7
GO:1902710	GABA receptor complex	3/149	20/19717	0.000439	0.003062	0.001932	GABRG3/GABRD/GABRA3	3
GO:0031970	organelle envelope lumen	5/149	88/19717	0.000545	0.003692	0.00233	BCHE/SNCA/SOD1/CAT/GATM	5
GO:0034707	chloride channel complex	4/149	50/19717	0.000551	0.003692	0.00233	GABRG3/GABRD/CFTR/GABRA3	4
GO:0005788	endoplasmic reticulum lumen	9/149	309/19717	0.000583	0.003762	0.002374	COL1A1/APOE/BCHE/F2/PTGS2/IL6/INS/SERPINH1/IGFBP3	9
GO:0042383	sarcolemma	6/149	136/19717	0.000591	0.003762	0.002374	NOS1/ABCC9/CACNA1C/CAV1/CACNA1S/SCN5A	6
GO:0045178	basal part of cell	4/149	51/19717	0.000594	0.003762	0.002374	MET/P2RY12/EGFR/ERBB2	4
GO:0031091	platelet alpha granule	5/149	91/19717	0.000636	0.003907	0.002465	IGF1/SNCA/TGFB1/IGF2/SPARC	5

GO:0030315	T-tubule	4/149	52/19717	0.00064	0.003907	0.002465	NOS1/CACNA1C/CACNA1S/SCN5A	4
GO:0033267	axon part	10/149	382/19717	0.000666	0.003995	0.002521	NOS1/OPRK1/MAPT/DRD2/SNCA/HSP90AA1/FMR1/SOD1/GRIN1/CHRM3	10
GO:0098878	neurotransmitter receptor complex	4/149	53/19717	0.000688	0.004018	0.002535	HTR3A/GRIN2B/SHANK3/GRIN1	4
GO:0045177	apical part of cell	10/149	384/19717	0.000693	0.004018	0.002535	DPP4/DUOX2/SLC12A2/ABCB1/CTNNB1/EGFR/ERBB2/SLC12A1/CFTR/NOTCH1	10
GO:0016324	apical plasma membrane	9/149	318/19717	0.000715	0.004075	0.002571	DPP4/DUOX2/SLC12A2/ABCB1/EGFR/ERBB2/SLC12A1/CFTR/NOTCH1	9
GO:0031968	organelle outer membrane	7/149	201/19717	0.000854	0.004787	0.00302	BCL2/CASP8/SNCA/MAOB/PGR/BAX/CYP24A1	7
GO:0019867	outer membrane	7/149	203/19717	0.000905	0.004921	0.003105	BCL2/CASP8/SNCA/MAOB/PGR/BAX/CYP24A1	7
GO:0032589	neuron projection membrane	4/149	57/19717	0.000907	0.004921	0.003105	MAPT/GABRG3/TRPV1/GABRA3	4
GO:0030017	sarcomere	7/149	204/19717	0.000931	0.004975	0.003139	MMP2/NOS1/CTNNB1/ABCC9/CACNA1C/CACNA1S/SCN5A	7
GO:0031252	cell leading edge	10/149	403/19717	0.001001	0.005265	0.003322	DPP4/DUOX2/MAPT/CTNNB1/GABRG3/TRPV1/MYLK/ABL1/SRC/GABRA3	10
GO:0044449	contractile fiber part	7/149	221/19717	0.001478	0.007661	0.004834	MMP2/NOS1/CTNNB1/ABCC9/CACNA1C/CACNA1S/SCN5A	7
GO:0030016	myofibril	7/149	224/19717	0.001597	0.00815	0.005143	MMP2/NOS1/CTNNB1/ABCC9/CACNA1C/CACNA1S/SCN5A	7
GO:0031093	platelet alpha granule lumen	4/149	67/19717	0.001659	0.008346	0.005266	IGF1/TGFB1/IGF2/SPARC	4
GO:0030665	clathrin-coated vesicle membrane	5/149	115/19717	0.001816	0.009	0.005679	APOE/AVPR2/EGFR/CFTR/AVP	5
GO:0031256	leading edge membrane	6/149	170/19717	0.001871	0.009139	0.005766	DPP4/MAPT/GABRG3/TRPV1/SRC/GABRA3	6
GO:0043292	contractile fiber	7/149	234/19717	0.002045	0.009852	0.006216	MMP2/NOS1/CTNNB1/ABCC9/CACNA1C/CACNA1S/SCN5A	7
GO:0030139	endocytic vesicle	8/149	303/19717	0.002178	0.010344	0.006527	DPP4/APOE/DRD2/MPO/HSP90AA1/EGFR/CAV1/SPARC	8
GO:1904813	ficolin-1-rich granule lumen	5/149	124/19717	0.002524	0.011823	0.00746	CTSD/HSP90AA1/MAPK1/CAT/MMP9	5

GO:0017146	NMDA selective glutamate receptor complex	2/149	11/19717	0.002983	0.013788	0.0087	GRIN2B/GRIN1	2
GO:0005758	mitochondrial intermembrane space	4/149	79/19717	0.003034	0.013834	0.008729	SNCA/SOD1/CAT/GATM	4
GO:0032590	dendrite membrane	3/149	40/19717	0.003405	0.015229	0.009609	GABRG3/TRPV1/GABRA3	3
GO:0043204	perikaryon	5/149	134/19717	0.003525	0.015229	0.009609	OPRK1/DRD2/CACNA1C/FMR1/MAPK1	5
GO:0030877	beta-catenin destruction complex	2/149	12/19717	0.003562	0.015229	0.009609	CTNNB1/GSK3B	2
GO:1990454	L-type voltage-gated calcium channel complex	2/149	12/19717	0.003562	0.015229	0.009609	CACNA1C/CACNA1S	2
GO:1990909	Wnt signalosome	2/149	12/19717	0.003562	0.015229	0.009609	CTNNB1/GSK3B	2
GO:0005925	focal adhesion	9/149	405/19717	0.003723	0.015719	0.009918	PLAU/SLC6A4/DPP4/CTNNB1/MAPK1/EGFR/ICAM1/CAV1/CAT	9
GO:0120111	neuron projection cytoplasm	4/149	84/19717	0.003785	0.015785	0.009959	MAPT/FMR1/MAPK1/SOD1	4
GO:0005924	cell-substrate adherens junction	9/149	408/19717	0.003906	0.015932	0.010052	PLAU/SLC6A4/DPP4/CTNNB1/MAPK1/EGFR/ICAM1/CAV1/CAT	9
GO:0000307	cyclin-dependent protein kinase holoenzyme complex	3/149	42/19717	0.003913	0.015932	0.010052	CCND1/RB1/CDKN1A	3
GO:0030055	cell-substrate junction	9/149	412/19717	0.004162	0.016747	0.010566	PLAU/SLC6A4/DPP4/CTNNB1/MAPK1/EGFR/ICAM1/CAV1/CAT	9
GO:1902554	serine/threonine protein kinase complex	4/149	88/19717	0.004469	0.017772	0.011214	CCND1/RB1/ACVR1B/CDKN1A	4
GO:0031674	I band	5/149	143/19717	0.004645	0.018258	0.01152	NOS1/CTNNB1/CACNA1C/CACNA1S/SCN5A	5
GO:0001518	voltage-gated sodium channel complex	2/149	14/19717	0.004863	0.018901	0.011926	SCN10A/SCN5A	2
GO:0005769	early endosome	8/149	350/19717	0.005205	0.01982	0.012505	APOE/RET/KCNQ1/KDR/MAPK1/EGFR/CAV1/CFTR	8
GO:0072562	blood microparticle	5/149	147/19717	0.005216	0.01982	0.012505	APOE/PON1/BCHE/F2/TGFB1	5
GO:0043209	myelin sheath	3/149	49/19717	0.006043	0.022712	0.014331	BCL2/HSP90AA1/ERBB2	3

GO:0008328	ionotropic glutamate receptor complex	3/149	51/19717	0.006756	0.025116	0.015847	GRIN2B/SHANK3/GRIN1	3
GO:0031092	platelet alpha granule membrane	2/149	17/19717	0.007161	0.026336	0.016617	SNCA/SPARC	2
GO:0099634	postsynaptic specialization membrane	4/149	101/19717	0.007258	0.026407	0.016662	GRIN2B/GRIN1/CHRM3/GABRA3	4
GO:0016328	lateral plasma membrane	3/149	57/19717	0.009186	0.032726	0.020649	DRD2/CTNNB1/SCN5A	3
GO:1904115	axon cytoplasm	3/149	57/19717	0.009186	0.032726	0.020649	MAPT/FMR1/SOD1	3
GO:1902911	protein kinase complex	4/149	109/19717	0.009444	0.033296	0.021008	CCND1/RB1/ACVR1B/CDKN1A	4
GO:0005775	vacuolar lumen	5/149	172/19717	0.009954	0.034738	0.021918	MPO/CTSD/HSP90AA1/FASLG/MAPK1	5
GO:0099091	postsynaptic specialization, intracellular component	2/149	21/19717	0.010842	0.037454	0.023632	CTNNB1/SRC	2
GO:0030662	coated vesicle membrane	5/149	182/19717	0.012483	0.042692	0.026937	APOE/AVPR2/EGFR/CFTR/AVP	5
GO:0034706	sodium channel complex	2/149	23/19717	0.012934	0.043367	0.027363	SCN10A/SCN5A	2
GO:0046930	pore complex	2/149	23/19717	0.012934	0.043367	0.027363	BCL2/BAX	2
GO:0101002	ficolin-1-rich granule	5/149	185/19717	0.013319	0.044225	0.027904	CTSD/HSP90AA1/MAPK1/CAT/MMP9	5
GO:0061695	transferase complex, transferring phosphorus-containing groups	6/149	259/19717	0.014019	0.045732	0.028855	CCND1/RB1/ACVR1B/TP53/CDKN1A/PIK3R1	6
GO:0097386	glial cell projection	2/149	24/19717	0.01404	0.045732	0.028855	MAPT/FMR1	2
GO:0030136	clathrin-coated vesicle	5/149	188/19717	0.014192	0.045791	0.028892	APOE/AVPR2/EGFR/CFTR/AVP	5

Supplementary Table S6-3: Datailed information of molecular functions(MF) enrichment of common targets

ID	Description	GeneRatio	BgRatio	pvalue	p.adjust	qvalue	geneID

GO:0019903	protein phosphatase binding	15/149	140/17697	8.23E-13	4.43E-10	2.60E-10	MET/BCL2/PPARG/MAPT/CTNNB1/STAT1/KCNQ1/HMGCR/EGFR/SOD1/ERBB2/TP53/JAK3/AKT1/PIK3R1
GO:0019902	phosphatase binding	16/149	185/17697	3.86E-12	1.04E-09	6.09E-10	MET/BCL2/PPARG/MAPT/CTNNB1/STAT1/KCNQ1/HMGCR/MAPK1/EGFR/SOD1/ERBB2/TP53/JAK3/AKT1/PIK3R1
GO:0020037	heme binding	14/149	135/17697	7.72E-12	1.38E-09	8.13E-10	NOS2/NOS1/CYP1A1/DUOX2/MPO/PTGS2/CYP2E1/HMOX1/TPO/SRC/CAT/PTGS1/CYP24A1/CYP3A4
GO:0046906	tetrapyrrole binding	14/149	145/17697	2.05E-11	2.76E-09	1.62E-09	NOS2/NOS1/CYP1A1/DUOX2/MPO/PTGS2/CYP2E1/HMOX1/TPO/SRC/CAT/PTGS1/CYP24A1/CYP3A4
GO:0042165	neurotransmitter binding	10/149	55/17697	2.79E-11	3.00E-09	1.76E-09	SLC6A4/BCHE/HTR3A/CRP/GRIN2B/ACHE/HTR1A/GRIN1/CHRM3/HTR2A
GO:0070405	ammonium ion binding	10/149	75/17697	6.86E-10	6.15E-08	3.61E-08	SLC6A4/BCHE/HTR3A/CRP/DRD2/ACHE/HTR1A/DRD4/CHRM3/HTR2A
GO:0005126	cytokine receptor binding	16/149	286/17697	2.54E-09	1.82E-07	1.07E-07	CASP8/CXCL8/TNF/IL1A/TGFB1/IL13/STAT1/IL6/CASP3/FASLG/IL10/NODAL/IL2/IFNG/PIK3R1/IL1B
GO:0016209	antioxidant activity	10/149	86/17697	2.71E-09	1.82E-07	1.07E-07	APOE/DUOX2/MPO/PTGS2/NQO1/SOD1/TPO/IYD/CAT/PTGS1
GO:0048018	receptor ligand activity	20/149	482/17697	4.18E-09	2.26E-07	1.33E-07	IGF1/LEP/MAPT/CXCL8/TNF/IL1A/F2/TGFB1/IL13/IL6/FASLG/INS/IL10/IGF2/NODAL/IL2/IFNG/FGF2/IL1B/AVP
GO:0030594	neurotransmitter receptor activity	11/149	117/17697	4.20E-09	2.26E-07	1.33E-07	HTR3A/DRD2/GRIN2B/GABRG3/HTR1A/GRIN1/GABRD/DRD4/CHRM3/HTR2A/GABRA3
GO:0015267	channel activity	19/149	456/17697	9.86E-09	4.58E-07	2.69E-07	BCL2/HTR3A/CACNA1B/GRIN2B/TRPA1/GABRG3/KCNQ1/TRPV1/ABCC9/CACNA1C/CACNA1A/GRIN1/GABRD/CACNA1S/C[illegible] A/GABRA3/BAX/SCN5A
GO:0022803	passive transmembrane transporter activity	19/149	457/17697	1.02E-08	4.58E-07	2.69E-07	BCL2/HTR3A/CACNA1B/GRIN2B/TRPA1/GABRG3/KCNQ1/TRPV1/ABCC9/CACNA1C/CACNA1A/GRIN1/GABRD/CACNA1S/C[illegible] A/GABRA3/BAX/SCN5A
GO:0005516	calmodulin binding	13/149	200/17697	1.45E-08	5.98E-07	3.51E-07	NOS2/NOS1/FAS/PLA2G6/KCNQ1/TRPV1/CACNA1C/EGFR/MYLK/GRIN1/CACNA1S/AKT1/SCN5A
GO:0022839	ion gated channel activity	16/149	334/17697	2.29E-08	8.81E-07	5.17E-07	HTR3A/CACNA1B/GRIN2B/TRPA1/GABRG3/KCNQ1/TRPV1/CACNA1C/CACNA1A/GRIN1/GABRD/CACNA1S/CFTR/SCN10A/[illegible] CN5A
GO:0022836	gated channel activity	16/149	343/17697	3.32E-08	1.19E-06	6.99E-07	HTR3A/CACNA1B/GRIN2B/TRPA1/GABRG3/KCNQ1/TRPV1/CACNA1C/CACNA1A/GRIN1/GABRD/CACNA1S/CFTR/SCN10A/[illegible] CN5A
GO:0005125	cytokine activity	13/149	220/17697	4.48E-08	1.51E-06	8.85E-07	CXCL8/TNF/IL1A/TGFB1/IL13/IL6/FASLG/IL10/NODAL/IL2/IFNG/FGF2/IL1B
GO:0005216	ion channel activity	17/149	416/17697	8.27E-08	2.62E-06	1.54E-06	HTR3A/CACNA1B/GRIN2B/TRPA1/GABRG3/KCNQ1/TRPV1/ABCC9/CACNA1C/CACNA1A/GRIN1/GABRD/CACNA1S/CFTR/S[illegible] BRA3/SCN5A
GO:0022838	substrate-specific channel activity	17/149	428/17697	1.24E-07	3.71E-06	2.18E-06	HTR3A/CACNA1B/GRIN2B/TRPA1/GABRG3/KCNQ1/TRPV1/ABCC9/CACNA1C/CACNA1A/GRIN1/GABRD/CACNA1S/CFTR/S[illegible] BRA3/SCN5A

GO:0046873	metal ion transmembrane transporter activity	17/149	438/17697	1.73E-07	4.89E-06	2.87E-06	SLC6A2/SLC6A4/CACNA1B/SLC12A2/GRIN2B/TRPA1/KCNQ1/TRPV1/ABCC9/CACNA1C/CACNA1A/GRIN1/SLC12A1/SLC6A8[illegible] SCN10A/SCN5A
GO:0051721	protein phosphatase 2A binding	6/149	32/17697	2.44E-07	6.50E-06	3.81E-06	BCL2/MAPT/STAT1/HMGCR/TP53/AKT1
GO:0004601	peroxidase activity	7/149	52/17697	2.54E-07	6.50E-06	3.81E-06	DUOX2/MPO/PTGS2/TPO/IYD/CAT/PTGS1
GO:0044389	ubiquitin-like protein ligase binding	14/149	308/17697	3.44E-07	8.41E-06	4.93E-06	BCL2/CASP8/GSK3B/STAT1/HSP90AA1/EGFR/RB1/SRC/ACVR1B/TP53/CDKN1A/NFKBIA/JUN/SCN5A
GO:0016684	oxidoreductase activity, acting on peroxide as acceptor	7/149	56/17697	4.28E-07	1.00E-05	5.87E-06	DUOX2/MPO/PTGS2/TPO/IYD/CAT/PTGS1
GO:0033613	activating transcription factor binding	8/149	85/17697	5.82E-07	1.30E-05	7.66E-06	PRDM16/PPARG/NFE2L2/CTNNB1/MYC/FOS/RB1/JUN
GO:0097110	scaffold protein binding	7/149	59/17697	6.16E-07	1.33E-05	7.78E-06	NOS1/CASP8/SHANK3/KCNQ1/HSP90AA1/SRC/SCN5A
GO:0005158	insulin receptor binding	5/149	22/17697	9.28E-07	1.69E-05	9.93E-06	IGF1/INS/IGF2/SRC/PIK3R1
GO:0016705	oxidoreductase activity, acting on paired donors, with incorporation or reduction of molecular oxygen	10/149	159/17697	9.66E-07	1.69E-05	9.93E-06	NOS2/NOS1/CYP1A1/PTGS2/CYP2E1/HMOX1/PTGS1/CYP24A1/CYP3A4/PAH
GO:0008331	high voltage-gated calcium channel activity	4/149	10/17697	9.74E-07	1.69E-05	9.93E-06	CACNA1B/CACNA1C/CACNA1A/CACNA1S
GO:0015378	sodium:chloride symporter activity	4/149	10/17697	9.74E-07	1.69E-05	9.93E-06	SLC6A2/SLC6A4/SLC12A2/SLC12A1
GO:0043176	amine binding	4/149	10/17697	9.74E-07	1.69E-05	9.93E-06	SLC6A4/HTR3A/HTR1A/HTR2A
GO:0051378	serotonin binding	4/149	10/17697	9.74E-07	1.69E-05	9.93E-06	SLC6A4/HTR3A/HTR1A/HTR2A
GO:0044325	ion channel binding	9/149	124/17697	1.04E-06	1.75E-05	1.03E-05	NOS1/CTNNB1/KCNQ1/ABCC9/FMR1/CAV1/SRC/SCN10A/SCN5A
GO:0031625	ubiquitin protein ligase binding	13/149	290/17697	1.07E-06	1.75E-05	1.03E-05	BCL2/CASP8/GSK3B/HSP90AA1/EGFR/RB1/SRC/ACVR1B/TP53/CDKN1A/NFKBIA/JUN/SCN5A
GO:0004713	protein tyrosine kinase activity	9/149	134/17697	1.99E-06	3.11E-05	1.83E-05	MET/ALK/RET/KDR/EGFR/ERBB2/ABL1/SRC/JAK3
GO:0015108	chloride transmembrane transporter activity	8/149	100/17697	2.02E-06	3.11E-05	1.83E-05	SLC6A2/SLC6A4/SLC12A2/GABRG3/SLC12A1/GABRD/CFTR/GABRA3
GO:0015276	ligand-gated ion channel activity	9/149	138/17697	2.54E-06	3.70E-05	2.17E-05	HTR3A/GRIN2B/TRPA1/GABRG3/TRPV1/GRIN1/GABRD/CFTR/GABRA3

GO:0022834	ligand-gated channel activity	9/149	138/17697	2.54E-06	3.70E-05	2.17E-05	HTR3A/GRIN2B/TRPA1/GABRG3/TRPV1/GRIN1/GABRD/CFTR/GABRA3
GO:0005261	cation channel activity	13/149	319/17697	3.09E-06	4.37E-05	2.57E-05	HTR3A/CACNA1B/GRIN2B/TRPA1/KCNQ1/TRPV1/ABCC9/CACNA1C/CACNA1A/GRIN1/CACNA1S/SCN10A/SCN5A
GO:0005230	extracellular ligand-gated ion channel activity	7/149	75/17697	3.20E-06	4.38E-05	2.57E-05	HTR3A/GRIN2B/GABRG3/TRPV1/GRIN1/GABRD/GABRA3
GO:0015373	anion:sodium symporter activity	4/149	13/17697	3.25E-06	4.38E-05	2.57E-05	SLC6A2/SLC6A4/SLC12A2/SLC12A1
GO:0001540	amyloid-beta binding	7/149	78/17697	4.17E-06	5.48E-05	3.21E-05	APOE/BCHE/CACNA1B/GRIN2B/ACHE/CACNA1A/GRIN1
GO:0019199	transmembrane receptor protein kinase activity	7/149	79/17697	4.55E-06	5.82E-05	3.42E-05	MET/ALK/RET/KDR/EGFR/ERBB2/ACVR1B
GO:0051219	phosphoprotein binding	7/149	83/17697	6.33E-06	7.92E-05	4.65E-05	SNCA/TRPV1/MAPK1/ABL1/RB1/SRC/PIK3R1
GO:0001085	RNA polymerase II transcription factor binding	9/149	155/17697	6.60E-06	8.07E-05	4.74E-05	PPARG/NFE2L2/CTNNB1/ESR1/GSK3B/FOS/RB1/TP53/JUN
GO:0005159	insulin-like growth factor receptor binding	4/149	16/17697	8.12E-06	9.50E-05	5.57E-05	IGF1/INS/IGF2/PIK3R1
GO:0015377	cation:chloride symporter activity	4/149	16/17697	8.12E-06	9.50E-05	5.57E-05	SLC6A2/SLC6A4/SLC12A2/SLC12A1
GO:0005262	calcium channel activity	8/149	123/17697	9.52E-06	0.000109	6.39E-05	CACNA1B/GRIN2B/TRPA1/TRPV1/CACNA1C/CACNA1A/GRIN1/CACNA1S
GO:0008083	growth factor activity	9/149	163/17697	9.92E-06	0.00011	6.47E-05	IGF1/F2/TGFB1/IL6/IL10/IGF2/NODAL/IL2/FGF2
GO:0008227	G protein-coupled amine receptor activity	6/149	59/17697	1.00E-05	0.00011	6.47E-05	ADRA2A/DRD2/HTR1A/DRD4/CHRM3/HTR2A
GO:0022824	transmitter-gated ion channel activity	6/149	61/17697	1.22E-05	0.000129	7.56E-05	HTR3A/GRIN2B/GABRG3/GRIN1/GABRD/GABRA3
GO:0022835	transmitter-gated channel activity	6/149	61/17697	1.22E-05	0.000129	7.56E-05	HTR3A/GRIN2B/GABRG3/GRIN1/GABRD/GABRA3
GO:0002020	protease binding	8/149	128/17697	1.28E-05	0.000132	7.74E-05	COL1A1/DPP4/BCL2/TNF/GSK3B/CASP3/INS/TP53
GO:0004714	transmembrane receptor protein tyrosine kinase activity	6/149	62/17697	1.34E-05	0.000136	8.00E-05	MET/ALK/RET/KDR/EGFR/ERBB2
GO:0005178	integrin binding	8/149	132/17697	1.60E-05	0.000159	9.34E-05	IGF1/KDR/IGF2/EGFR/FGF2/ICAM1/SRC/IL1B

GO:0015296	anion:cation symporter activity	4/149	19/17697	1.70E-05	0.000166	9.73E-05	SLC6A2/SLC6A4/SLC12A2/SLC12A1
GO:0004497	monooxygenase activity	7/149	99/17697	2.03E-05	0.000195	0.000114	NOS2/NOS1/CYP1A1/CYP2E1/CYP24A1/CYP3A4/PAH
GO:0015085	calcium ion transmembrane transporter activity	8/149	139/17697	2.32E-05	0.000219	0.000129	CACNA1B/GRIN2B/TRPA1/TRPV1/CACNA1C/CACNA1A/GRIN1/CACNA1S
GO:0030331	estrogen receptor binding	5/149	42/17697	2.62E-05	0.000243	0.000143	PPARG/CTNNB1/ESR1/PARP1/SRC
GO:0017171	serine hydrolase activity	9/149	186/17697	2.84E-05	0.000259	0.000152	MMP2/PLAU/DPP4/PLA2G6/F2/ACHE/MMP1/F12/MMP9
GO:0016247	channel regulator activity	8/149	144/17697	3.00E-05	0.000269	0.000158	NOS1/BCL2/NRXN1/TRPV1/ABCC9/CAV1/DRD4/CFTR
GO:0015103	inorganic anion transmembrane transporter activity	8/149	146/17697	3.31E-05	0.000292	0.000171	SLC6A2/SLC6A4/SLC12A2/GABRG3/SLC12A1/GABRD/CFTR/GABRA3
GO:0032813	tumor necrosis factor receptor superfamily binding	5/149	46/17697	4.11E-05	0.000352	0.000207	CASP8/TNF/STAT1/CASP3/FASLG
GO:0042277	peptide binding	11/149	295/17697	4.12E-05	0.000352	0.000207	APOE/BCHE/OPRK1/PPARG/CACNA1B/GRIN2B/ACHE/CACNA1A/GRIN1/PIK3R1/NFKBIA
GO:0005244	voltage-gated ion channel activity	9/149	197/17697	4.46E-05	0.000366	0.000215	CACNA1B/GRIN2B/KCNQ1/CACNA1C/CACNA1A/GRIN1/CACNA1S/SCN10A/SCN5A
GO:0022832	voltage-gated channel activity	9/149	197/17697	4.46E-05	0.000366	0.000215	CACNA1B/GRIN2B/KCNQ1/CACNA1C/CACNA1A/GRIN1/CACNA1S/SCN10A/SCN5A
GO:0004879	nuclear receptor activity	5/149	47/17697	4.56E-05	0.000366	0.000215	THRB/PPARG/ESR1/PGR/THRA
GO:0098531	transcription factor activity, direct ligand regulated sequence-specific DNA binding	5/149	47/17697	4.56E-05	0.000366	0.000215	THRB/PPARG/ESR1/PGR/THRA
GO:0046332	SMAD binding	6/149	80/17697	5.79E-05	0.000458	0.000269	PRDM16/CTNNB1/PARP1/FOS/ACVR1B/JUN
GO:0099604	ligand-gated calcium channel activity	4/149	27/17697	7.28E-05	0.000568	0.000333	GRIN2B/TRPA1/TRPV1/GRIN1
GO:0001102	RNA polymerase II activating transcription factor binding	5/149	53/17697	8.20E-05	0.00063	0.00037	NFE2L2/CTNNB1/FOS/RB1/JUN
GO:0001228	DNA-binding transcription activator activity, RNA polymerase II-specific	13/149	439/17697	8.87E-05	0.000672	0.000394	IRF1/NFE2L2/SOX9/ESR1/MYC/STAT1/PARP1/FOS/PGR/TP53/NFKB1/JUN/NOTCH1
GO:0003707	steroid hormone receptor activity	5/149	56/17697	0.000107	0.000799	0.000469	THRB/PPARG/ESR1/PGR/THRA

GO:00085			30/1769	0.0001	0.0008	0.0004	
19	ammonium transmembrane transporter activity	4/149	7	12	22	83	SLC6A2/SLC6A4/SLC12A2/SLC6A8
GO:00352			92/1769	0.0001	0.0009	0.0005	
58	steroid hormone receptor binding	6/149	7	27	13	36	PPARG/CTNNB1/ESR1/PARP1/RB1/SRC
GO:00051			31/1769	0.0001	0.0009	0.0005	
64	tumor necrosis factor receptor binding	4/149	7	27	13	36	CASP8/TNF/STAT1/FASLG
GO:00353			133/176	0.0001	0.0009	0.0005	
26	enhancer binding	7/149	97	34	47	56	NFE2L2/SOX9/NKX2-1/TCF3/TP53/NFKB1/JUN
GO:00708			134/176	0.0001	0.0009	0.0005	
51	growth factor receptor binding	7/149	97	4	79	75	IL1A/IL6/IL10/IL2/FGF2/SRC/IL1B
GO:00166			13/1769	0.0001	0.0010	0.0006	
53	oxidoreductase activity, acting on NAD(P)H, heme protein as acceptor	3/149	7	57	71	29	NOS2/NOS1/NQO1
GO:00342			13/1769	0.0001	0.0010	0.0006	
36	protein kinase A catalytic subunit binding	3/149	7	57	71	29	SOX9/GSK3B/KCNQ1
GO:00198			137/176	0.0001	0.0010	0.0006	
38	growth factor binding	7/149	97	61	81	35	COL1A1/KDR/EGFR/ERBB2/ACVR1B/IGFBP3/SCN5A
GO:00977			33/1769	0.0001	0.0010	0.0006	
18	disordered domain specific binding	4/149	7	63	85	37	CTNNB1/HSP90AA1/RB1/TP53
GO:00514			185/176	0.0001	0.0011	0.0006	
27	hormone receptor binding	8/149	97	74	42	7	LEP/PPARG/CTNNB1/ESR1/STAT1/PARP1/RB1/SRC
GO:00049			34/1769	0.0001	0.0011	0.0006	
93	G protein-coupled serotonin receptor activity	4/149	7	84	78	91	HTR1A/DRD4/CHRM3/HTR2A
GO:00995			34/1769	0.0001	0.0011	0.0006	
89	serotonin receptor activity	4/149	7	84	78	91	HTR1A/DRD4/CHRM3/HTR2A
GO:00509			14/1769	0.0001	0.0012	0.0007	
98	nitric-oxide synthase binding	3/149	7	99	55	37	SLC6A4/CAV1/SCN5A
GO:00228			142/176	0.0002	0.0012	0.0007	
43	voltage-gated cation channel activity	7/149	97	01	55	37	CACNA1B/GRIN2B/KCNQ1/CACNA1C/CACNA1A/GRIN1/CACNA1S
GO:00332			356/176	0.0002	0.0013	0.0007	
18	amide binding	11/149	97	18	46	9	APOE/BCHE/OPRK1/PPARG/CACNA1B/GRIN2B/ACHE/CACNA1A/GRIN1/PIK3R1/NFKBIA
GO:00425			102/176	0.0002	0.0013	0.0008	
62	hormone binding	6/149	97	23	64	01	THRB/ACHE/EGFR/THRA/PIK3R1/CHRM3
GO:00971			15/1769	0.0002	0.0014	0.0008	
53	cysteine-type endopeptidase activity involved in apoptotic process	3/149	7	47	94	77	CASP8/CASP3/CASP9
GO:00150			149/176	0.0002	0.0016	0.0009	
81	sodium ion transmembrane transporter activity	7/149	97	7	11	46	SLC6A2/SLC6A4/SLC12A2/SLC12A1/SLC6A8/SCN10A/SCN5A

GO:0010181	FMN binding	3/149	16/17697	0.000302	0.001761	0.001033	NOS2/NOS1/IYD
GO:0005506	iron ion binding	7/149	152/17697	0.000304	0.001761	0.001033	CYP1A1/CYP2E1/SNCA/FECH/CYP24A1/CYP3A4/PAH
GO:0035257	nuclear hormone receptor binding	7/149	152/17697	0.000304	0.001761	0.001033	PPARG/CTNNB1/ESR1/STAT1/PARP1/RB1/SRC
GO:0016709	oxidoreductase activity, acting on paired donors, with incorporation or reduction of molecular oxygen, NAD(P)H as one donor, and incorporation of one atom of oxygen	4/149	39/17697	0.000316	0.001807	0.00106	NOS2/NOS1/CYP1A1/CYP2E1
GO:0070491	repressing transcription factor binding	5/149	71/17697	0.000329	0.001866	0.001095	PPARG/CTNNB1/MYC/STAT1/TCF3
GO:0015370	solute:sodium symporter activity	5/149	72/17697	0.000352	0.001971	0.001157	SLC6A2/SLC6A4/SLC12A2/SLC12A1/SLC6A8
GO:0005123	death receptor binding	3/149	17/17697	0.000365	0.002023	0.001187	CASP8/CASP3/FASLG
GO:0003713	transcription coactivator activity	10/149	319/17697	0.000377	0.002064	0.001211	PRDM16/THRB/PPARG/CTNNB1/FGF2/ABL1/RB1/THRA/TCF3/JUN
GO:0051879	Hsp90 protein binding	4/149	41/17697	0.000384	0.002064	0.001211	CYP1A1/MAPT/CYP2E1/KDR
GO:0099528	G protein-coupled neurotransmitter receptor activity	4/149	41/17697	0.000384	0.002064	0.001211	HTR1A/DRD4/CHRM3/HTR2A
GO:0004252	serine-type endopeptidase activity	7/149	160/17697	0.000415	0.002211	0.001298	MMP2/PLAU/DPP4/F2/MMP1/F12/MMP9
GO:0030544	Hsp70 protein binding	4/149	42/17697	0.000421	0.002222	0.001304	CYP1A1/CYP2E1/SNCA/BAX
GO:0008509	anion transmembrane transporter activity	10/149	327/17697	0.000458	0.002392	0.001404	SLC6A2/SLC6A4/SLC12A2/GABRG3/SLC25A4/SLC12A1/GABRD/SLC6A8/CFTR/GABRA3
GO:0001158	enhancer sequence-specific DNA binding	6/149	119/17697	0.000512	0.002606	0.001529	NFE2L2/SOX9/NKX2-1/TP53/NFKB1/JUN
GO:0031072	heat shock protein binding	6/149	119/17697	0.000512	0.002606	0.001529	CYP1A1/MAPT/CYP2E1/SNCA/KDR/BAX
GO:0004890	GABA-A receptor activity	3/149	19/17697	0.000513	0.002606	0.001529	GABRG3/GABRD/GABRA3
GO:0048156	tau protein binding	4/149	45/17697	0.00055	0.002765	0.001623	APOE/SNCA/GSK3B/HSP90AA1
GO:0099095	ligand-gated anion channel activity	3/149	20/17697	0.0006	0.002963	0.001739	GABRG3/CFTR/GABRA3

GO:1901338	catecholamine binding	3/149	20/17697	0.0006	0.002963	0.001739	ADRA2A/DRD2/DRD4
GO:1904315	transmitter-gated ion channel activity involved in regulation of postsynaptic membrane potential	4/149	47/17697	0.00065	0.003178	0.001865	GABRG3/GRIN1/GABRD/GABRA3
GO:0005245	voltage-gated calcium channel activity	4/149	48/17697	0.000704	0.003413	0.002003	CACNA1B/CACNA1C/CACNA1A/CACNA1S
GO:0016917	GABA receptor activity	3/149	22/17697	0.000801	0.003847	0.002258	GABRG3/GABRD/GABRA3
GO:0070888	E-box binding	4/149	50/17697	0.000823	0.003883	0.002279	PPARG/MYC/TCF3/SNAI2
GO:0099529	neurotransmitter receptor activity involved in regulation of postsynaptic membrane potential	4/149	50/17697	0.000823	0.003883	0.002279	GABRG3/GRIN1/GABRD/GABRA3
GO:0008236	serine-type peptidase activity	7/149	182/17697	0.000892	0.004174	0.00245	MMP2/PLAU/DPP4/F2/MMP1/F12/MMP9
GO:0070412	R-SMAD binding	3/149	23/17697	0.000915	0.004246	0.002492	PARP1/FOS/JUN
GO:0050661	NADP binding	4/149	52/17697	0.000955	0.004352	0.002555	NOS2/NOS1/HMGCR/CAT
GO:0098960	postsynaptic neurotransmitter receptor activity	4/149	52/17697	0.000955	0.004352	0.002555	GABRG3/GRIN1/GABRD/GABRA3
GO:0004175	endopeptidase activity	11/149	427/17697	0.000994	0.004496	0.002639	MMP2/PLAU/DPP4/CASP8/F2/CTSD/MMP1/CASP3/CASP9/F12/MMP9
GO:0008022	protein C-terminus binding	7/149	187/17697	0.001045	0.004685	0.00275	PPARG/CACNA1B/CTNNB1/SHANK3/ERBB2/ABL1/SRC
GO:0033612	receptor serine/threonine kinase binding	3/149	25/17697	0.001174	0.005201	0.003053	OPRK1/NODAL/SRC
GO:0001047	core promoter binding	4/149	55/17697	0.001179	0.005201	0.003053	MYC/STAT1/FOS/TP53
GO:0005496	steroid binding	5/149	95/17697	0.001248	0.005457	0.003203	APOE/ESR1/CAV1/PGR/CYP3A4
GO:0043621	protein self-association	4/149	56/17697	0.001262	0.005475	0.003214	PPARG/SHANK3/ACHE/TP53
GO:0005328	neurotransmitter:sodium symporter activity	3/149	26/17697	0.001319	0.005679	0.003333	SLC6A2/SLC6A4/SLC6A8
GO:0000980	RNA polymerase II distal enhancer sequence-specific DNA binding	5/149	99/17697	0.001499	0.006402	0.003757	NFE2L2/NKX2-1/TP53/NFKB1/JUN

GO:0005507	copper ion binding	4/149	59/17697	0.001533	0.006445	0.003783	IL1A/SNCA/SOD1/TP53
GO:0016597	amino acid binding	4/149	59/17697	0.001533	0.006445	0.003783	NOS2/NOS1/GRIN2B/GRIN1
GO:0015294	solute:cation symporter activity	5/149	102/17697	0.001711	0.007082	0.004157	SLC6A2/SLC6A4/SLC12A2/SLC12A1/SLC6A8
GO:0051087	chaperone binding	5/149	102/17697	0.001711	0.007082	0.004157	MAPT/SOD1/CFTR/TP53/BAX
GO:0019207	kinase regulator activity	7/149	207/17697	0.001873	0.007692	0.004515	CCND1/TGFB1/CASP3/IGF2/IL2/CDKN1A/PIK3R1
GO:0099094	ligand-gated cation channel activity	5/149	105/17697	0.001944	0.007925	0.004651	HTR3A/GRIN2B/TRPA1/TRPV1/GRIN1
GO:0016712	oxidoreductase activity, acting on paired donors, with incorporation or reduction of molecular oxygen, reduced flavin or flavoprotein as one donor, and incorporation of one atom of oxygen	3/149	32/17697	0.002426	0.009772	0.005736	CYP1A1/CYP2E1/CYP3A4
GO:0005518	collagen binding	4/149	67/17697	0.002452	0.009772	0.005736	ACHE/SERPINH1/SPARC/MMP9
GO:0030374	nuclear receptor transcription coactivator activity	4/149	67/17697	0.002452	0.009772	0.005736	THRB/PPARG/FGF2/THRA
GO:0005217	intracellular ligand-gated ion channel activity	3/149	33/17697	0.002652	0.010339	0.006069	TRPA1/TRPV1/CFTR
GO:0071813	lipoprotein particle binding	3/149	33/17697	0.002652	0.010339	0.006069	APOE/CRP/MAPT
GO:0071814	protein-lipid complex binding	3/149	33/17697	0.002652	0.010339	0.006069	APOE/CRP/MAPT
GO:0004952	dopamine neurotransmitter receptor activity	2/149	10/17697	0.003031	0.011485	0.006741	DRD2/DRD4
GO:0016595	glutamate binding	2/149	10/17697	0.003031	0.011485	0.006741	GRIN2B/GRIN1
GO:0051400	BH domain binding	2/149	10/17697	0.003031	0.011485	0.006741	BCL2/BAX
GO:0070513	death domain binding	2/149	10/17697	0.003031	0.011485	0.006741	BCL2/BAX
GO:0099106	ion channel regulator activity	5/149	118/17697	0.00323	0.012151	0.007132	NRXN1/ABCC9/CAV1/DRD4/CFTR
GO:0050839	cell adhesion molecule binding	11/149	499/17697	0.003366	0.012576	0.007382	IGF1/CTNNB1/NRXN1/STAT1/KDR/IGF2/EGFR/FGF2/ICAM1/SRC/IL1B

GO:0019825	oxygen binding	3/149	36/17697	0.003407	0.012642	0.007421	CYP1A1/CYP2E1/CYP3A4
GO:0001618	virus receptor activity	4/149	74/17697	0.003518	0.012875	0.007557	DPP4/EGFR/ICAM1/HTR2A
GO:0104005	hijacked molecular function	4/149	74/17697	0.003518	0.012875	0.007557	DPP4/EGFR/ICAM1/HTR2A
GO:0022804	active transmembrane transporter activity	9/149	362/17697	0.003606	0.013109	0.007695	SLC6A2/SLC6A4/SLC12A2/ABCB1/ABCC9/SLC25A4/SLC12A1/SLC6A8/CFTR
GO:0008503	benzodiazepine receptor activity	2/149	11/17697	0.003685	0.013242	0.007773	GABRG3/GABRA3
GO:0005254	chloride channel activity	4/149	75/17697	0.003692	0.013242	0.007773	GABRG3/GABRD/CFTR/GABRA3
GO:0005179	hormone activity	5/149	122/17697	0.003726	0.013274	0.007791	IGF1/LEP/INS/IGF2/AVP
GO:0008395	steroid hydroxylase activity	3/149	38/17697	0.003977	0.013983	0.008208	CYP1A1/CYP2E1/CYP3A4
GO:0016248	channel inhibitor activity	3/149	38/17697	0.003977	0.013983	0.008208	BCL2/CAV1/CFTR
GO:0004861	cyclin-dependent protein serine/threonine kinase inhibitor activity	2/149	12/17697	0.004397	0.015262	0.008958	CASP3/CDKN1A
GO:0034452	dynactin binding	2/149	12/17697	0.004397	0.015262	0.008958	MAPT/GSK3B
GO:0001784	phosphotyrosine residue binding	3/149	40/17697	0.004601	0.015867	0.009313	MAPK1/ABL1/PIK3R1
GO:0017124	SH3 domain binding	5/149	130/17697	0.004877	0.016713	0.00981	MAPT/SHANK3/ABL1/DRD4/CASP9
GO:0015077	monovalent inorganic cation transmembrane transporter activity	9/149	382/17697	0.00511	0.017377	0.0102	SLC6A2/SLC6A4/SLC12A2/KCNQ1/ABCC9/SLC12A1/SLC6A8/SCN10A/SCN5A
GO:0022851	GABA-gated chloride ion channel activity	2/149	13/17697	0.005168	0.017377	0.0102	GABRG3/GABRA3
GO:0035240	dopamine binding	2/149	13/17697	0.005168	0.017377	0.0102	DRD2/DRD4
GO:0052689	carboxylic ester hydrolase activity	5/149	136/17697	0.005893	0.019598	0.011503	PLA2G2A/PON1/BCHE/PLA2G6/ACHE
GO:0031406	carboxylic acid binding	6/149	193/17697	0.005901	0.019598	0.011503	NOS2/NOS1/PPARG/GRIN2B/S100A8/GRIN1

GO:0036041	long-chain fatty acid binding	2/149	14/17697	0.005996	0.019711	0.01157	PPARG/S100A8
GO:0019209	kinase activator activity	4/149	86/17697	0.006008	0.019711	0.01157	TGFB1/IGF2/IL2/CDKN1A
GO:0001046	core promoter sequence-specific DNA binding	3/149	45/17697	0.006409	0.020896	0.012266	STAT1/FOS/TP53
GO:0005253	anion channel activity	4/149	89/17697	0.006776	0.021821	0.012808	GABRG3/GABRD/CFTR/GABRA3
GO:0004715	non-membrane spanning protein tyrosine kinase activity	3/149	46/17697	0.006814	0.021821	0.012808	ABL1/SRC/JAK3
GO:0042805	actinin binding	3/149	46/17697	0.006814	0.021821	0.012808	PPARG/CACNA1C/NFKB1
GO:0016594	glycine binding	2/149	15/17697	0.006881	0.021904	0.012857	GRIN2B/GRIN1
GO:0015293	symporter activity	5/149	144/17697	0.007467	0.02363	0.01387	SLC6A2/SLC6A4/SLC12A2/SLC12A1/SLC6A8
GO:0015238	drug transmembrane transporter activity	4/149	92/17697	0.007606	0.023931	0.014047	SLC6A2/ABCB1/SLC25A4/SLC6A8
GO:0005149	interleukin-1 receptor binding	2/149	16/17697	0.00782	0.024264	0.014242	IL1A/IL1B
GO:0005237	inhibitory extracellular ligand-gated ion channel activity	2/149	16/17697	0.00782	0.024264	0.014242	GABRG3/GABRA3
GO:0043177	organic acid binding	6/149	205/17697	0.007847	0.024264	0.014242	NOS2/NOS1/PPARG/GRIN2B/S100A8/GRIN1
GO:0016538	cyclin-dependent protein serine/threonine kinase regulator activity	3/149	49/17697	0.00812	0.024964	0.014653	CCND1/CASP3/CDKN1A
GO:0051018	protein kinase A binding	3/149	50/17697	0.008586	0.026246	0.015406	SOX9/GSK3B/KCNQ1
GO:0004935	adrenergic receptor activity	2/149	17/17697	0.008815	0.026346	0.015464	ADRA2A/DRD2
GO:0008391	arachidonic acid monooxygenase activity	2/149	17/17697	0.008815	0.026346	0.015464	CYP1A1/CYP2E1
GO:0017081	chloride channel regulator activity	2/149	17/17697	0.008815	0.026346	0.015464	TRPV1/CFTR
GO:0042166	acetylcholine binding	2/149	17/17697	0.008815	0.026346	0.015464	ACHE/CHRM3

GO:0005326	neurotransmitter transporter activity	3/149	51/17697	0.009067	0.026803	0.015733	SLC6A2/SLC6A4/SLC6A8
GO:0045309	protein phosphorylated amino acid binding	3/149	51/17697	0.009067	0.026803	0.015733	MAPK1/ABL1/PIK3R1
GO:0015459	potassium channel regulator activity	3/149	52/17697	0.009564	0.028117	0.016504	ABCC9/CAV1/DRD4
GO:0031435	mitogen-activated protein kinase kinase kinase binding	2/149	18/17697	0.009862	0.02868	0.016834	MAPK1/TCF3
GO:0070064	proline-rich region binding	2/149	18/17697	0.009862	0.02868	0.016834	CCND1/ABL1
GO:0005080	protein kinase C binding	3/149	53/17697	0.010076	0.029145	0.017107	ABL1/SRC/AKT1
GO:0004620	phospholipase activity	4/149	102/17697	0.010848	0.031204	0.018316	PLA2G2A/PLA2G6/HMOX1/CHRM3
GO:0004970	ionotropic glutamate receptor activity	2/149	19/17697	0.010962	0.031204	0.018316	GRIN2B/GRIN1
GO:0070696	transmembrane receptor protein serine/threonine kinase binding	2/149	19/17697	0.010962	0.031204	0.018316	NODAL/SRC
GO:0050662	coenzyme binding	7/149	291/17697	0.011723	0.033194	0.019484	NOS2/NOS1/PARP1/HMGCR/MAOB/IYD/CAT
GO:0001091	RNA polymerase II basal transcription factor binding	2/149	20/17697	0.012113	0.033942	0.019923	ESR1/TP53
GO:0005248	voltage-gated sodium channel activity	2/149	20/17697	0.012113	0.033942	0.019923	SCN10A/SCN5A
GO:0016651	oxidoreductase activity, acting on NAD(P)H	4/149	107/17697	0.012758	0.035564	0.020875	NOS2/NOS1/DUOX2/NQO1
GO:0015278	calcium-release channel activity	2/149	21/17697	0.013315	0.036548	0.021453	TRPA1/TRPV1
GO:0050750	low-density lipoprotein particle receptor binding	2/149	21/17697	0.013315	0.036548	0.021453	APOE/CRP
GO:0070840	dynein complex binding	2/149	21/17697	0.013315	0.036548	0.021453	SNCA/FMR1
GO:0047485	protein N-terminus binding	4/149	109/17697	0.013579	0.037083	0.021766	SNCA/TGFB1/PARP1/TP53
GO:0000149	SNARE binding	4/149	110/17697	0.014001	0.038043	0.02233	SLC6A4/SNCA/CACNA1A/ABL1

GO:00428 26	histone deacetylase binding	4/149	111/176 97	0.0144 32	0.0390 17	0.0229 02	CCND1/HSP90AA1/PARP1/TP53
GO:00170 25	TBP-class protein binding	2/149	22/1769 7	0.0145 66	0.0391 83	0.0229 99	ESR1/THRA
GO:00152 91	secondary active transmembrane transporter activity	6/149	237/176 97	0.0152 4	0.0407 91	0.0239 43	SLC6A2/SLC6A4/SLC12A2/SLC25A4/SLC12A1/SLC6A8
GO:00081 98	ferrous iron binding	2/149	24/1769 7	0.0172 14	0.0458 48	0.0269 11	SNCA/FECH
GO:00198 87	protein kinase regulator activity	5/149	180/176 97	0.0182 1	0.0482 61	0.0283 28	CCND1/TGFB1/CASP3/IGF2/CDKN1A
GO:00041 90	aspartic-type endopeptidase activity	2/149	25/1769 7	0.0186 09	0.0483 66	0.0283 89	CTSD/CASP3
GO:00430 27	cysteine-type endopeptidase inhibitor activity involved in apoptotic process	2/149	25/1769 7	0.0186 09	0.0483 66	0.0283 89	SNCA/AVP
GO:01025 67	phospholipase A2 activity (consuming 1,2-dipalmitoylphosphatidylcholine)	2/149	25/1769 7	0.0186 09	0.0483 66	0.0283 89	PLA2G2A/PLA2G6
GO:01025 68	phospholipase A2 activity consuming 1,2-dioleoylphosphatidylethanolamine)	2/149	25/1769 7	0.0186 09	0.0483 66	0.0283 89	PLA2G2A/PLA2G6

Supplementary Table S6-4: Datailed information of KEGG pathways enrichment of common targets

ID	Description	GeneRatio	BgRatio	pvalue	p.adjust	qvalue	geneID
hsa0516 1	Hepatitis B	26/139	162/811 1	1.35E-18	3.47E-16	1.07E-16	FAS/BCL2/CASP8/CXCL8/TNF/TGFB1/MYC/STAT1/IL6/CASP3/FASLG/MAPK1/FOS/RB1/SRC/TP53/CASP9/CDKN1A/JAK3/AKT1/PIK3R1/NFKB1/NFKBIA/JUN/BAX/MMP9
hsa0541 7	Lipid and atherosclerosis	28/139	215/811 1	1.71E-17	2.20E-15	6.77E-16	FAS/BCL2/CYP1A1/CASP8/PPARG/CXCL8/NFE2L2/TNF/GSK3B/MMP1/IL6/HSP90AA1/CASP3/FASLG/MAPK1/FOS/ICAM1/SRC/TP53/CASP9/AKT1/PIK3R1/NFKB1/IL1B/NFKBIA/JUN/BAX/MMP9
hsa0520 5	Proteoglycans in cancer	27/139	205/811 1	5.18E-17	4.44E-15	1.36E-15	MMP2/IGF1/PLAU/MET/COL1A1/FAS/TNF/CTNNB1/CCND1/ESR1/TGFB1/MYC/CASP3/FASLG/KDR/MAPK1/IGF2/EGFR/ERBB2/FGF2/CAV1/SRC/TP53/CDKN1A/AKT1/PIK3R1/MMP9
hsa0521 5	Prostate cancer	20/139	97/8111	1.06E-16	6.84E-15	2.10E-15	IGF1/PLAU/BCL2/CTNNB1/CCND1/GSK3B/HSP90AA1/INS/MAPK1/EGFR/ERBB2/RB1/TP53/CASP9/CDKN1A/AKT1/PIK3R1/NFKB1/NFKBIA/MMP9
hsa0516 7	Kaposi sarcoma-associated herpesvirus infection	26/139	194/811 1	1.36E-16	7.01E-15	2.15E-15	FAS/CASP8/CXCL8/PTGS2/CTNNB1/CCND1/MYC/GSK3B/STAT1/IL6/CASP3/MAPK1/FOS/FGF2/ICAM1/RB1/SRC/TP53/CASP9/CDKN1A/AKT1/PIK3R1/NFKB1/NFKBIA/JUN/BAX
hsa0465 7	IL-17 signaling pathway	19/139	94/8111	9.72E-16	3.35E-14	1.03E-14	CASP8/CXCL8/TNF/PTGS2/S100A8/IL13/GSK3B/MMP1/IL6/HSP90AA1/CASP3/MAPK1/IFNG/FOS/NFKB1/IL1B/NFKBIA/JUN/MMP9

hsa05162	Measles	22/139	139/8111	1.04E-15	3.35E-14	1.03E-14	FAS/BCL2/CASP8/IL1A/CCND1/GSK3B/STAT1/IL6/CASP3/FASLG/IL2/FOS/TP53/CASP9/JAK3/AKT1/PIK3R1/NFKB1/IL1B/NFKBIA/JUN/BAX
hsa05418	Fluid shear stress and atherosclerosis	22/139	139/8111	1.04E-15	3.35E-14	1.03E-14	MMP2/BCL2/NFE2L2/TNF/IL1A/CTNNB1/HSP90AA1/KDR/HMOX1/NQO1/IFNG/FOS/ICAM1/CAV1/SRC/TP53/AKT1/PIK3R1/NFKB1/IL1B/JUN/MMP9
hsa01522	Endocrine resistance	19/139	98/8111	2.21E-15	6.30E-14	1.94E-14	MMP2/IGF1/BCL2/CCND1/ESR1/MAPK1/EGFR/ERBB2/FOS/RB1/SRC/TP53/CDKN1A/AKT1/PIK3R1/JUN/NOTCH1/BAX/MMP9
hsa04933	AGE-RAGE signaling pathway in diabetic complications	19/139	100/8111	3.28E-15	8.42E-14	2.59E-14	MMP2/COL1A1/BCL2/CXCL8/TNF/IL1A/CCND1/TGFB1/STAT1/IL6/CASP3/MAPK1/ICAM1/AKT1/PIK3R1/NFKB1/IL1B/JUN/BAX
hsa05142	Chagas disease	19/139	102/8111	4.82E-15	1.13E-13	3.46E-14	NOS2/FAS/CASP8/CXCL8/TNF/TGFB1/IL6/FASLG/IL10/MAPK1/IL2/IFNG/FOS/AKT1/PIK3R1/NFKB1/IL1B/NFKBIA/JUN
hsa05160	Hepatitis C	22/139	157/8111	1.46E-14	3.12E-13	9.59E-14	FAS/CASP8/TNF/CTNNB1/CCND1/MYC/GSK3B/STAT1/CASP3/FASLG/MAPK1/EGFR/IFNG/RB1/TP53/CASP9/CDKN1A/AKT1/PIK3R1/NFKB1/NFKBIA/BAX
hsa05163	Human cytomegalovirus infection	25/139	225/8111	4.81E-14	9.43E-13	2.90E-13	FAS/CASP8/CXCL8/TNF/PTGS2/CTNNB1/CCND1/MYC/GSK3B/IL6/CASP3/FASLG/MAPK1/EGFR/RB1/SRC/TP53/CASP9/CDKN1A/AKT1/PIK3R1/NFKB1/IL1B/NFKBIA/BAX
hsa05210	Colorectal cancer	17/139	86/8111	5.14E-14	9.43E-13	2.90E-13	BCL2/CTNNB1/CCND1/TGFB1/MYC/GSK3B/CASP3/MAPK1/EGFR/FOS/TP53/CASP9/CDKN1A/AKT1/PIK3R1/JUN/BAX
hsa05219	Bladder cancer	13/139	41/8111	7.29E-14	1.25E-12	3.84E-13	MMP2/CXCL8/CCND1/MYC/MMP1/MAPK1/EGFR/ERBB2/RB1/SRC/TP53/CDKN1A/MMP9
hsa04932	Non-alcoholic fatty liver disease	21/139	155/8111	1.22E-13	1.95E-12	6.00E-13	LEP/FAS/CASP8/PPARG/CXCL8/TNF/IL1A/CYP2E1/TGFB1/GSK3B/IL6/CASP3/FASLG/INS/FOS/AKT1/PIK3R1/NFKB1/IL1B/JUN/BAX
hsa04010	MAPK signaling pathway	27/139	294/8111	4.51E-13	6.46E-12	1.98E-12	IGF1/MET/FAS/CACNA1B/MAPT/TNF/IL1A/TGFB1/MYC/CASP3/CACNA1C/FASLG/KDR/INS/MAPK1/IGF2/EGFR/ERBB2/CACNA1A/FOS/FGF2/CACNA1S/TP53/AKT1/NFKB1/IL1B/JUN
hsa05224	Breast cancer	20/139	147/8111	4.52E-13	6.46E-12	1.98E-12	IGF1/CTNNB1/CCND1/ESR1/MYC/GSK3B/MAPK1/EGFR/ERBB2/FOS/FGF2/RB1/PGR/TP53/CDKN1A/AKT1/PIK3R1/JUN/NOTCH1/BAX
hsa05223	Non-small cell lung cancer	15/139	72/8111	7.56E-13	1.02E-11	3.14E-12	MET/ALK/RET/CCND1/MAPK1/EGFR/ERBB2/RB1/TP53/CASP9/CDKN1A/JAK3/AKT1/PIK3R1/BAX
hsa04668	TNF signaling pathway	17/139	112/8111	4.77E-12	6.13E-11	1.88E-11	IRF1/FAS/CASP8/TNF/PTGS2/IL6/CASP3/MAPK1/FOS/ICAM1/AKT1/PIK3R1/NFKB1/IL1B/NFKBIA/JUN/MMP9
hsa05207	Chemical carcinogenesis - receptor activation	22/139	212/8111	7.52E-12	9.20E-11	2.83E-11	BCL2/CYP1A1/CACNA1B/CCND1/ESR1/MYC/HSP90AA1/CACNA1C/MAPK1/EGFR/CACNA1A/FOS/FGF2/CACNA1S/RB1/SRC/PGR/AKT1/PIK3R1/NFKB1/JUN/CYP3A4
hsa05164	Influenza A	20/139	172/8111	8.99E-12	1.05E-10	3.23E-11	FAS/CASP8/CXCL8/TNF/IL1A/STAT1/IL6/CASP3/FASLG/MAPK1/SLC25A4/IFNG/ICAM1/CASP9/AKT1/PIK3R1/NFKB1/IL1B/NFKBIA/BAX
hsa05213	Endometrial cancer	13/139	58/8111	1.02E-11	1.14E-10	3.50E-11	CTNNB1/CCND1/MYC/GSK3B/MAPK1/EGFR/ERBB2/TP53/CASP9/CDKN1A/AKT1/PIK3R1/BAX
hsa04210	Apoptosis	18/139	136/8111	1.18E-11	1.26E-10	3.88E-11	FAS/BCL2/CASP8/TNF/CTSD/CASP3/PARP1/FASLG/MAPK1/FOS/TP53/CASP9/AKT1/PIK3R1/NFKB1/NFKBIA/JUN/BAX

hsa04625	C-type lectin receptor signaling pathway	16/139	104/8111	1.70E-11	1.75E-10	5.38E-11	IRF1/CASP8/TNF/PTGS2/STAT1/IL6/IL10/MAPK1/IL2/SRC/AKT1/PIK3R1/NFKB1/IL1B/NFKBIA/JUN
hsa05169	Epstein-Barr virus infection	21/139	202/8111	2.28E-11	2.26E-10	6.93E-11	FAS/BCL2/CASP8/TNF/CCND1/MYC/STAT1/IL6/CASP3/ICAM1/RB1/TP53/CASP9/CDKN1A/JAK3/AKT1/PIK3R1/NFKB1/NFKBIA/JUN/BAX
hsa05212	Pancreatic cancer	14/139	76/8111	2.70E-11	2.57E-10	7.90E-11	CCND1/TGFB1/STAT1/MAPK1/EGFR/ERBB2/RB1/TP53/CASP9/CDKN1A/AKT1/PIK3R1/NFKB1/BAX
hsa05222	Small cell lung cancer	15/139	92/8111	3.21E-11	2.89E-10	8.86E-11	NOS2/BCL2/PTGS2/CCND1/MYC/CASP3/RB1/TP53/CASP9/CDKN1A/AKT1/PIK3R1/NFKB1/NFKBIA/BAX
hsa05140	Leishmaniasis	14/139	77/8111	3.26E-11	2.89E-10	8.86E-11	NOS2/TNF/IL1A/PTGS2/TGFB1/STAT1/IL10/MAPK1/IFNG/FOS/NFKB1/IL1B/NFKBIA/JUN
hsa01521	EGFR tyrosine kinase inhibitor resistance	14/139	79/8111	4.68E-11	4.01E-10	1.23E-10	IGF1/MET/BCL2/GSK3B/IL6/KDR/MAPK1/EGFR/ERBB2/FGF2/SRC/AKT1/PIK3R1/BAX
hsa05226	Gastric cancer	18/139	149/8111	5.63E-11	4.67E-10	1.43E-10	MET/BCL2/ABCB1/CTNNB1/CCND1/TGFB1/MYC/GSK3B/MAPK1/EGFR/ERBB2/FGF2/RB1/TP53/CDKN1A/AKT1/PIK3R1/BAX
hsa05010	Alzheimer disease	27/139	369/8111	9.51E-11	7.64E-10	2.35E-10	NOS2/NOS1/APOE/FAS/CASP8/MAPT/TNF/IL1A/GRIN2B/PTGS2/CTNNB1/SNCA/GSK3B/IL6/CASP3/CACNA1C/INS/MAPK1/SLC25A4/GRIN1/CACNA1S/CASP9/AKT1/PIK3R1/NFKB1/IL1B/CHRM3
hsa05166	Human T-cell leukemia virus 1 infection	21/139	219/8111	1.07E-10	8.30E-10	2.55E-10	TNF/CCND1/TGFB1/MYC/IL6/MAPK1/SLC25A4/IL2/FOS/ICAM1/RB1/TCF3/TP53/CDKN1A/JAK3/AKT1/PIK3R1/NFKB1/NFKBIA/JUN/BAX
hsa05152	Tuberculosis	19/139	180/8111	1.71E-10	1.29E-09	3.98E-10	NOS2/BCL2/CASP8/TNF/IL1A/CTSD/TGFB1/STAT1/IL6/CASP3/IL10/MAPK1/IFNG/SRC/CASP9/AKT1/NFKB1/IL1B/BAX
hsa01524	Platinum drug resistance	13/139	73/8111	2.24E-10	1.64E-09	5.05E-10	FAS/BCL2/CASP8/CASP3/FASLG/MAPK1/ERBB2/TP53/CASP9/CDKN1A/AKT1/PIK3R1/BAX
hsa05133	Pertussis	13/139	76/8111	3.80E-10	2.64E-09	8.10E-10	NOS2/IRF1/CXCL8/TNF/IL1A/IL6/CASP3/IL10/MAPK1/FOS/NFKB1/IL1B/JUN
hsa05220	Chronic myeloid leukemia	13/139	76/8111	3.80E-10	2.64E-09	8.10E-10	CCND1/TGFB1/MYC/MAPK1/ABL1/RB1/TP53/CDKN1A/AKT1/PIK3R1/NFKB1/NFKBIA/BAX
hsa05225	Hepatocellular carcinoma	18/139	168/8111	4.21E-10	2.85E-09	8.75E-10	MET/NFE2L2/CTNNB1/CCND1/TGFB1/MYC/GSK3B/HMOX1/MAPK1/IGF2/EGFR/NQO1/RB1/TP53/CDKN1A/AKT1/PIK3R1/BAX
hsa04926	Relaxin signaling pathway	16/139	129/8111	4.76E-10	3.14E-09	9.64E-10	MMP2/NOS2/NOS1/COL1A1/TGFB1/MMP1/MAPK1/EGFR/FOS/SRC/AKT1/PIK3R1/NFKB1/NFKBIA/JUN/MMP9
hsa05321	Inflammatory bowel disease	12/139	65/8111	7.38E-10	4.74E-09	1.46E-09	TNF/IL1A/TGFB1/IL13/STAT1/IL6/IL10/IL2/IFNG/NFKB1/IL1B/JUN
hsa04151	PI3K-Akt signaling pathway	25/139	354/8111	1.10E-09	6.92E-09	2.12E-09	IGF1/MET/COL1A1/BCL2/CCND1/MYC/GSK3B/IL6/HSP90AA1/FASLG/KDR/INS/MAPK1/IGF2/EGFR/IL2/ERBB2/FGF2/TP53/CASP9/CDKN1A/JAK3/AKT1/PIK3R1/NFKB1
hsa05022	Pathways of neurodegeneration - multiple diseases	29/139	476/8111	1.36E-09	8.35E-09	2.56E-09	NOS2/NOS1/FAS/BCL2/CASP8/CACNA1B/MAPT/TNF/IL1A/GRIN2B/PTGS2/CTNNB1/SNCA/GSK3B/IL6/CASP3/CACNA1C/FASLG/MAPK1/SLC25A4/SOD1/GRIN1/CACNA1S/CASP9/CAT/NFKB1/IL13/BAX

hsa04919	Thyroid hormone signaling pathway	15/139	121/8111	1.73E-09	1.04E-08	3.19E-09	THRB/CTNNB1/CCND1/ESR1/MYC/GSK3B/STAT1/MAPK1/SRC/THRA/TP53/CASP9/AKT1/PIK3R1/NOTCH1
hsa04917	Prolactin signaling pathway	12/139	70/8111	1.81E-09	1.06E-08	3.25E-09	IRF1/CCND1/ESR1/GSK3B/STAT1/INS/MAPK1/FOS/SRC/AKT1/PIK3R1/NFKB1
hsa05218	Melanoma	12/139	72/8111	2.54E-09	1.45E-08	4.46E-09	IGF1/MET/CCND1/MAPK1/EGFR/FGF2/RB1/TP53/CDKN1A/AKT1/PIK3R1/BAX
hsa04066	HIF-1 signaling pathway	14/139	109/8111	3.90E-09	2.18E-08	6.69E-09	IGF1/NOS2/BCL2/IL6/HMOX1/INS/MAPK1/EGFR/ERBB2/IFNG/CDKN1A/AKT1/PIK3R1/NFKB1
hsa05145	Toxoplasmosis	14/139	112/8111	5.59E-09	3.01E-08	9.24E-09	NOS2/BCL2/CASP8/TNF/TGFB1/STAT1/CASP3/IL10/MAPK1/IFNG/CASP9/AKT1/NFKB1/NFKBIA
hsa05130	Pathogenic Escherichia coli infection	18/139	197/8111	5.62E-09	3.01E-08	9.24E-09	FAS/CASP8/CXCL8/TNF/F2/IL6/CASP3/FASLG/MAPK1/FOS/ABL1/SRC/CASP9/NFKB1/IL1B/NFKBIA/JUN/BAX
hsa05208	Chemical carcinogenesis - reactive oxygen species	19/139	223/8111	6.57E-09	3.44E-08	1.06E-08	MET/CYP1A1/NFE2L2/CYP2E1/HMOX1/MAPK1/EGFR/SLC25A4/NQO1/SOD1/FOS/ABL1/SRC/CAT/AKT1/PIK3R1/NFKB1/NFKBIA/JUN
hsa05216	Thyroid cancer	9/139	37/8111	8.14E-09	4.18E-08	1.29E-08	PPARG/RET/CTNNB1/CCND1/MYC/MAPK1/TP53/CDKN1A/BAX
hsa05135	Yersinia infection	15/139	137/8111	9.88E-09	4.98E-08	1.53E-08	CXCL8/TNF/GSK3B/IL6/IL10/MAPK1/IL2/FOS/SRC/AKT1/PIK3R1/NFKB1/IL1B/NFKBIA/JUN
hsa04620	Toll-like receptor signaling pathway	13/139	104/8111	2.02E-08	9.99E-08	3.07E-08	CASP8/CXCL8/TNF/STAT1/IL6/MAPK1/FOS/AKT1/PIK3R1/NFKB1/IL1B/NFKBIA/JUN
hsa04380	Osteoclast differentiation	14/139	128/8111	3.21E-08	1.53E-07	4.69E-08	PPARG/TNF/IL1A/TGFB1/STAT1/MAPK1/IFNG/FOS/AKT1/PIK3R1/NFKB1/IL1B/NFKBIA/JUN
hsa04659	Th17 cell differentiation	13/139	108/8111	3.21E-08	1.53E-07	4.69E-08	TGFB1/STAT1/IL6/HSP90AA1/MAPK1/IL2/IFNG/FOS/JAK3/NFKB1/IL1B/NFKBIA/JUN
hsa04115	p53 signaling pathway	11/139	73/8111	3.62E-08	1.69E-07	5.19E-08	IGF1/FAS/BCL2/CASP8/CCND1/CASP3/TP53/CASP9/CDKN1A/IGFBP3/BAX
hsa05165	Human papillomavirus infection	22/139	331/8111	3.78E-08	1.73E-07	5.32E-08	IRF1/COL1A1/FAS/CASP8/TNF/PTGS2/CTNNB1/CCND1/GSK3B/STAT1/CASP3/FASLG/MAPK1/EGFR/RB1/TP53/CDKN1A/AKT1/PIK3R1/NFKB1/NOTCH1/BAX
hsa04510	Focal adhesion	17/139	201/8111	4.89E-08	2.21E-07	6.78E-08	IGF1/MET/COL1A1/BCL2/CTNNB1/CCND1/GSK3B/KDR/MAPK1/EGFR/MYLK/ERBB2/CAV1/SRC/AKT1/PIK3R1/JUN
hsa04218	Cellular senescence	15/139	156/8111	5.81E-08	2.57E-07	7.91E-08	CXCL8/IL1A/CCND1/TGFB1/MYC/IL6/MAPK1/SLC25A4/RB1/TP53/CDKN1A/AKT1/PIK3R1/NFKB1/IGFBP3
hsa04726	Serotonergic synapse	13/139	115/8111	6.87E-08	2.99E-07	9.20E-08	SLC6A4/HTR3A/CACNA1B/PTGS2/CASP3/CACNA1C/HTR1A/MAOB/MAPK1/CACNA1A/CACNA1S/PTGS1/HTR2A
hsa04915	Estrogen signaling pathway	14/139	138/8111	8.39E-08	3.60E-07	1.10E-07	MMP2/BCL2/ESR1/CTSD/HSP90AA1/MAPK1/EGFR/FOS/SRC/PGR/AKT1/PIK3R1/JUN/MMP9

hsa04630	JAK-STAT signaling pathway	15/139	162/8111	9.63E-08	4.06E-07	1.25E-07	LEP/BCL2/CCND1/MYC/IL13/STAT1/IL6/IL10/EGFR/IL2/IFNG/CDKN1A/JAK3/AKT1/PIK3R1
hsa05144	Malaria	9/139	50/8111	1.36E-07	5.63E-07	1.73E-07	MET/CXCL8/TNF/TGFB1/IL6/IL10/IFNG/ICAM1/IL1B
hsa05146	Amoebiasis	12/139	102/8111	1.44E-07	5.88E-07	1.81E-07	NOS2/COL1A1/CXCL8/TNF/TGFB1/IL6/CASP3/IL10/IFNG/PIK3R1/NFKB1/IL1B
hsa05143	African trypanosomiasis	8/139	37/8111	1.54E-07	6.20E-07	1.90E-07	FAS/TNF/IL6/FASLG/IL10/IFNG/ICAM1/IL1B
hsa05020	Prion disease	19/139	273/8111	1.73E-07	6.83E-07	2.10E-07	CACNA1B/TNF/IL1A/GRIN2B/GSK3B/IL6/CASP3/CACNA1C/MAPK1/SLC25A4/SOD1/GRIN1/CAV1/CACNA1S/CASP9/PIK3R1/IL1B/NOTCH1/BAX
hsa04660	T cell receptor signaling pathway	12/139	104/8111	1.79E-07	6.98E-07	2.14E-07	TNF/GSK3B/IL10/MAPK1/IL2/IFNG/FOS/AKT1/PIK3R1/NFKB1/NFKBIA/JUN
hsa04012	ErbB signaling pathway	11/139	85/8111	1.82E-07	6.98E-07	2.14E-07	MYC/GSK3B/MAPK1/EGFR/ERBB2/ABL1/SRC/CDKN1A/AKT1/PIK3R1/JUN
hsa05206	MicroRNAs in cancer	20/139	310/8111	2.73E-07	1.03E-06	3.17E-07	PLAU/MET/BCL2/ABCB1/PTGS2/CCND1/MYC/CASP3/HMOX1/MAPK1/EGFR/ERBB2/ABL1/TP53/CDKN1A/PIK3R1/NFKB1/NOTCH1/CYP24A1/MMP9
hsa05235	PD-L1 expression and PD-1 checkpoint pathway in cancer	11/139	89/8111	2.94E-07	1.09E-06	3.36E-07	ALK/STAT1/MAPK1/EGFR/IFNG/FOS/AKT1/PIK3R1/NFKB1/NFKBIA/JUN
hsa04068	FoxO signaling pathway	13/139	131/8111	3.23E-07	1.19E-06	3.65E-07	IGF1/CCND1/TGFB1/IL6/FASLG/INS/IL10/MAPK1/EGFR/CDKN1A/CAT/AKT1/PIK3R1
hsa04014	Ras signaling pathway	17/139	232/8111	3.93E-07	1.42E-06	4.37E-07	IGF1/MET/PLA2G2A/PLA2G6/GRIN2B/FASLG/KDR/INS/MAPK1/IGF2/EGFR/GRIN1/FGF2/ABL1/AKT1/PIK3R1/NFKB1
hsa04658	Th1 and Th2 cell differentiation	11/139	92/8111	4.14E-07	1.48E-06	4.54E-07	IL13/STAT1/MAPK1/IL2/IFNG/FOS/JAK3/NFKB1/NFKBIA/JUN/NOTCH1
hsa05332	Graft-versus-host disease	8/139	42/8111	4.39E-07	1.53E-06	4.70E-07	FAS/TNF/IL1A/IL6/FASLG/IL2/IFNG/IL1B
hsa05134	Legionellosis	9/139	57/8111	4.40E-07	1.53E-06	4.70E-07	CASP8/CXCL8/TNF/IL6/CASP3/CASP9/NFKB1/IL1B/NFKBIA
hsa04725	Cholinergic synapse	12/139	113/8111	4.49E-07	1.54E-06	4.73E-07	BCL2/CACNA1B/ACHE/KCNQ1/CACNA1C/MAPK1/CACNA1A/FOS/CACNA1S/AKT1/PIK3R1/CHRM3
hsa05323	Rheumatoid arthritis	11/139	93/8111	4.63E-07	1.56E-06	4.81E-07	CXCL8/TNF/IL1A/TGFB1/MMP1/IL6/IFNG/FOS/ICAM1/IL1B/JUN
hsa04217	Necroptosis	14/139	159/8111	4.93E-07	1.64E-06	5.05E-07	FAS/BCL2/CASP8/TNF/IL1A/STAT1/HSP90AA1/PARP1/FASLG/SLC25A4/IFNG/JAK3/IL1B/BAX
hsa05214	Glioma	10/139	75/8111	5.07E-07	1.67E-06	5.13E-07	IGF1/CCND1/MAPK1/EGFR/RB1/TP53/CDKN1A/AKT1/PIK3R1/BAX

hsa04940	Type I diabetes mellitus	8/139	43/8111	5.32E-07	1.73E-06	5.31E-07	FAS/TNF/IL1A/FASLG/INS/IL2/IFNG/IL1B
hsa04020	Calcium signaling pathway	17/139	240/8111	6.35E-07	2.04E-06	6.27E-07	NOS2/MET/NOS1/CACNA1B/RET/CACNA1C/KDR/EGFR/SLC25A4/MYLK/ERBB2/CACNA1A/GRIN1/FGF2/CACNA1S/CHRM3/HTR2A
hsa04722	Neurotrophin signaling pathway	12/139	119/8111	7.90E-07	2.51E-06	7.70E-07	BCL2/GSK3B/FASLG/MAPK1/ABL1/TP53/AKT1/PIK3R1/NFKB1/NFKBIA/JUN/BAX
hsa05202	Transcriptional misregulation in cancer	15/139	192/8111	8.85E-07	2.77E-06	8.52E-07	IGF1/PLAU/MET/PPARG/CXCL8/MPO/MYC/IL6/TCF3/TP53/CDKN1A/NFKB1/IGFBP3/BAX/MMP9
hsa05132	Salmonella infection	17/139	249/8111	1.07E-06	3.30E-06	1.01E-06	BCL2/CASP8/CXCL8/TNF/CTNNB1/MYC/IL6/HSP90AA1/CASP3/MAPK1/FOS/AKT1/NFKB1/IL1B/NFKBIA/JUN/BAX
hsa05230	Central carbon metabolism in cancer	9/139	70/8111	2.64E-06	8.06E-06	2.48E-06	MET/RET/MYC/MAPK1/EGFR/ERBB2/TP53/AKT1/PIK3R1
hsa05170	Human immunodeficiency virus 1 infection	15/139	212/8111	3.09E-06	9.33E-06	2.87E-06	FAS/BCL2/CASP8/TNF/CASP3/FASLG/MAPK1/FOS/CASP9/AKT1/PIK3R1/NFKB1/NFKBIA/JUN/BAX
hsa05033	Nicotine addiction	7/139	40/8111	4.34E-06	1.30E-05	3.98E-06	CACNA1B/GRIN2B/GABRG3/CACNA1A/GRIN1/GABRD/GABRA3
hsa04024	cAMP signaling pathway	15/139	219/8111	4.61E-06	1.36E-05	4.18E-06	SOX9/DRD2/GRIN2B/CACNA1C/HTR1A/MAPK1/GRIN1/FOS/CACNA1S/CFTR/AKT1/PIK3R1/NFKB1/NFKBIA/JUN
hsa04080	Neuroactive ligand-receptor interaction	19/139	341/8111	5.03E-06	1.47E-05	4.51E-06	LEP/THRB/ADRA2A/OPRK1/DRD2/GRIN2B/F2/GABRG3/AVPR2/TRPV1/HTR1A/GRIN1/GABRD/DRD4/THRA/CHRM3/HTR2A/GABRA3/AVP
hsa04550	Signaling pathways regulating pluripotency of stem cells	12/139	143/8111	5.57E-06	1.61E-05	4.94E-06	IGF1/CTNNB1/MYC/GSK3B/MAPK1/NODAL/FGF2/TCF3/ACVR1B/JAK3/AKT1/PIK3R1
hsa05171	Coronavirus disease - COVID-19	15/139	232/8111	9.31E-06	2.66E-05	8.17E-06	CXCL8/TNF/F2/MMP1/STAT1/IL6/MAPK1/EGFR/IL2/FOS/PIK3R1/NFKB1/IL1B/NFKBIA/JUN
hsa05203	Viral carcinogenesis	14/139	204/8111	9.55E-06	2.70E-05	8.28E-06	CASP8/CCND1/CASP3/MAPK1/RB1/SRC/TP53/CDKN1A/JAK3/PIK3R1/NFKB1/NFKBIA/JUN/BAX
hsa04064	NF-kappa B signaling pathway	10/139	104/8111	1.05E-05	2.92E-05	8.98E-06	PLAU/BCL2/CXCL8/TNF/PTGS2/PARP1/ICAM1/NFKB1/IL1B/NFKBIA
hsa04930	Type II diabetes mellitus	7/139	46/8111	1.14E-05	3.16E-05	9.70E-06	CACNA1B/TNF/CACNA1C/INS/MAPK1/CACNA1A/PIK3R1
hsa04015	Rap1 signaling pathway	14/139	210/8111	1.33E-05	3.64E-05	1.12E-05	IGF1/MET/DRD2/GRIN2B/CTNNB1/KDR/INS/MAPK1/EGFR/GRIN1/FGF2/SRC/AKT1/PIK3R1
hsa04211	Longevity regulating pathway	9/139	89/8111	1.96E-05	5.27E-05	1.62E-05	IGF1/PPARG/INS/TP53/CAT/AKT1/PIK3R1/NFKB1/BAX
hsa05131	Shigellosis	15/139	247/8111	1.97E-05	5.27E-05	1.62E-05	BCL2/CXCL8/TNF/GSK3B/MAPK1/EGFR/SRC/TP53/AKT1/PIK3R1/NFKB1/IL1B/NFKBIA/JUN/BAX

hsa05120	Epithelial cell signaling in Helicobacter pylori infection	8/139	70/8111	2.34E-05	6.21E-05	1.91E-05	MET/CXCL8/CASP3/EGFR/SRC/NFKB1/NFKBIA/JUN
hsa04935	Growth hormone synthesis, secretion and action	10/139	119/8111	3.43E-05	9.00E-05	2.76E-05	IGF1/GSK3B/STAT1/CACNA1C/MAPK1/FOS/CACNA1S/AKT1/PIK3R1/IGFBP3
hsa05330	Allograft rejection	6/139	38/8111	4.00E-05	0.000104	3.19E-05	FAS/TNF/FASLG/IL10/IL2/IFNG
hsa04370	VEGF signaling pathway	7/139	59/8111	6.05E-05	0.000156	4.78E-05	PTGS2/KDR/MAPK1/SRC/CASP9/AKT1/PIK3R1
hsa04921	Oxytocin signaling pathway	11/139	154/8111	6.26E-05	0.000159	4.89E-05	PTGS2/CCND1/CACNA1C/MAPK1/EGFR/MYLK/FOS/CACNA1S/SRC/CDKN1A/JUN
hsa05416	Viral myocarditis	7/139	60/8111	6.75E-05	0.00017	5.23E-05	CASP8/CCND1/CASP3/ICAM1/CAV1/ABL1/CASP9
hsa04621	NOD-like receptor signaling pathway	12/139	185/8111	7.40E-05	0.000184	5.66E-05	BCL2/CASP8/CXCL8/TNF/STAT1/IL6/HSP90AA1/MAPK1/NFKB1/IL1B/NFKBIA/JUN
hsa04662	B cell receptor signaling pathway	8/139	82/8111	7.45E-05	0.000184	5.66E-05	GSK3B/MAPK1/FOS/AKT1/PIK3R1/NFKB1/NFKBIA/JUN
hsa04728	Dopaminergic synapse	10/139	132/8111	8.33E-05	0.000204	6.26E-05	CACNA1B/DRD2/GRIN2B/GSK3B/CACNA1C/MAOB/CACNA1A/FOS/DRD4/AKT1
hsa04727	GABAergic synapse	8/139	89/8111	0.000133	0.000324	9.94E-05	CACNA1B/GABRG3/CACNA1C/CACNA1A/GABRD/CACNA1S/SRC/GABRA3
hsa05221	Acute myeloid leukemia	7/139	67/8111	0.000138	0.000331	0.000102	MPO/CCND1/MYC/MAPK1/AKT1/PIK3R1/NFKB1
hsa04060	Cytokine-cytokine receptor interaction	15/139	295/8111	0.000151	0.000358	0.00011	LEP/FAS/CXCL8/TNF/IL1A/TGFB1/IL13/IL6/FASLG/IL10/NODAL/IL2/IFNG/ACVR1B/IL1B
hsa05211	Renal cell carcinoma	7/139	69/8111	0.000166	0.000391	0.00012	MET/TGFB1/MAPK1/CDKN1A/AKT1/PIK3R1/JUN
hsa05030	Cocaine addiction	6/139	49/8111	0.000173	0.000405	0.000124	DRD2/GRIN2B/MAOB/GRIN1/NFKB1/JUN
hsa05415	Diabetic cardiomyopathy	12/139	203/8111	0.000179	0.000415	0.000127	MMP2/COL1A1/CTSD/TGFB1/GSK3B/PARP1/INS/SLC25A4/AKT1/PIK3R1/NFKB1/MMP9
hsa04215	Apoptosis - multiple species	5/139	32/8111	0.000191	0.000438	0.000135	BCL2/CASP8/CASP3/CASP9/BAX
hsa04071	Sphingolipid signaling pathway	9/139	119/8111	0.000193	0.000439	0.000135	BCL2/TNF/CTSD/MAPK1/TP53/AKT1/PIK3R1/NFKB1/BAX
hsa04520	Adherens junction	7/139	71/8111	0.000199	0.000448	0.000138	MET/CTNNB1/MAPK1/EGFR/ERBB2/SRC/SNAI2

hsa04152	AMPK signaling pathway	9/139	120/8111	0.000205	0.000459	0.000141	IGF1/LEP/PPARG/CCND1/HMGCR/INS/CFTR/AKT1/PIK3R1
hsa04750	Inflammatory mediator regulation of TRP channels	8/139	98/8111	0.000261	0.000578	0.000178	IGF1/PLA2G6/TRPA1/TRPV1/SRC/PIK3R1/IL1B/HTR2A
hsa04611	Platelet activation	9/139	124/8111	0.000263	0.000578	0.000178	COL1A1/F2/P2RY12/MAPK1/MYLK/SRC/AKT1/PIK3R1/PTGS1
hsa04931	Insulin resistance	8/139	108/8111	0.000507	0.001103	0.000339	TNF/GSK3B/IL6/INS/AKT1/PIK3R1/NFKB1/NFKBIA
hsa00590	Arachidonic acid metabolism	6/139	61/8111	0.000582	0.001257	0.000386	PLA2G2A/PLA2G6/PTGS2/CYP2E1/GGT1/PTGS1
hsa04213	Longevity regulating pathway - multiple species	6/139	62/8111	0.000635	0.001361	0.000418	IGF1/INS/SOD1/CAT/AKT1/PIK3R1
hsa04072	Phospholipase D signaling pathway	9/139	148/8111	0.000961	0.002024	0.000622	CXCL8/F2/AVPR2/INS/MAPK1/EGFR/AKT1/PIK3R1/AVP
hsa04723	Retrograde endocannabinoid signaling	9/139	148/8111	0.000961	0.002024	0.000622	CACNA1B/PTGS2/GABRG3/CACNA1C/MAPK1/CACNA1A/GABRD/CACNA1S/GABRA3
hsa04912	GnRH signaling pathway	7/139	93/8111	0.001037	0.002166	0.000665	MMP2/CACNA1C/MAPK1/EGFR/CACNA1S/SRC/JUN
hsa04350	TGF-beta signaling pathway	7/139	94/8111	0.001104	0.002289	0.000703	TNF/TGFB1/MYC/MAPK1/NODAL/IFNG/ACVR1B
hsa05031	Amphetamine addiction	6/139	69/8111	0.001124	0.00231	0.00071	GRIN2B/CACNA1C/MAOB/GRIN1/FOS/JUN
hsa04110	Cell cycle	8/139	124/8111	0.00126	0.00257	0.000789	CCND1/TGFB1/MYC/GSK3B/ABL1/RB1/TP53/CDKN1A
hsa04934	Cushing syndrome	9/139	155/8111	0.001332	0.002695	0.000828	CTNNB1/CCND1/GSK3B/CACNA1C/MAPK1/EGFR/CACNA1S/RB1/CDKN1A
hsa00591	Linoleic acid metabolism	4/139	29/8111	0.001406	0.002824	0.000867	PLA2G2A/PLA2G6/CYP2E1/CYP3A4
hsa04914	Progesterone-mediated oocyte maturation	7/139	100/8111	0.001586	0.00316	0.000971	IGF1/HSP90AA1/INS/MAPK1/PGR/AKT1/PIK3R1
hsa04062	Chemokine signaling pathway	10/139	192/8111	0.001653	0.003267	0.001004	CXCL8/GSK3B/STAT1/MAPK1/SRC/JAK3/AKT1/PIK3R1/NFKB1/NFKBIA
hsa04650	Natural killer cell mediated cytotoxicity	8/139	131/8111	0.001791	0.003514	0.00108	FAS/TNF/CASP3/FASLG/MAPK1/IFNG/ICAM1/PIK3R1
hsa01523	Antifolate resistance	4/139	31/8111	0.001814	0.003533	0.001085	TNF/IL6/NFKB1/IL1B

hsa05168	Herpes simplex virus 1 infection	18/139	498/8111	0.002032	0.003911	0.001201	FAS/BCL2/CASP8/TNF/STAT1/IL6/CASP3/FASLG/IFNG/SRC/TP53/CASP9/AKT1/PIK3R1/NFKB1/IL1B/NFKBIA/BAX
hsa05320	Autoimmune thyroid disease	5/139	53/8111	0.002039	0.003911	0.001201	FAS/FASLG/IL10/IL2/TPO
hsa04923	Regulation of lipolysis in adipocytes	5/139	57/8111	0.002817	0.005363	0.001648	PTGS2/INS/AKT1/PIK3R1/PTGS1
hsa04742	Taste transduction	6/139	86/8111	0.003477	0.006571	0.002019	HTR3A/CACNA1C/HTR1A/CACNA1A/CHRM3/GABRA3
hsa04960	Aldosterone-regulated sodium reabsorption	4/139	37/8111	0.003518	0.006599	0.002027	IGF1/INS/MAPK1/PIK3R1
hsa05410	Hypertrophic cardiomyopathy	6/139	90/8111	0.004356	0.008058	0.002475	IGF1/TNF/TGFB1/IL6/CACNA1C/CACNA1S
hsa05217	Basal cell carcinoma	5/139	63/8111	0.004358	0.008058	0.002475	CTNNB1/GSK3B/TP53/CDKN1A/BAX
hsa04929	GnRH secretion	5/139	64/8111	0.004664	0.008561	0.00263	CACNA1C/MAPK1/CACNA1S/AKT1/PIK3R1
hsa04664	Fc epsilon RI signaling pathway	5/139	68/8111	0.006039	0.011007	0.003381	TNF/IL13/MAPK1/AKT1/PIK3R1
hsa04713	Circadian entrainment	6/139	97/8111	0.006274	0.011354	0.003488	NOS1/GRIN2B/CACNA1C/MAPK1/GRIN1/FOS
hsa04920	Adipocytokine signaling pathway	5/139	69/8111	0.006423	0.011543	0.003546	LEP/TNF/AKT1/NFKB1/NFKBIA
hsa05231	Choline metabolism in cancer	6/139	98/8111	0.006591	0.011763	0.003613	MAPK1/EGFR/FOS/AKT1/PIK3R1/JUN
hsa04622	RIG-I-like receptor signaling pathway	5/139	70/8111	0.006824	0.012095	0.003715	CASP8/CXCL8/TNF/NFKB1/NFKBIA
hsa04270	Vascular smooth muscle contraction	7/139	133/8111	0.007741	0.013627	0.004186	PLA2G2A/PLA2G6/CACNA1C/MAPK1/MYLK/CACNA1S/AVP
hsa04022	cGMP-PKG signaling pathway	8/139	167/8111	0.007848	0.013721	0.004215	ADRA2A/CACNA1C/INS/MAPK1/SLC25A4/MYLK/CACNA1S/AKT1
hsa04928	Parathyroid hormone synthesis, secretion and action	6/139	106/8111	0.009562	0.016604	0.0051	BCL2/MAPK1/EGFR/FOS/CDKN1A/CYP24A1
hsa04672	Intestinal immune network for IgA production	4/139	49/8111	0.009638	0.017354	0.005107	TGFB1/IL6/IL10/IL2
hsa05100	Bacterial invasion of epithelial cells	5/139	77/8111	0.010132	0.017359	0.005333	MET/CTNNB1/CAV1/SRC/PIK3R1

hsa05012	Parkinson disease	10/139	249/8111	0.010327	0.017576	0.005399	MAPT/NFE2L2/DRD2/SNCA/CASP3/MAOB/SLC25A4/TP53/CASP9/BAX
hsa00330	Arginine and proline metabolism	4/139	51/8111	0.011074	0.018601	0.005714	NOS2/NOS1/MAOB/GATM
hsa04913	Ovarian steroidogenesis	4/139	51/8111	0.011074	0.018601	0.005714	IGF1/CYP1A1/PTGS2/INS
hsa04810	Regulation of actin cytoskeleton	9/139	218/8111	0.012452	0.020781	0.006384	F2/INS/MAPK1/EGFR/MYLK/FGF2/SRC/PIK3R1/CHRM3
hsa04724	Glutamatergic synapse	6/139	114/8111	0.013385	0.022193	0.006817	GRIN2B/SHANK3/CACNA1C/MAPK1/CACNA1A/GRIN1
hsa04261	Adrenergic signaling in cardiomyocytes	7/139	150/8111	0.014421	0.023758	0.007298	BCL2/KCNQ1/CACNA1C/MAPK1/CACNA1S/AKT1/SCN5A
hsa05310	Asthma	3/139	31/8111	0.015594	0.025526	0.007841	TNF/IL13/IL10
hsa05016	Huntington disease	11/139	306/8111	0.015789	0.025683	0.007889	CASP8/PPARG/CACNA1B/GRIN2B/CASP3/SLC25A4/SOD1/GRIN1/TP53/CASP9/BAX
hsa04150	mTOR signaling pathway	7/139	155/8111	0.016996	0.027471	0.008439	IGF1/TNF/GSK3B/INS/MAPK1/AKT1/PIK3R1
hsa04540	Gap junction	5/139	88/8111	0.017317	0.027816	0.008545	DRD2/MAPK1/EGFR/SRC/HTR2A
hsa04730	Long-term depression	4/139	60/8111	0.019212	0.030668	0.009421	IGF1/NOS1/MAPK1/CACNA1A
hsa05032	Morphine addiction	5/139	91/8111	0.019743	0.031321	0.009622	CACNA1B/GABRG3/CACNA1A/GABRD/GABRA3
hsa05014	Amyotrophic lateral sclerosis	12/139	365/8111	0.022491	0.035368	0.010865	NOS2/NOS1/BCL2/TNF/GRIN2B/CASP3/SOD1/GRIN1/TP53/CASP9/CAT/BAX
hsa04623	Cytosolic DNA-sensing pathway	4/139	63/8111	0.02257	0.035368	0.010865	IL6/NFKB1/IL1B/NFKBIA
hsa00350	Tyrosine metabolism	3/139	36/8111	0.023286	0.036269	0.011141	ADH1C/MAOB/TPO
hsa05414	Dilated cardiomyopathy	5/139	96/8111	0.024264	0.037566	0.01154	IGF1/TNF/TGFB1/CACNA1C/CACNA1S
hsa04720	Long-term potentiation	4/139	67/8111	0.027572	0.04243	0.013034	GRIN2B/CACNA1C/MAPK1/GRIN1
hsa04061	Viral protein interaction with cytokine and cytokine receptor	5/139	100/8111	0.028326	0.043332	0.013311	CXCL8/TNF/IL6/IL10/IL2

hsa05204	Chemical carcinogenesis - DNA adducts	4/139	69/8111	0.030302	0.04608	0.014155	CYP1A1/PTGS2/CYP2E1/CYP3A4
hsa04972	Pancreatic secretion	5/139	102/8111	0.030509	0.046123	0.014168	PLA2G2A/SLC12A2/KCNQ1/CFTR/CHRM3
hsa04371	Apelin signaling pathway	6/139	138/8111	0.031012	0.046609	0.014318	NOS2/NOS1/CCND1/MAPK1/MYLK/AKT1
