# Supplementary material for: Exploring Molecular Mechanisms of Aloe barbadmsis Miller on Diphenoxylate-Induced Constipation in Mice
Source: Evid Based Complement Alternat Med. 2022 May 6;2022:6225758. doi: 10.1155/2022/6225758 (PMC9106447; doi:10.1155/2022/6225758)

Original images of Hematoxylin and eosin (H&E) staining in colon tissues

NC group-repeat 1

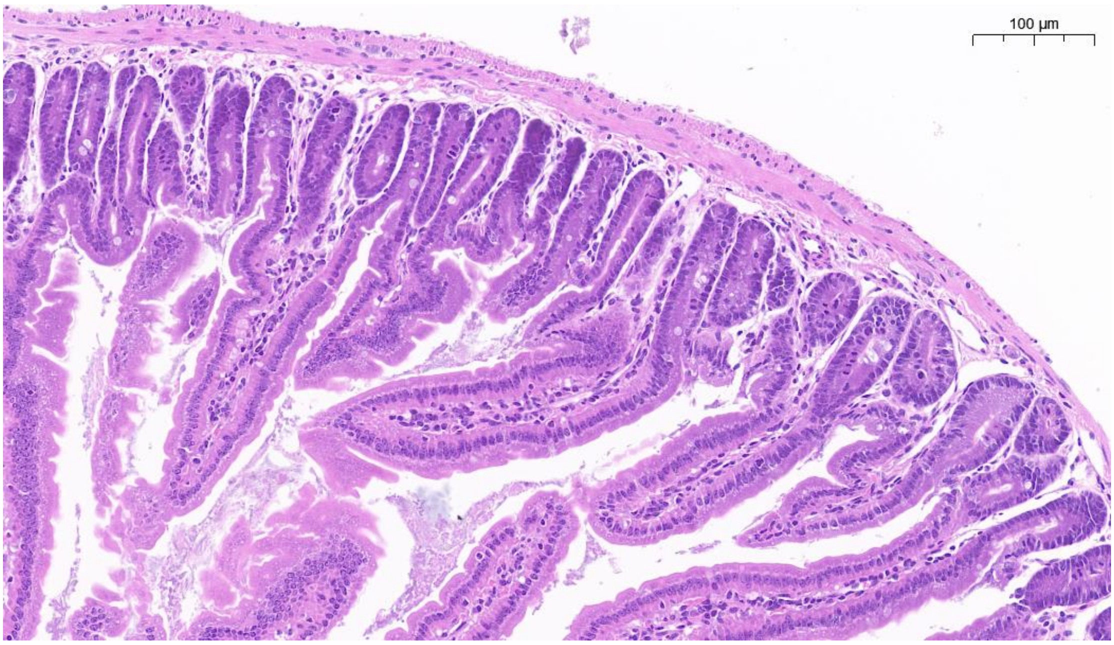

NC group- repeat 2

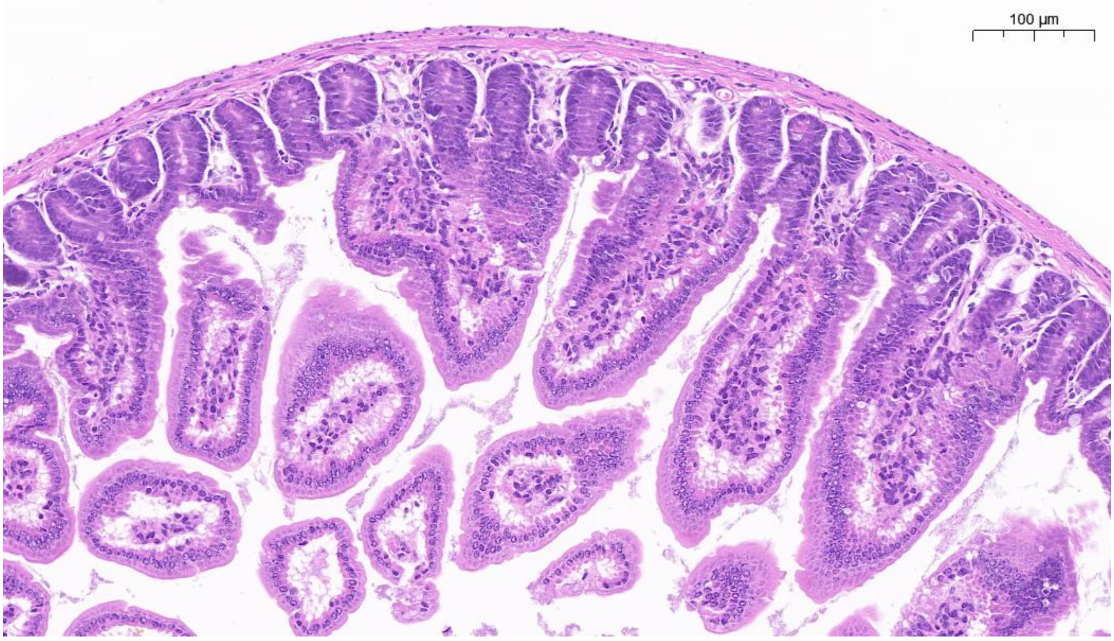

NC group- repeat 3

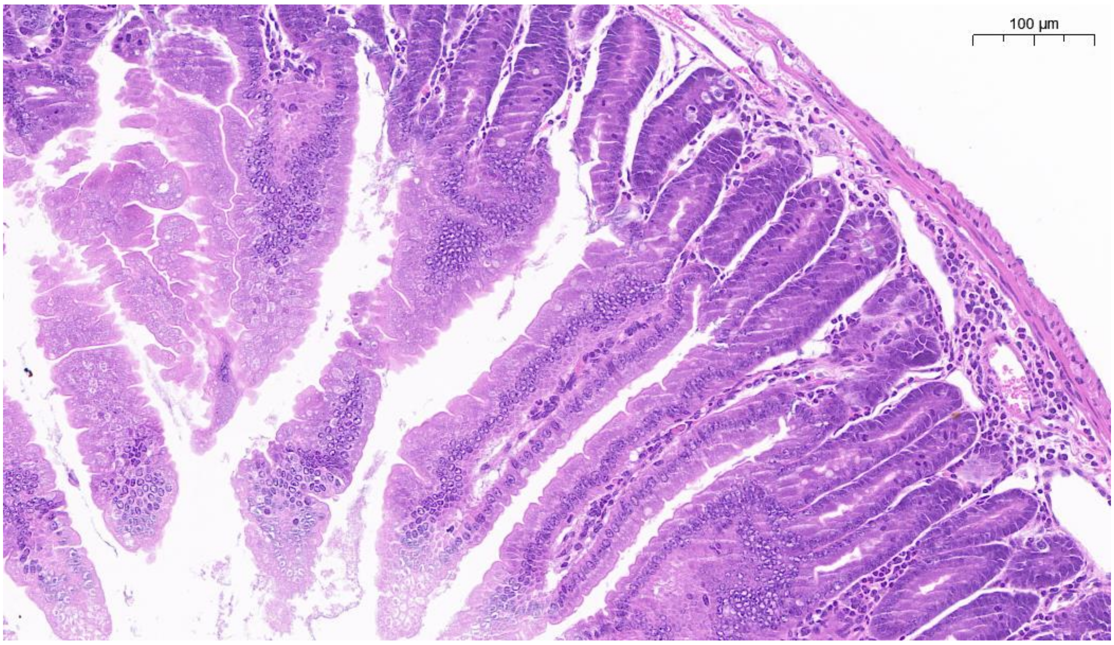

MC group- repeat 1

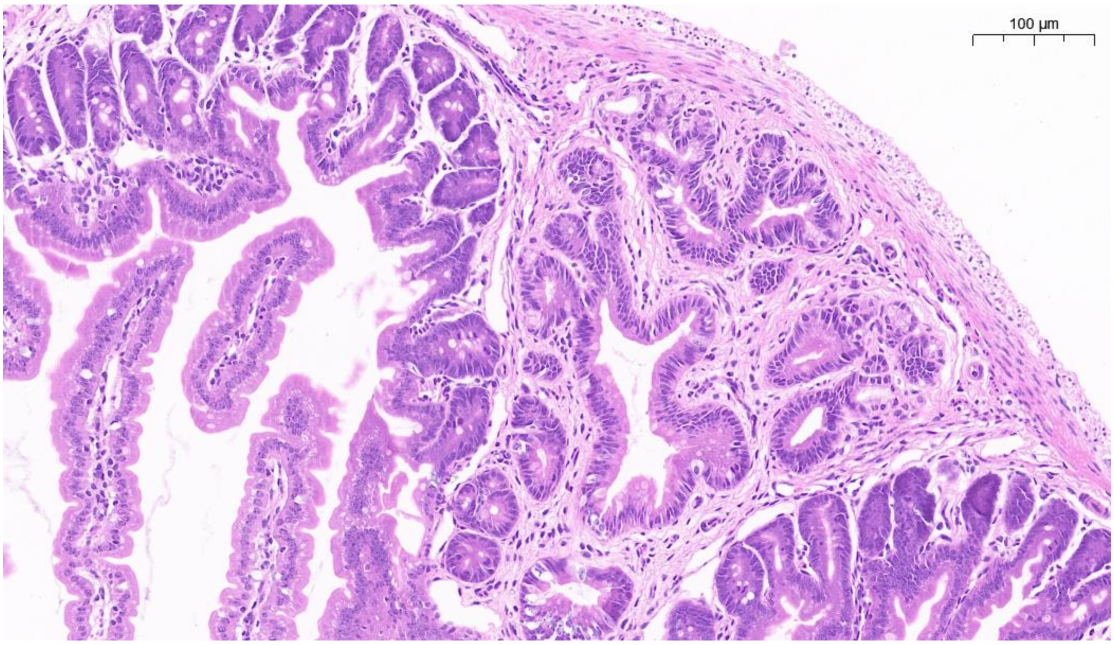

MC group- repeat 2

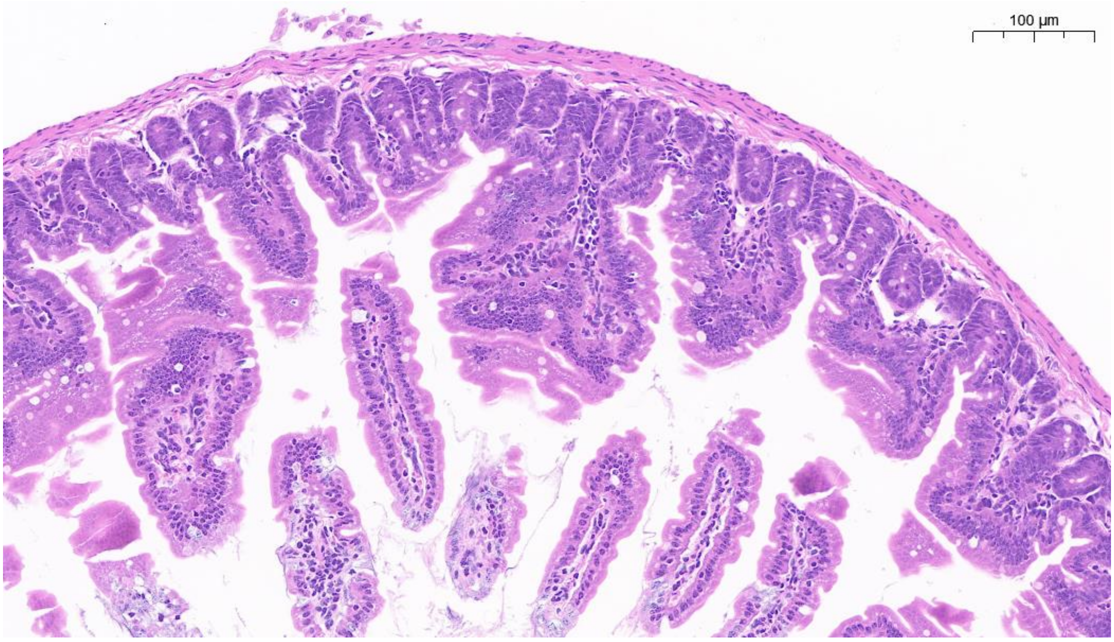

MC group- repeat 3

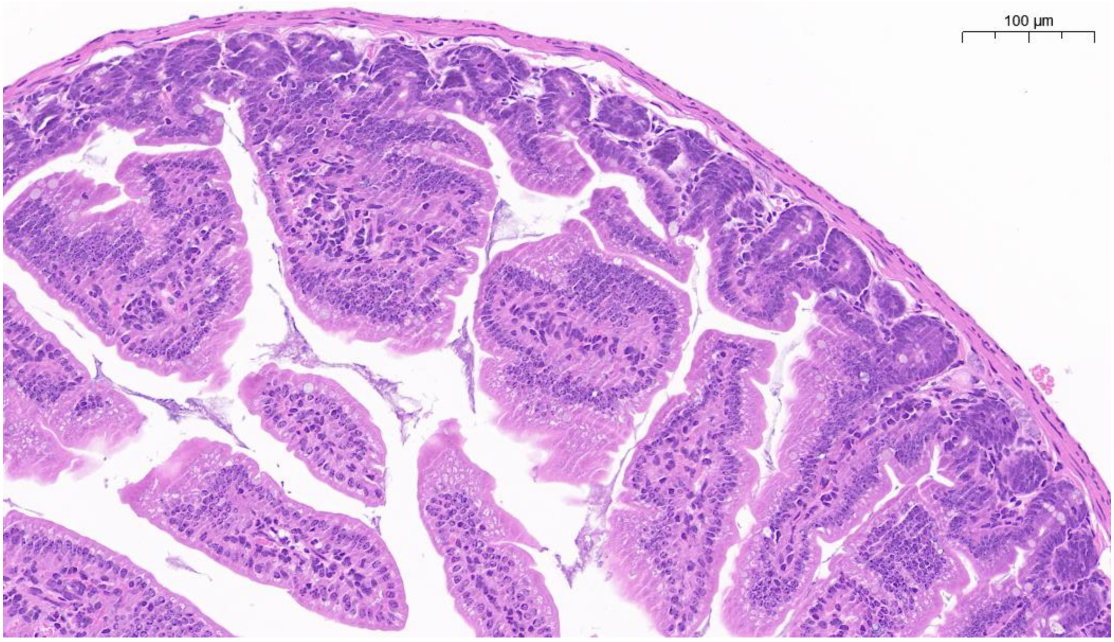

PC group- repeat 1

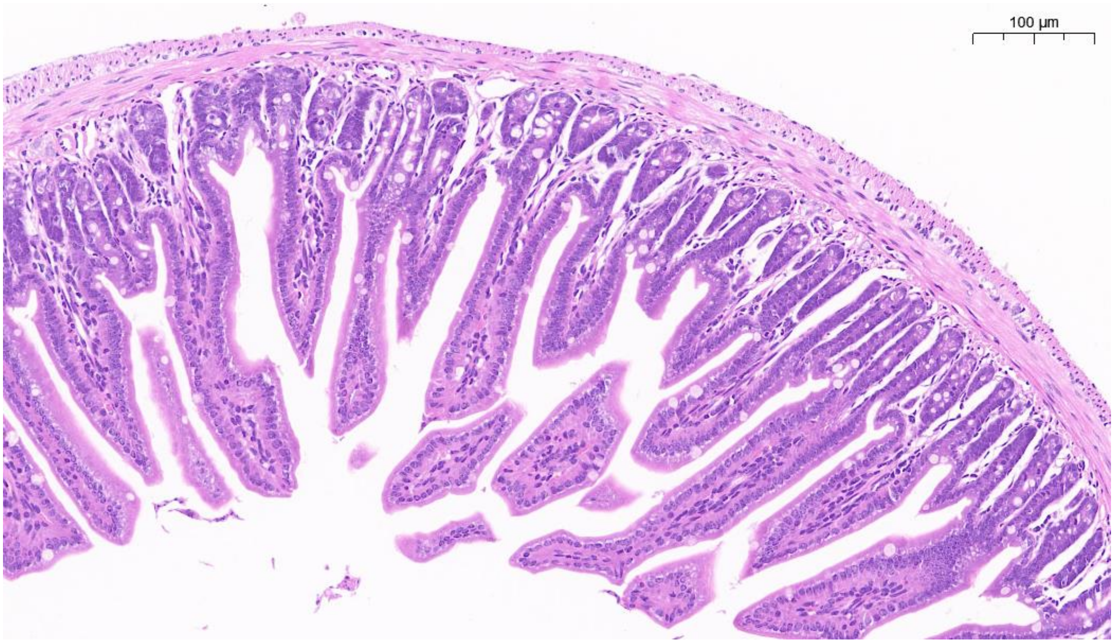

PC group- repeat 2

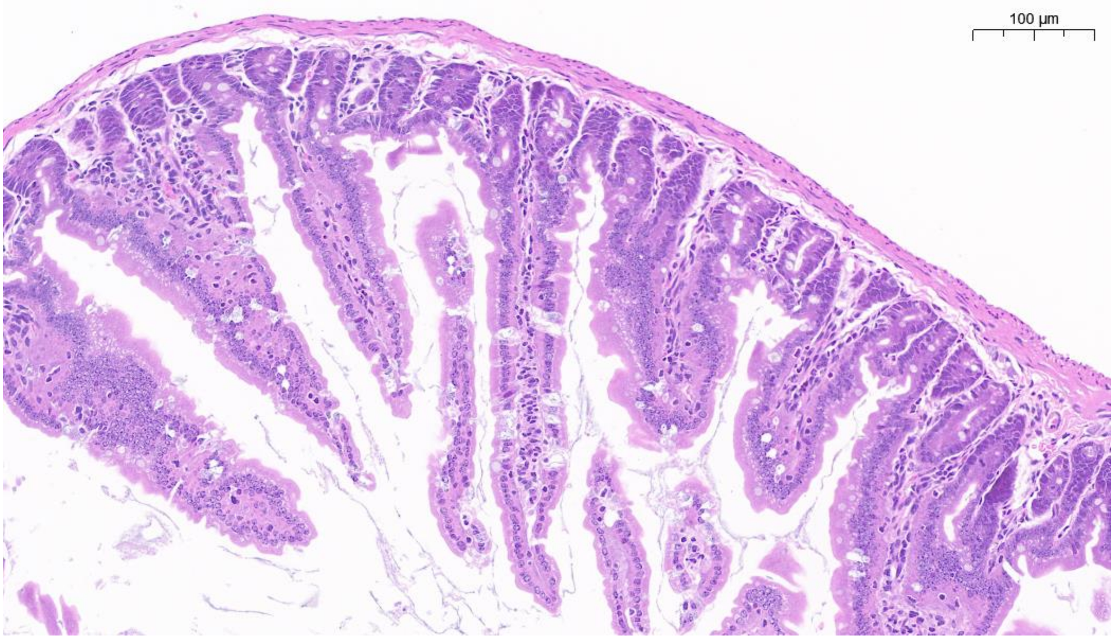

PC group- repeat 3

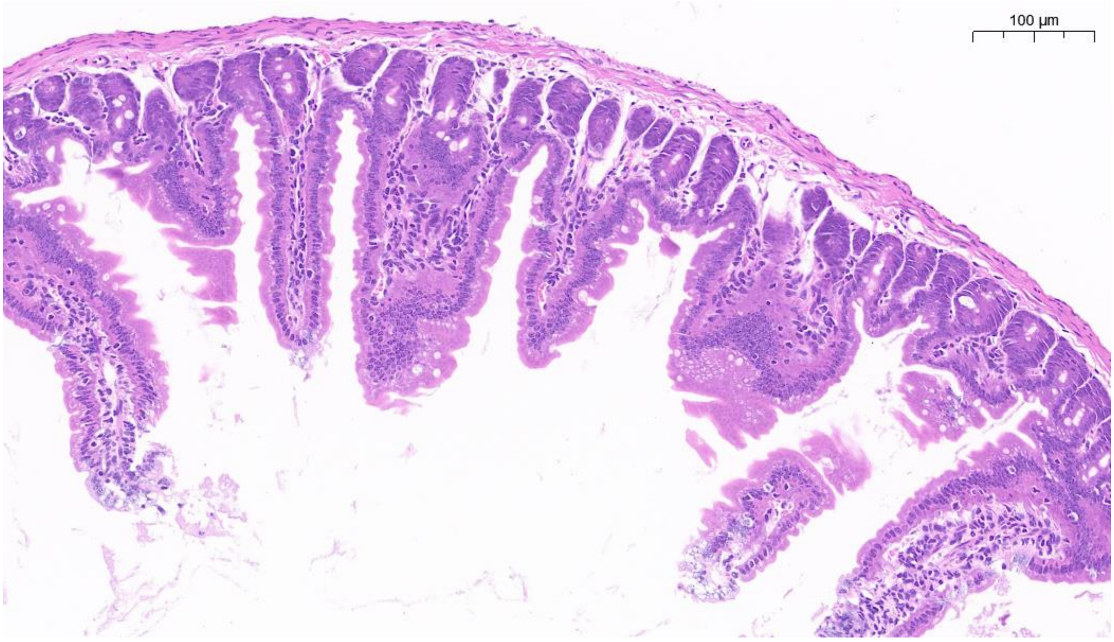

Aloe-L group- repeat 1

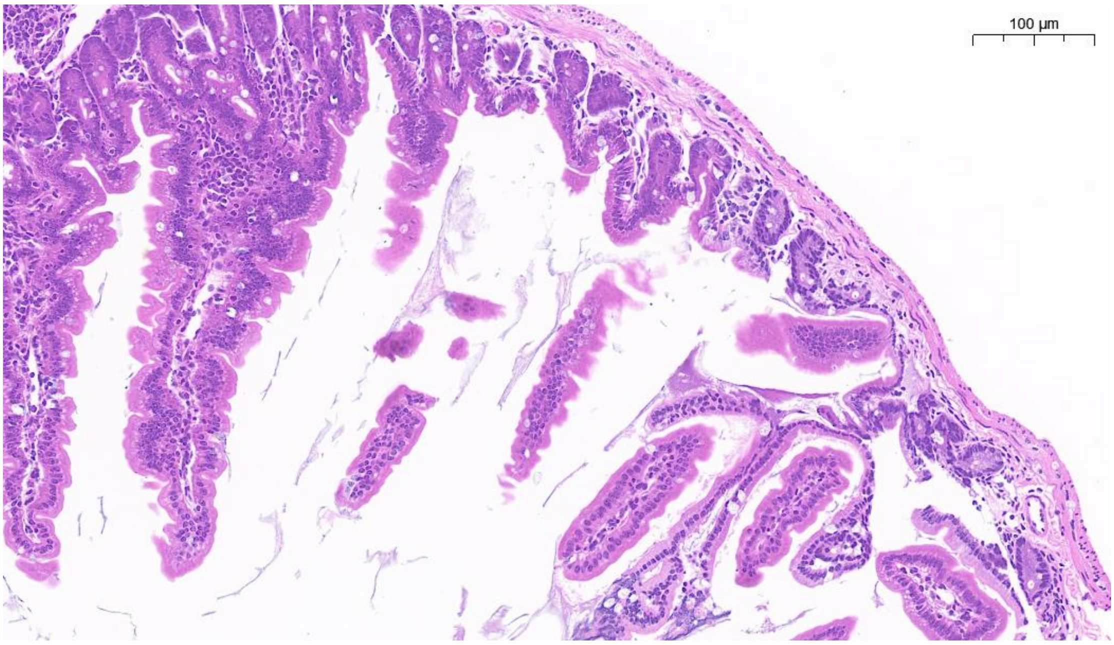

Aloe-L group- repeat 2

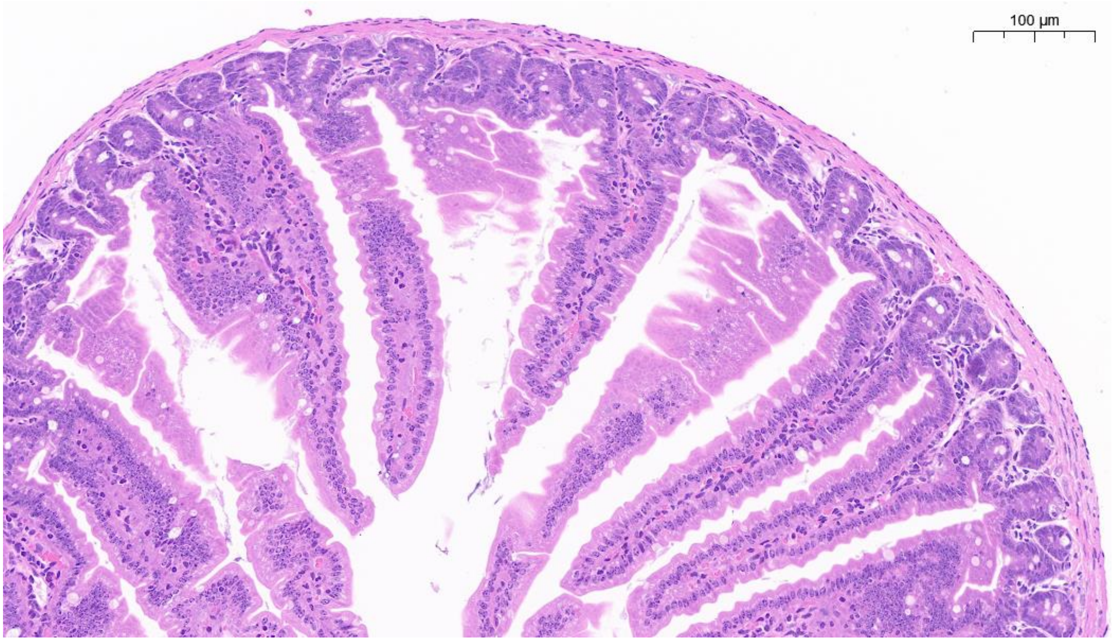

Aloe-L group- repeat 3

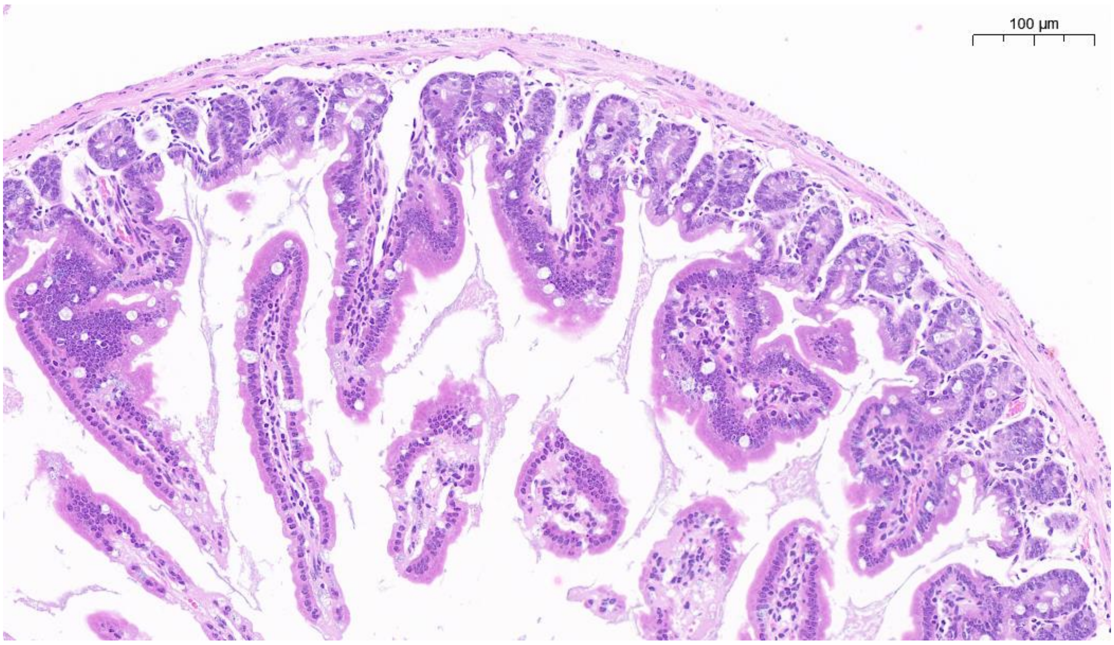

Aloe-M group- repeat 1

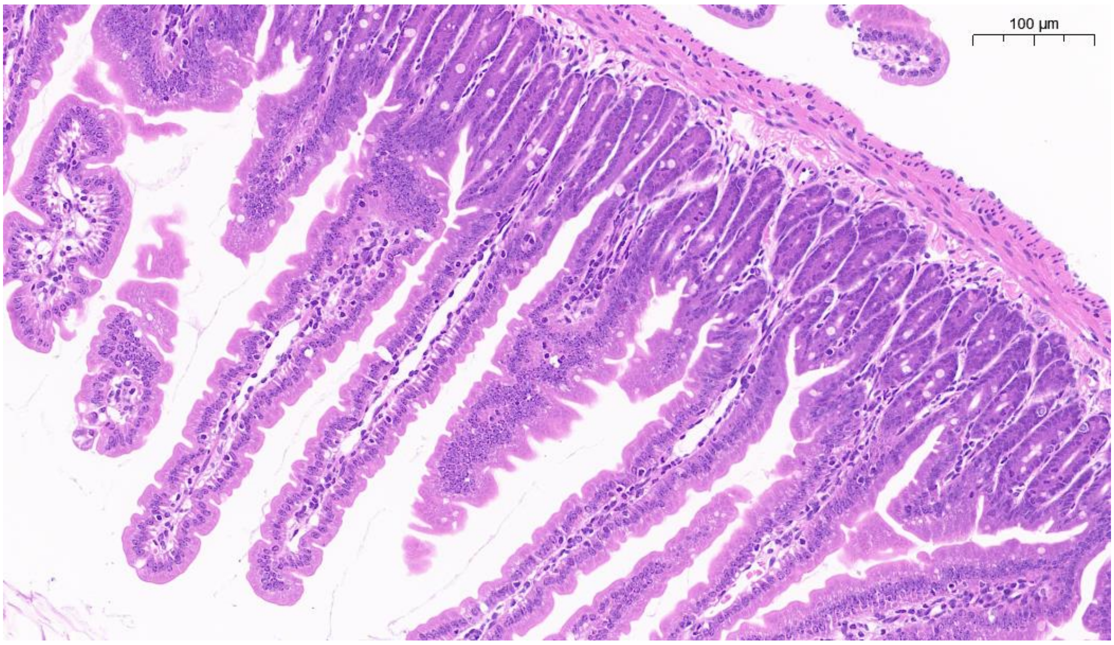

Aloe-M group- repeat 2

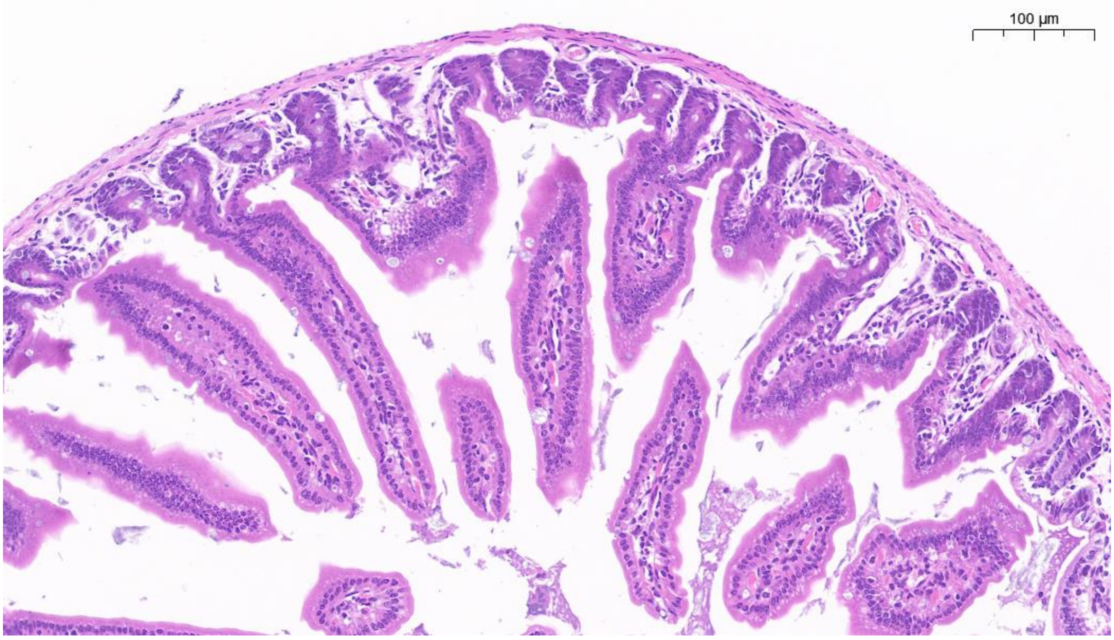

Aloe-M group- repeat 3

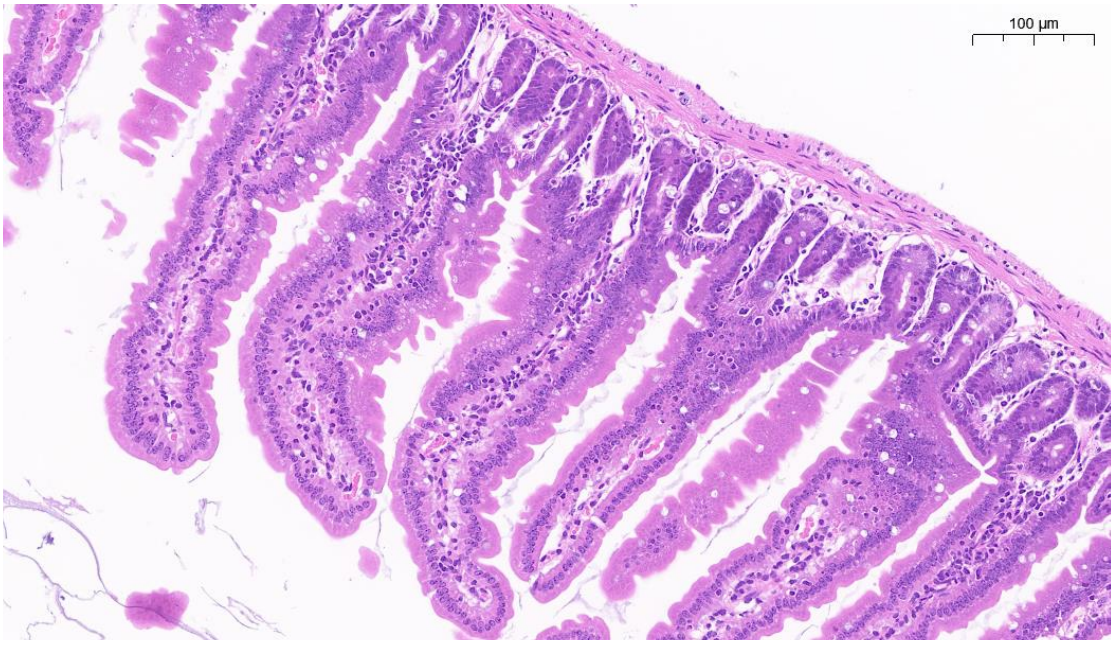

Aloe-H group- repeat 1

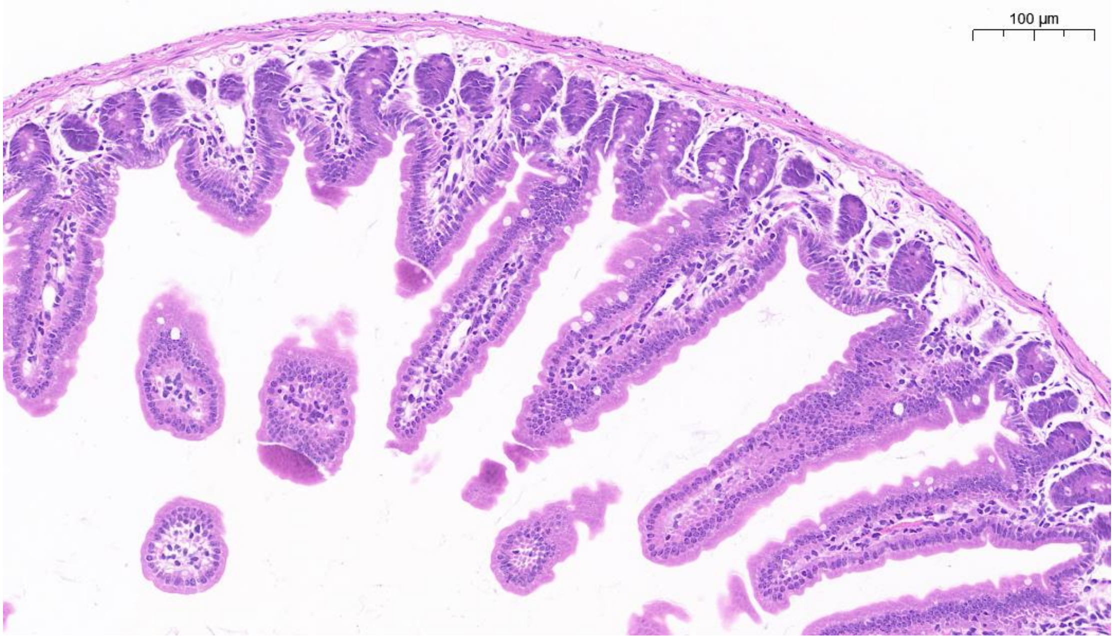

Aloe-H group- repeat 2

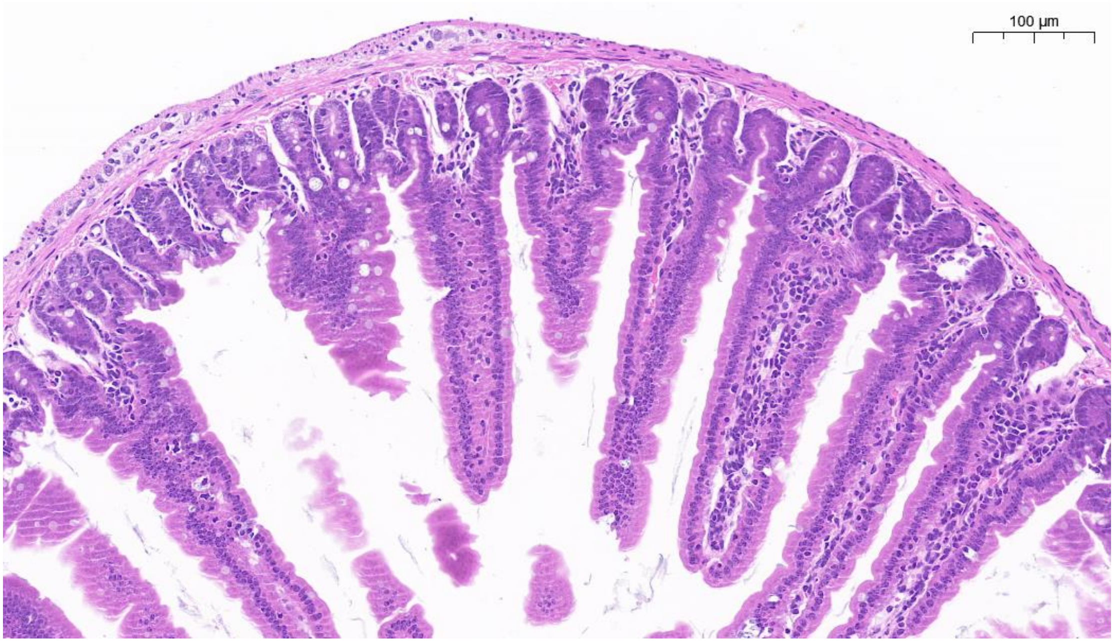

Aloe-H group- repeat 3

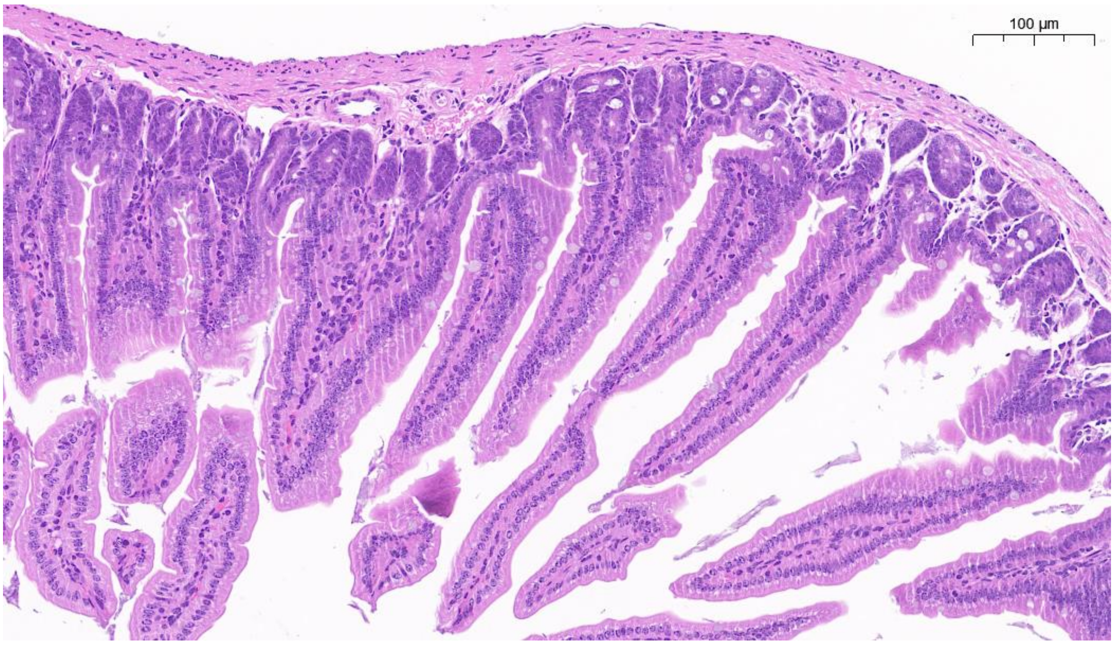

Supplement: Supplementary Materials — Table S1. Active ingredients of Aloe. Table S2. Potential targets related to active ingredients. Table S3. potential targets related to constipation. Table S4. Common targets related to active ingredients. Table S5. Table S5-1. Detailed information of BP enrichment of PPI network cluster 1 targets; Table S5-2. Detailed information of CC enrichment of PPI network cluster 1 targets; Table S5-3. Detailed information of MF enrichment of PPI network cluster 1 targets; Table S5-4. Detailed information of KEGG pathways enrichment of PPI network cluster 1 targets. Table S6. Table S6-1. Detailed information of BP enrichment of common targets; Table S6-2. Detailed information of CC enrichment of common targets; Table S6-3. Detailed information of MF enrichment of common targets; Table S6-4. Detailed information of KEGG pathways enrichment of common targets. Table S7. Original images of H&E staining in colon of three repeats in each group. Table S8. Raw data of 5-HT, SP, and VIP in serum and colon determined by ELISA kits. Table S9. Raw data of NF-κB p65, AKT, ERK, and JNK in colon determined by RT-PCR method. Table S10. Original images of ERK, JNK, AKT, and NF-κB p65 in colon of Western Blot, and its raw data quantification. [file 6225758.f1.zip › suppl table 1-10/Table S7 (1) (1).pdf]
