# Supplementary material for: Exploring Molecular Mechanisms of Aloe barbadmsis Miller on Diphenoxylate-Induced Constipation in Mice
Source: Evid Based Complement Alternat Med. 2022 May 6;2022:6225758. doi: 10.1155/2022/6225758 (PMC9106447; doi:10.1155/2022/6225758)

Raw data of ELISA

Raw data of 5-HT in serum (ng/ml) detected by ELISA mathod

groups	number	Original OD value of test sample	concentration (pg/ml)	concentration (ng/ml)
NC group	1	0.178	130875.95	130.88
NC group	2	0.23	92436.98	92.44
NC group	3	0.208	105356.76	105.36
NC group	4	0.175	134177.78	134.18
NC group	5	0.239	88072.88	88.07
NC group	6	0.216	100233.04	100.23
NC group	7	0.211	103371.01	103.37
NC group	8	0.226	94529.27	94.53
NC group	9	0.248	84127.81	84.13
NC group	10	0.22	97865.26	97.87
MC group	1	0.238	88535.90	88.54
MC group	2	0.29	69793.85	69.79
MC group	3	0.268	76583.07	76.58
MC group	4	0.235	89956.83	89.96
MC group	5	0.299	67374.12	67.37
MC group	6	0.276	73955.24	73.96
MC group	7	0.271	75574.40	75.57
MC group	8	0.286	70930.60	70.93
MC group	9	0.308	65128.30	65.13
MC group	10	0.28	72712.61	72.71
PC group	1	0.204	108134.82	108.13
PC group	2	0.205	107425.74	107.43
PC group	3	0.25	83301.95	83.30
PC group	4	0.225	95068.29	95.07
PC group	5	0.223	96166.33	96.17
PC group	6	0.236	89477.79	89.48
PC group	7	0.197	113392.75	113.39
PC group	8	0.285	71221.04	71.22
PC group	9	0.253	82095.24	82.10
PC group	10	0.235	89956.83	89.96
Aloe-L group	1	0.237	89004.17	89.00
Aloe-L group	2	0.25	83301.95	83.30
Aloe-L group	3	0.196	114188.80	114.19
Aloe-L group	4	0.321	62154.67	62.15
Aloe-L group	5	0.209	104685.85	104.69

Original OD value of standard sample	concentration of standard sample (pg/ml)
0.08	4000.00
0.10	2000.00
0.14	1000.00
0.23	500.00
0.40	250.00
1.18	0.00

POWER index form

Model of Harris: y=1/(a+bx^c)

Coefficient Data:

a = -0.00118662

b = 0.01119087

c = 0.82063084

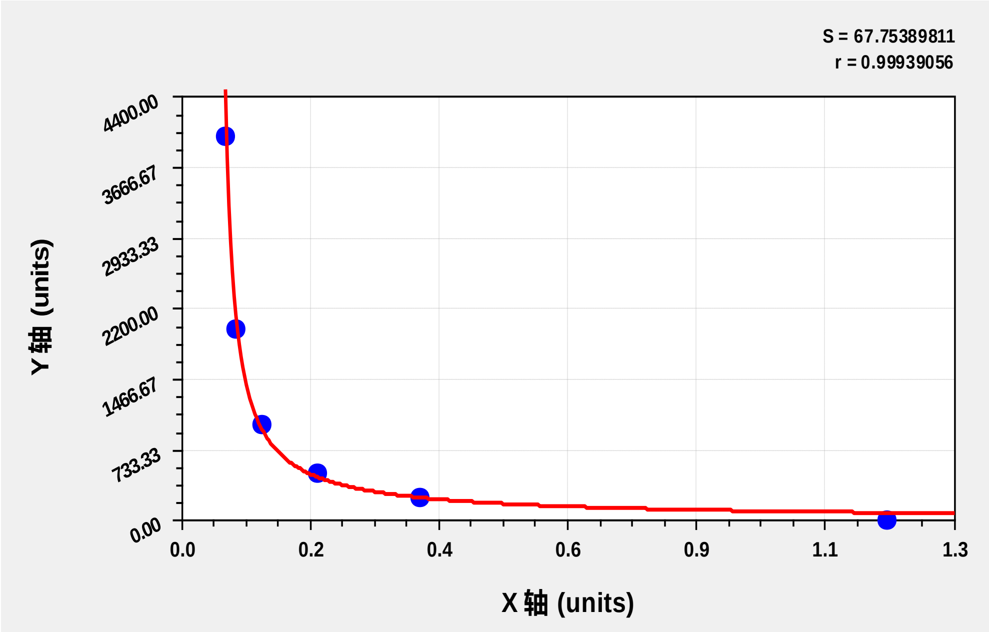

Note: The test sample was diluted 200 times.

groups	number	Original OD value of test sample	concentration (pg/ml)	concentration (ng/ml)
Aloe-L group	6	0.263	78330.18	78.33
Aloe-L group	7	0.197	113392.75	113.39
Aloe-L group	8	0.213	102090.95	102.09
Aloe-L group	9	0.263	78330.18	78.33
Aloe-L group	10	0.205	107425.74	107.43
Aloe-M group	1	0.238	88535.90	88.54
Aloe-M group	2	0.199	111835.59	111.84
Aloe-M group	3	0.211	103371.01	103.37
Aloe-M group	4	0.2	111073.96	111.07
Aloe-M group	5	0.218	99034.03	99.03
Aloe-M group	6	0.232	91427.53	91.43
Aloe-M group	7	0.226	94529.27	94.53
Aloe-M group	8	0.189	120113.28	120.11
Aloe-M group	9	0.26	79420.24	79.42
Aloe-M group	10	0.238	88535.90	88.54
Aloe-H group	1	0.218	99034.03	99.03
Aloe-H group	2	0.249	83712.69	83.71
Aloe-H group	3	0.224	95613.94	95.61
Aloe-H group	4	0.184	124763.25	124.76
Aloe-H group	5	0.18	128768.79	128.77
Aloe-H group	6	0.218	99034.03	99.03
Aloe-H group	7	0.254	81701.29	81.70
Aloe-H group	8	0.199	111835.59	111.84
Aloe-H group	9	0.195	114996.85	115.00
Aloe-H group	10	0.249	83712.69	83.71

Raw data of SP in serum (pg/ml) detected by ELISA mathod

groups	number	Original OD value of test sample	concentration (pg/ml)
NC group	1	0.308	1024.88
NC group	2	0.292	1146.40
NC group	3	0.296	1113.95
NC group	4	0.289	1171.73
NC group	5	0.364	727.20
NC group	6	0.316	971.59
NC group	7	0.364	727.20
NC group	8	0.31	1011.13
NC group	9	0.342	825.54
NC group	10	0.33	888.31
MC group	1	0.396	613.81
MC group	2	0.443	491.23
MC group	3	0.482	416.16
MC group	4	0.392	626.41
MC group	5	0.423	538.25
MC group	6	0.511	371.29
MC group	7	0.417	553.74
MC group	8	0.386	646.09
MC group	9	0.399	604.62
MC group	10	0.396	613.81
PC group	1	0.33	888.31
PC group	2	0.289	1171.73
PC group	3	0.328	899.50
PC group	4	0.418	551.11
PC group	5	0.323	928.48
PC group	6	0.358	752.11
PC group	7	0.341	830.50
PC group	8	0.443	491.23
PC group	9	0.361	739.49
PC group	10	0.381	663.23
Aloe-L group	1	0.35	787.48
Aloe-L group	2	0.39	632.87
Aloe-L group	3	0.351	782.92
Aloe-L group	4	0.753	176.06
Aloe-L group	5	0.405	586.88

Original OD value of standard sample	concentration of standard sample (pg/ml)
0.129	1000
0.183	333.33
0.31	111.11
0.488	37.04
0.728	12.35
1.117	0

POWER index form

Model of Harris: y=1/(a+bx^c)

Coefficient Data:

a = -0.00114323

b = 0.09845742

c = 1.86881017

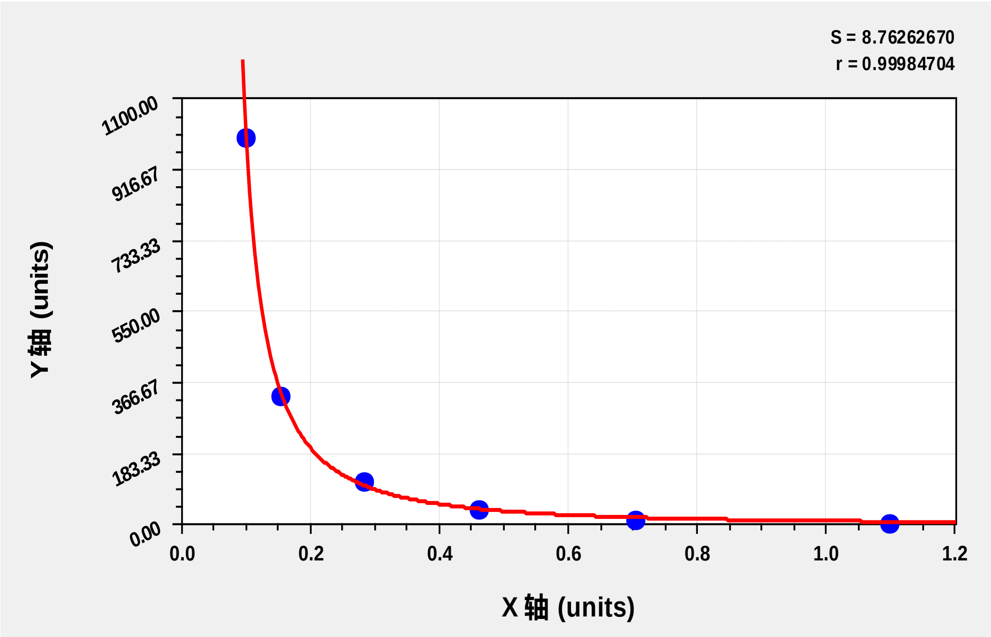

Note: The test sample was diluted 10 times.

groups	number	Original OD value of test sample	concentration (pg/ml)
Aloe-L group	6	0.398	607.66
Aloe-L group	7	0.302	1067.94
Aloe-L group	8	0.36	743.66
Aloe-L group	9	0.316	971.59
Aloe-L group	10	0.313	991.05
Aloe-M group	1	0.332	877.33
Aloe-M group	2	0.344	815.75
Aloe-M group	3	0.404	589.78
Aloe-M group	4	0.343	820.62
Aloe-M group	5	0.368	711.30
Aloe-M group	6	0.302	1067.94
Aloe-M group	7	0.401	598.62
Aloe-M group	8	0.386	646.09
Aloe-M group	9	0.447	482.60
Aloe-M group	10	0.346	806.15
Aloe-H group	1	0.386	646.09
Aloe-H group	2	0.308	1024.88
Aloe-H group	3	0.286	1197.96
Aloe-H group	4	0.402	595.65
Aloe-H group	5	0.422	540.78
Aloe-H group	6	0.463	450.32
Aloe-H group	7	0.485	411.14
Aloe-H group	8	0.327	905.18
Aloe-H group	9	0.376	681.10
Aloe-H group	10	0.307	1031.86

Raw data of VIP in serum (pg/ml) detected by ELISA mathod

groups	number	Original OD value of test sample	concentration (pg/ml)
NC group	1	0.385	337.92
NC group	2	0.39	332.20
NC group	3	0.367	360.13
NC group	4	0.452	273.79
NC group	5	0.401	320.22
NC group	6	0.48	253.29
NC group	7	0.439	284.39
NC group	8	0.416	305.12
NC group	9	0.336	405.45
NC group	10	0.342	395.87
MC group	1	0.328	418.91
MC group	2	0.254	597.92
MC group	3	0.301	471.15
MC group	4	0.262	572.07
MC group	5	0.274	537.00
MC group	6	0.307	458.52
MC group	7	0.252	604.72
MC group	8	0.291	493.70
MC group	9	0.299	475.50
MC group	10	0.286	505.74
PC group	1	0.289	498.45
PC group	2	0.283	513.23
PC group	3	0.496	242.81
PC group	4	0.329	417.18
PC group	5	0.356	375.09
PC group	6	0.309	454.45
PC group	7	0.379	345.03
PC group	8	0.35	383.74
PC group	9	0.413	308.03
PC group	10	0.25	611.67
Aloe-L group	1	0.291	493.70
Aloe-L group	2	0.332	412.07
Aloe-L group	3	0.346	389.72
Aloe-L group	4	0.302	469.00
Aloe-L group	5	0.364	364.10

Original OD value of standard sample	concentration of standard sample (pg/ml)
0.084	500
0.133	166.67
0.302	55.56
0.573	18.52
0.894	6.17
1.401	0

POWER index form

Model of Harris: y=1/(a+bx^c)
Coefficient Data:
a = -0.00340299
b = 0.10259185
c = 1.18843612

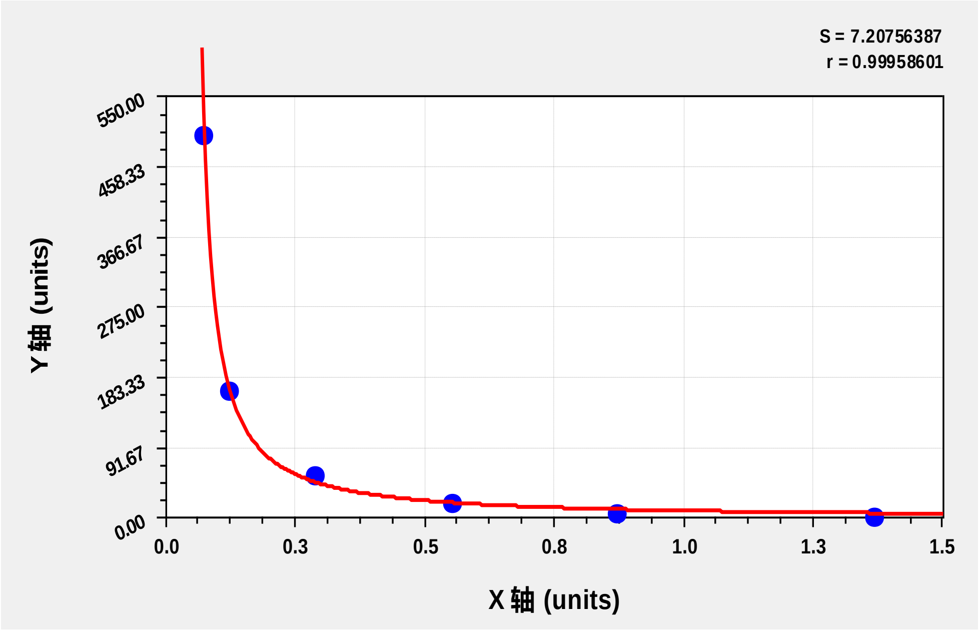

Note: The test sample was diluted 10 times.

groups	number	Original OD value of test sample	concentration （pg/ml)
Aloe-L group	6	0.348	386.71
Aloe-L group	7	0.313	446.51
Aloe-L group	8	0.269	551.11
Aloe-L group	9	0.303	466.87
Aloe-L group	10	0.373	352.43
Aloe-M group	1	0.358	372.28
Aloe-M group	2	0.341	397.44
Aloe-M group	3	0.29	496.06
Aloe-M group	4	0.334	408.74
Aloe-M group	5	0.324	425.95
Aloe-M group	6	0.41	311.00
Aloe-M group	7	0.421	300.38
Aloe-M group	8	0.247	622.38
Aloe-M group	9	0.31	452.44
Aloe-M group	10	0.318	436.94
Aloe-H group	1	0.353	379.37
Aloe-H group	2	0.398	323.40
Aloe-H group	3	0.339	400.61
Aloe-H group	4	0.411	310.00
Aloe-H group	5	0.321	431.38
Aloe-H group	6	0.34	399.02
Aloe-H group	7	0.3	473.32
Aloe-H group	8	0.256	591.25
Aloe-H group	9	0.389	333.33
Aloe-H group	10	0.357	373.68

Raw data of 5-HT in colon (ng/ml)

groups	number	Original OD value of test sample	concentration (pg/ml)	concentration (ng/ml)
NC group	1	0.705	35488.84	35.49
NC group	2	0.78	33743.39	33.74
NC group	3	0.814	33045.36	33.05
NC group	4	0.591	38881.39	38.88
NC group	5	0.522	41578.38	41.58
NC group	6	0.793	33470.36	33.47
MC group	1	0.555	40211.67	40.21
MC group	2	0.344	53184.74	53.18
MC group	3	0.5	42582.16	42.58
MC group	4	0.299	58294.48	58.29
MC group	5	0.456	44861.24	44.86
MC group	6	0.503	42440.46	42.44
PC group	1	0.657	36788.68	36.79
PC group	2	0.502	42487.51	42.49
PC group	3	0.536	40979.59	40.98
PC group	4	0.396	48745.18	48.75
PC group	5	0.511	42070.20	42.07
PC group	6	0.385	49584.87	49.58
Aloe-L group	1	0.51	42115.89	42.12
Aloe-L group	2	0.563	39902.62	39.90
Aloe-L group	3	0.453	45031.88	45.03
Aloe-L group	4	0.52	41666.36	41.67
Aloe-L group	5	0.432	46289.75	46.29
Aloe-L group	6	0.464	44416.48	44.42
Aloe-M group	1	0.715	35237.57	35.24
Aloe-M group	2	0.509	42161.74	42.16
Aloe-M group	3	0.362	51503.79	51.50
Aloe-M group	4	0.445	45497.65	45.50
Aloe-M group	5	0.655	36846.51	36.85
Aloe-M group	6	0.364	51327.33	51.33
Aloe-H group	1	0.595	38742.66	38.74
Aloe-H group	2	0.436	46041.19	46.04
Aloe-H group	3	0.426	46671.01	46.67
Aloe-H group	4	0.329	54728.06	54.73
Aloe-H group	5	0.412	47602.36	47.60
Aloe-H group	6	0.362	51503.79	51.50

Original OD value of standard sample	concentration of standard sample (pg/ml)
0.056	4000
0.065	2000
0.09	1000
0.164	500
0.383	250
1.52	0

POWER index form

Model of Harris: y=1/(a+bx^c)

Coefficient Data:

a = -0.00605870

b = 0.01273348

c = 0.24355175

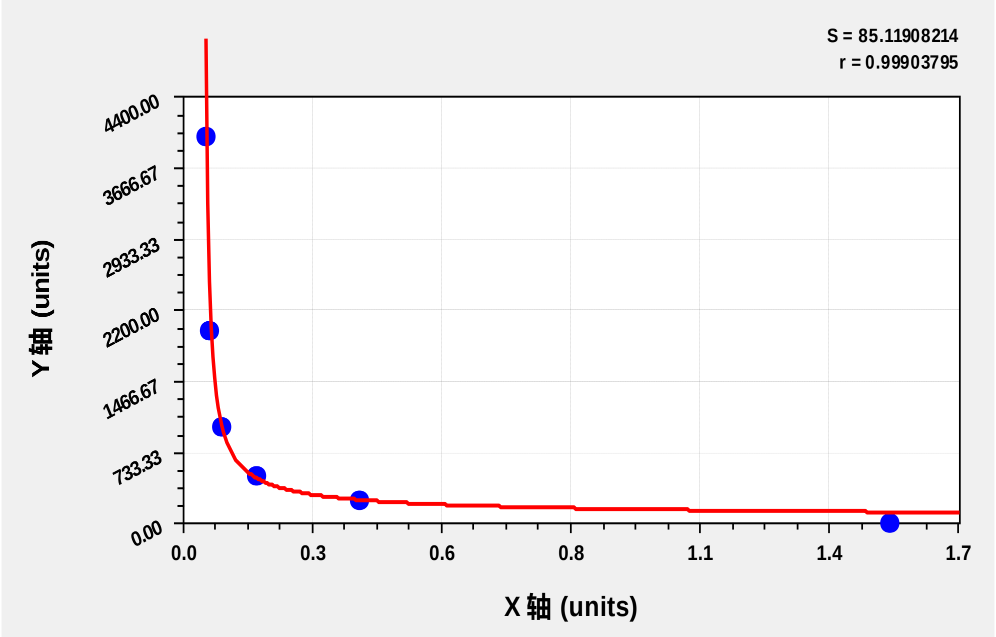

Note: The test sample was diluted 200 times.

Raw data of SP in colon (pg/ml)

groups	number	Original OD value of test sample	concentration (pg/ml)
NC group	1	0.294	102.57
NC group	2	0.295	101.75
NC group	3	0.347	71.67
NC group	4	0.302	96.35
NC group	5	0.298	99.36
NC group	6	0.286	109.63
MC group	1	0.345	72.50
MC group	2	0.37	63.30
MC group	3	0.338	75.56
MC group	4	0.371	62.98
MC group	5	0.353	69.29
MC group	6	0.384	59.08
PC group	1	0.326	81.44
PC group	2	0.317	86.47
PC group	3	0.309	91.48
PC group	4	0.314	88.29
PC group	5	0.325	81.97
PC group	6	0.309	91.48
Aloe-L group	1	0.323	83.05
Aloe-L group	2	0.336	76.49
Aloe-L group	3	0.311	90.17
Aloe-L group	4	0.338	75.56
Aloe-L group	5	0.309	91.48
Aloe-L group	6	0.319	85.30
Aloe-M group	1	0.319	85.30
Aloe-M group	2	0.334	77.43
Aloe-M group	3	0.312	89.54
Aloe-M group	4	0.33	79.39
Aloe-M group	5	0.297	100.14
Aloe-M group	6	0.317	86.47
Aloe-H group	1	0.298	99.36
Aloe-H group	2	0.315	87.67
Aloe-H group	3	0.308	92.14
Aloe-H group	4	0.318	85.88
Aloe-H group	5	0.312	89.54
Aloe-H group	6	0.297	100.14

Original OD value of standard sample	concentration of standard sample (pg/ml)
0.18	1000
0.206	333.33
0.317	111.11
0.44	37.04
0.757	12.35
1.074	0

POWER index form

Model of Harris: y=1/(a+bx^c)

Coefficient Data:

a = -0.01114917

b = 0.08088746

c = 1.10553127

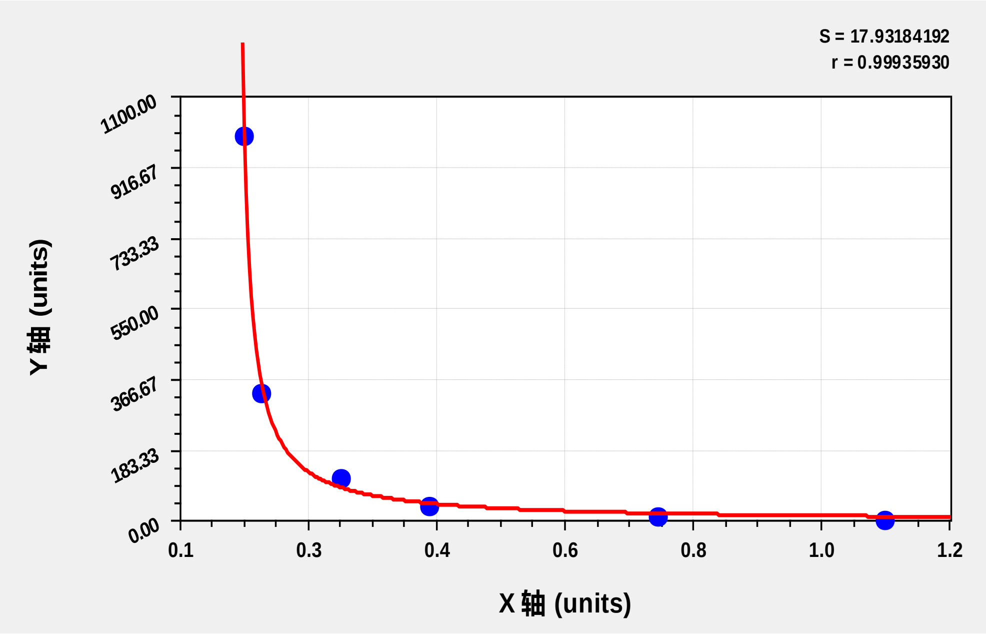

Raw data of VIP in colon (pg/ml)

groups	number	Original OD value of test sample	concentration （pg/ml)
NC group	1	0.86	10.94
NC group	2	0.776	12.68
NC group	3	0.649	16.43
NC group	4	0.764	12.97
NC group	5	0.767	12.90
NC group	6	0.859	10.95
MC group	1	0.718	14.19
MC group	2	0.694	14.91
MC group	3	0.736	13.69
MC group	4	0.665	15.86
MC group	5	0.656	16.18
MC group	6	0.585	19.11
PC group	1	0.824	11.63
PC group	2	0.714	14.31
PC group	3	0.837	11.37
PC group	4	0.755	13.20
PC group	5	0.87	10.76
PC group	6	0.79	12.36
Aloe-L group	1	0.754	13.22
Aloe-L group	2	0.777	12.66
Aloe-L group	3	0.718	14.19
Aloe-L group	4	0.702	14.66
Aloe-L group	5	0.716	14.25
Aloe-L group	6	0.765	12.95
Aloe-M group	1	0.861	10.92
Aloe-M group	2	0.702	14.66
Aloe-M group	3	0.763	13.00
Aloe-M group	4	0.672	15.62
Aloe-M group	5	0.781	12.57
Aloe-M group	6	0.898	10.28
Aloe-H group	1	0.82	11.71
Aloe-H group	2	0.743	13.51
Aloe-H group	3	0.718	14.19
Aloe-H group	4	0.851	11.10
Aloe-H group	5	0.838	11.35
Aloe-H group	6	0.818	11.75

Original OD value of standard sample	concentration of standard sample （pg/ml)
0.088	500
0.147	166.67
0.302	55.56
0.604	18.52
0.943	6.17
1.36	0

POWER index form

Model of Harris: =1/(a+bx^c)

Coefficient Data:

a = -0.00171165

b = 0.11529487

c = 1.41371257

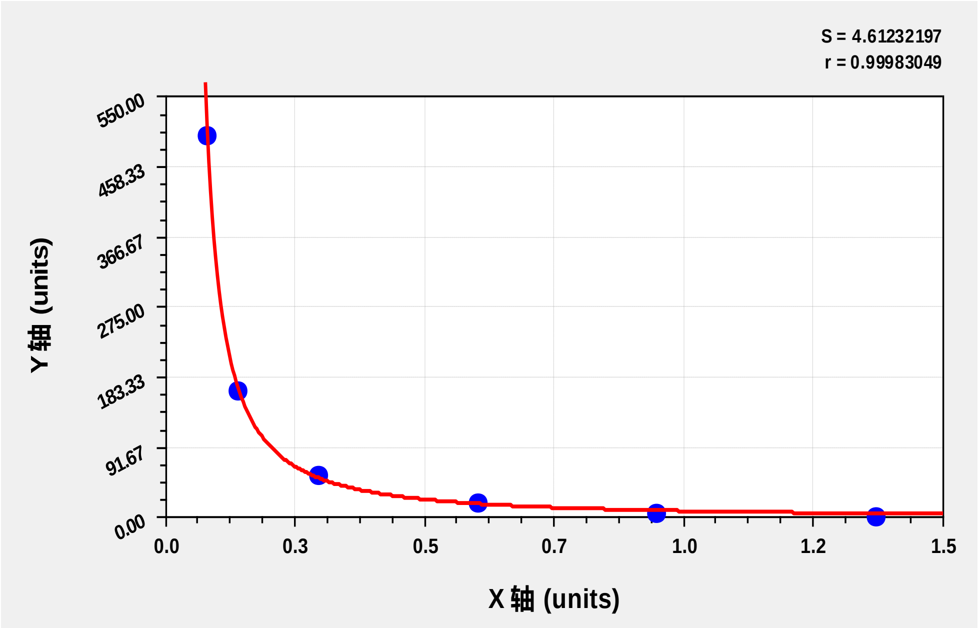

Supplement: Supplementary Materials — Table S1. Active ingredients of Aloe. Table S2. Potential targets related to active ingredients. Table S3. potential targets related to constipation. Table S4. Common targets related to active ingredients. Table S5. Table S5-1. Detailed information of BP enrichment of PPI network cluster 1 targets; Table S5-2. Detailed information of CC enrichment of PPI network cluster 1 targets; Table S5-3. Detailed information of MF enrichment of PPI network cluster 1 targets; Table S5-4. Detailed information of KEGG pathways enrichment of PPI network cluster 1 targets. Table S6. Table S6-1. Detailed information of BP enrichment of common targets; Table S6-2. Detailed information of CC enrichment of common targets; Table S6-3. Detailed information of MF enrichment of common targets; Table S6-4. Detailed information of KEGG pathways enrichment of common targets. Table S7. Original images of H&E staining in colon of three repeats in each group. Table S8. Raw data of 5-HT, SP, and VIP in serum and colon determined by ELISA kits. Table S9. Raw data of NF-κB p65, AKT, ERK, and JNK in colon determined by RT-PCR method. Table S10. Original images of ERK, JNK, AKT, and NF-κB p65 in colon of Western Blot, and its raw data quantification. [file 6225758.f1.zip › suppl table 1-10/Table S8 (1) (1).pdf]
